# Unprecedented selective homogeneous cobalt-catalysed reductive alkoxylation of cyclic imides under mild conditions†
†Electronic supplementary information (ESI) available: General information concerning experimental procedures, additional tables, figures, schemes, characterization data and NMR spectra of the isolated compounds are available. See DOI: 10.1039/c7sc01175j



**DOI:** 10.1039/c7sc01175j

**Published:** 2017-06-12

**Authors:** Jose R. Cabrero-Antonino, Rosa Adam, Veronica Papa, Mattes Holsten, Kathrin Junge, Matthias Beller

**Affiliations:** a Leibniz-Institut für Katalyse e.V. an der Universität Rostock , Albert-Einstein-Straße 29a , 18059 Rostock , Germany . Email: matthias.beller@catalysis.de

## Abstract

The first general and efficient non-noble metal-catalysed reductive C2-alkoxylation of cyclic imides (phthalimides and succinimides) is presented. Crucial for the success is the use of [Co(BF_4_)_2_·6H_2_O/triphos (**L1**)] combination and no external additives are required. Using the optimal cobalt-system, the hydrogenation of the aromatic ring of the parent phthalimide is avoided and only one of the carbonyl groups is selectively functionalized. The resulting products, *N*- and aryl-ring substituted 3-alkoxy-2,3-dihydro-1*H*-isoindolin-1-one and *N*-substituted 3-alkoxy-pyrrolidin-2-one derivatives, are prepared under mild conditions in good to excellent isolated yields. Intramolecular reductive couplings can also be performed affording tricyclic compounds in a one-step process. The present protocol opens the way to the development of new base-metal processes for the straightforward synthesis of functionalized *N*-heterocyclic compounds of pharmaceutical and biological interest.

## Abstract 2

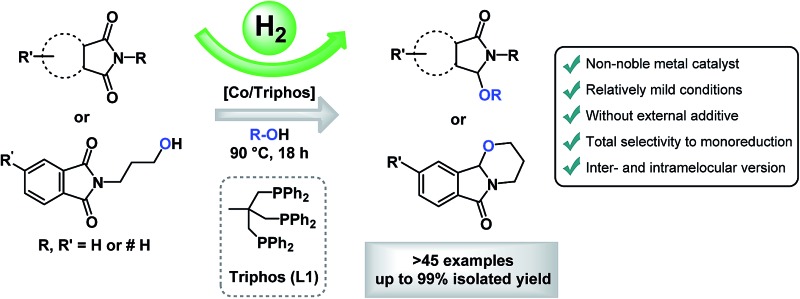
The first general and efficient non-noble metal-catalysed reductive C2-alkoxylation of cyclic imides (phthalimides and succinimides) is presented. Crucial for the success is the use of [Co(BF_4_)_2_·6H_2_O/triphos (**L1**)] combination and no external additives are required.

## Introduction

Catalytic reductive transformations of carboxylic acid derivatives are of importance and a current hot topic in catalysis.^1^ These reactions are already applied in industry for the formation of bulk products and intermediates. Moreover, they offer interesting possibilities for valorization of biomass-derived building blocks and to apply new strategies in organic synthesis. Hence, the design of improved catalysts, and also the development of new methodologies for the selective reduction of this class of compounds, continues to attract the interest of academic and industrial researchers.

**Fig. 1:**
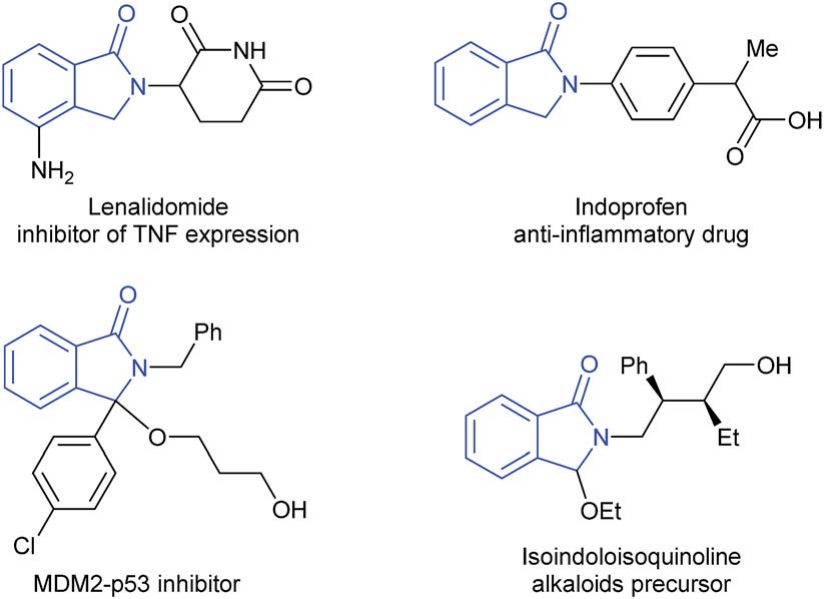
Examples of relevant isoindolinone derivatives.

Cyclic imides, and in particular phthalimides,^2^ are an important type of carboxylic acid derivative and several of these compounds show interesting biological activities. Among the possible products obtained from the reduction of phthalimides, isoindolinones and substituted derivatives are the most desired as they are valuable scaffolds in pharmaceuticals and agrochemicals, as well as relevant building blocks for organic synthesis (Fig. 1).^3^

As a consequence, in the last years several – often multi-step – organic methodologies have been reported for their synthesis.^3q,4^ Clearly, the selective mono-reduction of readily available phthalimides represents the most suitable and direct approximation to these compounds. Traditionally, procedures for this reduction required the use of over-stoichiometric amounts of Zn or Sn in the presence of strong acids or organometallic hydrides (NaBH_4_, B_2_H_6_ and LiAlH_4_). Despite the usefulness of these methods on laboratory scale, they have drawbacks due to their limited functional group tolerance, the generation of over-reduction products and significant amounts of waste.^3f,t,4g,k^

**Fig. 2:**
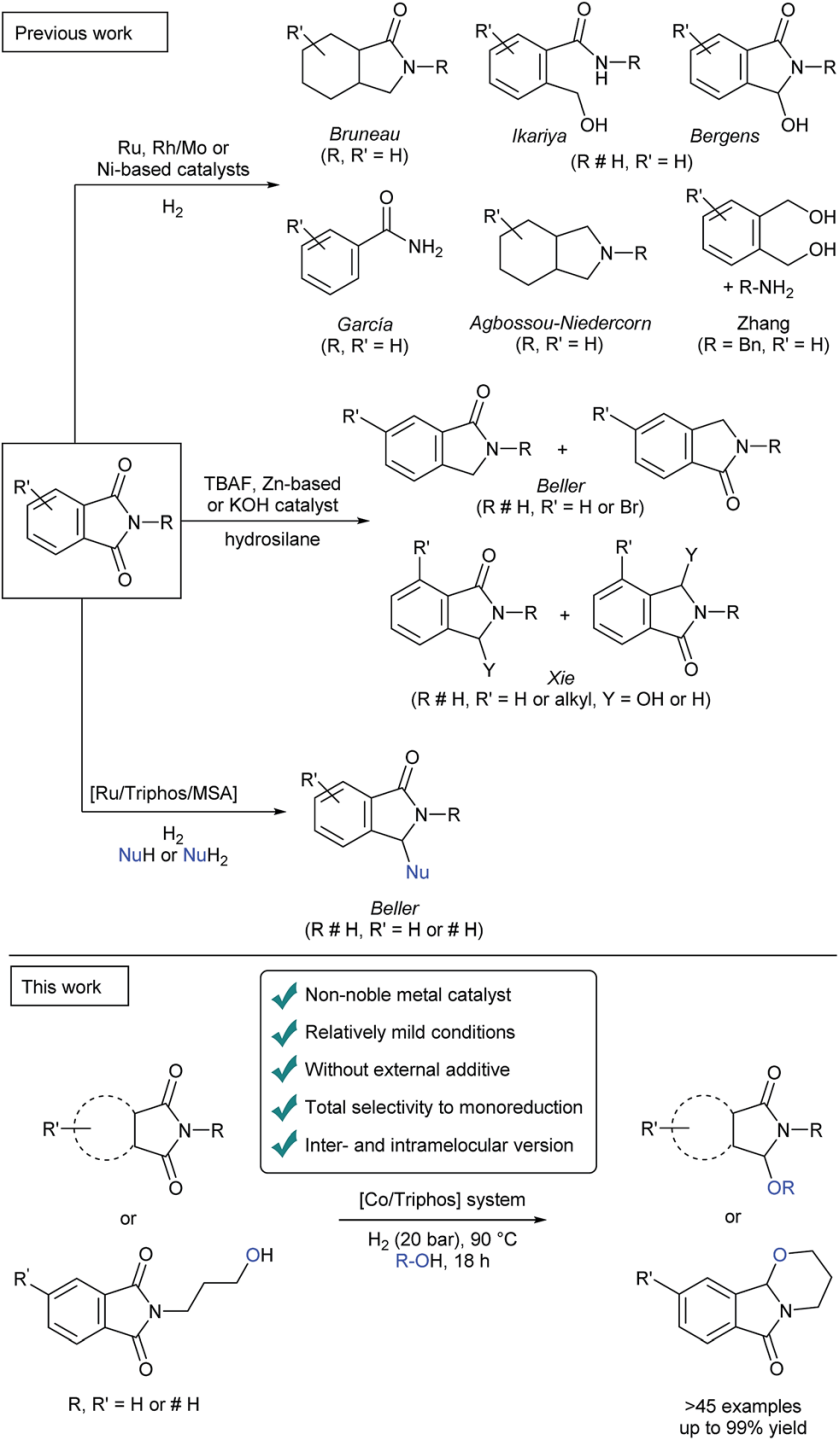
(Up) Described examples of homogeneous catalytic reduction of phthalimides using hydrogen or silanes as reductor. (Bottom) General [Co/triphos]-catalysed inter- and intramolecular selective reductive alkoxylation of cyclic imides (this work). (Bn = benzyl).

As a greener approach to the reduction of phthalimides, hydrogenations using heterogeneous catalysts have been applied (*i.e.* RANEY® nickel), although they require harsh reaction conditions.^1d,f^ To overcome these limitations, in the last decade also methodologies based on molecularly-defined complexes have been developed proceeding at milder reaction conditions (Fig. 2). After the original report by Patton and Drago dealing with the hydrogenation of *N*-methylsuccinimide with a ruthenium catalyst,^5^ alternative procedures were reported by the groups of Bruneau,^6^ Ikariya,^7^ Bergens,^8^ García,^9^ Agbossou-Niedercorn,^10^ Xie,^11^ Zhang^12^ and our group.^13^ These protocols afforded valuable products such as aliphatic lactams, 2-hydroxymethylbenzamides, ω-hydroxylactams, benzamides, aliphatic cyclic amines, 1,4-diols and isoindolinone derivatives directly from phthalimides. Despite all these advancements, still significant limitations exist, especially related with narrow substrate scope, the use of precious catalysts and/or the employment of hydrosilanes as reducing agents. Moreover, from the point of view of obtaining the desired isoindolinone derivatives, some of these protocols present drawbacks as the concomitant hydrogenation of the aromatic ring, the lack of selectivity in the reduction of one of the carbonyl groups or the occurrence of C–N bond cleavage.

Recently, our group developed a direct protocol for the selective one-pot C2-alkoxylation and amination of cyclic imides to give 3-substituted-2,3-dihydro-1*H*-isoindolinones.^14^

In this Ru-catalysed methodology, aromatic ring hydrogenations were completely avoided and aryl ring-substituted phthalimides showed selective monoalkoxylation of one of the carbonyl groups (Fig. 2). Crucial for the catalytic activity was the presence of methanosulfonic acid (MSA) as an additive.

In the last years, (1,1,1-tris(diphenylphosphinomethyl)-ethane), so-called triphos, became a privileged ligand for the hydrogenation of carboxylic acids and related derivatives.^14,15^ Basically, in all these cases, active Ru catalysts are generated. In the last decade, the replacement of precious metals by inexpensive and widely abundant first-row base metals such as Fe,^16^ Co^16h,i^ and Mn^17^ has gained increasing importance in hydrogenation chemistry. For example, several cobalt-based systems have shown interesting activity for reductions of C–O,^18^ C–N,^18b,19^ C–C^18b,c,20^ multiple bonds and *N*-heterocycles.^21^ Notably in 2015, the groups of de Bruin and Elsevier^18*f*^ achieved for the first time the hydrogenation of carboxylic acids and esters using [Co(BF_4_)_2_·6H_2_O/triphos (**L1**)]. In addition, our group reported the CO_2_ hydrogenation to methanol using a modified related catalyst [Co(acac)_3_/triphos (**L1**)/HNTf_2_].^18*i*^

Inspired by these works, we envisaged the possibility to perform the selective reduction of imides using a cobalt-based catalyst system. Here, we show for the first time, a general and efficient methodology for the non-noble metal-catalysed reductive C2-functionalization of cyclic imides (phthalimides and succinimides).

## Results and discussion

At the start of this project the reductive methoxylation of *N*-methylphthalimide **1a** using methanol as solvent was selected as benchmark reaction (Table 1). Initially, the reaction was performed with Co(BF_4_)_2_·6H_2_O using similar conditions (150 °C, 60 bar H_2_, 18 h) known for Ru catalysts. However, no activity was observed (Table 1, entry 1). When the same reaction was conducted in the presence of 5 mol% of triphos **L1**, a quantitative yield of 3-methoxy-2-methylisoindolin-1-one **2a** was obtained with excellent selectivity (Table 1, entry 2). Gratifyingly, no traces of products coming from reduction of both carbonyl groups or aromatic ring hydrogenation were observed.

**Table 1:** [Co/triphos (**L1**)]-catalysed reductive methoxylation of *N*-methylphthalimide **1a**: optimization of the reaction conditions

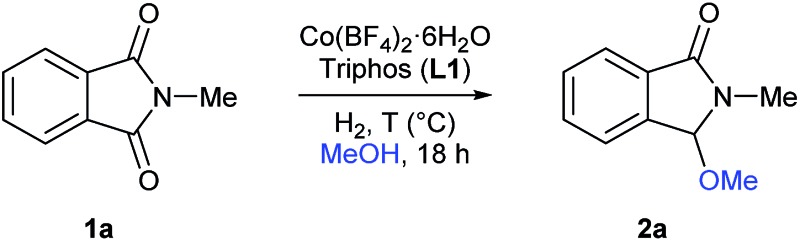						
Entry^*a*^	*T* (°C)	H_2_ (bar)	[Co]	[**L1**]	Conv.^*b*^ (%)	**2a** ^*b*^ (%)
1	150	60	2.5	—	—	—
2	150	60	2.5	5	>99	>99
3	130	60	2.5	5	>99	>99
4	130	30	2.5	5	>99	>99
5	110	60	2.5	5	>99	>99
6	110	30	2.5	5	>99	>99
7	90	50	2.5	5	>99	>99
8	90	30	2.5	5	>99	>99
**9**	**90**	**20**	**2.5**	**5**	**>99**	**>99**
10	90	10	2.5	5	86	84
11	70	60	2.5	5	91	90
12	70	40	2.5	5	87	84
13	70	20	5	10	90	89
14^*c*^	90	—	2.5	5	—	—
15^*d*^	90	20	2.5	5	53	52
16	90	20	2.5	3.75	77	75
17	90	20	2.5	2.5	60	58
18	90	20	5	5	>99	99
19	90	20	1.5	3	93	93
20	90	20	0.5	1	40	38
21	90	30	1.5	3	>99	96

^*a*^Standard reaction conditions: *N*-methylphthalimide **1a** (82.2 mg, 0.5 mmol), Co(BF_4_)_2_·6H_2_O (0.5 to 5 mol%), triphos **L1** (1 to 10 mol%), H_2_ (10–60 bar), MeOH (2 mL), 90–150 °C and 3–18 h. [Co] = [Co(BF_4_)_2_·6H_2_O] and [**L1**] in mol% with respect to **1a**.

^*b*^Conversion of **1a** and yields of **2a** were calculated by GC using hexadecane as internal standard.

^*c*^The reaction was carried out without hydrogen using a pressure tube.

^*d*^Run at 3 h.

Then, the effect of pressure and temperature was evaluated in more detail (Table 1, entries 3–13). To our delight, the reaction proceeds efficiently at much milder conditions, and excellent yields of methoxylated product **2a** were obtained at 90 °C and 20 bar of hydrogen (Table 1, entry 9). In addition, the catalytic system also showed high activities at 70 °C, albeit higher pressures of hydrogen or catalyst loadings were required in these cases (Table 1, entries 11–13). To demonstrate the need of hydrogen, we performed the reaction at 90 °C in a pressure tube and no conversion was detected (Table 1, entry 14).

At this point, the effect of the relative amounts of ligand **L1** with respect to the cobalt precursor was investigated in more detail (Table 1, entries 16–18). While for 2.5 mol% of cobalt pre-catalyst, two equivalents of ligand **L1** were required to perform the reaction efficiently (Table 1, entries 9, 16 and 17), at higher cobalt catalyst loadings (5 mol%) only one equivalent of **L1** was enough for an equally efficient methoxylation of *N*-methylphthalimide **1a** (Table 1, entry 18). Finally, the catalytic system also afforded high yields of product **2a** at 1.5 mol%, but using 30 bar of hydrogen (Table 1, entry 21).

Next, the catalytic activity of different metal pre-catalysts was evaluated (Table S1†). Among all the different cobalt precursors tested (Table S1,† entries 1–12), [Co(BF_4_)_2_·6H_2_O] and [Co(ClO_4_)_2_·6H_2_O] (Table S1,† entries 1 and 10) afforded the highest activity. In contrast, Co(acac)_3_, Co(acac)_2_ and also Ru(acac)_3_ in combination with ligand **L1** (triphos) were not active under the optimal reaction conditions (Table S1,† entry 2, 3 and 13, respectively). The non-activity of the ruthenium pre-catalyst is notworthy, since [Ru(acac)_3_/**L1**/MSA] has been recently described as an active catalytic system for the same reaction. Apparently, for the formation of our active non-noble catalyst no extra acid additive is necessary. Taking into account the important role of the tetrafluoroborate anion (^–^BF_4_) in present system, tetrafluoroborate salts of copper(ii), iron(ii) and zinc(ii) were also tested in the benchmark reaction (Table S1,† entries 14–16). However, no activity was detected for any of them, indicating that cobalt is unique for this reaction.

**Scheme 1:**
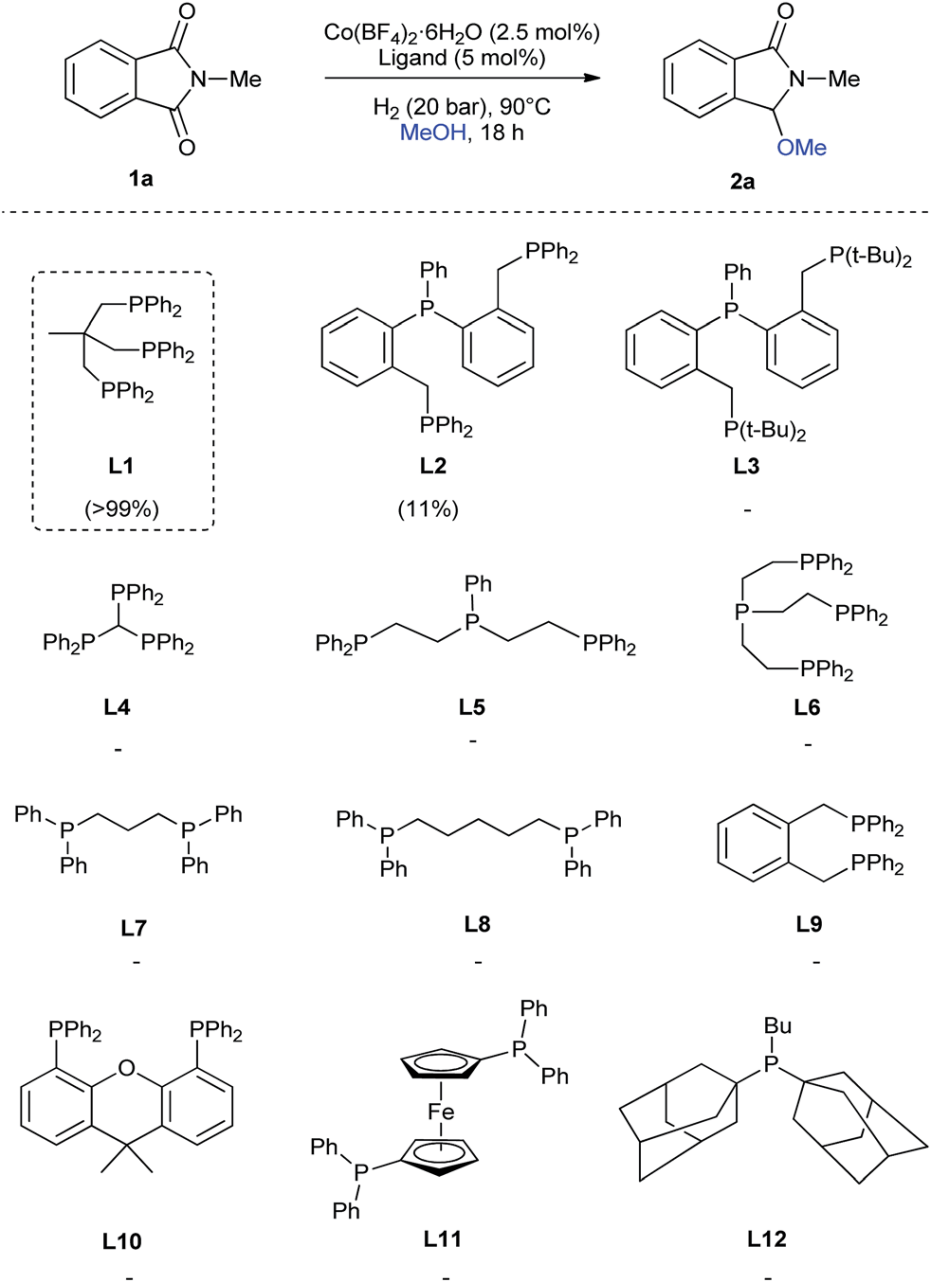
Cobalt-catalysed reductive methoxylation of *N*-methylphthalimide (**1a**): influence of the ligand. Standard reaction conditions: *N*-methylphthalimide **1a** (82.2 mg, 0.5 mmol), Co(BF_4_)_2_·6H_2_O (4.3 mg, 0.0125 mmol, 2.5 mol%), ligand (0.025 mmol, 5 mol%, 2.5 eq. to Co), H_2_ (20 bar), MeOH (2 mL), 90 °C and 18 h. Yields of product **2a** were calculated by GC using hexadecane as internal standard.

Regarding the ligand, we tested, besides **L1**, several tridentate (**L2–L5**), tetradentate (**L6**), bidentate (**L7–L11**) and monodentate (**L12**) ligands (Scheme 1) for the reductive methoxylation of *N*-methylphthalimide (**1a**). Apart from triphos (**L1**), which exhibited the best yield (>99%), only the tridentate ligand **L2** afforded **2a**, albeit in low yields (11%).

Having established the [Co(BF_4_)_2_·6H_2_O/triphos (**L1**)] system as the best catalyst, we decided to explore its activity for the reductive methoxylation of more than 20 symmetrical substituted cyclic imides (Table 2). In general, all the reactions were conducted using 2.5 mol% of cobalt pre-catalyst, 5 mol% of ligand **L1** under 90 °C and 20 bar of hydrogen.

**Table 2:** [Co/triphos (**L1**)]-catalysed reductive methoxylation of different substituted cyclic imides

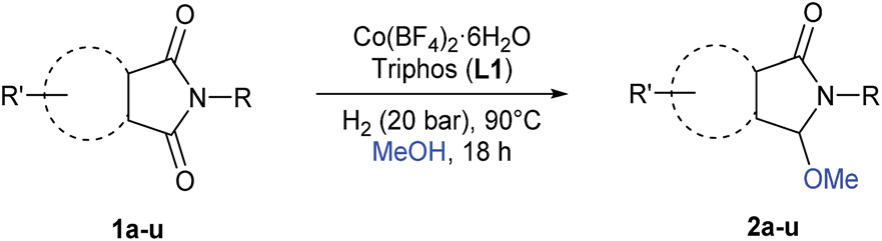			
Entry^*a*^	Cyclic imide **1**	[Co] (mol%)	**2** ^*b*^ [%]
1	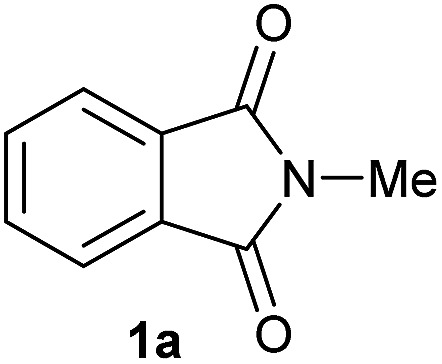	2.5	**2a** [95]
2	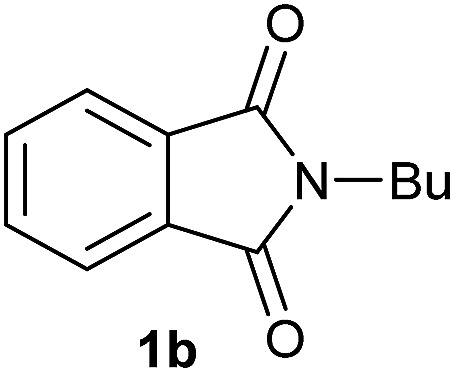	4	**2b** [89]
3	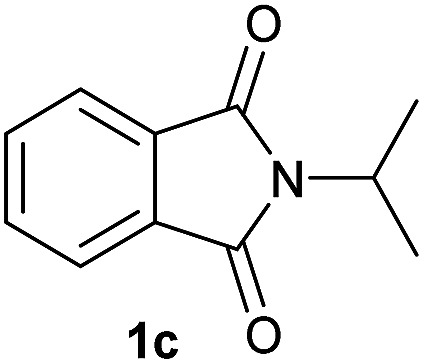	4	**2c** [90]
4	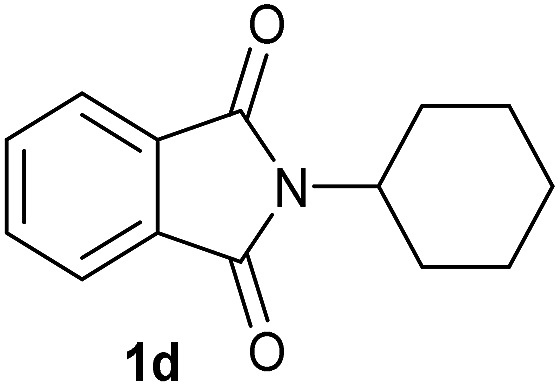	4	**2d** [96]
5	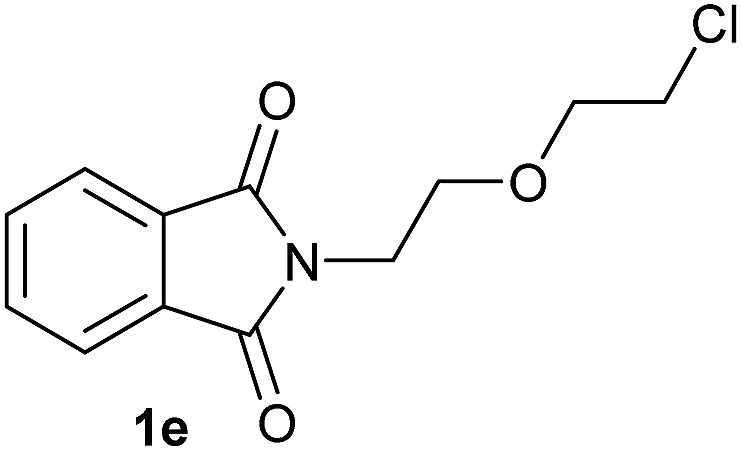	2.5	**2e** [97]
6	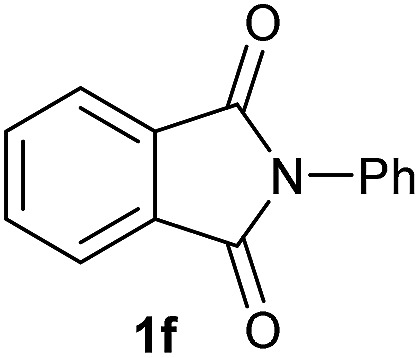	2.5	**2f** [93]
7	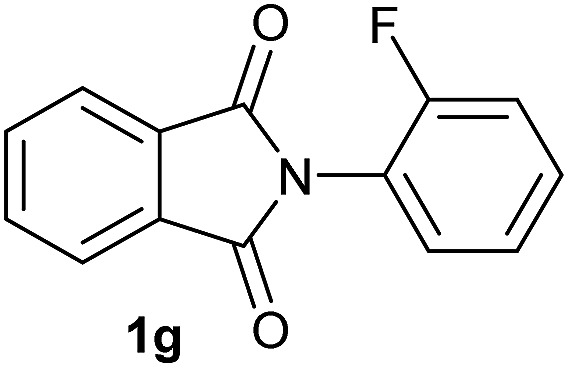	2.5	**2g** [88]
8	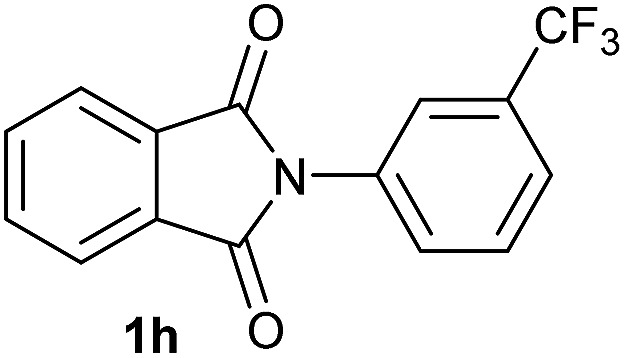	6	**2h** [91]
9	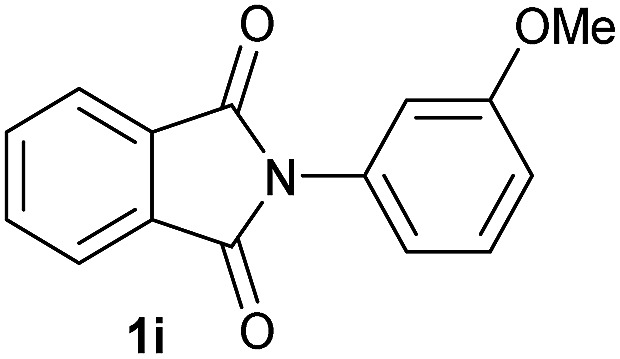	4	**2i** [98]
10^*c*^	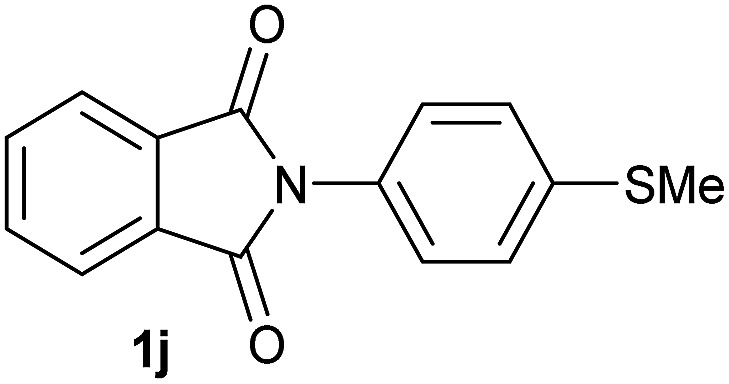	2.5	**2j** [95]
11	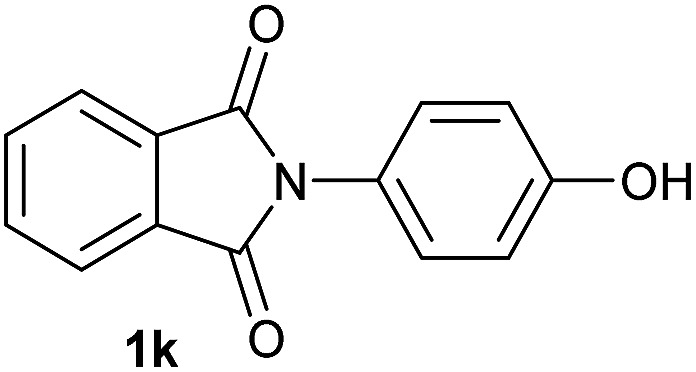	2.5	**2k** [96]
12	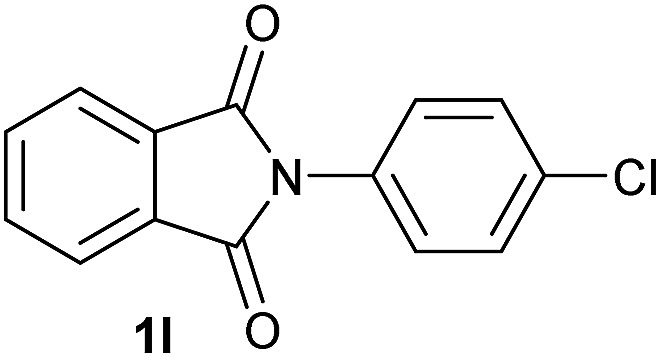	2.5	**2l** [94]
13	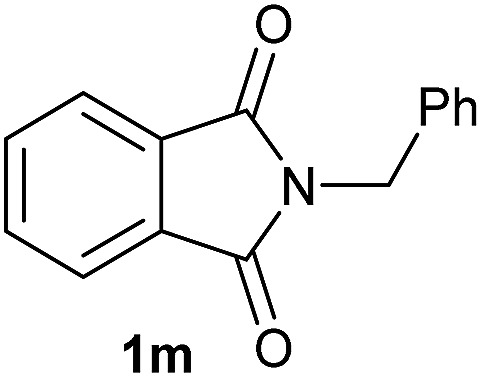	4	**2m** [99]
14	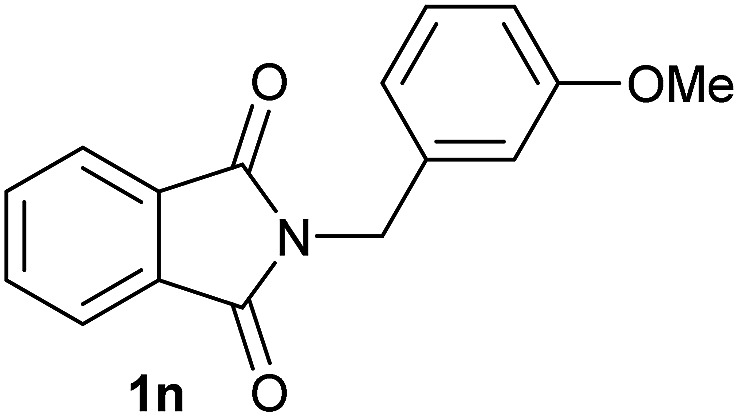	5	**2n** [99]
15^*c*^	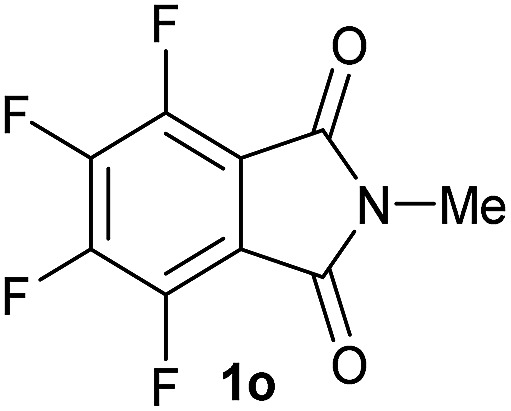	2.5	**2o** [86]
16	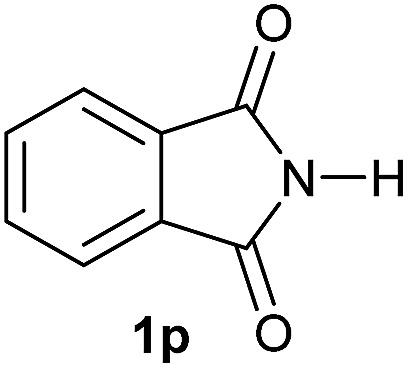	2.5	**2p** [89]
17	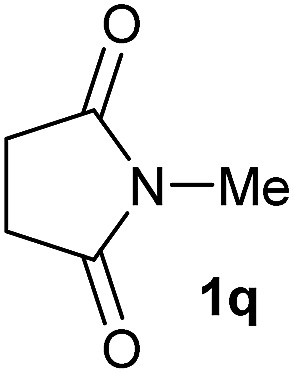	2.5	**2q** [81]
18	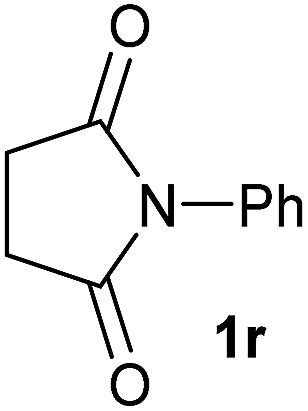	2.5	**2r** [89]
19	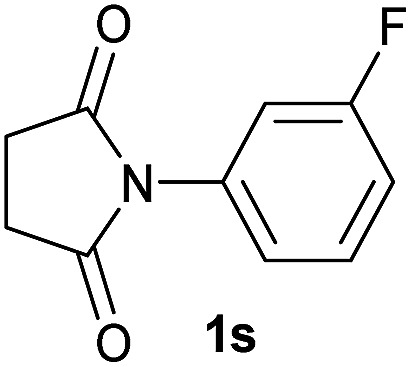	2.5	**2s** [94]
20	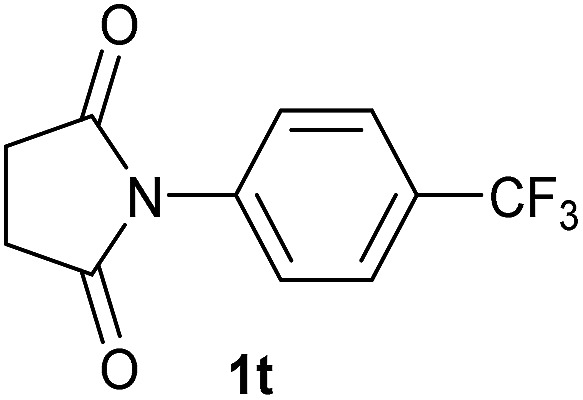	2.5	**2t** [85]
21	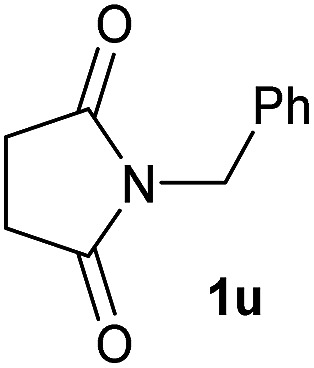	4	**2u** [80]

^*a*^Standard reaction conditions: cyclic imide (0.5 mmol), Co(BF_4_)_2_·6H_2_O (4.25 mg, 0.0125 mmol, 2.5 mol%), triphos **L1** (15.6 mg, 0.025 mmol, 5 mol%, 2 eq. to Co), H_2_ (20 bar), MeOH (2 mL), 90 °C and 18 h. When the reaction was carried out using 4 to 6 mol% of cobalt precatalyst, 1.5 eq. of **L1** respect to the metal was added.

^*b*^Isolated yield of the product after purification by column chromatography on silica are given between brackets.

^*c*^Run at 110 °C.

Gratifyingly, excellent selectivity for the monoalkoxylation product was observed for all the studied substrates. *N*-Alkyl substituted phthalimides (**1a–1e**) afforded 3-methoxylated isoindolinones **2a–2e** in very good isolated yields (89–97%, Table 2, entries 1–5). To study the influence on the catalytic activity of the electronic character of the *N*-substituent, different *N*-aryl or *N*-benzyl substituted phthalimides (**1f–n**) were tested with the cobalt system. Phthalimides containing fluoro-, trifluoromethyl-, methoxy-, methylthio-, hydroxy- and chloride-substituted aryl rings in *ortho*-, *meta*- and *para*-position were successfully converted affording the corresponding methoxylated products **2f–n** in high isolated yields (86–99%, Table 2, entries 6–14). Notably, this unusual reductive transformation worked well for the fluorinated phthalimide derivative **1o** obtaining the methoxy compound **2o** in high isolated yield (86%, Table 2, entry 15). For the first time, the [Co/triphos] allowed for this reductive transformation of NH-phthalimides. In fact, the system showed an excellent activity for the methoxylation of **1p** affording the methoxy product **2p** in excellent yield (89%, Table 2, entry 16). Finally, different *N*-substituted succinimides (**1q–u**) were tested, too. *N*-Methyl, *N*-phenyl, and *N*-benzyl succinimides, were smoothly methoxylated giving the desired 3-alkoxy-pyrrolidin-2-one derivatives **2q–u** in good to very good isolated yields (80–94%, Table 2, entries 17–21). Unfortunately, when *N*-benzyl-2,3-pyridinedicarboximide and *N*-anisoyl-2-pyrrolidinone, as examples of heterocyclic and linear imides respectively, were subjected to the optimized reaction conditions no desired product could be obtained.

**Scheme 2:**
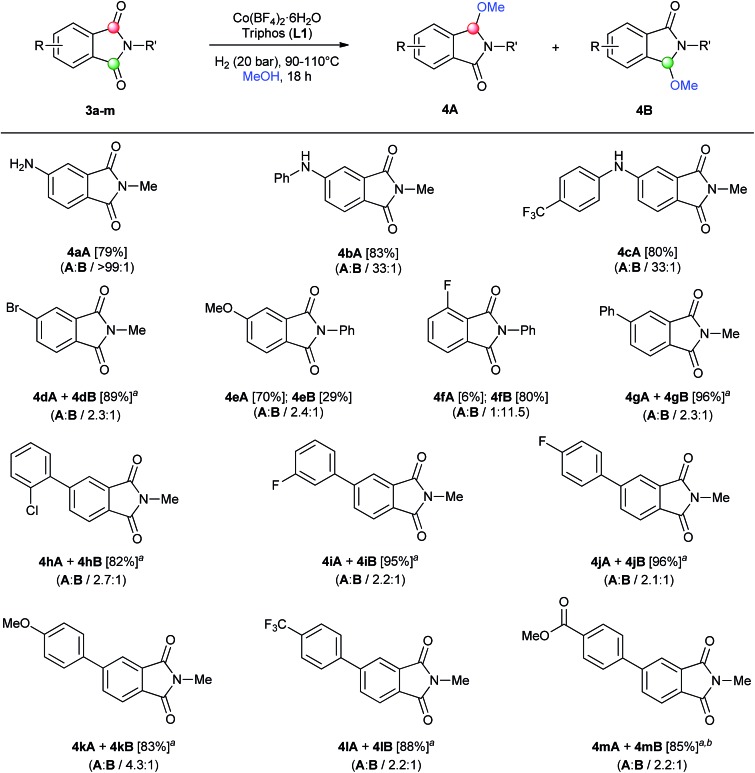
Regioselective [Co/triphos (**L1**)]-catalysed reductive methoxylation of several asymmetrical ring-substituted phthalimides. Standard reaction conditions: phthalimide (0.5 mmol), Co(BF_4_)_2_·6H_2_O (4–6 mol%), triphos **L1** (6–9 mol%, 1.5 eq. to Co), H_2_ (20 bar), MeOH (2 mL), 90–110 °C and 18 h. Specific reaction conditions for cyclic imides **3a**, **3e** and **3g**: Co(BF_4_)_2_·6H_2_O (4 mol%) at 110 °C, for cyclic imides **3b–d** and **3f**: Co(BF_4_)_2_·6H_2_O (4 mol%) at 90 °C and for cyclic imides **3h–m**: Co(BF_4_)_2_·6H_2_O (6 mol%) at 110 °C. Isolated yields of the products are given between brackets. In parentheses is shown the relative selectivity for each regioisomer **A** and **B**, calculated by GC-MS and ^1^H-NMR analysis (see ESI†). ^a^The isolated yield corresponds to the unseparable mixture of regioisomers **A** and **B**. ^b^Small amounts (<5%) of products **A** and **B** containing ester group hydrogenated to alcohol were detected.

Once we had shown the generality of our protocol, we became interested to study the selective reduction of non-symmetrical phthalimides (Scheme 2). These are more challenging substrates as the reactivity of the two carbonyl functions might be similar. Up to date, there is only one ruthenium catalyst described,^14^ which showed moderate to good regioselectivities in such transformations (see Fig. 2). Therefore, the development of new non-precious metal-based strategies to selectively functionalize one of the carbonyl groups still remains a challenging task.

As shown in Scheme 2 the regioselective monomethoxylation of unsymmetrical aryl ring-substituted phthalimides using the cobalt/triphos system proceeded selectively. Most of the phthalimides used for this study are not commercially available and had to be synthetized (see ESI for Experimental details†). To our delight, C4-substituted phthalimides with nitrogen-based electron-donating groups (**3a–c**), exhibited excellent regioselectivities (>33 : 1) for the monofunctionalization on the C2 carbonyl group (isomer **A**), affording the corresponding isoindolinones in good yields. On the other hand, C4-substituted phthalimides with an electron-withdrawing group such as Br (**3d**), or an oxygen-based electron-donating group like methoxy (**3e**), gave lower regioselectivities (2.3 : 1 and 2.4 : 1) to the same carbonyl group than nitrogen-based substituents. Furthermore, excellent isolated yields were achieved for the mixture of regioisomer products (**4dA** + **4dB**) (89%) as well as for the regioisomers **4eA** and **4eB** (70 and 29% yield, respectively). The difference between the observed regioselectivities for an amino and a methoxy C4-substituted phthalimide can be explained by the more important coordinating character of the nitrogen, that can direct the cobalt complex to functionalize the C2 carbonyl.^11*a*^ Interestingly, a C3-fluorine substituted *N*-phenyl phthalimide (**3f**) afforded a good regioselectivity for the functionalization in the carbonyl group but at position C7, hence giving isomer **B**. This switch in the regioselectivity could be exploited as a synthetic tool. Both regioisomers (**4fA** and **4fB**) were isolated separately in 6 and 80% yield, respectively. Next, a small family of C4-aryl substituted *N*-methyl phthalimides **3g–m** was synthetized (see Scheme S2†) and their reductive alkoxylation was studied. Different substituents such as *o*-Cl (**4h**), *m*- and *p*-F (**4i–j**), *p*-OMe (**4k**), *p*-CF_3_ (**4l**) and *p*-C(O)OMe (**4m**) aryl groups afforded moderate to good regioselectivities (>2.2 : 1) to the carbonyl **A** position, with no influence of their electronic character. For all of these examples, alkoxylated products **4g–m** were successfully isolated in up to 96% yield as a mixture of regioisomers (**A** and **B**). Functional groups like halogen, ether, trifluoromethyl and ester groups were tolerated in the presence of this cobalt-based system.

**Scheme 3:**
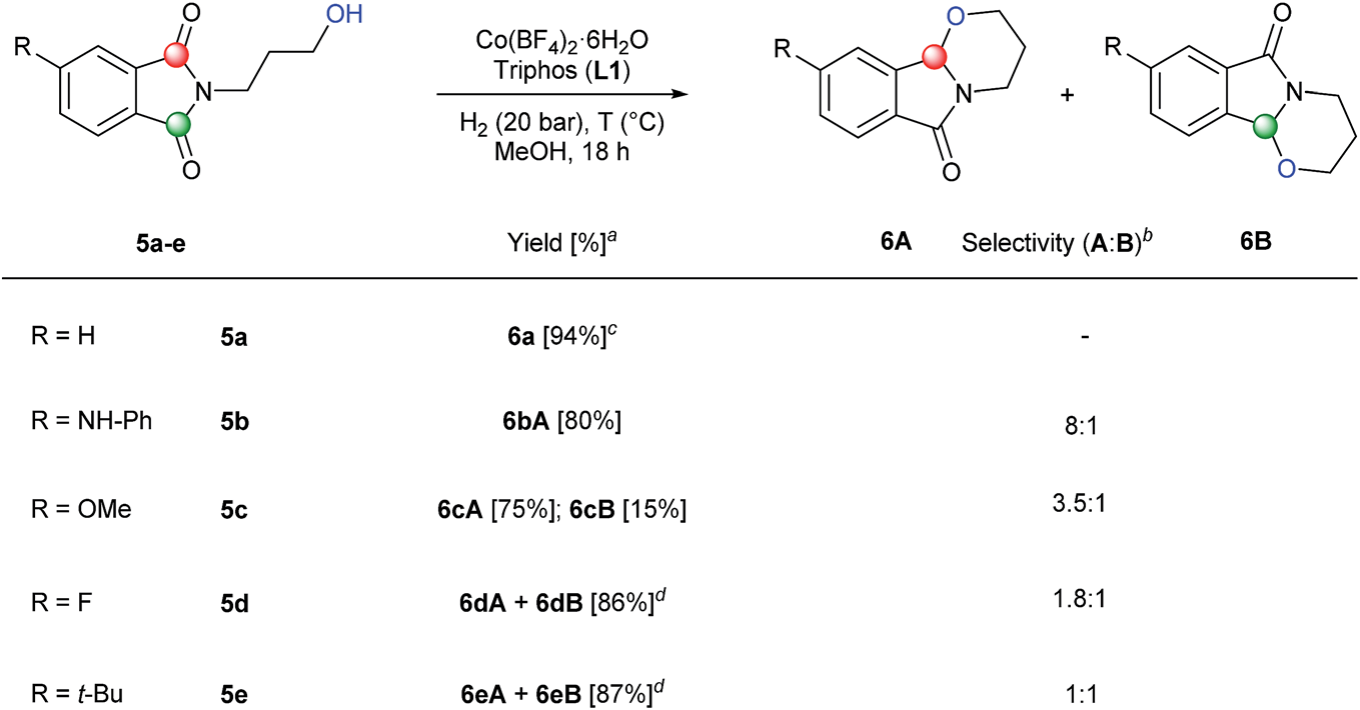
Synthesis of tricyclic compounds by one-step regioselective [Co/triphos (**L1**)]-catalysed intramolecular reductive cyclization of different C4-substituted *N*-hydroxypropyl phthalimides. Standard reaction conditions: phthalimide (0.5 mmol), Co(BF_4_)_2_·6H_2_O (2.5–6 mol%), triphos **L1** (5–9 mol%, 1.5–2 eq. to Co), H_2_ (20 bar), MeOH (2 mL), 90–110 °C and 18 h. When the reaction was carried out using 6 mol% of cobalt precatalyst, 1.5 eq. of ligand **L1** respect to the metal was added. Specific reaction conditions for cyclic imide **5a**: Co(BF_4_)_2_·6H_2_O (2.5 mol%) at 90 °C, for cyclic imides **5b** and **5d**: Co(BF_4_)_2_·6H_2_O (6 mol%) at 90 °C and for cyclic imides **5c** and **5e**: Co(BF_4_)_2_·6H_2_O (6 mol%) at 110 °C. ^a^Isolated yield of the products are given. ^b^The relative selectivity for each cyclic regioisomer **A** and **B** was calculated by GC-MS and ^1^H-NMR analysis (see ESI†). ^c^When (R = H) compounds **A** and **B** are the same. ^d^Isolated yield of the unseparable mixture of products **A** and **B** is given.

Furthermore, we envisaged the possibility to perform selective intramolecular reductive alkoxylations. This route gives straightforward access to interesting building blocks for the synthesis of alkaloids and intermediates for the production of a stereogenic carbon on the α-positon to the nitrogen lactam.^3c,d,g^ Using several *N*-(3-hydroxypropyl)phthalimides (**5a–e**), it was possible to efficiently synthesize these tricyclic compounds^22^ in one-step (Scheme 3). In order to achieve full conversions, the reactions were conducted under 20 bar of hydrogen in methanol at 90 or 110 °C in the presence of 2.5–6 mol% catalyst. *N*-(3-Hydroxypropyl)phthalimide **5a** with no substitution in the aromatic ring afforded cyclic compound **6a** in an excellent isolated yield (94%). Encouraged by this result, different C4-substituted *N*-(3-hydroxypropyl)phthalimides were studied in order to explore the regioselectivity of the process. Substrates with an electron-donating group in C4 position such as NH-Ph (**5b**) or OMe (**5c**), showed good to excellent regioselectivities (8 : 1 and 3.5 : 1, respectively) to the attack of carbonyl group **A**. The better regioselectivity for the amino substituted phthalimide **5b** in comparison with the methoxy one **5c** can be also explained by the directing effect of the nitrogen.^11*a*^ Regioselectivity was moderate towards isomer **A** in the case of a phthalimide substituted with an electron-withdrawing group such as F (**5d**), giving products **6dA** and **6dB** in 86% yield. Finally, no regioselectivity was detected in the intramolecular alkoxylation of the phthalimide **5e**, containing an alkyl substituent in the aromatic ring. Two regioisomeric positions **A** and **B** were reacted with the same selectivity, affording the mixture of regioisomers **6eA** and **6eB** in 87% isolated yield.

**Scheme 4:**
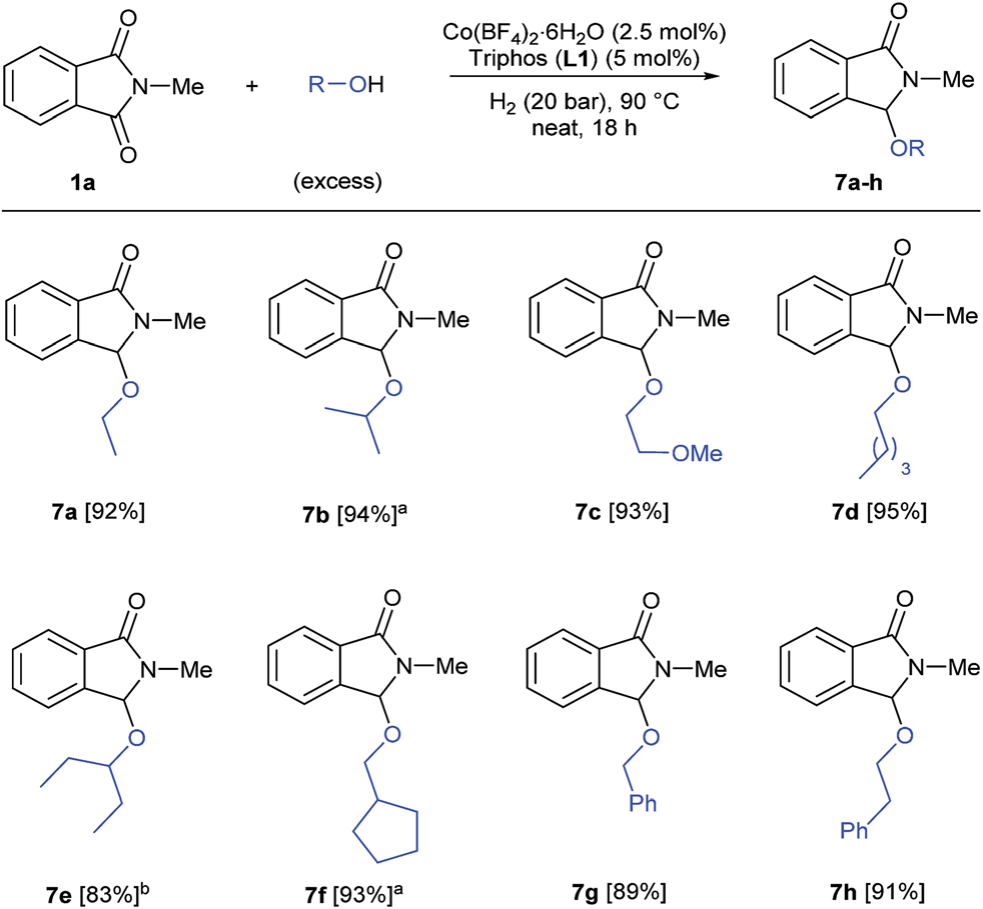
[Co/triphos]-catalysed reductive alkoxylation of *N*-methylphthalimide **1a** with different alcohols. Standard reaction conditions: *N*-methylphthalimide **1a** (82.2 mg, 0.5 mmol), Co(BF_4_)_2_·6H_2_O (4.25 mg, 0.0125 mmol, 2.5 mol%), triphos **L1** (16.7 mg, 0.025 mmol, 5 mol%, 2 eq. to Co), H_2_ (20 bar), alcohol (2 mL), 90 °C and 18 h. Isolated yields of the products are given. [a] Run with Co(BF_4_)_2_·6H_2_O (4 mol%) and triphos **L1** (6 mol%). [b] Run with Co(BF_4_)_2_·6H_2_O (6 mol%) and triphos **L1** (9 mol%).

Finally, we decided to investigate the general applicability of different alcohols in this reductive functionalization, reacting phthalimide **1a** with a wide range of alcohols under neat conditions (Scheme 4). All the reactions were conducted under the previously optimized conditions for methanol (2.5 mol% Co, 5 mol% **L1**, 20 bar of hydrogen, 90 °C, 18 h) and in specific cases, higher catalyst loadings were required to obtain full conversions of **1a**. Both aliphatic primary alcohols (ethanol, 2-methoxyethanol, pentanol, cyclopentanemethanol) and secondary ones (isopropanol, 3-pentanol) afforded the corresponding 3-alkoxylated isoindolinones **7a–f** with excellent isolated yields (83–95%). In addition, benzyl and phenethyl alcohols also reacted successfully to give the corresponding C3 functionalized isoindolinones in very good yields (89 and 91%, respectively). In conclusion, this cobalt-catalysed transformation allows the straightforward synthesis of a variety of functionalized isoindolinone derivatives.

**Fig. 3:**
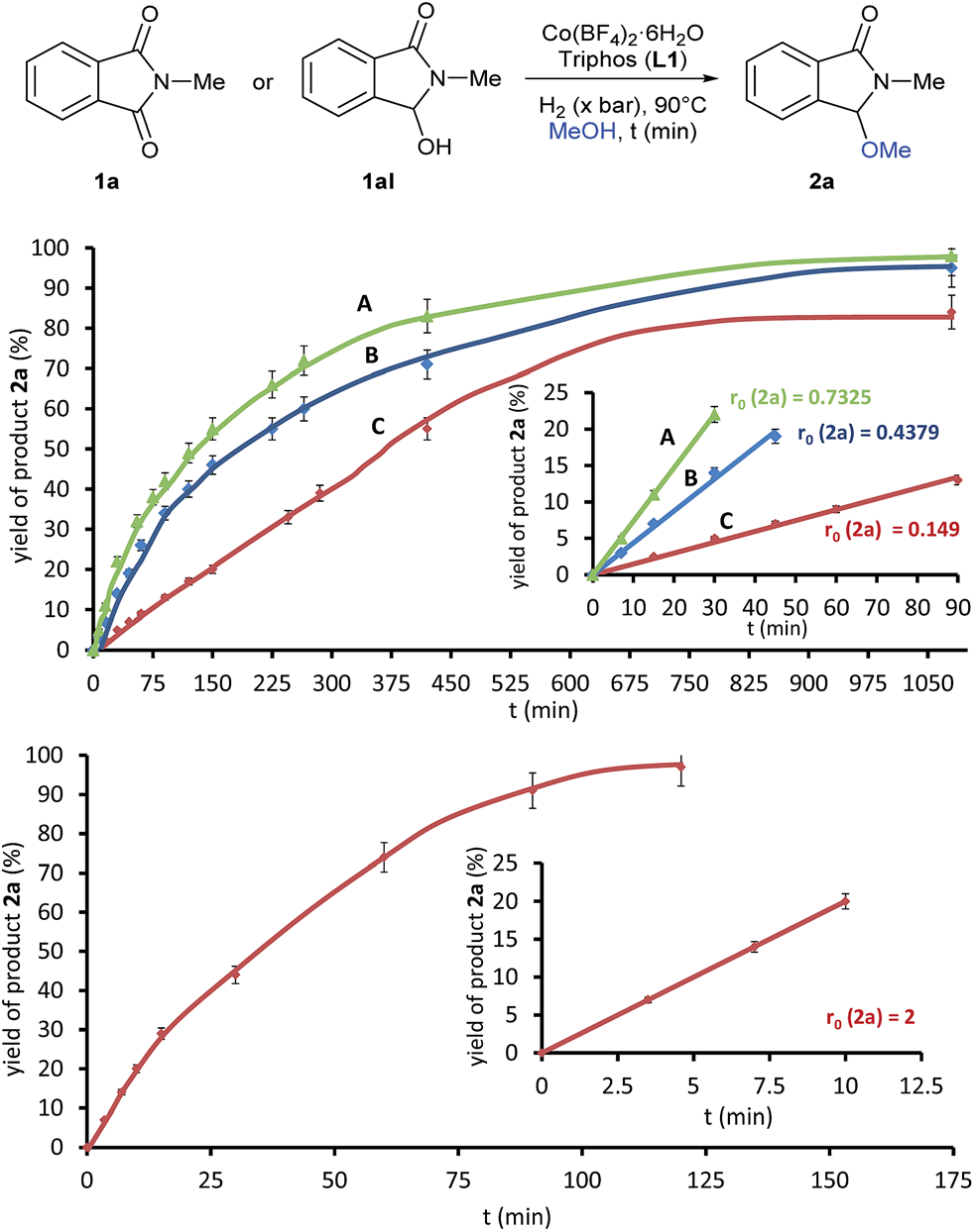
(Up) Yield/time kinetic profile for the formation of product **2a** in the reductive methoxylation of *N*-methylphthalimide **1a** using methanol at 90 °C under different pressures of molecular hydrogen: (A) 30 bar, (B) 20 bar and (C) 10 bar. (Bottom) Yield/time kinetic profile for the formation of product **2a** from the intermediate hemiaminal **1aI** using methanol and molecular hydrogen (20 bar) at 90 °C. Insets correspond to the initial rate plots where (*r*_0_) is the slope of the linear equation: [yield (%) = *r*_0_ × time (min)] defined at initial reaction times and expressed as [yield (%) of product × *t* (min)^–1^]. Standard reaction conditions: substrate **1a** or **1aI** (3.0 mmol), Co(BF_4_)_2_·6H_2_O (25.5 mg, 0.075 mmol, 2.5 mol%), triphos **L1** (100.5 mg, 0.15 mmol, 5 mol%, 2 eq. to Co), MeOH (12.0 mL) and H_2_ (10, 20 or 30 bar) at 90 °C. Yields of product **2a** were calculated by GC using hexadecane as internal standard. Vertical error bar (5%) for all data points is shown.

To gain insight in the mechanism of the cobalt-catalysed reaction, some kinetic studies (see Fig. 3 and S1–S4†) were performed. Fig. 3 (up) shows the yield/time kinetic profiles for the formation of 3-methoxy-2-methylisoindolin-1-one **2a** in the reductive methoxylation of **1a** at different hydrogen pressures: (**A**) 30 bar, (**B**) 20 bar and (**C**) 10 bar. No induction period was detected in any experiment, indicating that the catalytically active species can be formed easily. The comparison of the different kinetic profiles reveals that the initial rates (*r*_0_), expressed as [yield (%) of **2a** × *t* (min)^–1^], decrease notably from 30 to 10 bar of hydrogen (0.7325, 0.4379 and 0.149, respectively). Therefore, the reaction exhibits a strong dependence on the hydrogen pressure indicating that the initial hydrogenation of the phthalimide **1a** to the intermediate hemiaminal **1aI** is the rate limiting step of the overall process. Thus, the subsequent methoxylation of **1aI** is expected to be the fast step. In order to confirm these assumptions, additional kinetic experiments using the hemiaminal **1aI** as substrate were performed. Fig. 3 (bottom) shows the yield/time kinetic profile of the reductive methoxylation of **1aI** under 20 bar of hydrogen (see also Fig. S4†). The initial rate for the formation of **2a** in this case (*r*_0_ = 2) is almost five times larger than the one obtained using phthalimide **1a** as starting material (*r*_0_ = 0.4379).

This observation supports the methoxylation of **1aI** to **2a** as the fast step, and the hemiaminal **1a** as a real intermediate of this transformation.^23^ Indeed, additional control experiments starting from **1aI** corroborate this observation (see Scheme S5†). The reaction of the hemiaminal **1aI** in the presence of lower catalyst loadings (0.5 mol% Co) afforded good yields of the methoxylated product **2a**. Moreover, **2a** can be produced in quantitative yields (98%) from **1aI** with ligand-free [Co(BF_4_)_2_·6H_2_O] as catalyst.^4*m*^ Apparently, this simple cobalt salt is able to catalyze the alkoxylation process. Interestingly, when the same reaction is performed adding ligand **L1** and in the absence of hydrogen, *N*-methylphthalimide (**1a**) was detected in 19% yield as a by-product coming from the de-hydrogenation reaction of **1aI** mediated by [Co/**L1**].

In addition, poisoning studies with TEMPO (2,2,6,6-tetramethylpiperidine-1-oxyl), a radical inhibitor, and TMTU (tetramethylurea), a binding poison, were performed (Table S2†).^18*f*^ In the case of TEMPO the reductive methoxylation proceeded successfully, indicating that no radicals are involved in the mechanism. In contrast, TMTU caused a complete inhibition of the reaction with only half equivalent with respect to the catalyst. This effect, previously observed for the carboxylic acids hydrogenation with the same catalyst,^18*f*^ can be explained by either a dinuclear cobalt species or TMTU acting as a bridging poison.

With the aim of understanding in more detail the catalytic system, high-resolution electrospray ionization mass spectrometry (HR ESI-MS) experiments were performed (Fig. S5–S9†). At short reaction times of 0.5 and 2 hours a signal at *m*/*z* 728.156 was detected, consistent with [Co(**L1**)(COO^–^)]^+^. The formation of this species is justified by the use of MeOH/0.1% HCOOH mixture as solvent for the ESI Experiment. In fact, in samples containing a simple mixture of Co(BF_4_)_2_·6H_2_O/**L1** and Co(BF_4_)_2_·6H_2_O/**L1**/**2a**, the same peak was detected. Any of these tests showed a cobalt species coordinated to *N*-methylphthalimide **1a**. Interestingly, a species with *m*/*z* 1309.404 corresponding to [Co(**L1**)_2_(H)_2_]^+^ could be detected as an important peak in samples at short reaction times. However, in a sample taken at the end of the reaction, this species becomes less important and, using acetonitrile as ESI-MS solvent, a new peak at *m*/*z* 901.245 appears. The latter signal is consistent with [Co(**L1**)(CH_3_CN)(**2a**)]^+^, a possible resting state containing the methoxylated product **2a**.

**Fig. 4:**
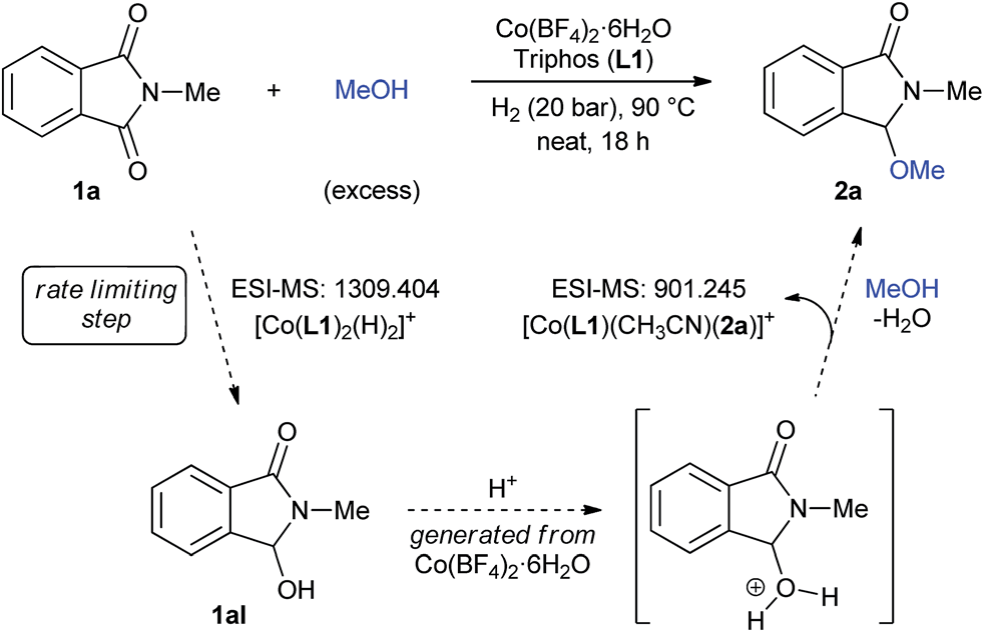
Possible reaction mechanism for the [Co/**L1**]-catalysed reductive methoxylation of cyclic imides.

Based on all these observations, a plausible mechanism for the [Co/triphos (**L1**)]-catalysed reductive alkoxylation of cyclic imides is depicted in Fig. 4. The [Co(**L1**)_2_(H)_2_]^+^ species, detected by ESI-MS, is proposed as the active catalyst for the hydrogenation of **1a** to the hemiaminal intermediate **1aI**. At the end of the reaction, [Co(**L1**)(CH_3_CN)(**2a**)]^+^ is detected as the resting state.

## Conclusions

In conclusion, a general and efficient cobalt-catalysed reductive alkoxylation of cyclic imides was presented for the first time. This green protocol avoids the use of stoichiometric amounts of silanes or metal hydrides. Hydrogenation of the aromatic ring of the phthalimide core does not take place and excellent chemoselectivity to the mono-alkoxylation products is obtained. A wide range of phthalimides/succinimides are selectively functionalized under mild conditions. Notably, the [Co/triphos] system is active without the need of any acid additive to give 3-alkoxy-2,3-dihydro-1*H*-isoindolin-1-one and 3-alkoxy-pyrrolidin-2-one derivatives in high isolated yields. Furthermore, this cobalt based catalyst allows the selective functionalization of one of the carbonyl groups in non-symmetrical aryl ring-substituted phthalimides. Additionally, the reaction can be performed in an intramolecular fashion, giving *N*,*O*-acetal tricyclic compounds in one-step with high yields. Kinetic investigations revealed that the initial hydrogenation of the phthalimide to the hemiaminal intermediate is the rate limiting step of the overall process. This novel base metal protocol opens a door to the development of environmentally-benign processes for the selective synthesis of functionalized *N*-heterocyclic compounds.

## Experimental details

### General procedure for the reductive methoxylation of *N*-methylphthalimide (**1a**)

A 4 mL glass vial containing a stirring bar was sequentially charged with *N*-methylphthalimide **1a** (82.2 mg, 0.5 mmol), Co(BF_4_)_2_·6H_2_O (4.25 mg, 0.0125 mmol, 2.5 mol%), triphos **L1** (16.75 mg, 0.025 mmol, 5 mol%, 2.5 eq. to Co), *n*-hexadecane (50.0 mg) as an internal standard and MeOH (2.0 mL) as solvent. Afterwards, the reaction vial was capped with a septum equipped with a syringe and set in the alloy plate, which was then placed into a 300 mL autoclave. Once sealed, the autoclave was purged three times with 30 bar of hydrogen, then pressurized to 20 bar and placed into an aluminium block, which was preheated at 90 °C. After 18 h, the autoclave was cooled in an ice bath, and the remaining gas was carefully released. Finally, the reaction mixture was diluted with ethyl acetate and analysed by GC.

### General procedure for the reductive alkoxylation of cyclic imides

A 4 mL glass vial containing a stirring bar was sequentially charged with cyclic imide (0.5 mmol), Co(BF_4_)_2_·6H_2_O (2.5–6 mol%), triphos **L1** (5–9 mol%, 1.5–2 eq. to Co) and alcohol (2.0 mL) as solvent. Afterwards, the reaction vial was capped with a septum equipped with a syringe and set in the alloy plate, which was then placed into a 300 mL autoclave. Once sealed, the autoclave was purged three times with 30 bar of hydrogen, then pressurized to 20 bar and placed into an aluminium block, which was preheated at 90–130 °C. After 18 h, the autoclave was cooled in an ice bath, and the remaining gas was carefully released. Finally, the reaction mixture was diluted with ethyl acetate and purified by silica gel column chromatography (*n*-heptane/ethyl acetate mixtures) obtaining the desired alkoxylated derivatives.

### General procedure for the kinetic studies

A 100 mL glass inlet containing a stirring bar was sequentially charged with the corresponding substrate **1a** or **1aI** (3.0 mmol), Co(BF_4_)_2_·6H_2_O (25.5 mg, 0.075 mmol, 2.5 mol%), triphos **L1** (100.5 mg, 0.15 mmol, 5 mol%, 2.5 eq. to Co), *n*-hexadecane (250.0 mg) as an internal standard and MeOH (12.0 mL) as solvent. Afterwards, the reaction inlet was then placed into a 100 mL autoclave. Once sealed, the autoclave was purged three times with 30 bar of hydrogen, then pressurized to 10, 20 or 30 bar and placed into an aluminium block, which was preheated at 90 °C. Periodically, aliquots of 200 μL were taken at different times of reaction, diluted with ethyl acetate and analysed by GC.
