# Supplementary material for: Unprecedented selective homogeneous cobalt-catalysed reductive alkoxylation of cyclic imides under mild conditions
Source: Chem Sci. 2017 Jun 12;8(8):5536–46. doi: 10.1039/c7sc01175j (PMC5618770; doi:10.1039/c7sc01175j)

Electronic Supplementary Material (ESI) for Chemical Science.
This journal is © The Royal Society of Chemistry 2017

ELECTRONIC SUPPLEMENTARY INFORMATION

Unprecedented Selective Homogeneous Cobalt-catalysed Reductive Alkoxylation of Cyclic Imides under Mild Conditions

Jose R. Cabrero-Antonino,[a] Rosa Adam,[a] Veronica Papa,[a] Mattes Holsten,[a] Kathrin Junge,[a] and Matthias Beller[a*]

[a]Leibniz Institute für k[illegible]lyse e. V. an der Universität Rostock,
Albert-Einstein-Straße 29a, 18059 Rostock (Germany);
Phone: +34963877800; Fax: +349638 77809
[*]Corresponding author: matthias.beller@catalysis.de

1. GENERAL INFORMATION

2. ADDITIONAL EXPERIMENTAL PROCEDURES

3. ADDITIONAL TABLES

Table S1. Reductive methoxylation of N-methylphthalimide (**1a**): Screening of the metal catalyst.

Table S2. Reductive methoxylation of N-methylphthalimide (**1a**): Influence of the organic additives.

4. ADDITIONAL SCHEMES

5. ADDITIONAL FIGURES

6. CHARACTERIZATION DATA OF THE ISOLATED PRODUCTS

7. REFERENCES

8. NMR SPECTRA OF THE ISOLATED PRODUCTS

1. GENERAL INFORMATION

All reagents were obtained commercially from various chemical companies and were used without further purification. Phosphine ligands **L2** and **L3** were synthetized according previously described methodology.[1] All hydrogenation experiments were carried out in 300 mL autoclave or 100 mL autoclave in the case of kinetic studies (PARR Instrument Company). In order to avoid unspecific reductions, all catalytic reactions were carried out in 4 mL glass vials, which were set in an alloy plate and placed inside the autoclave. In the case of kinetic studies a 100 mL glass inlet was used. GC conversion and yields were determined by GC-FID, HP 6890 with FID detector, column HP530 m x 250 mm x 0.25 µm. Mass spectra were recorded on a GC-MS Agilent 5973 Network equipped with a mass selective detector. All the products were isolated by silica gel column chromatography using as eluent (*n*-heptane / AcOEt) mixtures. ^{1}H NMR, ^{13}C NMR, ^{19}F NMR, 2D-NOESY NMR, 2D-HOESY (^{1}H-^{19}F) NMR spectra were recorded on a Bruker AV 300 or Bruker AV 400 spectrometer. All chemical shifts (δ) are reported in parts per million (ppm) and coupling constants (*J*) in herzs (Hz). All chemical shifts are reported relative to $CDCl_3$ (deuterated chloroform) peaks (δ 7.26 for ^{1}H NMR and δ 77.16 for ^{13}C NMR) or DMSO-d_6 (deuterated dimethyl sulfoxide) peaks (δ 3.33 for ^{1}H NMR and δ 39.52 for ^{13}C NMR). All measurements were carried out at room temperature unless otherwise stated. HRMS measurements of the all isolated products were performed using the electrospray ionization technique in UPLC (ultra-pressure) equipment.

2. ADDITIONAL EXPERIMENTAL PROCEDURES

General procedure for the synthesis of phthalimides by condensation[2]: A 25 mL Schlenk containing a stirring bar was sequentially charged with the corresponding anhydride (7.2 mmol, 1.2 eq), amine (6.0 mmol) and dry DMF (8 mL) as solvent. Afterwards, the Schlenk was sealed and set in an oil bath at 110 ºC during 2-16 h. When the reaction was complete, the crude reaction mixture was allowed to reach room temperature, passed to a funnel extraction, and 10 mL of distillated water were added. Then, the aqueous phase was extracted three times with 15 mL of ethyl acetate. The combined organic layers were dried with anhydrous Na_2SO_4 and filtered under gravity. The solvent was removed under reduced pressure. Finally, the reaction mixture was purified by silica gel column chromatography (*n*-heptane / ethyl acetate mixtures) obtaining the corresponding N-substituted cyclic imide derivative in (61-98%) yields.

General procedure for the synthesis of ring-substituted phthalimides through Suzuki-Miyaura cross coupling reactions[3]: A 25 mL Schlenk containing a stirring bar was sequentially charged under argon with 4-Br-*N*-methylphthalimide (**3d**) (480.12 mg, 2.0 mmol), phenylboronic acid (1.5 eq, 3.0 mmol), $Pd(OAc)_2$ (19.0 mg, 0.08 mmol, 4 mol%), PPh_3 (105.2 mg, 0.4 mmol, 20 mol%), Cs_2CO_3 (1.3 g, 4.0 mmol, 2 eq) and dry toluene (10.0 mL) as solvent. Afterwards, the Schlenk was sealed and set in an oil bath at 110 ºC during 15 h. When the reaction was complete, the crude reaction mixture was allowed to reach room temperature, passed to a funnel extraction, and 20 mL of distillated water were added. The aqueous phase was extracted three times with 20 mL of dichloromethane. The combined organic layers were dried with anhydrous Na_2SO_4 and filtered under gravity. The solvent was removed under reduced pressure. Finally, the reaction mixture was purified by silica gel column chromatography (*n*-heptane / ethyl acetate mixtures) obtaining the corresponding ring-substituted *N*-methylphthalimide derivative **3g-m** in (46-85%) yields.

General procedure for the synthesis of ring-substituted phthalimides through Buchwald-Hartwig amination[4]: A 25 mL Schlenk containing a stirring bar was sequentially charged under argon with 4-Br-*N*-methylphthalimide (**1x**) (480.12 mg, 2.0 mmol), amine (1.5 eq, 3.0 mmol), $Pd_2(dba)_3$ (18.3 mg, 0.02 mmol, 1 mol%, 2 mol% Pd), XPhos (76.3 mg, 0.16 mmol, 8 mol%), K_2CO_3 (553.0 mg, 4.0 mmol, 2 eq) and dry 1,4-dioxane (10.0 mL) as solvent. Afterwards, the Schlenk was sealed and set in an oil bath at 110 ºC during 15 h. When the reaction was complete, the crude reaction mixture was allowed to reach room temperature, passed to a funnel extraction, and 20 mL of distillated water were added. The aqueous phase was extracted three times with 20 mL of dichloromethane. The combined organic layers were dried with anhydrous Na_2SO_4 and filtered under gravity. The solvent was removed under reduced pressure. Finally, the reaction mixture was purified by silica gel column chromatography (*n*-heptane / ethyl acetate mixtures) obtaining the corresponding ring-substituted phthalimide derivative in (88-98%) yields.

Synthesis of hemiaminal (1al) from N-methylphthalimide (1a)[5]: A 50 mL Schlenk containing a stirring bar was sequentially charged under argon with N-methylphthalimide (**1a**) (1636.1 mg, 10.0 mmol), KOH (56.2 mmol, 1 mmol, 10.0 mol%) and dry DMF (10.0 mL). Next, PMHS (667.0 mg, 11.0 mmol, 1.1 equiv. based on H) was added slowly to the mixture under argon. The reaction mixture was stirred at 25 °C until **1a** was totally converted (cheeked by TLC) and the reaction mixture was quenched by adding NH_4OH (4 mL) and stirring at room temperature for two hours. Then, the mixture was treated with water (40 mL) and the aqueous phase was

extracted three times with 30 mL of dichloromethane. The combined organic layers were dried with anhydrous Na_2SO_4 and filtered under gravity. The solvent was removed under reduced pressure. Finally, the reaction mixture was purified by silica gel column chromatography (*n*-heptane / ethyl acetate mixtures) affording the hemiaminal **1al** in (71%) yield.

3. ADDITIONAL TABLES

Table S1. Reductive methoxylation of N-methylphthalimide (**1a**): Screening of the metal catalyst.

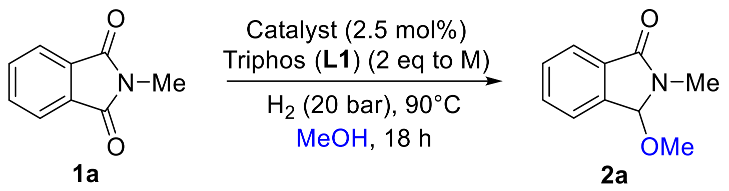

Entry[a]	Catalyst	Conv. (%)	**2a** (%)[b]
**1**	**$Co(BF_4)_2 \cdot 6H_2O$**	**>99**	**>99**
2	$Co(acac)_3$	19	-
3	$Co(acac)_2 \cdot H_2O$	3	-
4	$CoCl_2$	-	-
5	$Co(OAc)_2 \cdot 4H_2O$	5	-
6	CoF_2	<1	-
7	$CoBr_2$	-	-
8	$Co(NO_3)_2 \cdot 6H_2O$	-	-
9	$CoSO_4 \cdot 7H_2O$	-	-
10	$Co(ClO_4)_2 \cdot 6H_2O$	>99	89
11	$CoCO_3$	-	-
12	$Co(OH)_2$	-	-
13	$Ru(acac)_3$	-	-
14	$Cu(BF_4)_2 \cdot xH_2O$	-	-
15	$Fe(BF_4)_2 \cdot 6H_2O$	-	-
**16**	$Zn(BF_4)_2 \cdot xH_2O$	-	-

[a] Standard reaction conditions: N-methylphthalimide (**1a**) (82.2 mg, 0.5 mmol), catalyst (0.0125 mmol, 2.5 mol%), Triphos (**L1**) (15.6 mg, 0.025 mmol, 5 mol%), H_2 (20 bar), MeOH (2 mL), 90 °C and 18 h. [b] Conversion of **1a** and yields of **2a** were calculated by GC using hexadecane as internal standard.

Table S2. Reductive methoxylation of N-methylphthalimide (**1a**): Influence of the organic additives.

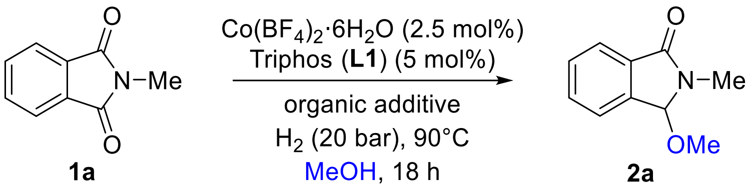

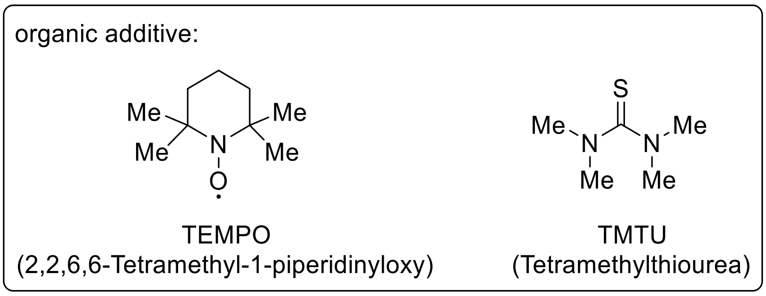

Entry[a]	Organic additive (eq to Co)	Conv. (%)	**2a** (%)[b]
1	none	>99	>99
2	TEMPO (0.25)	99	98
3	TEMPO (0.5)	>99	>99
4	TEMPO (1)	>99	>99
5	TMTU (0.25)	30	28
6	TMTU (0.5)	-	-

[a] Standard reaction conditions: N-methylphthalimide (**1a**) (82.2 mg, 0.5 mmol), $Co(BF_4)\cdot 6H_2O$ (4.25 mg, 0.0125 mmol, 2.5 mol%), Triphos (**L1**) (15.6 mg, 0.025 mmol, 5 mol%), organic additive (0.15-1 eq to Co), H_2 (20 bar), MeOH (2 mL), 90 °C and 18 h. [b] Conversion of **1a** and yields of **2a** were calculated by GC using hexadecane as internal standard.

4. ADDITIONAL SCHEMES

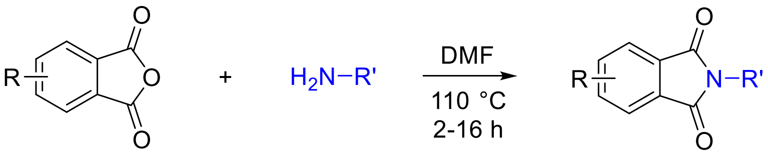

(1.2 eq)

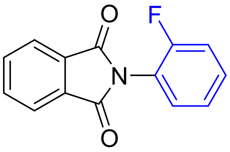

1g [65%]

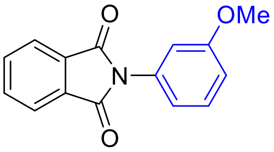

1i [88%]

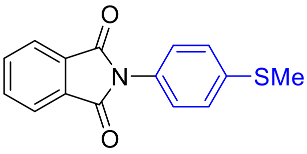

1j [81%]

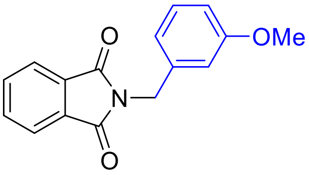

1n [82%]

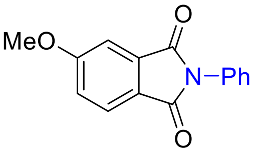

3e [61%]

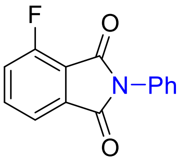

3f [63%]

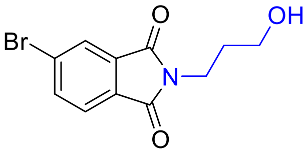

S1 [81%]

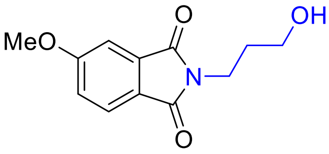

5c [76%]

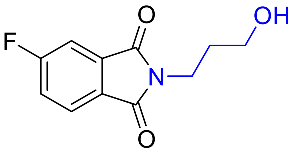

5d [70%]

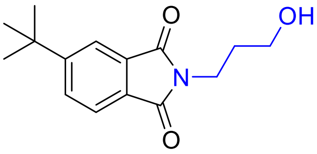

5e [98%]

Scheme S1. Synthesis of phthalimides by condensation reaction between an anhydride and the corresponding amine. In brackets are shown the isolated yields after purification by column chromatography on silica using (*n*-heptane/ethyl acetate) mixtures as eluent. Standard reaction conditions: anhydride (7.2 mmol, 1.2 eq), amine (6.0 mmol), dry DMF (10.0 mL) at 110 °C during 2-16 h.

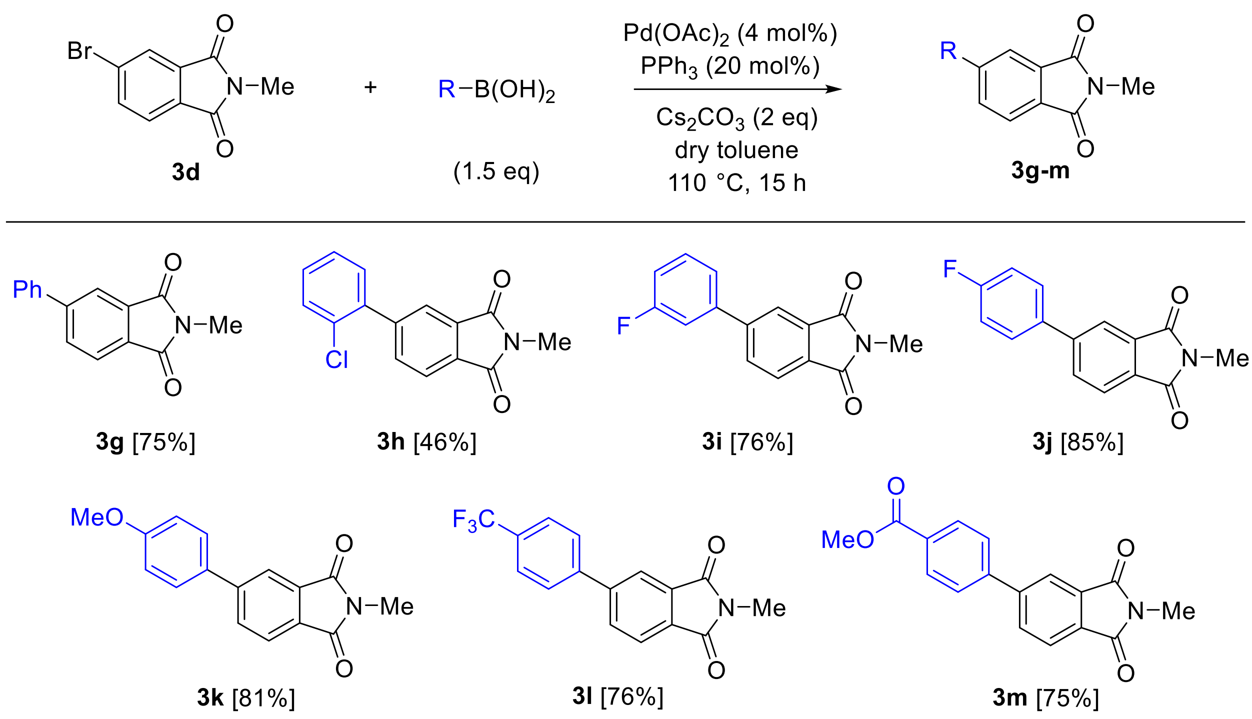

Scheme S2. Synthesis of several asymmetrical ring-substituted *N*-methylphthalimides using Suzuki-Miyaura cross-coupling between the corresponding phenylboronic acid and 4-Br-*N*-methylphthalimide (**3d**). In brackets are shown the isolated yields after purification by column chromatography on silica using (*n*-heptane/ethyl acetate) mixtures as eluent. Standard reaction conditions: 4-Br-*N*-methylphthalimide (**3d**) (480.12 mg, 2.0 mmol), organoborane (1.5 eq, 3.0 mmol), $Pd(OAc)_2$ (19.0 mg, 0.08 mmol, 4 mol%), PPh_3 (105.2 mg, 0.4 mmol, 20 mol%), Cs_2CO_3 (1.3 g, 4.0 mmol, 2 eq), dry toluene (10.0 mL) at 110 °C over 15 h.

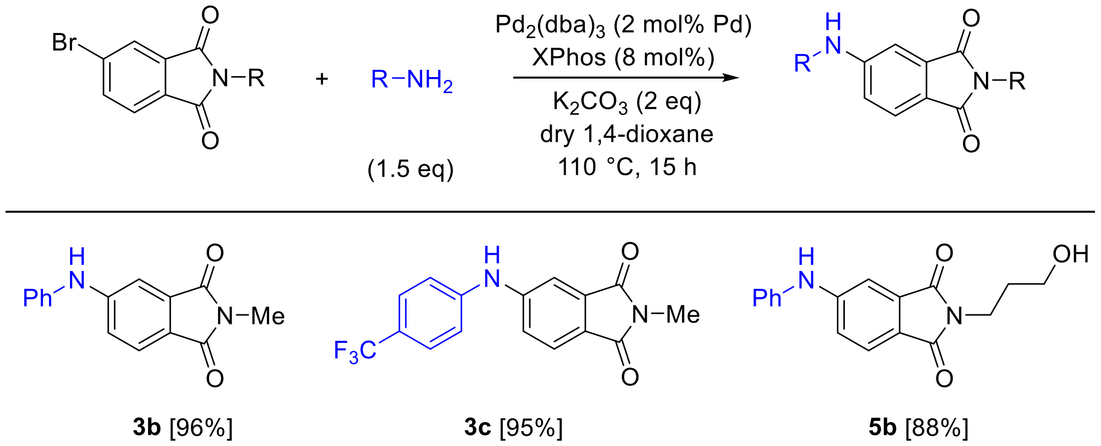

Scheme S3. Synthesis of ring-substituted phthalimides using Buchwald-Hartwig amination. In brackets are shown the isolated yields after purification by column chromatography on silica using (*n*-heptane/ethyl acetate) mixtures as eluent. Standard reaction conditions: phthalimide derivative (2.0 mmol), amine (3.0 mmol, 1.5 eq), $Pd_2(dba)_3$ (18.3 mg, 0.02 mmol, 1 mol%, 2 mol% Pd), XPhos (76.3 mg, 0.16 mmol, 8 mol%), K_2CO_3 (553.0 mg, 4.0 mmol, 2 eq), dry 1,4-dioxane (10.0 mL) at 110 °C over 15 h.

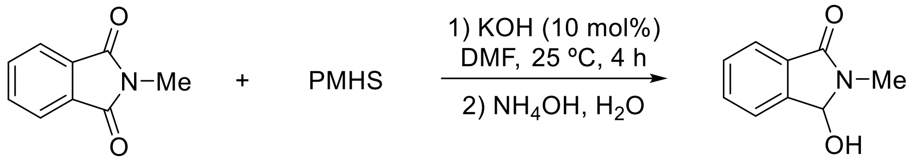

1a (1.1 eq of H) **1al** [71%]

Scheme S4. Synthesis of the intermediate hemiaminal **1al** from N-methylphthalimide (**1a**) using a previously reported methodology [5] based on hydrosilanes. In brackets is shown the isolated yield after purification by column chromatography on silica using (*n*-heptane/ethyl acetate) mixtures as eluent. Standard reaction conditions: 1) N-methylphthalimide (**1a**) (10.0 mmol, 1636.1 mg), PMHS (11.0 mmol, 667.0 mg), dry DMF (10.0 mL) at 25 °C during 4 h; 2) NH_4OH (4 mL) at 25 °C during 2 h.

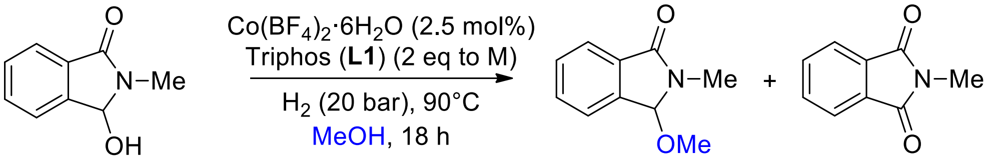

1al

Variation to standard cond.[a]	Conv. of **1al** (%)[b]	**2a** (%)[b]	**1a** (%)[b]
reaction time (0.5 h)	43	42	-
reaction time (2 h)	>99	98	-
standard conditions	>99	98	-
0.5 mol% of Co	75	74	-
without Triphos (**L1**)	>99	98	-
without Co/Triphos (**L1**)	-	-	-
without H_2	>99	78	19
without Triphos (**L1**) and H_2	>99	98	-

Scheme S5. Control experiments using the hemiaminal intermediate (**1al**) as starting material. [a] Standard reaction conditions: 3-hydroxy-2-methylisoindolin-1-one (**1al**) (81.6 mg, 0.5 mmol), $Co(BF_4)·6H_2O$ (4.25 mg, 0.0125 mmol, 2.5 mol%), Triphos (**L1**) (15.6 mg, 0.025 mmol, 5 mol%), H_2 (20 bar), MeOH (2 mL), 90 °C and 18 h. [b] Conversion of **1al** and yields of **2a** and **3a** were calculated by GC and GC-MS using hexadecane as internal standard.

5. ADDITIONAL FIGURES

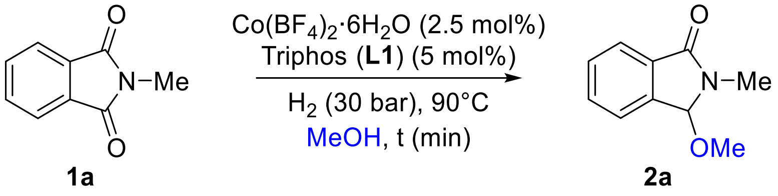

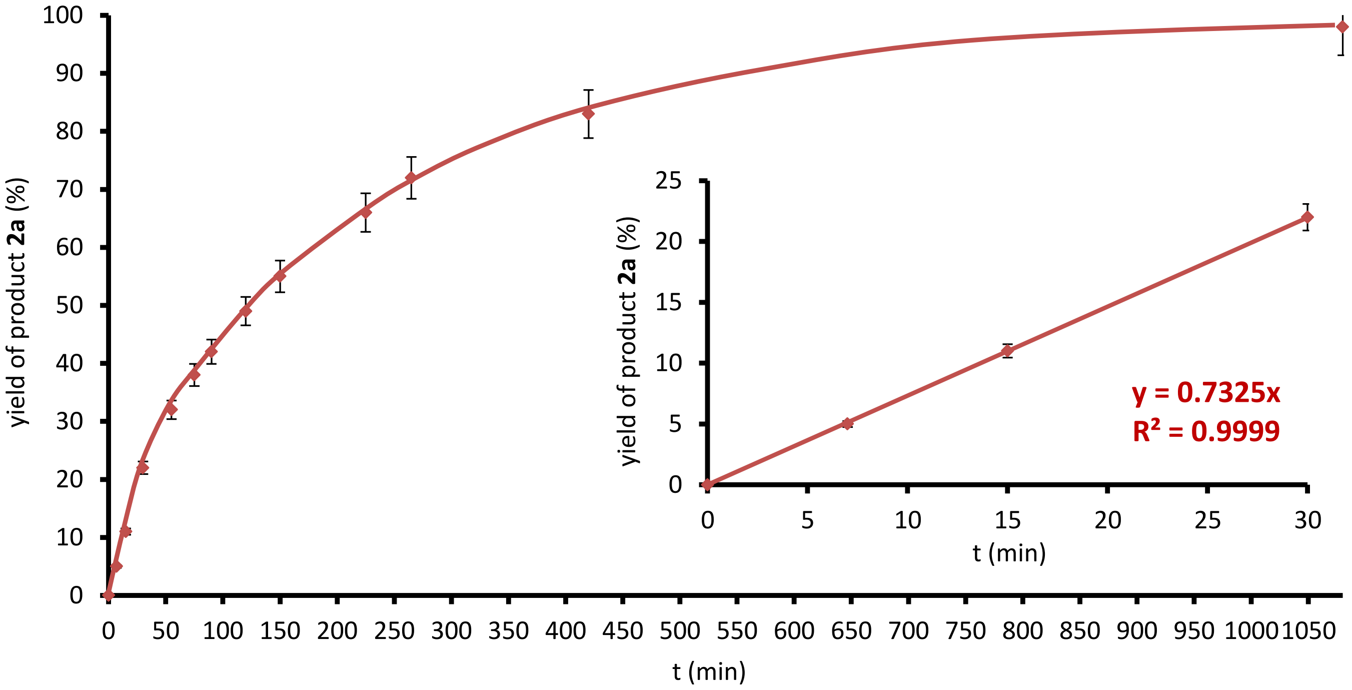

Figure S1. Yield/time kinetic profile for the formation of product **2a** in the reductive methoxylation of N-methylphthalimide (**1a**) using methanol and molecular hydrogen (30 bar) at 90 °C. Inset: Yield/time kinetic profile at initial times of the reaction. Standard reaction conditions: N-methylphthalimide (**1a**) (493.3 mg, 3.0 mmol), $Co(BF_4)_2{\cdot}6H_2O$ (25.5 mg, 0.075 mmol, 2.5 mol%), Triphos (**L1**) (100.5 mg, 0.15 mmol, 5 mol%), MeOH (12.0 mL) and H_2 (30 bar) at 90 °C. Yields of product **2a** were calculated by GC using hexadecane as internal standard. Vertical error bar (5%) for all data points is shown.

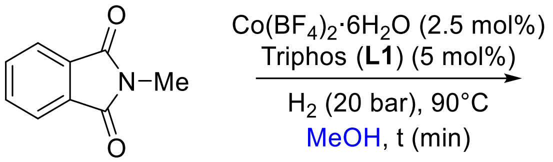

1a **2a**

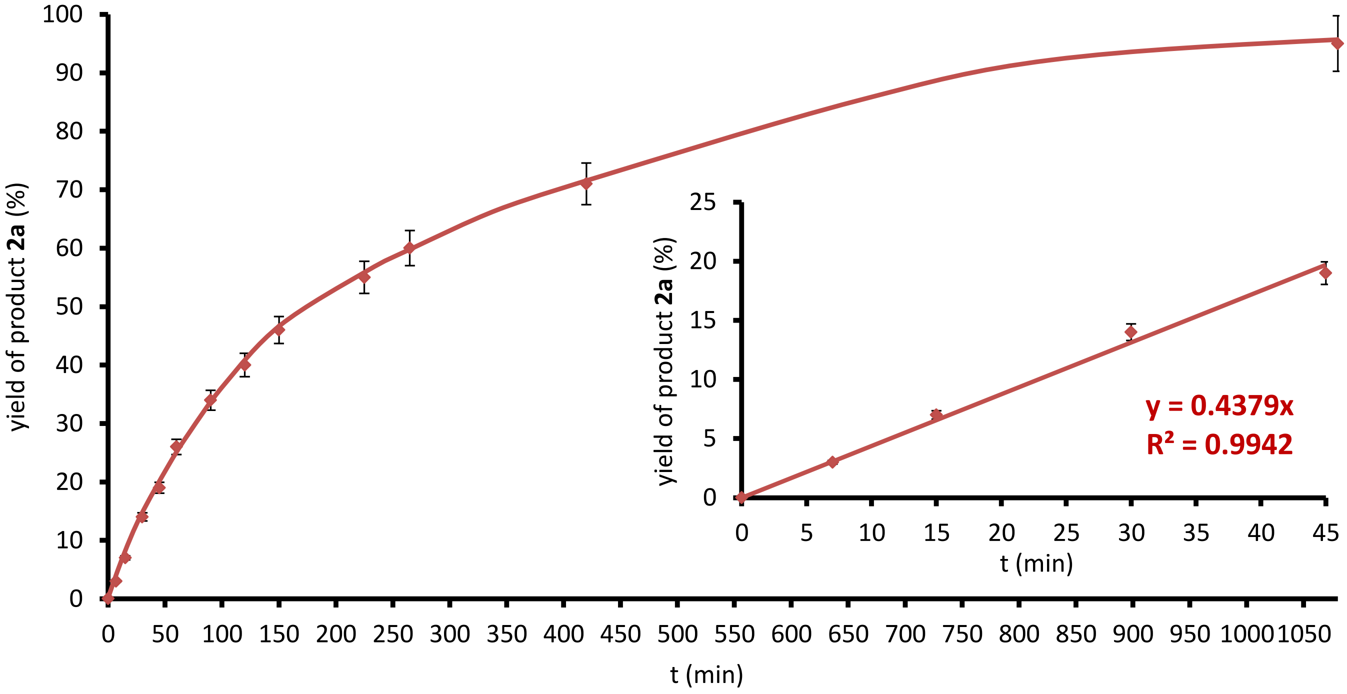

Figure S2. Yield/time kinetic profile for the formation of product **2a** in the reductive methoxylation of N-methylphthalimide (**1a**) using methanol and molecular hydrogen (20 bar) at 90 °C. Inset: Yield/time kinetic profile at initial times of the reaction. Standard reaction conditions: N-methylphthalimide (**1a**) (493.3 mg, 3.0 mmol), $Co(BF_4)_2{\cdot}6H_2O$ (25.5 mg, 0.075 mmol, 2.5 mol%), Triphos (**L1**) (100.5 mg, 0.15 mmol, 5 mol%), MeOH (12.0 mL) and H_2 (20 bar) at 90 °C. Yields of product **2a** were calculated by GC using hexadecane as internal standard. Vertical error bar (5%) for all data points is shown.

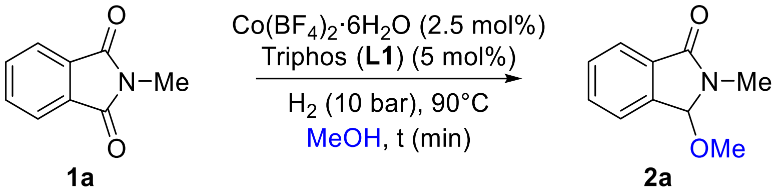

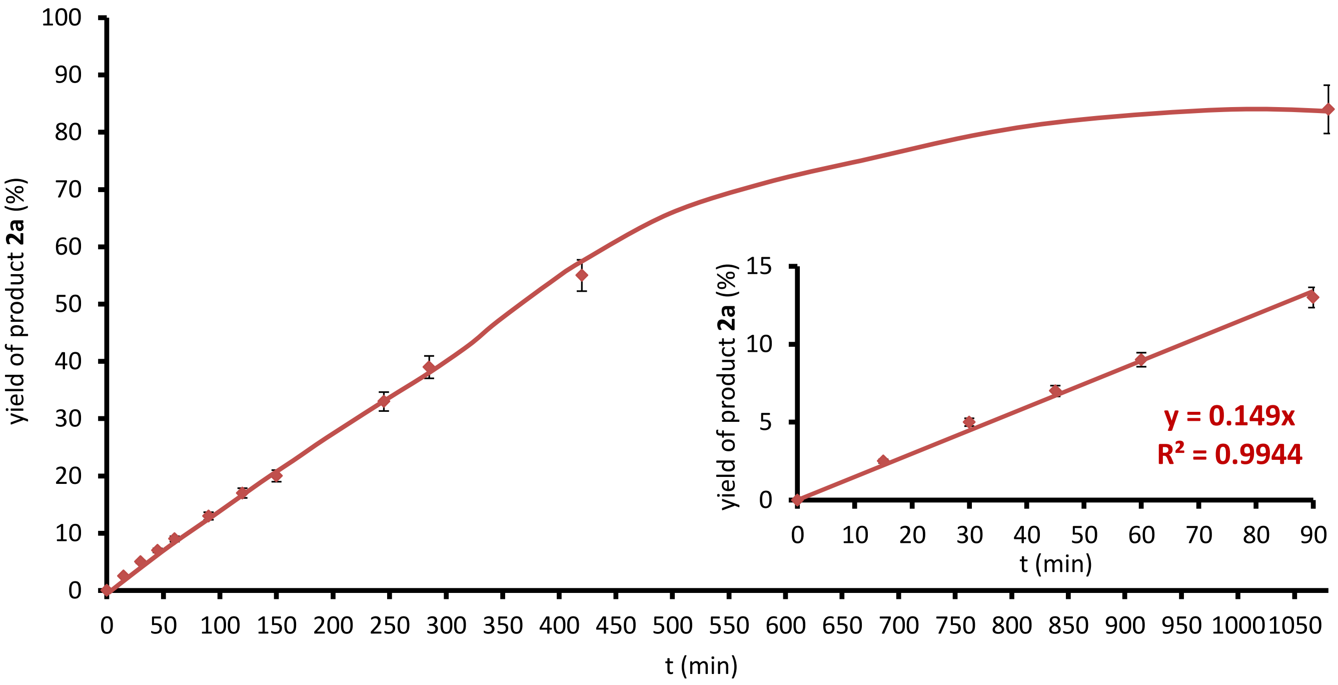

Figure S3. Yield/time kinetic profile for the formation of product **2a** in the reductive methoxylation of N-methylphthalimide (**1a**) using methanol and molecular hydrogen (10 bar) at 90 °C. Inset: Yield/time kinetic profile at initial times of the reaction. Standard reaction conditions: N-methylphthalimide (**1a**) (493.3 mg, 3.0 mmol), $Co(BF_4)_2{\cdot}6H_2O$ (25.5 mg, 0.075 mmol, 2.5 mol%), Triphos (**L1**) (100.5 mg, 0.15 mmol, 5 mol%), MeOH (12.0 mL) and H_2 (10 bar) at 90 °C. Yields of product **2a** were calculated by GC using hexadecane as internal standard. Vertical error bar (5%) for all data points is shown.

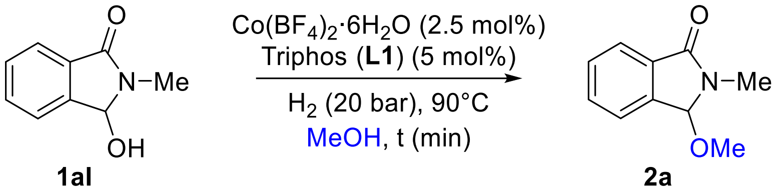

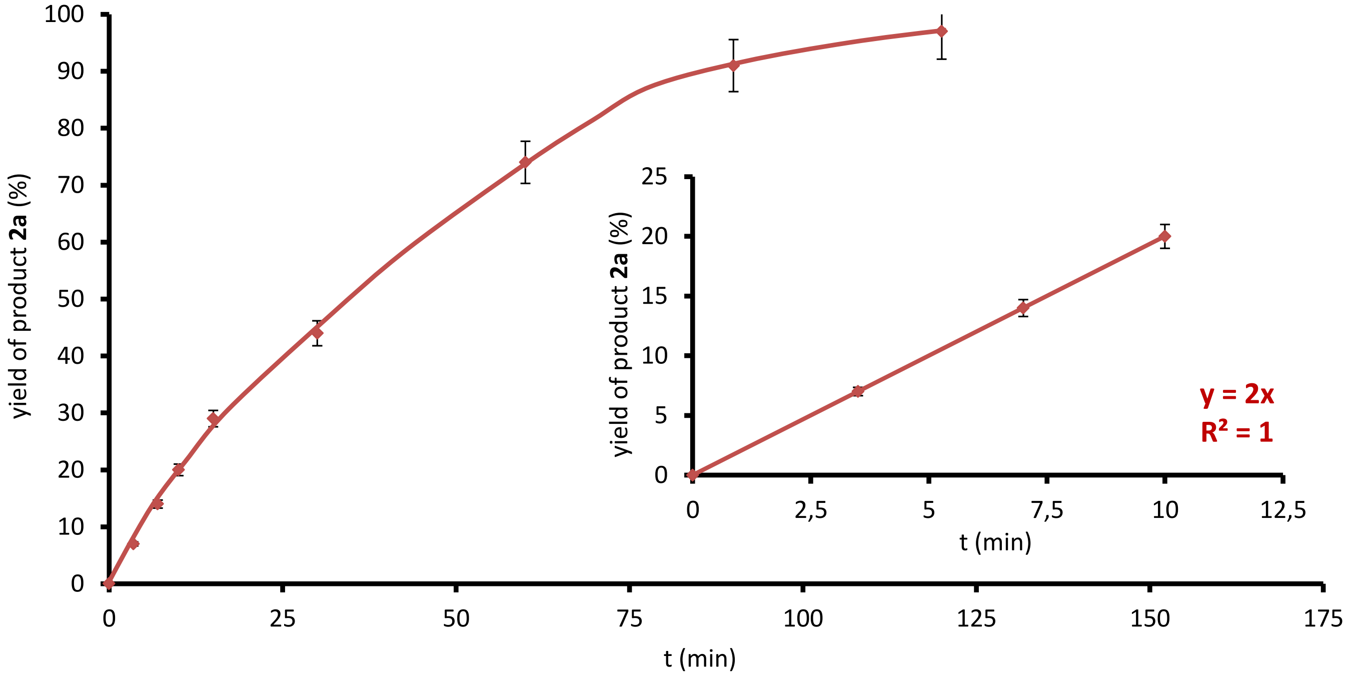

Figure S4. Yield/time kinetic profile for the formation of product **2a** from intermediate hemiaminal (**1al**) using methanol and molecular hydrogen (20 bar) at 90 °C. Inset: Yield/time kinetic profile at initial times of the reaction. Standard reaction conditions: 3-hydroxy-2-methylisoindolin-1-one (**1al**) (489.5 mg, 3.0 mmol), $Co(BF_4)_2{\cdot}6H_2O$ (25.5 mg, 0.075 mmol, 2.5 mol%), Triphos (**L1**) (100.5 mg, 0.15 mmol, 5 mol%), MeOH (12.0 mL) and H_2 (30 bar) at 90 °C. Yields of product **2a** were calculated by GC using hexadecane as internal standard. Vertical error bar (5%) for all data points is shown.

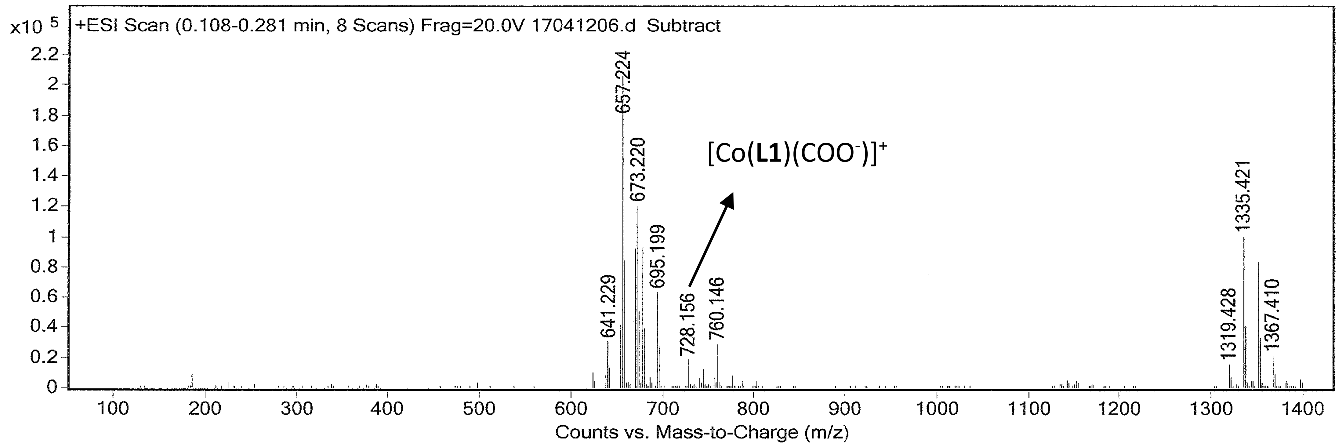

Figure S5. ESI-MS analysis (MeOH / 0.1% HCOOH) of the fresh mixture [Co(BF_4)$_2$·6H_2O/Triphos (**L1**)] with [1/2] molar ratio.

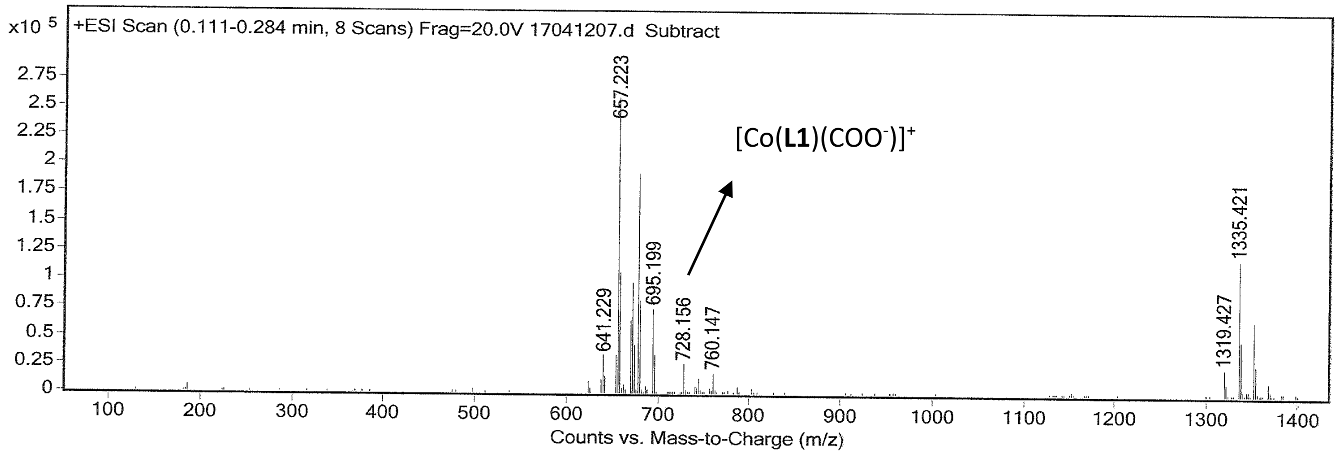

Figure S6. . ESI-MS analysis (MeOH / 0.1% HCOOH) of the fresh mixture [Co(BF_4)$_2$·6H_2O/Triphos (**L1**)/N-methylphthalimide (**1a**)] with [1/2/40] molar ratio.

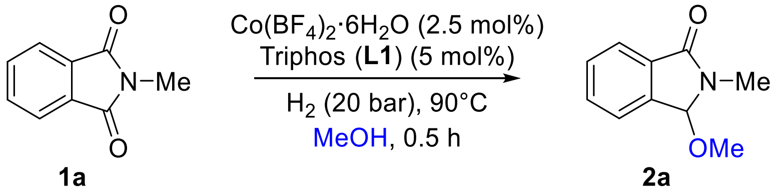

A)

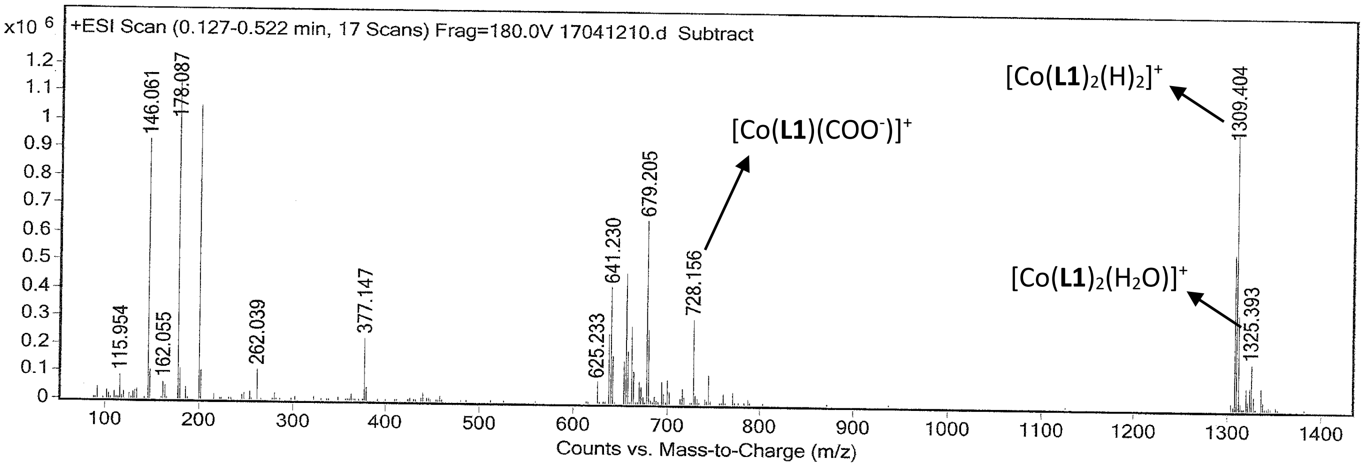

B)

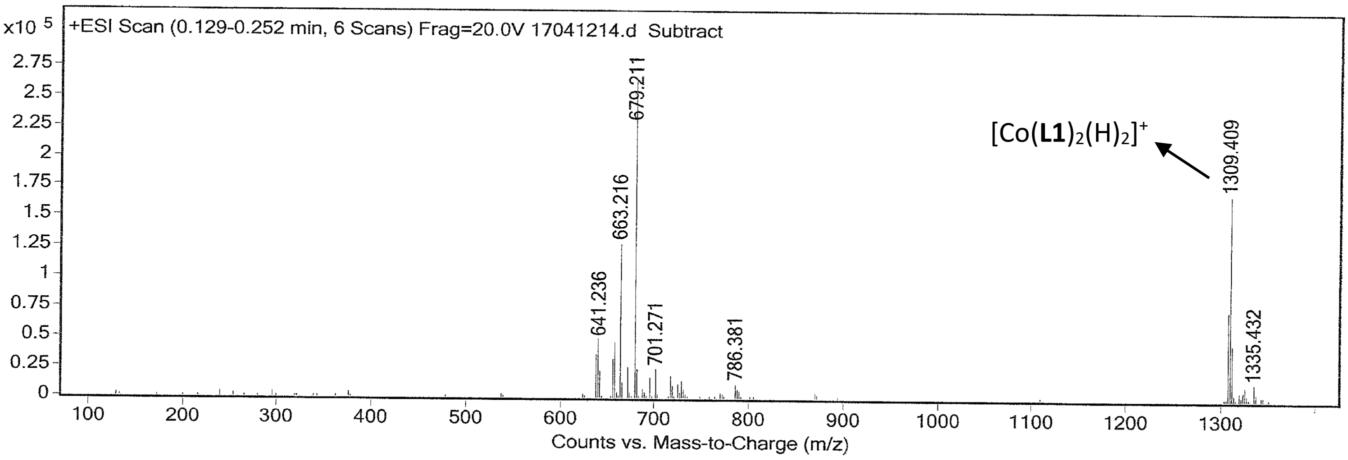

Figure S7. ESI-MS analysis at 0.5 h of reaction time for the cobalt-catalysed reductive methoxylation of **1a** under standard conditions: A) using MeOH / 0.1% HCOOH or B) using acetonitrile.

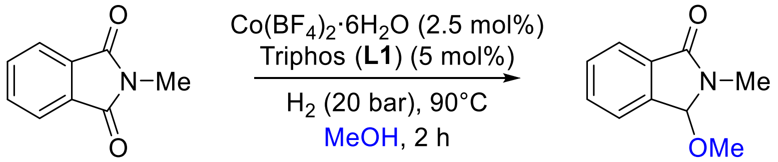

1a **2a**

A)

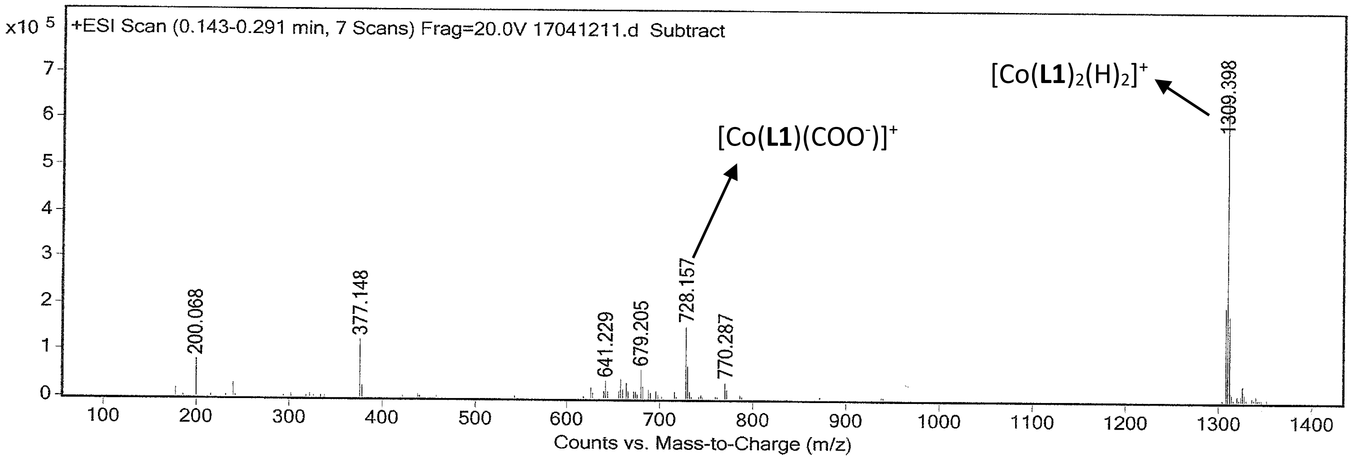

B)

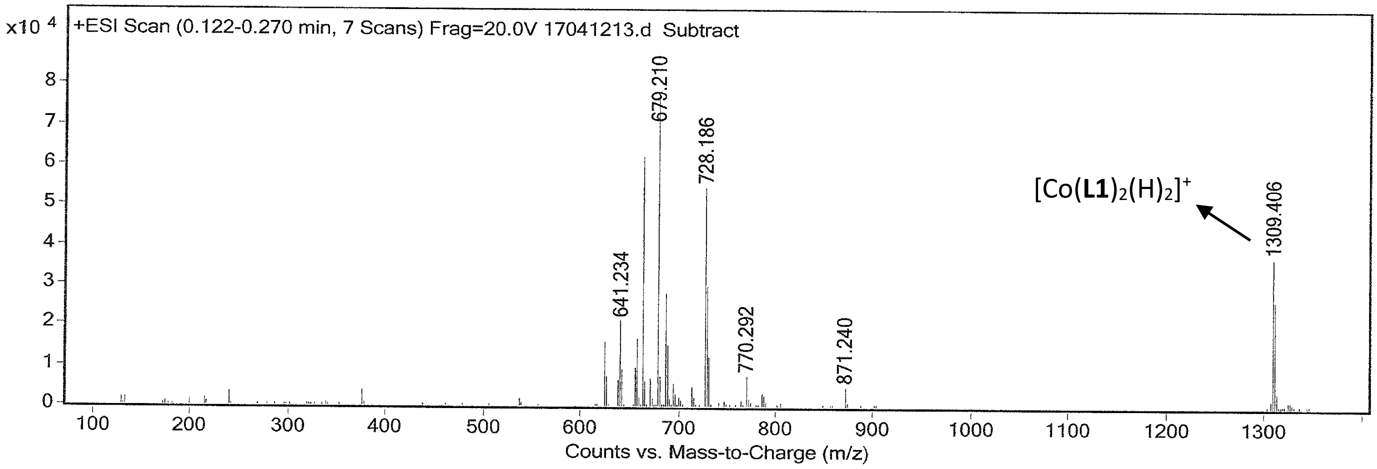

Figure S8. ESI-MS analysis at 2 h of reaction time for the cobalt-catalysed reductive methoxylation of **1a** under standard conditions: A) using MeOH / 0.1% HCOOH or B) using acetonitrile.

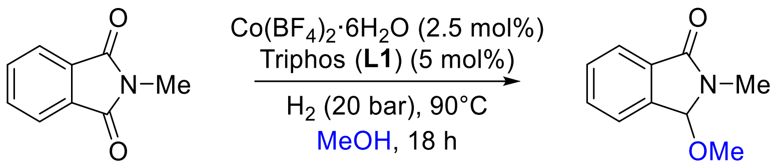

A)

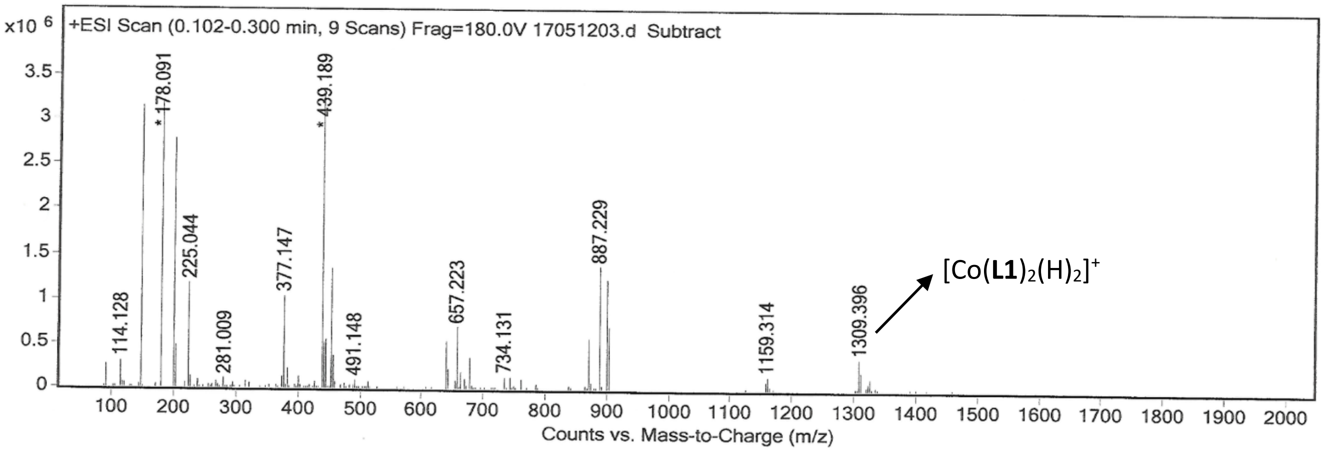

B)

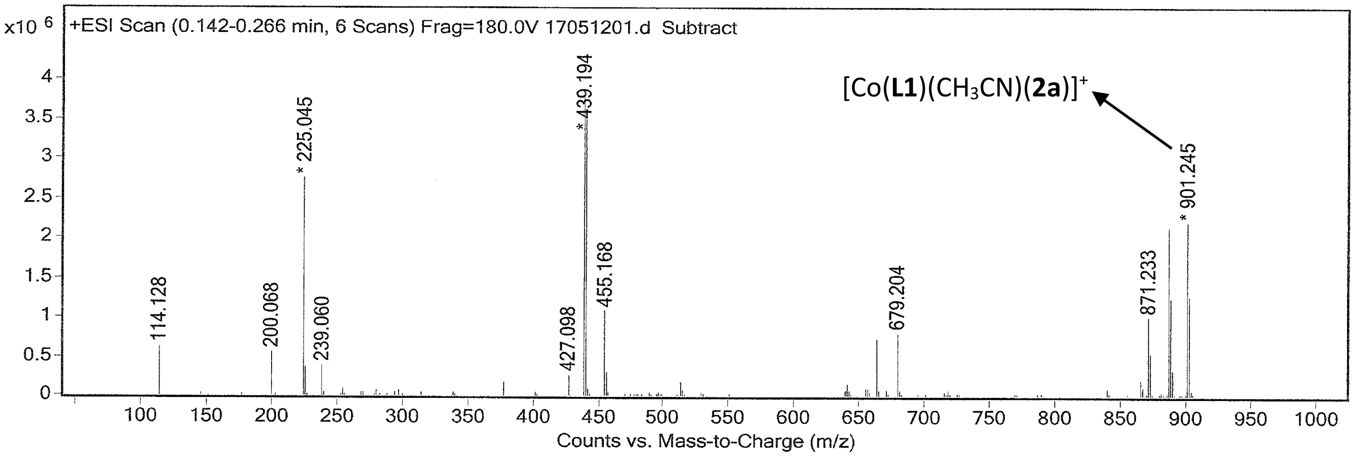

Figure S9. ESI-MS analysis at 18 h of reaction time for the cobalt-catalysed reductive methoxylation of **1a** under standard conditions: A) using MeOH / 0.1% HCOOH or B) using acetonitrile.

6. CHARACTERIZATION DATA OF THE ISOLATED PRODUCTS

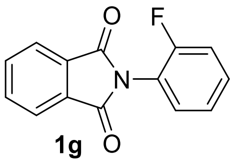

1g

***N*-(2-fluorophenyl)phthalimide (1g).[6]** Isolated yield: 65%. GC-MS (m/z, $M^{+\cdot}$ 241), major peaks found: 241 (100%), 197 (90%), 104 (20%), 76 (35%), 50 (8%). (The NMR spectrum is consistent with the reported data). ^{1}H NMR (300 MHz, $CDCl_3$) δ: 7.91-7.83 (m, 2H), 7.75-7.67 (m, 2H), 7.41-7.25 (m, 2H), 7.24-7.13 (m, 2H). ^{13}C NMR (75 MHz, $CDCl_3$) δ: 166.61 (2xC=O), 157.96 (d, $J^1_{C\text{-}F}$ = 252.6, C), 134.57 (2xCH), 132.01 (C), 130.84 (d, $J^3_{C\text{-}F}$ = 7.9, CH), 129.96 (CH), 124.74 (d, $J^4_{C\text{-}F}$ = 3.9, CH), 124.01 (2xCH), 119.45 (d, $J^2_{C\text{-}F}$ = 13.3, C), 116.84 (d, $J^2_{C\text{-}F}$ = 19.6, CH). ^{19}F NMR (282 MHz, $CDCl_3$) δ: -118.21-(-118.40) (m, 1F). HRMS (ESI) [M+Na$^+$; calculated for $C_{14}H_8O_2NF$: 264.0431] found m/z 264.0433.

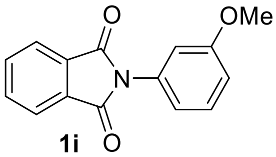

1i

***N*-(3-methoxyphenyl)phthalimide (1i).[7]** Isolated yield: 88%. GC-MS (m/z, $M^{+\cdot}$ 253), major peaks found: 253 (50%), 224 (10%), 209 (12%), 179 (12%), 104 (15%), 76 (25%). (The NMR spectrum is consistent with the reported data). ^{1}H NMR (300 MHz, $CDCl_3$) δ: 7.95 (dd, *J* = 5.5, 3.0, 2H), 7.79 (dd, *J* = 5.5, 3.1, 2H), 7.41 (ddd, *J* = 8.3, 8.3, 0.5, 1H), 7.03 (ddd, *J* = 7.9, 1.9, 0.9, 1H), 7.00-6.93 (m, 2H), 3.84 (s, 3H). ^{13}C NMR (75 MHz, $CDCl_3$) δ: 167.34 (2xC=O), 160.17 (C), 134.53 (2xCH), 132.79 (C), 131.87 (2xC), 129.93 (CH), 123.88 (2xCH), 119.00 (CH), 114.24 (CH), 112.48 (CH), 55.55 (O-CH_3). HRMS (ESI) [M+Na$^+$; calculated for $C_{15}H_{11}O_3N$: 276.0631] found m/z 276.0635.

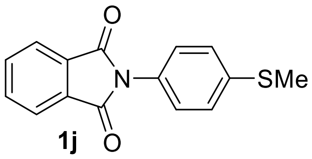

1j

***N*-(4-methylthiophenyl)phthalimide (1j).[8]** Isolated yield: 81%. GC-MS (m/z, $M^{+\cdot}$ 269), major peaks found: 269 (100%), 254 (40%), 122 (10%), 104 (8%), 76 (15%). (The NMR spectrum is consistent with the reported data). ^{1}H NMR (300 MHz, $CDCl_3$) δ: 7.94 (dd, *J* = 5.5, 3.0, 2H), 7.78 (dd, *J* = 5.5, 3.1, 2H), 7.36 (bs, 4H), 2.51 (s, 3H). ^{13}C NMR (75 MHz, $CDCl_3$) δ: ^{13}C NMR (75 MHz, $CDCl_3$) δ 167.37 (2xC=O), 139.02 (C), 134.54 (2xCH), 131.85 (2xC), 128.68 (C), 127.03 (2xCH), 126.94 (2xCH), 123.86 (2xCH), 15.91 (S-CH_3). HRMS (ESI) [M+H$^+$; calculated for $C_{15}H_{11}O_3NS$: 286.0532] found m/z 286.0534.

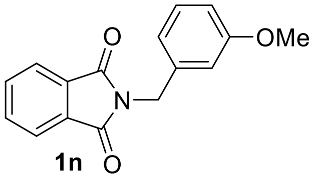

1n

N-(3-methoxybenzyl)phthalimide (1n).[9] Isolated yield: 82%. GC-MS (m/z, $M^{+\cdot}$ 267), major peaks found: 267 (100%), 249 (10%), 234 (12%), 206 (8%), 134 (14%), 77 (6%). (The NMR spectrum is consistent with the reported data). ^{1}H NMR (300 MHz, $CDCl_3$) δ: 7.83 (dd, J = 5.5, 3.0, 2H), 7.69 (dd, J = 5.4, 3.1, 2H), 7.22 (t, J = 7.9, 1H), 7.00 (d, J = 7.6, 1H), 6.98-6.95 (m, 1H), 6.80 (dd, J = 8.2, 2.5, 1H), 4.81 (s, 2H), 3.78 (s, 3H). ^{13}C NMR (75 MHz, $CDCl_3$) δ: 168.11 (2xC=O), 159.87 (C), 137.93 (C), 134.08 (2xCH), 132.20 (2xC), 129.80 (CH), 123.44 (2xCH), 120.88 (CH), 114.12 (CH), 113.49 (CH), 55.31 (O-CH_3), 41.63 (N-CH_2). HRMS (ESI) [$M+H^+$; calculated for $C_{16}H_{13}O_3N$: 268.0968] found m/z 268.0964.

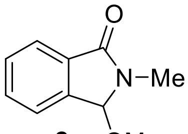

2a

3-methoxy-2-methylisoindolin-1-one (2a).[10] Isolated yield: 95%. GC-MS (m/z, $M^{+\cdot}$ 176), major peaks found: 176 (5%), 146 (100%), 91 (8%). (The NMR spectrum is consistent with the reported data). ^{1}H NMR (300 MHz, $CDCl_3$) δ: 7.81-7.76 (m, 1H), 7.58-7.52 (m, 1H), 7.51-7.45 (m, 2H), 5.72 (s, 1H), 3.05 (s, 3H), 2.86 (s, 3H). ^{13}C NMR (75 MHz, $CDCl_3$) δ: 167.78 (C=O), 140.30 (C), 133.20 (C), 131.99 (CH), 129.99 (CH), 123.39 (2xCH), 88.03 (O-CH-N), 49.19 (O-CH_3), 26.48 (N-CH_3). HRMS (ESI) [M^+; calculated for $C_{10}H_{11}O_2N$: 177.0784] found m/z 177.0780.

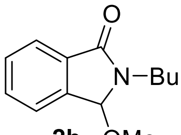

2b

2-buthyl-3-methoxyisoindolin-1-one (2b).[11] Isolated yield: 89%. GC-MS (m/z, $M^{+\cdot}$ 219), major peaks found: 219 (15%), 204 (83%), 188 (35%), 176 (37%), 146 (100%), 132 (42%). (The NMR spectrum is consistent with the reported data). ^{1}H NMR (300 MHz, $CDCl_3$) δ: 7.78 (ddd, J = 6.6, 1.6, 1.2, 1H), 7.59-7.43 (m, 3H), 5.84 (s, 1H), 3.74 (dt, J = 13.5, 7.8, 1H), 3.18 (ddd, J = 13.5, 7.7, 6.2, 1H), 2.84 (s, 3H), 1.67-1.54 (m, 2H), 1.35 (sext, J = 7.5, 2H), 0.91 (t, J = 7.3, 3H). ^{13}C NMR (75 MHz, $CDCl_3$) δ: 167.70 (C=O), 140.34 (C), 133.29 (C), 131.93 (CH), 129.95 (CH), 123.45 (CH), 123.42 (CH), 86.21 (O-CH-N), 49.13 (O-CH_3), 39.27 (N-CH_2), 30.27 (CH_2) 20.31 (CH_2), 13.85 (CH_3). HRMS (ESI) [M^+; calculated for $C_{13}H_{17}O_2N$: 219.1253] found m/z 219.1251.

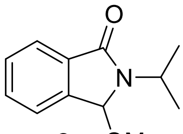

2c

2-isopropyl-3-methoxyisoindolin-1-one (2c).[11] Isolated yield: 90%. GC-MS (m/z, $M^{+\cdot}$ 205), major peaks found: 205 (5%), 190 (100%), 174 (45%), 158 (33%), 132 (73%). (The NMR spectrum is consistent with the reported

data). ^{1}H NMR (300 MHz, $CDCl_3$) δ: 7.82-7.72 (m, 1H), 7.61-7.42 (m, 3H), 5.99 (s, 1H), 4.37 (hept, *J* = 6.9, 1H), 2.88 (s, 3H), 1.38 (t, *J* = 6.9, 3H), 1.36 (t, *J* = 6.8, 3H).^{13}C NMR (75 MHz, $CDCl_3$) δ: 167.70 (C=O), 140.51 (C), 133.38 (C), 131.92 (CH), 129.87 (CH), 123.41 (CH), 123.28 (CH), 85.69 (O-CH-N), 48.94 ($O-CH_3$), 43.91 (N-CH), 21.09 (CH_3), 20.13 (CH_3). HRMS (ESI) [M^+; calculated for $C_{12}H_{15}O_2N$: 205.1097] found m/z 205.1096.

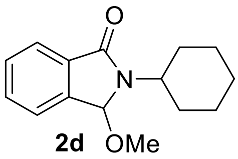

2d

2-cyclohexyl-3-methoxyisoindolin-1-one (2d). Isolated yield: 96%. GC-MS (m/z, $M^{+\cdot}$ 245), major peaks found: 245 (20%), 230 (35%), 214 (30%), 202 (100%), 164 (45%), 148 (17%), 132 (95%), 104 (9%), 77 (12%). ^{1}H NMR (400 MHz, $CDCl_3$) δ: 7.78-7.69 (m, 1H), 7.53-7.48 (m, 1H), 7.46-7.41 (m, 2H), 5.96 (s, 1H), 3.96 (tt, *J* = 11.9, 3.8, 1H), 2.83 (s, 3H), 1.95-1.58 (m, 7H), 1.42-1.27 (m, 2H), 1.23-1.09 (m, 1H). ^{13}C NMR (101 MHz, $CDCl_3$) δ: 167.55 (C=O), 140.45 (C), 133.27 (C), 131.79 (CH), 129.74 (CH), 123.34 (CH), 123.17 (CH), 85.67 (O-CH-N), 51.91 (N-CH), 48.78 ($O-CH_3$), 31.24 (CH_2), 30.46 (CH_2), 26.01 (CH_2), 25.96 (CH_2), 25.47 (CH_2). HRMS (ESI) [$M+H^+$; calculated for $C_{15}H_{19}O_2N$: 246.1488] found m/z 246.1490.

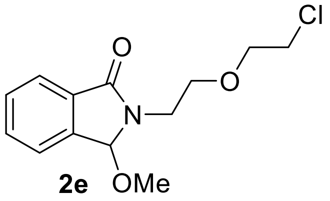

2e

2-(2-(2-chloroethoxy)ethyl)-3-methoxyisoindolin-1-one (2e).[11] Isolated yield: 97%. GC-MS (m/z, $M^{+\cdot}$ 269), major peaks found: 269 (2%), 256 (8%), 254 (25%), 240 (4%), 238 (11%), 206 (13%), 176 (80%), 146 (100%), 117 (15%). (The NMR spectrum is consistent with the reported data). ^{1}H NMR (300 MHz, $CDCl_3$) δ: 7.80 (dt, *J* = 7.2, 1.1, 1H), 7.60-7.46 (m, 3H), 6.07 (s, 1H), 3.97 (dt, *J* = 14.3, 4.3, 1H), 3.78-3.63 (m, 4H), 3.60-3.54 (m, 2H), 3.42 (dt, *J* = 14.4, 6.0, 1H), 2.90 (s, 3H). ^{13}C NMR (75 MHz, $CDCl_3$) δ: 167.83 (C=O), 140.88 (C), 132.91 (C), 132.08 (CH), 129.88 (CH), 123.49 (CH), 123.44 (CH), 87.46 (O-CH-N), 70.88 ($O-CH_2$), 69.45 ($O-CH_2$), 49.54 ($O-CH_3$), 42.92 ($N-CH_2$), 38.91 (CH_2-Cl). HRMS (ESI) [M^+; calculated for $C_{13}H_{16}O_3NCl$ (^{37}Cl): 271.0783] found m/z 271.0788 and [M^+; calculated for $C_{13}H_{16}O_3NCl$ (^{35}Cl): 269.0813] found m/z 269.0814.

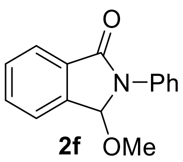

2f

3-methoxy-2-phenylisoindolin-1-one (2f).[11] Isolated yield: 93%. GC-MS (m/z, $M^{+\cdot}$ 239), major peaks found: 239 (43%), 224 (43%), 208 (100%), 152 (6%), 77 (20%). (The NMR spectrum is consistent with the reported data). ^{1}H NMR (300 MHz, $CDCl_3$) δ: 7.84 (d, *J* = 7.3, 1H), 7.77-7.70 (m, 2H), 7.61-7.46 (m, 3H), 7.41-7.31 (m, 2H), 7.19-7.10 (m, 1H), 6.39 (s, 1H), 2.83 (s, 3H). ^{13}C NMR (75 MHz, $CDCl_3$) δ: 166.78 (C=O), 139.80 (C), 137.38 (C), 132.99 (C),

132.89 (CH), 130.40 (CH), 129.19 (CH), 125.40 (CH), 124.01 (CH), 123.53 (CH), 121.79 (CH), 87.35 (O-CH-N), 49.21 (O-CH_3). HRMS (ESI) [M^+; calculated for $C_{15}H_{13}O_2N$: 239.0940] found m/z 239.0942.

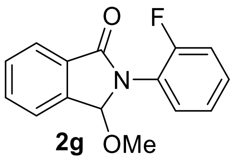

2g

2-(2-fluorophenyl)-3-methoxyisoindolin-1-one (2g). Isolated yield: 88%. GC-MS (m/z, $M^{+\cdot}$ 257), major peaks found: 257 (20%), 242 (90%), 226 (100%), 197 (6%), 170 (8%), 151 (7%). ^{1}H NMR (300 MHz, $CDCl_3$) δ: 7.83 (d, *J* = 7.3, 1H), 7.59-7.53 (m, 1H), 7.52-7.46 (m, 2H), 7.45-7.38 (m, 1H), 7.27-7.18 (m, 1H), 7.18-7.13 (m, *J* = 6.2, 4.5, 1H), 7.12-7.06 (m, 1H), 6.33 (s, 1H), 2.91 (s, 3H). ^{13}C NMR (75 MHz, $CDCl_3$) δ: 166.61 (C=O), 157.76 (d, J^1_{C-F} = 250.4, C), 140.72 (C), 132.78 (CH), 132.21 (C), 130.20 (CH), 128.96 (d, J^3_{C-F} = 6.3, CH), 128.90 (CH), 124.70 (d, J^4_{C-F} = 3.7, CH), 124.13 (CH), 123.82 (d, J^2_{C-F} = 12.0, C), 123.70 (CH), 116.76 (d, J^2_{C-F} = 20.2, CH), 88.27 (d, J^4_{C-F} = 4.7, CH), 50.33 (O-CH_3). ^{19}F NMR (282 MHz, $CDCl_3$) δ: -118.08-(-120.91) (m, 1F). HRMS (ESI) [M+H^+; calculated for $C_{15}H_{12}O_2NF$: 258.0924] found m/z 258.0922.

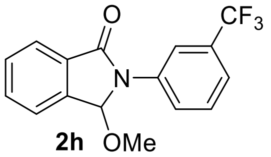

2h

3-methoxy-2-(3-(trifluoromethyl)phenyl)isoindolin-1-one (2h). Isolated yield: 91%. GC-MS (m/z, $M^{+\cdot}$ 307), major peaks found: 307 (8%), 292 (80%), 276 (100%), 272 (70%), 145 (9%), 127 (8%). ^{1}H NMR (300 MHz, $CDCl_3$) δ: 7.95-7.90 (m, 1H), 7.81 (dd, *J* = 7.8, 1.4, 1H), 7.71-7.63 (m, 2H), 7.62-7.54 (m, 3H), 7.51-7.40 (m, 1H), 6.14 (s, 1H), 3.15 (s, 3H). ^{13}C NMR (75 MHz, $CDCl_3$) δ: 167.88 (C=O), 141.18 (C), 134.36 (q, J^4_{C-F} = 2.0, C), 133.17 (CH), 132.94 (CH), 132.85 (CH), 131.84 (C), 130.31 (CH), 129.13 (CH), 127.77 (q, J^3_{C-F} = 4.8, CH), 125.35, 124.41 (CH), 123.87 (CH), 121.73, 90.27 (O-CH-N), 52.60 (O-CH_3). ^{19}F NMR (282 MHz, $CDCl_3$) δ: -60.32 (s, 3F). HRMS (ESI) [M+H^+; calculated for $C_{16}H_{12}O_2NF_3$: 308.0892] found m/z 308.0894.

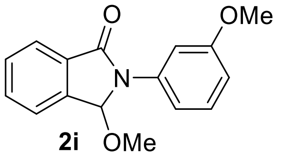

2i

3-methoxy-2-(3-methoxyphenyl)isoindolin-1-one (2i). Isolated yield: 98%. GC-MS (m/z, $M^{+\cdot}$ 269), major peaks found: 269 (45%), 254 (30%), 238 (100%), 167 (8%), 92 (10%). ^{1}H NMR (300 MHz, $CDCl_3$) δ: 7.90 (d, *J* = 7.3 Hz, 1H), 7.69-7.62 (m, 1H), 7.62-7.56 (m, 2H), 7.56-7.52 (m, 1H), 7.41-7.36 (m, 1H), 7.33 (t, *J* = 8.0, 1H), 6.78 (ddd, *J* = 7.8, 2.4, 1.3, 1H), 6.43 (s, 1H), 3.84 (s, 3H), 2.91 (s, 3H). ^{13}C NMR (75 MHz, $CDCl_3$) δ: 166.79 (C=O), 160.19 (C), 139.76 (C), 138.62 (C), 132.93 (CH), 132.91 (CH), 130.37 (CH), 129.80 (CH), 123.93 (C), 123.49 (CH), 113.70 (CH), 111.03 (CH), 107.53 (CH), 87.42 (O-CH-N), 55.38 (O-CH_3), 49.18 (O-CH_3). HRMS (ESI) [M+H^+; calculated for $C_{16}H_{15}O_3N$: 270.1124] found m/z 270.1123.

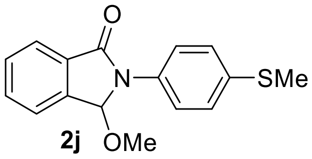

2j

3-methoxy-2-(4-(methylthio)phenyl)isoindolin-1-one (2j). Isolated yield: 95%. GC-MS (m/z, $M^{+\cdot}$ 285), major peaks found: 285 (100%), 270 (35%), 254 (98%), 239 (10%), 127 (8%). ^{1}H NMR (300 MHz, $CDCl_3$) δ: 7.89 (d, *J* = 7.2, 1H), 7.76 (d, *J* = 8.9, 1H), 7.68-7.61 (m, 1H), 7.61-7.53 (m, 3H), 7.32 (d, *J* = 8.9, 2H), 6.41 (s, 1H), 2.89 (s, 3H), 2.48 (s, 3H). ^{13}C NMR (75 MHz, $CDCl_3$) δ: 166.59 (C=O), 139.65 (C), 134.98 (C), 134.75 (C), 132.86 (C), 132.83 (CH), 130.35 (CH), 127.60 (2xCH), 123.88 (CH), 123.45 (CH), 122.04 (2xCH), 87.25 (O-CH-N), 49.12 (O-CH_3), 16.29 (S-CH_3). HRMS (ESI) [M+H$^+$; calculated for $C_{16}H_{15}O_2SN$: 286.0896] found m/z 286.0897.

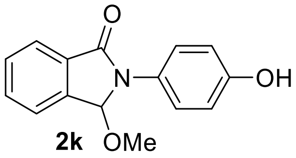

2k

2-(4-hydroxyphenyl)-3-methoxyisoindolin-1-one (2k). Isolated yield: 96%. GC-MS (m/z, $M^{+\cdot}$ 255), major peaks found: 255 (50%), 240 (35%), 224 (100%), 167 (8%), 130 (6%). ^{1}H NMR (300 MHz, DMSO-*d*$_6$) δ: 9.63 (bs, OH), 7.81-7.70 (m, 2H), 7.68-7.59 (m, 2H), 7.43 (d, *J* = 8.8, 2H), 6.84 (d, *J* = 8.8, 2H), 6.55 (s, 1H), 2.85 (s, 3H). ^{13}C NMR (75 MHz, DMSO-*d*$_6$) δ: 165.88 (C=O), 155.49 (C), 140.36 (C), 132.95 (CH), 132.43 (C), 130.46 (CH), 128.28 (C), 125.32 (2xCH), 123.96 (CH), 123.26 (CH), 115.62 (2xCH), 87.52 (O-CH-N), 49.73 (O-CH_3). HRMS (ESI) [M+H$^+$; calculated for $C_{15}H_{13}O_3N$: 256.0968] found m/z 256.0970.

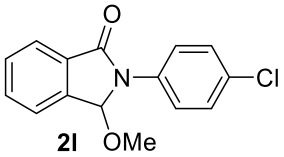

2l

2-(4-chlorophenyl)-3-methoxyisoindolin-1-one (2l).[11] Isolated yield: 94%. GC-MS (m/z, $M^{+\cdot}$ 273), major peaks found: 275 (15%), 273 (45%), 260 (20%), 258 (60%), 244 (33%), 242 (100%), 152 (5%). (The NMR spectrum is consistent with the reported data). ^{1}H NMR (300 MHz, $CDCl_3$): 7.89-7.84 (m, 1H), 7.83-7.76 (m, 2H), 7.68-7.61 (m, 1H), 7.59-7.52 (m, 2H), 7.38-7.31 (m, 2H), 6.39 (s, 1H), 2.86 (s, 3H). ^{13}C NMR (75 MHz, $CDCl_3$): 166.56 (C=O), 139.49 (C), 136.02 (C), 133.01 (CH), 132.61 (C), 130.41 (CH), 130.26 (C), 129.08 (2xCH), 123.93 (CH), 123.47 (CH), 122.38 (2xCH), 87.12 (O-CH-N), 49.02 (O-CH_3). HRMS (ESI) [M+H$^+$; calculated for $C_{15}H_{12}O_2NCl$ (^{37}Cl): 276.0604] found m/z 276.0603 and [M+H$^+$; calculated for $C_{15}H_{12}O_2NCl$ (^{35}Cl): 274.0629] found m/z 276.0632.

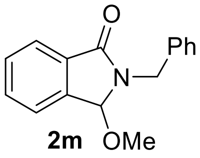

2m

2-benzyl-3-methoxyisoindolin-1-one (2m).[11] Isolated yield: 99%. GC-MS (m/z, $M^{+\cdot}$ 253), major peaks found: 253 (45 %), 222 (50 %), 133 (47 %), 91 (100 %). (The NMR spectrum is consistent with the reported data). ^{1}H

NMR (300 MHz, $CDCl_3$) δ: 7.80-7.72 (m, 1H), 7.48-7.39 (m, 2H), 7.39-7.34 (m, 1H), 7.29-7.10 (m, 5H), 5.60 (s, 1H), 5.07 (d, *J* = 14.7, 1H), 4.11 (d, *J* = 14.7, 1H), 2.77 (s, 3H). ^{13}C NMR (75 MHz, $CDCl_3$) δ: 167.42 (C=O), 140.41 (C), 136.82 (C), 132.90 (C), 132.07 (CH), 129.92 (CH), 128.67 (2xCH), 128.59 (2xCH), 127.60 (CH), 123.60 (CH), 123.47 (CH), 85.60 (O-CH-N), 49.32 (O-CH_3), 43.09 (CH_2). HRMS (ESI) [M^+; calculated for $C_{16}H_{15}O_2N$: 253.1097] found m/z 253.1099.

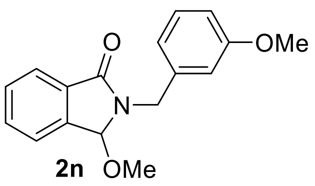

2n

3-methoxy-2-(3-methoxybenzyl)isoindolin-1-one (2n). Isolated yield: 99%. GC-MS (m/z, $M^{+\cdot}$ 283), major peaks found: 283 (25 %), 268 (23 %), 251 (70 %), 160 (30 %), 133 (33%), 121 (100%), 91 (23%). ^1H NMR (300 MHz, $CDCl_3$): 7.85 (d, *J* = 6.6, 1H), 7.58-7.43 (m, 3H), 7.21 (t, *J* = 7.9, 1H), 6.96-6.89 (m, 2H), 6.79 (dd, *J* = 8.2, 2.3, 1H), 5.70 (s, 1H), 5.14 (d, *J* = 14.6, 1H), 4.16 (d, *J* = 14.7, 1H), 3.75 (s, 3H), 2.87 (s, 3H). ^{13}C NMR (75 MHz, $CDCl_3$): 167.40 (C=O), 159.88 (C), 140.45 (C), 138.37 (C), 132.89 (C), 132.08 (CH), 129.91 (CH), 129.69 (CH), 123.61 (CH), 123.48 (CH), 120.81 (CH), 114.02 (CH), 113.17 (CH), 85.61 (O-CH-N), 55.16 (O-CH_3), 49.36 (O-CH_3), 43.03 (N-CH_2). HRMS (ESI) [M+H^+; calculated for $C_{17}H_{17}O_3N$: 284.1281] found m/z 284.1281.

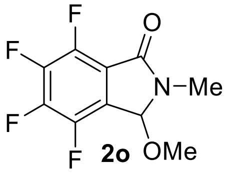

2o

4,5,6,7-tetrafluoro-3-methoxy-2-methylisoindolin-1-one (2o).[11] Isolated yield: 86%. GC-MS (m/z, $M^{+\cdot}$ 248), major peaks found: 248 (2%), 218 (100%), 191 (6%), 177 (5%), 163 (5%), 149 (4%). (The NMR spectrum is consistent with the reported data). ^1H NMR (300 MHz, $CDCl_3$) δ: 5.85 (s, 1H), 3.07 (s, 3H), 3.01 (s, 3H). ^{13}C NMR (75 MHz, $CDCl_3$) δ: 161.90 (d, $J^3_{C\text{-}F}$ = 1.9, C=O), 145.54-143.59 (m, 2xC-F), 142.01-140.19 (m, 2xC-F), 122.44-122.04 (m, C), 116.02 (dt, $J^2_{C\text{-}F}$ = 10.9, $J^3_{C\text{-}F}$ = 2.9, C), 85.86 (d, $J^3_{C\text{-}F}$ = 0.9, O-CH-N), 50.99 (O-CH_3), 26.66 (N-CH_3). ^{19}F NMR (282 MHz, $CDCl_3$) δ: -141.11 (td, *J* = 19.2, 6.4, 1F), -141.35 (td, *J* = 19.5, 3.7, 1F), -147.51 (td, *J* = 19.4, 6.7, 1F), -149.83-(-150.06) (m, 1F). HRMS (ESI) [M^+; calculated for $C_{10}H_7O_2F_4N$: 249.0407] found m/z 249.0408.

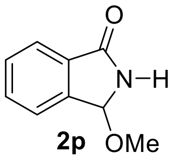

2p

3-methoxyisoindolin-1-one (2p).[12] Isolated yield: 89%. GC-MS (m/z, $M^{+\cdot}$ 163), major peaks found: 163 (5%), 148 (12%), 132 (100%), 104 (15%), 77 (15%). (The NMR spectrum is consistent with the reported data). ^1H NMR (300 MHz, $CDCl_3$) δ: 8.26 (bs, NH), 7.81 (d, *J* = 7.3, 1H), 7.61-7.46 (m, 3H), 5.93 (s, 1H), 3.22 (s, 3H). ^{13}C NMR (75 MHz, $CDCl_3$) δ: 170.96 (C=O), 142.96 (C), 132.57 (CH), 132.26 (C), 129.94 (CH), 123.81 (CH), 123.59 (CH), 84.75 (O-CH-N), 52.21 (O-CH_3). HRMS (ESI) [M+H^+; calculated for $C_9H_9O_2N$: 164.0706] found m/z 164.0705.

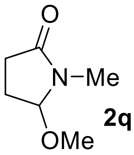

2q

5-methoxy-1-methylpyrrolidin-2-one (2q).[11] Isolated yield: 81%. GC-MS (m/z, $M^{+\cdot}$ 128), major peaks found: 128 (5%), 98 (100%), 74 (5%), 42 (10%). (The NMR spectrum is consistent with the reported data). ^{1}H NMR (300 MHz, $CDCl_3$) δ: 4.85 (dd, *J* = 6.4, 1.5, 1H), 3.26 (s, 2H), 2.85 (s, 3H), 2.49 (quint, *J* = 8.3, 1H), 2.30 (ddd, *J* = 17.0, 10.1, 3.1, 1H), 2.22-2.08 (m, 1H), 2.02-1.91 (m, 1H). ^{13}C NMR (75 MHz, $CDCl_3$) δ: 175.21 (C=O), 91.91 (O-CH-N), 52.89 (O-CH_3), 28.98 (CH_2), 27.84 (N-CH_3), 23.92 (CH_2). HRMS (ESI) [M^+H^+; calculated for $C_6H_{11}O_2N$: 130.0862] found m/z 130.0863.

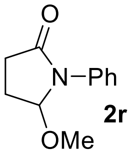

2r

5-methoxy-1-phenylpyrrolidin-2-one (2r).[11] Isolated yield: 89%. GC-MS (m/z, $M^{+\cdot}$ 191), major peaks found: 191 (55%), 160 (100%), 132 (30%), 104 (21%), 77 (27%). (The NMR spectrum is consistent with the reported data). ^{1}H NMR (300 MHz, $CDCl_3$): 7.53-7.47 (m, 2H), 7.41-7.32 (m, 2H), 7.24-7.16 (m, 1H), 5.31 (dd, *J* = 5.9, 1.1, 1H), 3.26 (s, 3H), 2.74 (dt, *J* = 17.3, 9.4, 1H), 2.47 (ddd, *J* = 17.3, 9.6, 2.5, 1H), 2.32-2.17 (m, 1H), 2.16-2.05 (m, 1H). ^{13}C NMR (75 MHz, $CDCl_3$): 174.39 (C=O), 137.93 (C), 128.98 (2xCH), 126.02 (CH), 123.17 (2xCH), 92.01 (O-CH-N), 53.58 (O-CH_3), 29.92 (CH_2), 24.42 (CH_2). HRMS (ESI) [$M+H^+$; calculated for $C_{11}H_{13}O_2N$: 192.1019] found m/z 192.1018.

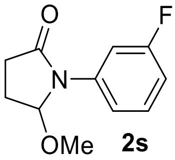

2s

1-(3-fluorophenyl)-5-methoxypyrrolidin-2-one (2s). Isolated yield: 94%. GC-MS (m/z, $M^{+\cdot}$ 209), major peaks found: 209 (65%), 178 (100%), 150 (50%), 135 (15%), 122 (35%), 109 (10%), 95 (38%), 71 (19%). ^{1}H NMR (300 MHz, $CDCl_3$) δ: 7.43-7.35 (m, 1H), 7.34-7.26 (m, 2H), 6.97-6.79 (m, 1H), 5.31 (dd, *J* = 5.8, 1.2, 1H), 3.29 (s, 3H), 2.74 (dt, *J* = 17.4, 9.5, 1H), 2.48 (ddd, *J* = 17.4, 9.3, 2.6, 1H), 2.30-2.18 (m, 1H), 2.18-2.08 (m, 1H). ^{13}C NMR (75 MHz, $CDCl_3$) δ: 174.34 (C=O), 162.79 (d, $J^1_{C\text{-}F}$ = 245.0, C), 139.51 (d, $J^3_{C\text{-}F}$ = 10.4, C), 130.03 (d, $J^3_{C\text{-}F}$ = 9.1, CH), 117.73 (d, $J^4_{C\text{-}F}$ = 3.1, CH), 112.45 (d, $J^2_{C\text{-}F}$ = 21.1,CH), 109.83 (d, $J^2_{C\text{-}F}$ = 25.3, CH), 91.61 (O-CH-N), 53.27 (O-CH_3), 29.98 (CH_2), 24.02 (CH_2). ^{19}F NMR (282 MHz, $CDCl_3$) δ: -110.47-(-112.02) (m, 1F). HRMS (ESI) [$M+H^+$; calculated for $C_{11}H_{12}O_2NF$: 210.0924] found m/z 210.0925.

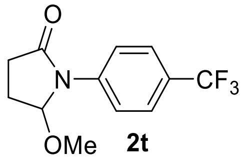

2t

5-methoxy-1-(4-(trifluoromethyl)phenyl)pyrrolidin-2-one (2t). Isolated yield: 85%. GC-MS (m/z, $M^{+\cdot}$ 259), major peaks found: 259 (40%), 240 (10%), 228 (100%), 200 (35%), 172 (25%), 130 (9%), 95 (8%), 71 (19%). ^{1}H NMR (300 MHz, $CDCl_3$) δ: 7.71 (d, *J* = 8.6, 2H), 7.60 (d, *J* = 8.8, 2H), 5.36 (dd, *J* = 5.7, 1.3, 1H), 3.30 (s, 3H), 2.77 (dt, *J* = 17.5, 9.5, 1H), 2.51 (ddd, *J* = 17.5, 9.2, 2.8, 1H), 2.32-2.10 (m, 2H). ^{13}C NMR (75 MHz, $CDCl_3$) δ: 174.52 (C=O), 141.19 (C), 127.29 (q, $J^2_{C\text{-}F}$ = 32.6, C), 126.09 (q, $J^3_{C\text{-}F}$ = 3.8, 2xCH), 124.09 (q, $J^1_{C\text{-}F}$ = 271.8, CF_3), 122.00 (2xCH), 91.37 (O-CH-N), 53.21 (O-CH_3), 30.03 (CH_2), 23.96 (CH_2). ^{19}F NMR (282 MHz, $CDCl_3$) δ: -61.96 (s, 1F). HRMS (ESI) [M+H$^+$; calculated for $C_{12}H_{12}O_2NF_3$: 260.0892] found m/z 260.0894.

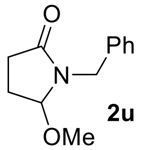

2u

1-benzyl-5-methoxypyrrolidin-2-one (2u).[11] Isolated yield: 80%. GC-MS (m/z, $M^{+\cdot}$ 205), major peaks found: 205 (35%), 174 (30%), 146 (40%), 104 (21%), 91 (100%), 65 (15%). (The NMR spectrum is consistent with the reported data). ^{1}H NMR (300 MHz, $CDCl_3$): 7.29-7.16 (m, 5H), 4.88 (d, *J* = 14.7, 1H), 4.65 (dd, *J* = 6.2, 1.7, 1H), 3.94 (d, *J* = 15.2, 1H), 3.14 (s, 3H), 2.58-2.43 (m, 1H), 2.30 (ddd, *J* = 17.3, 9.6, 3.5, 1H), 2.09-1.87 (m, 2H). ^{13}C NMR (75 MHz, $CDCl_3$): 174.87 (C=O), 136.47 (C), 128.68 (2xCH), 128.45 (2xCH), 127.61 (CH), 89.00 (O-CH-N), 52.97 (O-CH_3), 43.83 (N-CH_2), 29.07 (CH_2), 23.76 (CH_2). HRMS (ESI) [M^+; calculated for $C_{12}H_{15}O_2N$: 205.1097] found m/z 205.1101.

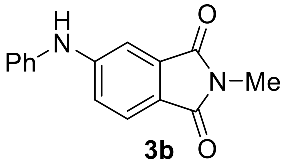

3b

2-methyl-5-(phenylamino)isoindoline-1,3-dione (3b).[13] Isolated yield: 96%. GC-MS (m/z, $M^{+\cdot}$ 252), major peaks found: 252 (100%), 208 (8%), 194 (12%), 167 (25%), 139 (5%). (The NMR spectrum is consistent with the reported data). ^{1}H NMR (300 MHz, $CDCl_3$): 7.63 (d, *J* = 8.3, 1H), 7.40-7.33 (m, 3H), 7.22-7.09 (m, 4H), 6.46 (bs, NH), 3.12 (s, 3H). ^{13}C NMR (75 MHz, $CDCl_3$): 168.81 (C=O), 168.60 (C=O), 150.06 (C), 140.00 (C), 135.01 (C), 129.86 (2xCH), 125.07 (CH), 124.39 (CH), 121.89 (C), 121.59 (2xCH), 118.56 (CH), 109.02 (CH), 23.96 (N-CH_3). HRMS (ESI) [M+H$^+$; calculated for $C_{15}H_{12}N_2O_2$: 253.0971] found m/z 253.0973.

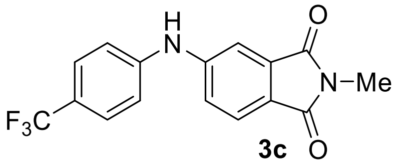

3c

2-methyl-5-((4-(trifluoromethyl)phenyl)amino)isoindoline-1,3-dione (3c). Isolated yield: 95%. GC-MS (m/z, $M^{+\cdot}$ 320), major peaks found: 320 (100%), 301 (8%), 276 (16%), 263 (14%), 235 (17%). ^{1}H NMR (300 MHz, $CDCl_3$) δ: 7.72 (d, J = 8.2, 1H), 7.60 (d, J = 8.6, 2H), 7.49 (d, J = 2.1, 1H), 7.26-7.21 (m, 3H), 6.49 (bs, NH), 3.15 (s, 3H). ^{13}C NMR (75 MHz, DMSO-d_6): 167.83 (C=O), 167.65 (C=O), 148.07 (C), 144.82 (C), 134.25 (C), 126.76 (q, J^{3}_{C-F} = 3.5, 2xCH), 124.78 (CH), 124.54 (q, J^{1}_{C-F} = 271.1, CF_3) 122.11 (C), 121.54 (d, J^{2}_{C-F} = 32.1, C), 119.85 (CH), 118.00 (2xCH), 109.60 (CH), 23.63 (N-CH_3). ^{19}F NMR (282 MHz, $CDCl_3$) δ: -61.58 (s, 3F). HRMS (ESI) [M+H$^+$; calculated for $C_{16}H_{11}O_2N_2F_3$: 321.0845] found m/z 321.0846.

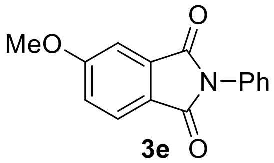

3e

5-methoxy-2-phenylisoindoline-1,3-dione (3e). Isolated yield: 61%. GC-MS (m/z, $M^{+\cdot}$ 253), major peaks found: 253 (100%), 209 (30%), 166 (12%), 134 (10%), 106 (16%), 63 (14%). ^{1}H NMR (300 MHz, $CDCl_3$) δ: 7.85 (d, J = 8.3, 1H), 7.54-7.47 (m, 2H), 7.46-7.35 (m, 4H), 7.23 (dd, J = 8.3, 2.3, 1H), 3.95 (s, 3H). ^{13}C NMR (75 MHz, $CDCl_3$) δ: 167.24 (C=O), 167.14 (C=O), 165.10 (C), 134.52 (C), 131.96 (C), 129.18 (2xCH), 128.07 (CH), 126.65 (2xCH), 125.58 (CH), 123.74 (C), 120.56 (CH), 108.31 (CH), 56.29 (O-CH_3). HRMS (ESI) [M+Na$^+$; calculated for $C_{15}H_{11}O_3N$: 276.0631] found m/z 276.0633.

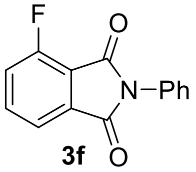

3f

4-fluoro-2-phenylisoindoline-1,3-dione (3f).[14] Isolated yield: 63%. GC-MS (m/z, $M^{+\cdot}$ 241), major peaks found: 241 (100%), 197 (80%), 170 (5%), 122 (10%), 94 (45%). (The NMR spectrum is consistent with the reported data). ^{1}H NMR (300 MHz, $CDCl_3$) δ: 7.83-7.74 (m, 2H), 7.55-7.48 (m, 2H), 7.48-7.38 (m, 4H). ^{13}C NMR (75 MHz, $CDCl_3$) δ: 166.24 (d, J^{3}_{C-F} = 3.0, C=O), 164.02 (d, J^{4}_{C-F} = 1.4, C=O), 158.02 (d, J^{1}_{C-F} = 266.6, C), 137.09 (d, J^{3}_{C-F} = 7.6, CH), 134.04 (d, J^{3}_{C-F} = 1.0, C), 131.38 (C), 129.29 (2xCH), 128.34 (CH), 126.71 (2xCH), 122.86 (d, J^{2}_{C-F} = 19.7, CH), 120.09 (d, J^{4}_{C-F} = 3.8, CH), 117.69 (d, J^{2}_{C-F} = 12.3, C). ^{19}F NMR (282 MHz, $CDCl_3$) δ: -110.44-(-112.96) (m, 1F). HRMS (ESI) [M+H$^+$; calculated for $C_{14}H_8O_2NF$: 242.0611] found m/z 242.0611.

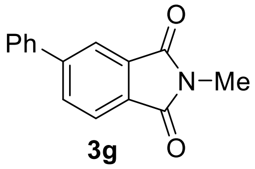

3g

***N*-methyl-4-phenylphthalimide (3g).**[15] Isolated yield: 75%. GC-MS (m/z, $M^{+\cdot}$ 237), major peaks found: 237 (100%), 209 (17%), 180 (15%), 152 (32%), 102 (29%), 76 (14%). (The NMR spectrum is consistent with the reported data). ^{1}H NMR (300 MHz, $CDCl_3$) δ: 8.06 (t, *J* = 1.2, 1H), 7.91-7.89 (m, 2H), 7.67-7.61 (m, 2H), 7.53-7.40 (m, 3H), 3.21 (s, 3H). ^{13}C NMR (75 MHz, $CDCl_3$) δ: 168.59 (C=O), 168.51 (C=O), 147.50 (C), 139.22 (C), 133.26 (C), 132.57 (CH), 130.80 (C), 129.33 (2xCH), 128.97 (CH), 127.47 (2xCH), 123.78 (CH), 121.95 (CH), 24.18 (N-CH_3). HRMS (ESI) [M^+; calculated for $C_{15}H_{11}O_2N$: 237.0784] found m/z 237.0786.

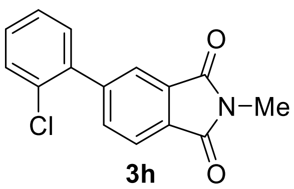

3h

4-(2-chlorophenyl)-*N*-methylphthalimide (3h). Isolated yield: 46%. GC-MS (m/z, $M^{+\cdot}$ 271), major peaks found: 273 (33%), 271 (100%), 245 (5%), 243 (15%), 229 (12%), 227 (36%), 216 (4%), 214 (12%), 188 (4%), 186 (12%), 177 (9%), 152 (15%), 138 (8%), 136 (25%), 75 (11%). ^{1}H NMR (300 MHz, $CDCl_3$) δ: 7.92 (dd, *J* = 1.5, 0.7, 1H), 7.90 (dd, *J* = 7.7, 0.6, 1H), 7.77 (dd, *J* = 7.7, 1.5, 1H), 7.54-7.47 (m, 1H), 7.39-7.32 (m, 3H), 3.20 (s, 3H). ^{13}C NMR (75 MHz, $CDCl_3$) δ: 168.38 (2xC=O), 145.50 (C), 138.62 (C), 135.20 (CH), 132.42 (C), 132.40 (C), 131.31 (C), 131.17 (CH), 130.42 (CH), 129.92 (CH), 127.34 (CH), 124.50 (CH), 123.10 (CH), 24.17 (N-CH_3). HRMS (ESI) [$M+H^+$; calculated for $C_{15}H_{10}O_2NCl$ (^{37}Cl): 274.0447] found m/z 274.0451 and [$M+H^+$; calculated for $C_{15}H_{10}O_2NCl$ (^{35}Cl): 272.0472] found m/z 272.0475.

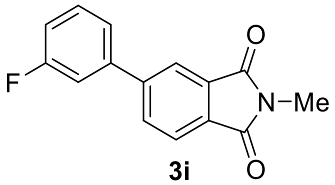

3i

4-(3-fluorophenyl)-*N*-methylphthalimide (3i). Isolated yield: 76%. GC-MS (m/z, $M^{+\cdot}$ 255), major peaks found: 255 (100%), 227 (16%), 211 (46%), 198 (15%), 170 (35%), 120 (30%), 85 (12%). ^{1}H NMR (300 MHz, $CDCl_3$) δ: 8.03-8.01 (m, 1H), 7.92-7.85 (m, 2H), 7.51-7.38 (m, 2H), 7.35-7.28 (m, 1H), 7.13 (tdd, *J* = 8.4, 2.5, 1.4, 1H), 3.20 (s, 3H). ^{13}C NMR (75 MHz, $CDCl_3$) δ: 168.33 (C=O), 168.28 (C=O), 163.36 (d, $J^1_{C\text{-}F}$ = 246.9, C), 146.05 (d, $J^4_{C\text{-}F}$ = 2.3, C), 141.36 (d, $J^3_{C\text{-}F}$ = 7.7, C), 133.31 (C), 132.58 (CH), 131.31 (C), 130.91 (d, $J^3_{C\text{-}F}$ = 8.4, CH), 123.86 (CH), 123.14 (d, $J^4_{C\text{-}F}$ = 2.8, CH), 121.91 (CH), 115.84 (d, $J^2_{C\text{-}F}$ = 21.2, CH), 114.45 (d, $J^2_{C\text{-}F}$ = 22.5, CH), 24.19 (N-CH_3). ^{19}F NMR (282 MHz, $CDCl_3$) δ: -111.52 (td, $J^3_{F\text{-}H}$ = 9.1, $J^4_{F\text{-}H}$ = 6.2, 1F). HRMS (ESI) [$M+H^+$; calculated for $C_{15}H_{10}O_2NF$: 256.0768] found m/z 256.0772.

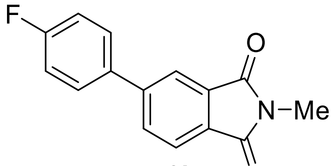

3j

4-(4-fluorophenyl)-*N*-methylphthalimide (3j).[11] Isolated yield: 85%. GC-MS (m/z, $M^{+\cdot}$ 255), major peaks found: 255 (100%), 227 (16%), 211 (46%), 198 (15%), 170 (35%). (The NMR spectrum is consistent with the reported data).^{1}H NMR (300 MHz, $CDCl_3$) δ: 8.02-8.00 (m, 1H), 7.90 (dd, *J* = 7.8, 0.8, 1H), 7.85 (dd, *J* = 7.8, 1.5, 1H), 7.65-7.56 (m, 2H), 7.24-7.14 (m, 2H), 3.21 (s, 3H). ^{13}C NMR (75 MHz, $CDCl_3$) δ: 168.48 (C=O), 168.41 (C=O), 163.43 (d, J^1_{C-F} = 248.9, C), 146.41 (C), 135.35 (d, J^4_{C-F} = 3.6, C), 133.31 (C), 132.38 (CH), 130.78 (C), 129.21 (d, J^3_{C-F} = 8.3, 2xCH), 123.84 (CH), 121.76 (CH), 116.36 (d, J^2_{C-F} = 21.8, 2xCH), 24.20 (N-CH_3). ^{19}F NMR (282 MHz, $CDCl_3$) δ: -112.46 (tt, J^3_{F-H} = 8.9, J^4_{F-H} = 5.1, 1F). HRMS (ESI) [M^+; calculated for $C_{15}H_{10}O_2NF$: 255.0690] found m/z 255.0694.

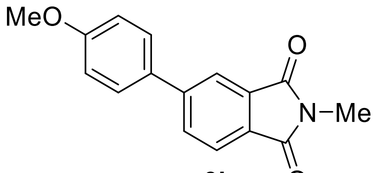

3k

4-(4-methoxyphenyl)-*N*-methylphthalimide (3k).[11] Isolated yield: 81%. GC-MS (m/z, $M^{+\cdot}$ 267), major peaks found: 267 (100%), 224 (18%), 182 (6%), 139 (15%). (The NMR spectrum is consistent with the reported data). ^{1}H NMR (300 MHz, $CDCl_3$) δ: 8.04-8.00 (m, 1H), 7.86 (d, *J* = 1.1, 2H), 7.62-7.56 (m, 2H), 7.06-6.99 (m, 2H), 3.87 (s, 3H), 3.20 (s, 3H). ^{13}C NMR (75 MHz, $CDCl_3$) δ: 168.72 (C=O), 168.60 (C=O), 160.48 (C), 147.07 (C), 133.26 (C), 131.83 (CH), 131.56 (C), 130.05 (C), 128.62 (2xCH), 123.76 (CH), 121.33 (CH), 114.76 (2xCH), 55.57 (O-CH_3), 24.15 (N-CH_3). HRMS (ESI) [M^+; calculated for $C_{16}H_{13}O_3N$: 267.0889] found m/z 267.0889.

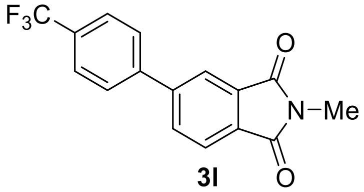

3l

***N*-methyl-4-(4-trifluorophenyl)phthalimide (3l).[11]** Isolated yield: 76%. GC-MS (m/z, $M^{+\cdot}$ 305), major peaks found: 277 (28%), 261 (63%), 248 (23%), 220 (24%), 207 (22%), 170 (25%), 152 (12%). (The NMR spectrum is consistent with the reported data). ^{1}H NMR (300 MHz, $CDCl_3$) δ: 8.08-8.06 (m, 1H), 7.97-7.90 (m, 2H), 7.79-7.72 (m, 4H), 3.22 (s, 3H). ^{13}C NMR (75 MHz, $CDCl_3$) δ: 168.26 (C=O), 168.21 (C=O), 145.88 (C), 142.68 (C), 133.40 (C), 132.85 (CH), 131.65 (C), 131.00 (d, J^2_{C-F} = 32.7, C), 127.86 (2xCH), 126.30 (q, J^4_{C-F} = 3.8, 2xCH), 124.25 (q, J^1_{C-F} = 60.3, CF_3), 123.98 (CH), 122.11 (CH), 24.27 (N-CH_3). ^{19}F NMR (282 MHz, $CDCl_3$) δ: -62.25 (s, 3F). HRMS (ESI) [M^+; calculated for $C_{16}H_{10}O_2NF_3$: 305.0658] found m/z 305.0653.

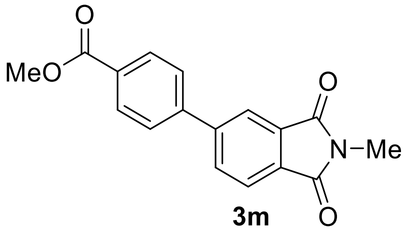

3m

methyl 4-(*N*-methylphthalamidyl)benzoate (3m). Isolated yield: 75%. GC-MS (m/z, $M^{+\cdot}$ 295), major peaks found: 295 (75%), 264 (100%), 251 (5%), 179 (20%), 151 (22%). ^{1}H NMR (300 MHz, $CDCl_3$): 8.17-8.12 (m, 2H), 8.08-8.05 (m, 1H), 7.96-7.88 (m, 2H), 7.72-7.66 (m, 2H), 3.95 (s, 3H), 3.20 (s, 3H). ^{13}C NMR (75 MHz, $CDCl_3$): 168.29 (C=O), 168.25 (C=O), 166.67 (C=O), 146.15 (C), 143.39 (C), 133.29 (C), 132.79 (CH), 131.49 (C), 130.54 (2xCH), 130.46 (C), 127.45 (2xCH), 123.88 (CH), 122.07 (CH), 52.45 (O-CH_3), 24.21 (N-CH_3). HRMS (ESI) [M+H^+; calculated for $C_{17}H_{13}O_4N$: 296.0917] found m/z 296.0915.

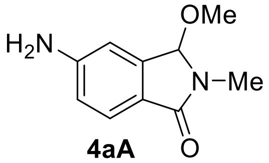

4aA

5-amino-3-methoxy-2-methylisoindolin-1-one (4aA).[11] Isolated yield: 79%. GC-MS (m/z, $M^{+\cdot}$ 192), major peaks found: 192 (35%), 161 (100%), 133 (8%), 120 (5%), 106 (7%). (The NMR spectrum is consistent with the reported data). ^{1}H NMR (300 MHz, $CDCl_3$) δ: 7.56 (d, *J* = 8.0, 1H), 6.80-6.62 (m, 2H), 5.61 (s, 1H), 4.19 (bs, NH_2), 3.01 (s, 3H), 2.89 (s, 3H). ^{13}C NMR (75 MHz, $CDCl_3$): 168.33 (C=O), 150.65 (C), 142.82 (C), 124.77 (CH), 122.98 (C), 115.92 (CH), 108.70 (CH), 87.79 (O-CH-N), 48.99 (O-CH_3), 26.45 (N-CH_3). HRMS (ESI) [M^+; calculated for $C_{10}H_{12}O_2N_2$: 192.0893] found m/z 192.0895.

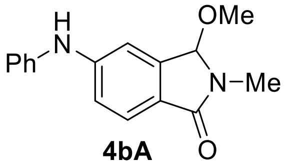

4bA

3-methoxy-2-methyl-5-(phenylamino)isoindolin-1-one (4bA). Isolated yield: 83%. GC-MS (m/z, $M^{+\cdot}$ 268), major peaks found: 268 (40%), 237 (100%), 207 (5%), 167 (10%), 118 (8%). ^{1}H NMR (300 MHz, DMSO-d_6) δ: 8.76 (s, NH), 7.51 (d, *J* = 8.9, 1H), 7.36-7.29 (m, 2H), 7.22-7.16 (m, 2H), 7.12 (dd, *J* = 6.0, 2.2, 2H), 6.98 (t, *J* = 7.3, 1H), 5.76 (s, 1H), 2.90 (s, 3H), 2.88 (s, 3H). ^{13}C NMR (75 MHz, DMSO-d_6): 166.80 (C=O), 147.93 (C), 142.76 (C), 141.54 (C), 129.37 (2xCH), 123.92 (CH), 122.65 (C), 121.76 (CH), 119.12 (2xCH), 116.13 (CH), 108.59 (CH), 87.17 (O-CH-N), 49.10 (O-CH_3), 26.13 (N-CH_3). HRMS (ESI) [M+Na^+; calculated for $C_{16}H_{16}O_2N_2$: 291.1104] found m/z 291.1102.

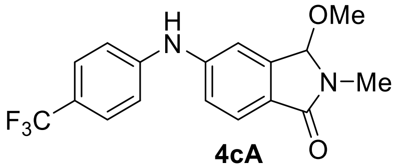

4cA

3-methoxy-2-methyl-5-((4-(trifluoromethyl)phenyl)amino)isoindolin-1-one (4cA). Isolated yield: 87%. GC-MS (m/z, $M^{+\cdot}$ 336), major peaks found: 336 (30%), 317 (5%), 305 (100%), 235 (5%), 152 (8%), 117 (6%). ^{1}H NMR (300 MHz, DMSO-d_6) δ: 9.19 (s, NH), 7.68-7.53 (m, 3H), 7.36-7.20 (m, 4H), 5.80 (s, 1H), 2.92 (s, 3H), 2.91 (s, 3H). ^{13}C NMR (75 MHz, DMSO-d_6) δ: 166.53 (C=O), 145.88 (d, J = 9.4, C), 145.69 (C), 142.72 (C), 126.66 (q, J^3_{C-F} = 3.8, 2xCH), 124.69 (q, J^1_{C-F} = 271.0, CF_3), 124.60 (C), 123.98 (CH), 120.42 (q, J^2_{C-F} = 32.0, C), 118.20 (d, J = 5.0, CH), 116.79 (d, J = 4.9, 2xCH), 110.99 (d, J = 4.9, CH), 87.21 (O-CH-N), 49.31 (O-CH_3), 26.15 (N-CH_3). ^{19}F NMR (282 MHz, DMSO-d_6) δ: -59.55 (s, 3F). HRMS (ESI) [M+H$^+$; calculated for $C_{17}H_{15}O_2N_2F_3$: 337.1158] found m/z 337.1157.

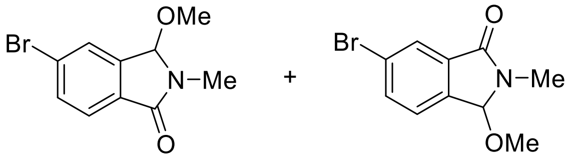

4dA (68%) **4dB** (32%)

5-bromo-3-methoxy-2-methylisoindolin-1-one (4dA) / 6-bromo-3-methoxy-2-methylisoindolin-1-one (4dB). Isolated yield: 89%. GC-MS (m/z, $M^{+\cdot}$ 255) (compound **4dA**), major peaks found: 257 (5%), 255 (5%), 226 (100%), 224 (100%), 171 (6%), 169 (6%), 145 (8%). GC-MS (m/z, $M^{+\cdot}$ 255) (compound **4dB**), major peaks found: 257 (5%), 255 (5%), 226 (100%), 224 (100%), 171 (6%), 169 (6%), 145 (8%). ^{1}H NMR (300 MHz, $CDCl_3$) δ: (compound **4dA**) 7.63-7.59 (m, 3H), 5.67 (s, 1H), 3.00 (s, 3H), 2.87 (s, 3H), (compound **4dB**) 7.87 (d, J = 1.7, 1H), 7.64 (dd, J = 6.8, 1.9, 1H), 7.35 (d, J = 8.0, 1H), 5.67 (s, 1H), 3.01 (s, 3H), 2.84 (s, 3H). ^{13}C NMR (75 MHz, $CDCl_3$) δ: (compound **4dA**) 166.66 (C=O), 142.22 (C), 133.30 (CH), 132.01 (C), 126.73 (CH), 124.98 (C), 124.74 (CH), 87.39 (O-CH-N), 49.47 (O-CH_3), 26.49 (N-CH_3), (compound **4dB**) 166.11 (C=O), 138.93 (C), 135.10 (C), 134.89 (CH), 126.60 (CH), 124.92 (CH), 124.17 (C), 87.62 (O-CH-N), 49.28 (O-CH_3), 26.53 (N-CH_3). HRMS (ESI) (compound **4dA**) [M$^+$; calculated for $C_{10}H_{10}O_2N_2Br$ (^{81}Br): 256.9869] found m/z 256.9871 and [M$^+$; calculated for $C_{10}H_{10}O_2N_2Br$ (^{79}Br): 254.9889], found m/z 254.9886. HRMS (ESI) (compound **4dB**) [M$^+$; calculated for $C_{10}H_{10}O_2N_2Br$ (^{81}Br): 256.9869] found m/z 256.9871 and [M$^+$; calculated for $C_{10}H_{10}O_2N_2Br$ (^{79}Br): 254.9889] found m/z 254.9886.

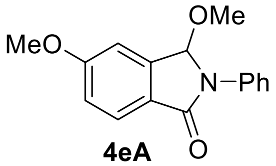

4eA

3,5-dimethoxy-2-methylisoindolin-1-one (4eA). Isolated yield: 70%. GC-MS (m/z, $M^{+\cdot}$ 269), major peaks found: 269 (35%), 254 (100%), 238 (85%), 195 (5%), 167 (8%), 77 (15%). ^{1}H NMR (300 MHz, $CDCl_3$) δ: 7.85-7.79 (m, 3H), 7.46-7.40 (m, 2H), 7.24-7.19 (m, 1H), 7.11-7.06 (m, 2H), 6.40 (s, 1H), 3.92 (s, 3H), 2.93 (s, 3H). ^{13}C NMR (101 MHz, $CDCl_3$) δ: 166.74 (C=O), 163.98 (C), 142.25 (C), 137.70 (C), 129.18 (2xCH), 125.53 (CH), 125.42 (C),

125.12 (CH), 121.55 (2xCH), 116.92 (CH), 108.17 (CH), 86.94 (O-CH-N), 55.94 (O-CH_3), 49.09 (O-CH_3). HRMS (ESI) [M+H$^+$; calculated for $C_{16}H_{15}O_3N$: 270.1124] found m/z 270.1125.

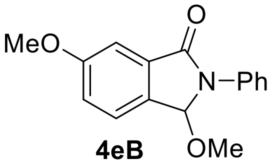

4eB

3,6-dimethoxy-2-phenylisoindolin-1-one (4eB). Isolated yield: 29%. GC-MS (m/z, M$^{+\cdot}$ 269), major peaks found: 269 (18%), 254 (8%), 238 (100%), 223 (4%), 167 (6%), 77 (8%). ^{1}H NMR (400 MHz, $CDCl_3$) δ: 7.83-7.79 (m, 2H), 7.51-7.39 (m, 4H), 7.26-7.17 (m, 2H), 6.43 (s, 1H), 3.91 (s, 3H), 2.91 (s, 3H). ^{13}C NMR (101 MHz, $CDCl_3$) δ: 166.78 (C=O), 161.77 (C), 137.48 (C), 134.66 (C), 131.83 (C), 129.22 (2xCH), 125.42 (CH), 124.57 (CH), 121.82 (2xCH), 120.86 (CH), 106.96 (CH), 87.18 (O-CH-N), 55.93 (O-CH_3), 49.02 (O-CH_3). HRMS (ESI) [M+H$^+$; calculated for $C_{16}H_{15}O_3N$: 270.1124] found m/z 270.1124.

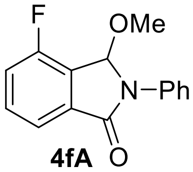

4fA

4-fluoro-3-methoxy-2-phenylisoindolin-1-one (4fA). Isolated yield: 6%. GC-MS (m/z, M$^{+\cdot}$ 257), major peaks found: 257 (40%), 242 (35%), 226 (100%), 207 (5%), 197 (10%), 170 (8%), 148 (7%), 107 (7%), 94 (4%), 77 (28%). ^{1}H NMR (300 MHz, $CDCl_3$) δ: 7.84-7.70 (m, 3H), 7.60 (td, J = 7.8, 4.5, 1H), 7.50-7.42 (m, 2H), 7.36-7.29 (m, 1H), 7.29-7.23 (m, 1H), 6.61 (s, 1H), 3.05 (s, 3H). ^{13}C NMR (75 MHz, $CDCl_3$) δ: 165.54 (C=O), 158.02 (d, $J^1_{C\text{-}F}$ = 254.7, C), 144.72 (d, $J^2_{C\text{-}F}$ = 6.0, C), 136.97 (C), 135.82 (d, $J^3_{C\text{-}F}$ = 2.7, C), 132.90 (d, $J^3_{C\text{-}F}$ = 6.5, CH), 129.34 (2xCH), 125.92 (CH), 122.19 (2xCH), 120.15 (d, $J^4_{C\text{-}F}$ = 4.2, CH), 119.98 (d, $J^2_{C\text{-}F}$ = 20.3, CH), 85.92 (O-CH-N), 50.44 (O-CH_3). ^{19}F NMR (282 MHz, $CDCl_3$) δ: -118.14 (dd, $J^3_{F\text{-}H}$ = 8.5, $J^4_{F\text{-}H}$ = 4.4, 1F). HRMS (ESI) [M+Na$^+$; calculated for $C_{15}H_{12}O_2NF$: 280.0744] found m/z 280.0748.

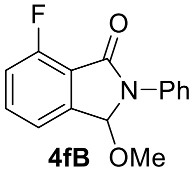

4fB

7-fluoro-3-methoxy-2-phenylisoindolin-1-one (4fB). Isolated yield: 80%. GC-MS (m/z, M$^{+\cdot}$ 257), major peaks found: 257 (40%), 242 (55%), 226 (100%), 207 (20%), 197 (22%), 170 (7%), 148 (7%), 94 (9%), 77 (20%). ^{1}H NMR (300 MHz, $CDCl_3$) δ: 7.73-7.67 (m, 2H), 7.56 (ddd, J = 8.2, 7.5, 4.6, 1H), 7.40-7.30 (m, 3H), 7.19-7.10 (m, 2H), 6.36 (s, 1H), 2.86 (s, 3H). ^{13}C NMR (75 MHz, $CDCl_3$) δ: 163.55 (d, $J^3_{C\text{-}F}$ = 2.4, C=O), 159.02 (d, $J^1_{C\text{-}F}$ = 263.0, C), 142.54 (d, $J^3_{C\text{-}F}$ = 1.8, C), 137.00 (C), 135.00 (d, $J^3_{C\text{-}F}$ = 7.6, CH), 129.23 (2xCH), 125.65 (CH), 121.92 (2xCH), 119.90 (d, $J^2_{C\text{-}F}$ = 12.4, C), 119.63 (d, $J^4_{C\text{-}F}$ = 4.3, CH), 117.96 (d, $J^2_{C\text{-}F}$ = 19.4, CH), 86.92 (d, $J^4_{C\text{-}F}$ = 1.2, O-CH-N), 49.36 (O-CH_3). ^{19}F NMR (282 MHz, $CDCl_3$) δ: -116.21 (dd, $J^3_{F\text{-}H}$ = 9.1, $J^4_{F\text{-}H}$ = 4.5, 1F). HRMS (ESI) [M+H$^+$; calculated for $C_{15}H_{12}O_2NF$: 258.0924] found m/z 258.0924.

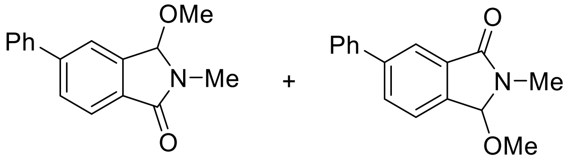

4gA (70%) **4gB** (30%)

5-phenyl-3-methoxy-2-methylisoindolin-1-one (4gA) / 6-phenyl-3-methoxy-2-methylisoindolin-1-one (4gB).[11] Isolated yield: 96%. GC-MS (m/z, $M^{+\cdot}$ 253) (compound **4gA**), major peaks found: 253 (10%), 222 (100%), 165 (9%), 152 (10%). GC-MS (m/z, $M^{+\cdot}$ 253) (compound **4gB**), major peaks found: 253 (10%), 222 (100%), 165 (9%), 152 (10%). (The NMR spectrum is consistent with the reported data). ^{1}H NMR (300 MHz, $CDCl_3$): (compound **4gA**) 7.86 (d, *J* = 8.3, 1H), 7.72 (dt, *J* = 4.8, 1.5, 2H), 7.64-7.55 (m, 2H), 7.50-7.34 (m, 3H), 5.78 (s, 1H), 3.09 (s, 3H), 2.93 (s, 3H), (compound **4gB**) 8.04 (d, *J* = 1.2, 1H), 7.78 (dd, *J* = 7.8, 1.7, 1H), 7.64-7.55 (m, 3H), 7.50-7.34 (m, 3H), 5.78 (s, 1H), 3.09 (s, 3H), 2.93 (s, 3H). ^{13}C NMR (75 MHz, $CDCl_3$) δ: (compound **4gA** and **4gB**) 167.69 (C=O, **4gB**), 167.64 (C=O, **4gA**), 145.30 (C, **4gA**), 143.37 (C, **4gB**), 141.11 (C, **4gA**), 140.04 (C, **4gA**), 139.90 (C, **4gB**), 139.04 (C, **4gB**), 133.94 (C, **4gB**), 132.03 (C, **4gA**), 130.89 (CH), 129.06 (CH), 129.04 (CH), 128.28 (CH), 128.05 (CH), 127.43 (CH), 127.29 (CH), 123.75 (CH), 121.99 (CH), 121.87 (CH), 88.03 (O-CH-N, **4gA**), 87.95 (O-CH-N, **4gB**), 49.30 (O-CH_3, **4gA**), 49.27 (O-CH_3, **4gB**), 26.55 (N-CH_3). HRMS (ESI) (compound **4gA**) [M^+; calculated for $C_{16}H_{15}O_2N$: 253.1097] found m/z 253.1095. HRMS (ESI) (compound **4gB**) [M^+; calculated for $C_{16}H_{15}O_2N$: 253.1097] found m/z 253.1095.

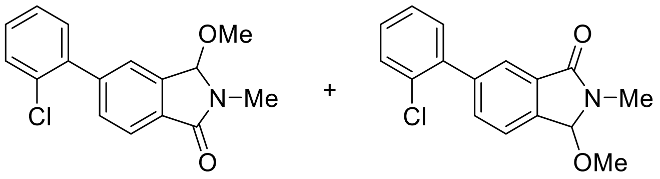

4hA (73%) **4hB** (27%)

5-(2-chlorophenyl)-3-methoxy-2-methylisoindolin-1-one (4hA) / 6-(2-chlorophenyl)-3-methoxy-2-methylisoindolin-1-one (4hB). Isolated yield: 82%. GC-MS (m/z, $M^{+\cdot}$ 287) (compound **4hA**), major peaks found: 287 (5%), 258 (33%), 256 (100%), 165 (10%), 152 (8%), 128 (9%). GC-MS (m/z, $M^{+\cdot}$ 287) (compound **4hB**), major peaks found: 287 (5%), 258 (33%), 256 (100%), 165 (10%), 152 (8%), 128 (9%). ^{1}H NMR (300 MHz, $CDCl_3$) δ: (compound **4hA**) 7.92-7.83 (m, 1H), 7.62 (d, *J* = 0.7, 1H), 7.60-7.54 (m, 1H), 7.52-7.46 (m, 1H), 7.39-7.27 (m, 3H), 5.81 (s, 1H), 3.10 (s, 3H), 2.96 (s, 3H), (compound **4hB**) 7.92-7.83 (m, 1H), 7.66 (dd, *J* = 7.7, 1.6, 1H), 7.60-7.54 (m, 1H), 7.52-7.46 (m, 1H), 7.39-7.27 (m, 3H), 5.81 (s, 1H), 3.10 (s, 3H), 2.97 (s, 3H). ^{13}C NMR (75 MHz, $CDCl_3$) δ: (compound **4hA** and **4hB**) 167.57 (C=O, **4hB**), 167.55 (C=O, **4hA**), 143.33, 141.47, 140.23, 139.54, 139.49, 139.35, 133.39, 133.35, 132.47, 132.45, 131.42, 131.39, 131.31, 130.27 (CH, **4hA**), 130.21 (CH, **4hB**), 129.40 (CH, **4hA**), 129.28 (CH, **4hB**), 127.15 (CH, **4hA**), 124.69 (CH, **4hA**), 124.44 (CH, **4hB**), 123.22 (CH, **4hA**), 123.09 (CH, **4hB**), 88.09 (O-CH-N), 49.50 (O-CH_3, **4hB**), 49.44 (O-CH_3, **4hA**), 26.63 (N-CH_3). HRMS (ESI) (compound **4hA**) [$M+H^+$; calculated for $C_{16}H_{14}O_2NCl$ (^{37}Cl): 290.0760] found m/z 290.0760 and $M+H^+$; calculated for $C_{16}H_{14}O_2NCl$ (^{35}Cl): 288.0785] found m/z 288.0785. HRMS (ESI) (compound **4hB**) [$M+H^+$; calculated for $C_{16}H_{14}O_2NCl$ (^{37}Cl): 290.0760] found m/z 290.0760 and $M+H^+$; calculated for $C_{16}H_{14}O_2NCl$ (^{35}Cl): 288.0785] found m/z 288.0785.

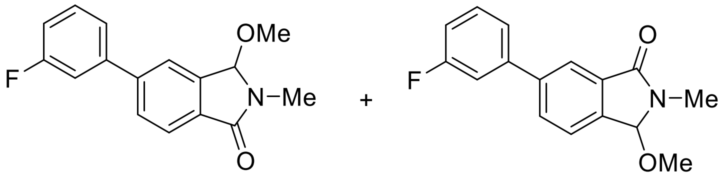

4iA (69%) **4iB** (31%)

5-(3-fluorophenyl)-3-methoxy-2-methylisoindolin-1-one (4iA) / 6-(3-fluorophenyl)-3-methoxy-2-methylisoindolin-1-one (4iB). Isolated yield: 95%. GC-MS (m/z, $M^{+\cdot}$ 271) (compound **4iA**), major peaks found: 271 (10%), 240 (100%), 183 (8%), 170 (8%). GC-MS (m/z, $M^{+\cdot}$ 271) (compound **4iB**), major peaks found: 271 (10%), 240 (100%), 183 (8%), 170 (8%). ^{1}H NMR (300 MHz, $CDCl_3$) δ: (compound **4iA**) 7.86 (d, *J* = 8.3, 1H), 7.72-7.67 (m, 2H), 7.47-7.35 (m, 2H), 7.34-7.27 (m, 1H), 7.12-7.02 (m, 1H), 5.79 (s, 1H), 3.08 (s, 3H), 2.94 (s, 3H), (compound **4iB**) 8.00 (d, *J* = 1.1, 1H), 7.76 (dd, *J* = 7.8, 1.7, 1H), 7.58 (d, *J* = 7.8, 1H), 7.47 – 7.35 (m, 2H), 7.34-7.27 (m, 1H), 7.12-7.02 (m, 1H), 5.79 (s, 1H), 3.09 (s, 3H), 2.93 (s, 3H). ^{13}C NMR (75 MHz, $CDCl_3$) δ: (compound **4iA**) 167.46 (C=O), 163.27 (d, $J^{1}_{C\text{-}F}$ = 246.5, C), 143.98 (d, $J^{4}_{C\text{-}F}$ = 2.3, C), 142.17 (d, $J^{3}_{C\text{-}F}$ = 10.1, C), 141.25 (C), 132.57 (C), 130.63 (d, $J^{3}_{C\text{-}F}$ = 8.4, CH), 129.09 (CH), 123.91 (CH), 123.13 (d, $J^{4}_{C\text{-}F}$ = 2.8, CH), 122.03 (CH), 115.14 (d, $J^{2}_{C\text{-}F}$ = 21.2, CH), 114.41 (d, $J^{2}_{C\text{-}F}$ = 22.4, CH), 88.03 (O-CH-N), 49.42 (O-CH_3), 26.61 (N-CH_3), (compound **4iB**) 167.51 (C=O), 163.27 (d, $J^{1}_{C\text{-}F}$ = 246.5, C), 142.34 (C), 142.15 (d, $J^{3}_{C\text{-}F}$ = 9.6, C), 139.65 (C), 134.08 (C), 130.88 (CH), 130.60 (d, $J^{3}_{C\text{-}F}$ = 8.4, CH), 123.93 (CH), 122.98 (d, $J^{4}_{C\text{-}F}$ = 2.9, CH), 121.92 (CH), 114.93 (d, $J^{2}_{C\text{-}F}$ = 21.2, CH), 114.26 (d, $J^{2}_{C\text{-}F}$ = 22.3, CH), 87.96 (O-CH-N), 49.37 (O-CH_3), 26.61 (N-CH_3). ^{19}F NMR (282 MHz, $CDCl_3$) δ: (compound **4iA**) -111.93-(-112.08) (m, 1F), (compound **4iB**) -112.03-(-112.18) (m, 1F). HRMS (ESI) (compound **4iA**) [M+H^{+}; calculated for $C_{16}H_{14}O_2NF$: 272.1081] found m/z 272.1084. HRMS (ESI) (compound **4iB**) [M+H^{+}; calculated for $C_{16}H_{14}O_2NF$: 272.1081] found m/z 272.1084.

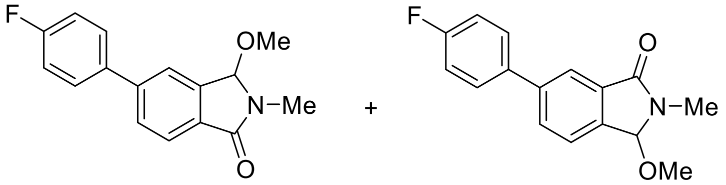

4jA (68%) **4jB** (32%)

5-(4-fluorophenyl)-3-methoxy-2-methylisoindolin-1-one (4jA) / 6-(4-fluorophenyl-3-methoxy-2-methylisoindolin-1-one (4jB).[11] Isolated yield: 96%. GC-MS (m/z, $M^{+\cdot}$ 271) (compound **4jA**), major peaks found: 271 (10%), 240 (100%), 183 (8%), 170 (8%). GC-MS (m/z, $M^{+\cdot}$ 271) (compound **4jB**), major peaks found: 271 (10%), 240 (100%), 183 (8%), 170 (8%). (The NMR spectrum is consistent with the reported data). ^{1}H NMR (300 MHz, $CDCl_3$) δ: (compound **4jA**) 7.85 (d, *J* = 8.3, 1H), 7.69-7.64 (m, 2H), 7.61-7.53 (m, 2H), 7.19-7.09 (m, 2H), 5.78 (s, 1H), 3.08 (s, 3H), 2.93 (s, 3H), (compound **4jB**) 7.97 (d, *J* = 1.3, 1H), 7.73 (dd, *J* = 7.7, 2.2, 1H), 7.61-7.53 (m, 3H), 7.19-7.09 (m, 2H), 5.78 (s, 1H), 3.09 (s, 3H), 2.93 (s, 3H). ^{13}C NMR (75 MHz, $CDCl_3$) δ: (compound **4jA** and **4jB**) 167.60 (C=O, **4jB**), 167.56 (C=O, **4jA**), 163.03 (d, $J^{1}_{C\text{-}F}$ = 248.1, C, **4jA**), 162.90 (d, $J^{1}_{C\text{-}F}$ = 247.7, C, **4jB**), 144.29 (C, **4jA**), 142.38 (C, **4jB**), 141.22 (C, **4jA**), 139.06 (C, **4jB**), 136.20 (d, $J^{4}_{C\text{-}F}$ = 3.3, C, **4jA**), 136.07 (d, $J^{4}_{C\text{-}F}$ = 3.1, C, **4jB**), 134.03 (C, **4jB**), 132.05 (C, **4jA**), 130.73 (CH, **4jA**), 129.12 (d, $J^{3}_{C\text{-}F}$ = 8.5, 2xCH, **4jA**), 129.01 (CH), 128.92 (CH), 123.84 (CH), 121.86 (CH, **4jA**), 121.74 (CH, **4jB**), 116.04 (d, $J^{2}_{C\text{-}F}$ = 21.6, 2xCH, **4jA**), 115.99 (d, $J^{2}_{C\text{-}F}$ = 21.5, 2xCH, **4jB**), 88.03 (O-CH-N, **4jA**), 87.96 (O-CH-N, **4jB**), 49.36 (O-CH_3, **4jA**), 49.31 (O-CH_3, **4jB**), 26.60 (N-CH_3). ^{19}F NMR (282 MHz, $CDCl_3$) δ: (compound **4jA**) -113.59 (tt, $J^{3}_{F\text{-}H}$ = 8.9, $J^{4}_{F\text{-}H}$ = 5.2, 1F), (compound **4jB**) -114.03 (tt, $J^{3}_{F\text{-}H}$ = 8.7, $J^{4}_{F\text{-}H}$ = 5.2, 1F). HRMS (ESI) (compound **4jA**) [M^{+}; calculated for $C_{16}H_{14}O_2NF$: 271.1003],

found m/z 271.1000. HRMS (ESI) (compound **4jB**) [M^+; calculated for $C_{16}H_{14}O_2NF$: 271.1003], found m/z 271.1000.

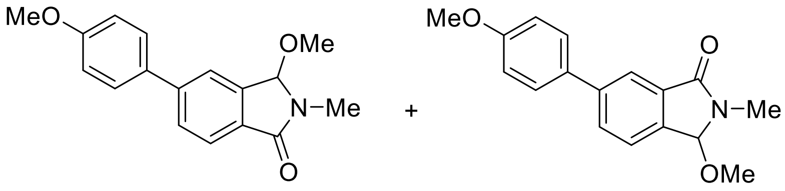

4kA (81%) **4kB** (19%)

5-(4-methoxy)phenyl-3-methoxy-2-methylisoindolin-1-one (4kA) / 6-(4-methoxy)phenyl-3-methoxy-2-methylisoindolin-1-one (4kB).[11] Isolated yield: 83%. GC-MS (m/z, $M^{+\cdot}$ 283) (compound **4kA**), major peaks found: 283 (30%), 252 (100%), 209 (9%). GC-MS (m/z, $M^{+\cdot}$ 283) (compound **4kB**), major peaks found: 283 (30%), 252 (100%), 209 (9%). (The NMR spectrum is consistent with the reported data). ^{1}H NMR (300 MHz, $CDCl_3$) δ: (compound **4kA**) 7.84 (d, *J* = 8.3, 1H), 7.69 (dd, *J* = 5.9, 1.8, 2H), 7.59-7.51 (m, 2H), 7.03-6.95 (m, 2H), 5.78 (s, 1H), 3.85 (s, 3H), 3.09 (s, 3H), 2.93 (s, 3H), (compound **4kB**) 7.99 (d, *J* = 1.6, 1H), 7.75 (dd, *J* = 7.9, 1.7, 1H), 7.59-7.51 (m, 3H), 7.03-6.95 (m, 2H), 5.78 (s, 1H), 3.85 (s, 3H), 3.09 (s, 3H), 2.93 (s, 3H). ^{13}C NMR (75 MHz, $CDCl_3$) δ: (compound **4kA** and **4kB**) 167.85 (C=O, **4kB**), 167.79 (C=O, **4kA**), 159.97 (C, **4kA**), 159.77 (C, **4kB**), 144.94 (C, **4kA**), 143.00 (C, **4kB**), 141.14 (C, **4kA**), 138.39 (C, **4kB**), 133.94 (C, **4kB**), 132.48 (C, **4kA**), 132.40 (C, **4kB**), 131.43 (C, **4kA**), 130.41 (CH), 128.56 (CH), 128.51 (CH), 128.39 (CH), 123.75 (CH), 121.45 (CH), 121.36 (CH), 114.54 (CH), 114.51 (CH), 88.08 (O-CH-N, **4kA**), 88.00 (O-CH-N, **4kB**), 55.49 (O-CH_3, **4kA**), 55.46 (O-CH_3, **4kB**), 49.30 (O-CH_3, **4kA**), 49.26 (O-CH_3, **4kB**), 26.59 (N-CH_3). HRMS (ESI) (compound **4kA**) [M^+; calculated for $C_{17}H_{17}O_3N$: 283.1202], found m/z 283.1201. HRMS (ESI) (compound **4kB**) [M^+; calculated for $C_{17}H_{17}O_3N$: 283.1202], found m/z 283.1201.

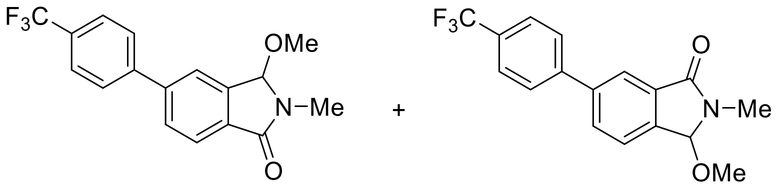

4lA (69%) **4lB** (31%)

5-(4-trifluoromethyl)phenyl-3-methoxy-2-methylisoindolin-1-one (4lA) / 6-(4-trifluoromethyl)phenyl-3-methoxy-2-methylisoindolin-1-one (4lB).[11] Isolated yield: 88%. GC-MS (m/z, $M^{+\cdot}$ 321) (compound **4lA**), major peaks found: 321 (6%), 290 (100%), 165 (6%). GC-MS (m/z, $M^{+\cdot}$ 321) (compound **4lB**), major peaks found: 321 (6%), 290 (100%), 165 (6%). (The NMR spectrum is consistent with the reported data). ^{1}H NMR (300 MHz, $CDCl_3$) δ: (compound **4lA**) 7.90 (d, *J* = 8.3, 1H), 7.76-7.70 (m, 6H), 5.82 (s, 1H), 3.10 (s, 3H), 2.95 (s, 3H), (compound **4lB**) 8.04 (d, *J* = 1.2, 1H), 7.80 (dd, *J* = 7.8, 1.7, 1H), 7.76-7.70 (m, 4H), 7.62 (d, *J* = 7.9, 1H), 5.82 (s, 1H), 3.10 (s, 3H), 2.95 (s, 3H). ^{13}C NMR (75 MHz, $CDCl_3$) δ: (compound **4lA** and **4lB**) 167.43 (C=O, **4lB**), 167.38 (C=O, **4lA**), 143.81, 143.64, 143.62, 143.47, 141.95, 141.38, 140.05, 134.25, 132.97, 132.04, 131.07, 130.59, 130.40, 130.15, 130.04, 129.96, 129.34, 127.86 (CH, **4lA**), 127.69 (CH, **4lB**), 126.06 (q, $J^4_{C\text{-}F}$ = 3.6, 2xCH), 124.08, 124.06, 122.26, 122.14, 88.06 (O-CH-N, **4lA**), 87.99 (O-CH-N, **4lB**), 49.48 (O-CH_3, **4lA**), 49.45 (O-CH_3, **4lB**), 26.67 (N-CH_3). ^{19}F NMR (282 MHz, $CDCl_3$) δ: (compound **4lA**) -62.14 (s, 3F), (compound **4lB**) -62.12 (s, 3F). HRMS (ESI) (compound

2lA) [M^+; calculated for $C_{17}H_{14}O_2NF_3$: 321.0971] found m/z 321.0971. HRMS (ESI) (compound **2lB**) [M^+; calculated for $C_{17}H_{14}O_2NF_3$: 321.0971] found m/z 321.0971.

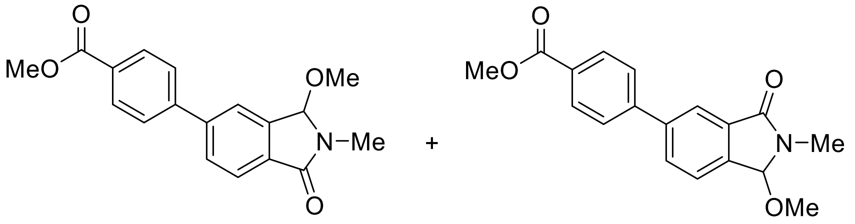

4mA (69%) **4mB** (31%)

methyl 4-(1-methoxy-2-methyl-3-oxoisoindolin-5-yl)benzoate (4mA) / methyl 4-(3-methoxy-2-methyl-1-oxoisoindolin-5-yl)benzoate (4mB). Isolated yield: 85%. GC-MS (m/z, $M^{+\cdot}$ 311) (compound **4mA**), major peaks found: 311 (10%), 280 (100%), 264 (5%), 221 (8%), 207 (10%), 165 (10%), 124 (12%). GC-MS (m/z, $M^{+\cdot}$ 311) (compound **4mA**), major peaks found: 311 (10%), 280 (100%), 264 (8%), 221 (10%), 207 (18%), 165 (10%), 125 (12%). ^{1}H NMR (300 MHz, $CDCl_3$) δ: (compound **4mA**) 8.14-8.08 (m, 2H), 7.88 (d, *J* = 8.3, 1H), 7.77-7.73 (m, 2H), 7.70-7.64 (m, 2H), 5.80 (s, 1H), 3.93 (s, 3H), 3.09 (s, 3H), 2.94 (s, 3H), (compound **4mB**) 8.14-8.08 (m, 2H), 8.05 (d, *J* = 1.2, 1H), 7.82 (dd, *J* = 7.8, 1.7, 1H), 7.70-7.64 (m, 2H), 7.60 (d, *J* = 7.8, 1H), 5.80 (s, 1H), 3.93 (s, 3H), 3.09 (s, 3H), 2.93 (s, 3H). ^{13}C NMR (75 MHz, $CDCl_3$) δ: (compound **4mA**) 167.41 (C=O), 166.78 (C=O), 144.39 (C), 144.06 (C), 141.27 (C), 132.83 (C), 130.35 (2xCH), 129.86 (C), 129.29 (CH), 127.44 (2xCH), 123.94 (CH), 122.20 (CH), 88.03 (O-CH-N), 52.34 (O-CH_3), 49.44 (O-CH_3), 26.64 (N-CH_3), (compound **4mB**) 167.44 (C=O), 166.83 (C=O), 144.26 (C), 142.19 (C), 139.92 (C), 134.13 (C), 131.05 (CH), 130.35 (2xCH), 129.68 (C), 127.28 (2xCH), 123.97 (CH), 122.10 (CH), 87.95 (O-CH-N), 52.30 (O-CH_3), 49.38 (O-CH_3), 26.64 (N-CH_3). HRMS (ESI) (compound **4mA**) [$M+H^+$; calculated for $C_{18}H_{17}O_4N$: 312.1230], found m/z 312.1233. HRMS (ESI) (compound **4mB**) [$M+H^+$; calculated for $C_{18}H_{17}O_4N$: 312.1230], found m/z 312.1233.

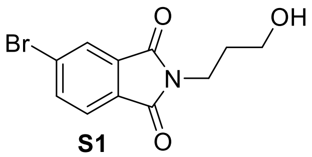

S1

4-bromo-*N*-(3-hydroxypropyl)phthalimide (S1). Isolated yield: 81%. GC-MS (m/z, $M^{+\cdot}$ 285), major peaks found: 285 (20%), 283 (20%), 267 (23%), 265 (23%), 255 (18%), 253 (18%), 240 (100%), 238 (100%), 229 (15%), 227 (15%), 210 (18%), 208 (16%), 184 (18%), 182 (18%), 156 (15%), 154 (15%), 132 (8%), 103 (13%), 75 (35%). ^{1}H NMR (300 MHz, $CDCl_3$) δ: 7.97 (dd, *J* = 1.7, 0.4, 1H), 7.86 (dd, *J* = 7.9, 1.7, 1H), 7.70 (dd, *J* = 7.9, 0.4, 1H), 3.84 (t, *J* = 6.3, 2H), 3.61 (t, *J* = 5.8, 2H), 2.27 (bs, OH), 1.94-1.81 (m, 2H). ^{13}C NMR (75 MHz, $CDCl_3$) δ: 168.15 (C=O), 167.61 (C=O), 137.21 (CH), 133.75 (C), 130.62 (C), 129.17 (C), 126.87 (CH), 124.83 (CH), 59.24 (O-CH_2), 34.73 (N-CH_2), 31.34 (CH_2). HRMS (ESI) [$M+H^+$; calculated for $C_{11}H_{10}O_3NBr$ (^{81}Br): 285.9897] found m/z 285.9898 and [$M+H^+$; calculated for $C_{11}H_{10}O_3NBr$ (^{79}Br): 283.9916] found m/z 283.9920.

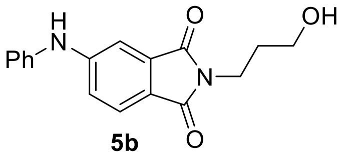

5b

***N*-(3-hydroxypropyl)-4-phenylaminophthalimide (5b).** Isolated yield: 88%. GC-MS (m/z, $M^{+\cdot}$ 296), major peaks found: 296 (40%), 281 (36%), 251 (38%), 207 (100%), 195 (15%), 167 (17%), 147 (7%), 133 (9%), 96 (7%), 73 (15%). ^{1}H NMR (300 MHz, $CDCl_3$) δ: 7.65 (d, *J* = 8.2, 1H), 7.42-7.34 (m, 3H), 7.24-7.09 (m, 4H), 6.36 (s, NH), 3.81 (t, *J* = 6.1, 2H), 3.60 (q, *J* = 5.8, 2H), 2.69 (t, *J* = 6.3, OH), 1.84 (quint, *J* = 6.0, 2H). ^{13}C NMR (75 MHz, $CDCl_3$) δ: 169.24 (C=O), 169.08 (C=O), 150.35 (C), 139.82 (C), 134.85 (C), 129.94 (2xCH), 125.35 (CH), 124.67 (CH), 121.84 (2xCH), 121.52 (C), 118.77 (CH), 109.07 (CH), 59.02 (O-CH_2), 34.01 (N-CH_2), 31.54 (CH_2). HRMS (ESI) [$M+H^+$; calculated for $C_{17}H_{16}O_3N_2$: 297.1233] found m/z 297.1236.

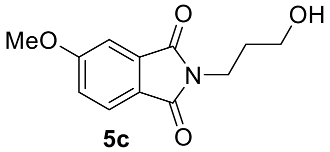

5c

***N*-(3-hydroxypropyl)-4-methoxyphthalimide (5c).** Isolated yield: 76%. GC-MS (m/z, $M^{+\cdot}$ 235), major peaks found: 235 (35%), 217 (10%), 205 (8%), 190 (100%), 178 (6%), 160 (10%), 135 (15%), 106 (8%). ^{1}H NMR (400 MHz, $CDCl_3$) δ: 7.74 (d, *J* = 8.3, 1H), 7.32 (d, *J* = 2.3, 1H), 7.15 (dd, *J* = 8.3, 2.3, 1H), 3.92 (s, 3H), 3.81 (t, *J* = 6.3, 2H), 3.60 (t, *J* = 5.8, 2H), 2.40 (bs, OH), 1.86 (quint, *J* = 6.0, 2H). ^{13}C NMR (101 MHz, $CDCl_3$) δ: 168.87 (C=O), 164.91 (C=O), 134.72 (C), 125.20 (CH), 123.92 (C), 119.85 (CH), 108.40 (CH), 59.09 (O-CH_2), 56.24 (O-CH_3), 34.25 (N-CH_2), 31.49 (CH_2). HRMS (ESI) [$M+H^+$; calculated for $C_{12}H_{13}O_4N$: 236.0917] found m/z 236.0920.

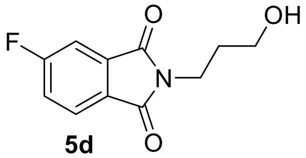

5d

4-fluoro-*N*-(3-hydroxypropyl)phthalimide (5d). Isolated yield: 70%. GC-MS (m/z, $M^{+\cdot}$ 223), major peaks found: 223 (10%), 205 (20%), 193 (18%), 178 (100%), 167 (12%), 148 (22%), 122 (20%), 94 (21%). ^{1}H NMR (300 MHz, $CDCl_3$) δ: 7.83 (dd, *J* = 8.2, 4.5, 1H), 7.49 (dd, *J* = 7.0, 2.3, 1H), 7.37 (ddd, *J* = 8.8, 8.3, 2.3, 1H), 3.82 (t, *J* = 6.3, 2H), 3.61 (t, *J* = 5.9, 2H), 3.32 (bs, OH), 1.86 (quint, *J* = 6.1, 2H). ^{13}C NMR (75 MHz, $CDCl_3$) δ: 168.21 (C=O), 167.52 (d, $J^4_{C\text{-}F}$ = 2.7, C=O), 166.35 (d, $J^1_{C\text{-}F}$ = 232.8, C-F), 134.90 (d, $J^3_{C\text{-}F}$ = 9.4, C), 127.83 (d, $J^4_{C\text{-}F}$ = 3.0, C), 125.80 (d, $J^3_{C\text{-}F}$ = 9.3, CH), 121.14 (d, $J^2_{C\text{-}F}$ = 23.7, CH), 111.34 (d, $J^2_{C\text{-}F}$ = 24.9, CH), 59.25 (O-CH_2), 34.75 (N-CH_2), 31.31 (CH_2). ^{19}F NMR (282 MHz, $CDCl_3$) δ: -101.16-(-101.35) (m, 1F). HRMS (ESI) [$M+H^+$; calculated for $C_{11}H_{10}O_3NF$: 224.0717] found m/z 224.0719.

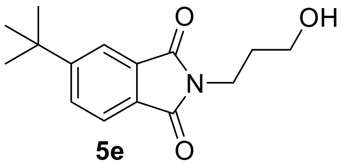

5e

***N*-(3-hydroxypropyl)-4-tert-butylphthalimide (5e).** Isolated yield: 98%. GC-MS (m/z, $M^{+\cdot}$ 261), major peaks found: 261 (45%), 243 (15%), 228 (17%), 216 (100%), 202 (20%), 186 (18%), 174 (16%), 160 (14%), 145 (18%), 115 (18%), 91 (9%). ^{1}H NMR (300 MHz, $CDCl_3$) δ: 7.86 (dd, *J* = 1.5, 0.9, 1H), 7.76-7.68 (m, 2H), 3.81 (t, *J* = 6.4, 2H), 3.59 (t, *J* = 5.8, 2H), 2.64 (bs, OH), 1.85 (quint, *J* = 6.0, 2H), 1.35 (s, 9H). ^{13}C NMR (75 MHz, $CDCl_3$) δ: 169.40 (C=O), 169.01 (C=O), 158.81 (C), 132.26 (C), 131.10 (CH), 129.34 (C), 123.24 (CH), 120.64 (CH), 59.09 (O-CH_2), 35.82 (C), 34.24 (N-CH_2), 31.45 (CH_2), 31.20 (3xCH_3). HRMS (ESI) [M+H^+; calculated for $C_{15}H_{19}O_3N$: 262.1437] found m/z 262.1437.

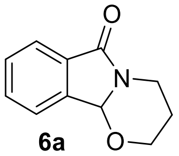

6a

3,4-dihydro-2H-[1,3]oxazino[2,3-a]isoindol-6(10bH)-one (6a).[11] Isolated yield: 94%. GC-MS (m/z, $M^{+\cdot}$ 189), major peaks found: 189 (80%), 188 (100%), 160 (80%), 146 (10%), 130 (65%), 104 (55%), 77 (48%). (The NMR spectrum is consistent with the reported data). ^{1}H NMR (400 MHz, $CDCl_3$): 7.79 (d, *J* = 7.2 Hz, 1H), 7.59-7.40 (m, 3H), 5.52 (s, 1H), 4.44 (dd, *J* = 13.4, 5.4, 1H), 4.27-4.14 (m, 1H), 3.91 (td, *J* = 12.3, 2.1, 1H), 3.22 (td, *J* = 12.9, 3.9, 1H), 1.94-1.72 (m, 1H), 1.60 (ddd, *J* = 13.6, 3.7, 1.9, 1H). ^{13}C NMR (101 MHz, $CDCl_3$): 165.94 (C=O), 141.23 (C), 132.81 (C), 131.92 (CH), 130.01 (CH), 123.65 (CH), 123.17 (CH), 85.16 (O-CH-N), 67.38 (O-CH_2), 37.89 (N-CH_2), 24.67 (CH_2). HRMS (ESI) [M+Na^+; calculated for $C_{11}H_{11}O_2N$: 212.0682] found m/z 212.0685.

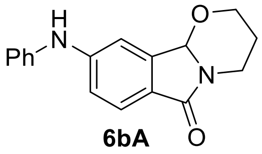

6bA

9-(phenylamino)-3,4-dihydro-2H-[1,3]oxazino[2,3-a]isoindol-6(10bH)-one (6bA). Isolated yield: 80%. GC-MS (m/z, $M^{+\cdot}$ 280), major peaks found: 280 (100%), 251 (30%), 222 (28%), 207 (9%), 195 (12%), 167 (30%), 139 (7%). ^{1}H NMR (400 MHz, DMSO-d_6) δ: 8.73 (s, NH), 7.51 (d, *J* = 8.2, 1H), 7.32 (td, *J* = 7.5, 1.8, 2H), 7.20-7.14 (m, 3H), 7.12 (dd, *J* = 8.3, 2.0, 1H), 6.98 (t, *J* = 7.3, 1H), 5.59 (s, 1H), 4.20-4.12 (m, 1H), 4.11-4.04 (m, 1H), 3.93-3.81 (m, 1H), 3.27-3.15 (m, 1H), 1.64-1.49 (m, 2H). ^{13}C NMR (101 MHz, DMSO-d_6) δ: 165.00 (C=O), 147.74 (C), 143.62 (C), 141.56 (C), 129.35 (2xCH), 124.13 (CH), 122.33 (C), 121.74 (CH), 119.13 (2xCH), 116.18 (CH), 108.80 (CH), 84.02 (O-CH-N), 66.35 (O-CH_2), 37.07 (N-CH_2), 24.56 (CH_2). HRMS (ESI) [M+H^+; calculated for $C_{17}H_{16}O_2N_2$: 281.1284] found m/z 281.1283.

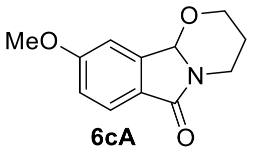

6cA

9-methoxy-3,4-dihydro-2H-[1,3]oxazino[2,3-a]isoindol-6(10bH)-one (6cA). Isolated yield: 75%. GC-MS (m/z, $M^{+\cdot}$ 219), major peaks found: 219 (100%), 218 (90%), 190 (90%), 176 (15%), 161 (20%), 146 (12%), 134 (50%), 119 (11%), 106 (14%), 92 (8%), 77 (12%), 63 (14%). ^{1}H NMR (300 MHz, $CDCl_3$): 7.71 (d, *J* = 8.3, 1H), 7.05 (d, *J* = 2.2, 1H), 7.00 (dd, *J* = 8.3, 2.3, 1H), 5.50 (s, 1H), 4.42 (dd, *J* = 13.4, 5.4, 1H), 4.28-4.13 (m, 1H), 3.92 (td, *J* = 12.2, 2.2, 1H), 3.85 (s, 3H), 3.22 (td, *J* = 12.9, 3.9, 1H), 1.91-1.75 (m, 1H), 1.61 (ddd, *J* = 13.5, 3.7, 1.9, 1H). ^{13}C NMR (75 MHz, $CDCl_3$): 166.01 (C=O), 163.19 (C), 143.56 (C), 125.23 (C), 125.09 (CH), 116.38 (CH), 108.26 (CH), 84.86 (O-CH-N), 67.48 (O-CH_2), 55.82 (O-CH_3), 37.87 (N-CH_2), 24.80 (CH_2). HRMS (ESI) [M+Na^+; calculated for $C_{12}H_{13}O_3N$: 242.0787] found m/z 242.0787.

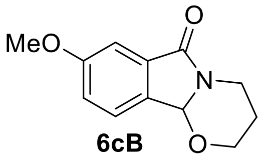

6cB

8-methoxy-3,4-dihydro-2H-[1,3]oxazino[2,3-a]isoindol-6(10bH)-one (6cB). Isolated yield: 15%. GC-MS (m/z, $M^{+\cdot}$ 219), major peaks found: 219 (80%), 218 (100%), 190 (90%), 176 (12%), 161 (40%), 146 (8%), 135 (38%), 119 (7%), 106 (12%), 92 (8%), 77 (12%), 63 (14%). ^{1}H NMR (300 MHz, $CDCl_3$) δ: 7.45 (d, *J* = 8.3, 1H), 7.32 (d, *J* = 2.4, 1H), 7.09 (dd, *J* = 8.3, 2.4, 1H), 5.50 (s, 1H), 4.46 (ddt, *J* = 13.4, 5.4, 1.5, 1H), 4.22 (ddt, *J* = 11.6, 3.7, 1.6, 1H), 3.92 (td, *J* = 12.3, 2.2, 1H), 3.86 (s, 3H), 3.25 (td, *J* = 12.9, 4.0, 1H), 1.95-1.76 (m, 1H), 1.64-1.58 (m, 1H). ^{13}C NMR (75 MHz, $CDCl_3$): 166.03 (C=O), 161.53 (C), 134.62 (C), 133.55 (C), 124.17 (CH), 119.47 (CH), 107.28 (CH), 85.08 (O-CH-N), 67.31 (O-CH_2), 55.89 (O-CH_3), 38.15 (N-CH_2), 24.75 (CH_2). HRMS (ESI) [M+H^+; calculated for $C_{12}H_{13}O_3N$: 220.0968] found m/z 220.0967.

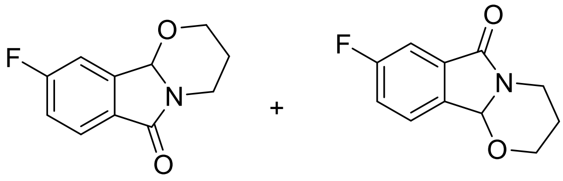

6dA (64%) **6dB** (36%)

9-fluoro-3,4-dihydro-2H-[1,3]oxazino[2,3-a]isoindol-6(10bH)-one (6dA) / 8-fluoro-3,4-dihydro-2H-[1,3]oxazino[2,3-a]isoindol-6(10bH)-one (6dB). Isolated yield: 86%. GC-MS (m/z, $M^{+\cdot}$ 207) (compound **6dA**), major peaks found: 207 (70%), 206 (100%), 178 (80%), 164 (15%), 148 (60%), 122 (50%), 108 (15%), 94 (25%), 75 (12%). GC-MS (m/z, $M^{+\cdot}$ 207) (compound **6dB**), major peaks found: 207 (70%), 206 (100%), 178 (80%), 164 (15%), 148 (60%), 122 (50%), 108 (15%), 94 (25%), 75 (12%). ^{1}H NMR (300 MHz, $CDCl_3$) δ: (compound **6dA**) 7.75 (dd, *J* = 8.3, 4.9, 1H), 7.25-7.12 (m, 2H), 5.49 (s, 1H), 4.46-4.35 (m, 1H), 4.19-4.15 (m, 1H), 3.89 (td, *J* = 12.1, 2.2, 1H), 3.29-3.13 (m, 1H), 1.90-1.72 (m, 1H), 1.65-1.61 (m, 2H), (compound **6dB**) 7.49 (dd, *J* = 8.3, 4.5, 1H), 7.44 (dd, *J* = 7.5, 2.4, 1H), 7.25-7.12 (m, 1H), 5.49 (s, 1H), 4.46-4.35 (m, 1H), 4.24-4.19 (m, 1H), 3.89 (td, *J* = 12.1, 2.2, 1H), 3.29-3.13 (m, 1H), 1.90-1.72 (m, 1H), 1.60-1.56 (m, 1H). ^{13}C NMR (75 MHz, $CDCl_3$) δ: (compound **6dA**)

165.21 (d, J^1_{C-F} = 252.3, C), 164.90 (C=O), 143.61 (d, J^3_{C-F} = 9.8, C), 128.76 (d, J^4_{C-F} = 2.5, C), 125.59 (d, J^3_{C-F} = 9.5, CH), 117.39 (d, J^2_{C-F} = 23.4, CH), 110.99 (d, J^2_{C-F} = 24.5, CH), 84.43 (d, J^4_{C-F} = 2.5, O-CH-N), 67.40 (O-CH_2), 37.92 (N-CH_2), 24.58 (CH_2), (compound **6dB**) 164.69 (d, J^4_{C-F} = 3.3, C=O), 163.93 (d, J^1_{C-F} = 252.3, C), 136.80 (d, J^4_{C-F} = 2.7, C), 135.22 (d, J^3_{C-F} = 8.9, C), 124.88 (d, J^3_{C-F} = 8, CH), 119.06 (d, J^2_{C-F} = 23.5, CH), 110.75 (d, J^2_{C-F} = 23.9, CH), 84.70 (O-CH-N), 67.28 (O-CH_2), 38.05 (N-CH_2), 24.51 (CH_2). ^{19}F NMR (282 MHz, $CDCl_3$) δ: (compound **6dA**) -106.39 (td, J^3_{F-H} = 8.4, J^4_{F-H} = 5.2, 1F), (compound **6dB**) -109.75 (td, J^3_{F-H} = 8.2, J^4_{F-H} = 4.8, 1F). HRMS (ESI) (compound **6dA**) [M+Na$^+$; calculated for $C_{11}H_{10}O_2NF$: 230.0587], found m/z 230.0588. HRMS (ESI) (compound **6dB**) [M+Na$^+$; calculated for $C_{11}H_{10}O_2NF$: 230.0587], found m/z 230.0588.

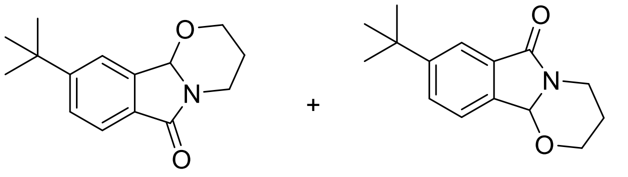

6eA (50%) **6eB** (50%)

9-(tert-butyl)-3,4-dihydro-2H-[1,3]oxazino[2,3-a]isoindol-6(10bH)-one (6eA) / 8-(tert-butyl)-3,4-dihydro-2H-[1,3]oxazino[2,3-a]isoindol-6(10bH)-one (6eB). Isolated yield: 87%. GC-MS (m/z, M$^{+\cdot}$ 245) (compound **6eA** or **6eB**), major peaks found: 245 (75%), 244 (100%), 230 (15%), 216 (70%), 202 (30%), 188 (45%), 172 (43%), 160 (22%), 145 (24%), 131 (9%), 115 (22%), 103 (7%), 91 (11%). GC-MS (m/z, M$^{+\cdot}$ 245) (compound **6eB** or **6eA**), major peaks found: 245 (75%), 244 (100%), 230 (20%), 216 (75%), 202 (23%), 188 (75%), 172 (45%), 160 (22%), 144 (18%), 131 (16%), 115 (19%), 103 (13%), 91 (16%). ^{1}H NMR (300 MHz, $CDCl_3$) δ: (compound **6eA** and **6eB**) 7.85 (d, *J* = 1.6, 1H, **6eB**), 7.74 (d, *J* = 7.9, 1H, **6eA**), 7.62-7.57 (m, 2H), 7.55 (dd, *J* = 8.0, 1.7, 1H, **6eA**), 7.48 (d, *J* = 7.9, 1H, **6eB**), 5.53 (s, 1H), 5.52 (s, 1H), 4.51-4.40 (m, 2H), 4.27-4.16 (m, 2H), 3.93 (tdd, *J* = 12.1, 4.7, 2.2, 2H), 3.24 (td, *J* = 12.8, 3.9, 2H), 1.93-1.74 (m, 2H), 1.66-1.57 (m, 2H), 1.34 (s, 9H), 1.34 (s, 9H). ^{13}C NMR (75 MHz, $CDCl_3$) δ: (compound **6eA** and **6eB**) 166.45 (C=O), 166.12 (C=O), 156.06 (C), 153.84 (C), 141.35 (C), 138.52 (C), 132.72 (C), 130.17 (C), 129.22 (CH), 127.39 (CH), 123.32 (CH), 122.74 (CH), 120.60 (CH), 120.02 (CH), 85.28 (CH), 85.07 (CH), 67.43 (O-CH_2), 67.32 (O-CH_2), 37.98 (N-CH_2), 37.89 (N-CH_2), 35.46 (C), 35.24 (C), 31.45 (3xCH_3), 31.43 (3xCH_3), 24.76 (CH_2). HRMS (ESI) (compound **6eA** and **6eB**) [M+Na$^+$; calculated for $C_{15}H_{19}O_2N$: 268.1308], found m/z 268.1308.

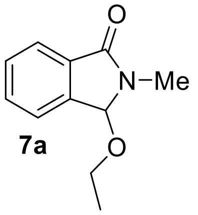

7a

3-ethoxy-2-methylisoindolin-1-one (7a).[10] Isolated yield: 93%. GC-MS (m/z, M$^{+\cdot}$ 191), major peaks found: 191 (3%), 162 (6%), 146 (100%), 91 (8%). (The NMR spectrum is consistent with the reported data). ^{1}H NMR (300 MHz, $CDCl_3$) δ: 7.78 (dt, *J* = 7.2, 1.1, 1H), 7.61-7.42 (m, 3H), 5.73 (s, 1H), 3.15 (dq, *J* = 9.1, 7.1, 1H), 3.06 (s, 3H), 2.99 (dq, *J* = 9.2, 7.0, 1H), 1.12 (t, *J* = 7.0, 2H). ^{13}C NMR (75 MHz, $CDCl_3$): 167.70 (C=O), 141.04 (C), 133.01 (C), 131.97 (CH), 129.89 (CH), 123.38 (CH), 123.31 (CH), 87.86 (O-CH-N), 57.85 (O-CH_2), 26.59 (N-CH_3), 15.21 (CH_3). HRMS (ESI) [M$^+$; calculated for $C_{11}H_{13}O_2N$: 191.0940] found m/z 191.0939.

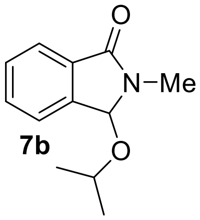

7b

3-isopropoxy-2-methylisoindolin-1-one (7b).[11] Isolated yield: 94%. GC-MS (m/z, $M^{+\cdot}$ 205), major peaks found: 205 (3%), 162 (6%), 146 (100%), 91 (8%). (The NMR spectrum is consistent with the reported data). ^{1}H NMR (300 MHz, $CDCl_3$) δ: 7.75 (d, *J* = 7.2, 1H), 7.56-7.49 (m, 2H), 7.49-7.42 (m, 1H), 5.65 (s, 1H), 3.62 (hept, *J* = 6.2, 1H), 3.07 (s, 3H), 1.16 (d, *J* = 6.1, 3H), 1.07 (d, *J* = 6.2, 3H). ^{13}C NMR (75 MHz, $CDCl_3$) δ: 167.54 (C=O), 142.03 (C), 132.68 (C), 131.83 (CH), 129.75 (CH), 123.41 (CH), 123.26 (CH), 87.93 (O-CH-N), 68.27 (CH), 26.94 (N-CH_3), 23.73 (CH_3), 23.69 (CH_3). HRMS (ESI) [M^+; calculated for $C_{12}H_{15}O_2N$: 205.1097] found m/z 205.1100.

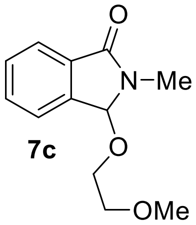

7c

3-(2-methoxyethoxy)-2-methylisoindolin-1-one (7c).[11] Isolated yield: 90%. GC-MS (m/z, $M^{+\cdot}$ 221), major peaks found: 221 (2%), 192 (4%), 162 (20%), 146 (100%), 91 (10%). (The NMR spectrum is consistent with the reported data). ^{1}H NMR (400 MHz, $CDCl_3$) δ: 7.79 (d, *J* = 7.4 , 1H), 7.56-7.53 (m, 2H), 7.51-7.46 (m, 1H), 5.82 (s, 1H), 3.44 (t, *J* = 4.6, 2H), 3.34 (s, 3H), 3.20 (dt, *J* = 10.6, 4.5, 1H), 3.10-3.04 (m, 4H). ^{13}C NMR (101 MHz, $CDCl_3$) δ: 167.69 (C=O), 140.56 (C), 133.05 (C), 132.08 (CH), 130.04 (CH), 123.51 (CH), 123.43 (CH), 87.86 (O-CH-N), 71.53 (O-CH_2), 61.15 (O-CH_2), 59.07 (O-CH_3), 26.57 (N-CH_3). HRMS (ESI) [M^+; calculated for $C_{12}H_{15}O_3N$: 221.1046] found m/z 221.1047.

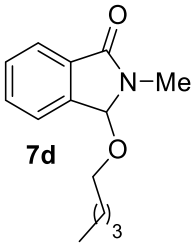

7d

2-methyl-3-(pentyloxy)isoindolin-1-one (7d). Isolated yield: 95%. GC-MS (m/z, $M^{+\cdot}$ 233), major peaks found: 233 (3%), 162 (6%), 146 (100%), 91 (8%). ^{1}H NMR (300 MHz, $CDCl_3$) δ: 7.73 (dt, *J* = 7.1, 1.0, 1H), 7.53-7.39 (m, 3H), 5.68 (s, 1H), 3.02 (m, 1H), 3.01 (s, 3H), 2.91-2.80 (m, 1H), 1.49-1.39 (m, 2H), 1.25-1.15 (m, 4H), 0.83-0.75 (m, 2H). ^{13}C NMR (75 MHz, $CDCl_3$) δ: 167.50 (C=O), 140.95 (C), 132.94 (C), 131.78 (CH), 129.70 (CH), 123.19 (2xCH), 87.70 (O-CH-N), 62.10 (O-CH_2), 29.21 (CH_2), 28.26 (CH_2), 26.45 (N-CH_3), 22.35 (CH_2), 13.91 (CH_3). HRMS (ESI) [M+H^+; calculated for $C_{14}H_{19}O_2N$: 234.1488] found m/z 234.1489.

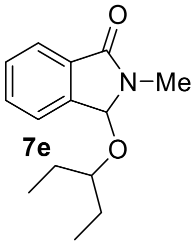

7e

2-methyl-3-(pentan-3-yloxy)isoindolin-1-one (7e). Isolated yield: 83%. GC-MS (m/z, $M^{+\cdot}$ 233), major peaks found: 233 (3%), 164 (6%), 146 (100%), 133 (5%), 91 (9%). ^{1}H NMR (400 MHz, $CDCl_3$) δ: 7.76 (dt, *J* = 7.3, 0.9, 1H), 7.58-7.38 (m, 3H), 5.65 (s, 1H), 3.43 (quint, *J* = 5.7, 1H), 3.09 (s, 3H), 1.64-1.53 (m, 2H), 1.53-1.42 (m, 2H), 0.87 (td, *J* = 7.5, 1.7, 6H). ^{13}C NMR (101 MHz, $CDCl_3$) δ: 167.55 (C=O), 142.41 (C), 132.51 (C), 131.77 (CH), 129.66 (CH), 123.39 (CH), 123.27 (CH), 88.22 (O-CH-N), 79.22 (O-CH), 27.22 (N-CH_3), 26.72 (CH_2), 26.17 (CH_2), 9.50 (CH_3), 9.09 (CH_3). HRMS (ESI) [M+Na^+; calculated for $C_{14}H_{19}O_2N$: 256.1308] found m/z 256.1308.

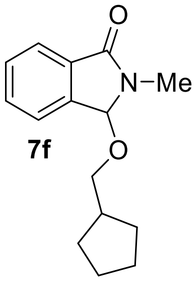

7f

3-(cyclopentylmethoxy)-2-methylisoindolin-1-one (7f). Isolated yield: 93%. GC-MS (m/z, $M^{+\cdot}$ 245), major peaks found: 245 (2%), 164 (10%), 146 (100%), 91 (8%). ^{1}H NMR (300 MHz, $CDCl_3$) δ: 7.75 (dt, *J* = 7.1, 1.1, 1H), 7.57-7.38 (m, 3H), 5.71 (s, 1H), 3.03 (s, 3H), 2.91 (dd, *J* = 8.8, 7.1, 1H), 2.74 (dd, *J* = 8.8, 7.0, 1H), 2.10-1.93 (m, 1H), 1.73-1.57 (m, 2H), 1.51-1.39 (m, 4H), 1.16-0.99 (m, 2H). ^{13}C NMR (75 MHz, $CDCl_3$) δ: 167.59 (C=O), 140.93 (C), 132.97 (C), 131.83 (CH), 129.74 (CH), 123.24 (2xCH), 87.67 (O-CH-N), 66.38 (O-CH_2), 39.27 (CH), 29.60 (CH_2), 29.54 (CH_2), 26.53 (N-CH_3), 25.30 (CH_2), 25.26 (CH_2). HRMS (ESI) [M+Na^+; calculated for $C_{15}H_{19}O_2N$: 268.1308] found m/z 268.1306.

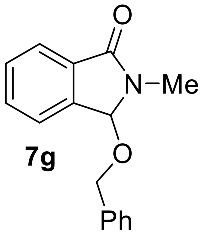

7g

3-(benzyloxy)-2-methylisoindolin-1-one (7g).[11] Isolated yield: 89%. GC-MS (m/z, $M^{+\cdot}$ 253), major peaks found: 253 (2%), 162 (2%), 146 (100%), 91 (15%). (The NMR spectrum is consistent with the reported data). ^{1}H NMR (400 MHz, $CDCl_3$) δ: 7.74 (dt, *J* = 7.2, 1.0, 1H), 7.51-7.41 (m, 3H), 7.26-7.13 (m, 5H), 5.79 (s, 1H), 4.04 (d, *J* = 11.2, 1H), 3.92 (d, *J* = 11.2, 1H), 3.00 (s, 3H). ^{13}C NMR (75 MHz, $CDCl_3$) δ: 167.70 (C=O), 140.63 (C), 137.23 (C), 133.00 (C), 132.10 (CH), 130.06 (CH), 128.52 (2xCH), 127.94 (CH), 127.83 (2xCH), 123.45 (2xCH), 87.87 (O-CH-N), 64.43 (O-CH_2), 26.72 (N-CH_3). HRMS (ESI) [M+H^+; calculated for $C_{16}H_{16}O_2N$: 254.1175] found m/z 254.1176.

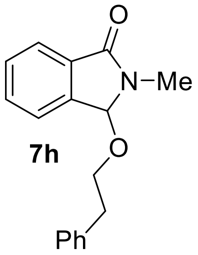

7h

2-methyl-3-phenethoxyisoindolin-1-one (7h).[11] Isolated yield: 91%. GC-MS (m/z, $M^{+\cdot}$ 267), major peaks found: 267 (2%), 162 (10%), 146 (100%), 91 (11%). (The NMR spectrum is consistent with the reported data). ^{1}H NMR (400 MHz, $CDCl_3$) δ: 7.69 (dd, *J* = 6.2, 1.8, 1H), 7.41 (td, *J* = 6.2, 1.2, 2H), 7.27-7.24 (m, 1H), 7.21-7.15 (m, 2H), 7.14-7.09 (m, 1H), 7.06-7.02 (m, 2H), 5.63 (s, 1H), 3.21 (dt, *J* = 9.1, 6.8, 1H), 3.07 (dt, *J* = 9.2, 6.5, 1H), 2.82 (s, 3H), 2.71 (t, *J* = 6.7, 2H). ^{13}C NMR (101 MHz, $CDCl_3$) δ: 167.62 (C=O), 140.66 (C), 138.65 (C), 133.00 (C), 131.91 (CH), 129.87 (CH), 128.99 (2xCH), 128.41 (2xCH), 126.44 (CH), 123.33 (CH), 123.32 (CH), 87.76 (O-CH-N), 62.94 (O-CH_2), 36.16 (CH_2), 26.41 (N-CH_3). HRMS (ESI) [M+H^+; calculated for $C_{17}H_{18}O_2N$: 268.1332] found m/z 268.1333.

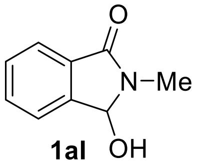

1al

3-hydroxy-2-methylisoindolin-1-one (1al).[16] Isolated yield: 71%. GC-MS (m/z, $M^{+\cdot}$ 163), major peaks found: 163 (80%), 162 (100%), 146 (65%), 133 (30%), 105 (45%), 91 (18%), 77 (30%). (The NMR spectrum is consistent with the reported data). ^{1}H NMR (400 MHz, $CDCl_3$) δ: 7.54 (d, *J* = 7.5, 1H), 7.48 (td, *J* = 7.3, 1.2, 1H), 7.42-7.38 (m, 1H), 7.33 (td, *J* = 7.4, 1.1, 1H), 5.53 (s, 1H), 2.82 (s, 3H). ^{13}C NMR (101 MHz, $CDCl_3$) δ: 167.68 (C=O), 143.91 (C), 132.09 (CH), 131.25 (C), 129.51 (CH), 123.22 (CH), 122.88 (CH), 83.54 (CH-OH), 26.04 (N-CH_3). HRMS (EI) [M^+; calculated for $C_9H_9O_2N$: 163.0627] found m/z 163.0623.

7. REFERENCES

[1] Dutta Chowdhury, A.; Jackstell, R.; Beller, M. *ChemCatChem* **2014**, *6*, 3360

[2] Upadhyay, S. K.; Pingali, S. R. K.; Jursic, B. S. *Tetrahedron Lett.* **2010**, *51*, 2215

[3] Barder, T. E.; Walker, S. D.; Martinelli, J. R.; Buchwald, S. L. *J. Am. Chem. Soc.* **2005**, *127*, 4685

[4] Yeung, S. Y.; Kampmann, S.; Stubbs, K. A.; Skelton, B. W.; Kaskow, B. J.; Abraham, L. J. Stewart, S. G. *Med. Chem. Commun.* **2011**, *2*, 1073

[5] Ding, G.; Li, C.; Shen, Y.; Lu, B.; Zhang, Z.; Xie, X. *Adv. Synt. Catal.* **2016**, *358*, 1241

[6] Chen, D. C.; Ye, H. Q.; Wu, H. *Cat. Commun.* **2007**, *8*, 1527

[7] Nammalwar, B.; Muddala, N. P.; Watts, F. M.; Bunce, R. A. *Tetrahedron* **2015**, *71*, 9101

[8] Assis, S. P. O.; Araujo, T. G.; Sena, V. L. M.; Catanho, M. T. J. A.; Ramos, M. N.; Srivastava, R. M.; Lima, V. L. M. *Med. Chem. Res.* **2014**, *23*, 708

[9] Ungwitayatorn, J.; Wiwat, C.; Matayatsuk, C.; Pimthon, J.; Piyaviriyakul, S. *Chin. J. Chem.* **2008**, *26*, 379

[10] Kobayashi, K:; Chikazawa, Y.; Ezaki, K. *Helv. Chim. Acta* **2015**, *98*, 604

[11] Cabrero-Antonino, J. R.; Sorribes, I.; Junge, K.; Beller, M. *Angew. Chem., Int. Ed.* **2016**, *55*, 387

[12] Kim, S. S.; Mah, Y. J.; Lee, H. J.; Park, S. K. *J. Photosci.* **2003**, *10*, 241

[13] Yan, S. F.; Belov, V. N.; Bossi, M. L.; Hell, S. W. *Eur. J. Org. Chem.* **2008**, *15*, 2531

[14] Williams, F. J.; Donahue, P. E. *J. Org. Chem.* **1977**, *42*, 3414

[15] Du, Y.; Hyster, T. K.; Rovis, T. *Chem. Commun.* **2011**, *47*, 12074

[16] Yuan, X.-h.; Zhang, M.-j.; Kang, C.-q.; Guo, H.-q.; Qiu, X.-p.; Gao, L.-x. *Synth. Commun.* **2006**, *36*, 435

8. NMR SPECTRA OF THE ISOLATED PRODUCTS

^{1}H NMR

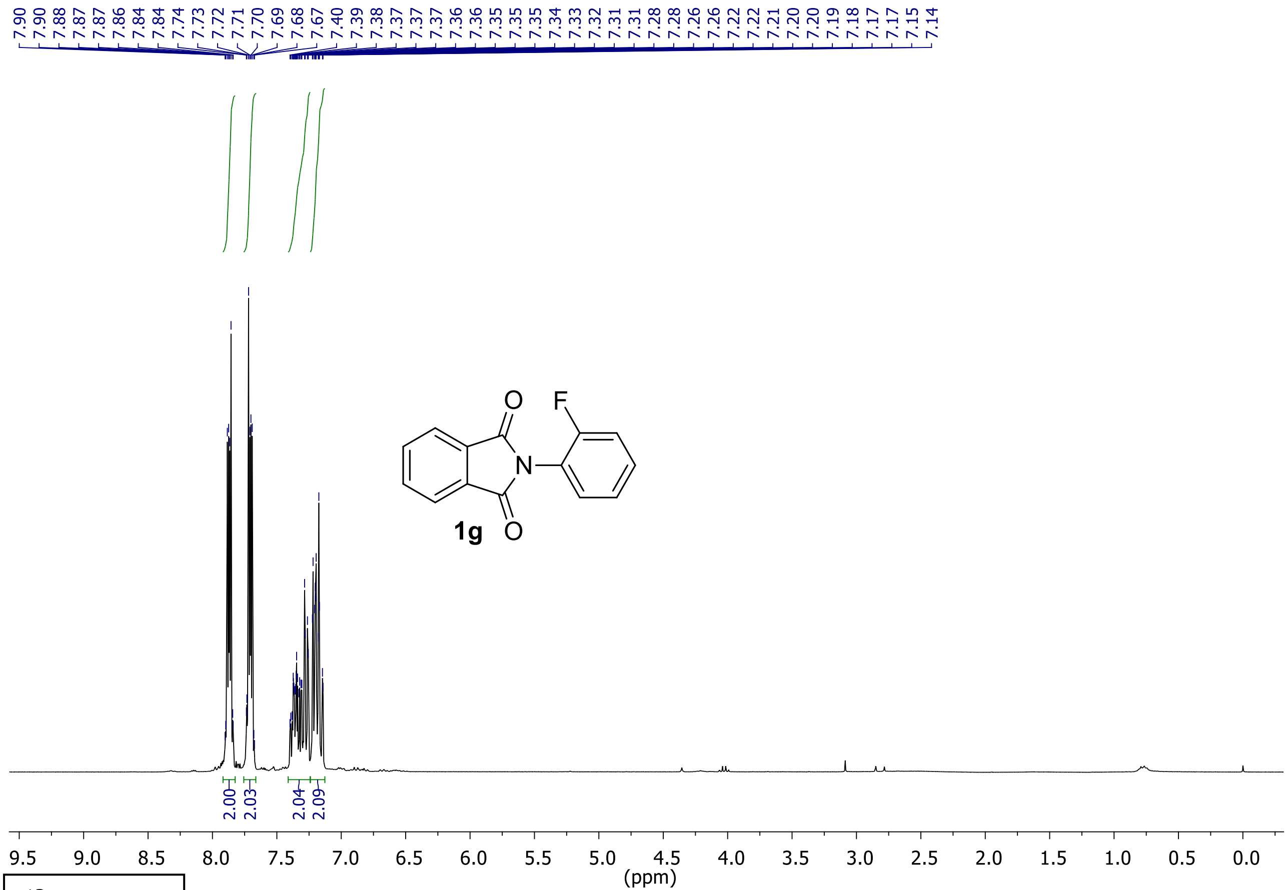

^{13}C NMR

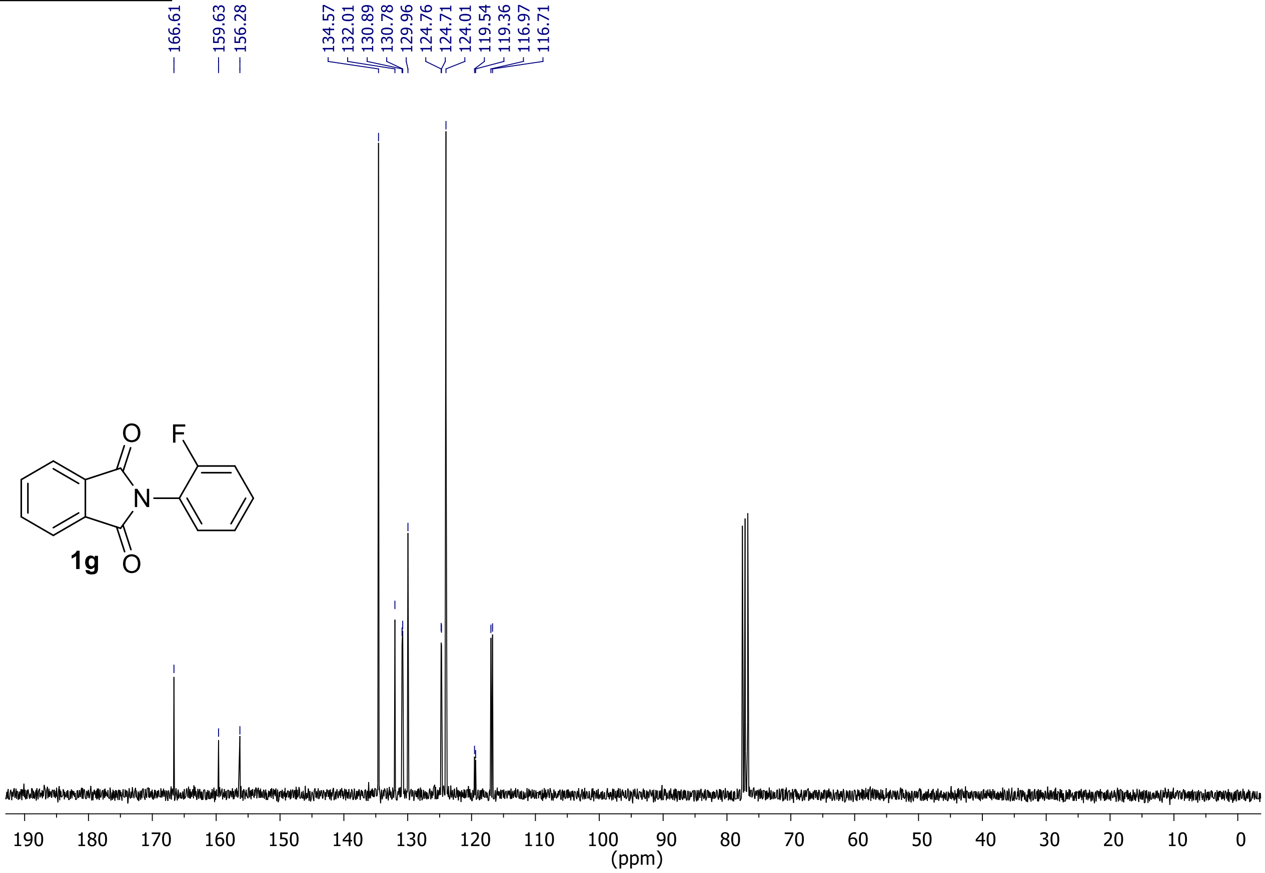

^{19}F NMR

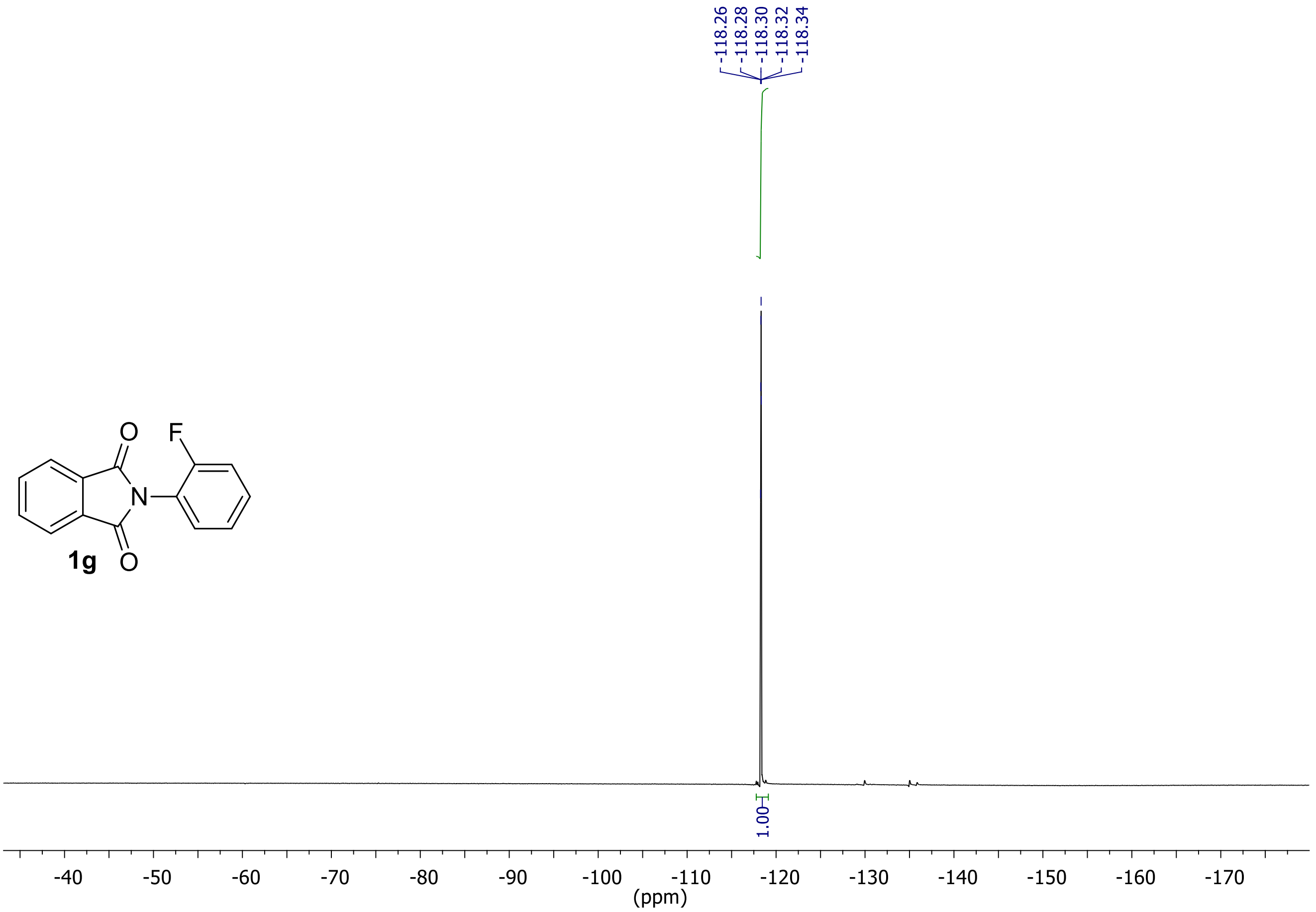

-118.26
-118.28
-118.30
-118.32
-118.34
O
F
N
O
1.00
-40
-50
-60
-70
-80
-90
-100
-110
-120
-130
-140
-150
-160
-170
(ppm)

1g

^{1}H NMR

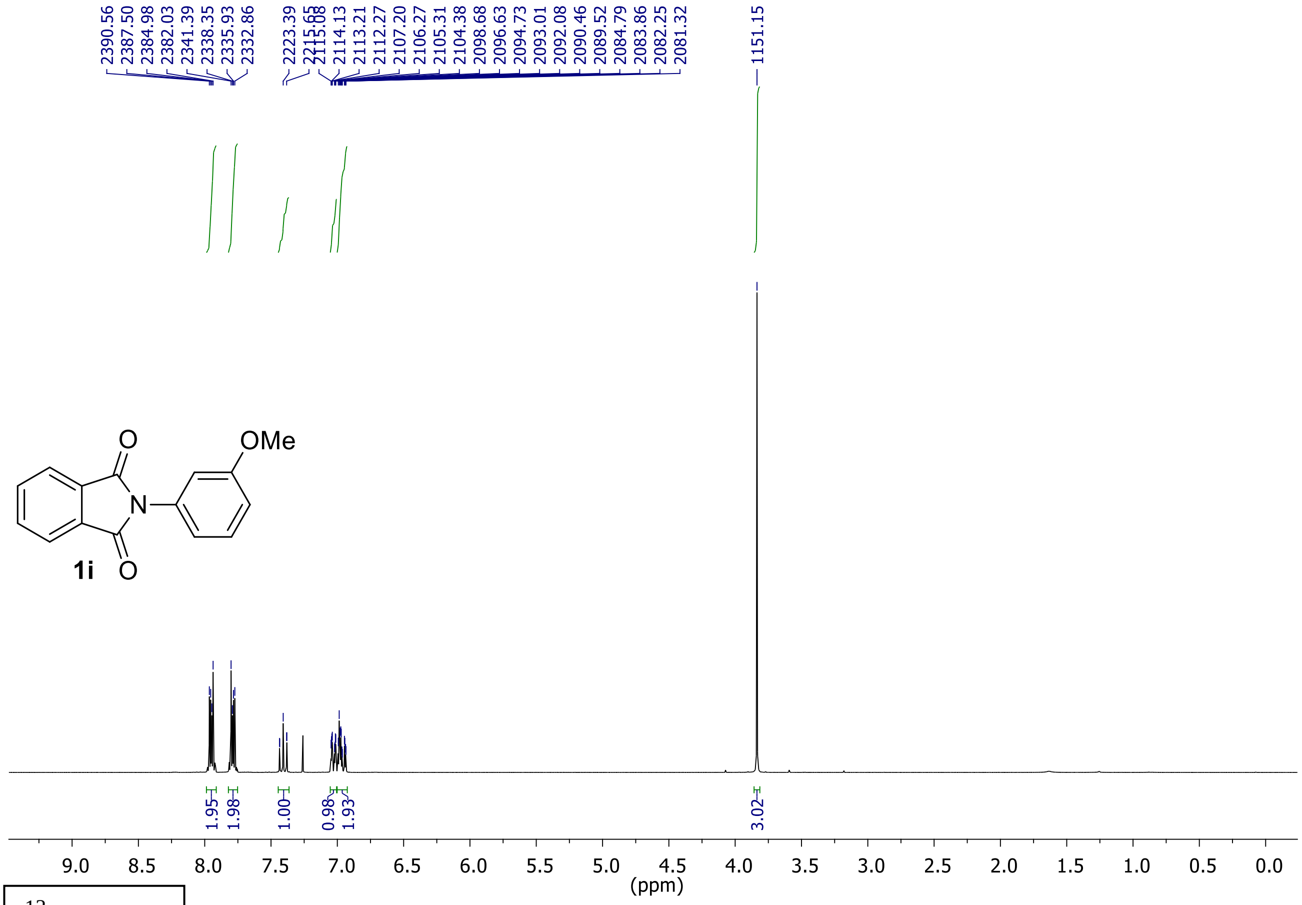

^{13}C NMR

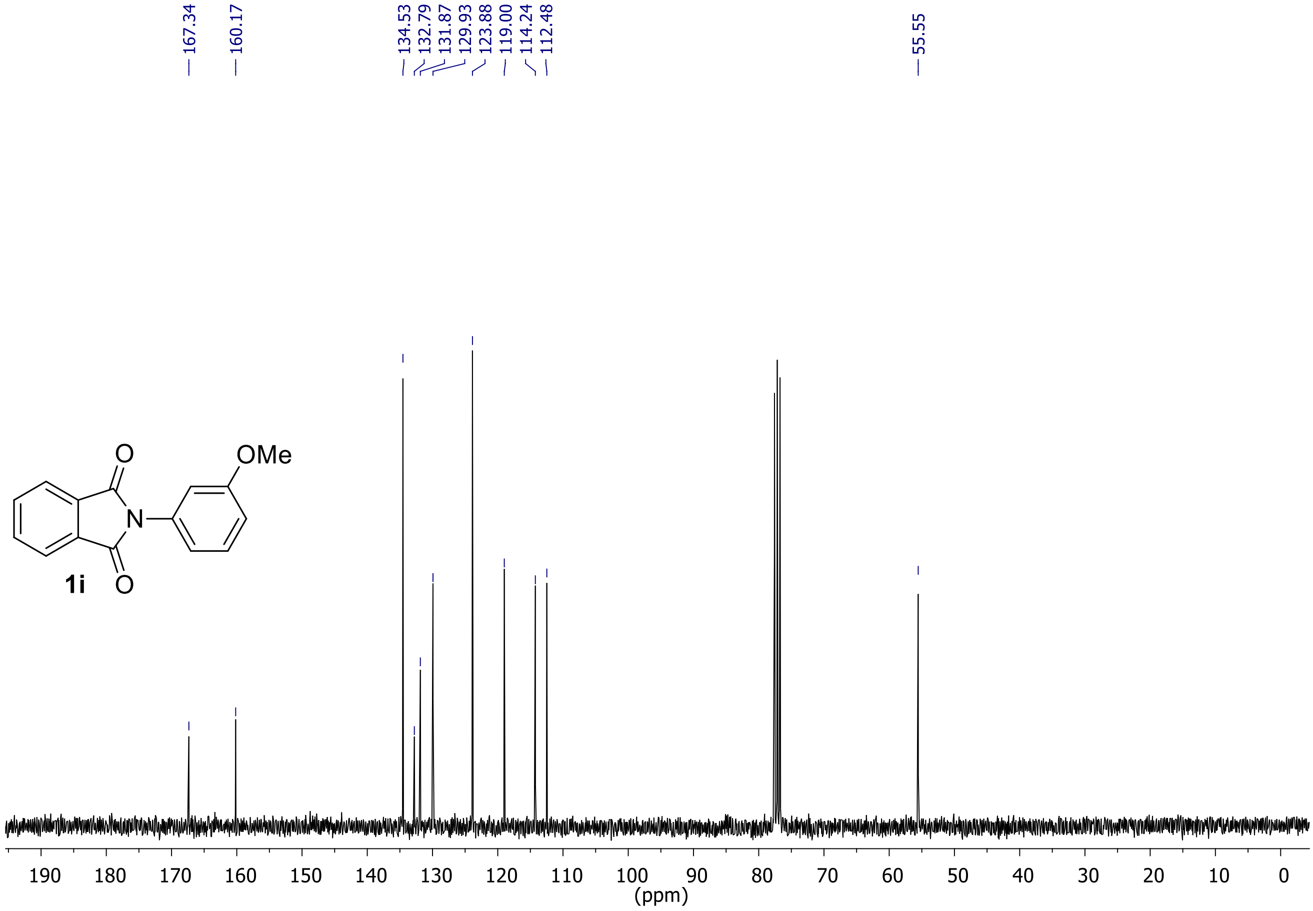

^{1}H NMR

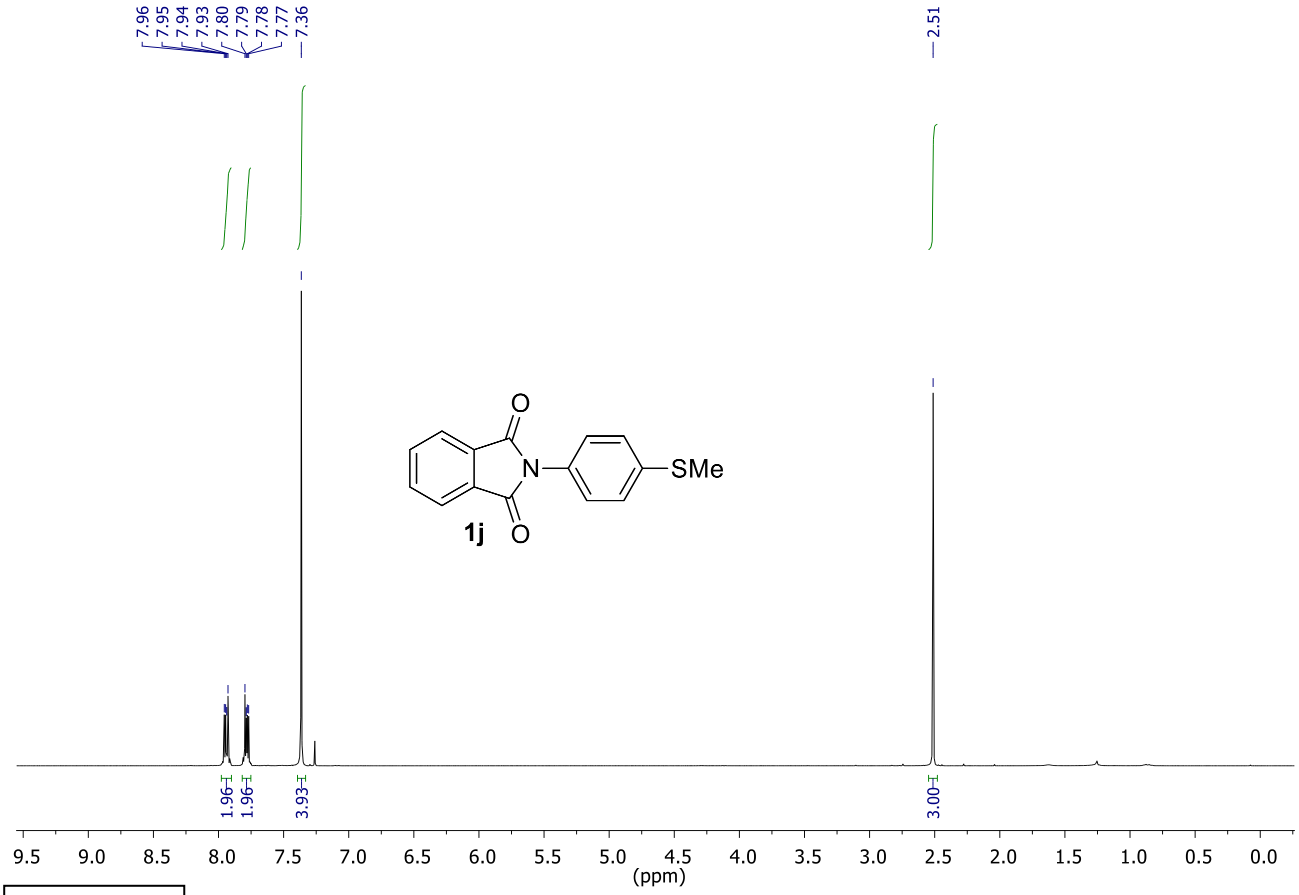

^{13}C NMR

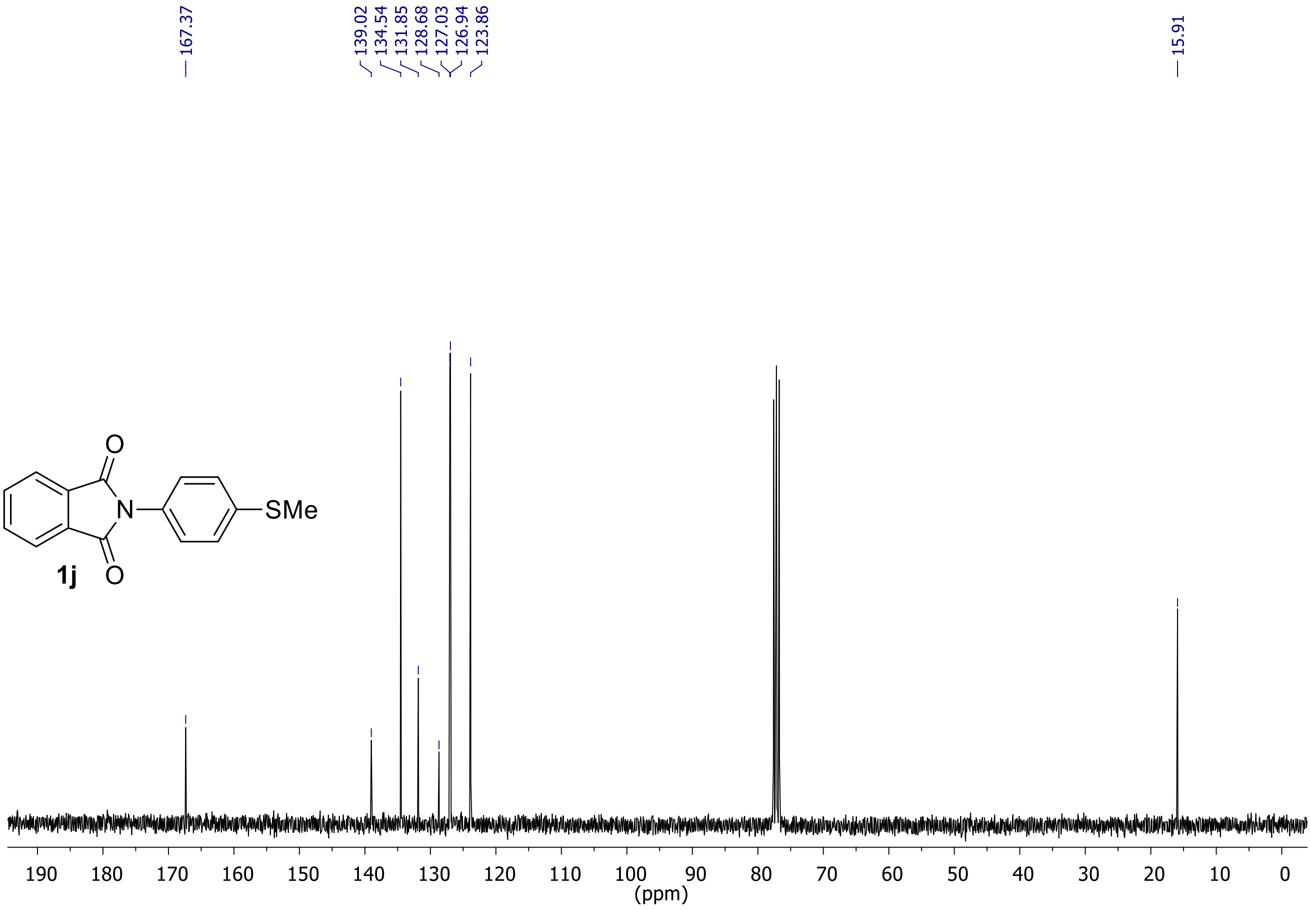

^{1}H NMR

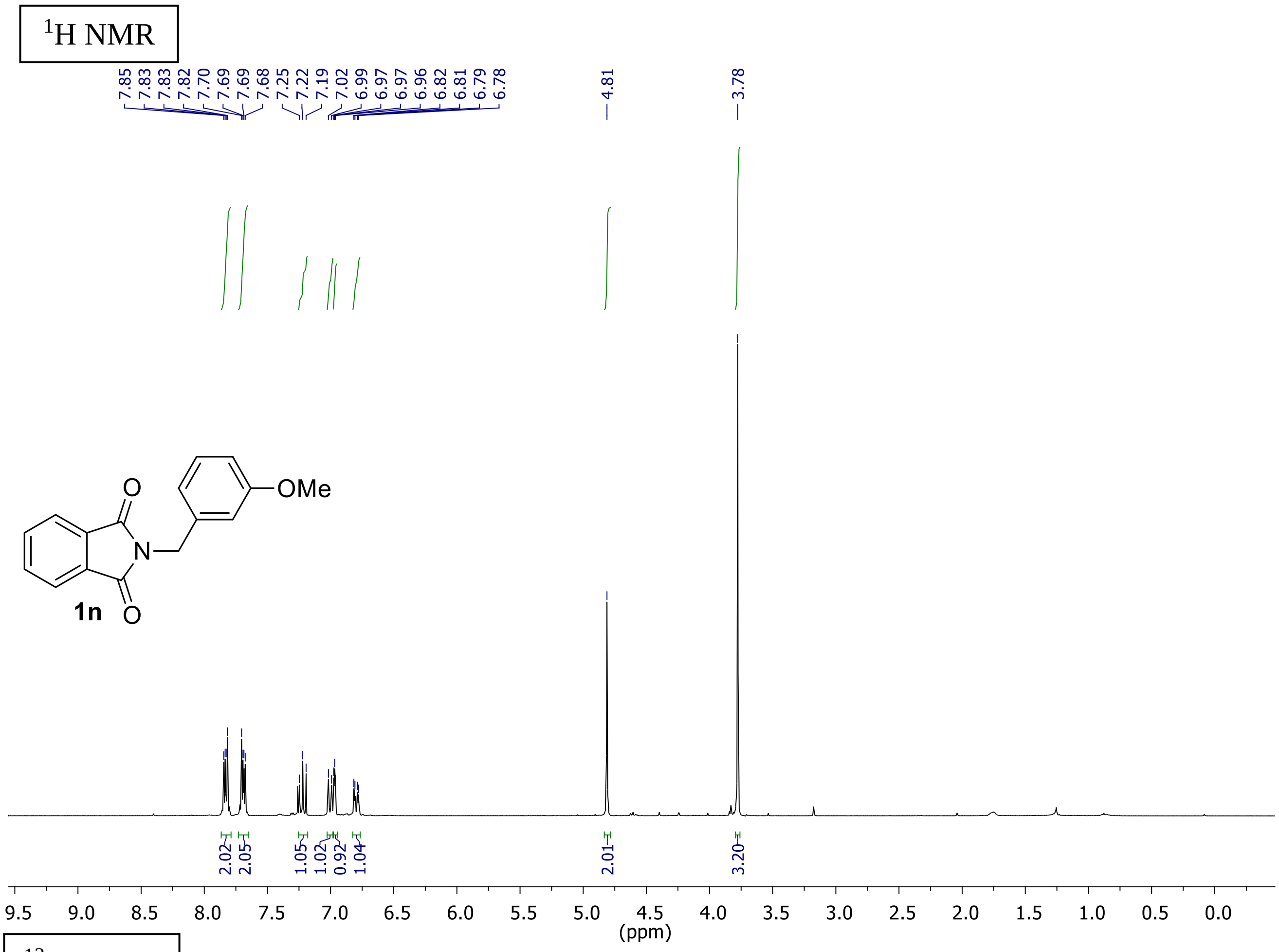

^{13}C NMR

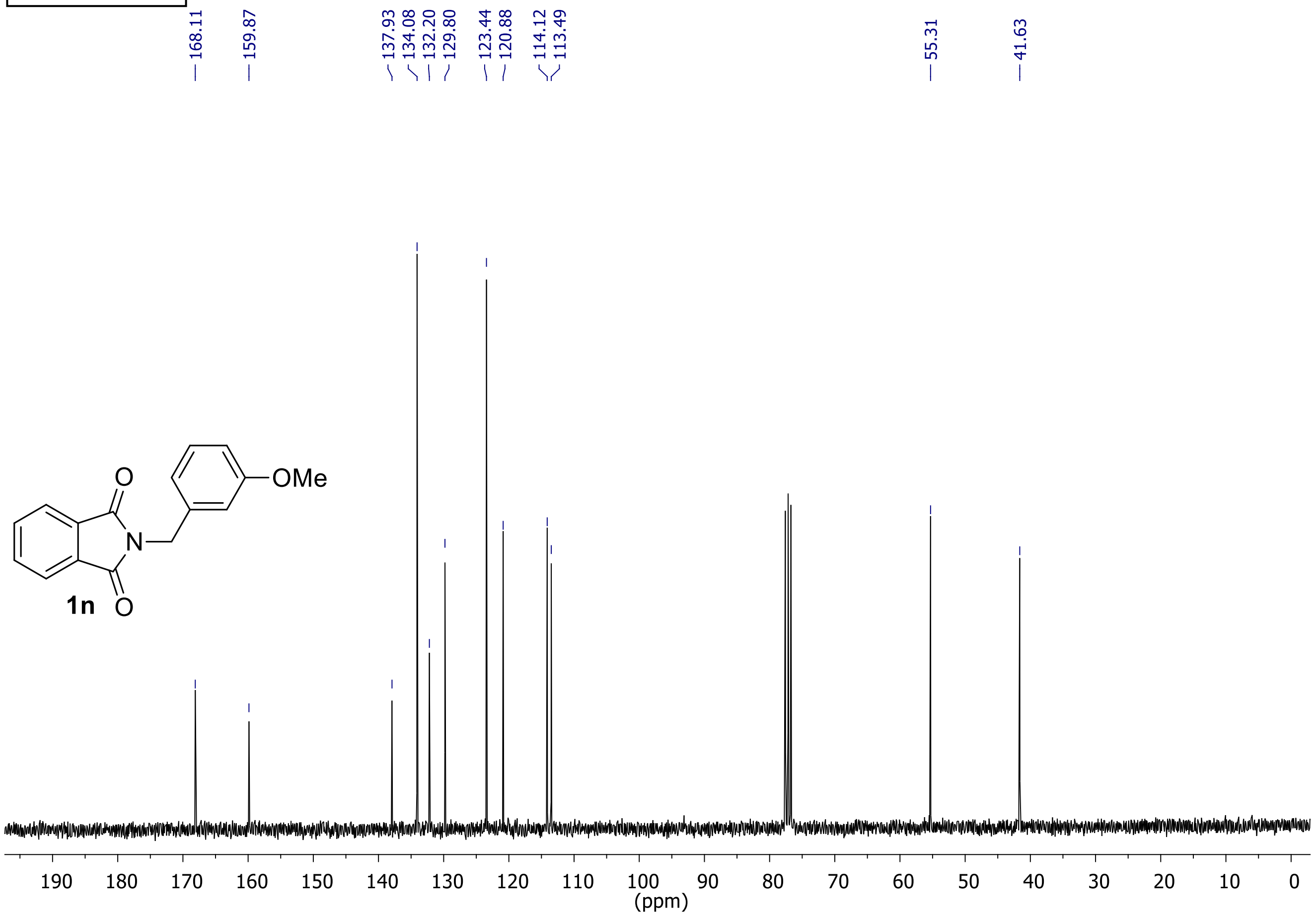

^{1}H NMR

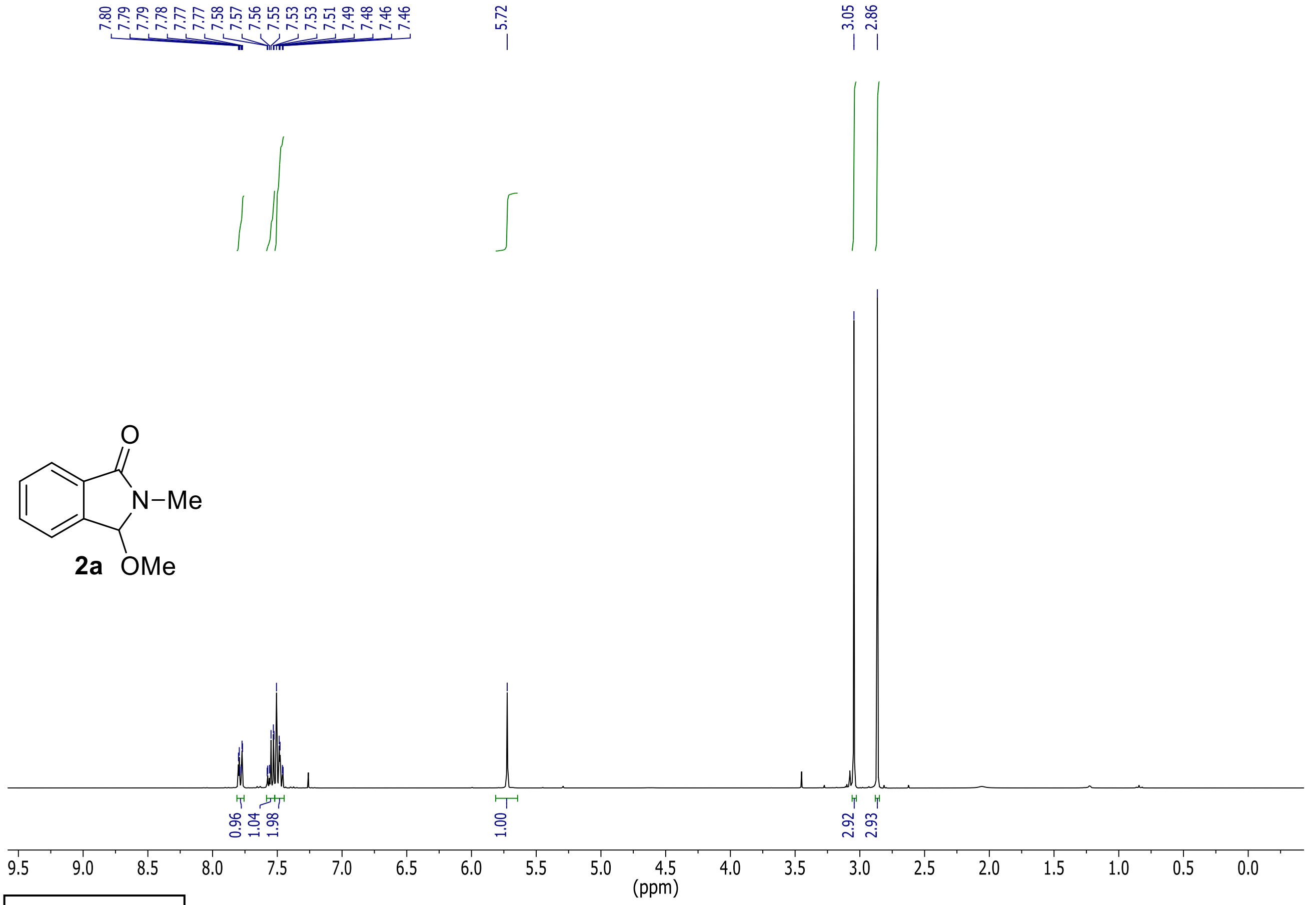

^{13}C NMR

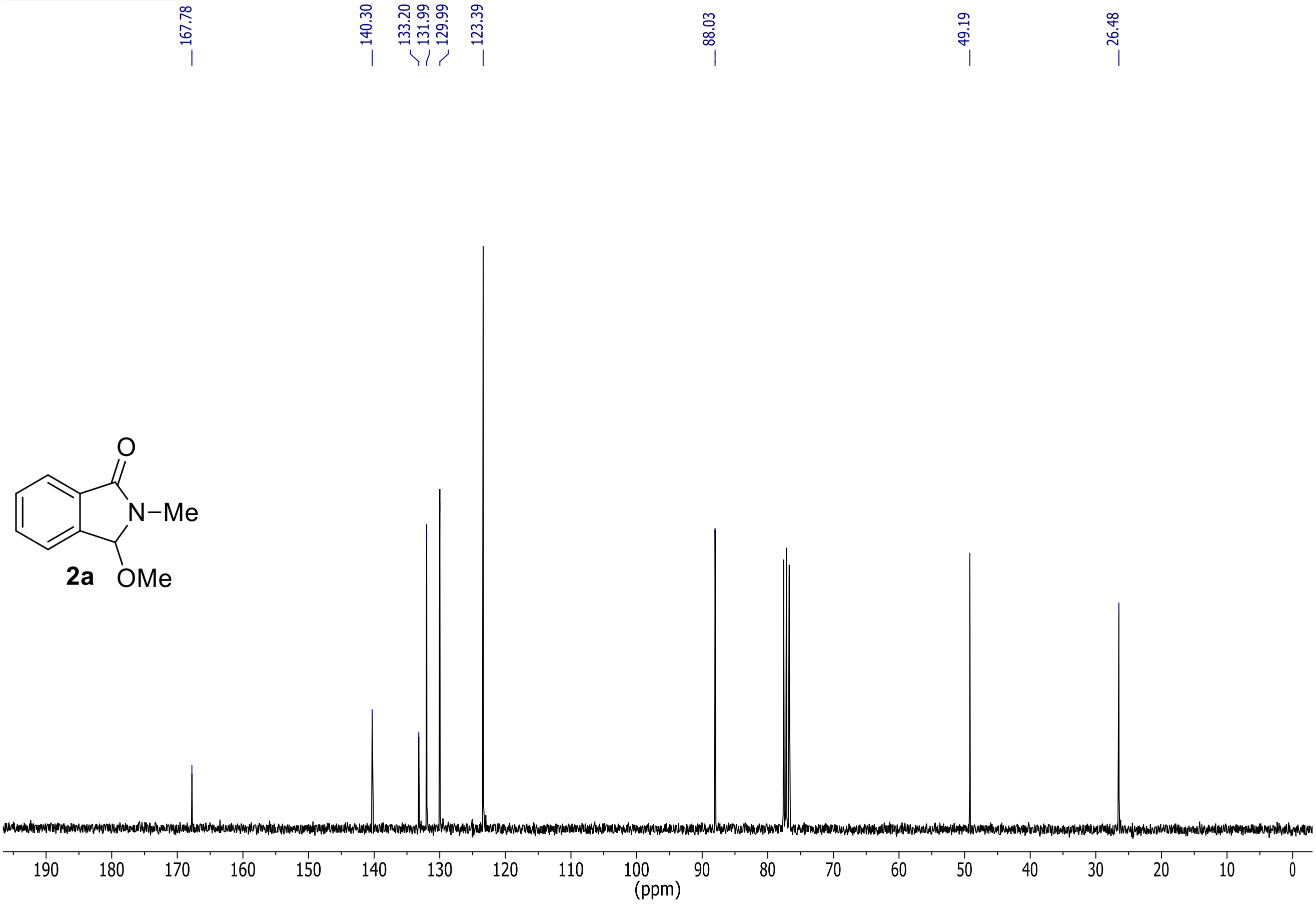

^{1}H NMR

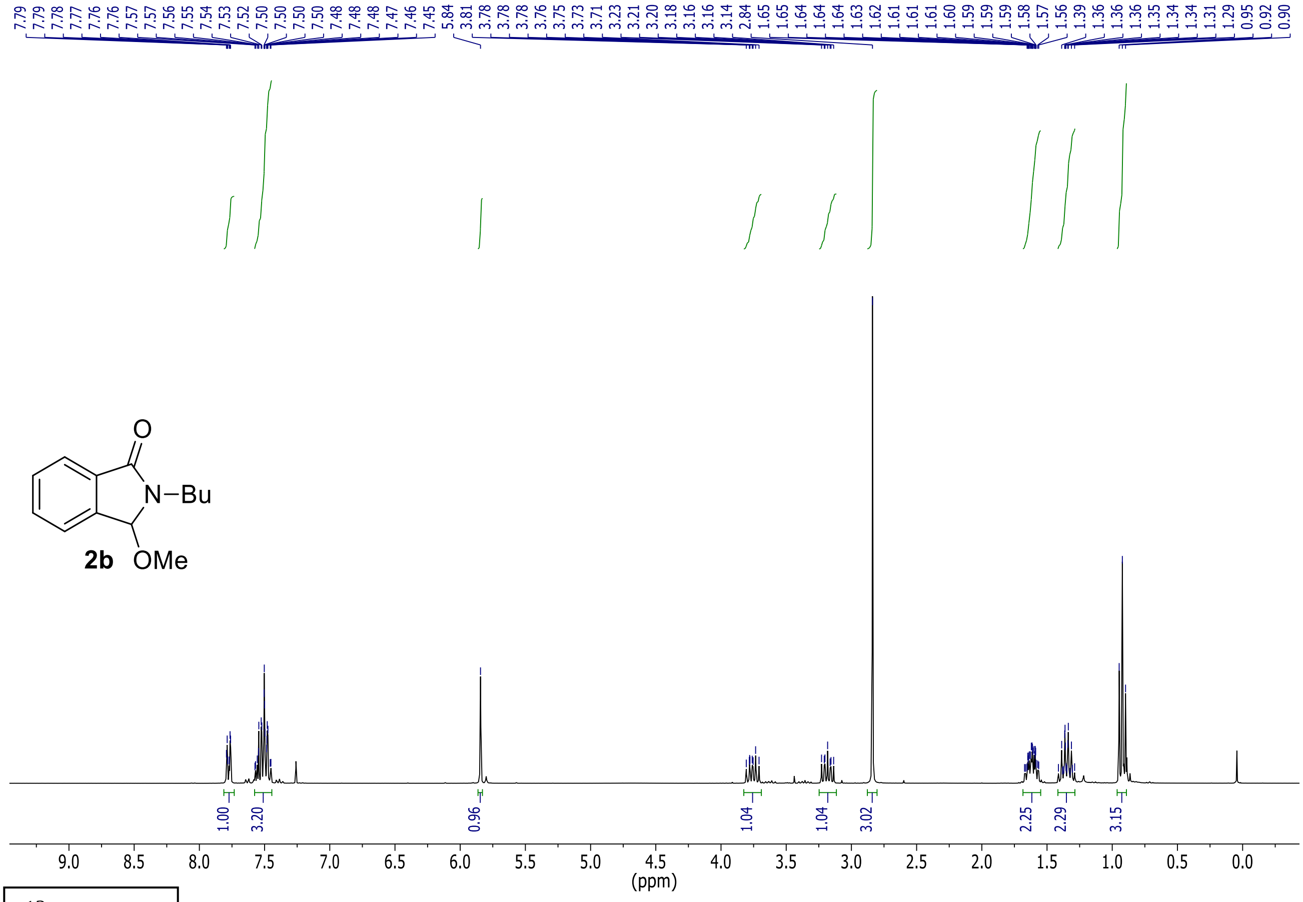

^{13}C NMR

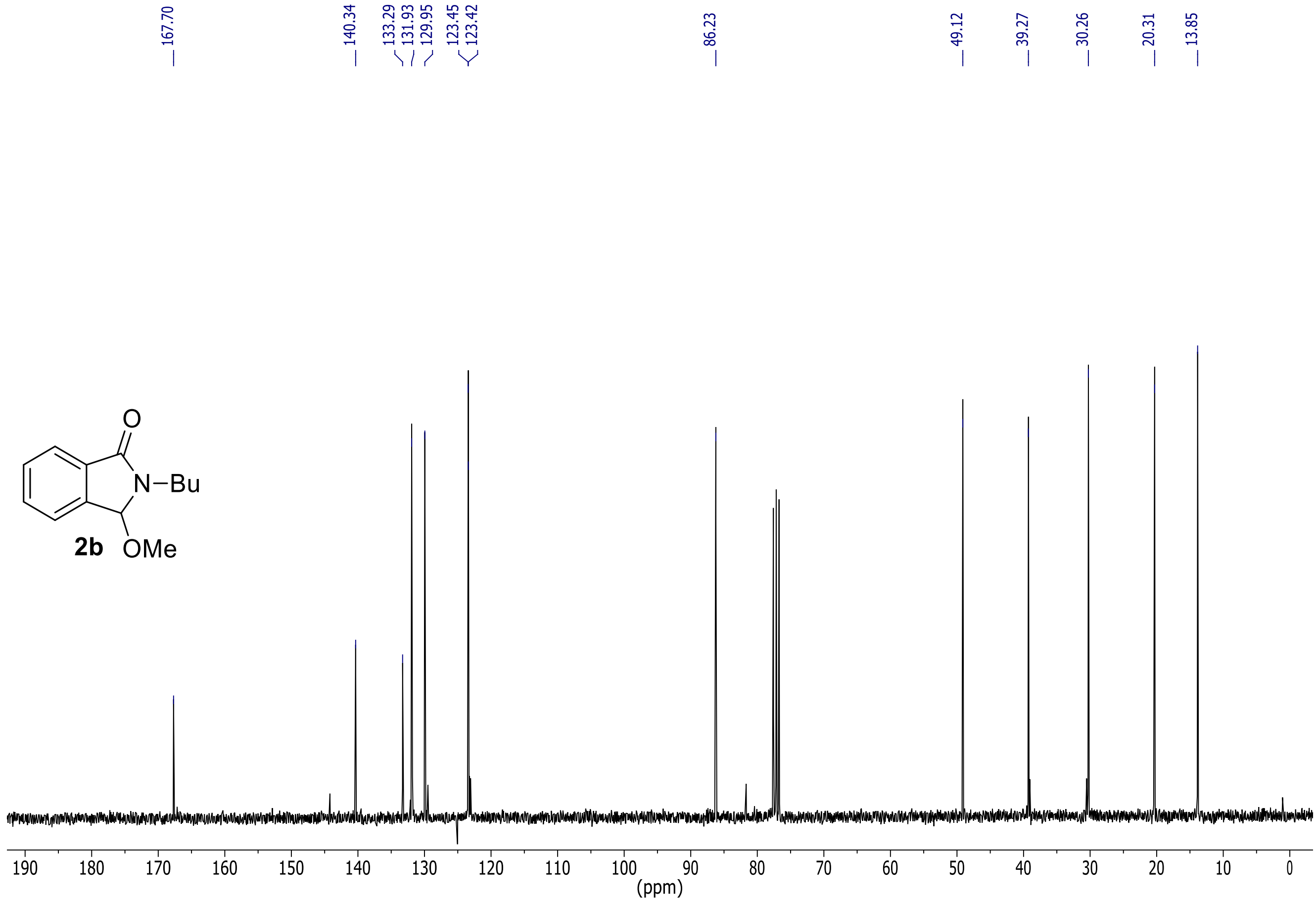

^{1}H NMR

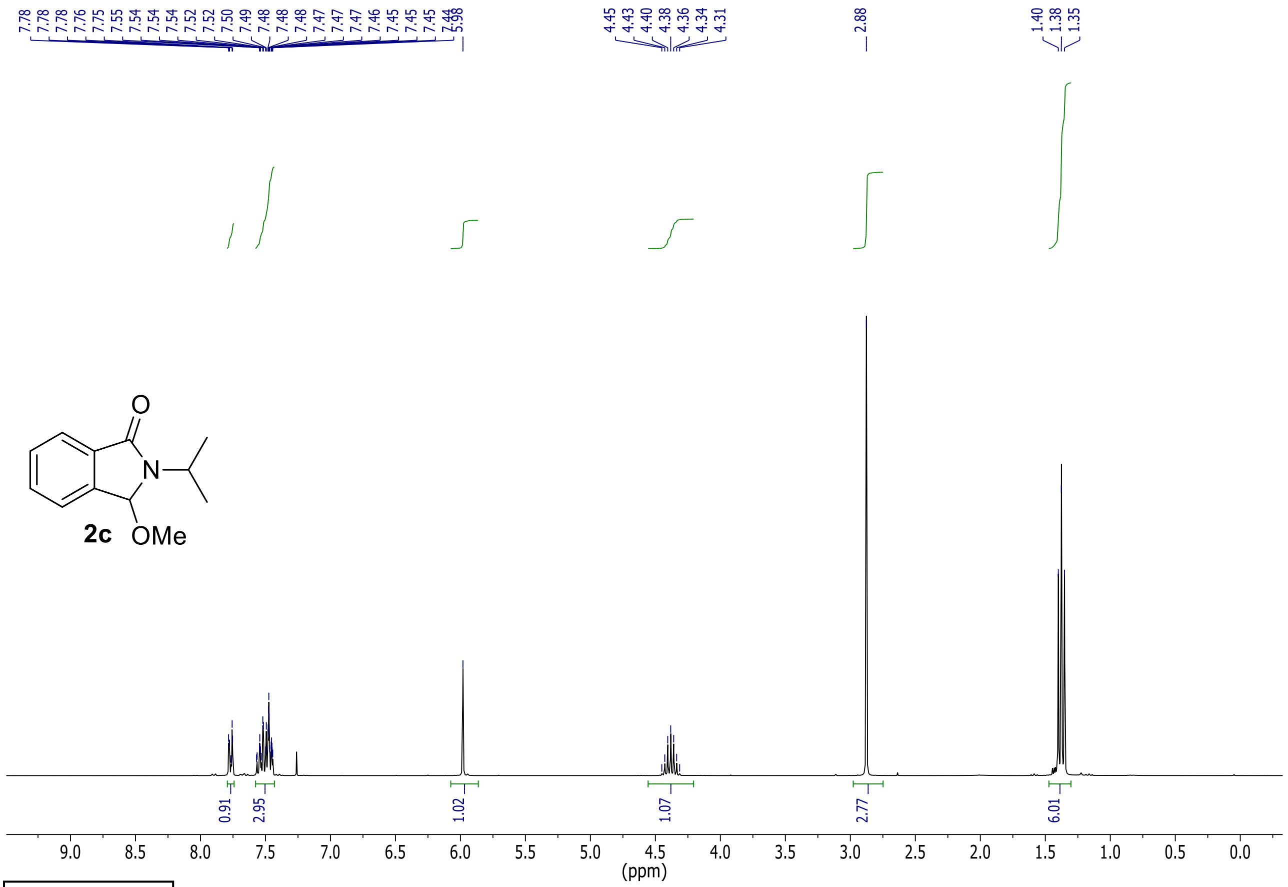

^{13}C NMR

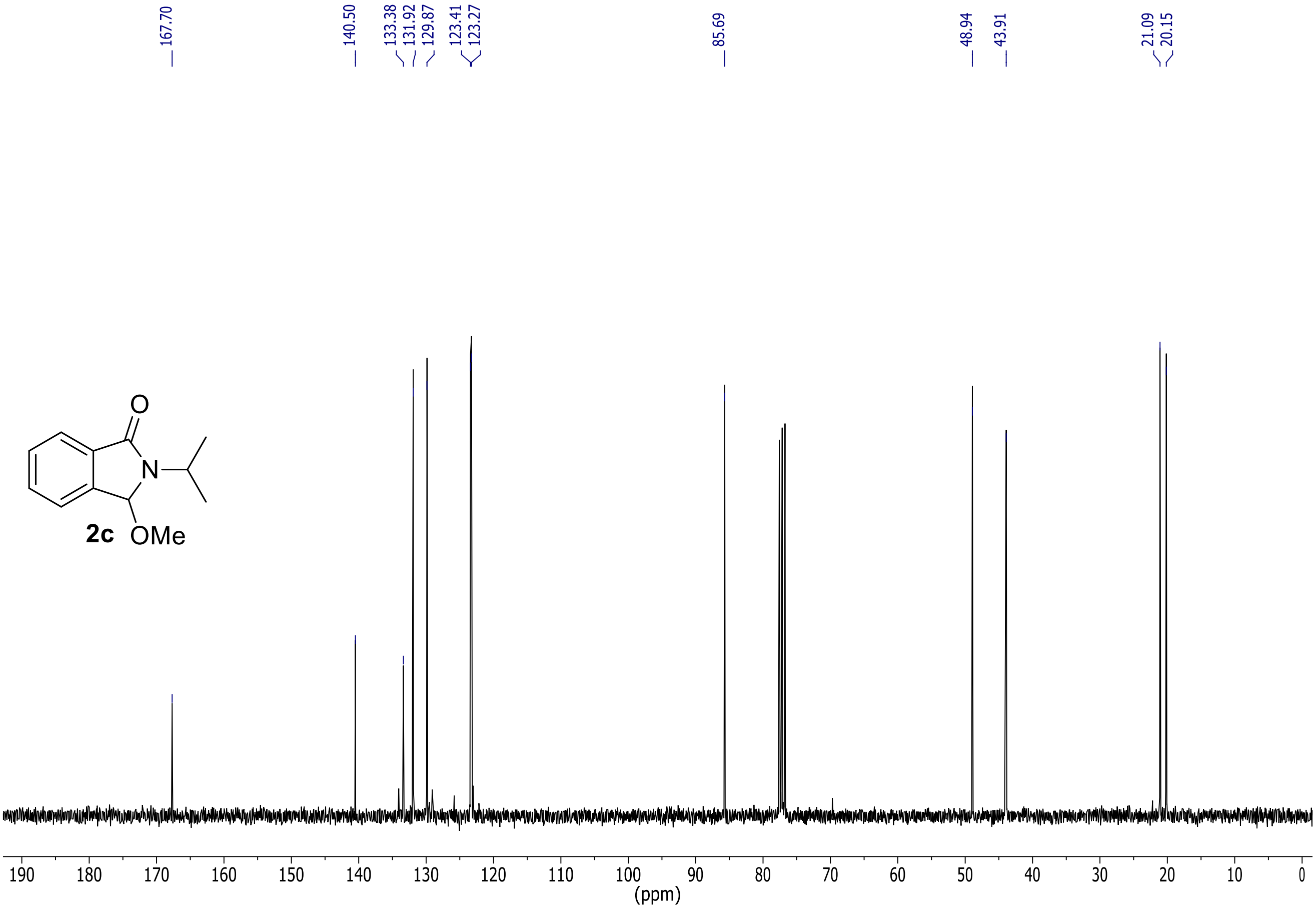

^{1}H NMR

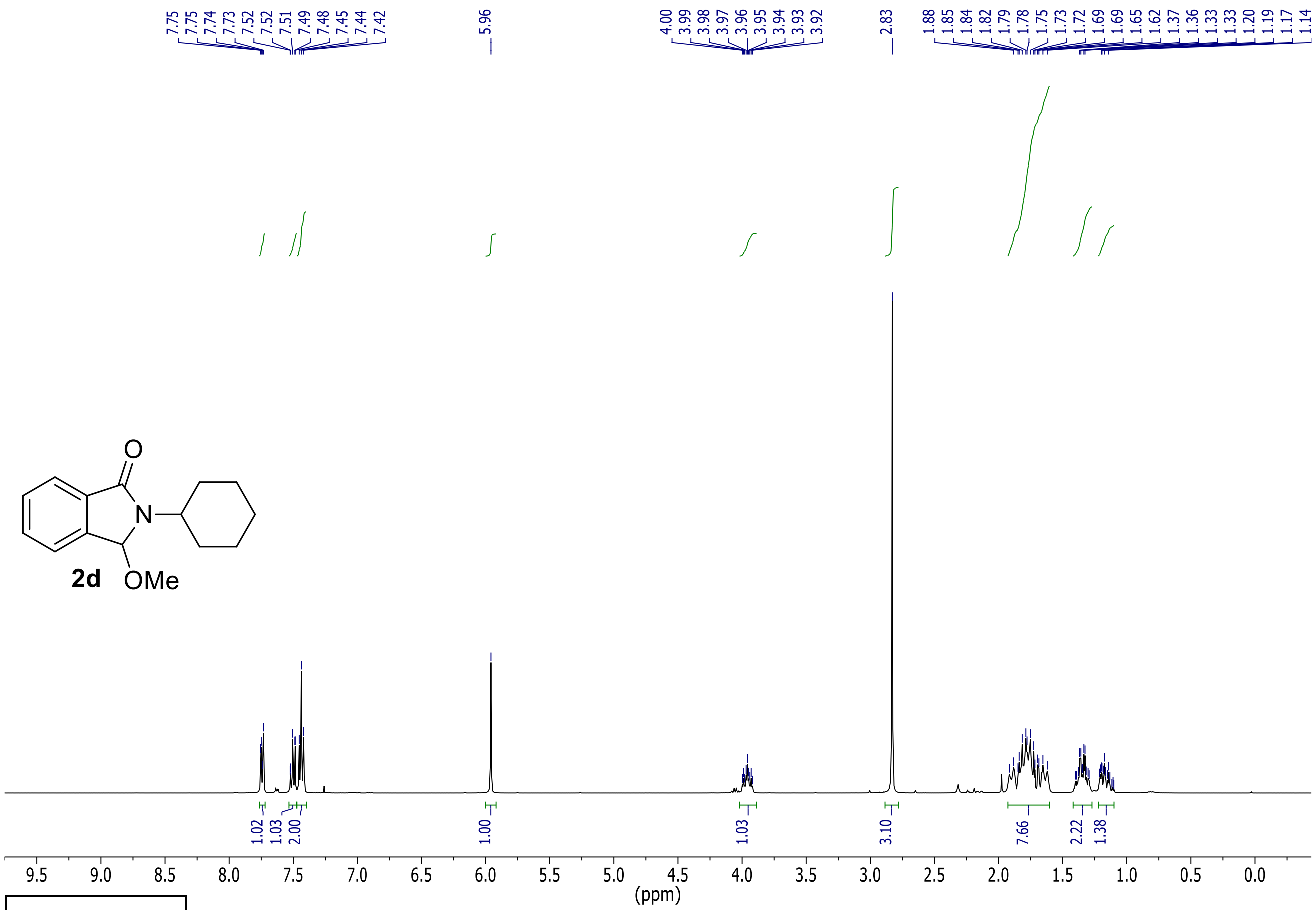

^{13}C NMR

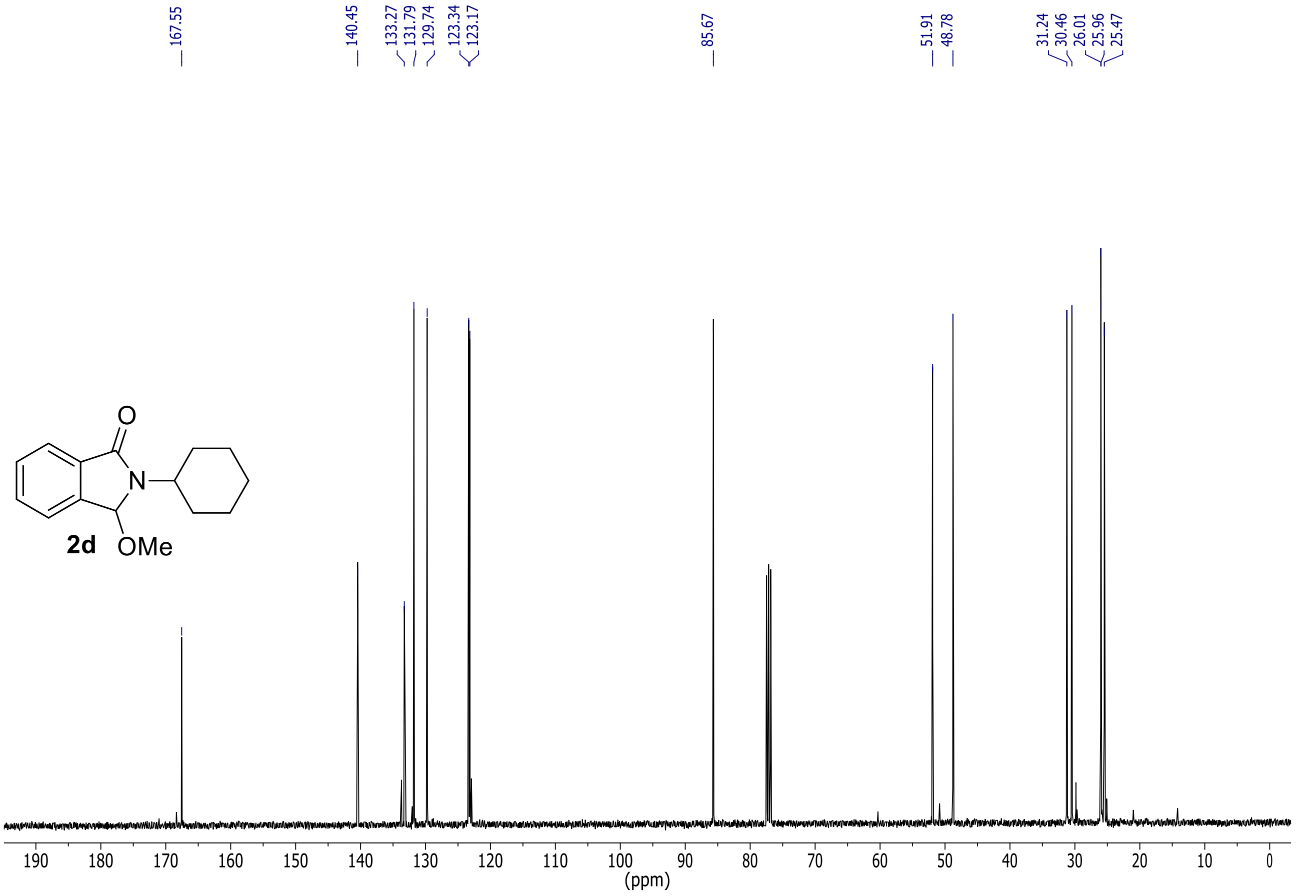

¹H NMR

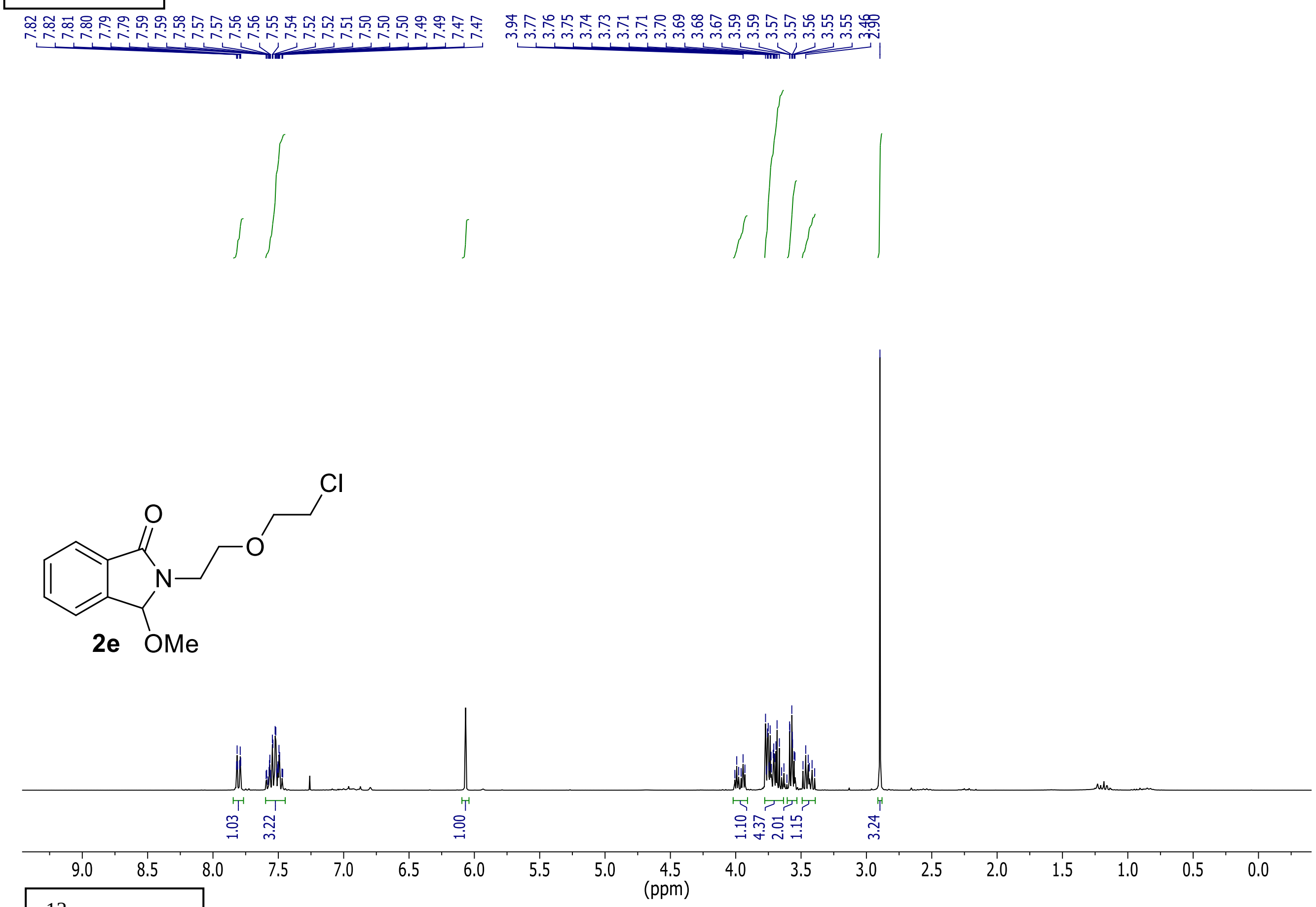

¹³C NMR

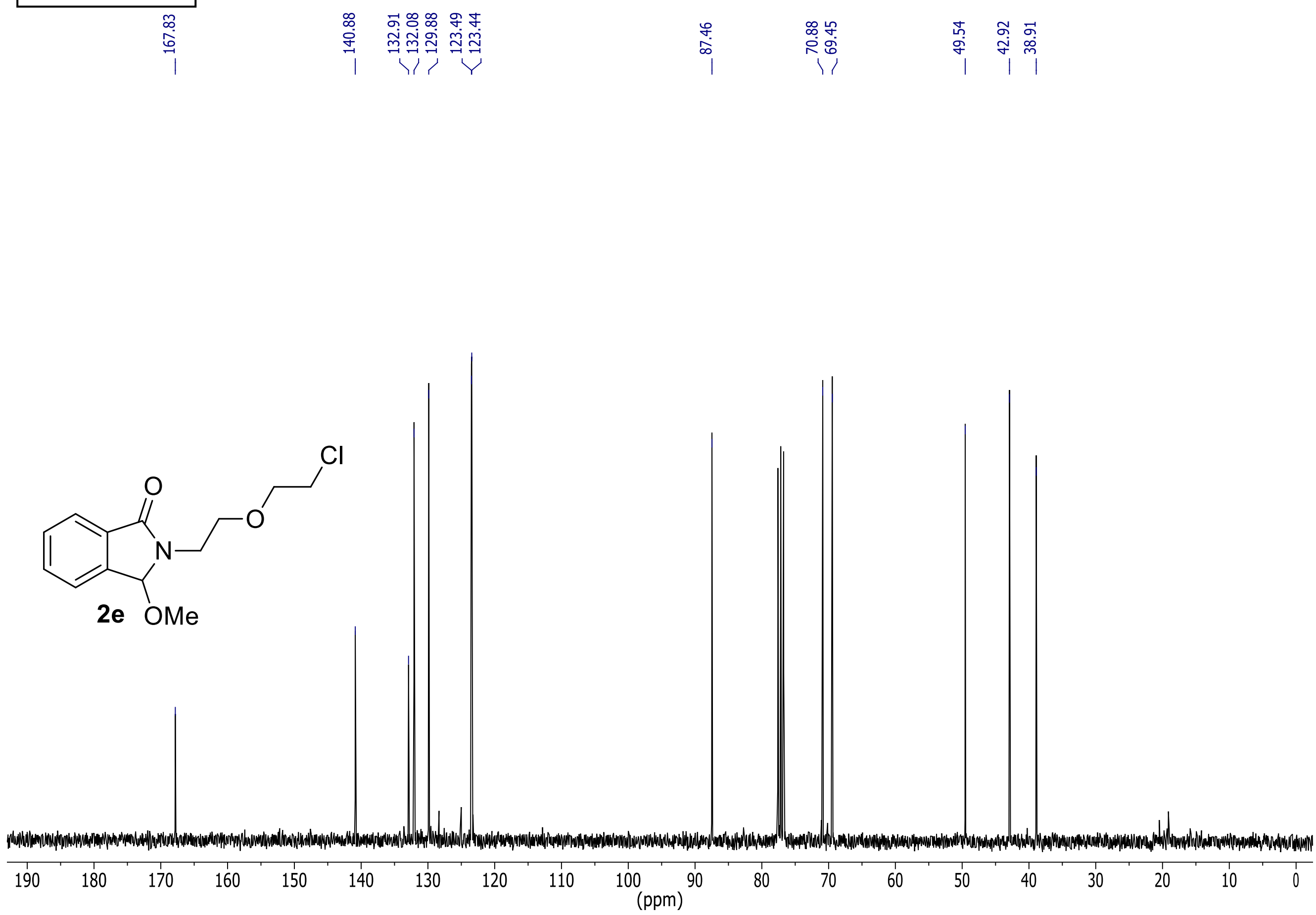

^{1}H NMR

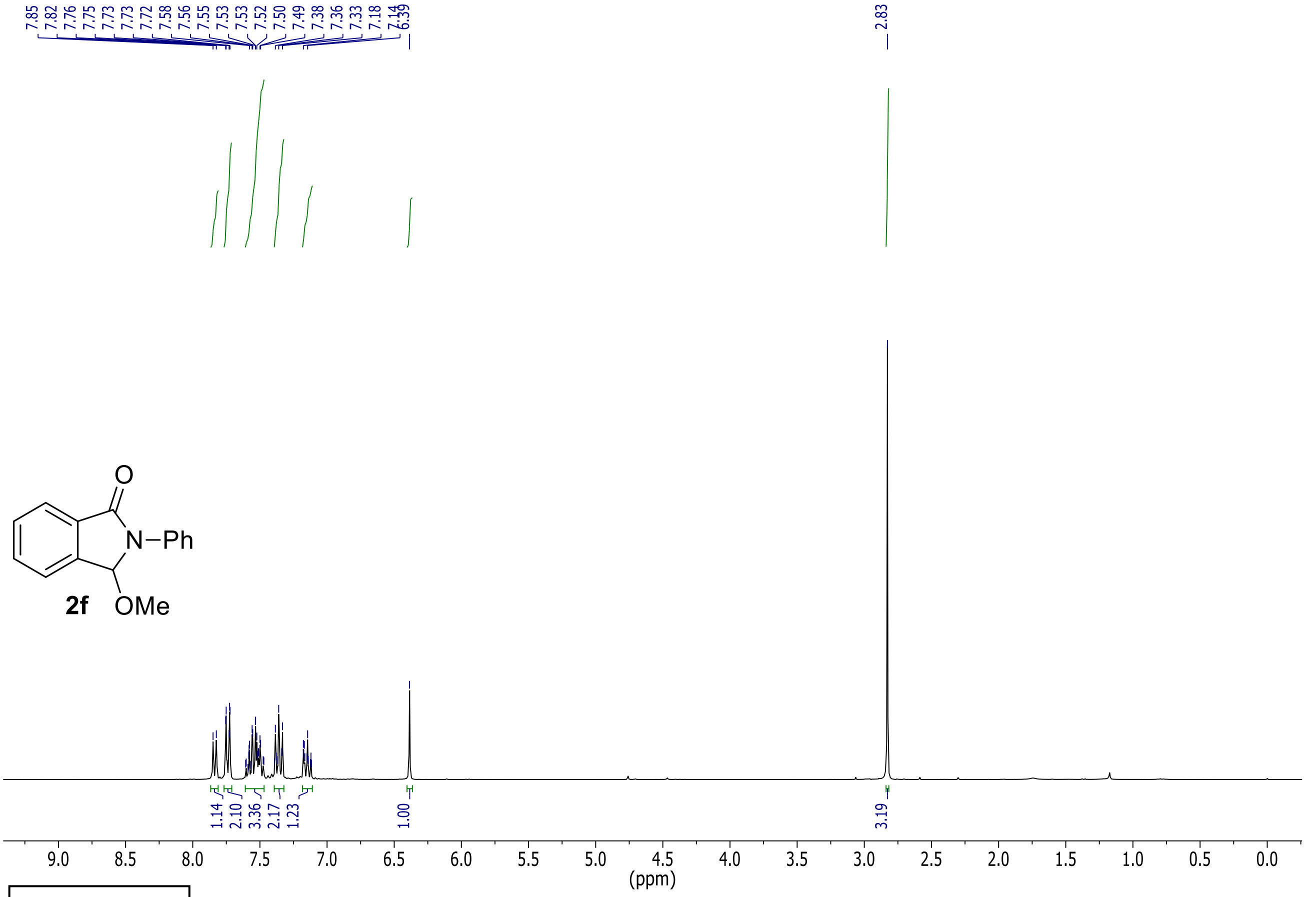

^{13}C NMR

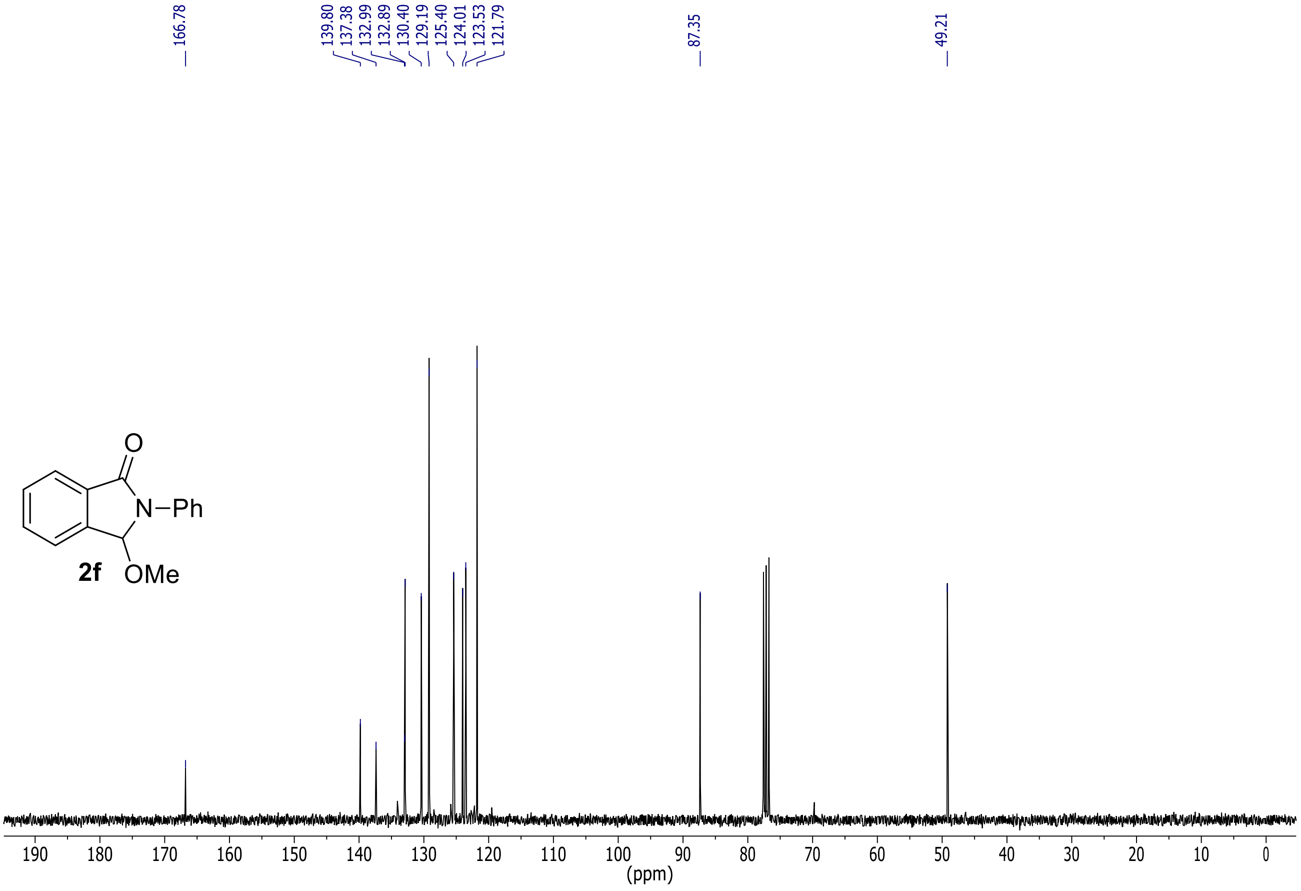

^{1}H NMR

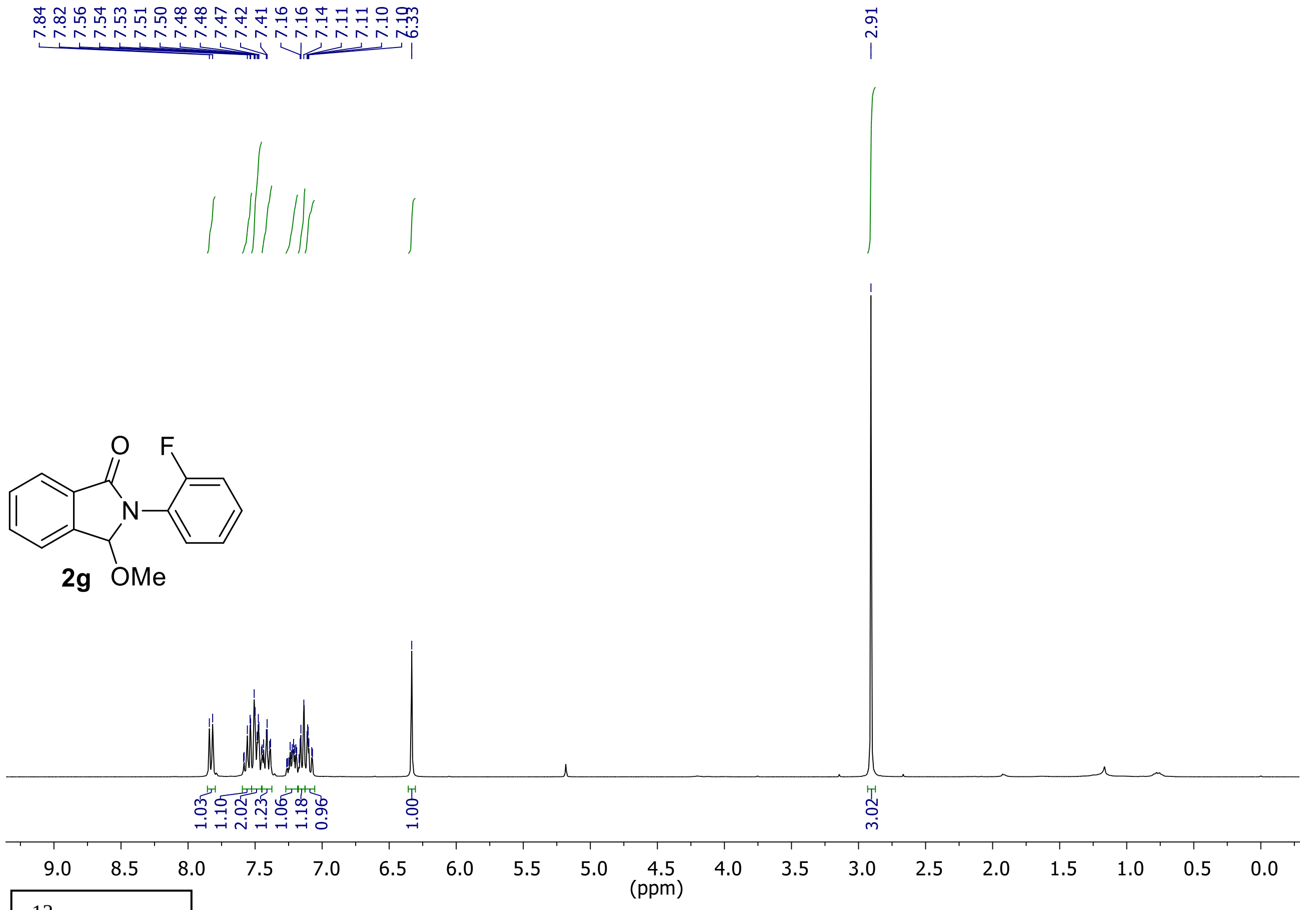

^{13}C NMR

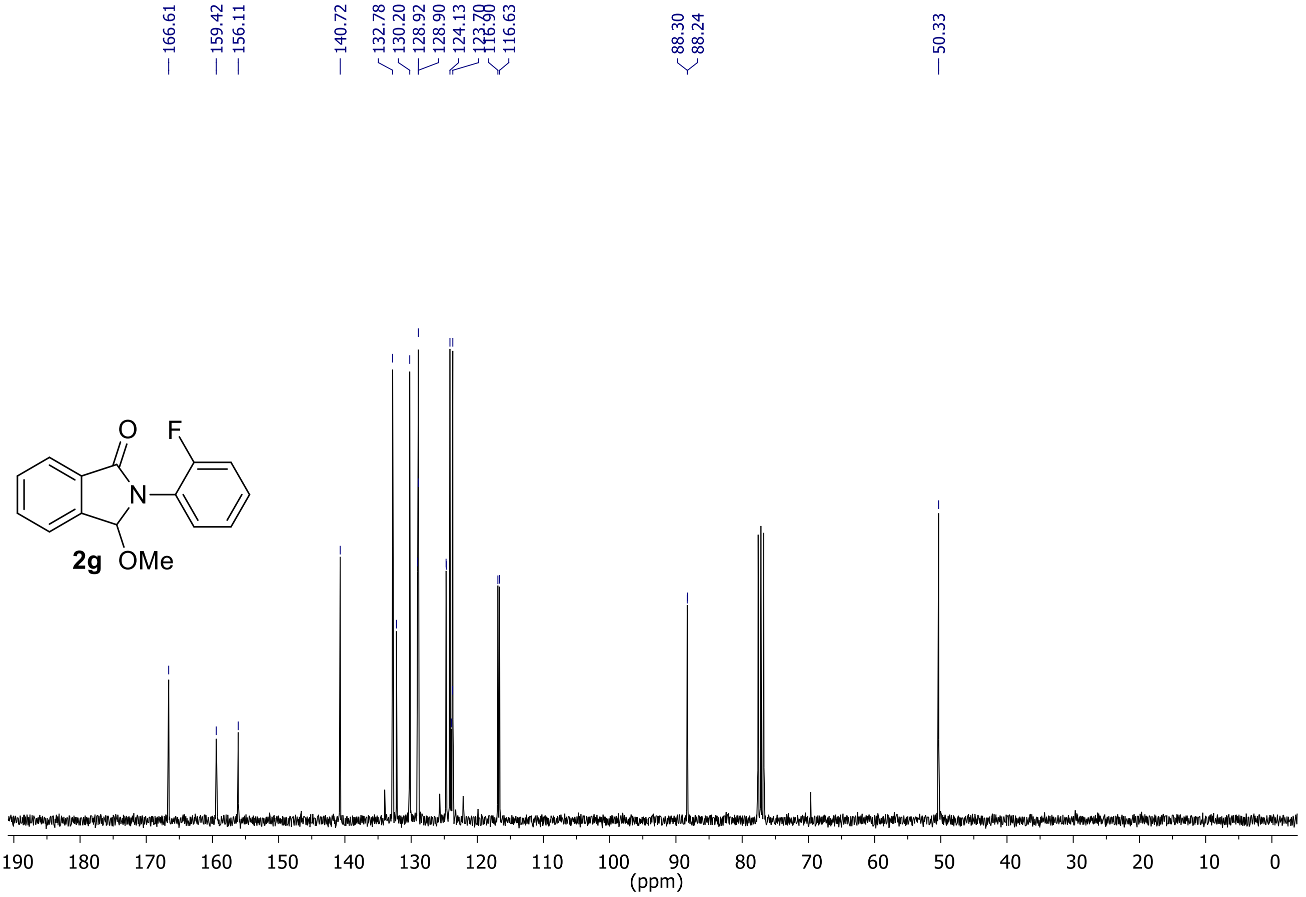

^{19}F NMR

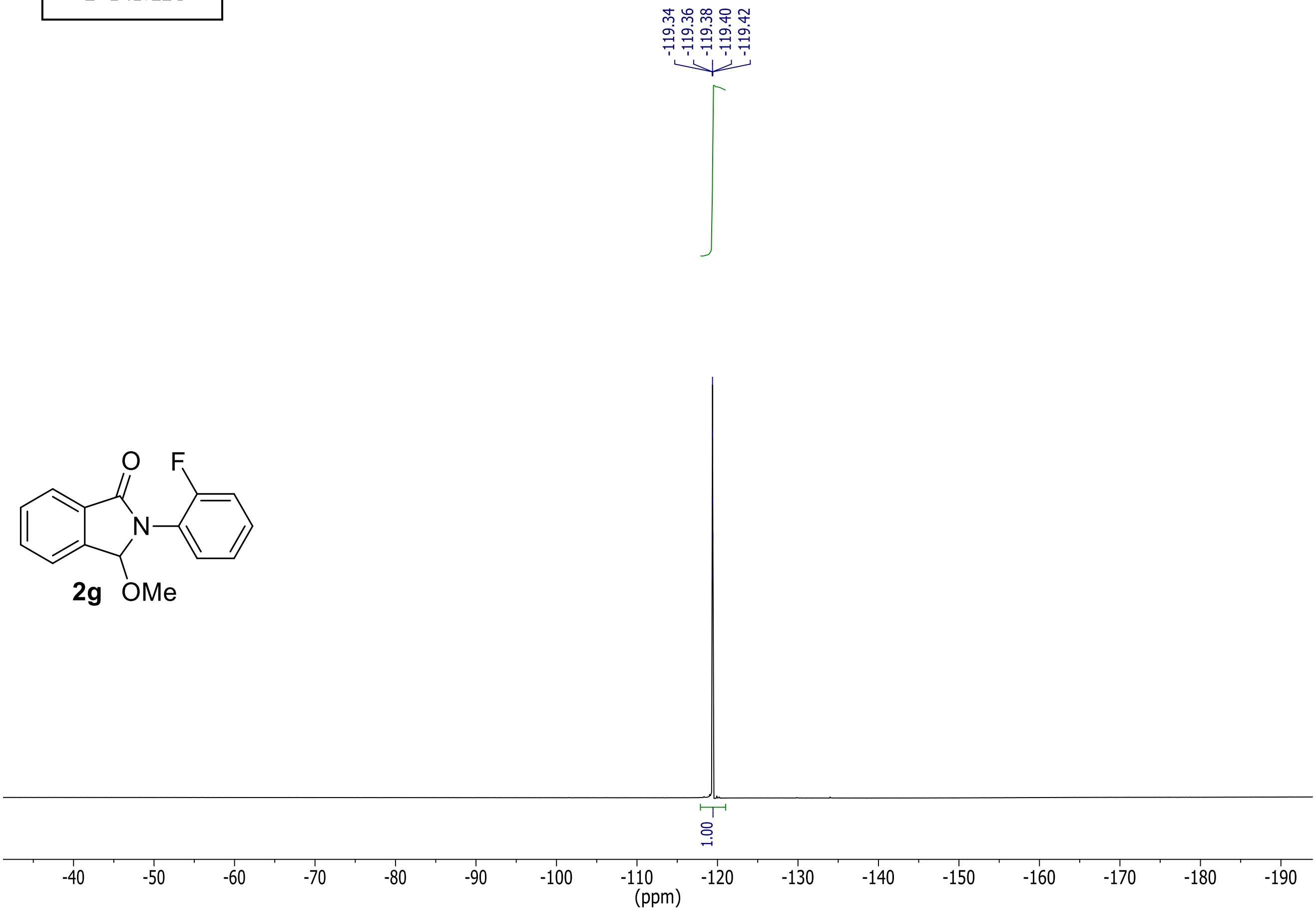
-119.34
-119.36
-119.38
-119.40
-119.42
2g
1.00
-40
-50
-60
-70
-80
-90
-100
-110
-120
-130
-140
-150
-160
-170
-180
-190
(ppm)

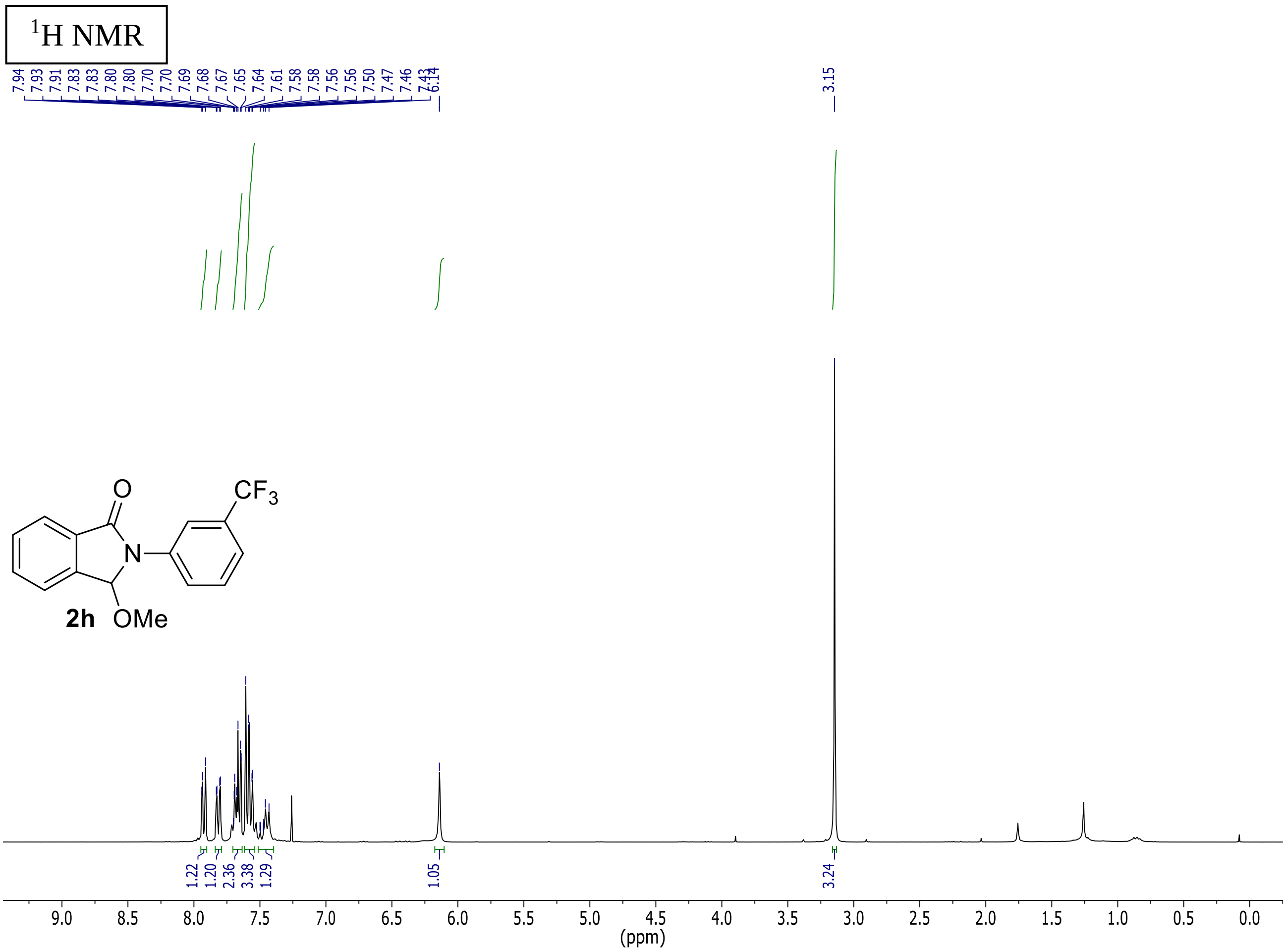

1H NMR
7.94
7.93
7.91
7.83
7.83
7.80
7.80
7.70
7.70
7.69
7.68
7.67
7.65
7.64
7.61
7.58
7.58
7.56
7.56
7.50
7.47
7.46
7.43
6.14
3.15
O
CF3
N
2h
OMe
1.22
1.20
2.36
3.38
1.29
1.05
3.24
9.0
8.5
8.0
7.5
7.0
6.5
6.0
5.5
5.0
4.5
4.0
3.5
3.0
2.5
2.0
1.5
1.0
0.5
0.0
(ppm)

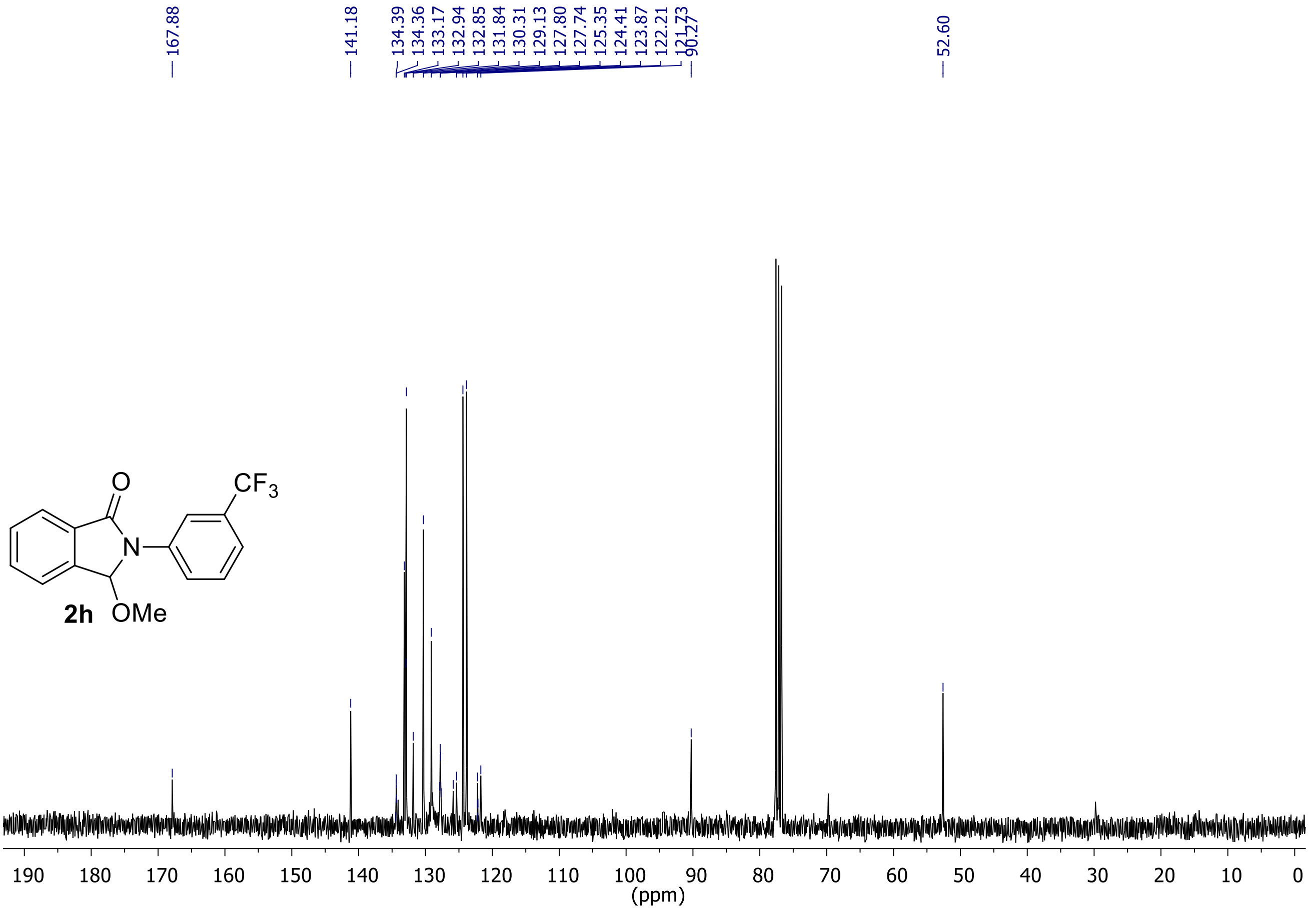

167.88
141.18
134.39
134.36
133.17
132.94
132.85
131.84
130.31
129.13
127.80
127.74
125.35
124.41
123.87
122.21
121.73
90.27
52.60
O
CF3
N
2h
OMe
190
180
170
160
150
140
130
120
110
100
90
80
70
60
50
40
30
20
10
0
(ppm)

^{1}H NMR

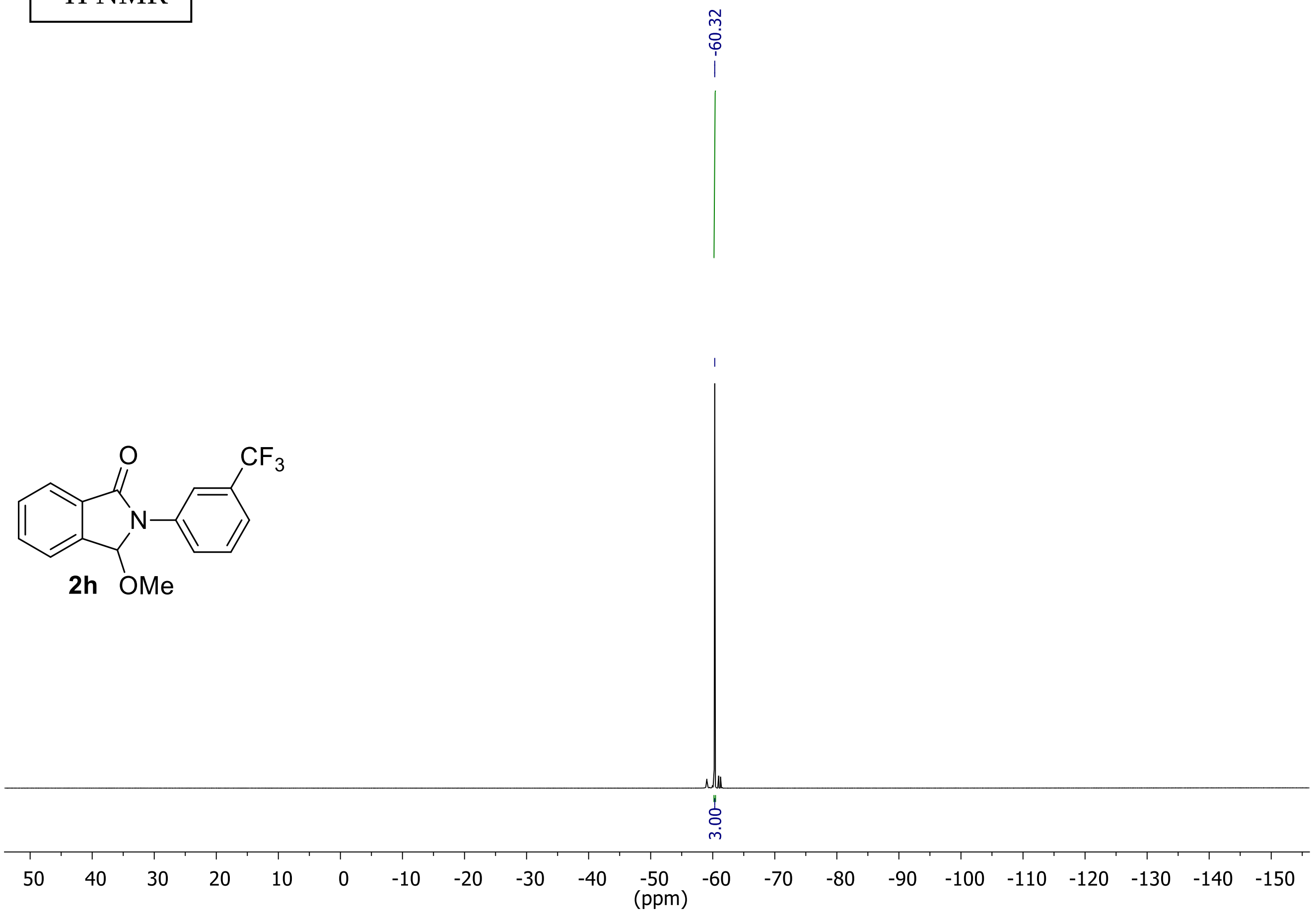
2h
-60.32
3.00
50 40 30 20 10 0 -10 -20 -30 -40 -50 -60 -70 -80 -90 -100 -110 -120 -130 -140 -150
(ppm)

^{1}H NMR

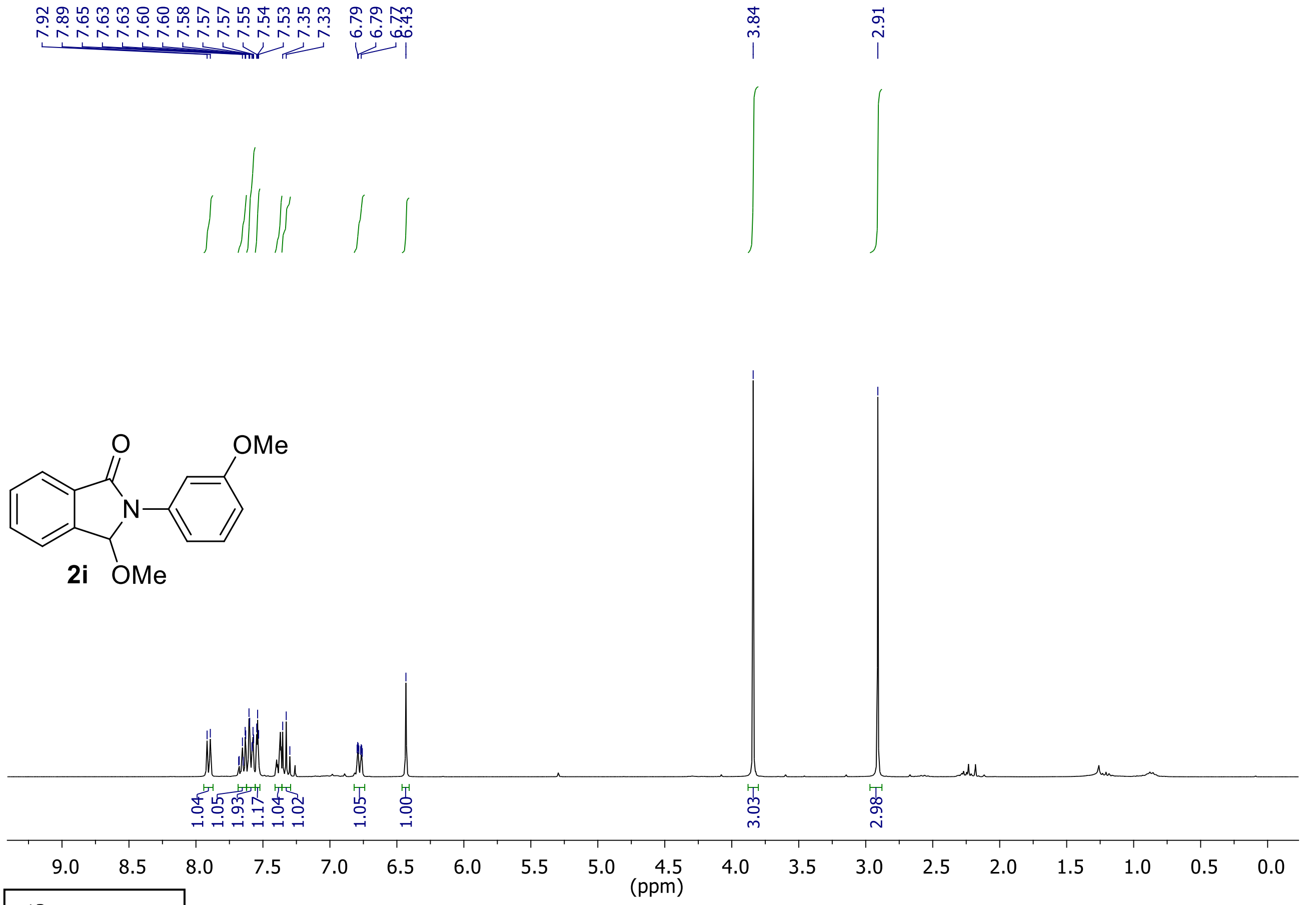

^{13}C NMR

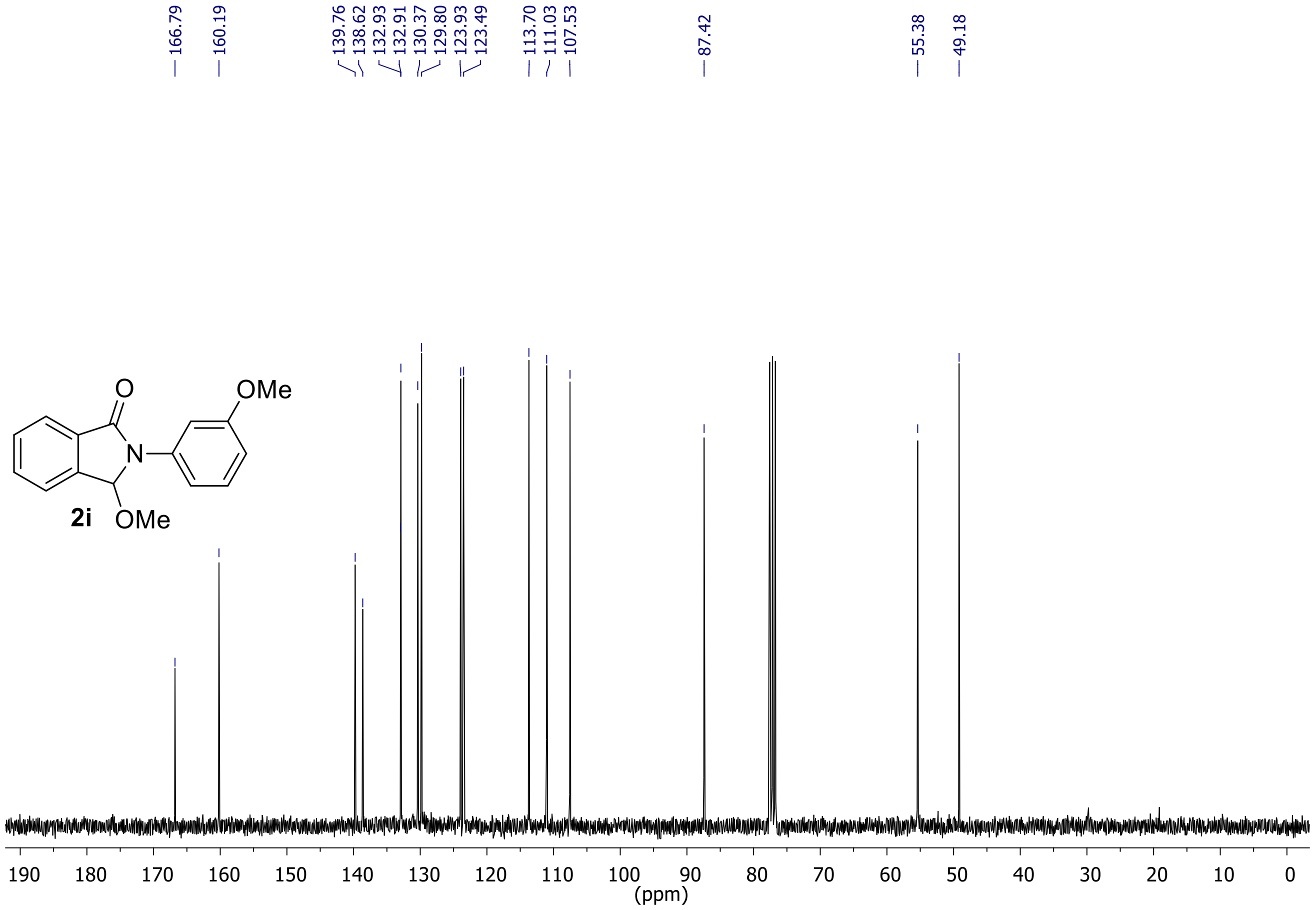

^{1}H NMR

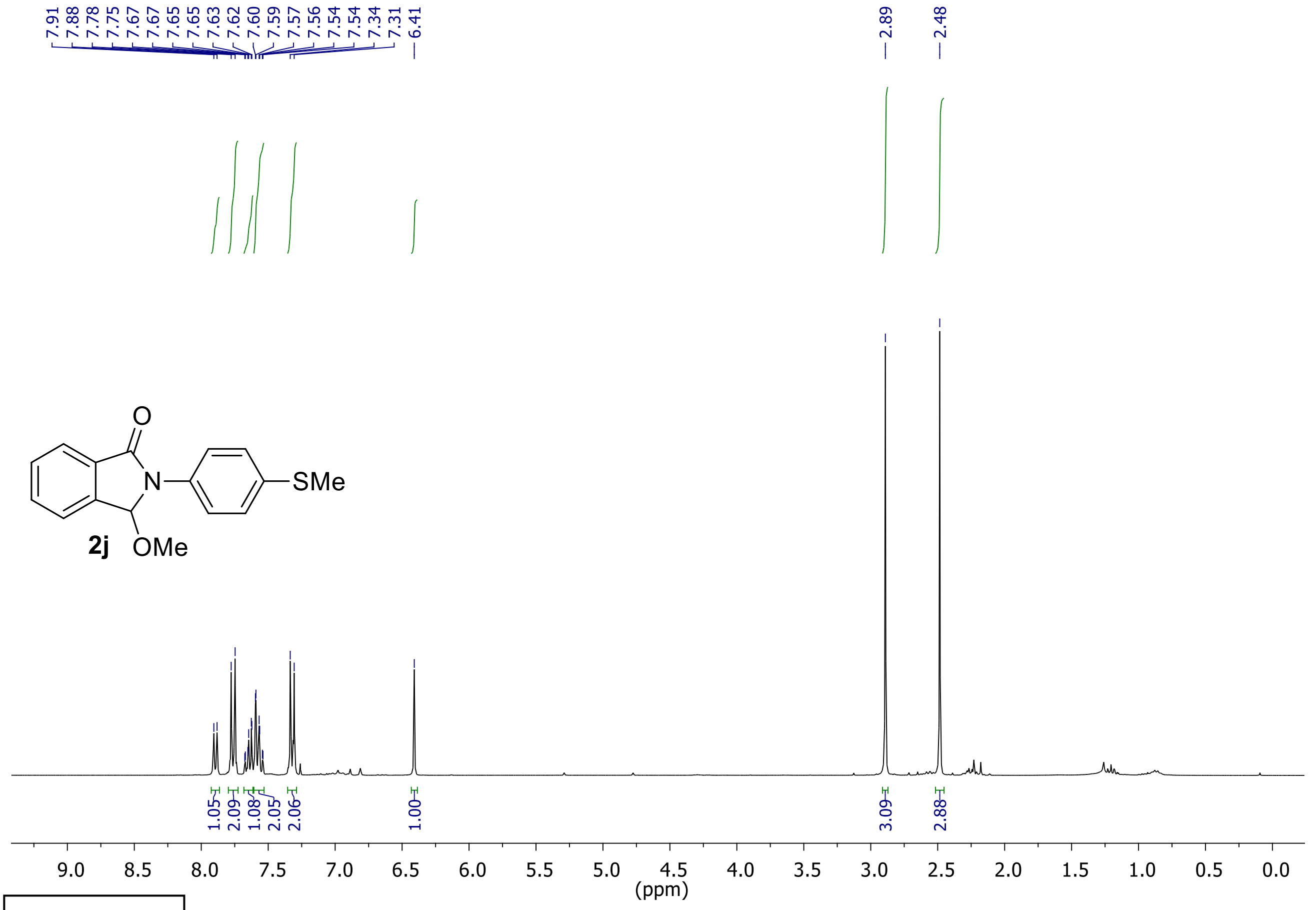

^{13}C NMR

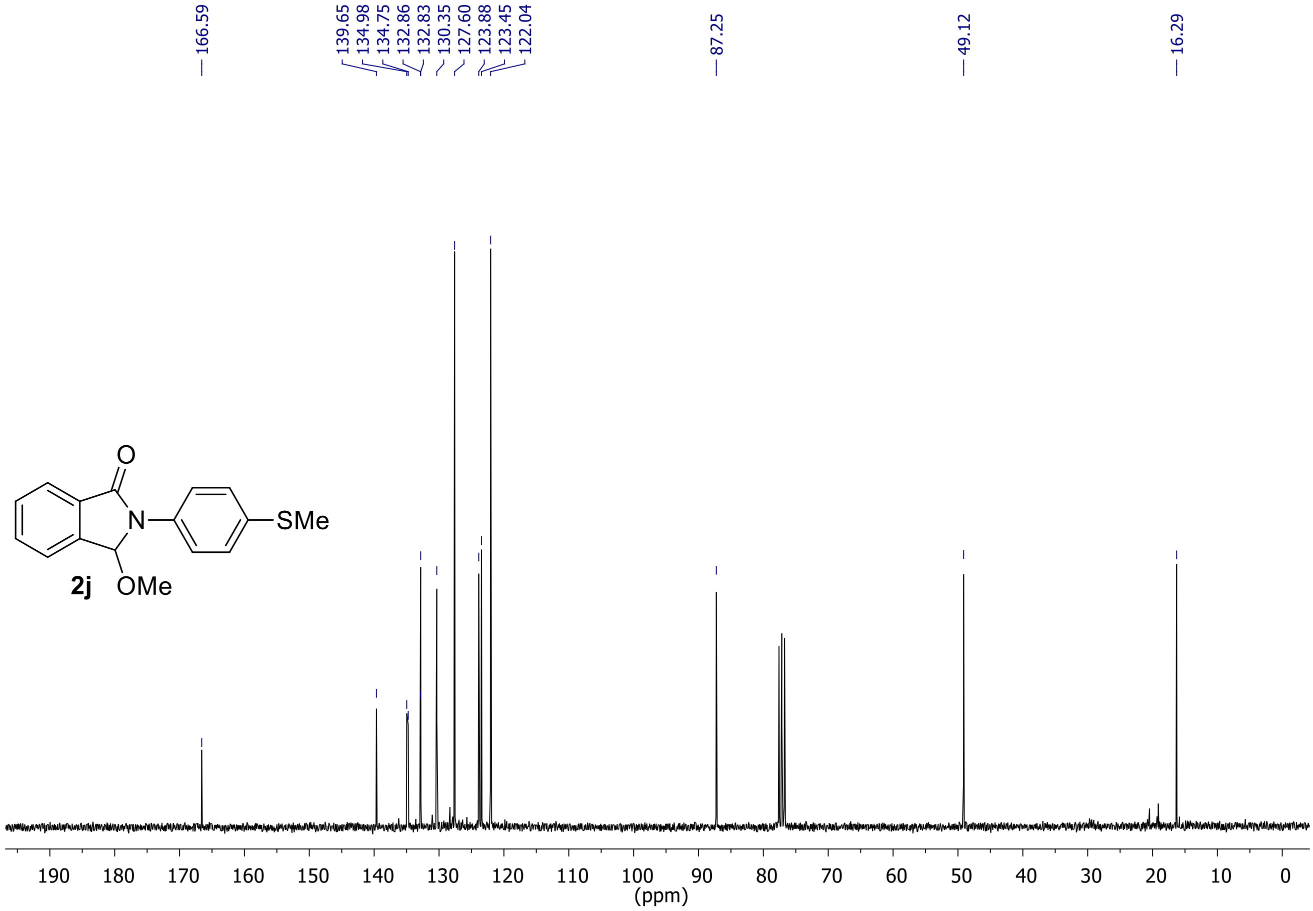

^{1}H NMR

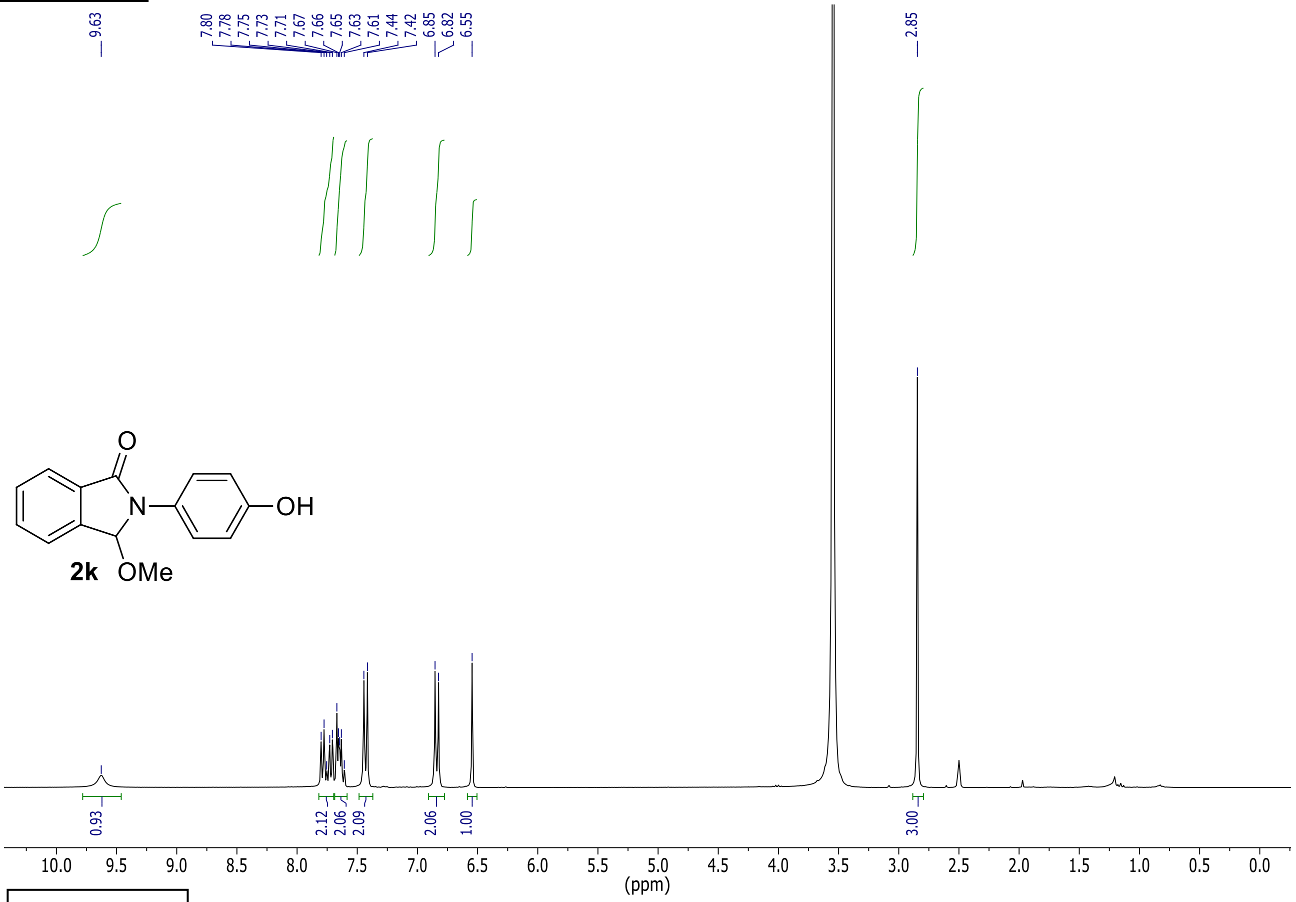

^{13}C NMR

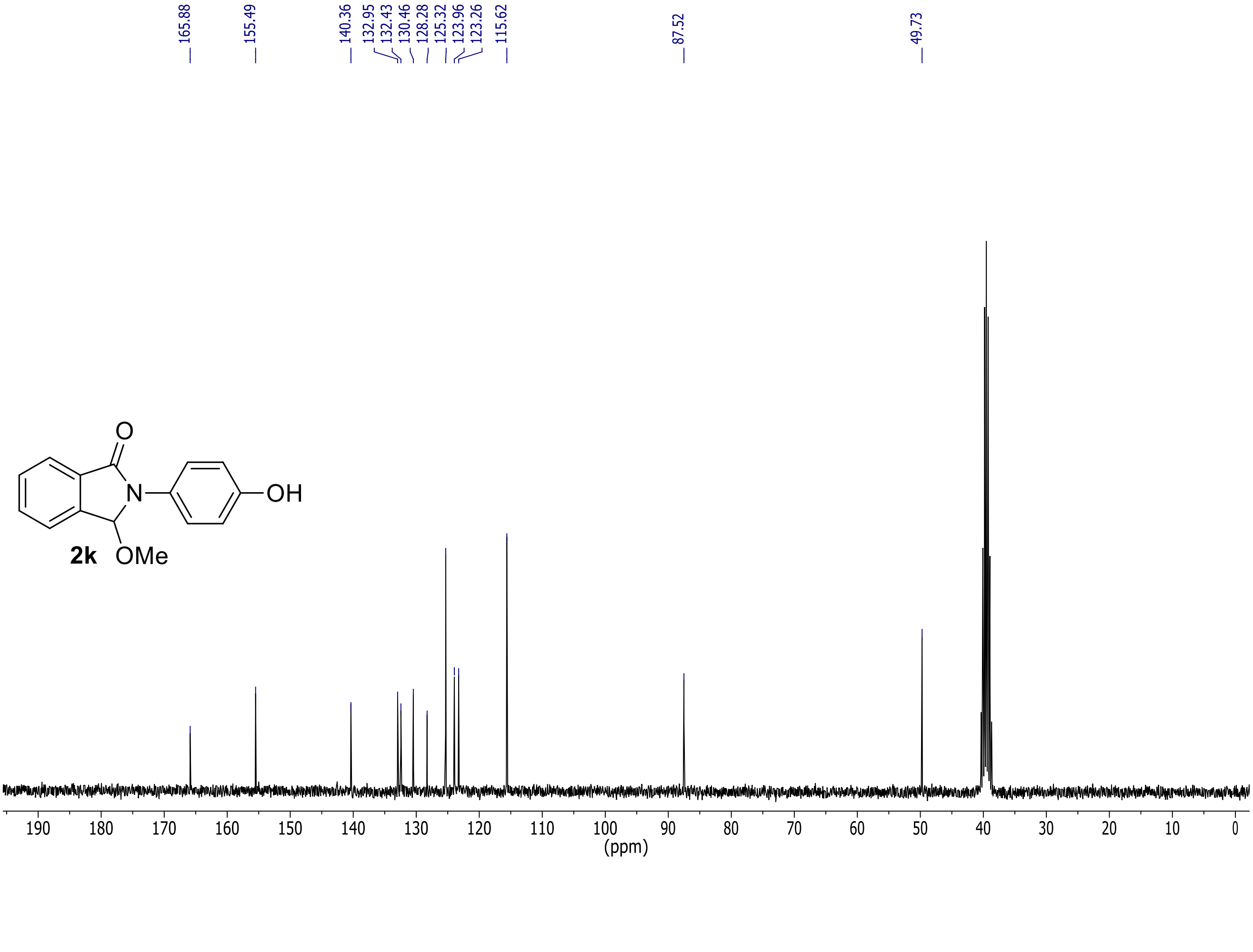

^{1}H NMR

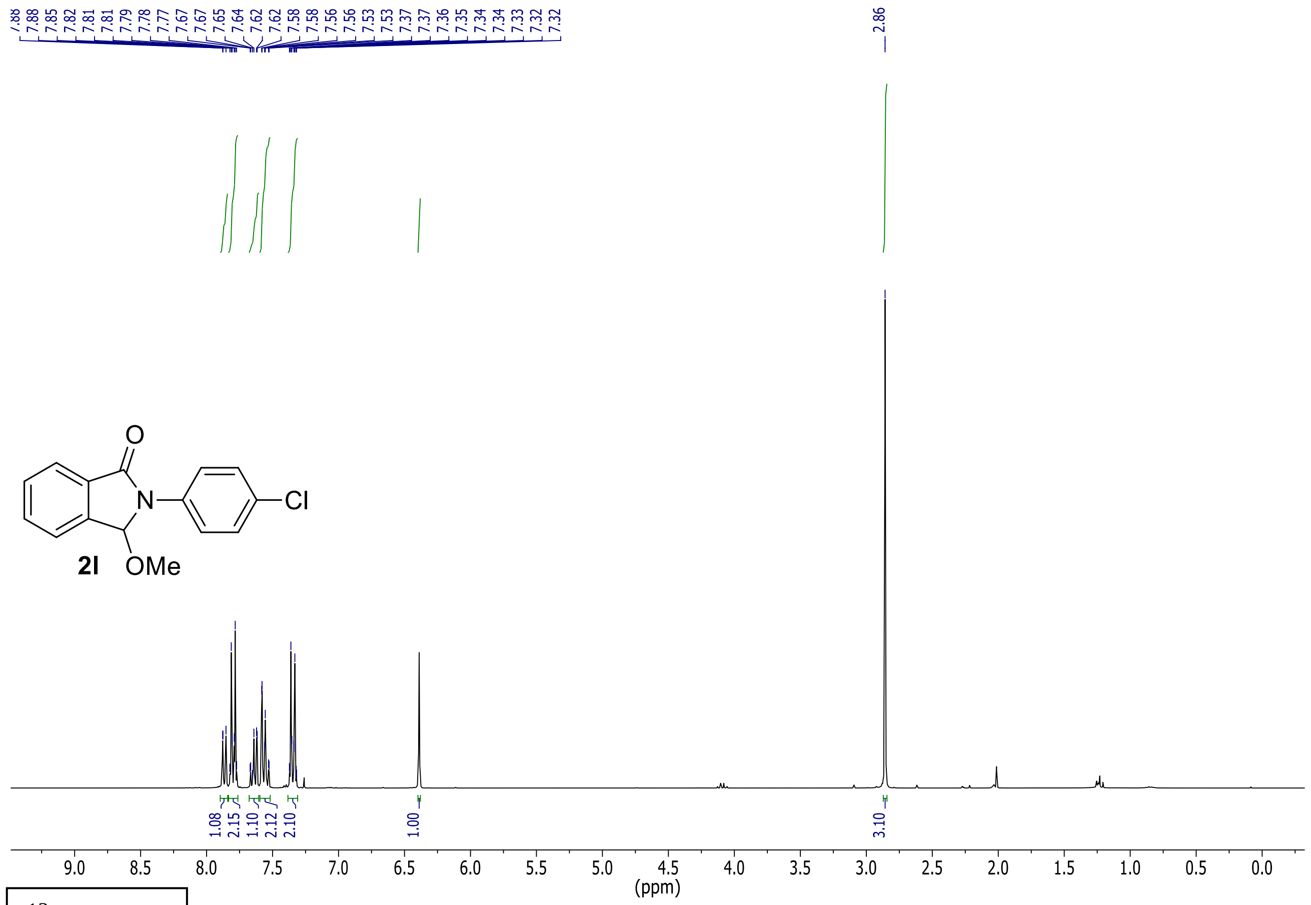

^{13}C NMR

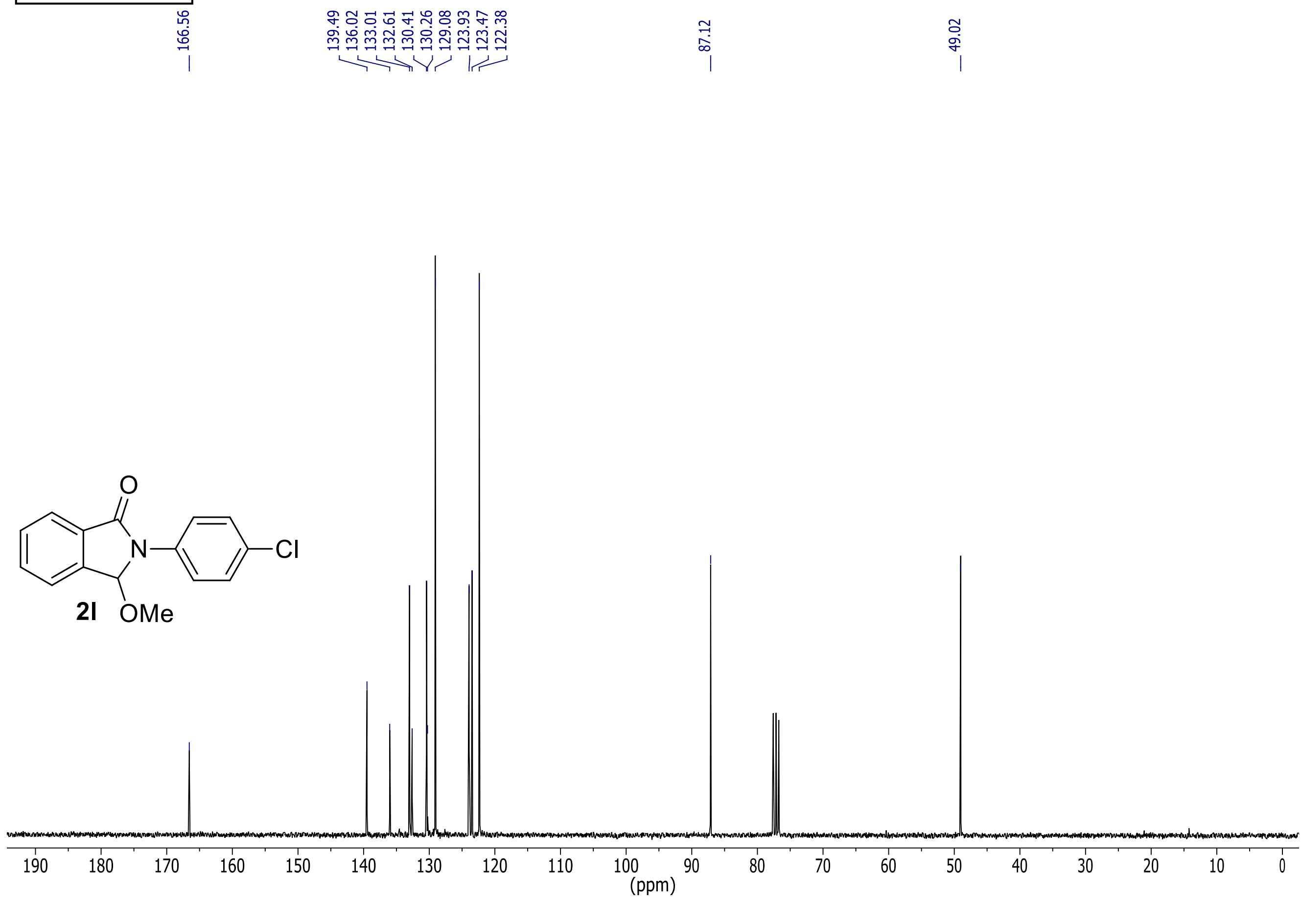

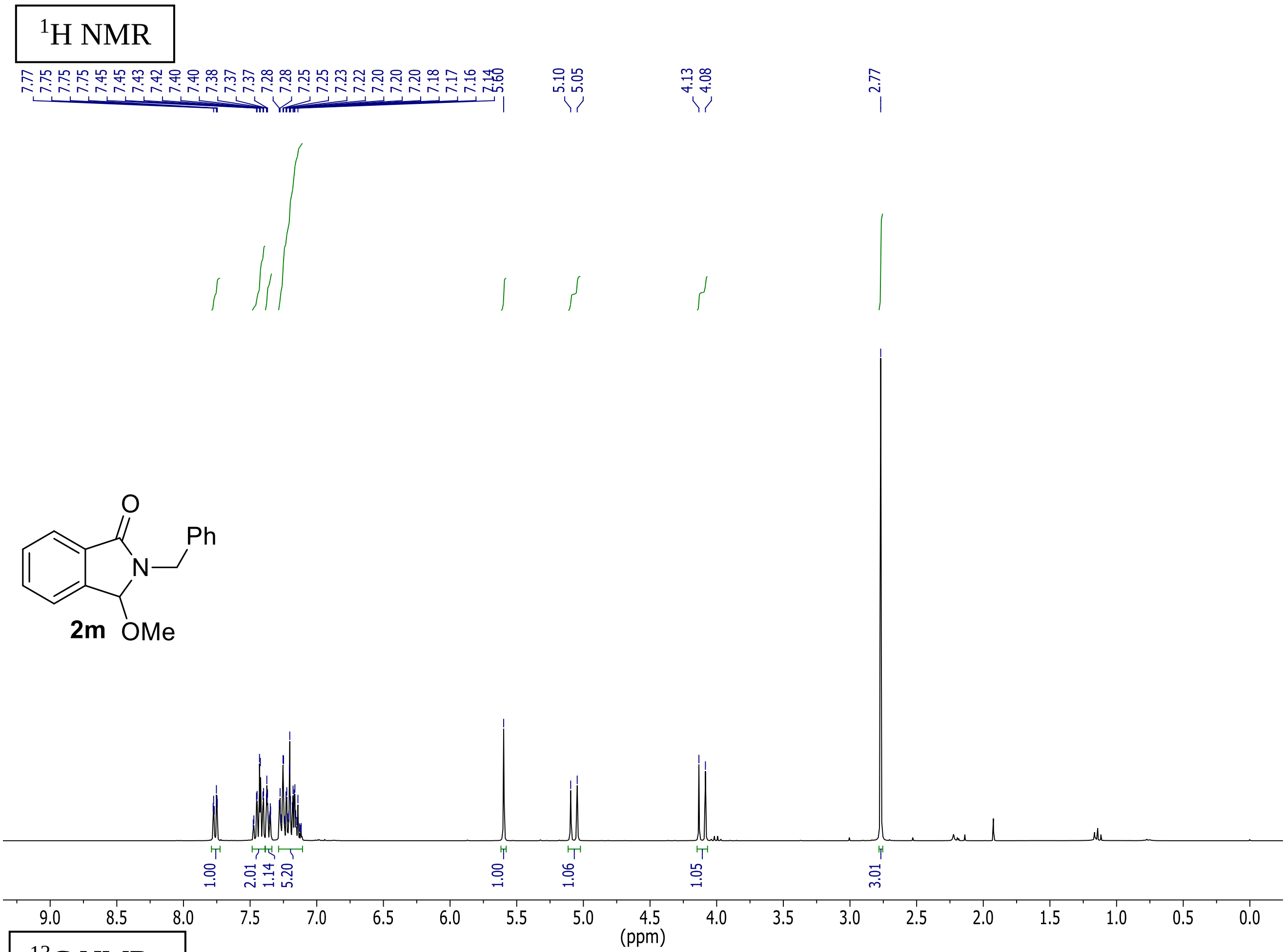

1H NMR
7.77
7.75
7.75
7.75
7.45
7.45
7.43
7.42
7.40
7.40
7.38
7.37
7.37
7.28
7.28
7.25
7.25
7.23
7.22
7.20
7.20
7.20
7.18
7.17
7.16
7.14
5.60
5.10
5.05
4.13
4.08
2.77
O
Ph
N
2m
OMe
1.00
2.01
1.14
5.20
1.00
1.06
1.05
3.01
9.0
8.5
8.0
7.5
7.0
6.5
6.0
5.5
5.0
4.5
4.0
3.5
3.0
2.5
2.0
1.5
1.0
0.5
0.0
(ppm)

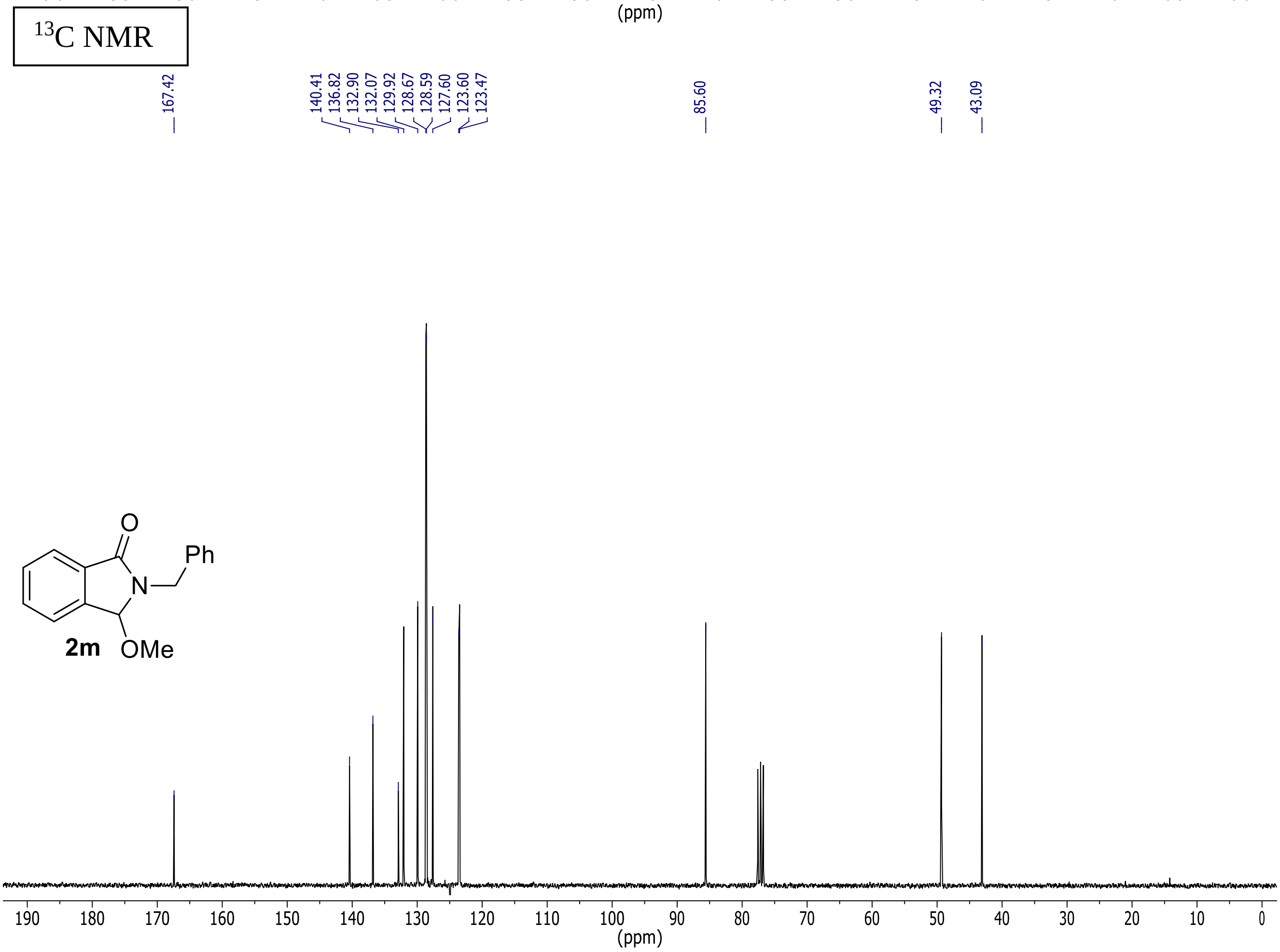

13C NMR
167.42
140.41
136.82
132.90
132.07
129.92
128.67
128.59
127.60
123.60
123.47
85.60
49.32
43.09
O
Ph
N
2m
OMe
190
180
170
160
150
140
130
120
110
100
90
80
70
60
50
40
30
20
10
0
(ppm)

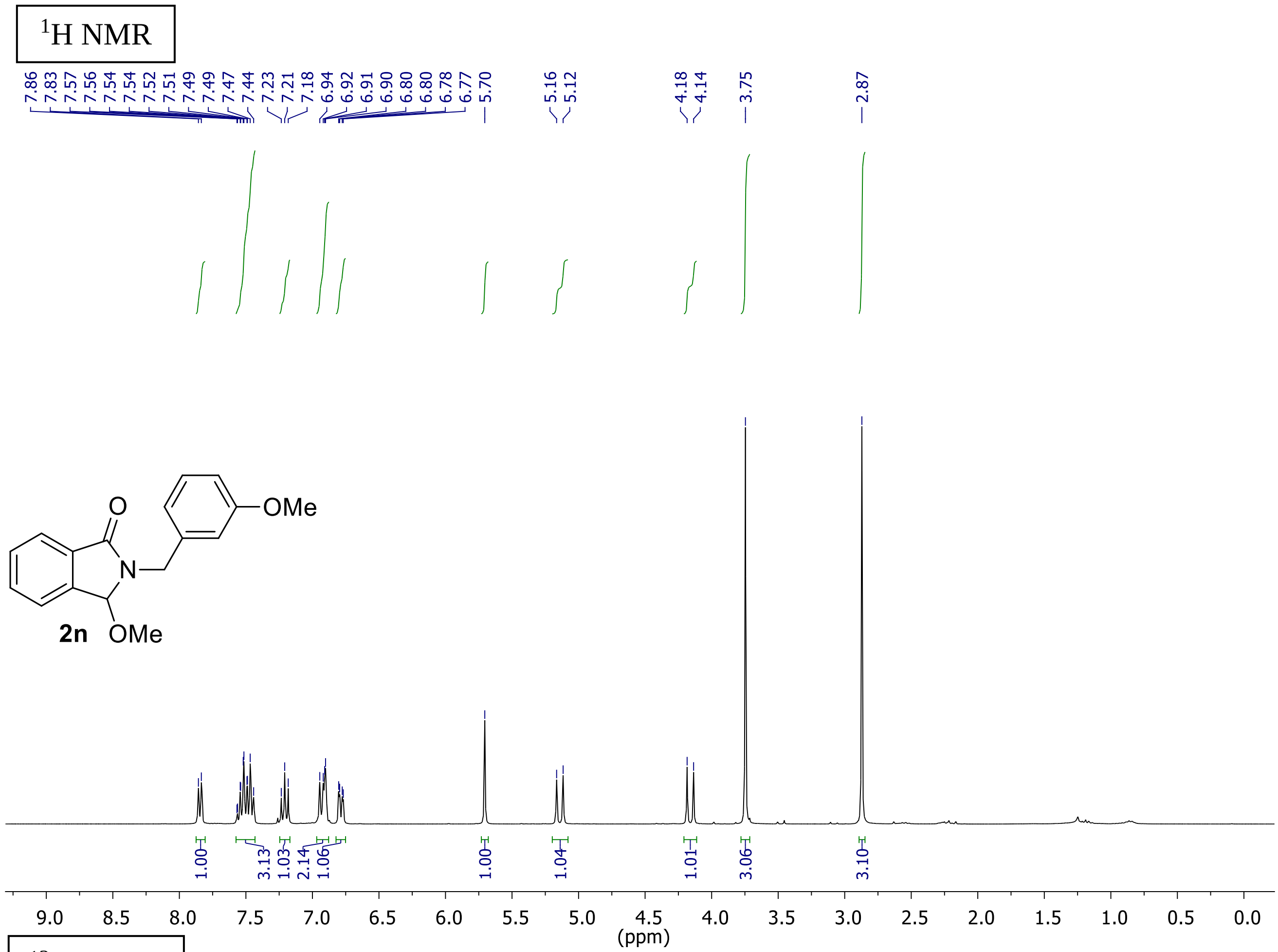

^{1}H NMR
7.86 7.83 7.57 7.56 7.54 7.54 7.52 7.51 7.49 7.49 7.47 7.44 7.23 7.21 7.18 6.94 6.92 6.91 6.90 6.80 6.80 6.78 6.77 5.70 5.16 5.12 4.18 4.14 3.75 2.87
OMe
O
N
2n
OMe
1.00 3.13 1.03 2.14 1.06 1.00 1.04 1.01 3.06 3.10
9.0 8.5 8.0 7.5 7.0 6.5 6.0 5.5 5.0 4.5 4.0 3.5 3.0 2.5 2.0 1.5 1.0 0.5 0.0
(ppm)

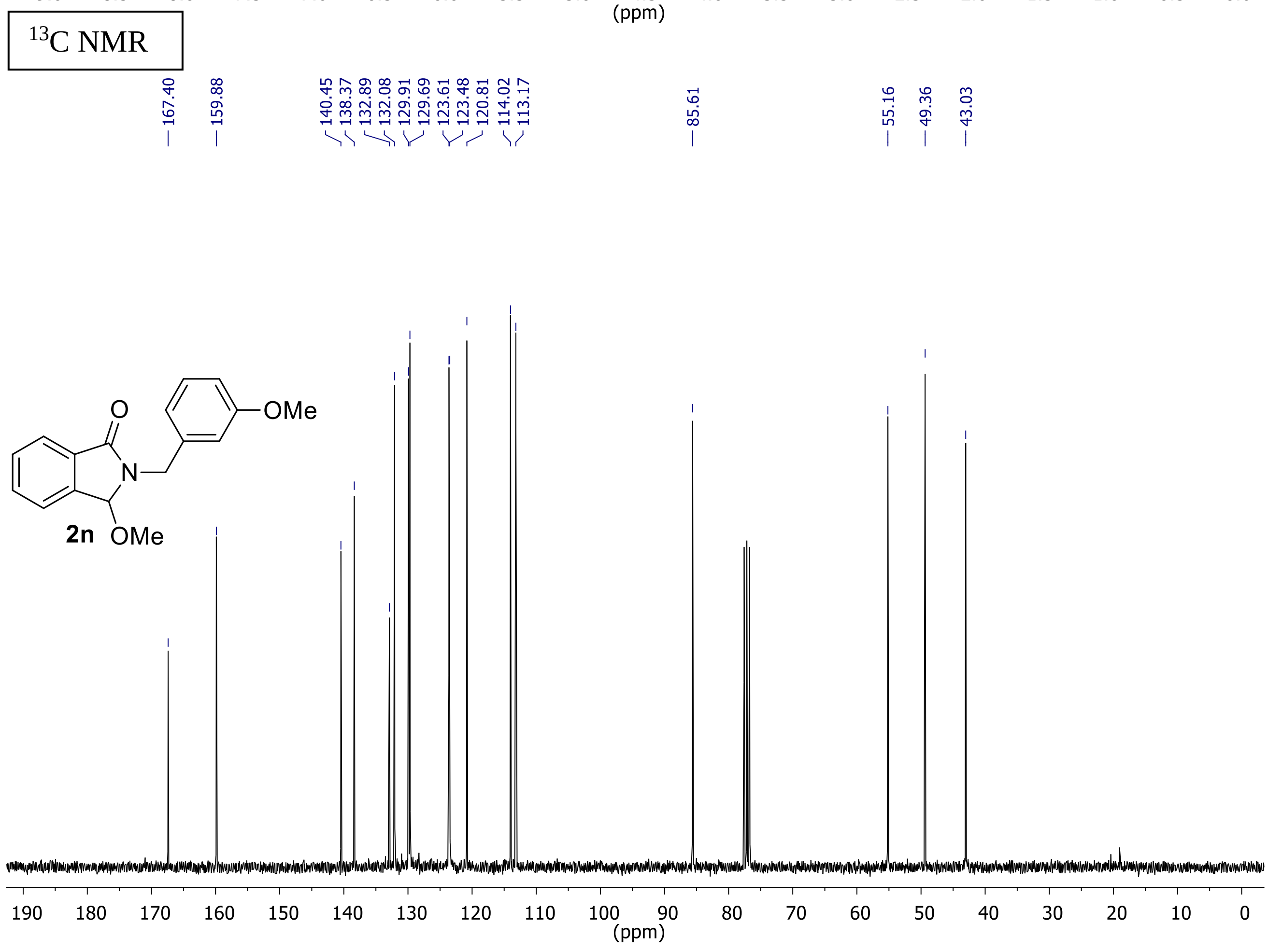

^{13}C NMR
167.40 159.88 140.45 138.37 132.89 132.08 129.91 129.69 123.61 123.48 120.81 114.02 113.17 85.61 55.16 49.36 43.03
OMe
O
N
2n
OMe
190 180 170 160 150 140 130 120 110 100 90 80 70 60 50 40 30 20 10 0
(ppm)

^{1}H NMR

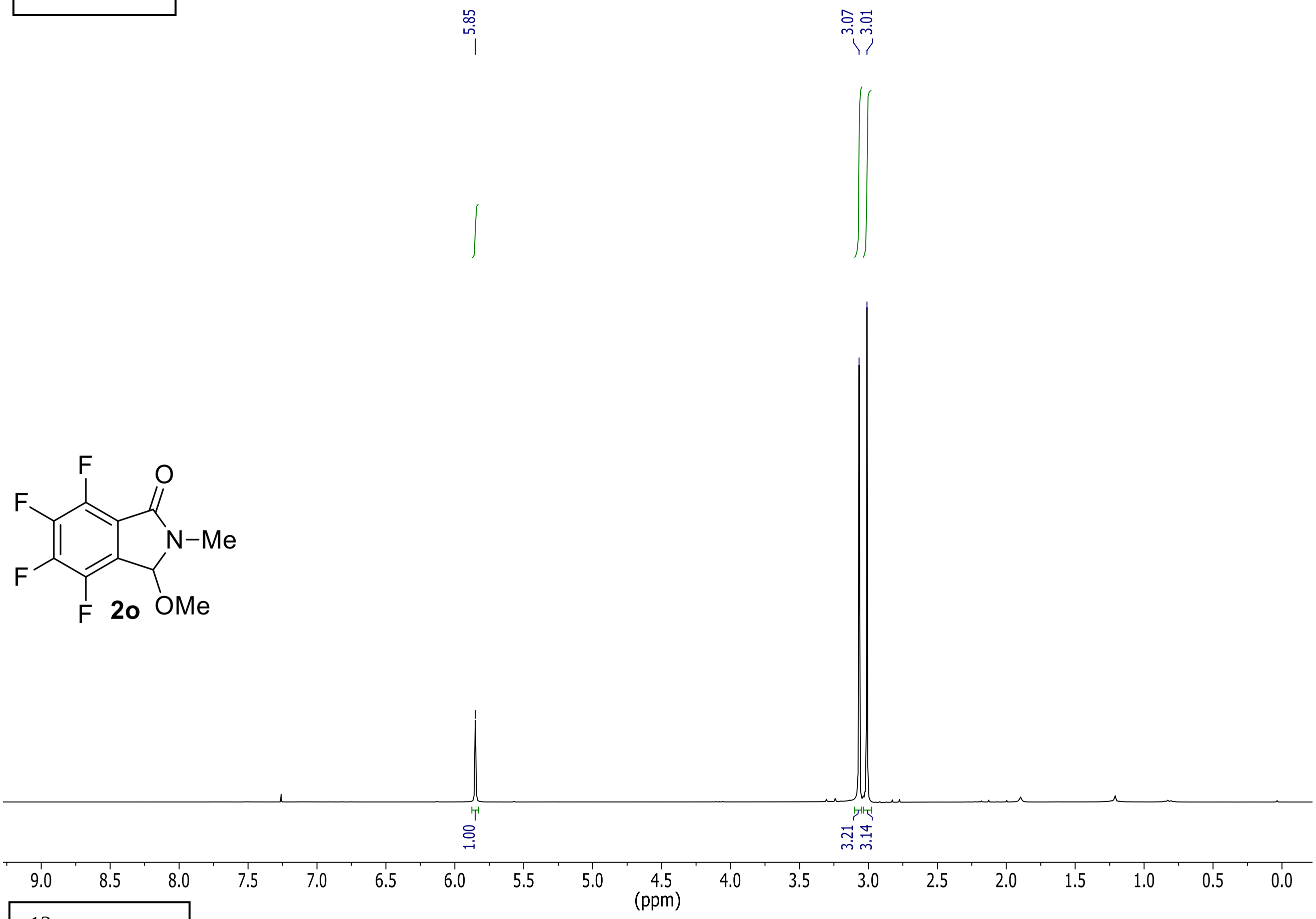

^{13}C NMR

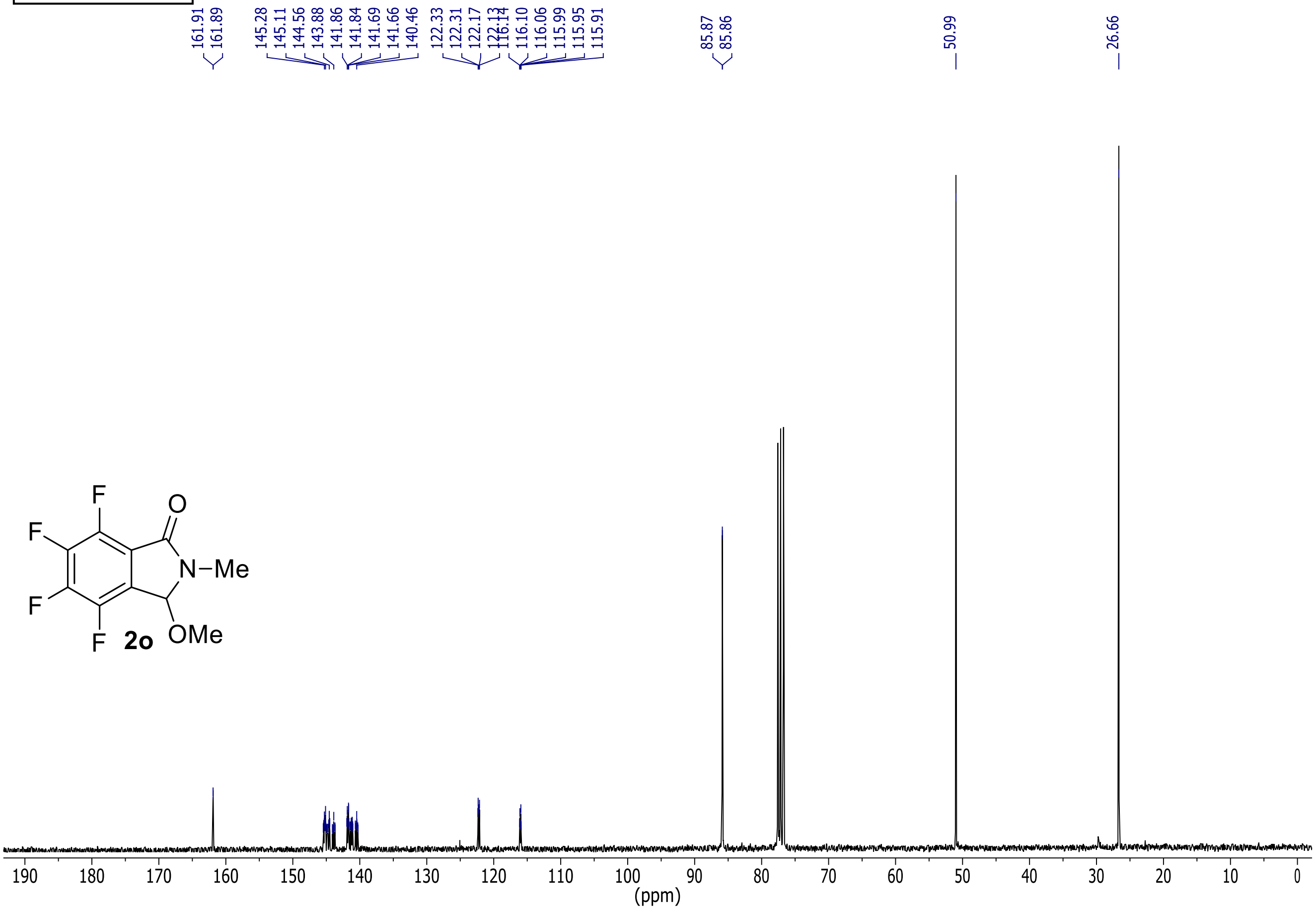

^{19}F NMR

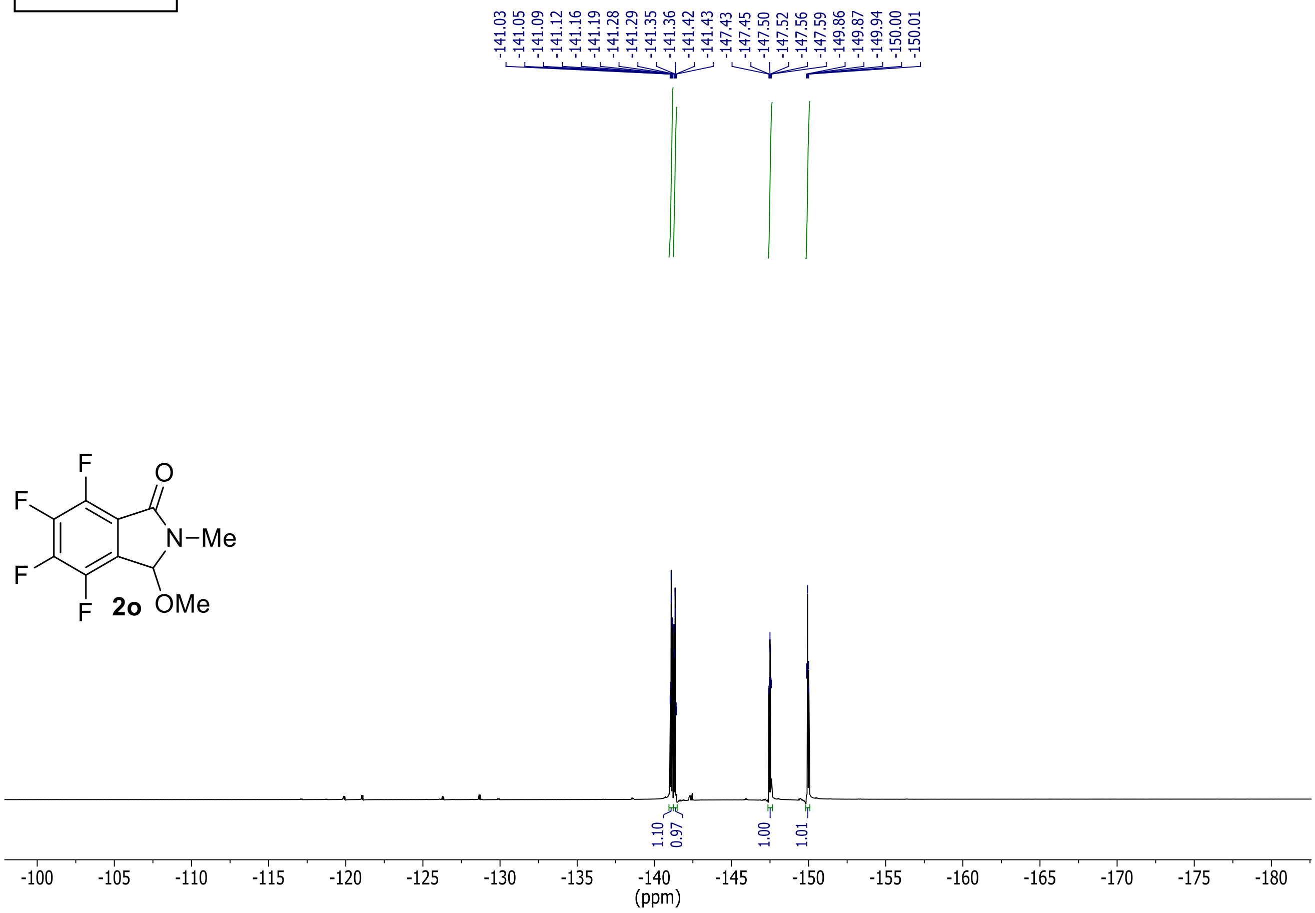
-141.03
-141.05
-141.09
-141.12
-141.16
-141.19
-141.28
-141.29
-141.35
-141.36
-141.42
-141.43
-147.43
-147.45
-147.50
-147.52
-147.56
-147.59
-149.86
-149.87
-149.94
-150.00
-150.01
F
F
F
F
O
N–Me
OMe
2o
1.10
0.97
1.00
1.01
-100
-105
-110
-115
-120
-125
-130
-135
-140
-145
-150
-155
-160
-165
-170
-175
-180
(ppm)

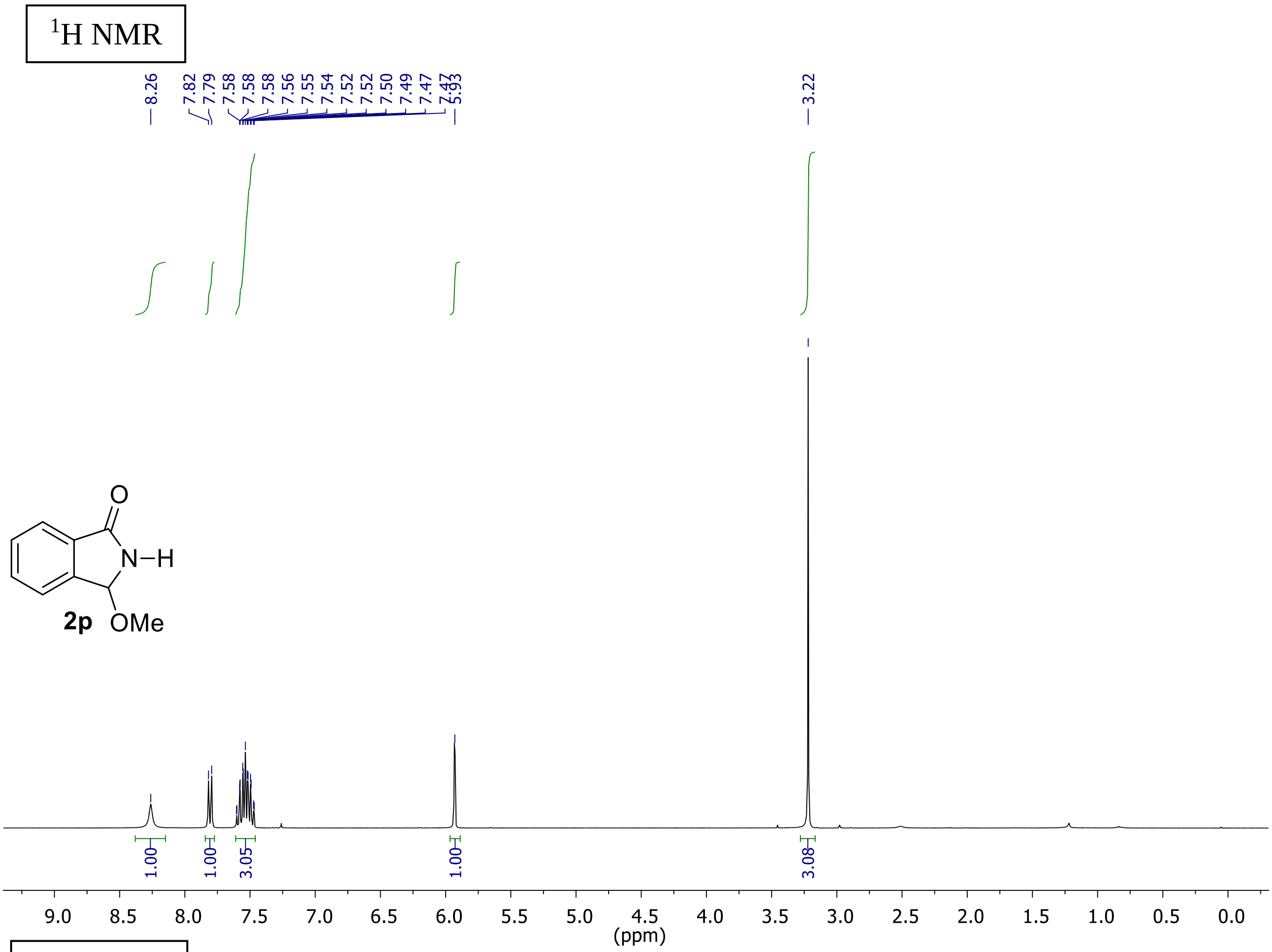

1H NMR
8.26
7.82
7.79
7.58
7.58
7.58
7.56
7.55
7.54
7.52
7.52
7.50
7.49
7.47
7.47
5.93
3.22
O
N−H
2p
OMe
1.00
1.00
3.05
1.00
3.08
9.0 8.5 8.0 7.5 7.0 6.5 6.0 5.5 5.0 4.5 4.0 3.5 3.0 2.5 2.0 1.5 1.0 0.5 0.0
(ppm)

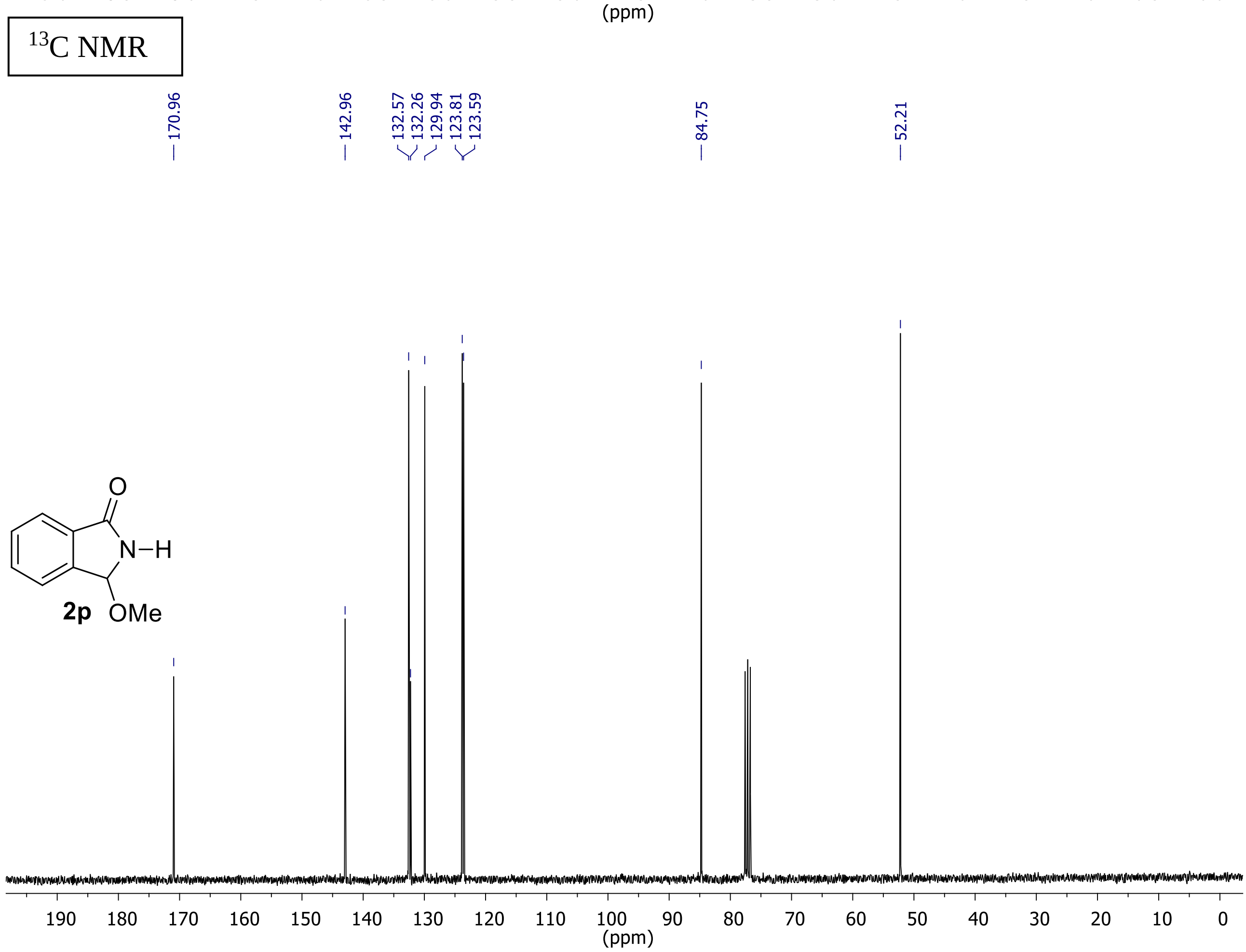

13C NMR
170.96
142.96
132.57
132.26
129.94
123.81
123.59
84.75
52.21
O
N−H
2p
OMe
190 180 170 160 150 140 130 120 110 100 90 80 70 60 50 40 30 20 10 0
(ppm)

^{1}H NMR

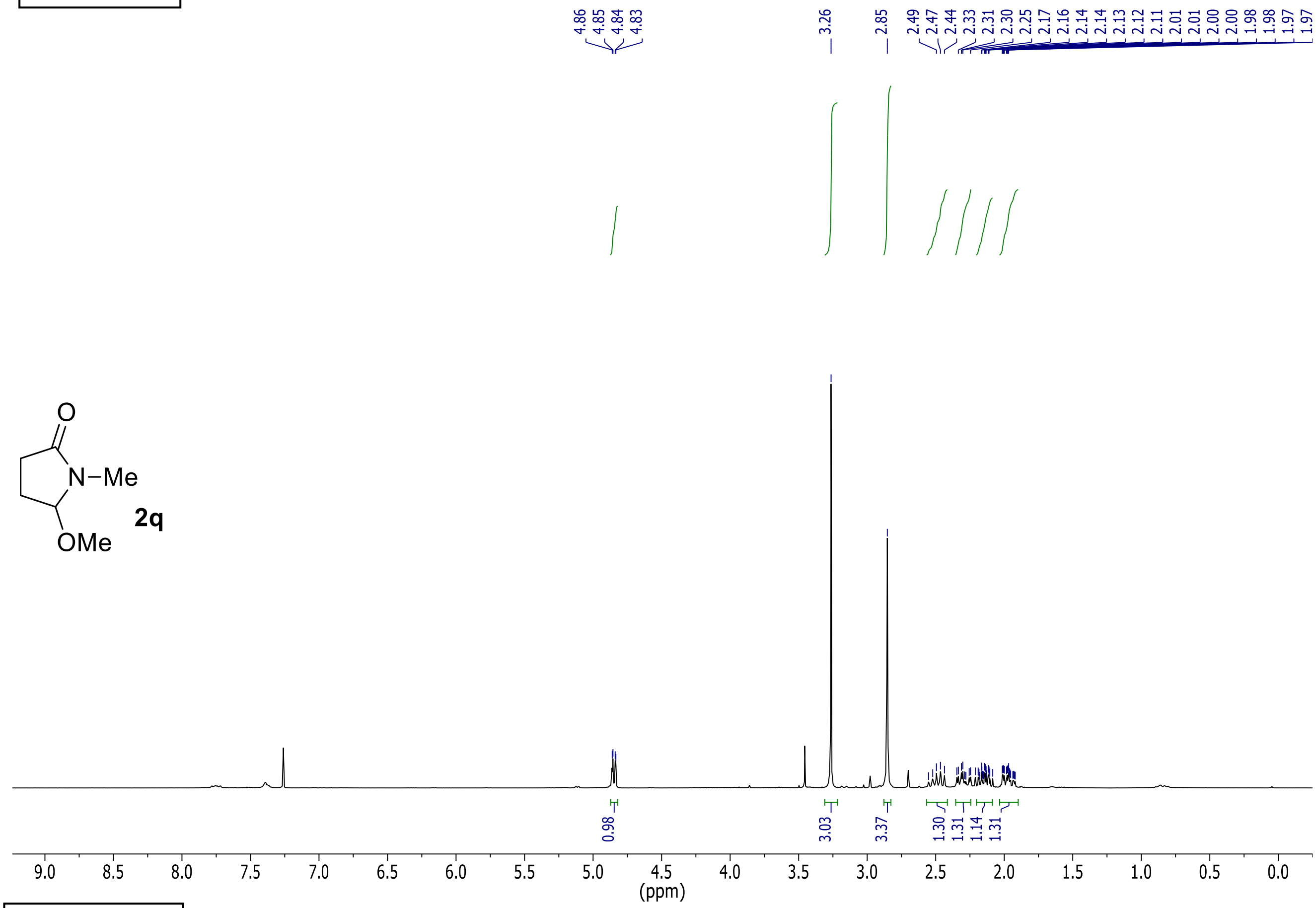

^{13}C NMR

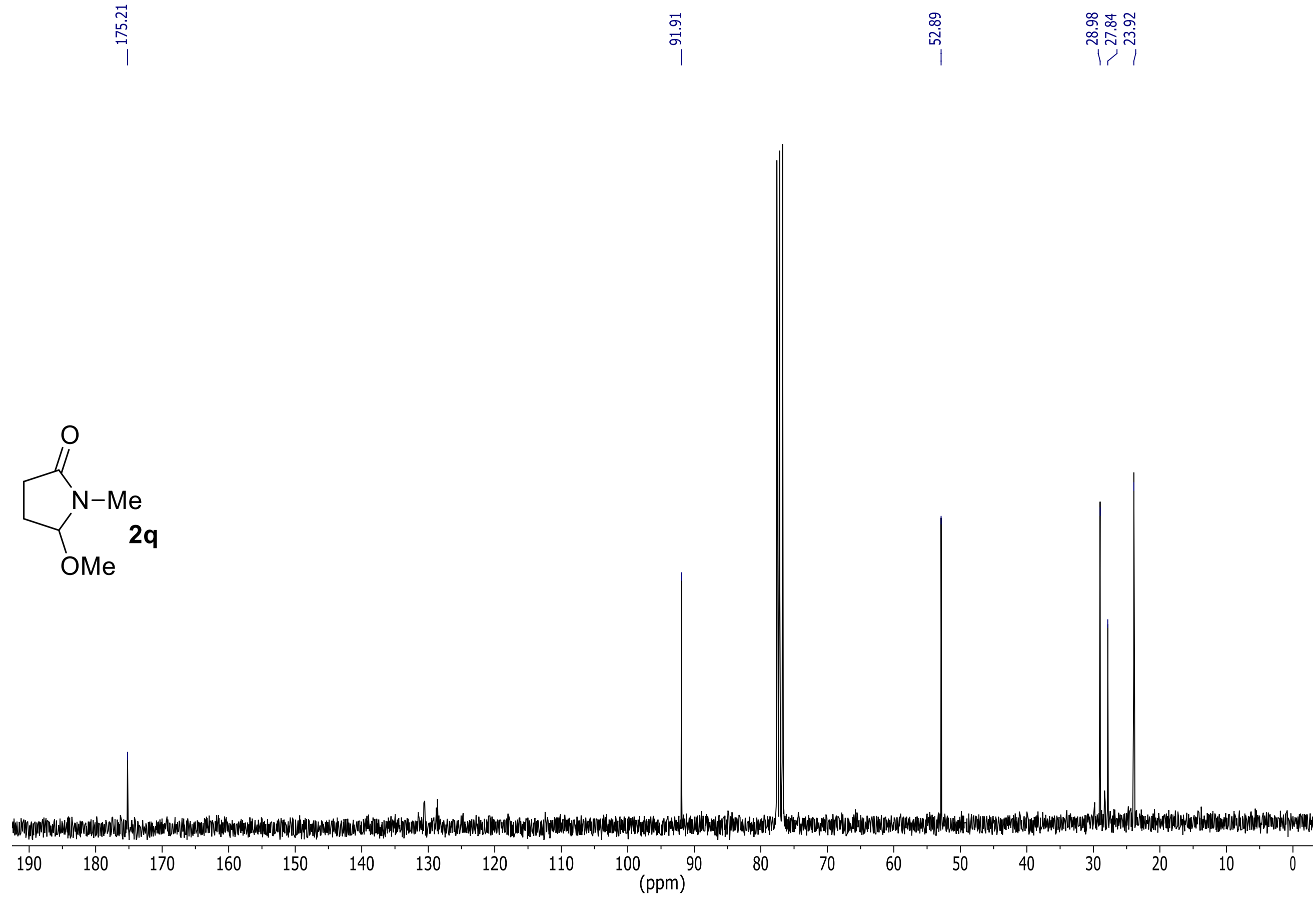

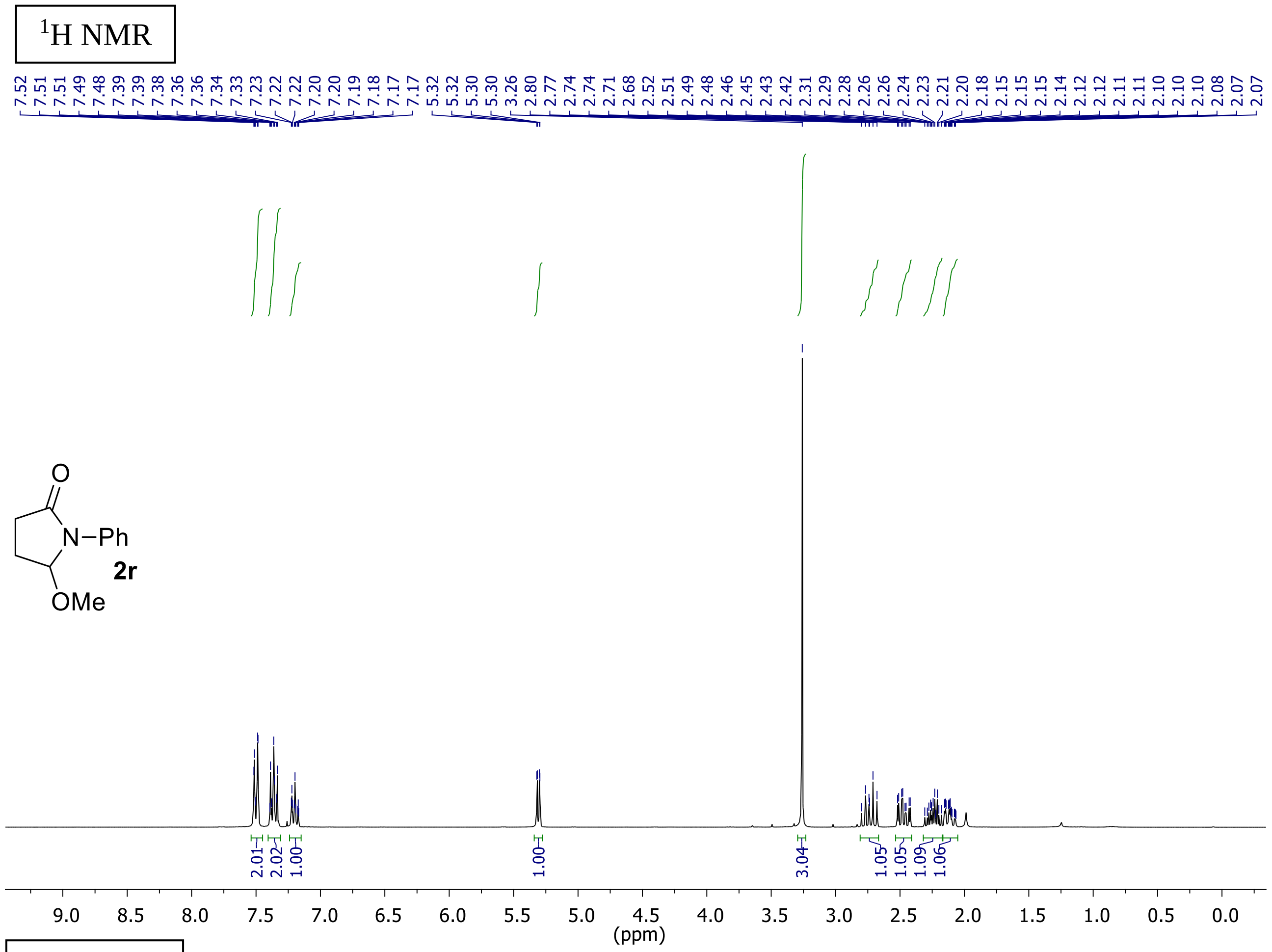

^{1}H NMR
7.52 7.51 7.51 7.49 7.48 7.39 7.39 7.38 7.36 7.36 7.34 7.33 7.23 7.22 7.22 7.20 7.19 7.18 7.17 7.17 5.32 5.32 5.30 5.30 3.26 2.80 2.77 2.74 2.74 2.71 2.68 2.52 2.51 2.49 2.48 2.46 2.45 2.43 2.42 2.31 2.29 2.28 2.26 2.26 2.24 2.23 2.21 2.20 2.18 2.15 2.15 2.15 2.14 2.12 2.12 2.11 2.11 2.10 2.10 2.10 2.08 2.07 2.07
O
N−Ph
2r
OMe
2.01
2.02
1.00
1.00
3.04
1.05
1.05
1.09
1.06
9.0 8.5 8.0 7.5 7.0 6.5 6.0 5.5 5.0 4.5 4.0 3.5 3.0 2.5 2.0 1.5 1.0 0.5 0.0
(ppm)

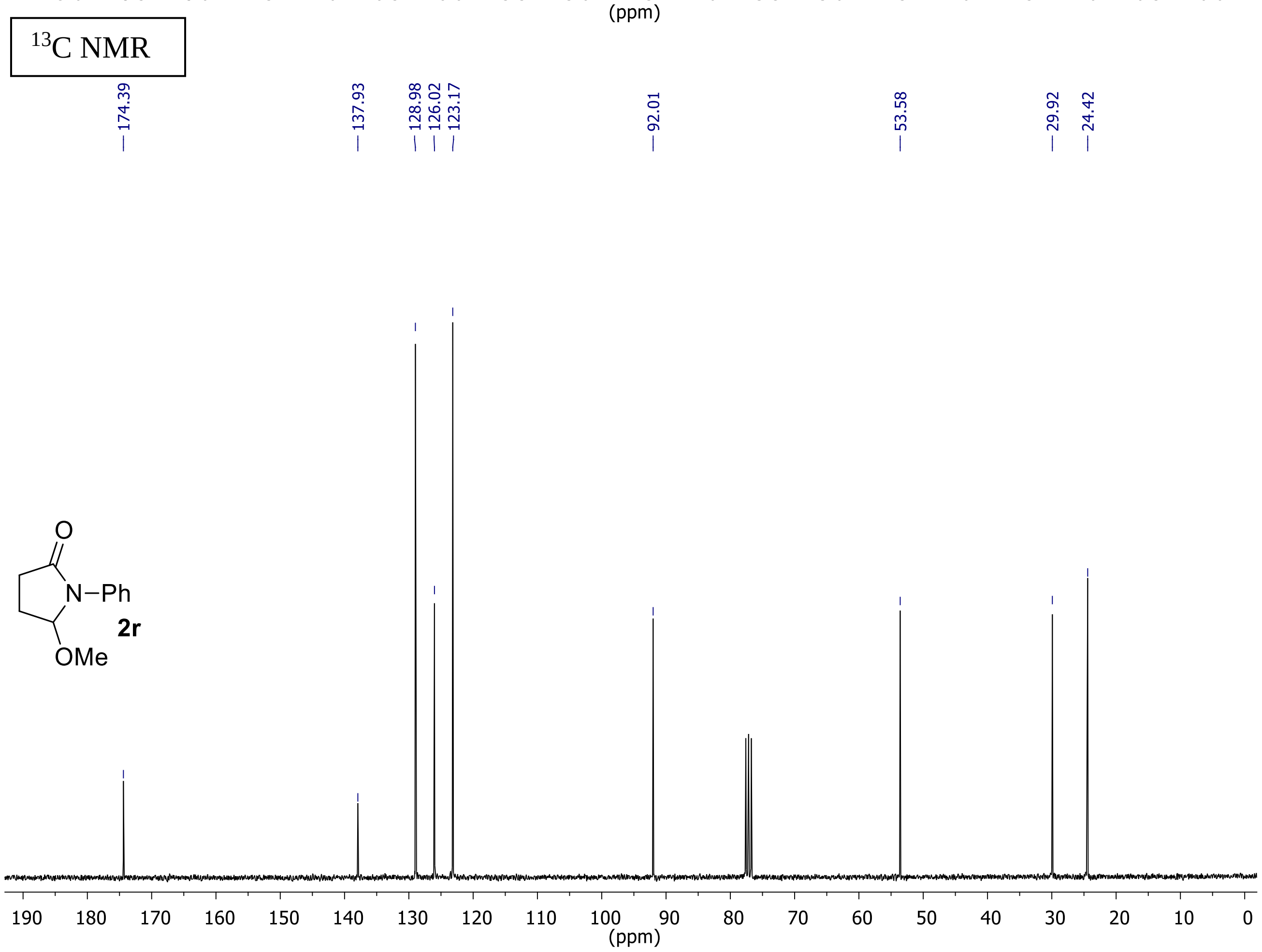

^{13}C NMR
174.39
137.93
128.98
126.02
123.17
92.01
53.58
29.92
24.42
O
N−Ph
2r
OMe
190 180 170 160 150 140 130 120 110 100 90 80 70 60 50 40 30 20 10 0
(ppm)

^{1}H NMR

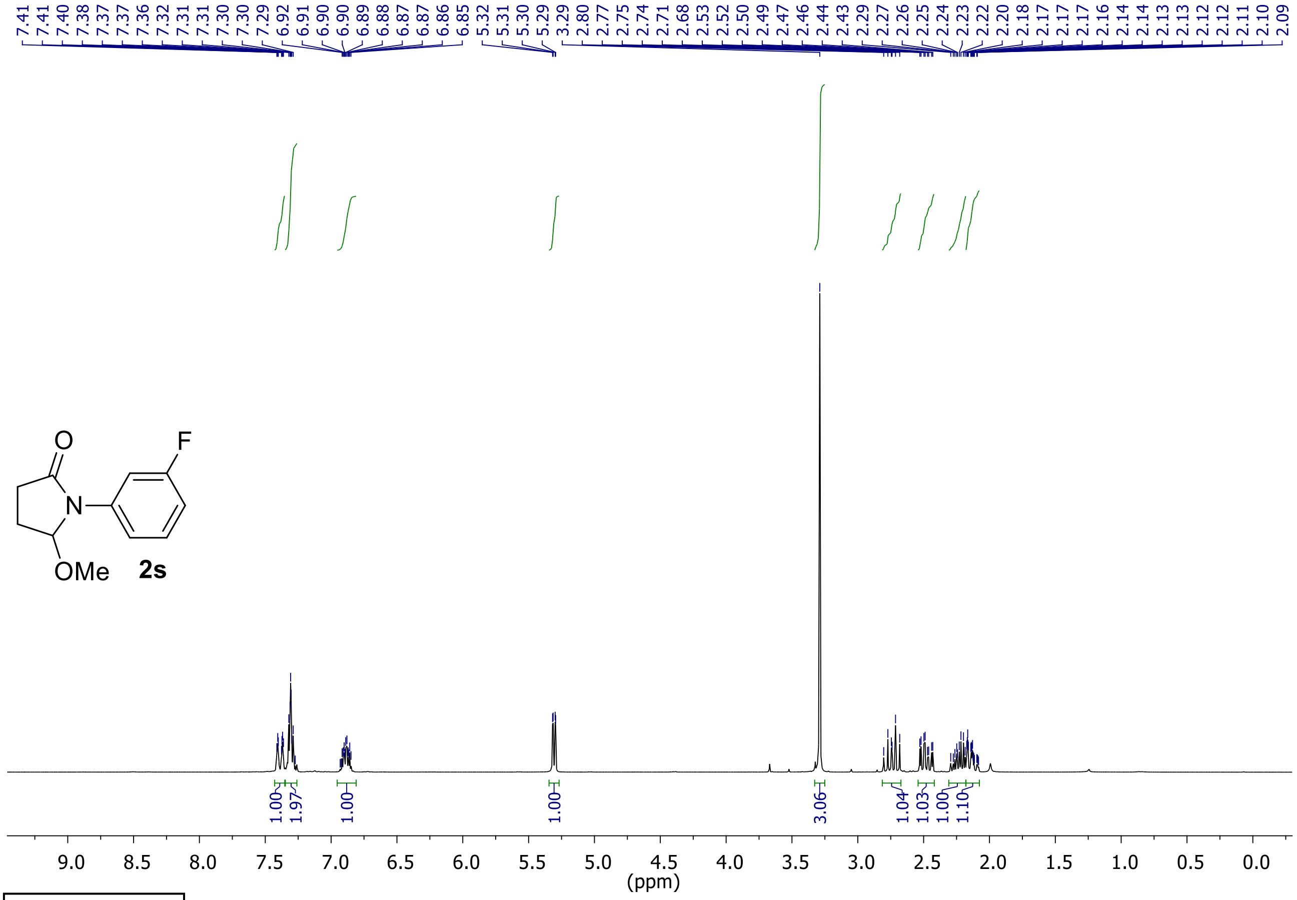

^{13}C NMR

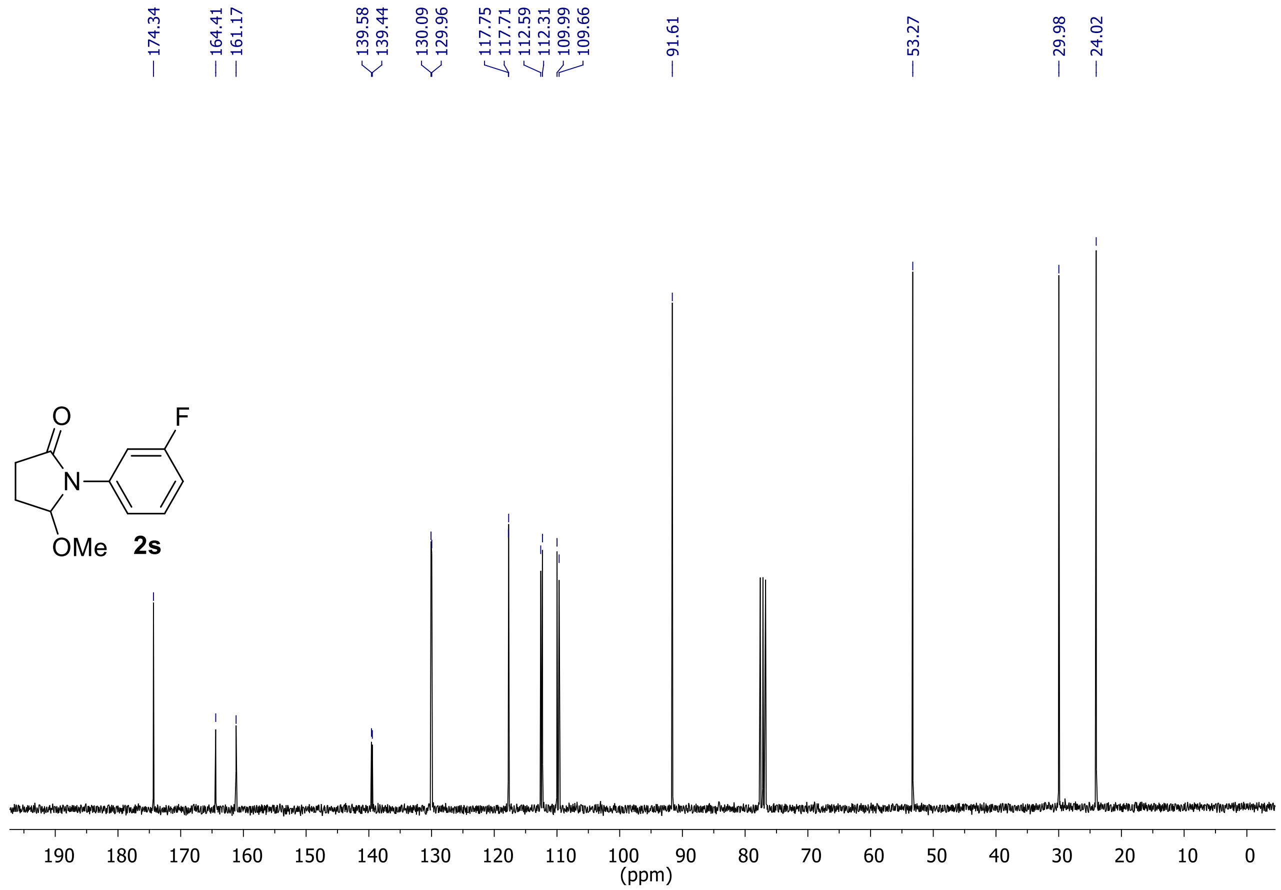

^{19}F NMR

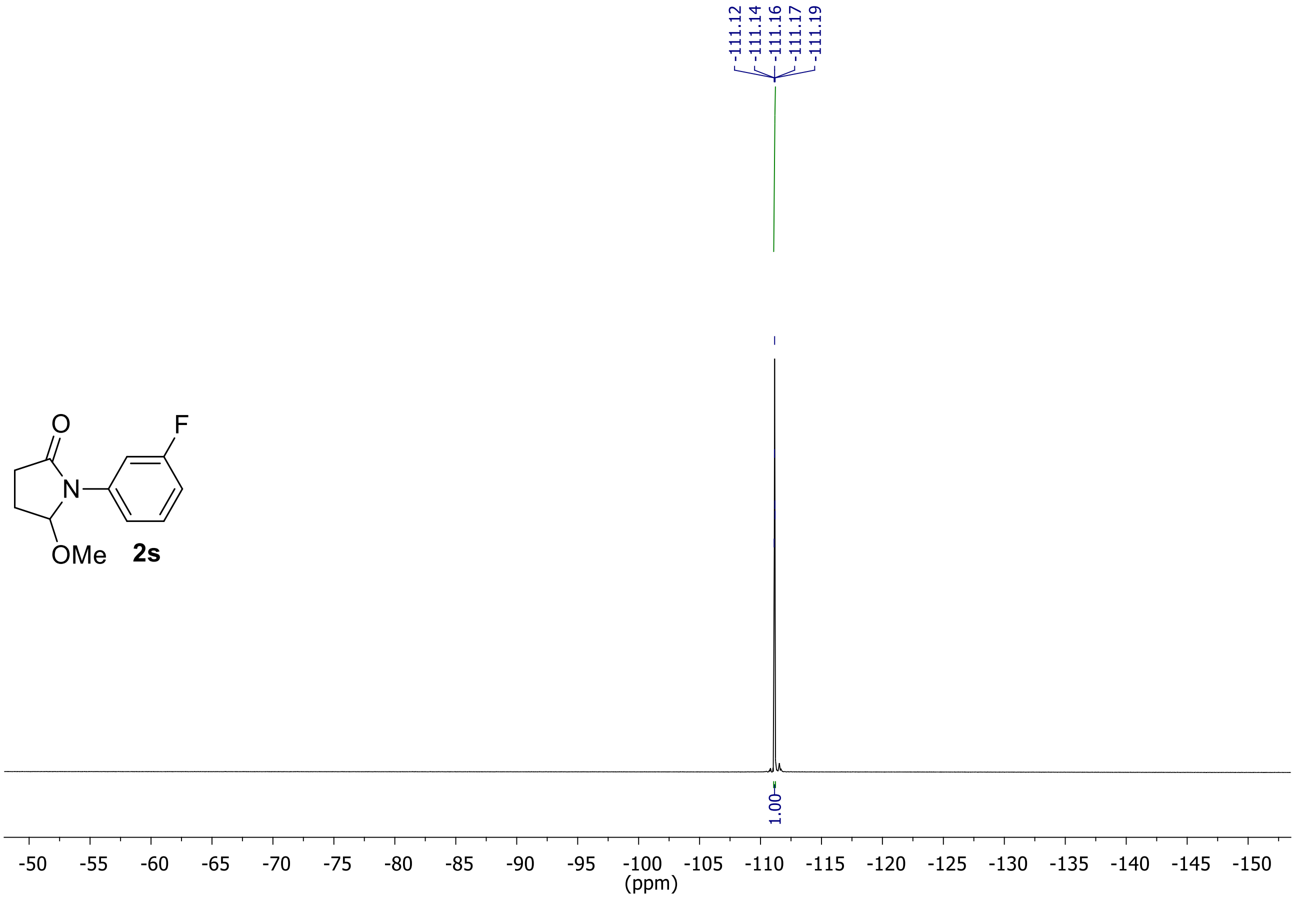

-111.12
-111.14
-111.16
-111.17
-111.19
O
F
N
OMe
2s
1.00
-50 -55 -60 -65 -70 -75 -80 -85 -90 -95 -100 -105 -110 -115 -120 -125 -130 -135 -140 -145 -150
(ppm)

^{1}H NMR

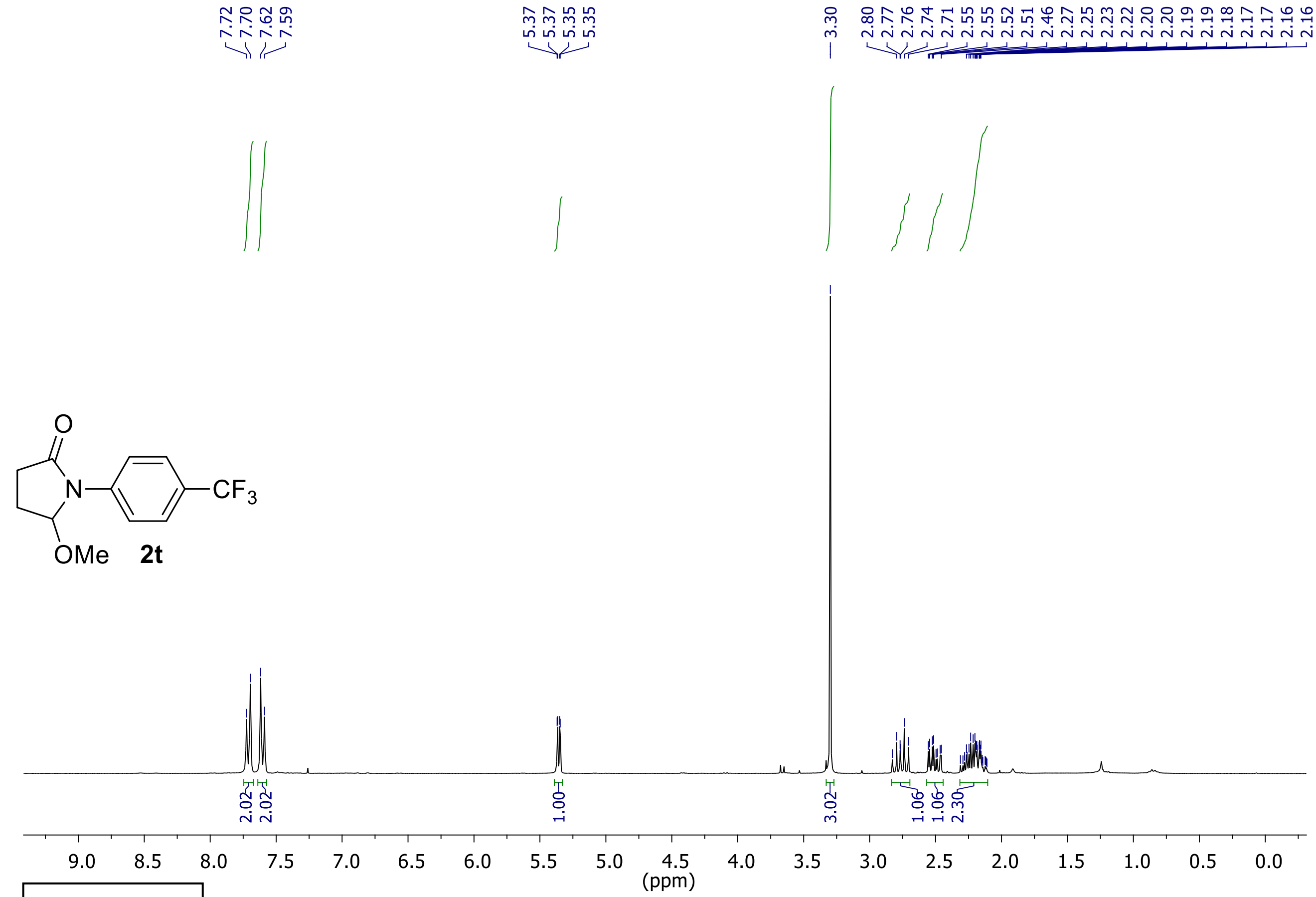

^{13}C NMR

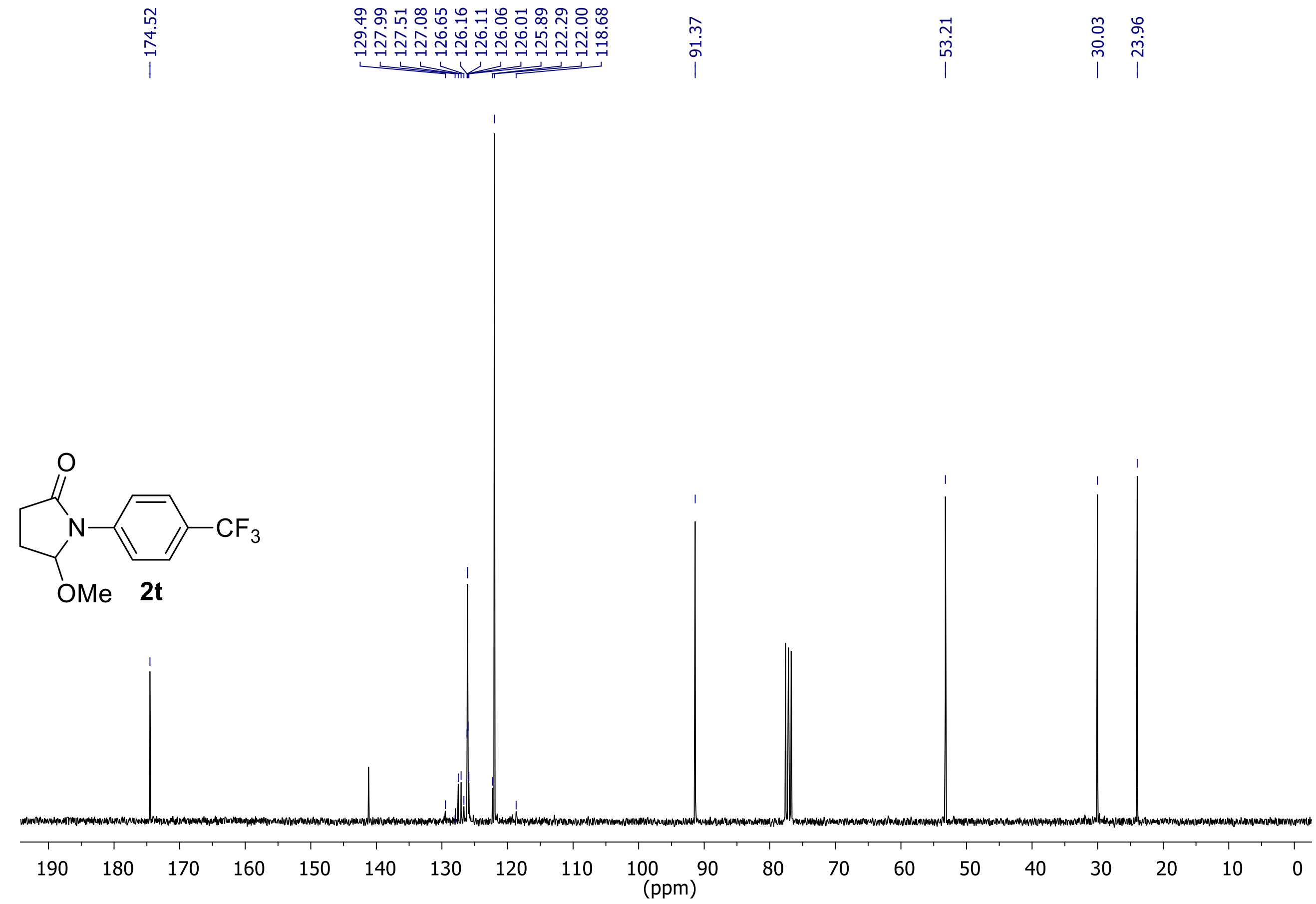

^{19}F NMR

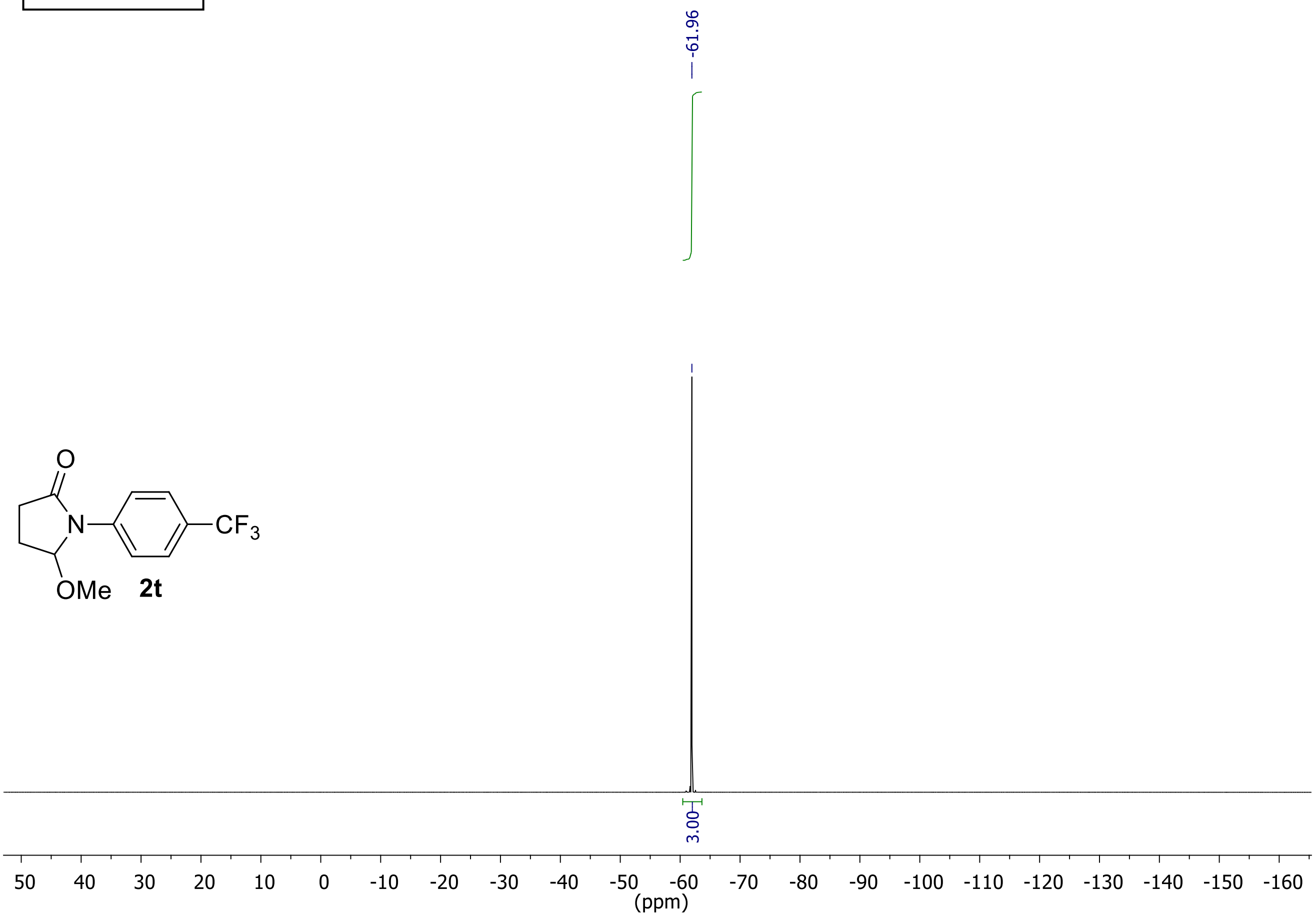

-61.96
O
N
CF3
OMe
2t
3.00
50 40 30 20 10 0 -10 -20 -30 -40 -50 -60 -70 -80 -90 -100 -110 -120 -130 -140 -150 -160
(ppm)

^{1}H NMR

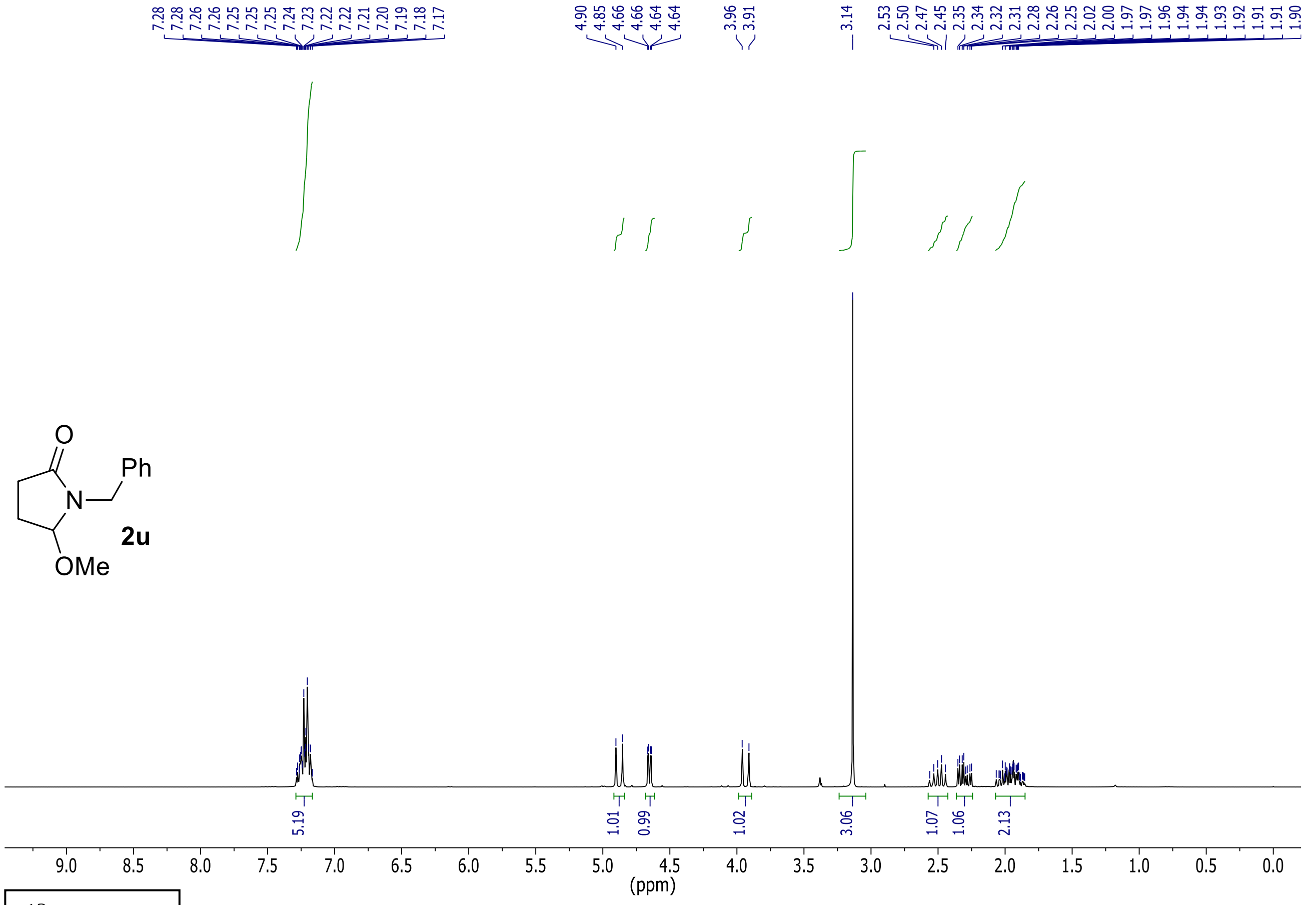

^{13}C NMR

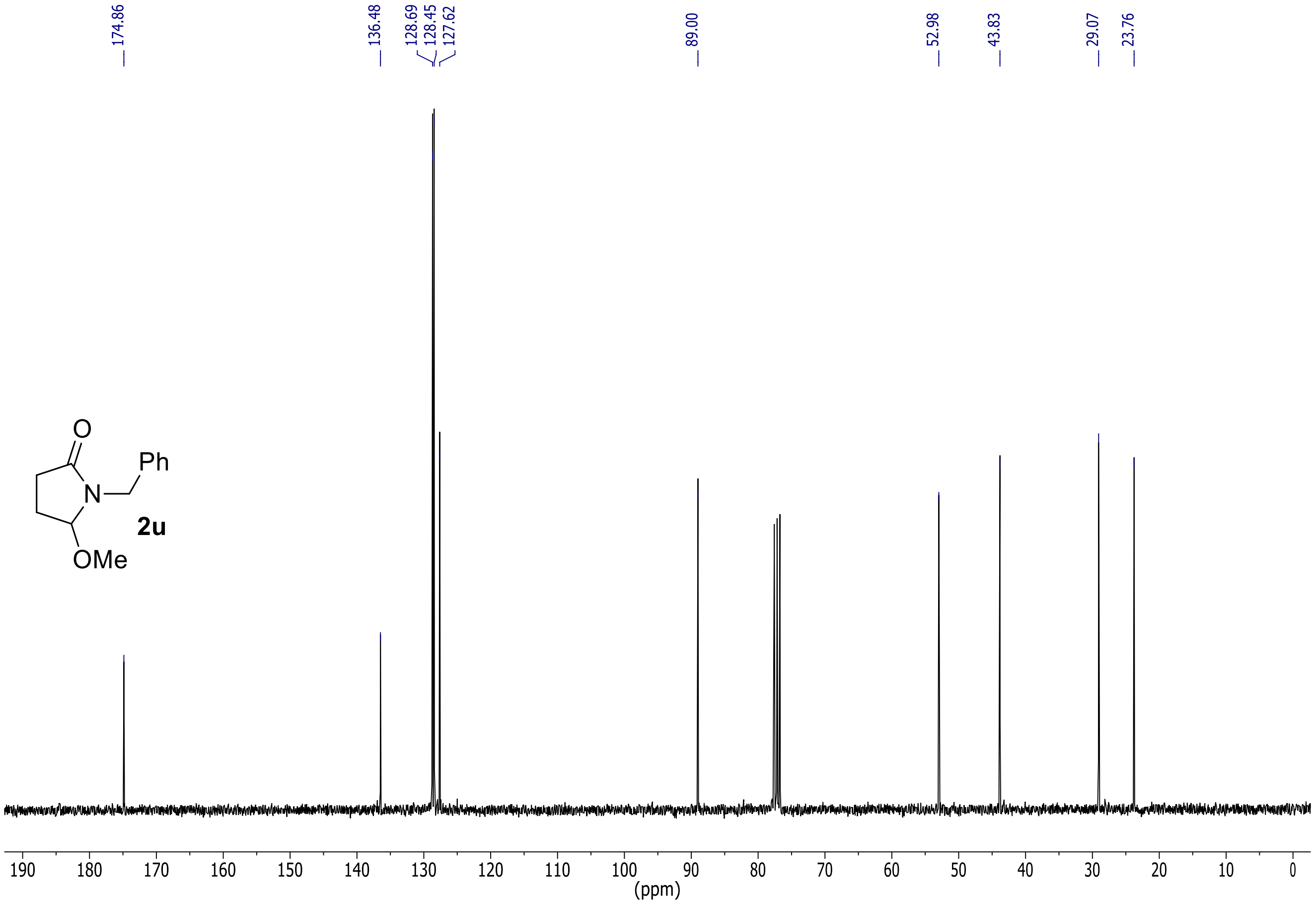

^{1}H NMR

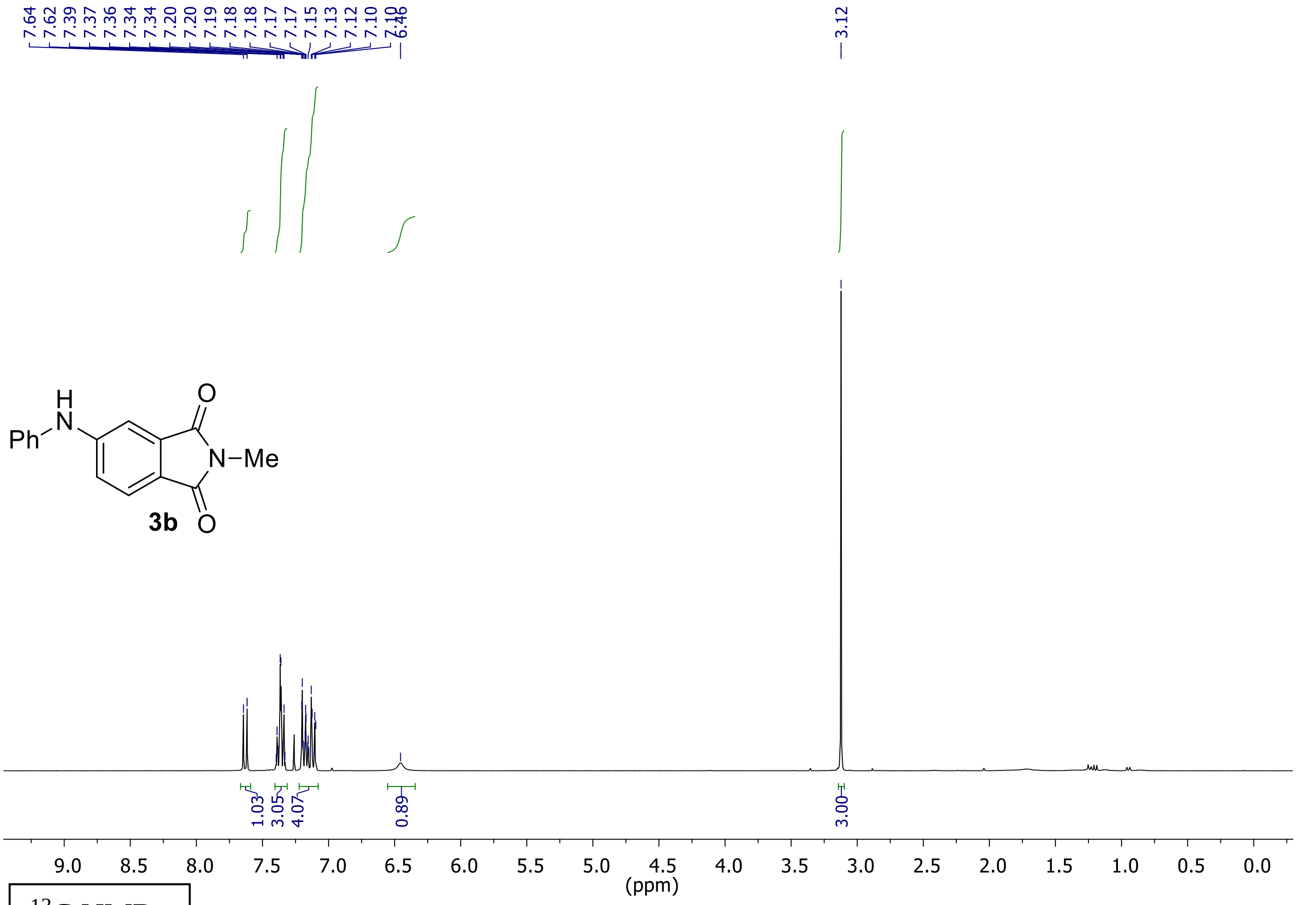

^{13}C NMR

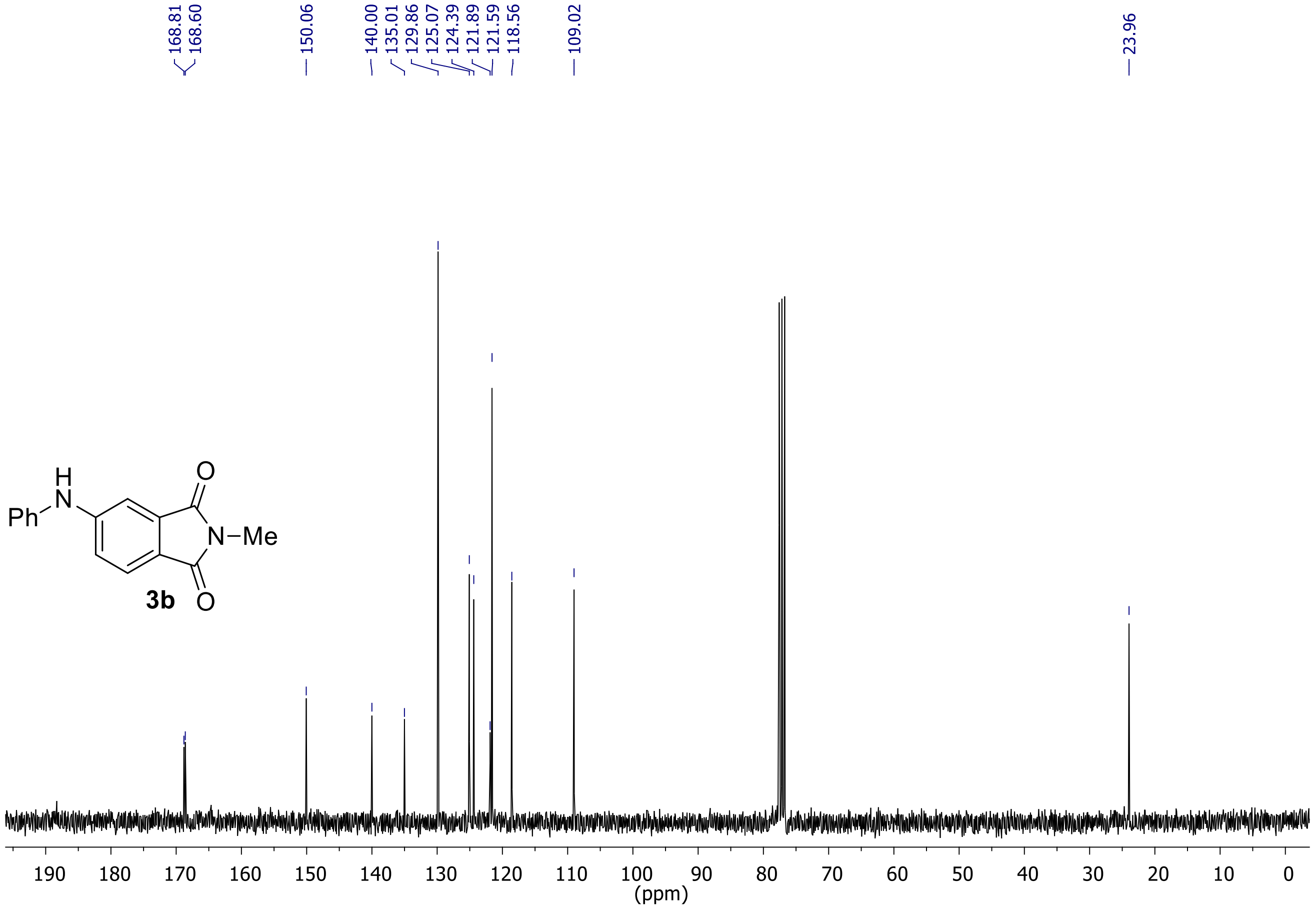

^{1}H NMR

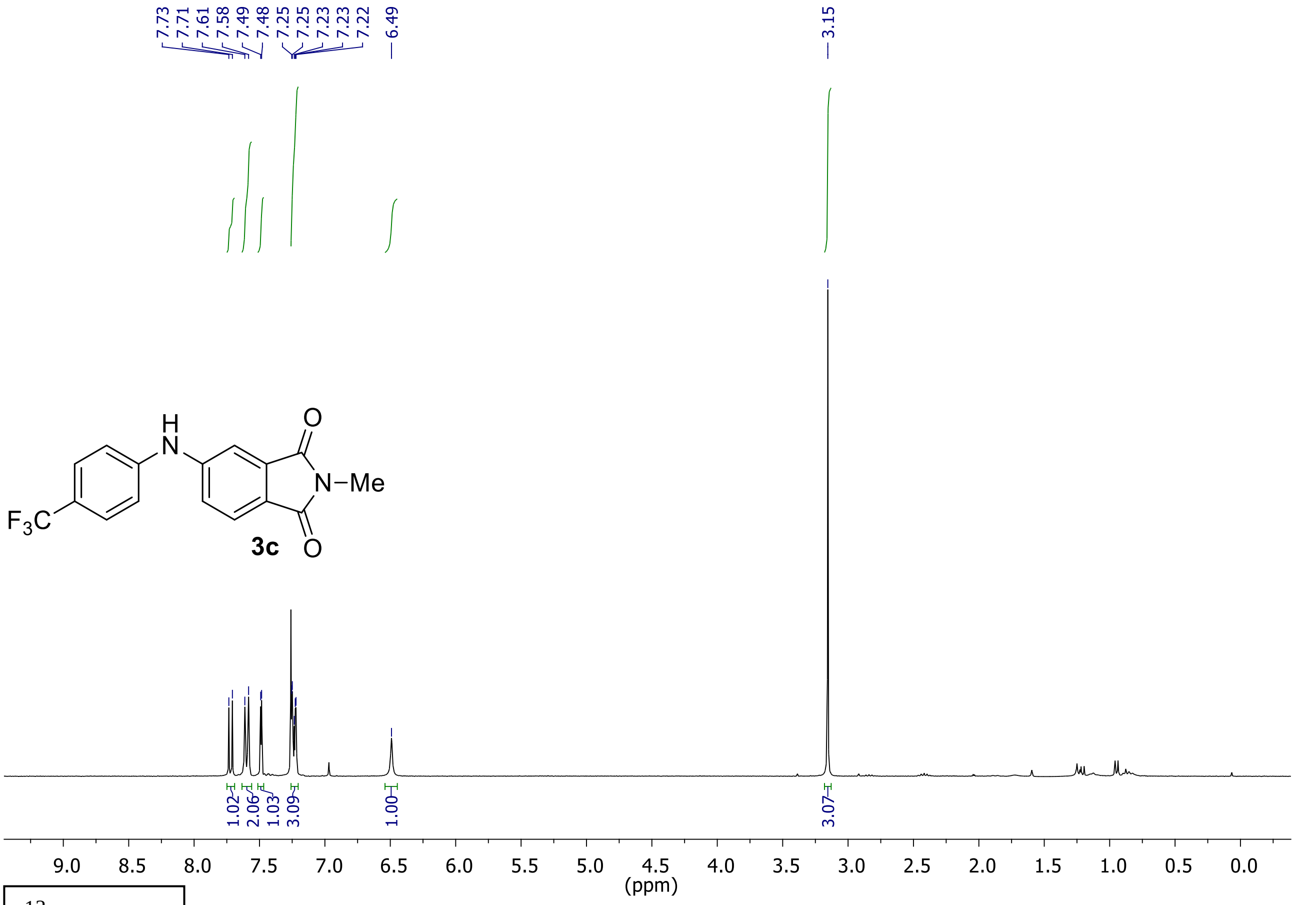

^{13}C NMR

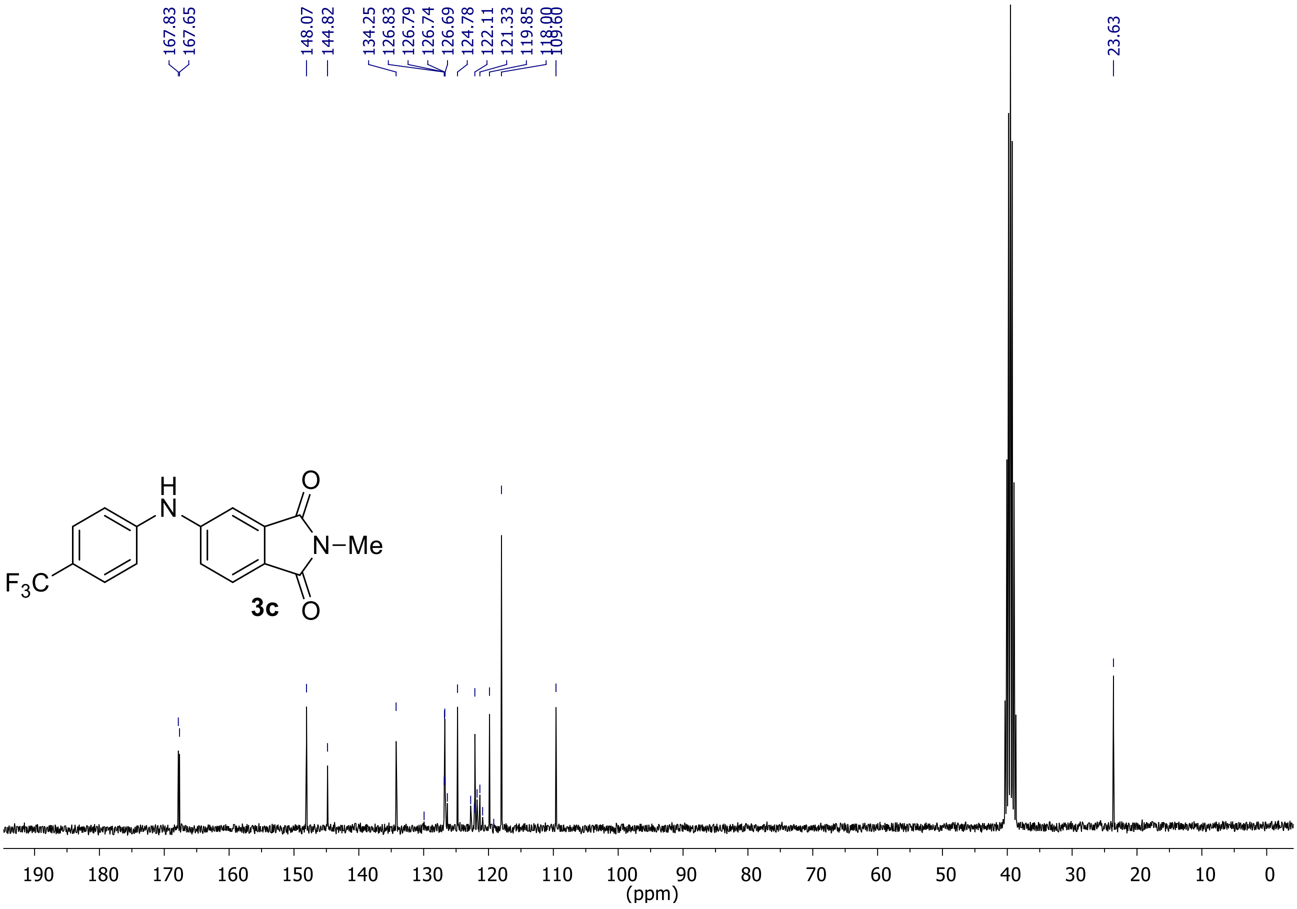

^{19}F NMR

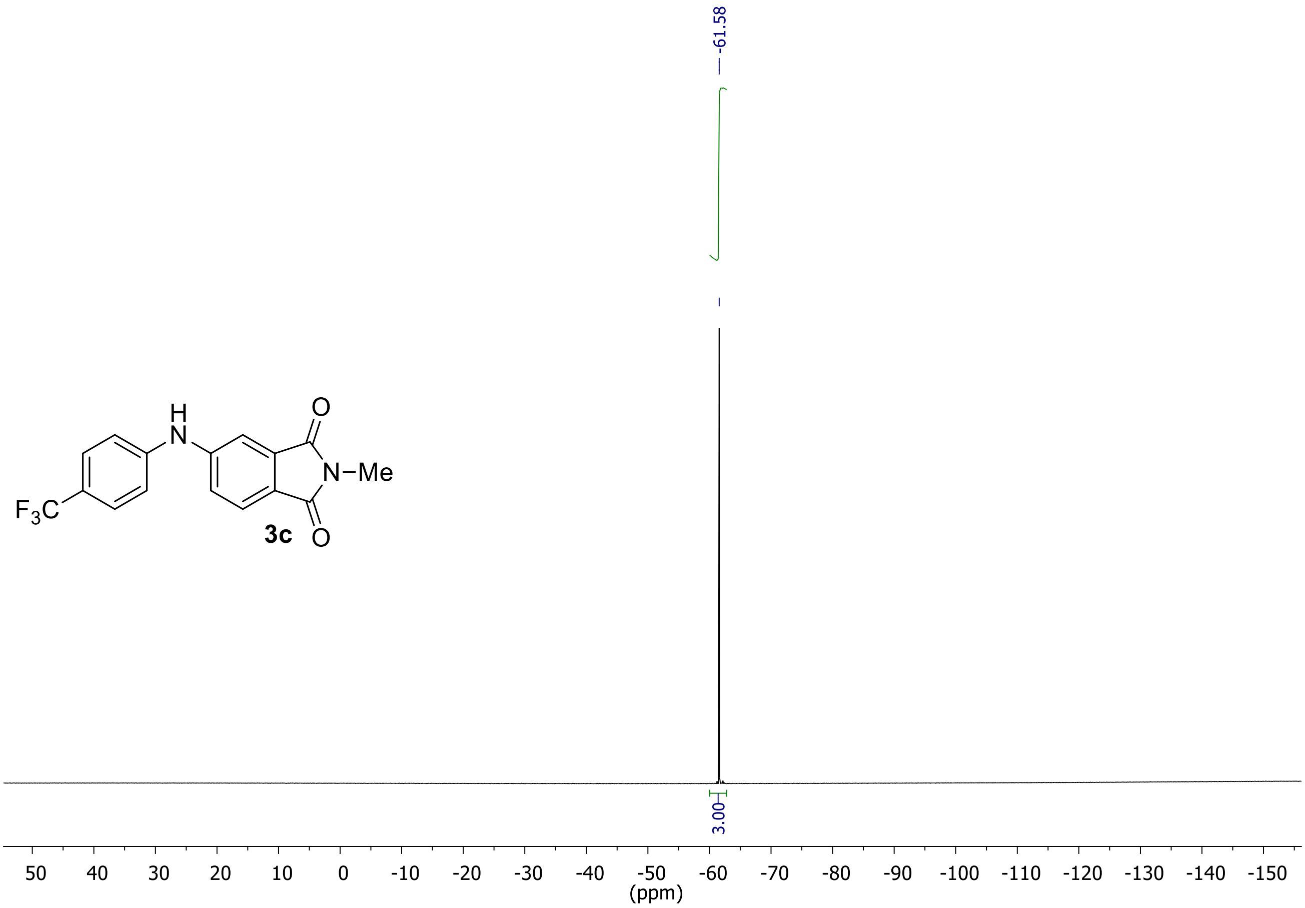
-61.58
H
N
O
N−Me
O
F3C
3c
3.00
50 40 30 20 10 0 -10 -20 -30 -40 -50 -60 -70 -80 -90 -100 -110 -120 -130 -140 -150
(ppm)

^{1}H NMR

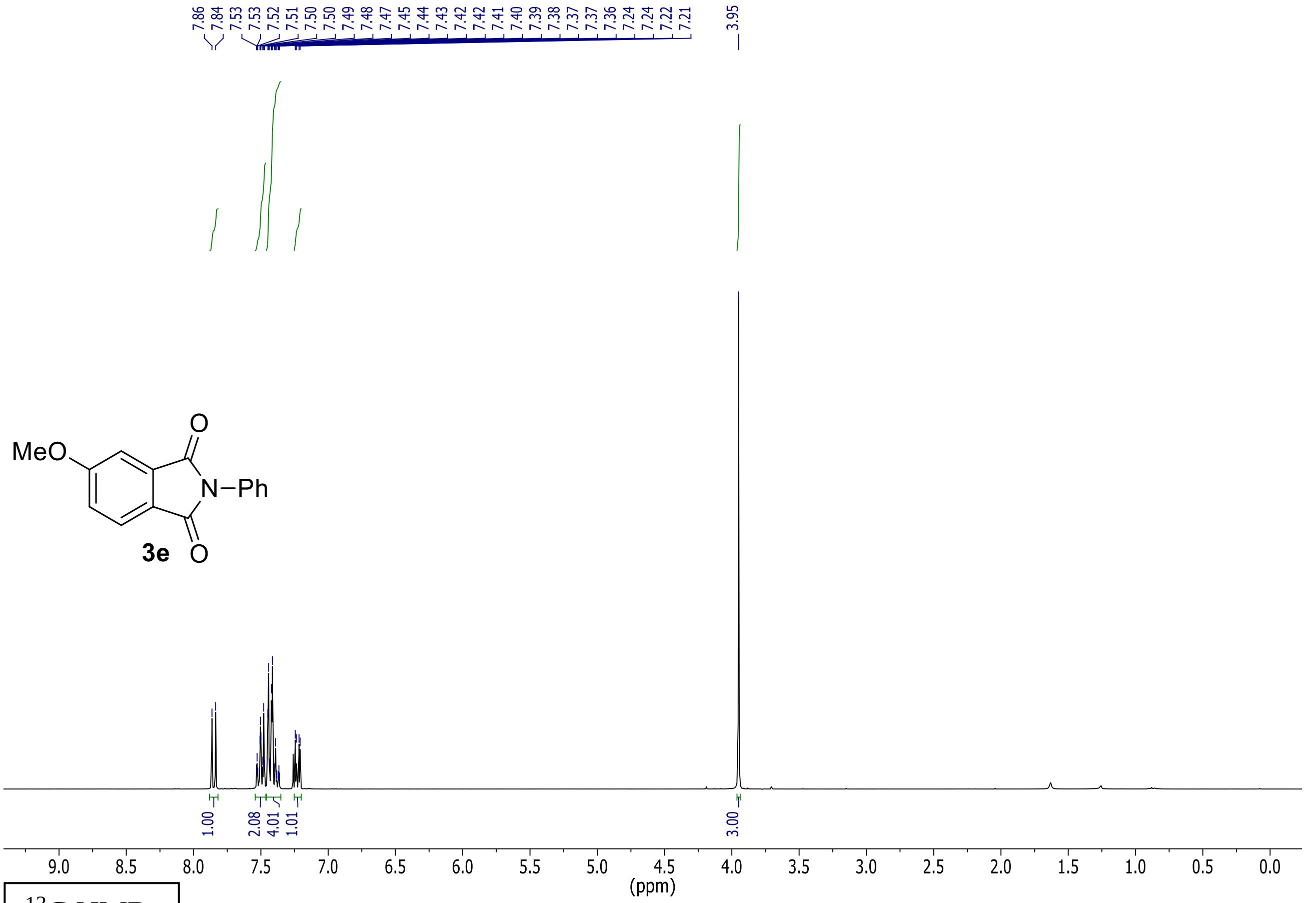

^{13}C NMR

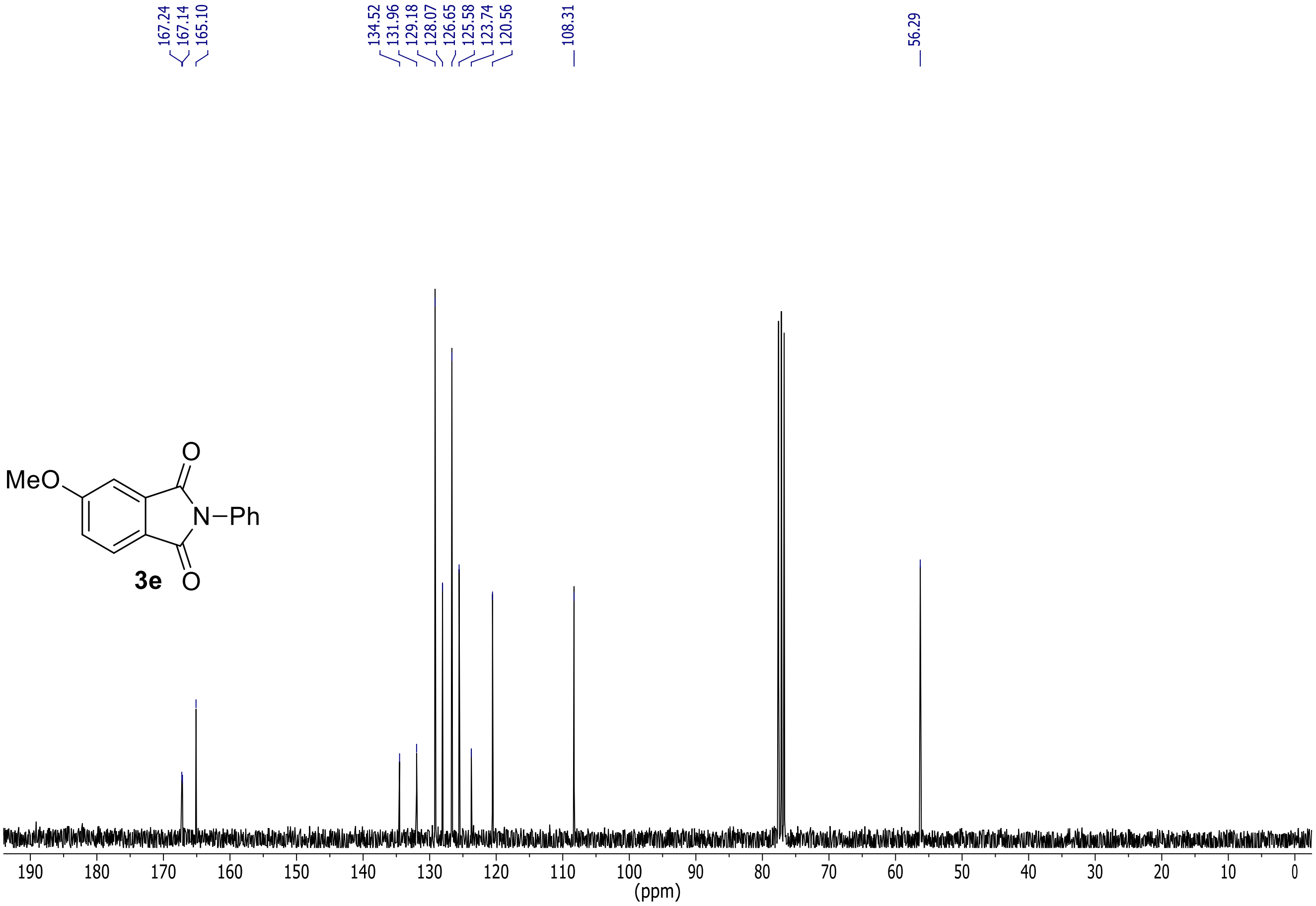

^{1}H NMR

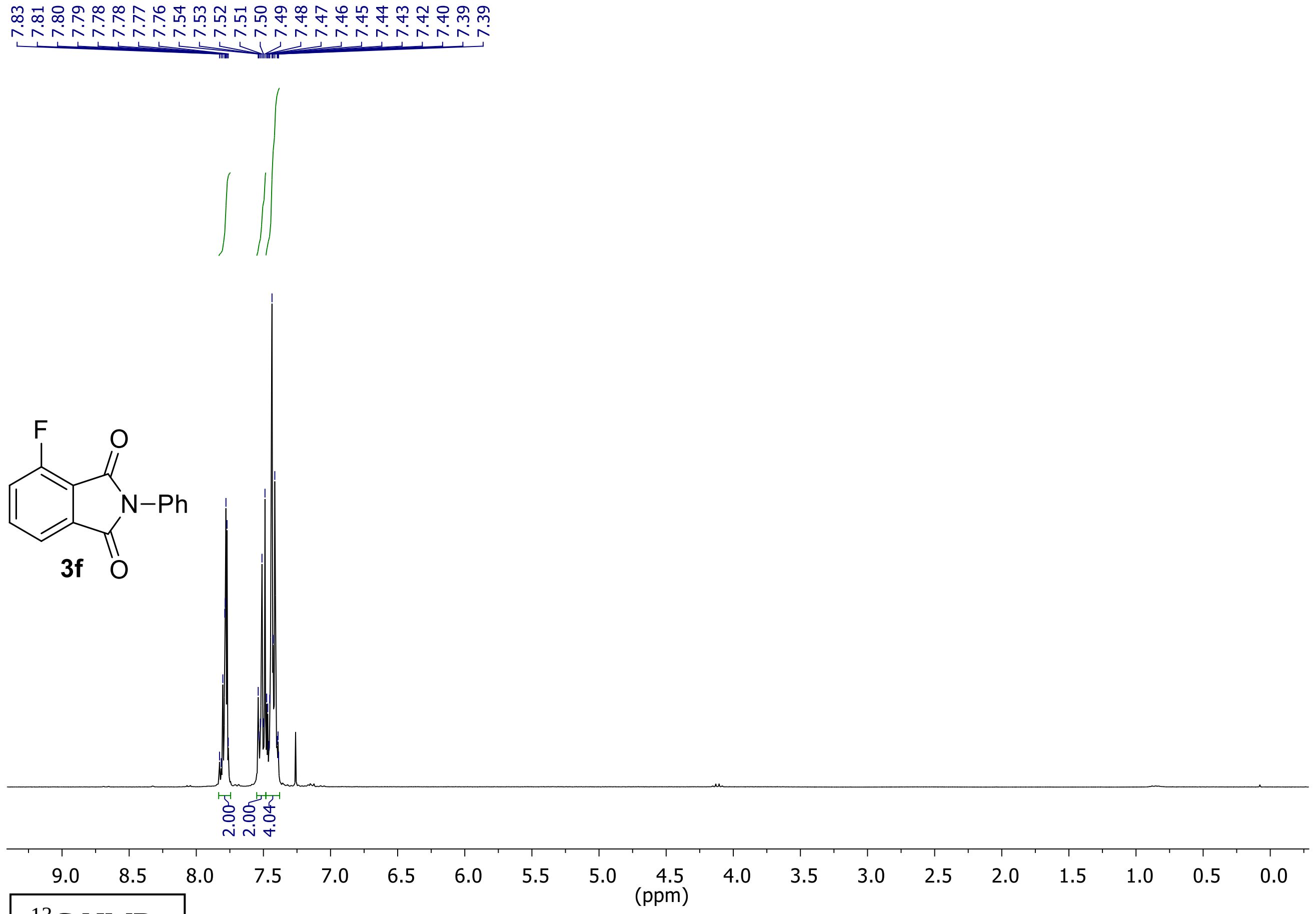

^{13}C NMR

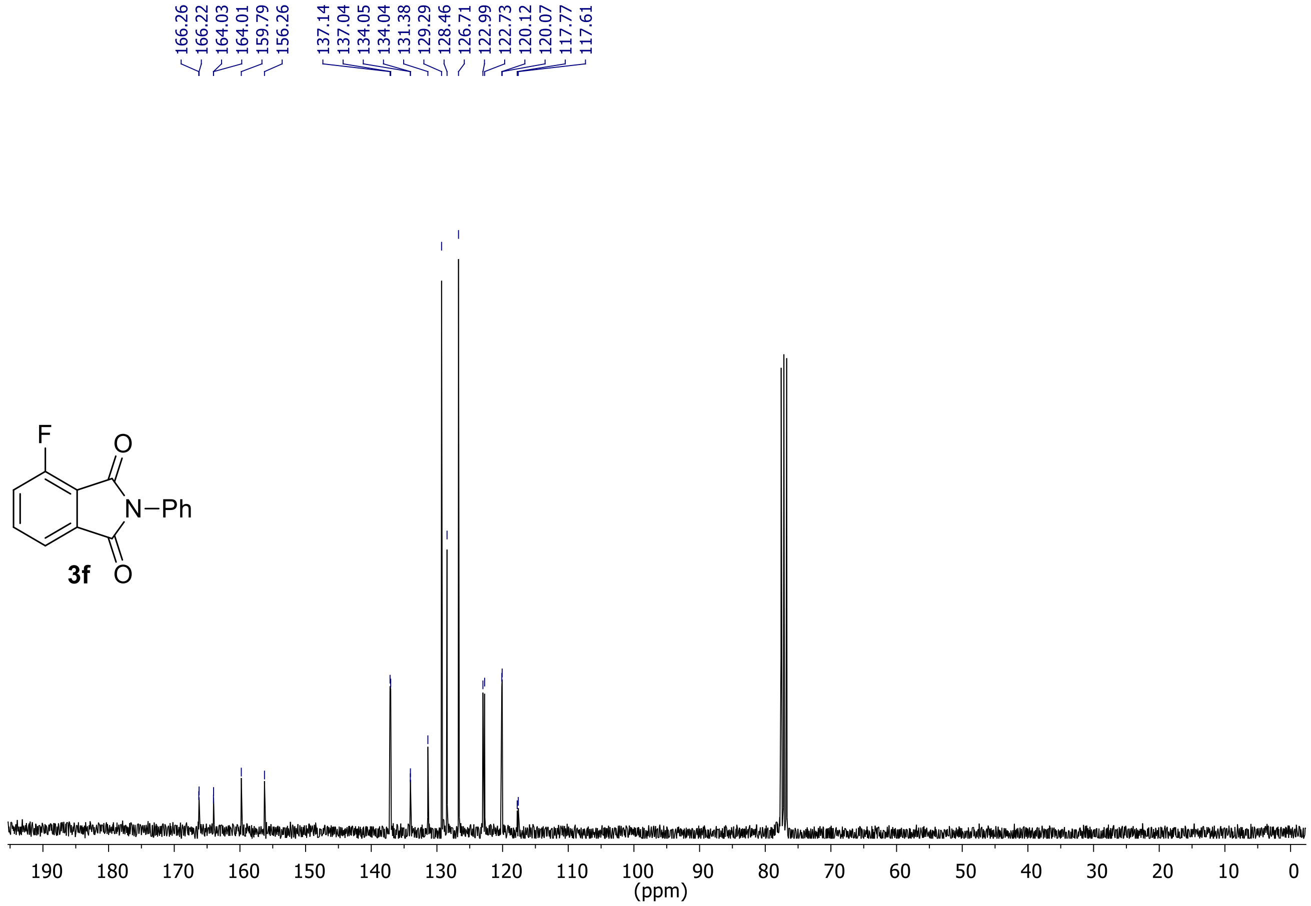

^{19}F NMR

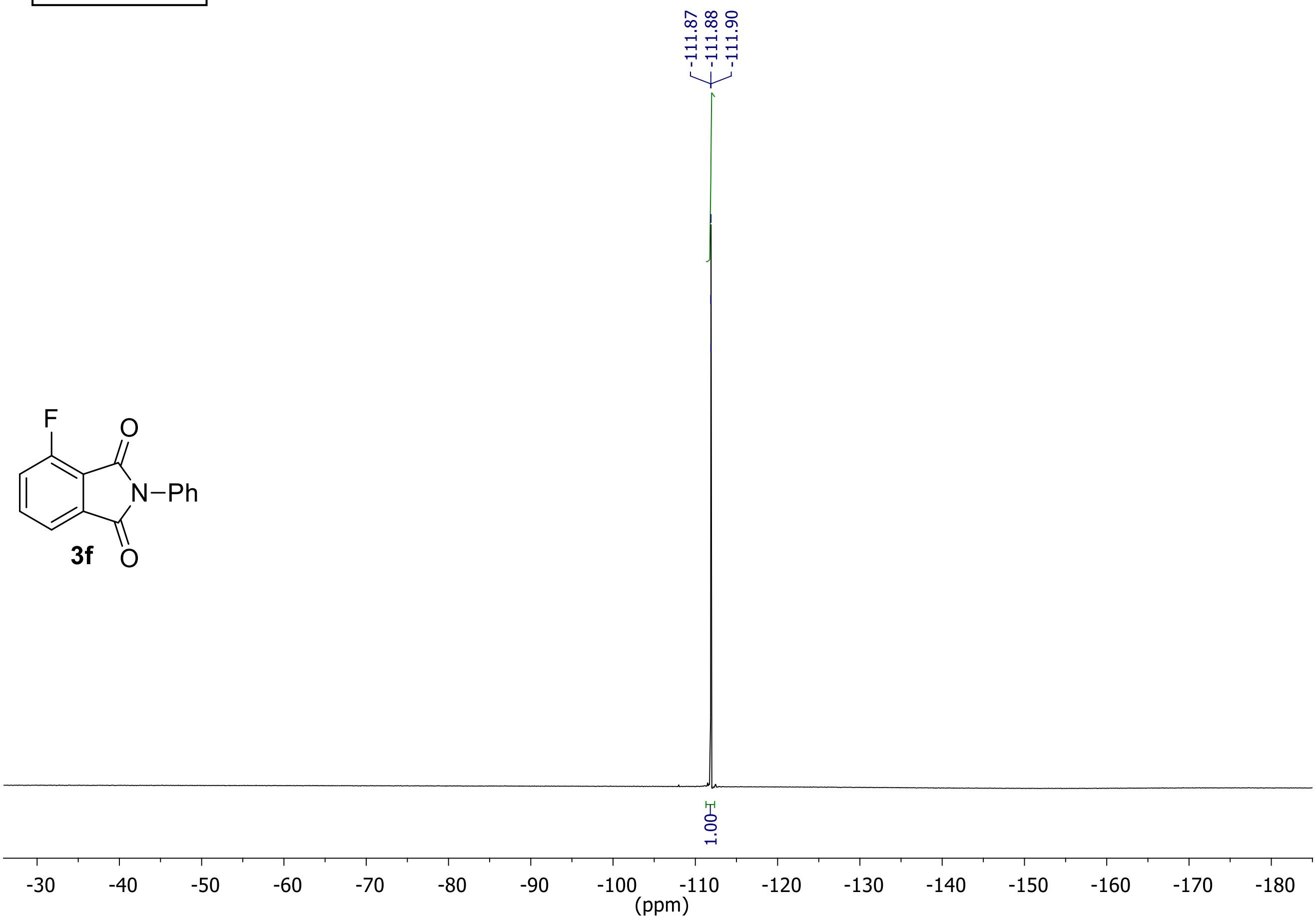

-111.87
-111.88
-111.90
F
O
N−Ph
O
3f
1.00
-30
-40
-50
-60
-70
-80
-90
-100
-110
-120
-130
-140
-150
-160
-170
-180
(ppm)

^{1}H NMR

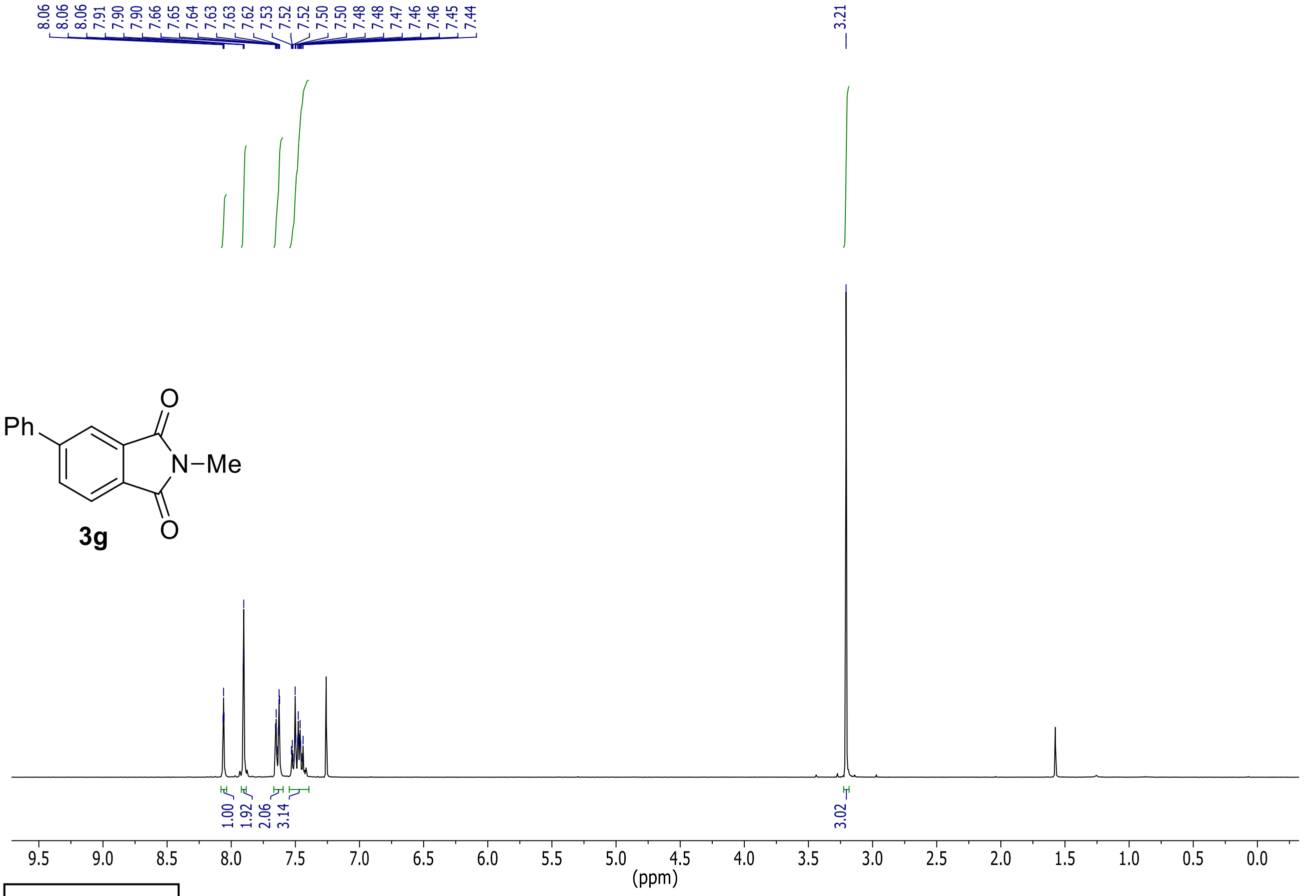

^{13}C NMR

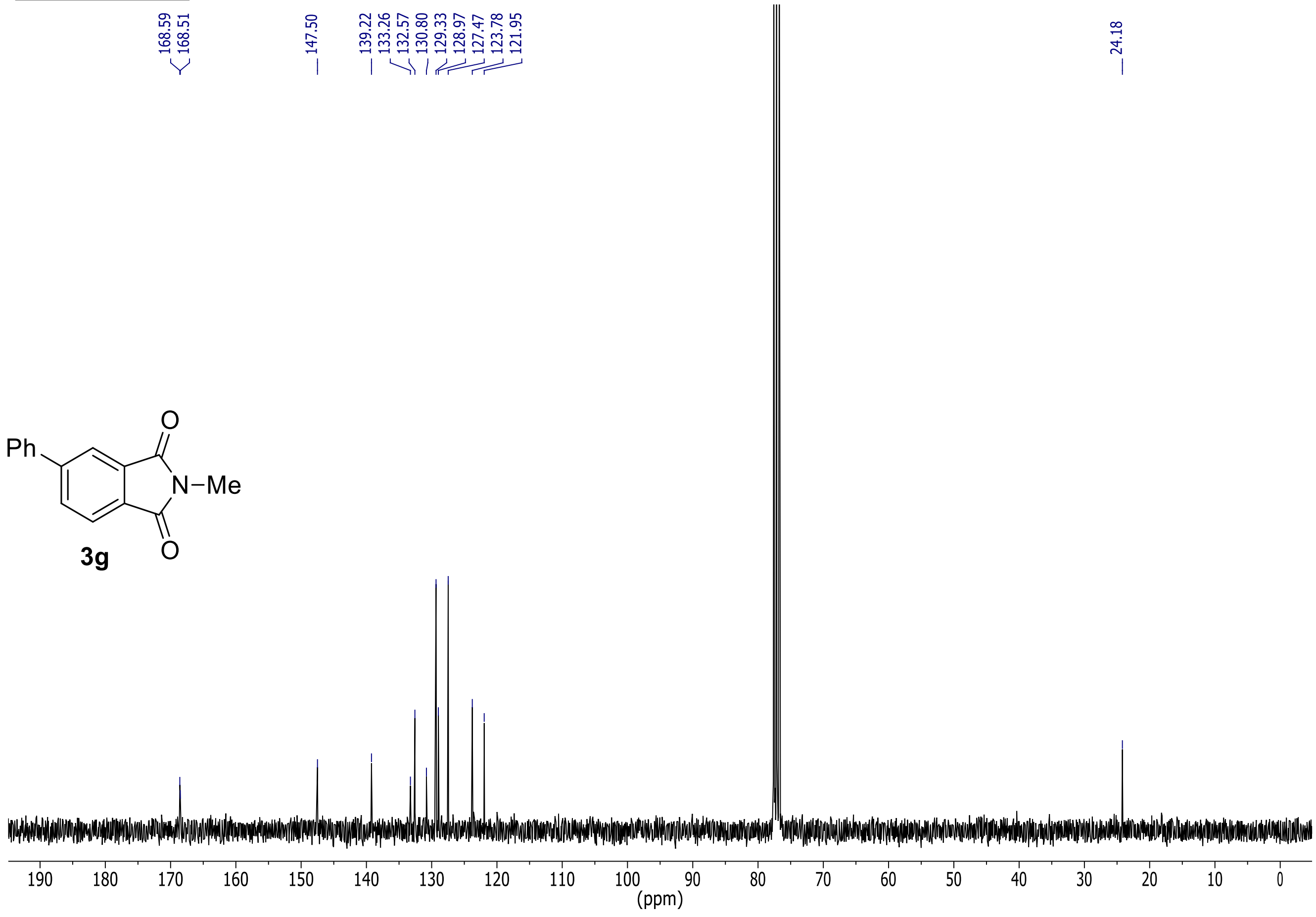

^{1}H NMR

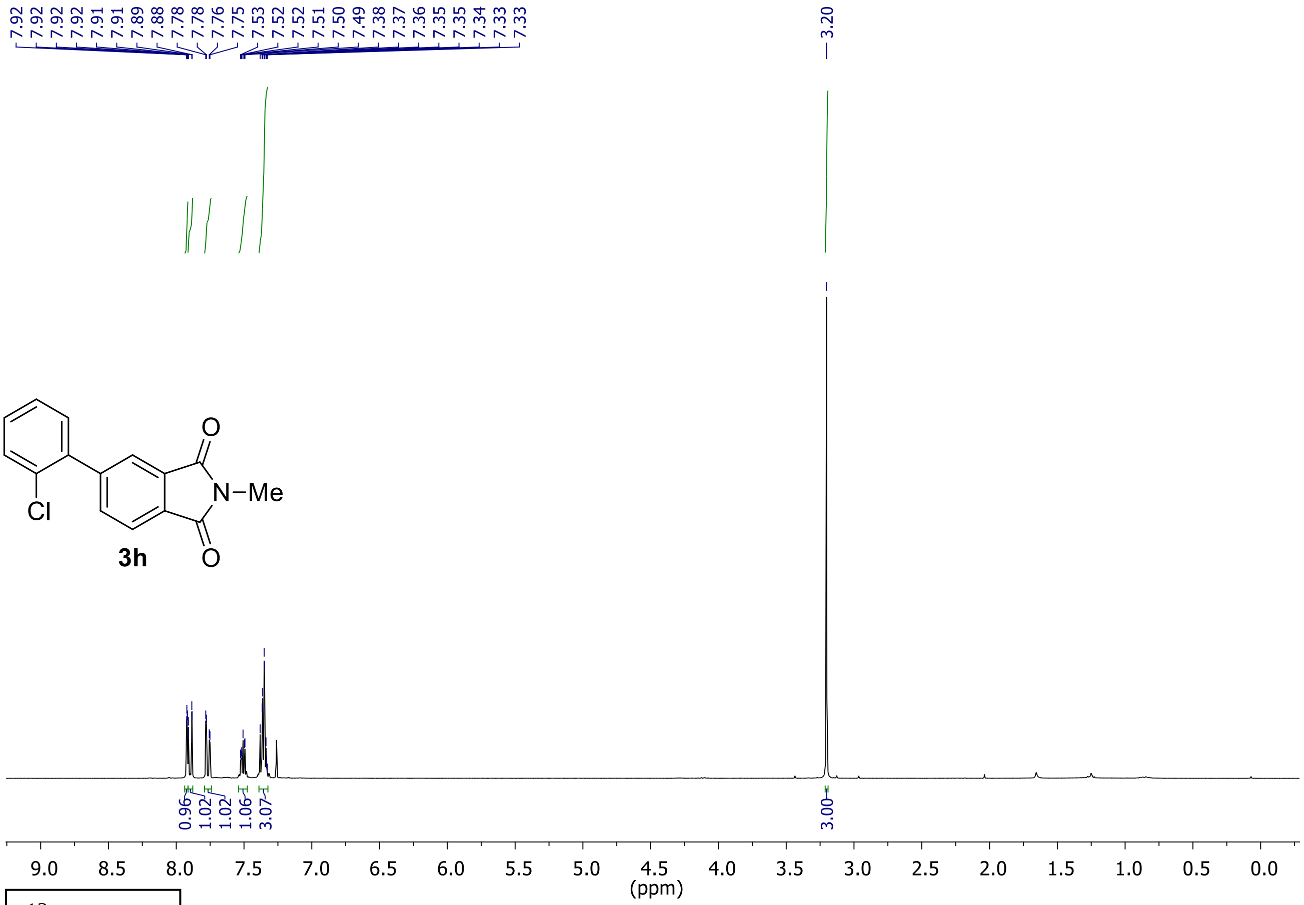

^{13}C NMR

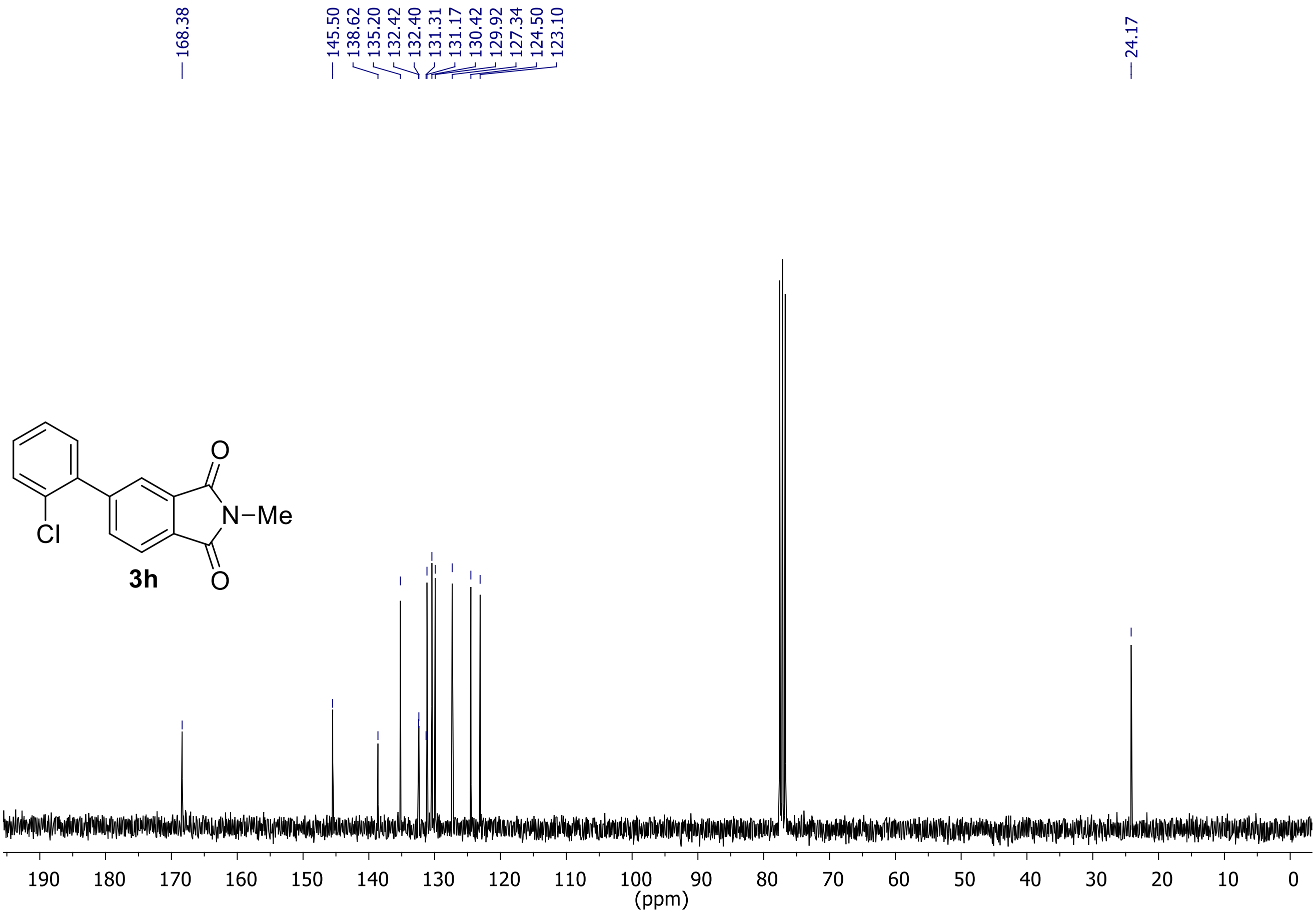

^{1}H NMR

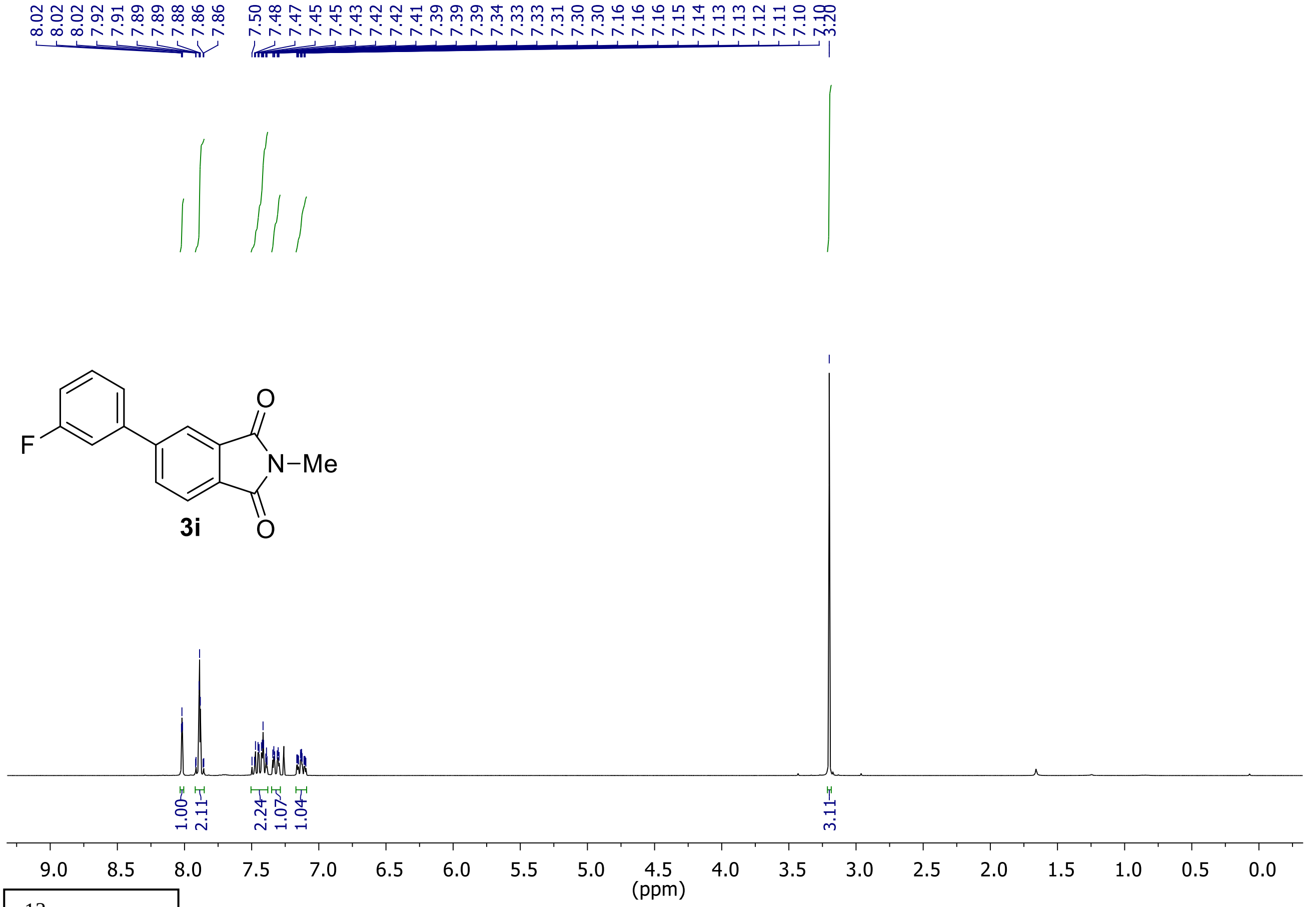

^{13}C NMR

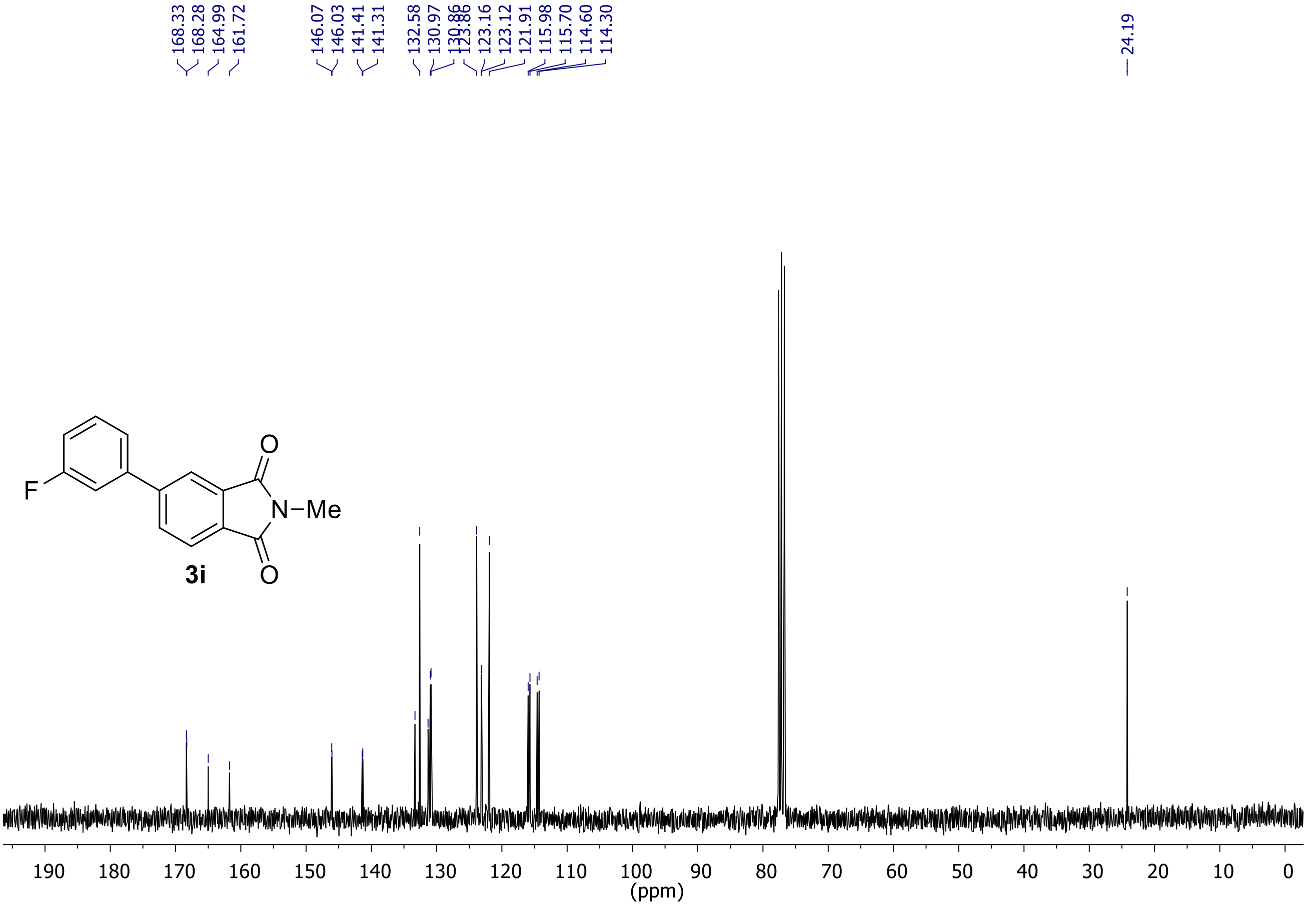

^{19}F NMR

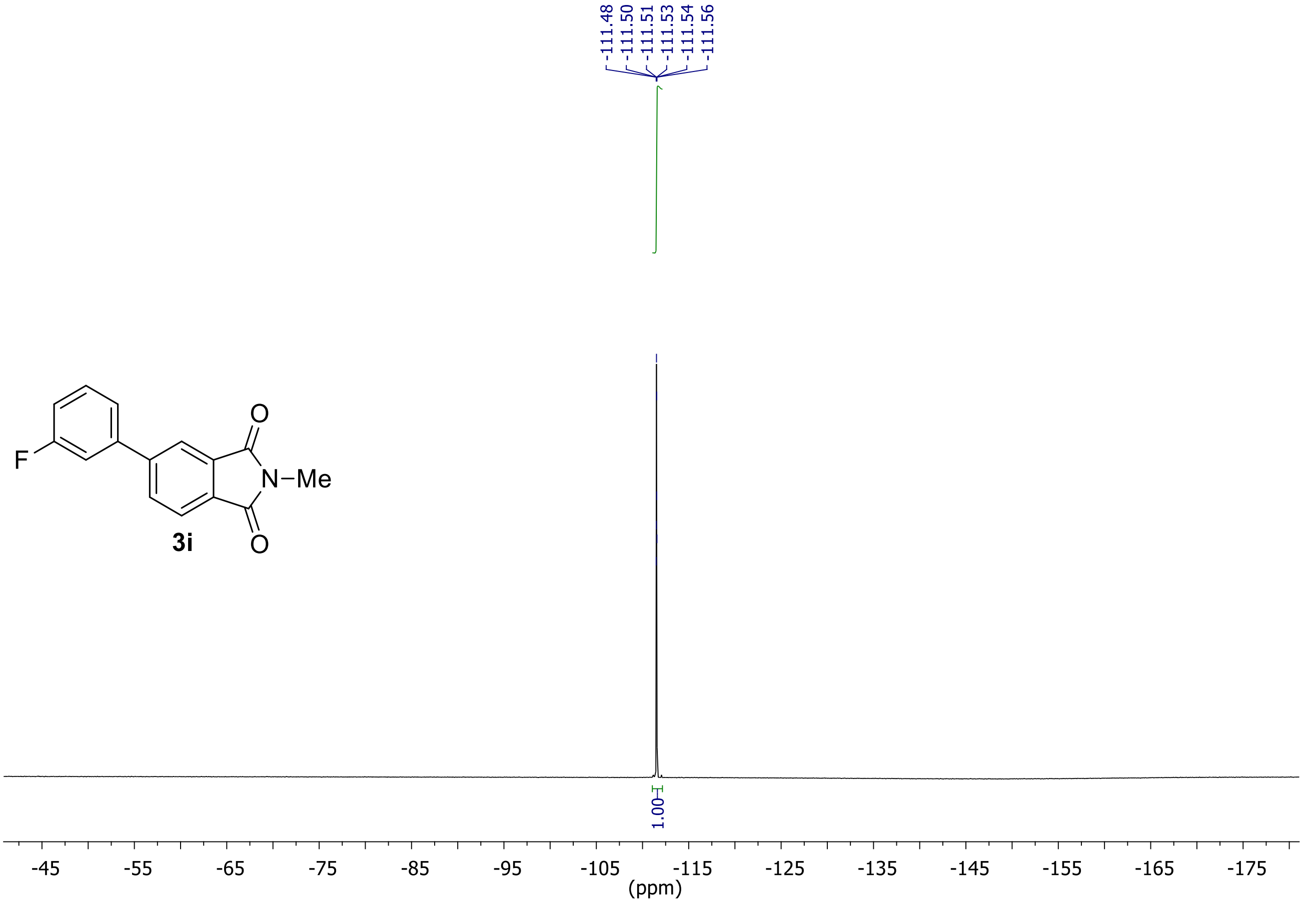

-111.48
-111.50
-111.51
-111.53
-111.54
-111.56
1.00
-45
-55
-65
-75
-85
-95
-105
-115
-125
-135
-145
-155
-165
-175
(ppm)
F
O
N–Me
O

3i

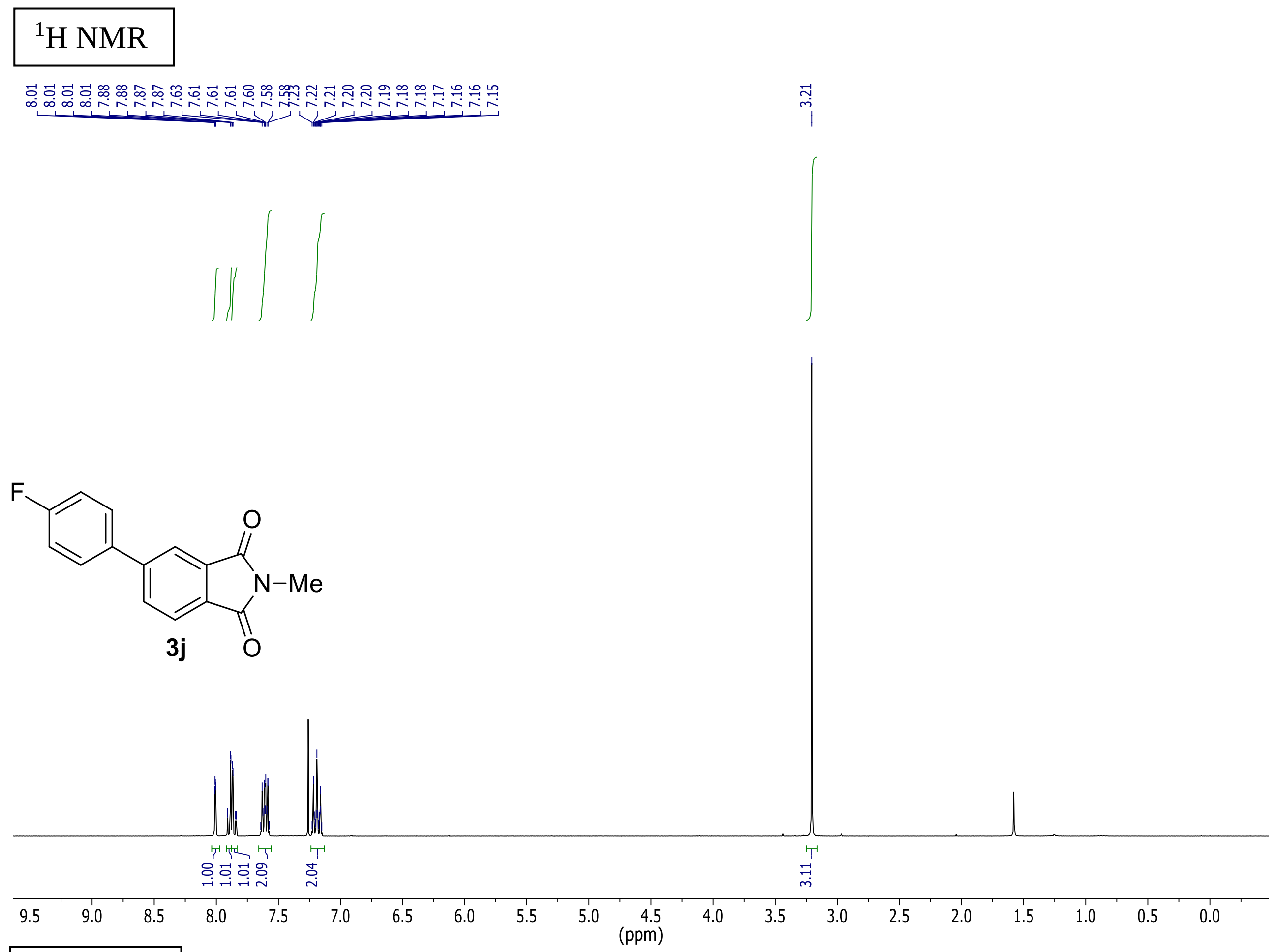

1H NMR
8.01 8.01 8.01 8.01 7.88 7.88 7.87 7.87 7.63 7.61 7.61 7.61 7.60 7.58 7.58 7.23 7.22 7.21 7.20 7.20 7.19 7.18 7.18 7.17 7.16 7.16 7.15
3.21
F
O
N−Me
O
3j
1.00 1.01 1.01 2.09 2.04
3.11
(ppm)

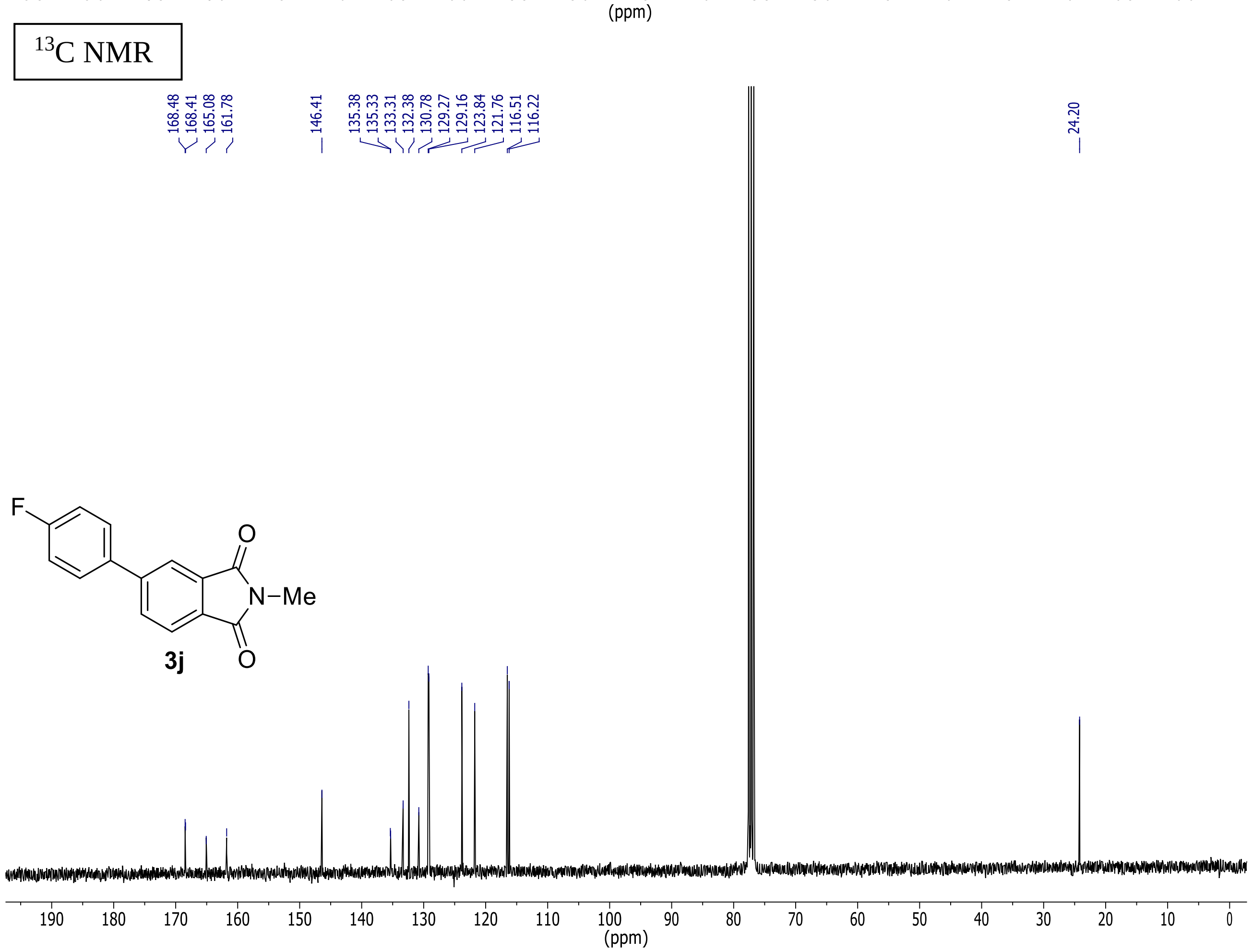

13C NMR
168.48 168.41 165.08 161.78
146.41
135.38 135.33 133.31 132.38 130.78 129.27 129.16 123.84 121.76 116.51 116.22
24.20
F
O
N−Me
O
3j
(ppm)

^{19}F NMR

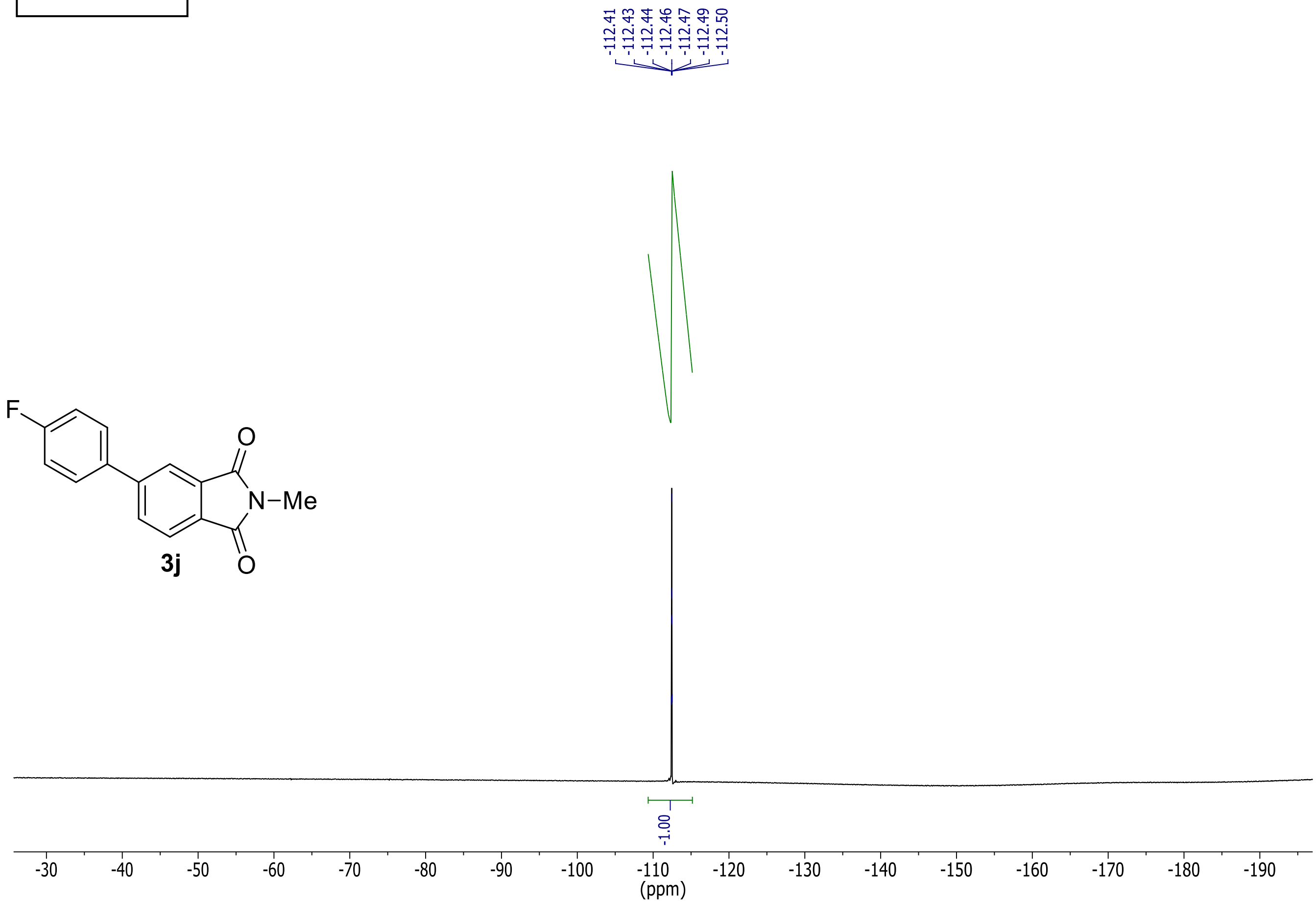

-112.41
-112.43
-112.44
-112.46
-112.47
-112.49
-112.50
-1.00
-30
-40
-50
-60
-70
-80
-90
-100
-110
-120
-130
-140
-150
-160
-170
-180
-190
(ppm)
F
O
N−Me
O

3j

^{1}H NMR

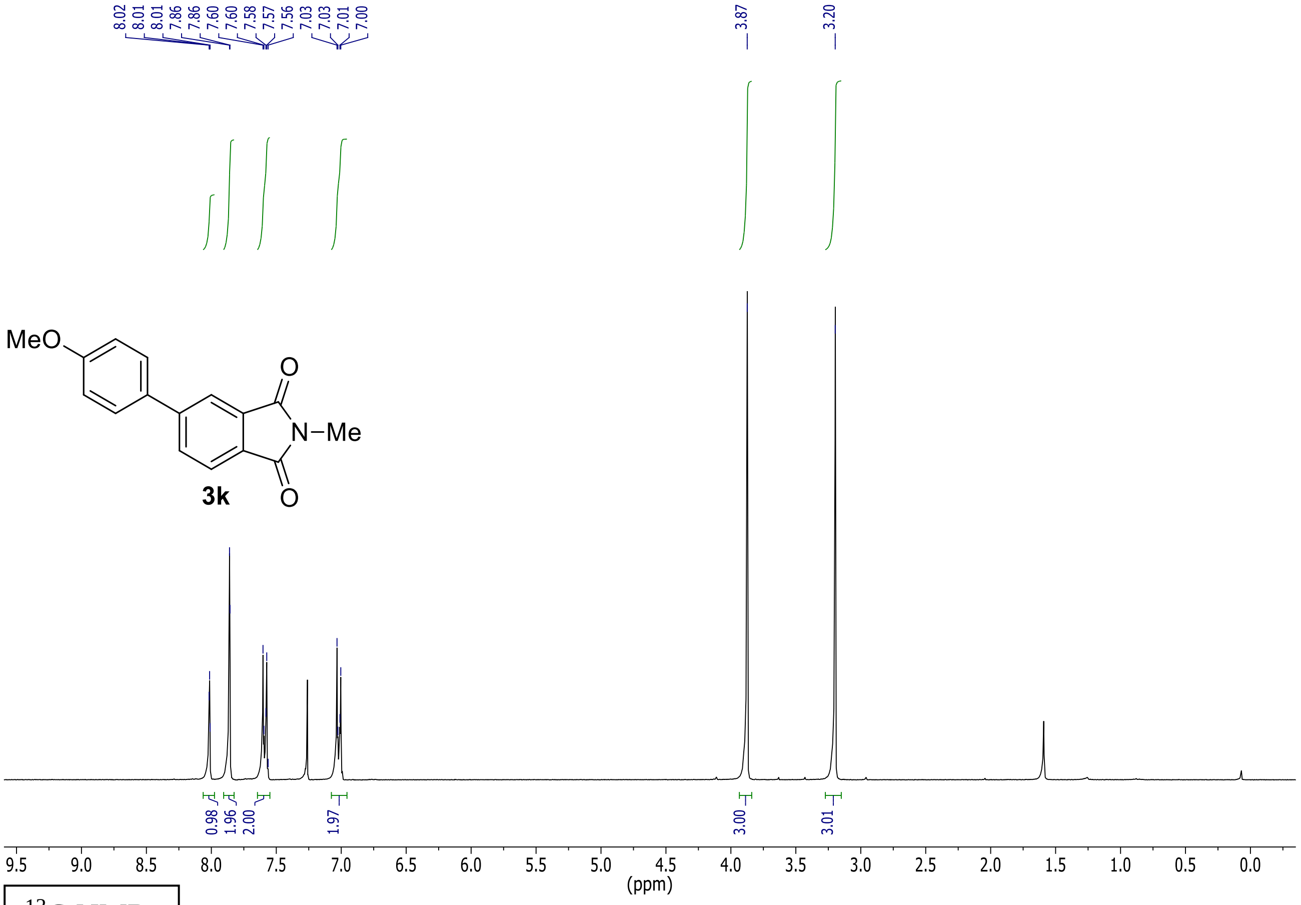

^{13}C NMR

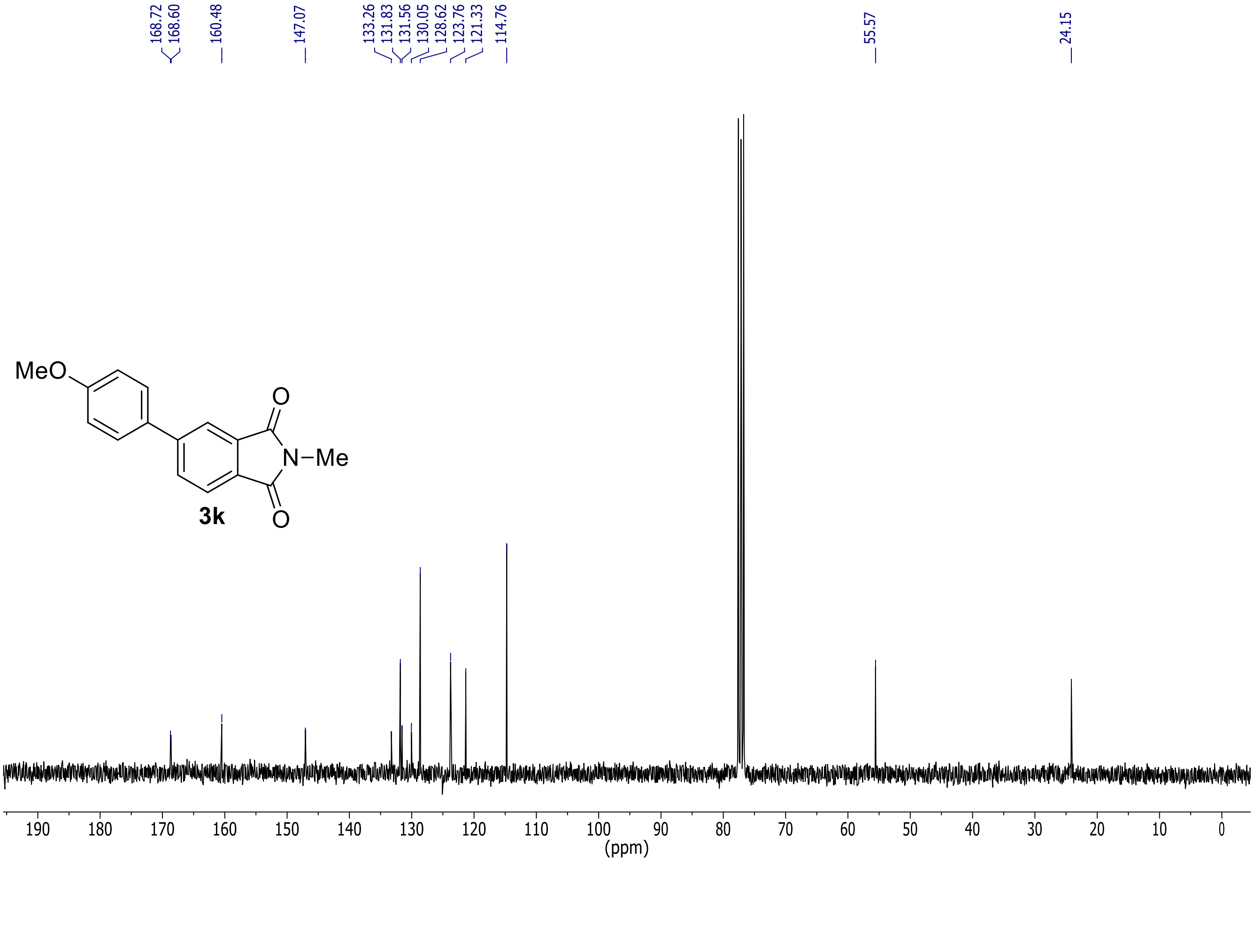

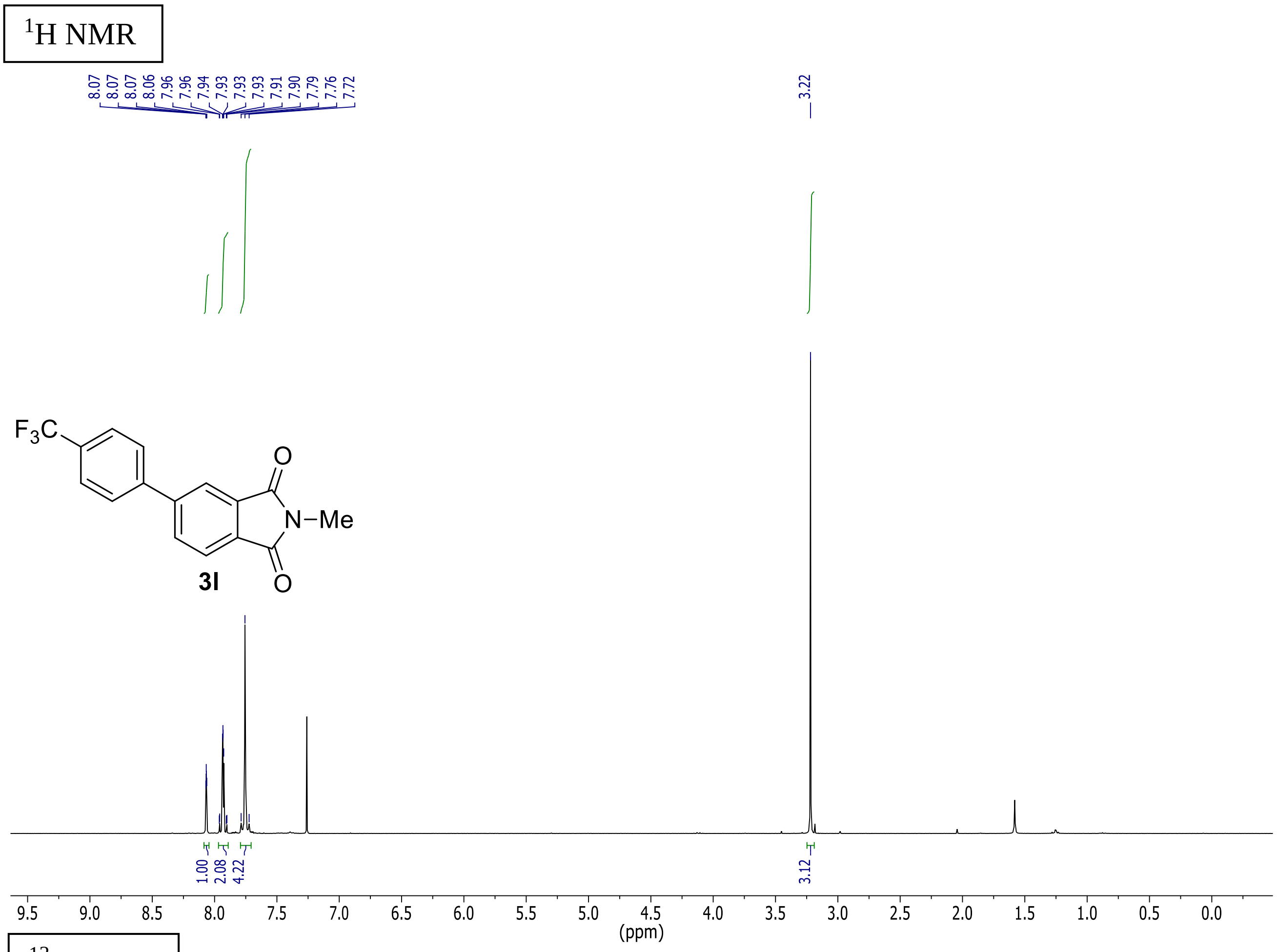

1H NMR
8.07
8.07
8.07
8.06
7.96
7.96
7.94
7.93
7.93
7.93
7.93
7.91
7.90
7.79
7.76
7.72
3.22
F3C
O
N−Me
3l
O
1.00
2.08
4.22
3.12
9.5
9.0
8.5
8.0
7.5
7.0
6.5
6.0
5.5
5.0
4.5
4.0
3.5
3.0
2.5
2.0
1.5
1.0
0.5
0.0
(ppm)

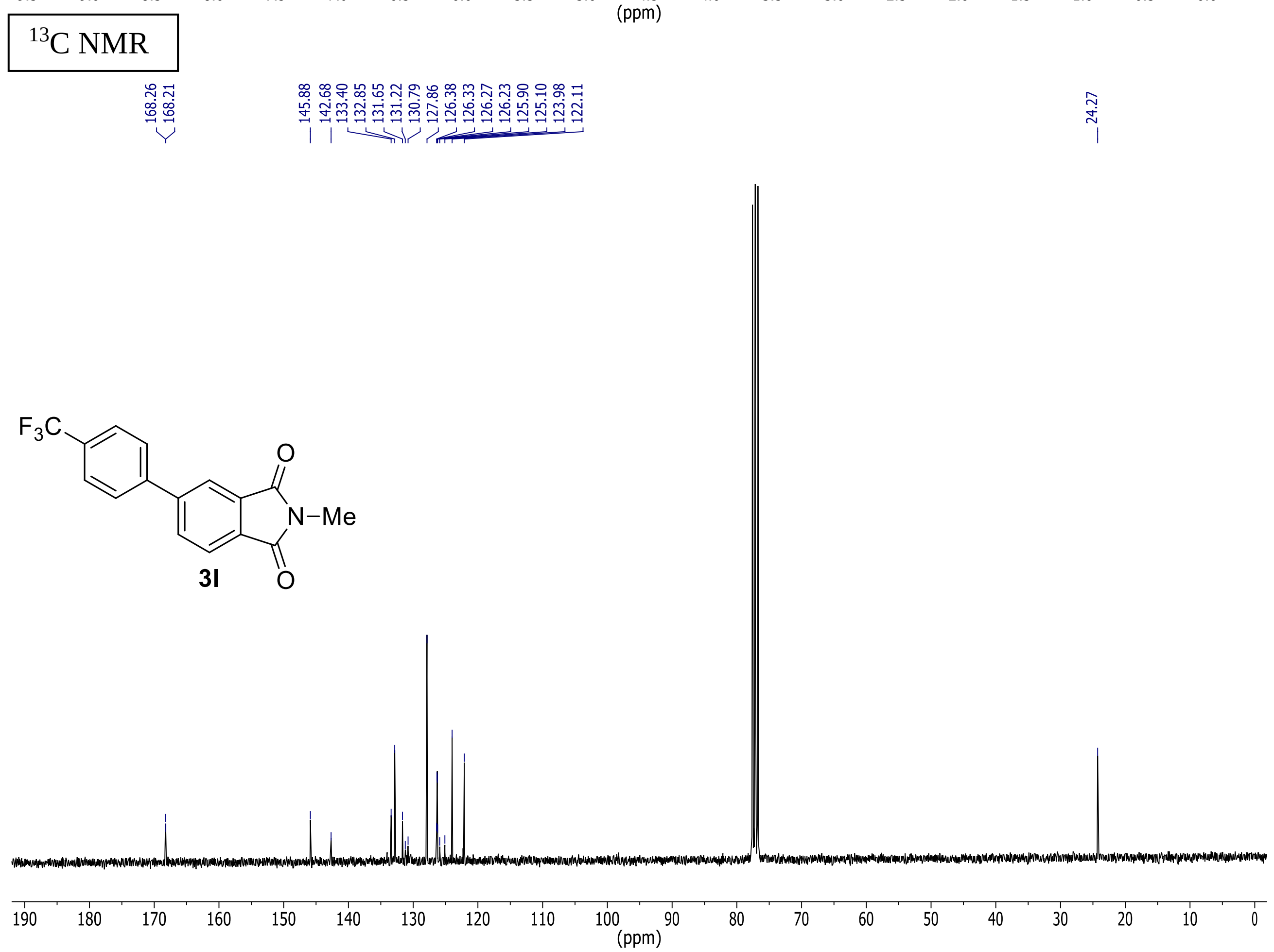

13C NMR
168.26
168.21
145.88
142.68
133.40
132.85
131.65
131.22
130.79
127.86
126.38
126.33
126.27
126.23
125.90
125.10
123.98
122.11
24.27
F3C
O
N−Me
3l
O
190
180
170
160
150
140
130
120
110
100
90
80
70
60
50
40
30
20
10
0
(ppm)

^{19}F NMR

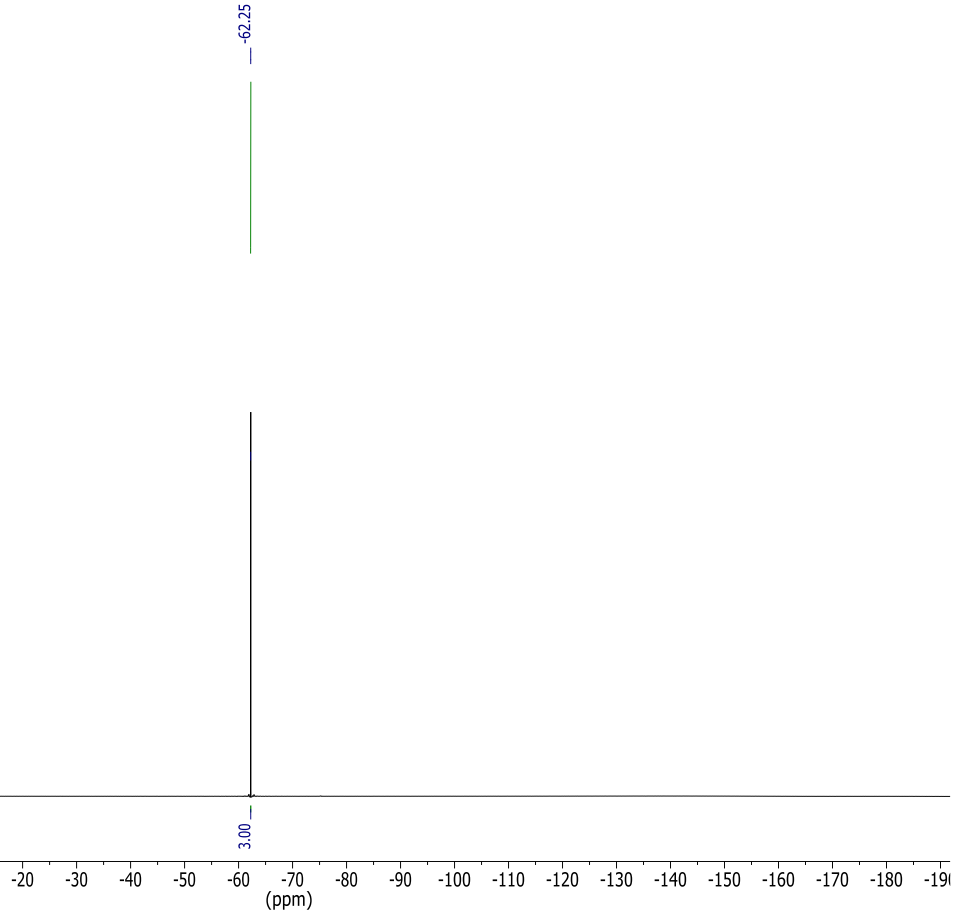

-62.25
3.00
50
40
30
20
10
0
-10
-20
-30
-40
-50
-60
-70
-80
-90
-100
-110
-120
-130
-140
-150
-160
-170
-180
(ppm)

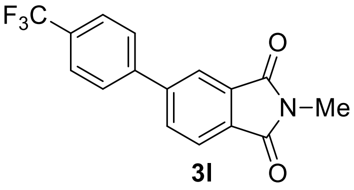

F3C
O
N−Me
O

3l

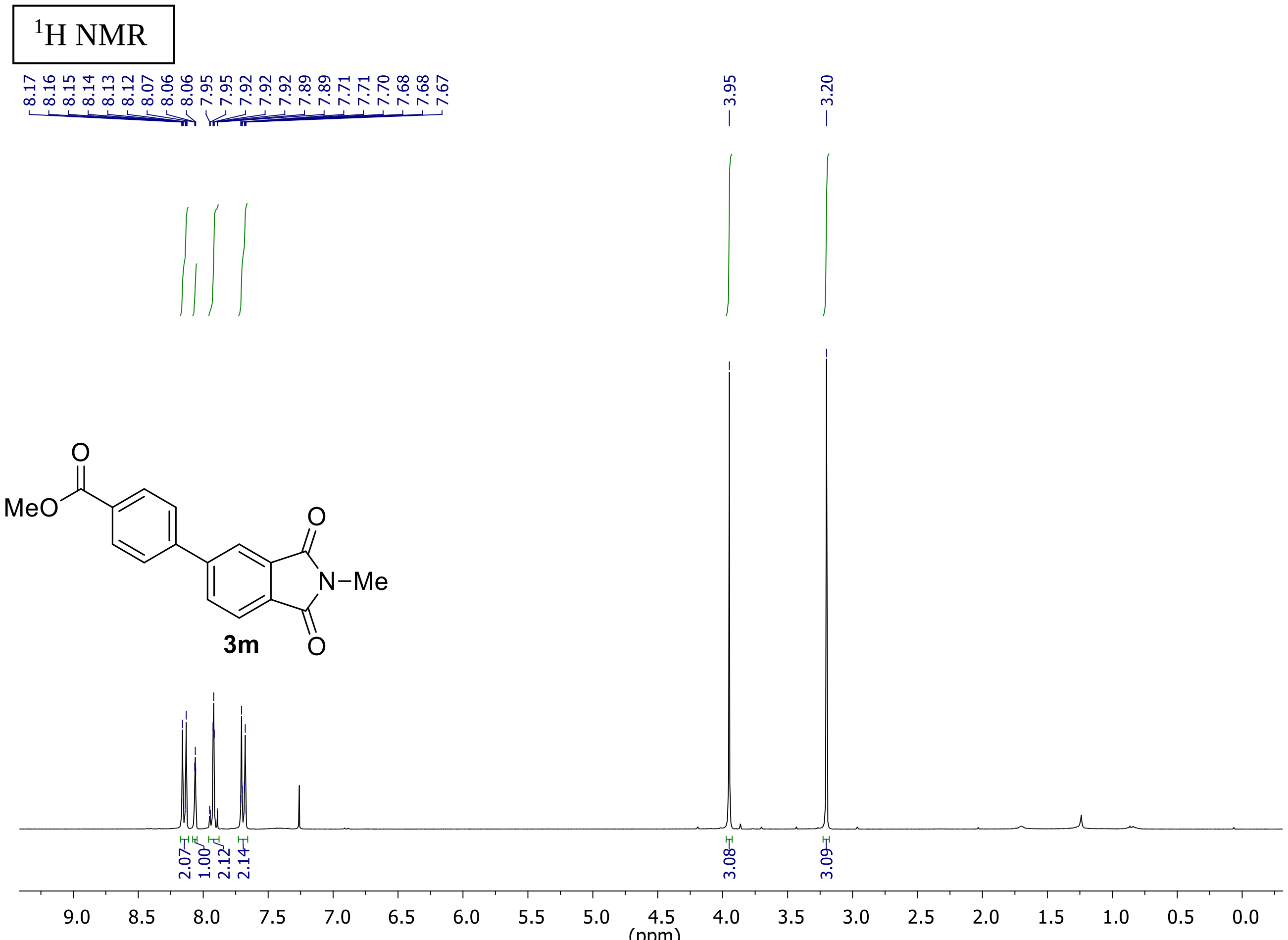

1H NMR
8.17
8.16
8.15
8.14
8.13
8.12
8.07
8.06
7.95
7.95
7.92
7.92
7.92
7.89
7.89
7.71
7.71
7.70
7.68
7.67
3.95
3.20
MeO
O
N−Me
3m
2.07
1.00
2.12
2.14
3.08
3.09
(ppm)

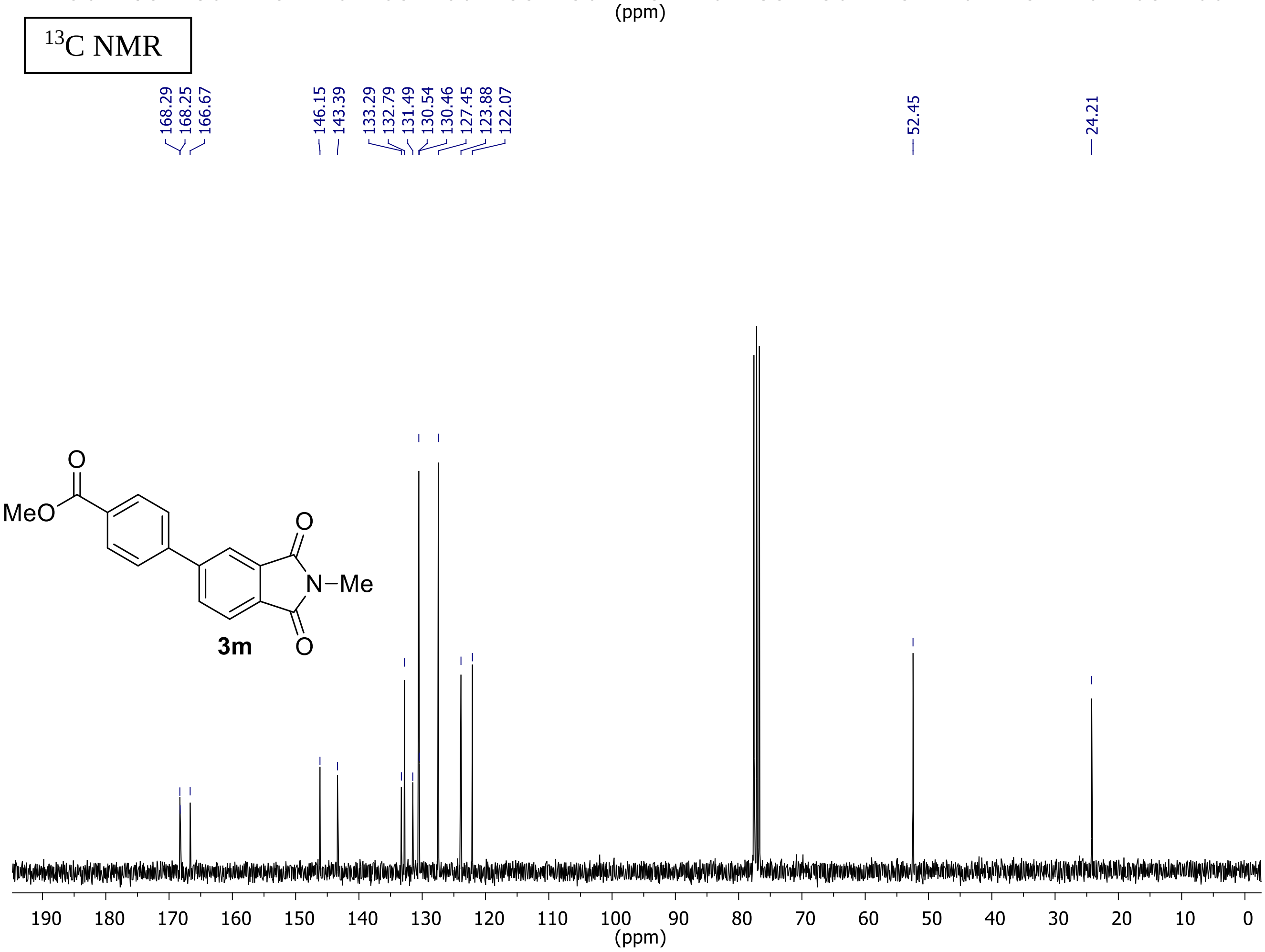

13C NMR
168.29
168.25
166.67
146.15
143.39
133.29
132.79
131.49
130.54
130.46
127.45
123.88
122.07
52.45
24.21
MeO
O
N−Me
3m
(ppm)

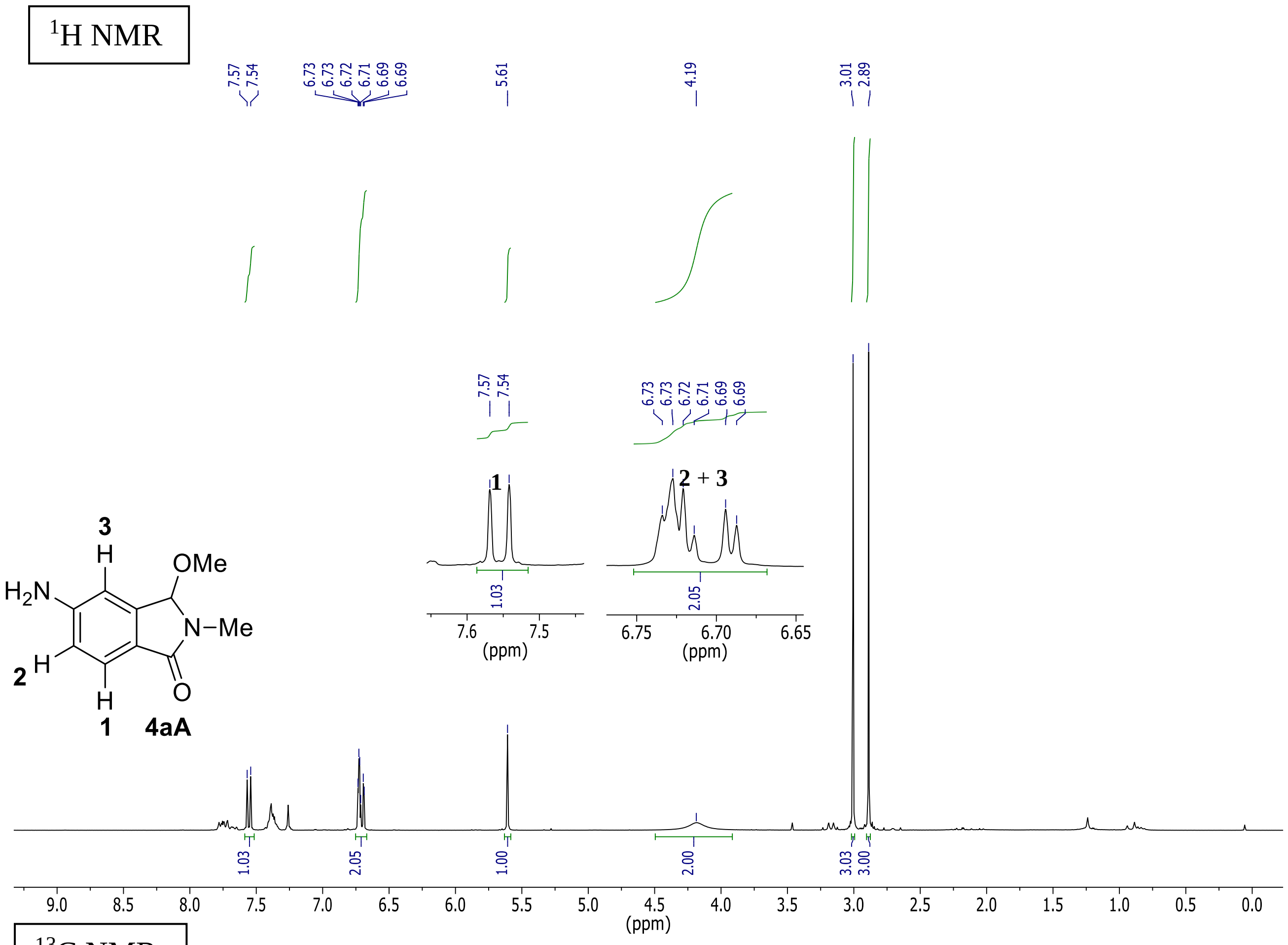

1H NMR
7.57
7.54
6.73
6.73
6.72
6.71
6.69
6.69
5.61
4.19
3.01
2.89
1
2 + 3
1.03
2.05
7.6
7.5
(ppm)
6.75
6.70
6.65
H2N
OMe
N−Me
O
4aA
1.03
2.05
1.00
2.00
3.03
3.00
9.0
8.5
8.0
7.5
7.0
6.5
6.0
5.5
5.0
4.5
4.0
3.5
3.0
2.5
2.0
1.5
1.0
0.5
0.0

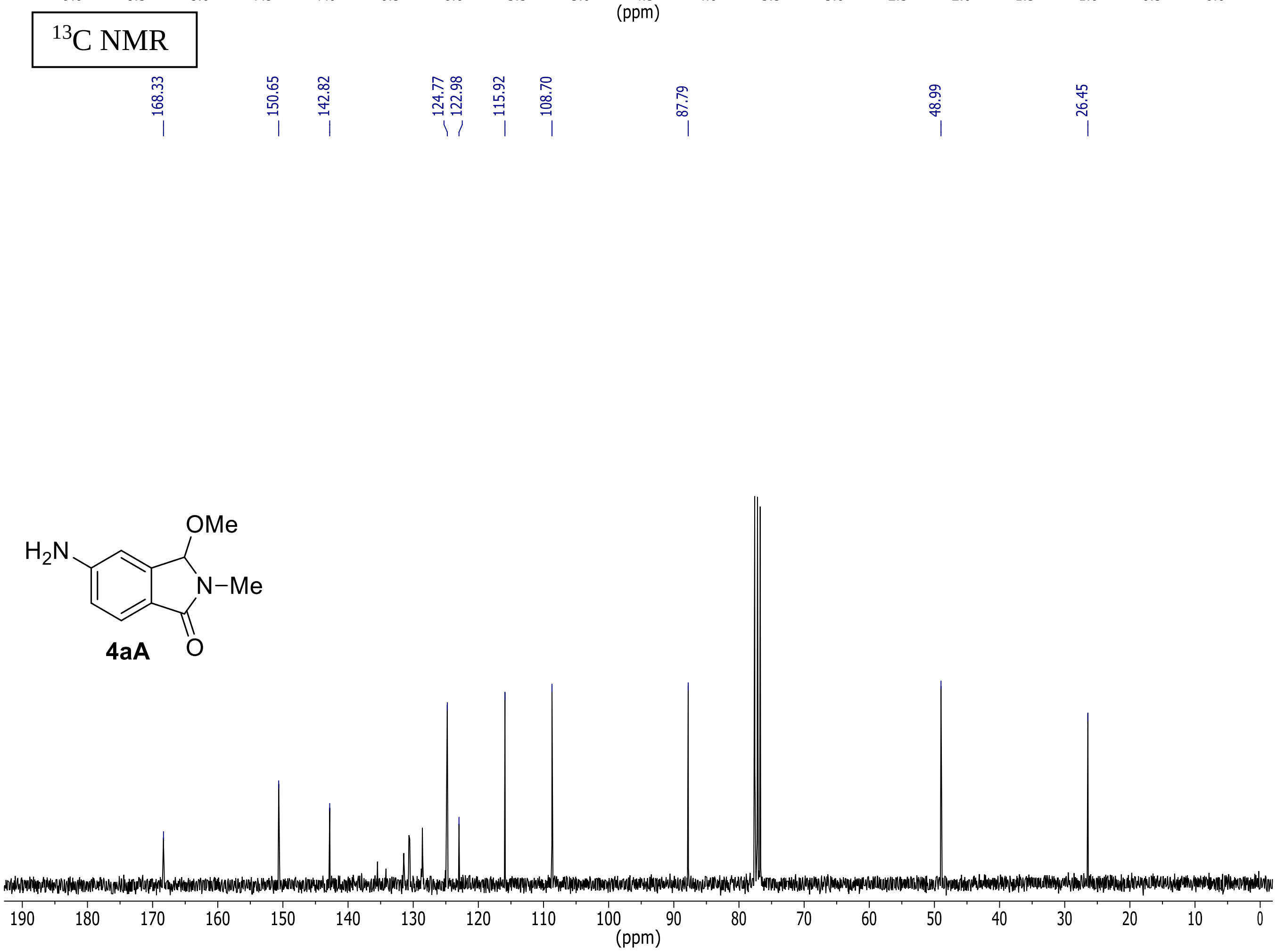

13C NMR
168.33
150.65
142.82
124.77
122.98
115.92
108.70
87.79
48.99
26.45
H2N
OMe
N−Me
O
4aA
190
180
170
160
150
140
130
120
110
100
90
80
70
60
50
40
30
20
10
0
(ppm)

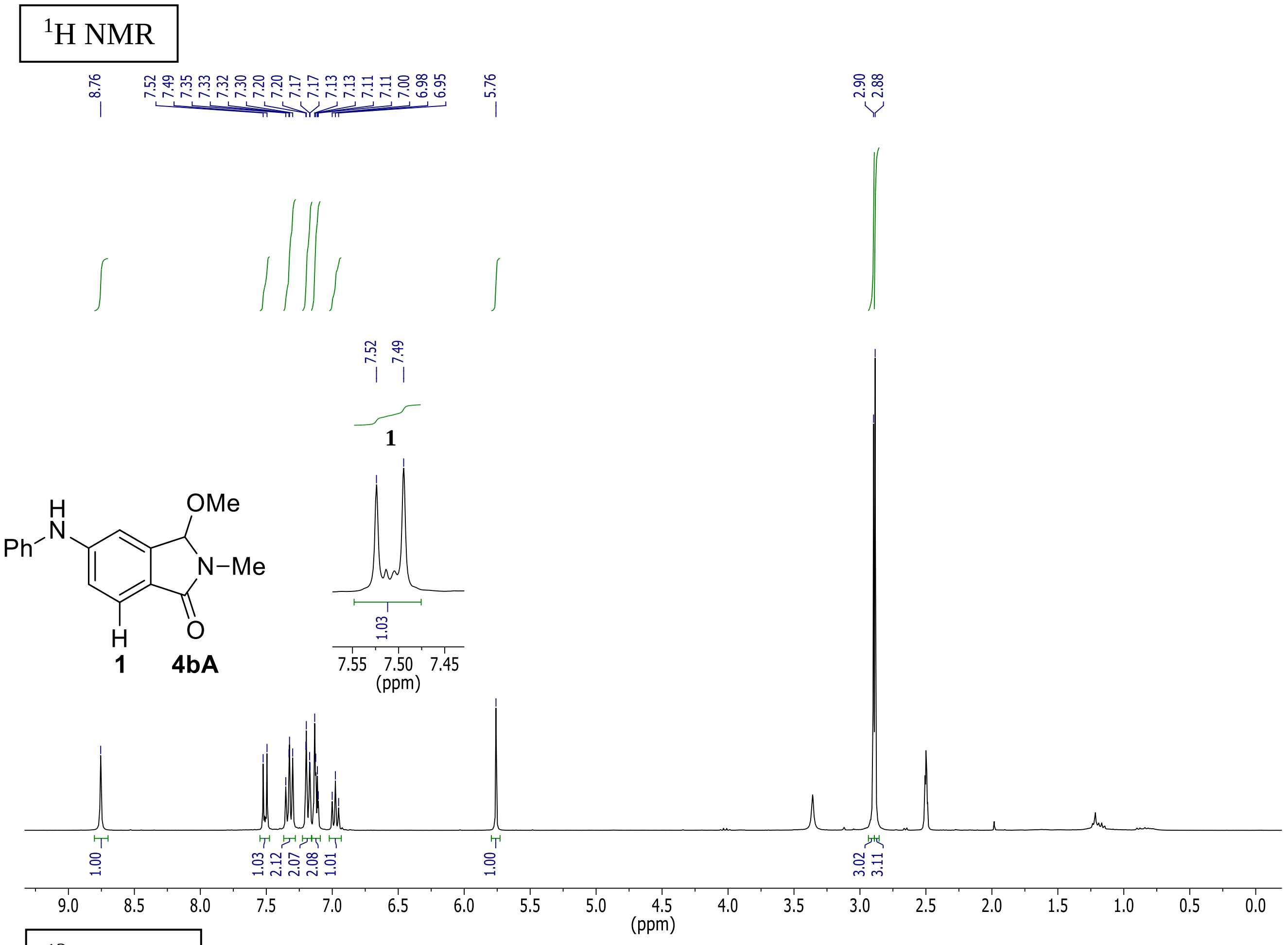

1H NMR
8.76
7.52 7.49 7.35 7.33 7.32 7.30 7.20 7.20 7.17 7.17 7.13 7.13 7.11 7.11 7.00 6.98 6.95
5.76
2.90 2.88
7.52 7.49
1.03
7.55 7.50 7.45
(ppm)
1.00 1.03 2.12 2.07 2.08 1.01 1.00 3.02 3.11
9.0 8.5 8.0 7.5 7.0 6.5 6.0 5.5 5.0 4.5 4.0 3.5 3.0 2.5 2.0 1.5 1.0 0.5 0.0
(ppm)
Ph
H
N
OMe
N−Me
O
H
1
4bA

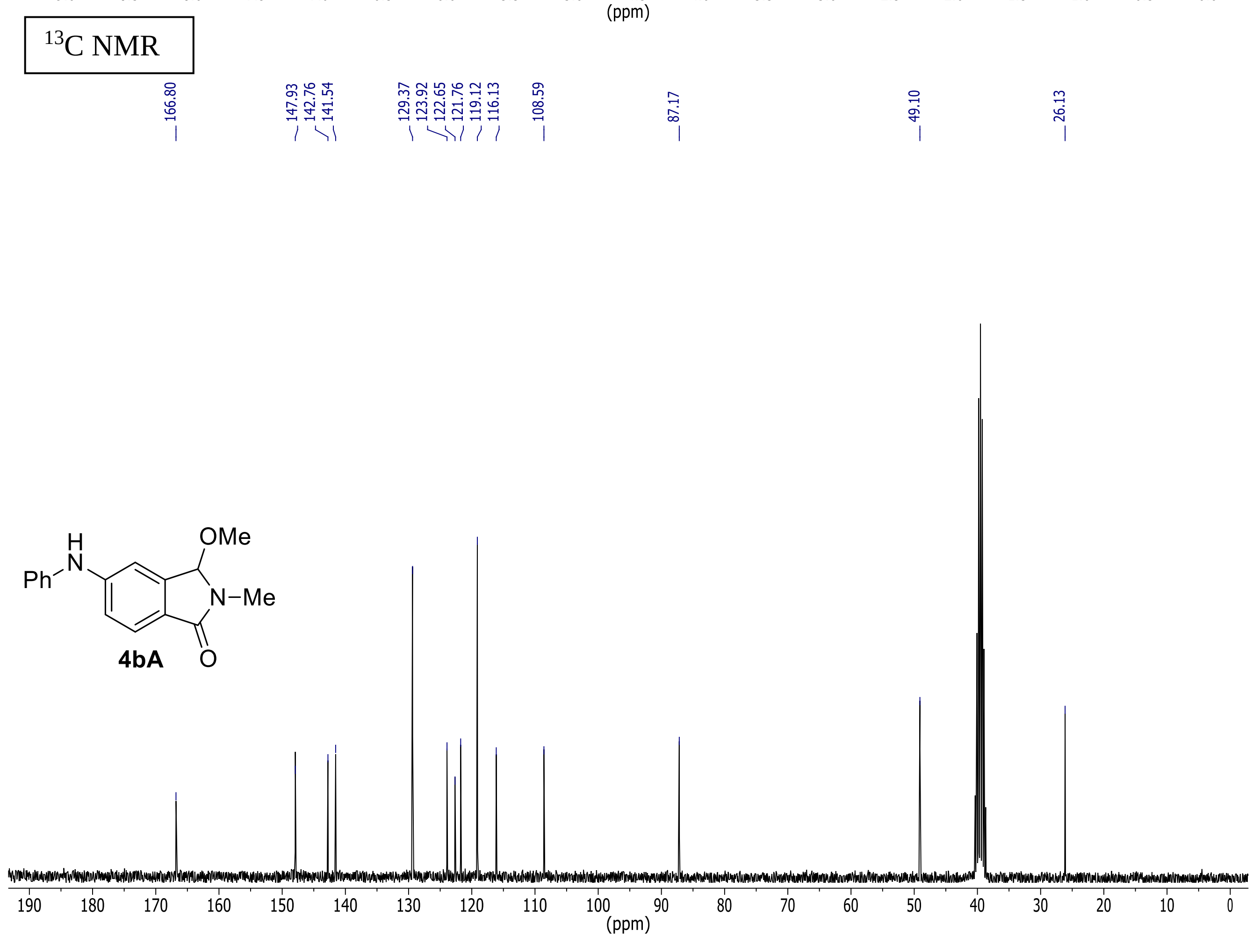

13C NMR
166.80
147.93 142.76 141.54
129.37 123.92 122.65 121.76 119.12 116.13
108.59
87.17
49.10
26.13
190 180 170 160 150 140 130 120 110 100 90 80 70 60 50 40 30 20 10 0
(ppm)
Ph
H
N
OMe
N−Me
O
4bA

^{1}H NMR

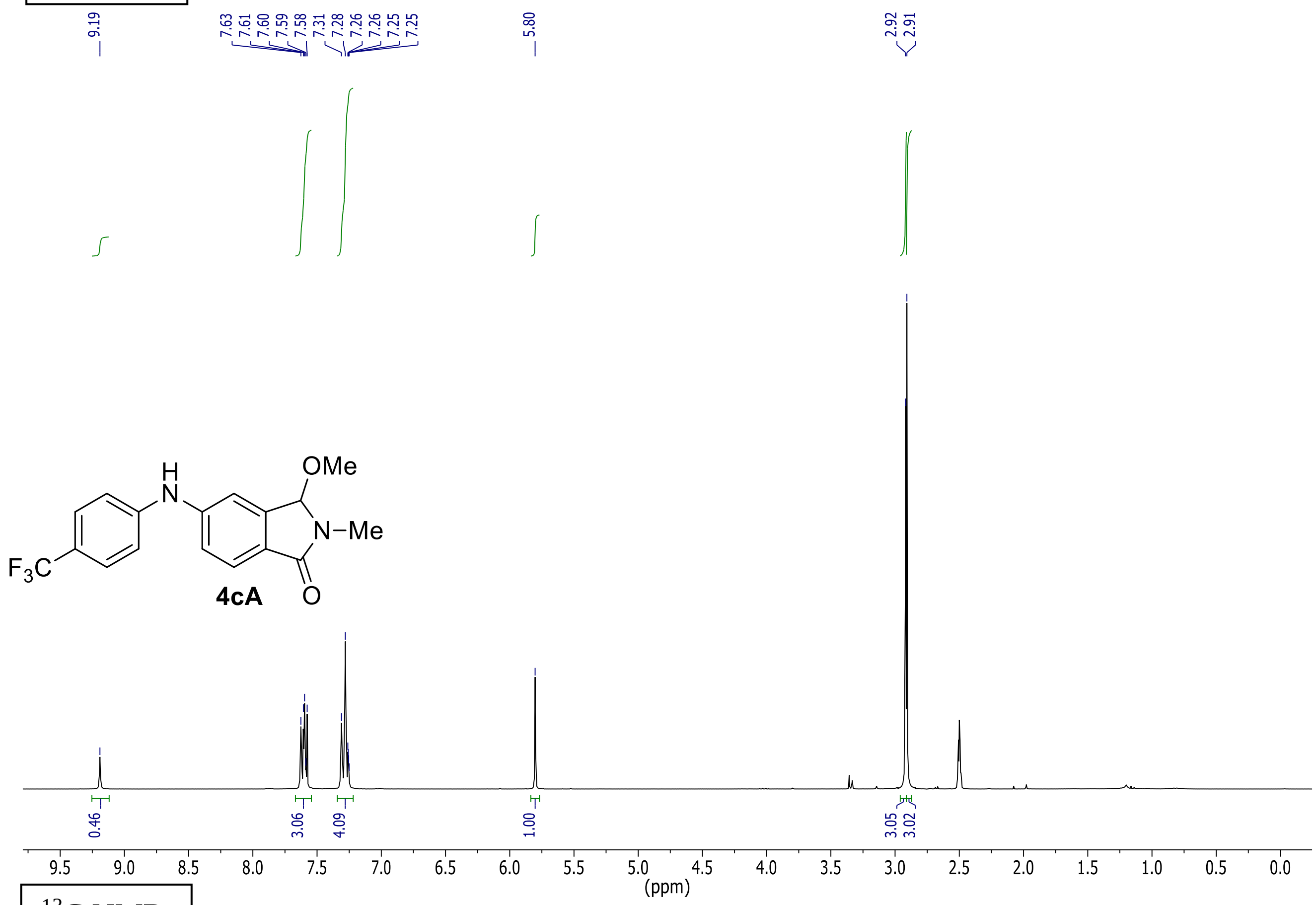

^{13}C NMR

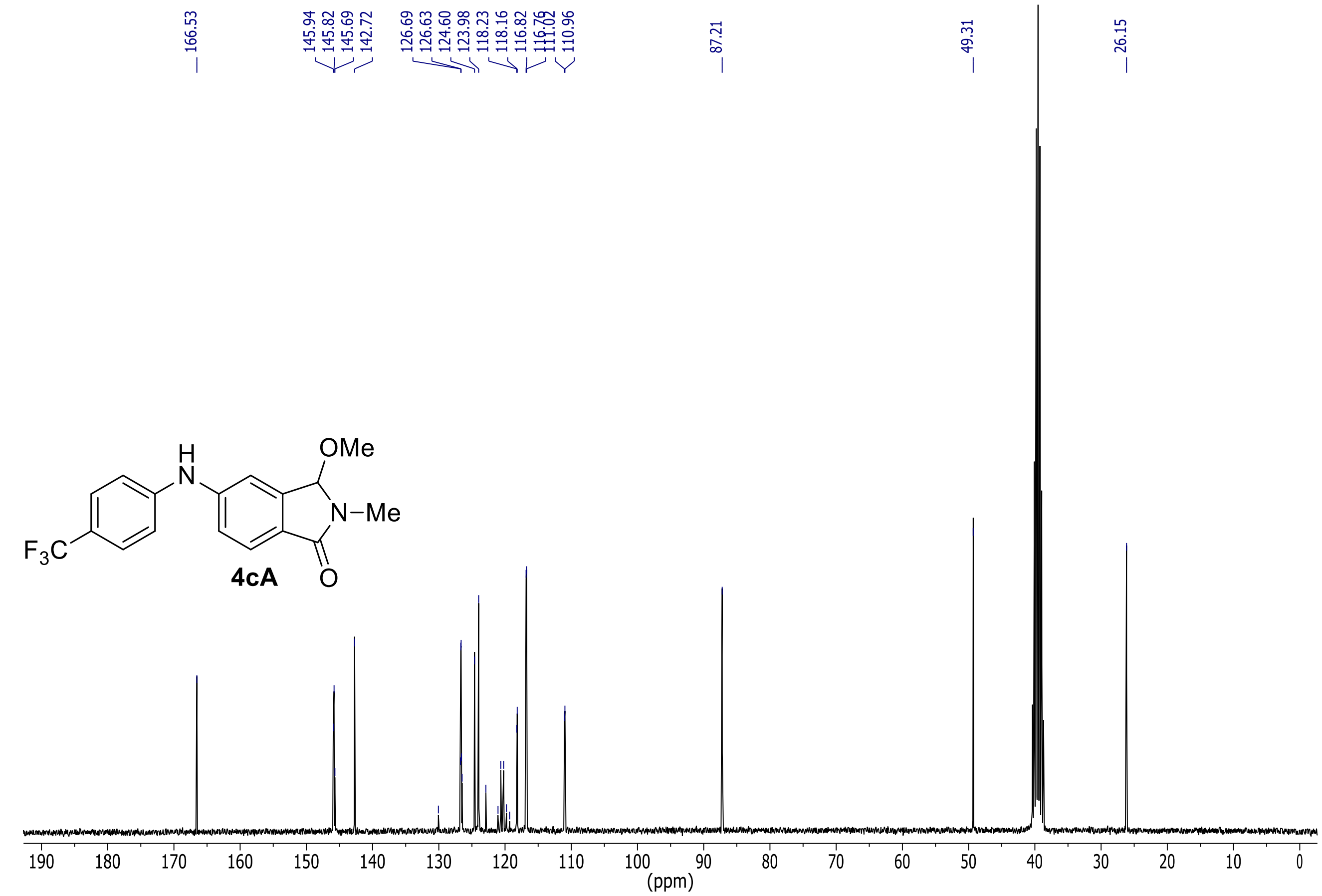

^{19}F NMR

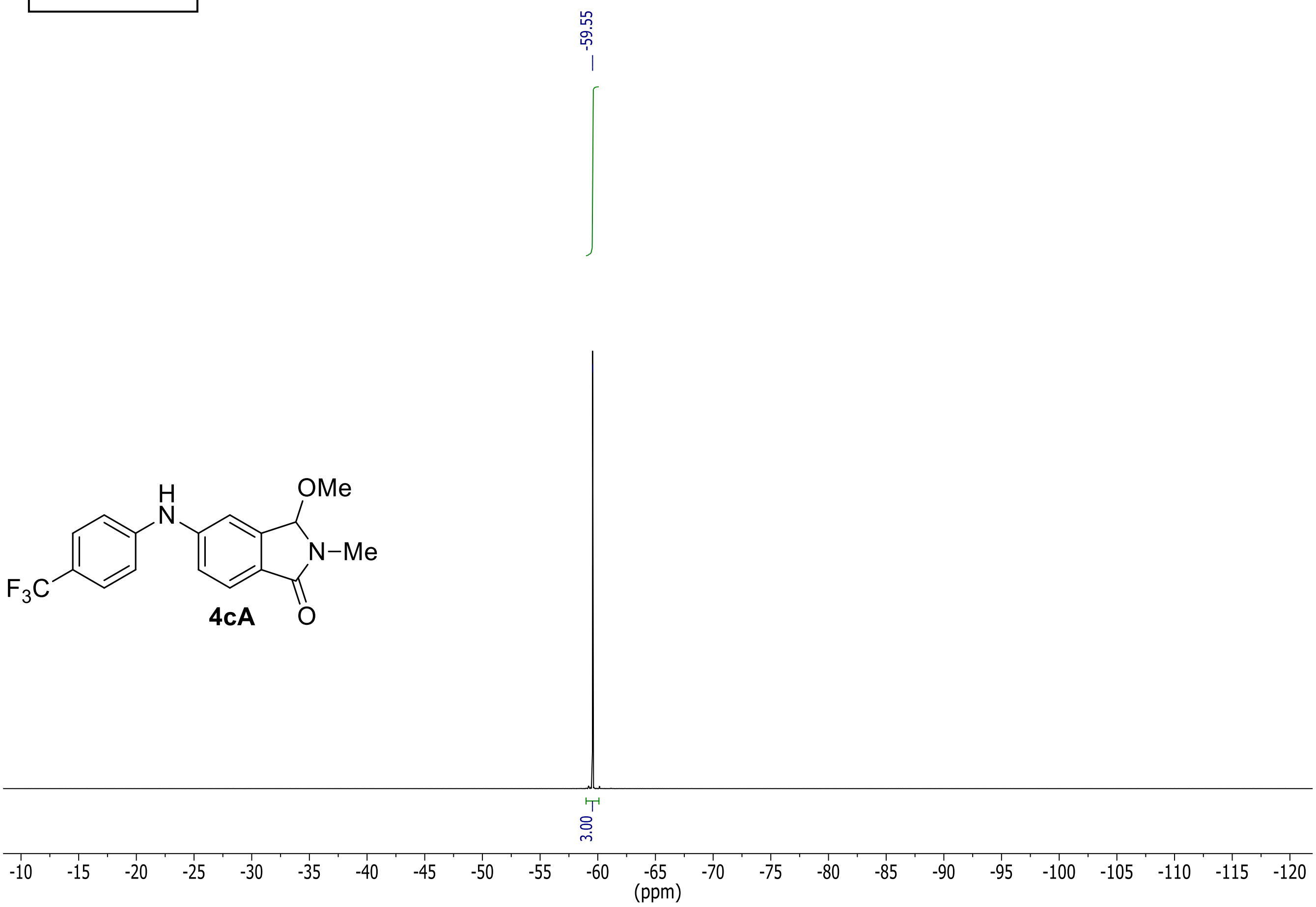
-59.55
H
N
OMe
N−Me
F3C
O
3.00
-10
-15
-20
-25
-30
-35
-40
-45
-50
-55
-60
-65
-70
-75
-80
-85
-90
-95
-100
-105
-110
-115
-120
(ppm)

4cA

NOESY NMR

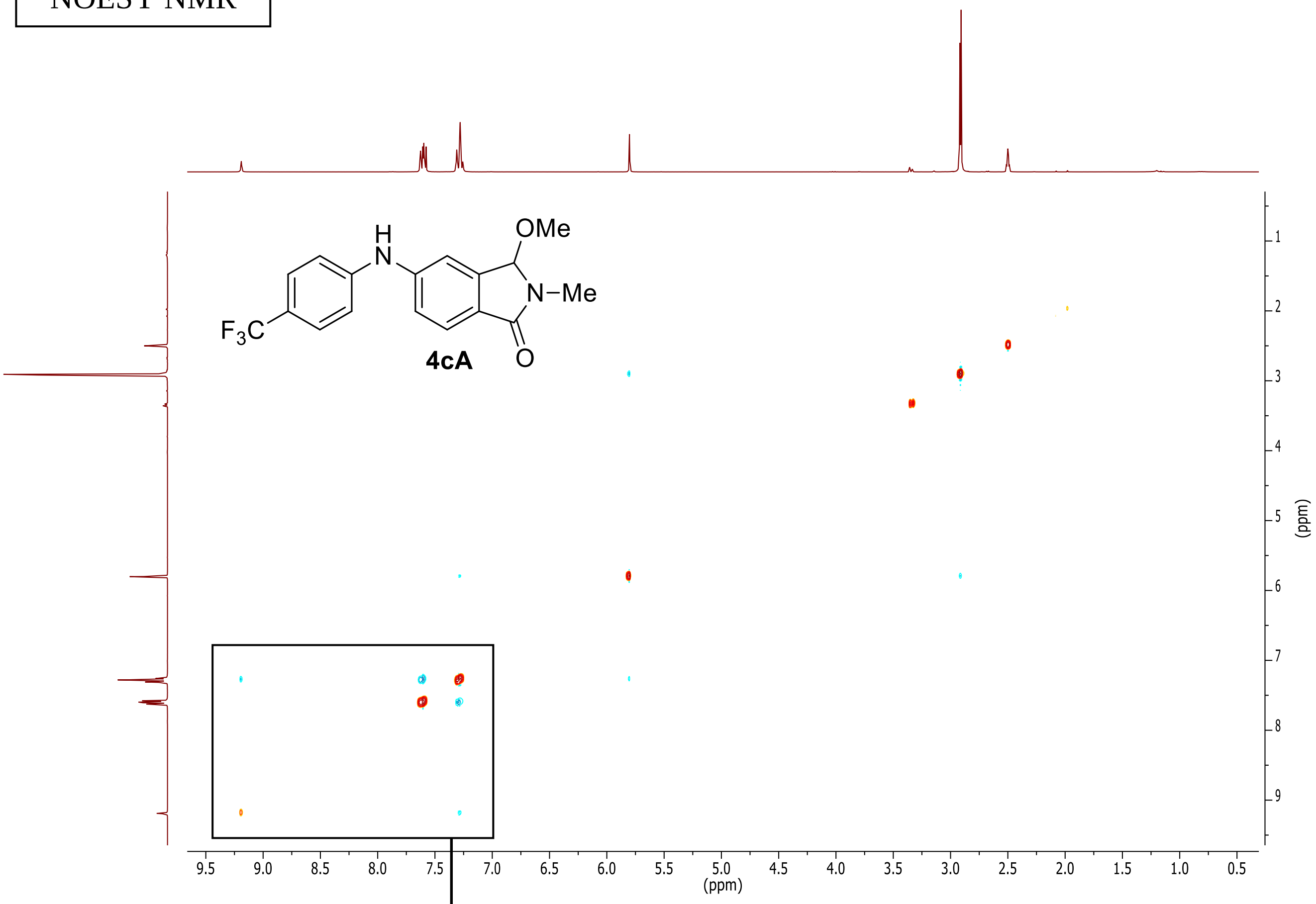

OMe
H
N
N–Me
F$_3$C
4cA
O
(ppm)
(ppm)

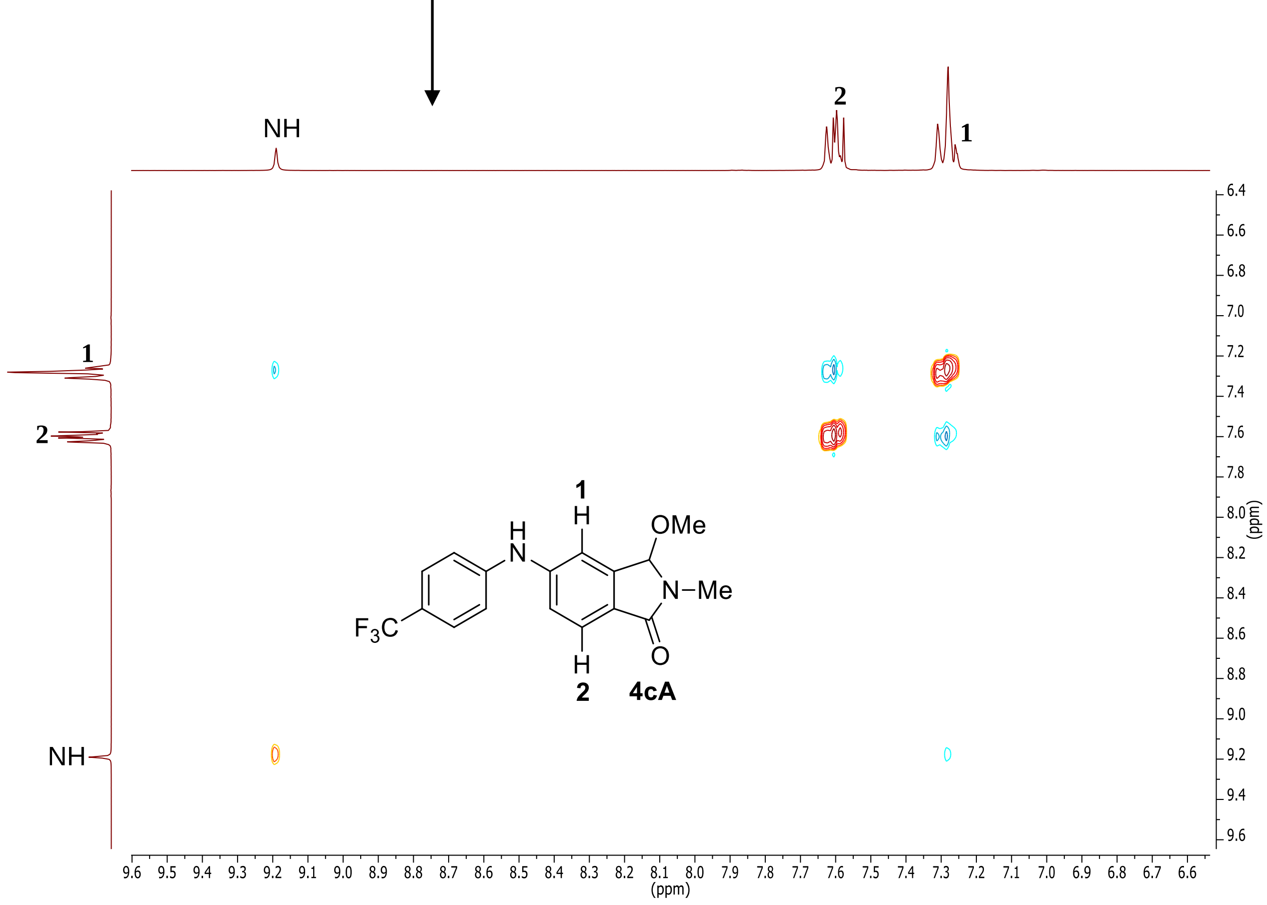

NH
2
1
1
2
NH
1
H
OMe
H
N
N–Me
F$_3$C
O
H
2
4cA
(ppm)
(ppm)

^{1}H NMR

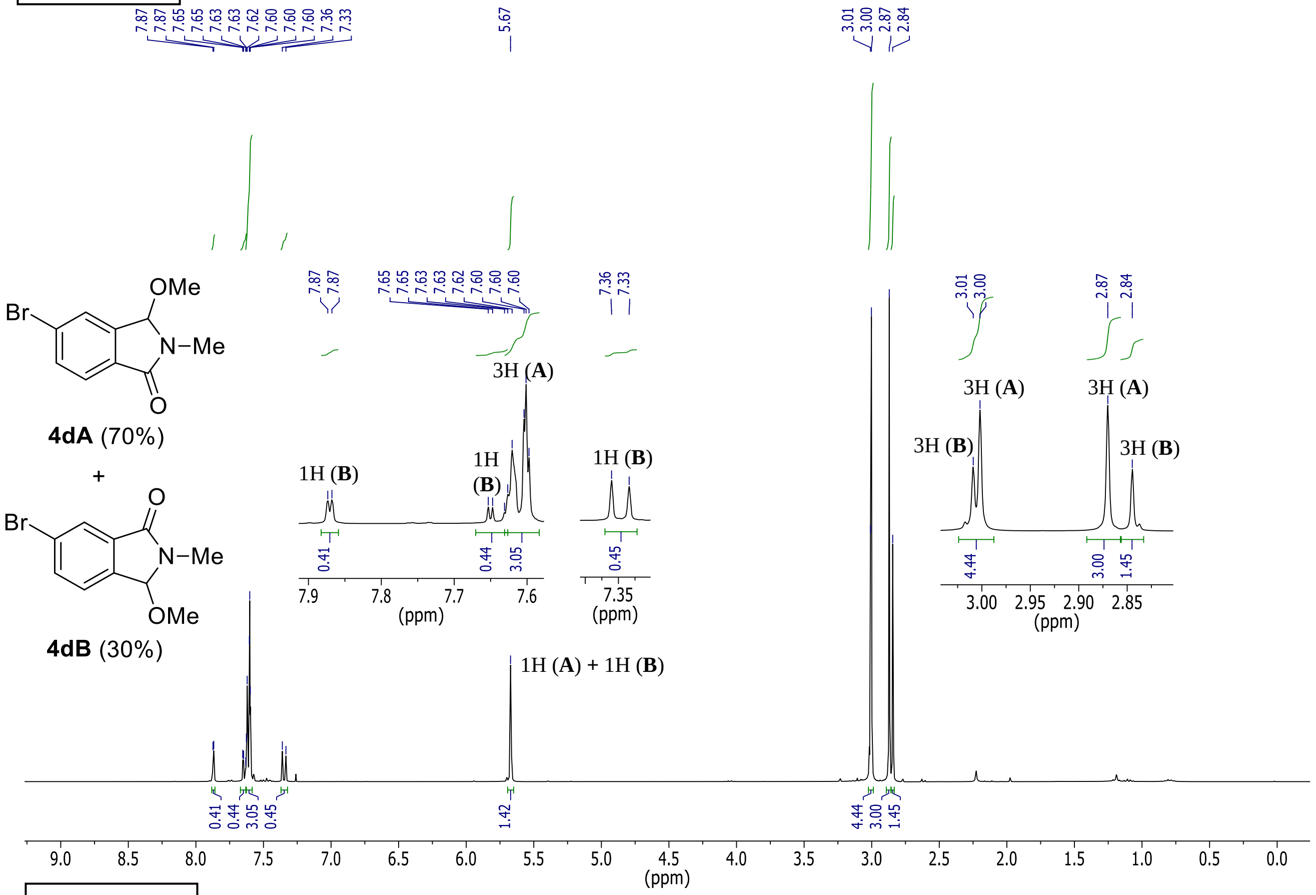

^{13}C NMR

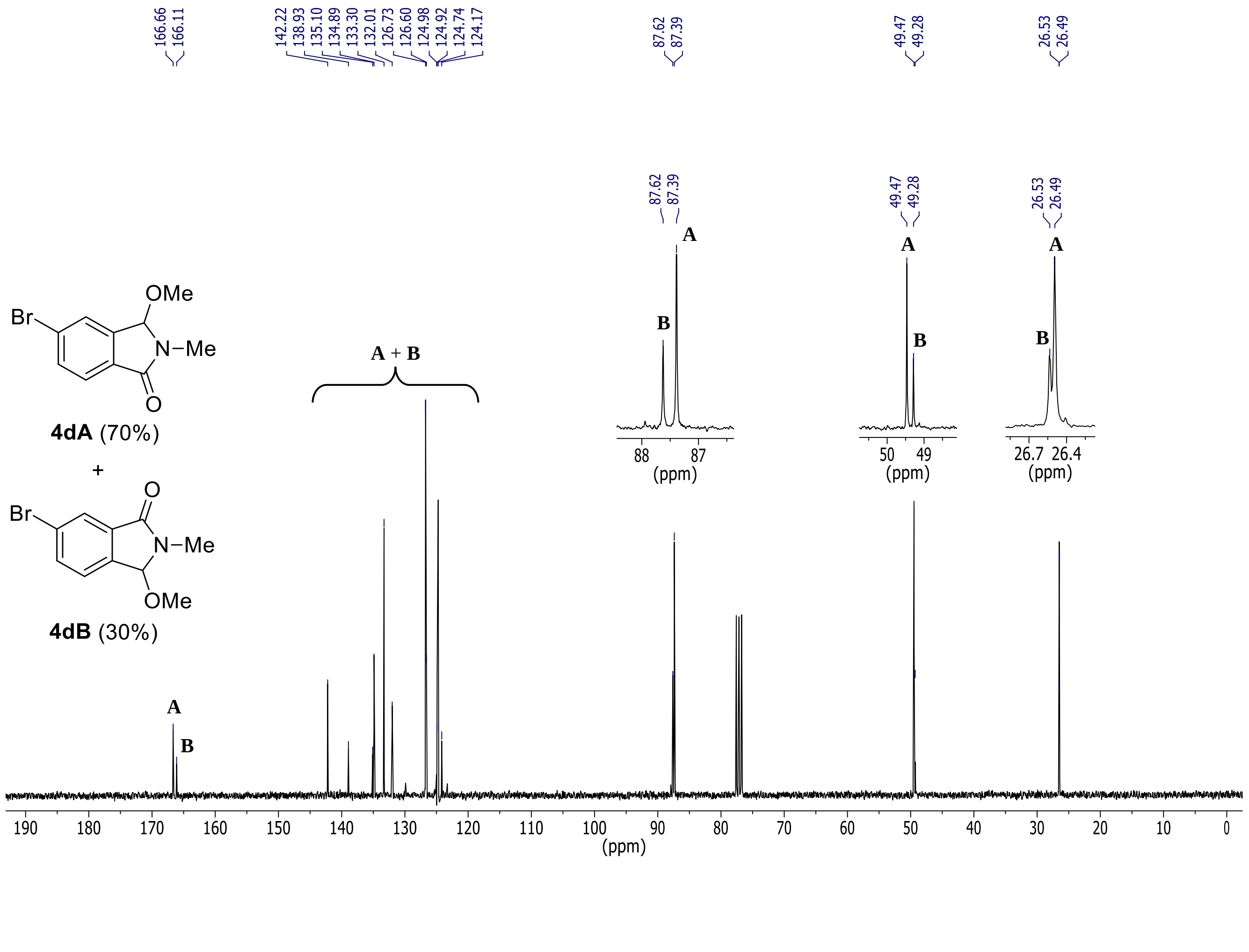

^{1}H NMR

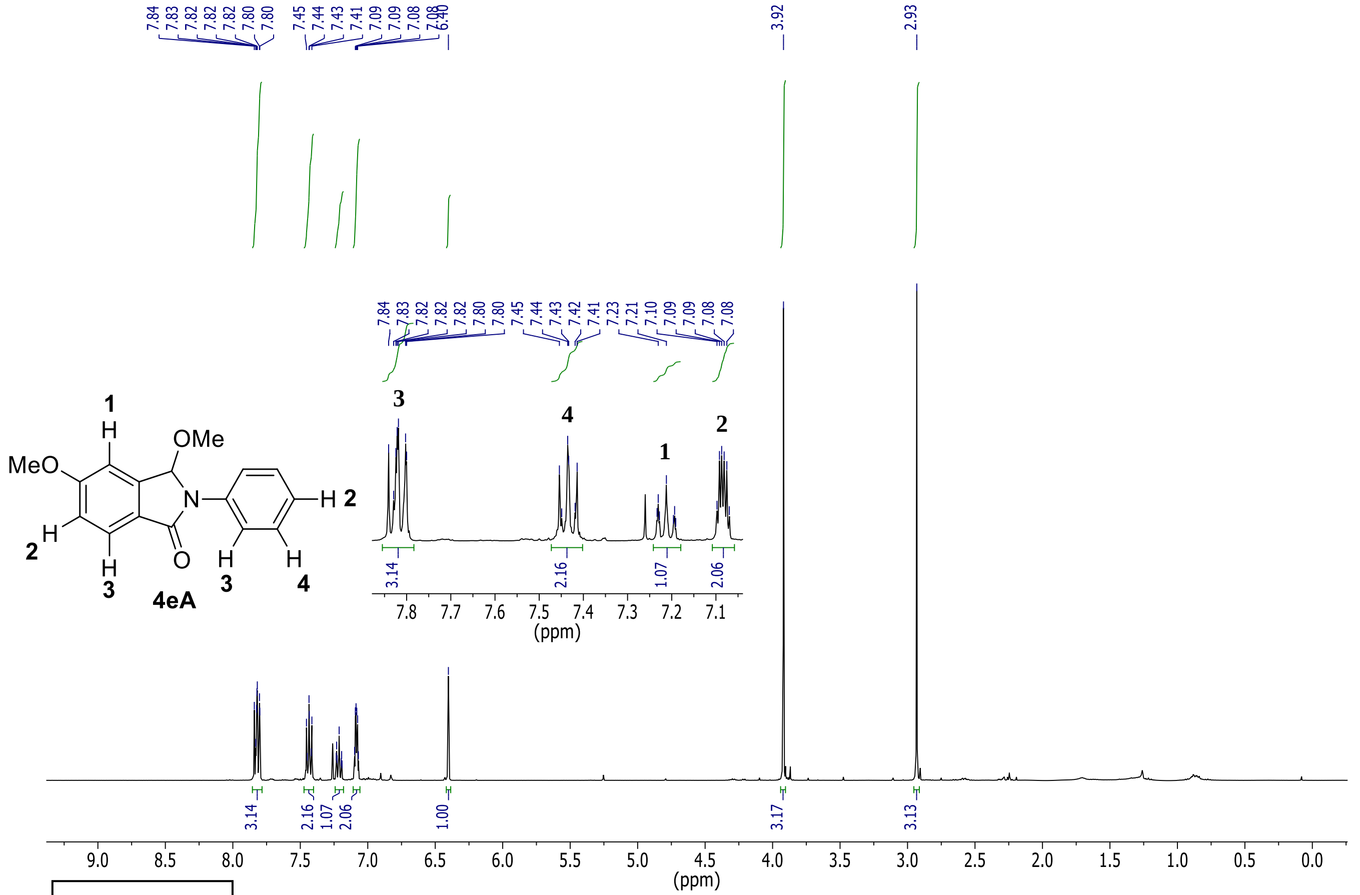

^{13}C NMR

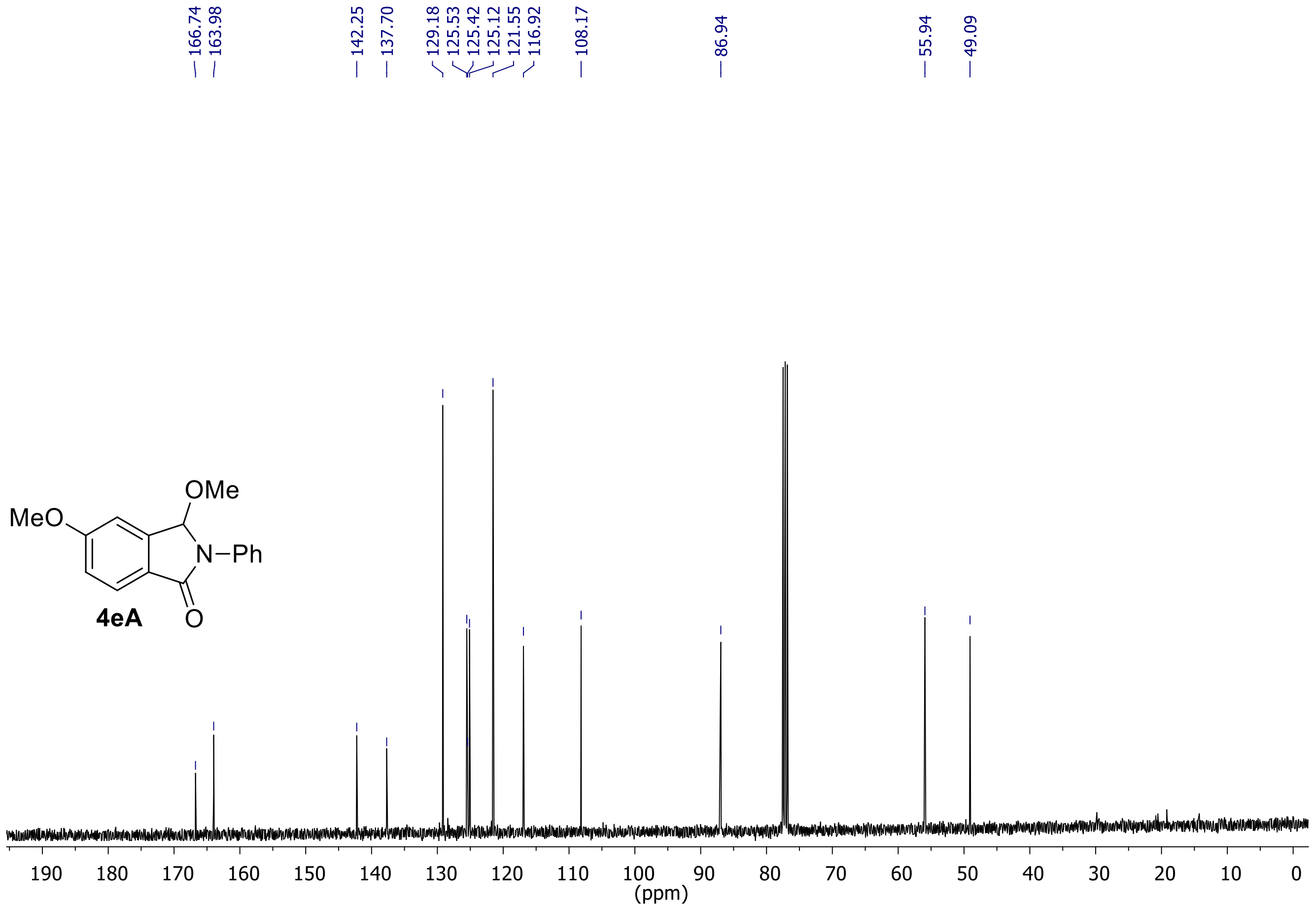

^{1}H NMR

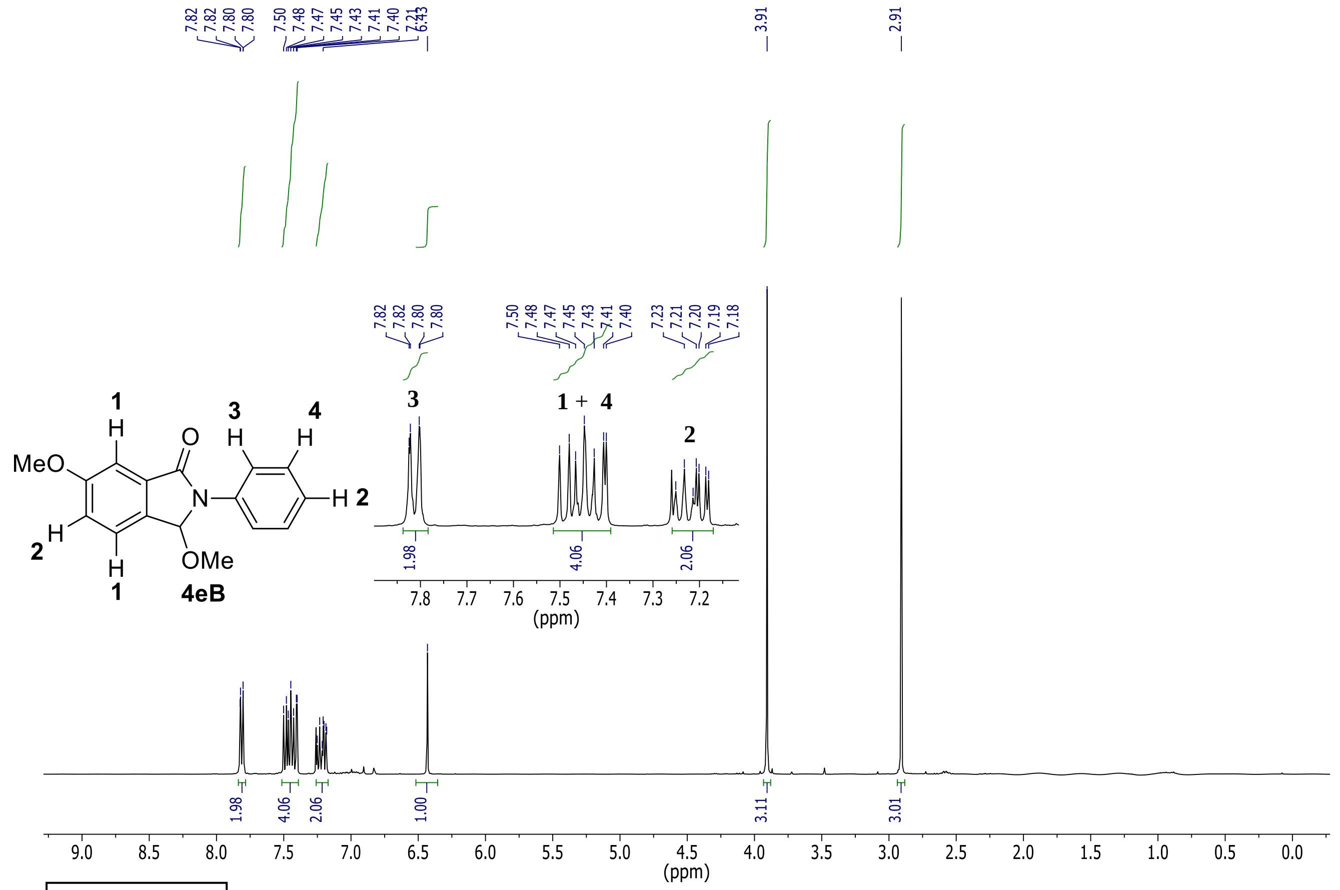

^{13}C NMR

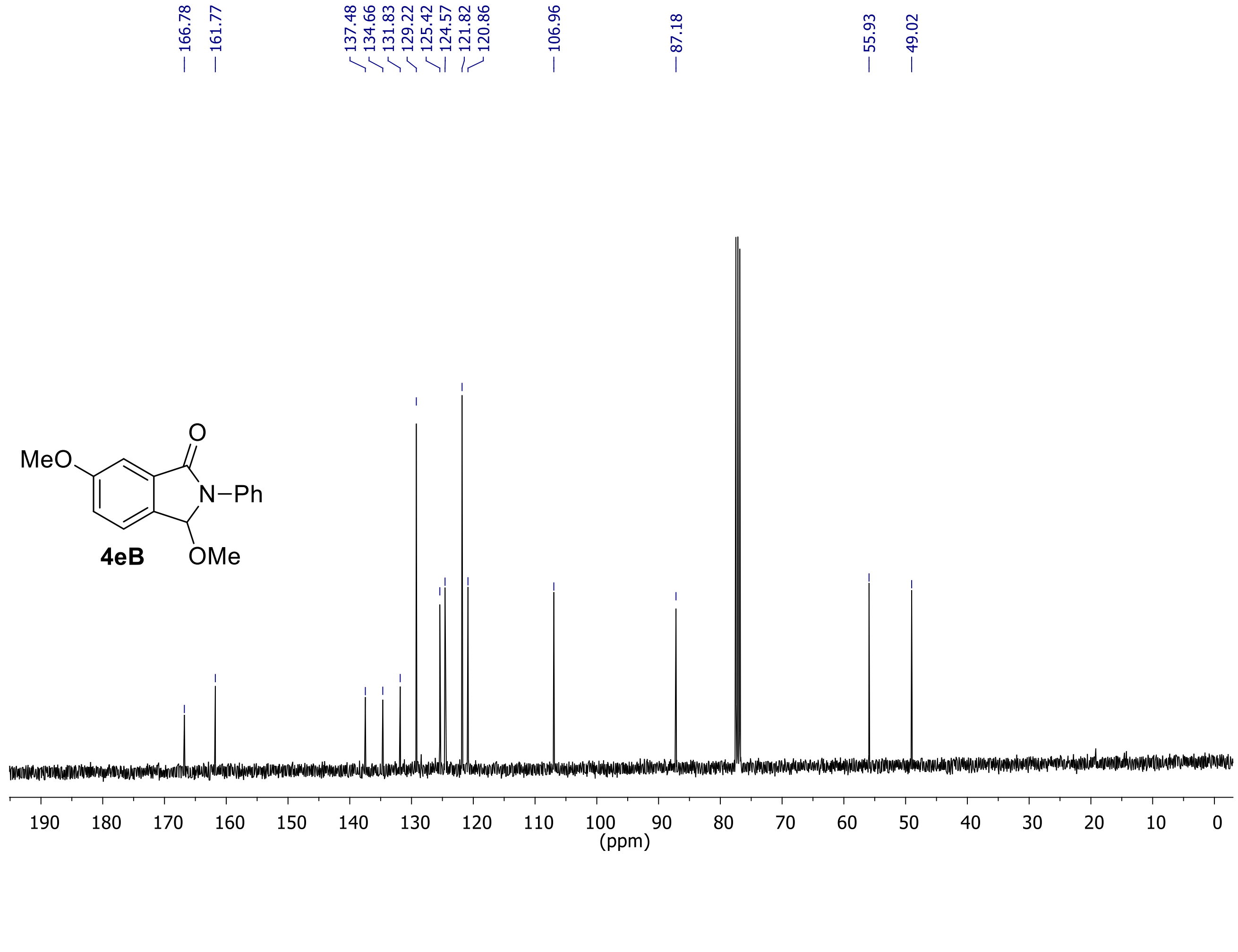

^{1}H NMR

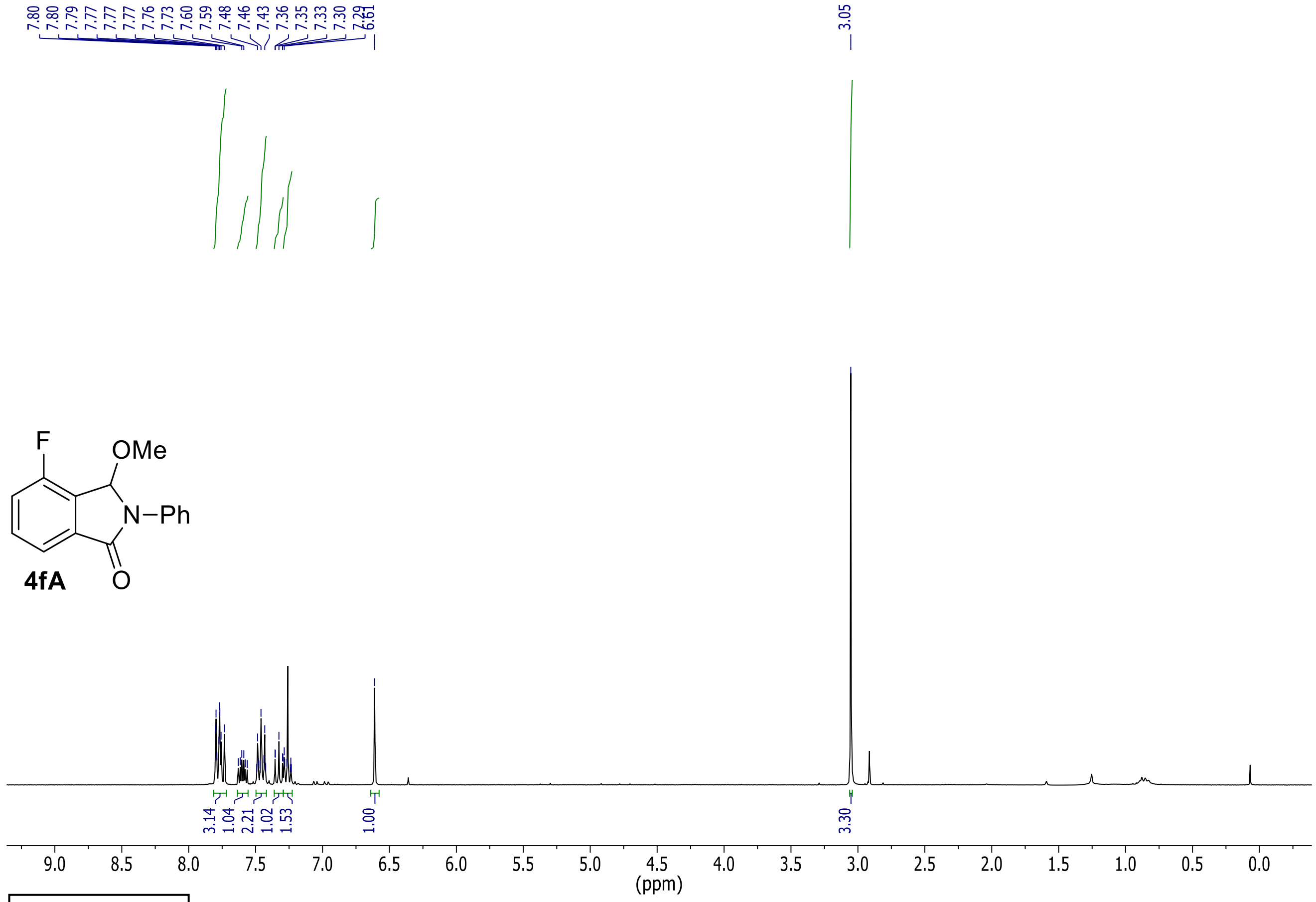

^{13}C NMR

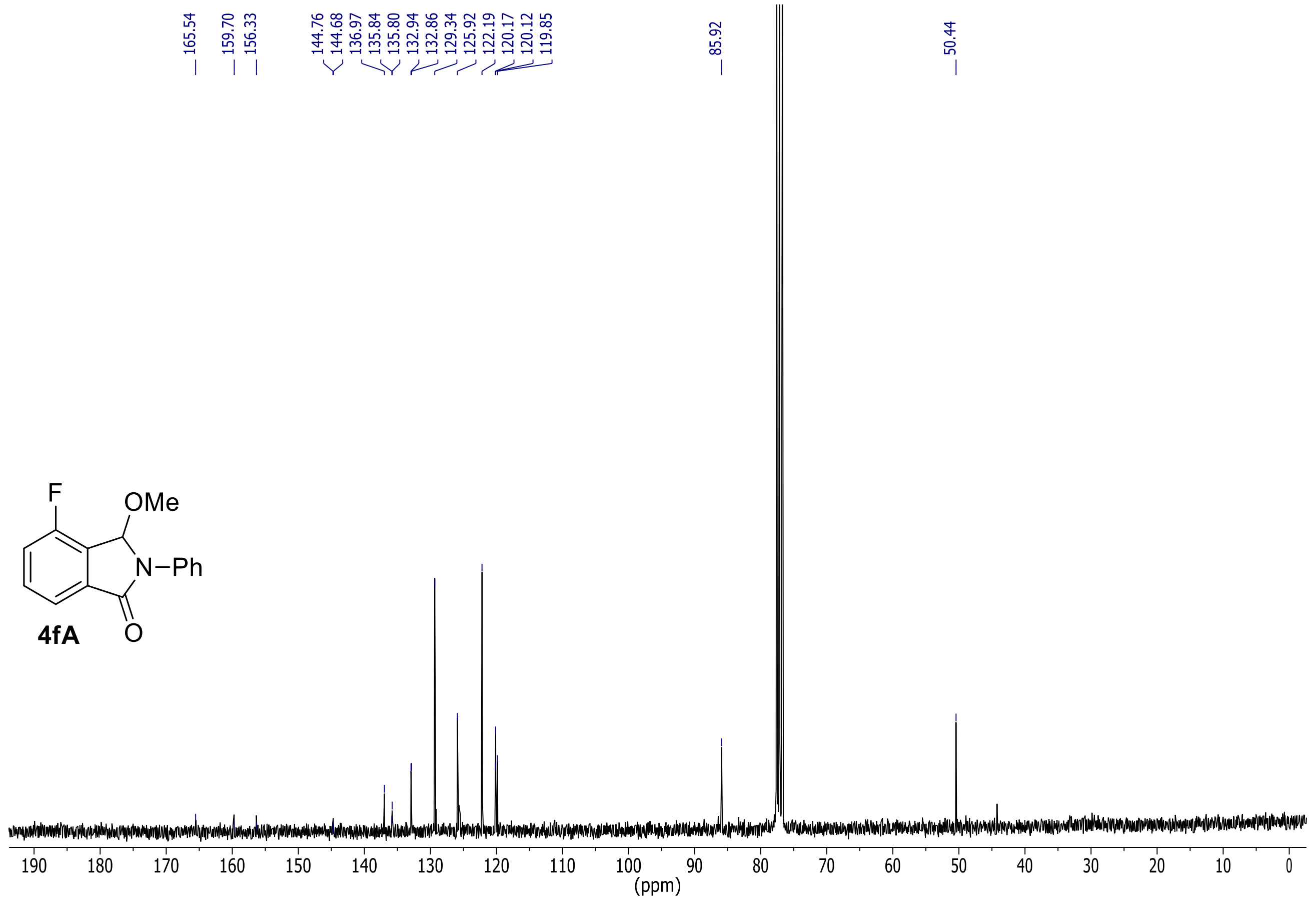

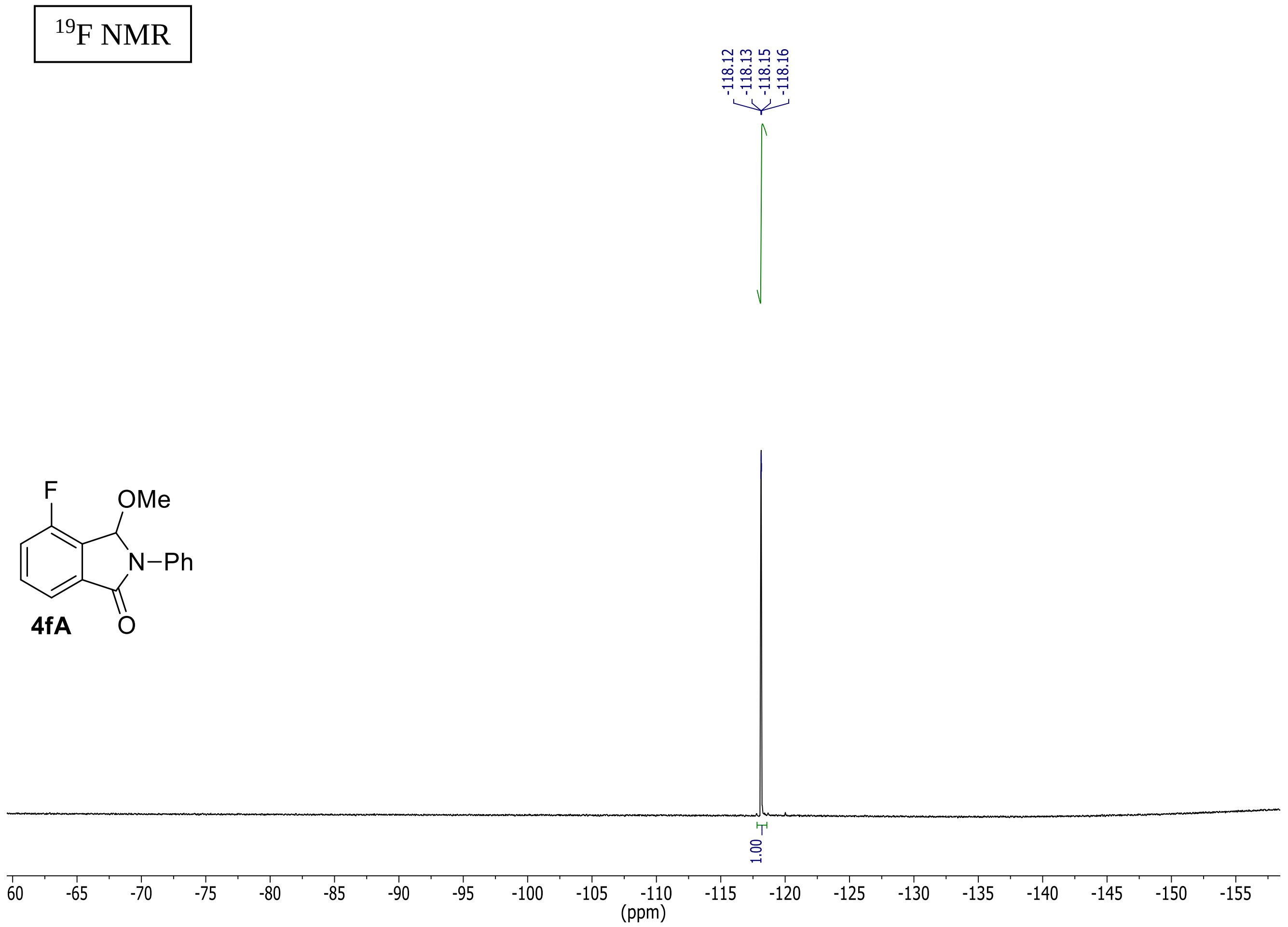

19F NMR
-118.12
-118.13
-118.15
-118.16
F
OMe
N−Ph
4fA
O
1.00
60 -65 -70 -75 -80 -85 -90 -95 -100 -105 -110 -115 -120 -125 -130 -135 -140 -145 -150 -155
(ppm)

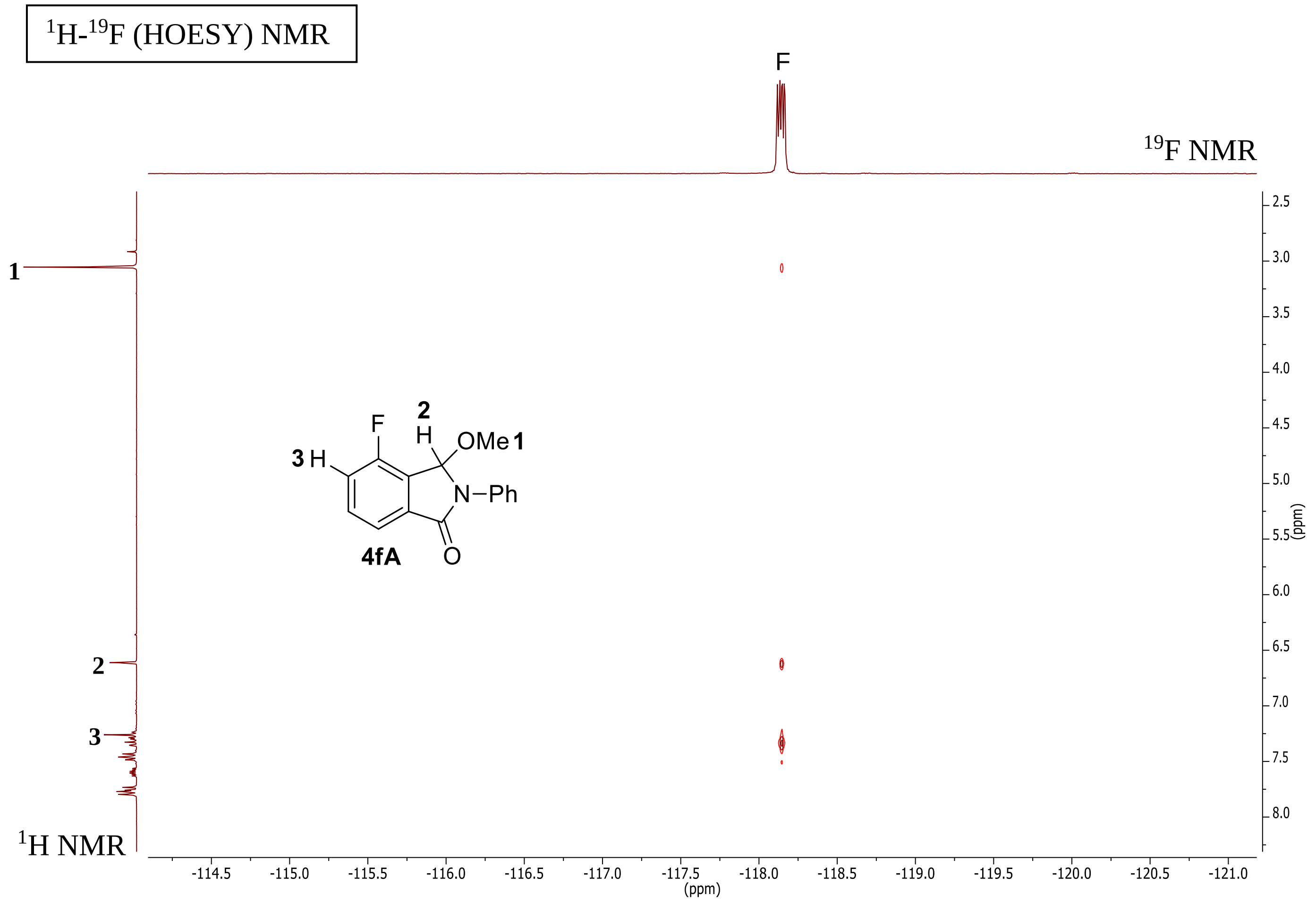

1H-19F (HOESY) NMR
F
19F NMR
1
2
3
1H NMR
3 H
F
2
H
OMe 1
N−Ph
4fA
O
2.5 3.0 3.5 4.0 4.5 5.0 5.5 6.0 6.5 7.0 7.5 8.0
(ppm)
-114.5 -115.0 -115.5 -116.0 -116.5 -117.0 -117.5 -118.0 -118.5 -119.0 -119.5 -120.0 -120.5 -121.0
(ppm)

^{1}H NMR

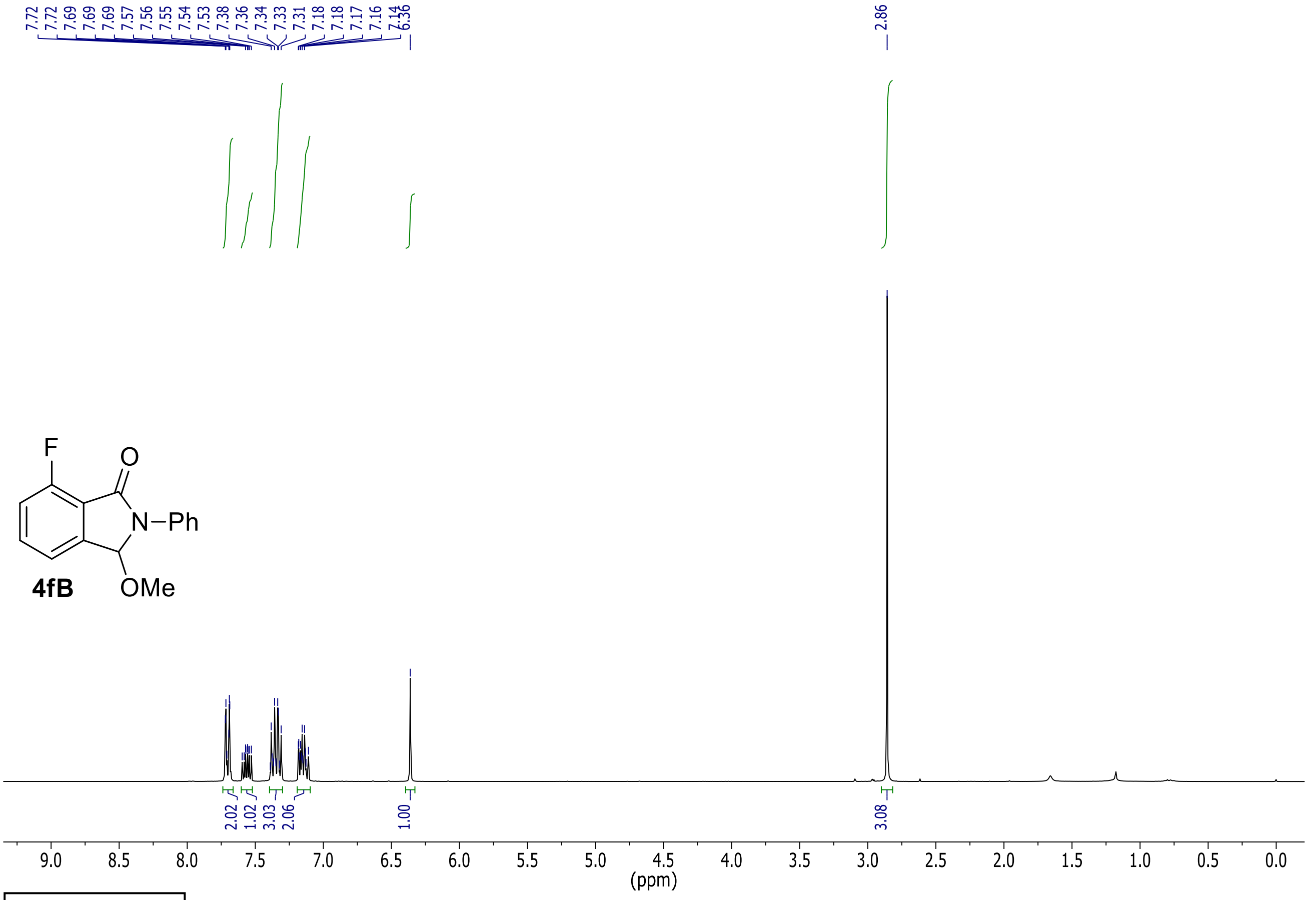

^{13}C NMR

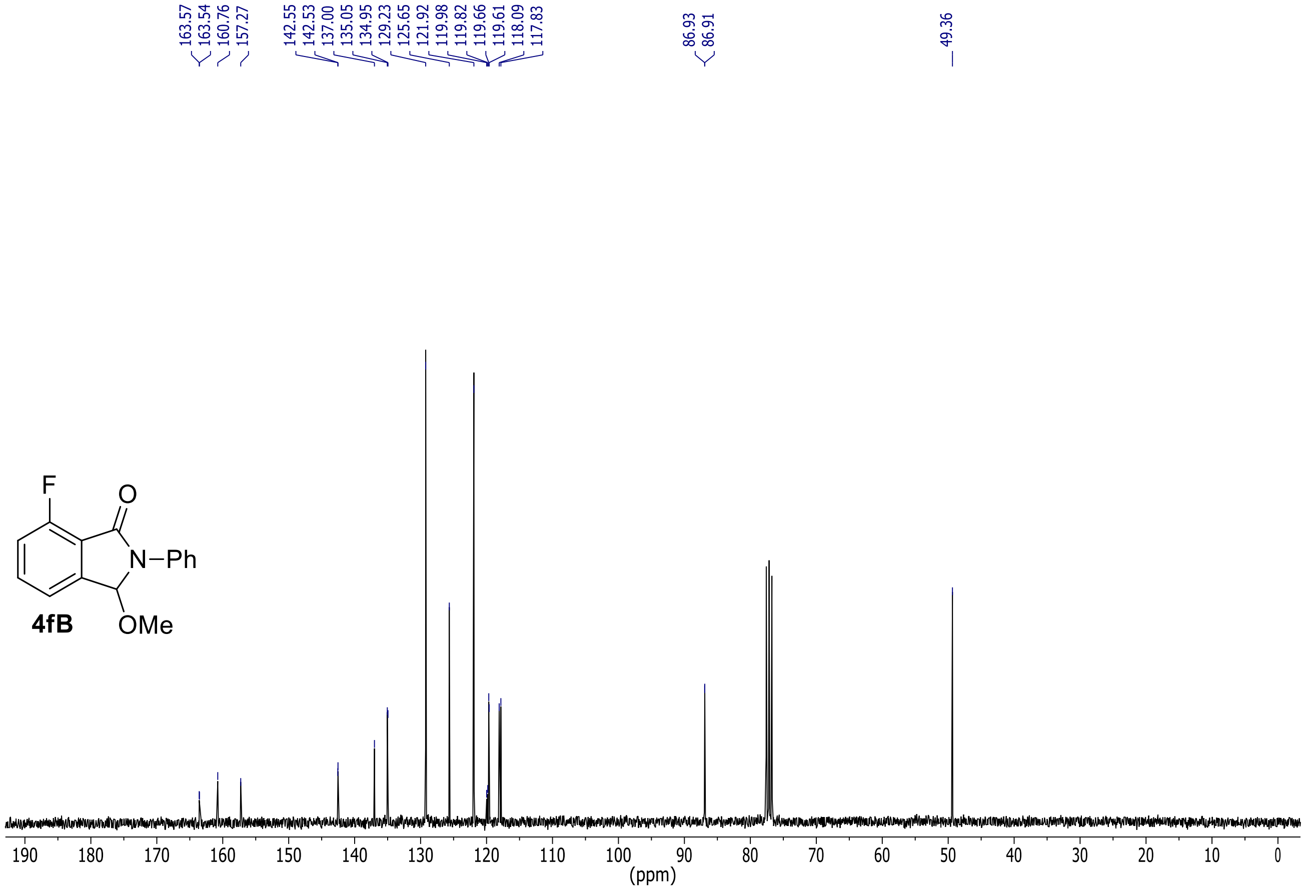

^{19}F NMR

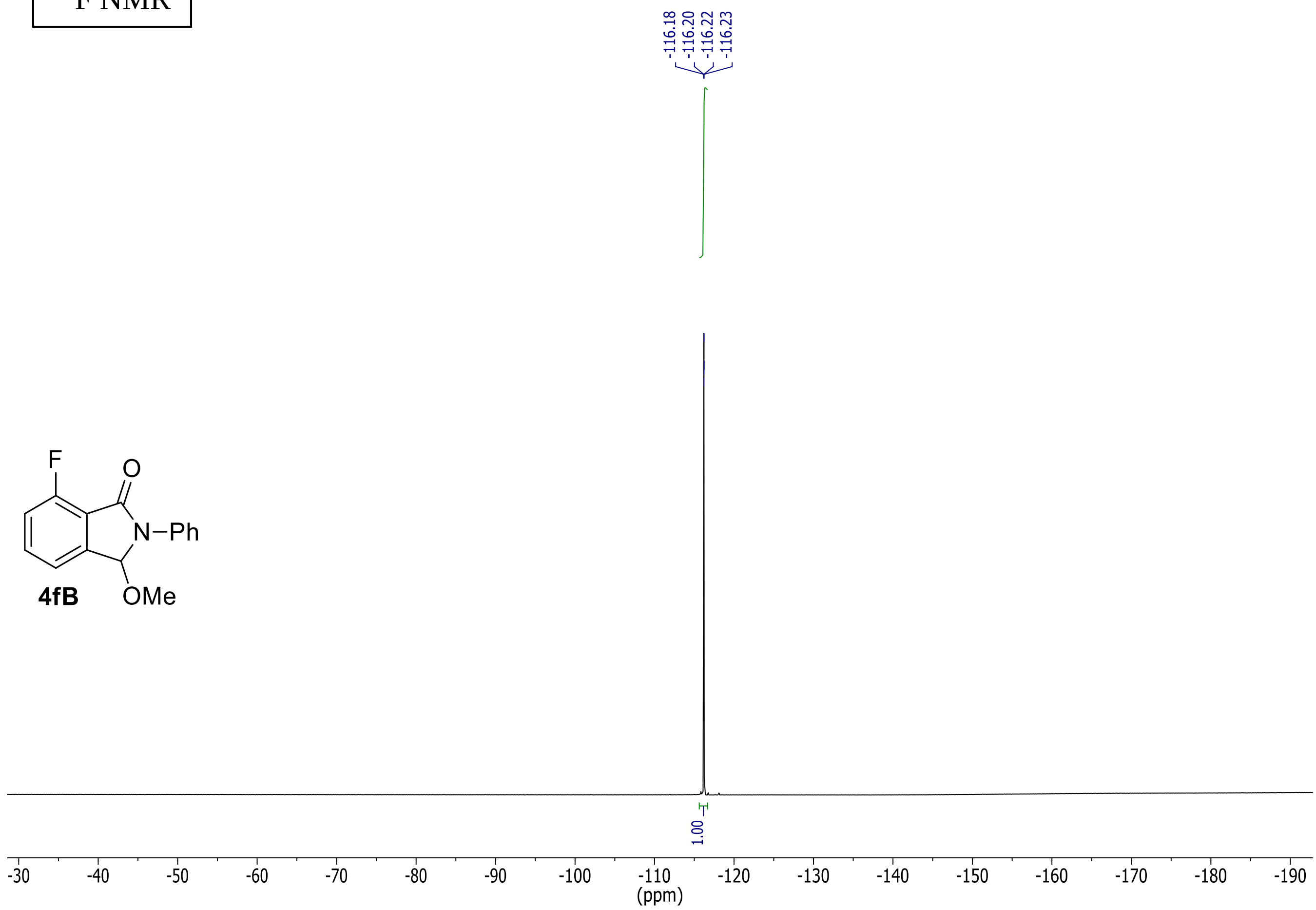

^{1}H-^{19}F (HOESY) NMR

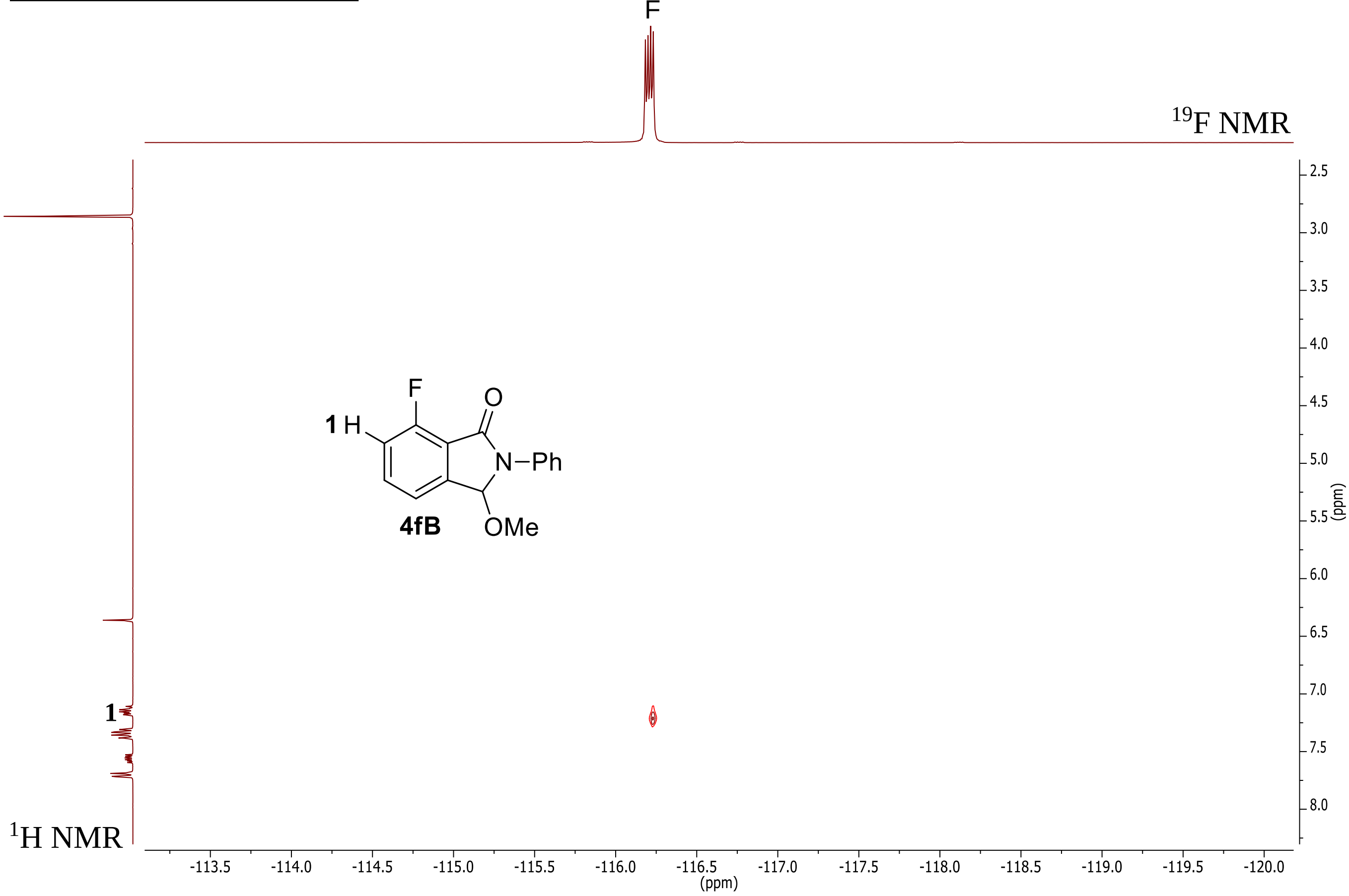

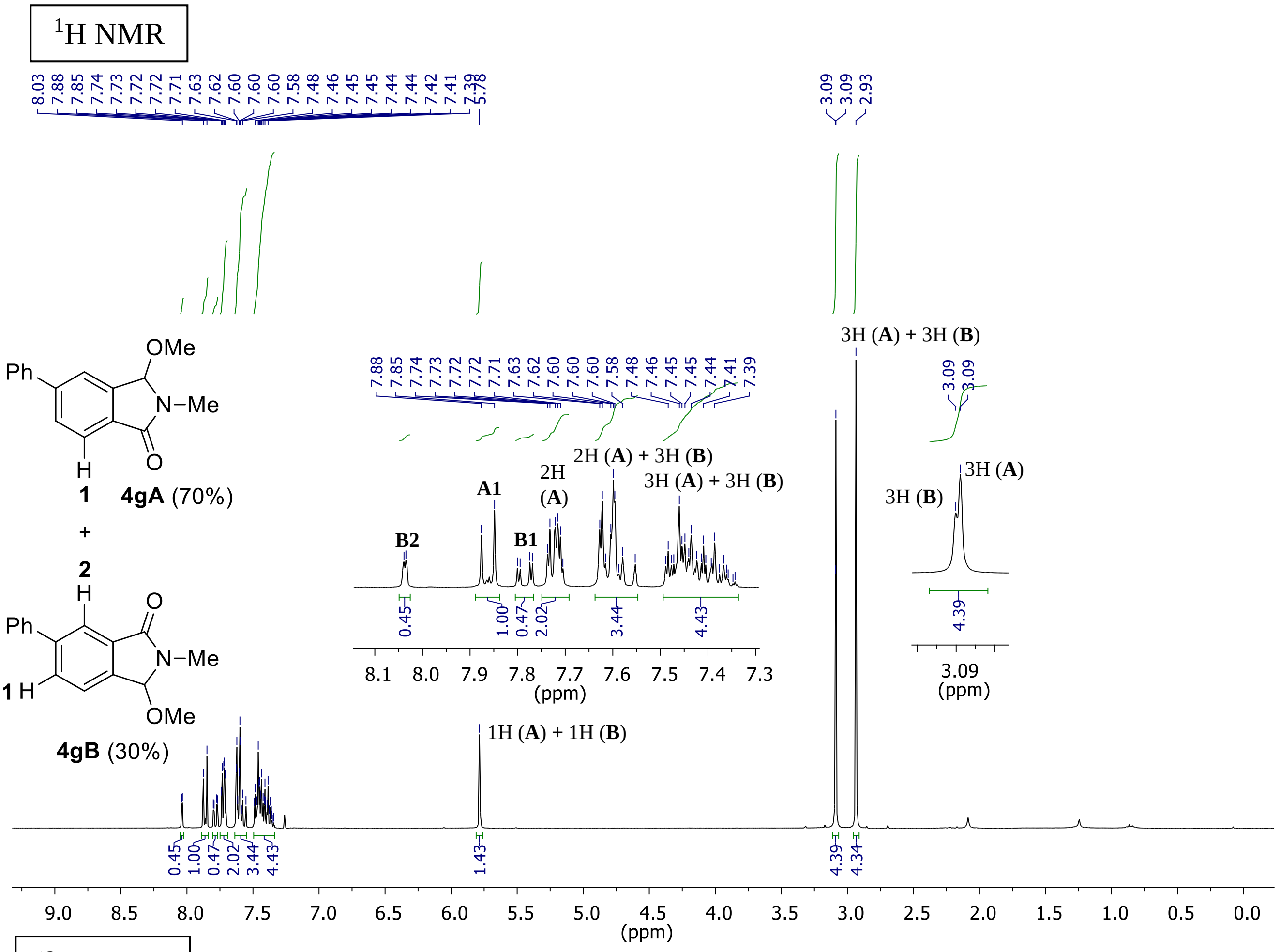

^{1}H NMR
8.03 7.88 7.85 7.74 7.73 7.72 7.72 7.71 7.63 7.62 7.60 7.60 7.60 7.58 7.48 7.46 7.45 7.45 7.44 7.44 7.42 7.41 7.39 5.78
3.09 3.09 2.93
Ph
OMe
N−Me
O
H
1
4gA (70%)
+
2
H
O
N−Me
1 H
OMe
4gB (30%)
7.88 7.85 7.74 7.73 7.72 7.72 7.71 7.63 7.62 7.60 7.60 7.58 7.48 7.46 7.45 7.45 7.44 7.41 7.39
B2
A1
B1
2H (A)
2H (A) + 3H (B)
3H (A) + 3H (B)
0.45 1.00 0.47 2.02 3.44 4.43
8.1 8.0 7.9 7.8 7.7 7.6 7.5 7.4 7.3
(ppm)
3H (A) + 3H (B)
3.09 3.09
3H (B)
3H (A)
4.39
3.09
(ppm)
1H (A) + 1H (B)
0.45 1.00 0.47 2.02 3.44 4.43
1.43
4.39 4.34
9.0 8.5 8.0 7.5 7.0 6.5 6.0 5.5 5.0 4.5 4.0 3.5 3.0 2.5 2.0 1.5 1.0 0.5 0.0
(ppm)

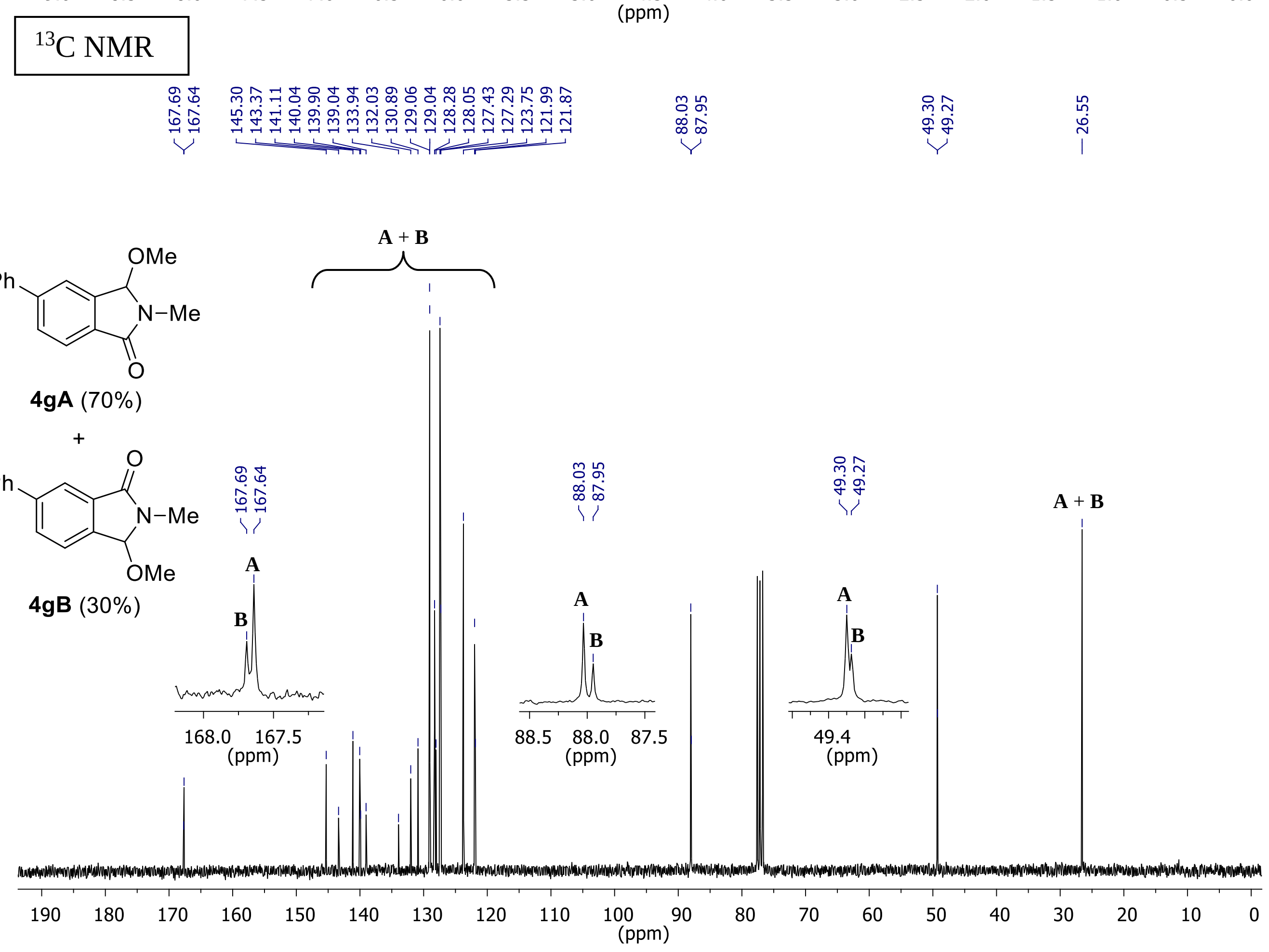

^{13}C NMR
167.69 167.64
145.30 143.37 141.11 140.04 139.90 139.04 133.94 132.03 130.89 129.06 129.04 128.28 128.05 127.43 127.29 123.75 121.99 121.87
88.03 87.95
49.30 49.27
26.55
A + B
Ph
OMe
N−Me
O
4gA (70%)
+
O
N−Me
OMe
4gB (30%)
167.69 167.64
A
B
168.0 167.5
(ppm)
88.03 87.95
A
B
88.5 88.0 87.5
(ppm)
49.30 49.27
A
B
49.4
(ppm)
A + B
190 180 170 160 150 140 130 120 110 100 90 80 70 60 50 40 30 20 10 0
(ppm)

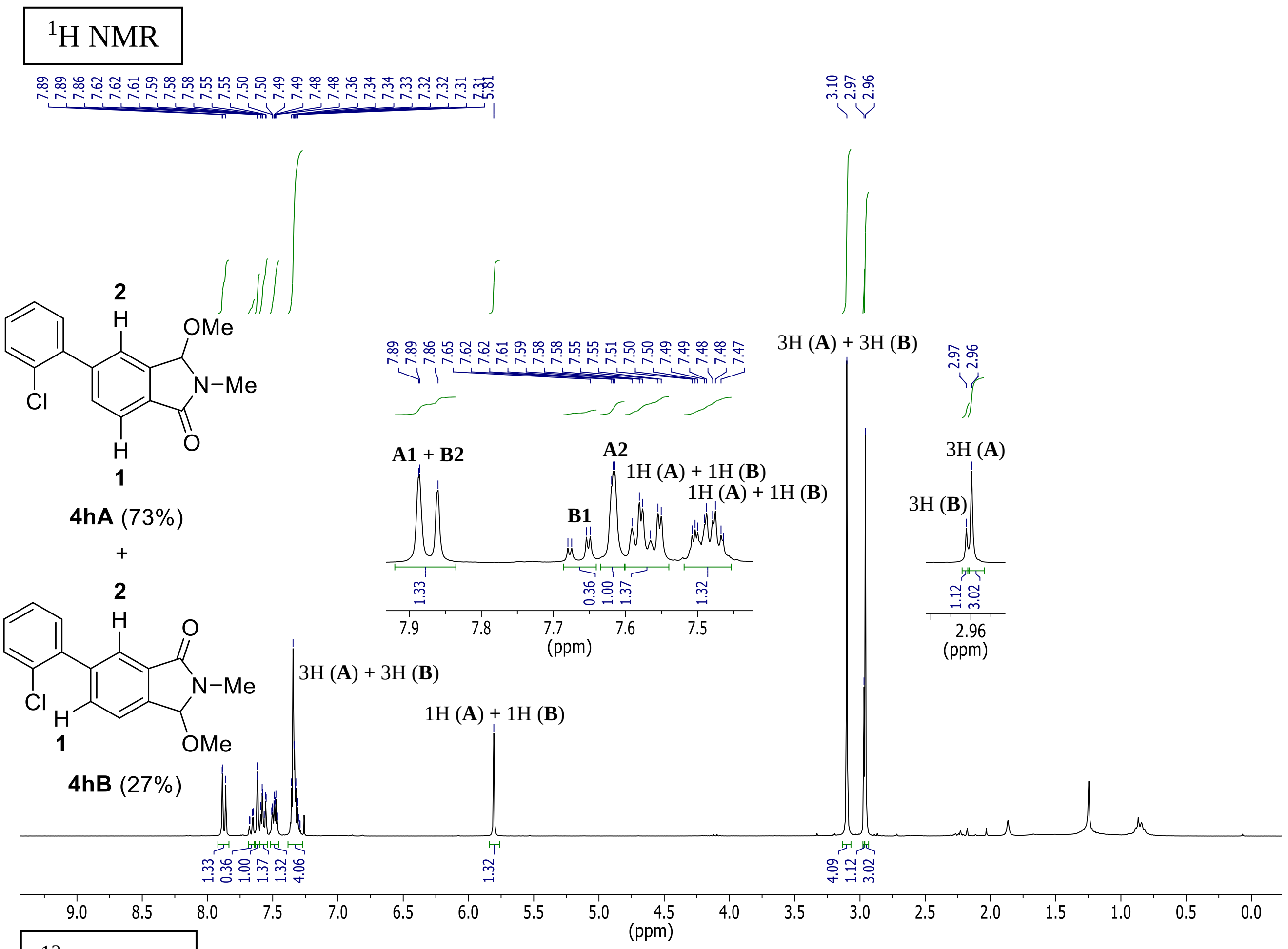

1H NMR
3H (A) + 3H (B)
1H (A) + 1H (B)
A1 + B2
B1
A2
3H (A)
3H (B)
4hA (73%)
+
4hB (27%)
(ppm)

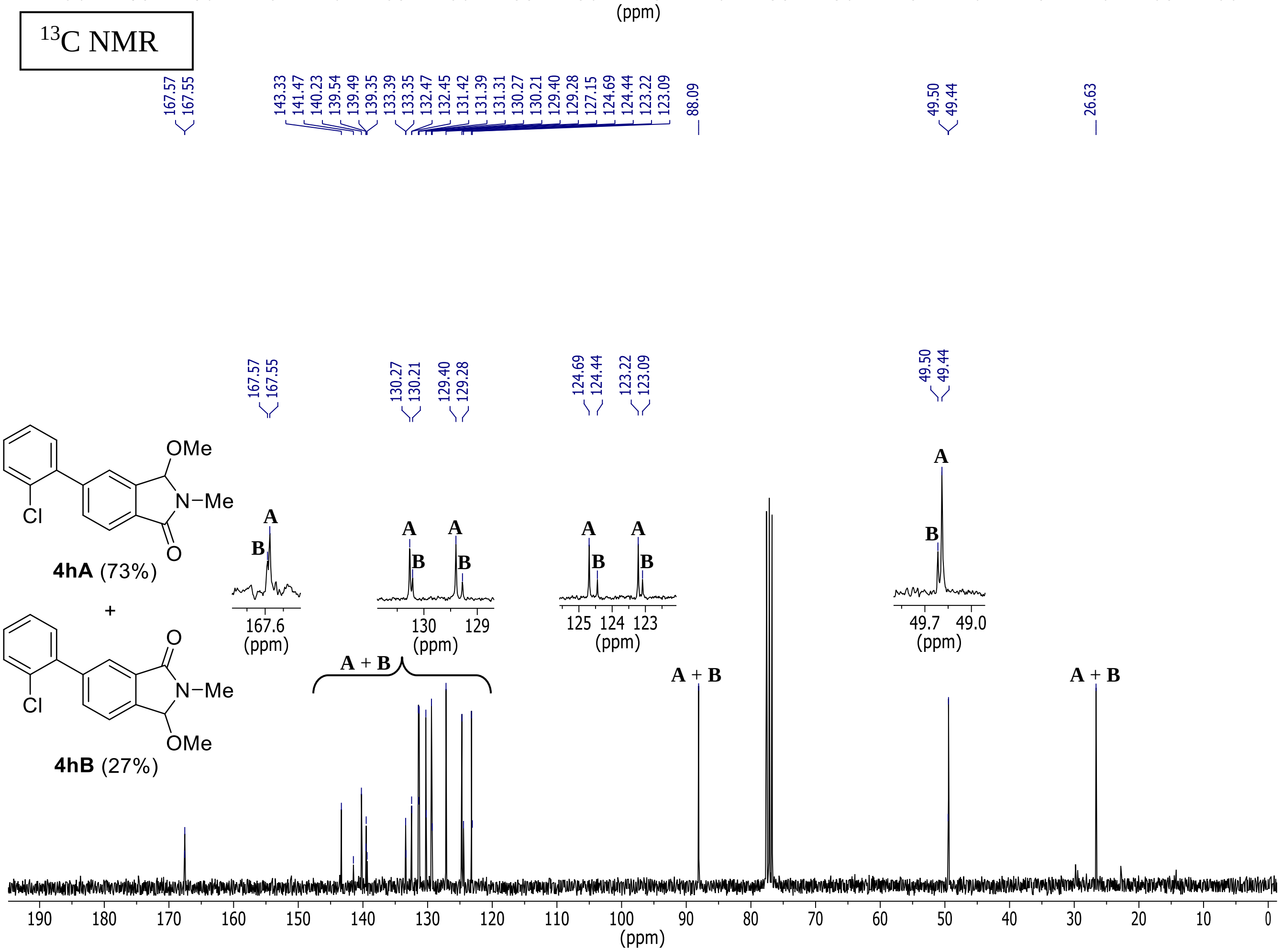

13C NMR
4hA (73%)
+
4hB (27%)
A + B
(ppm)

^{1}H NMR

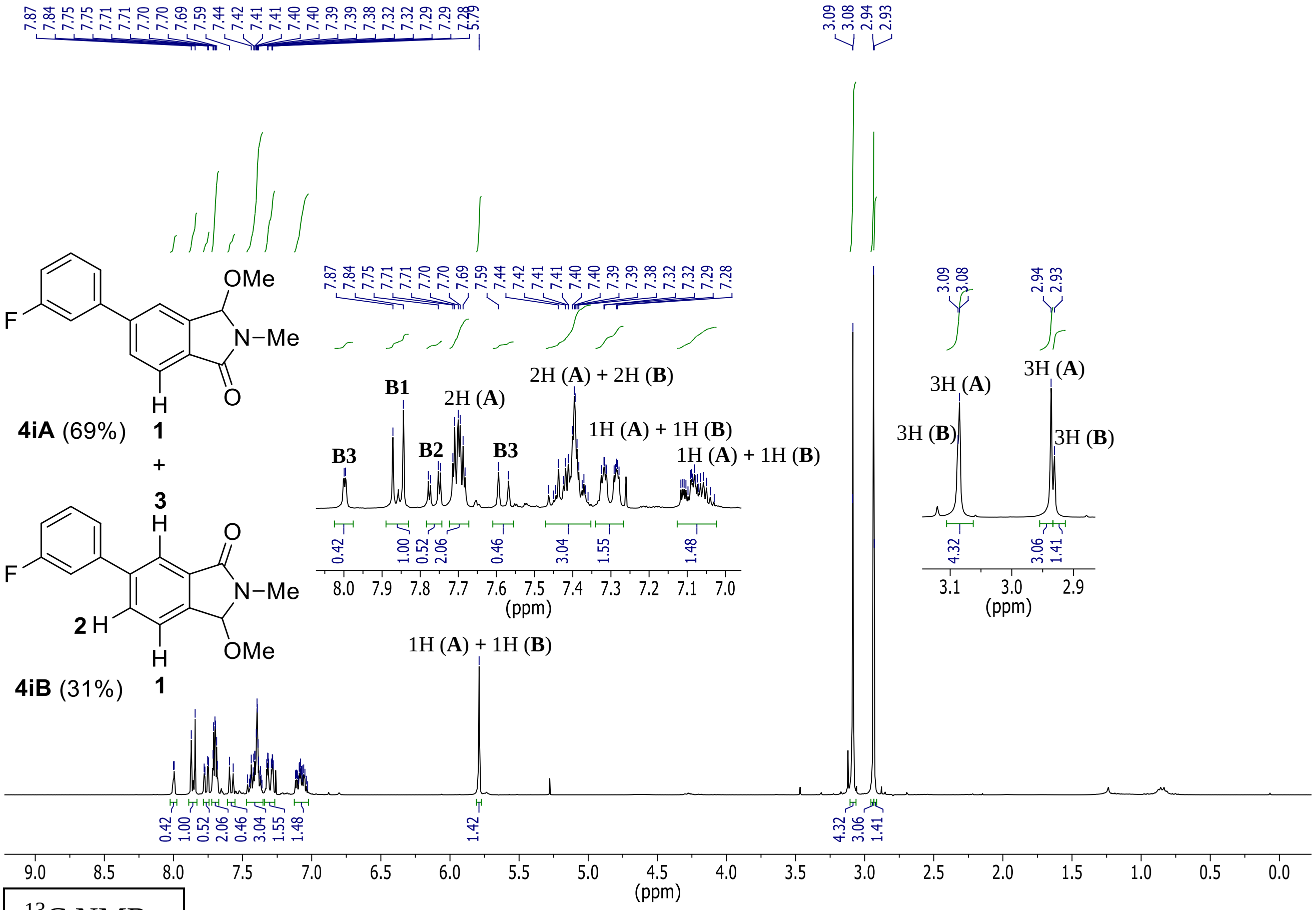

^{13}C NMR

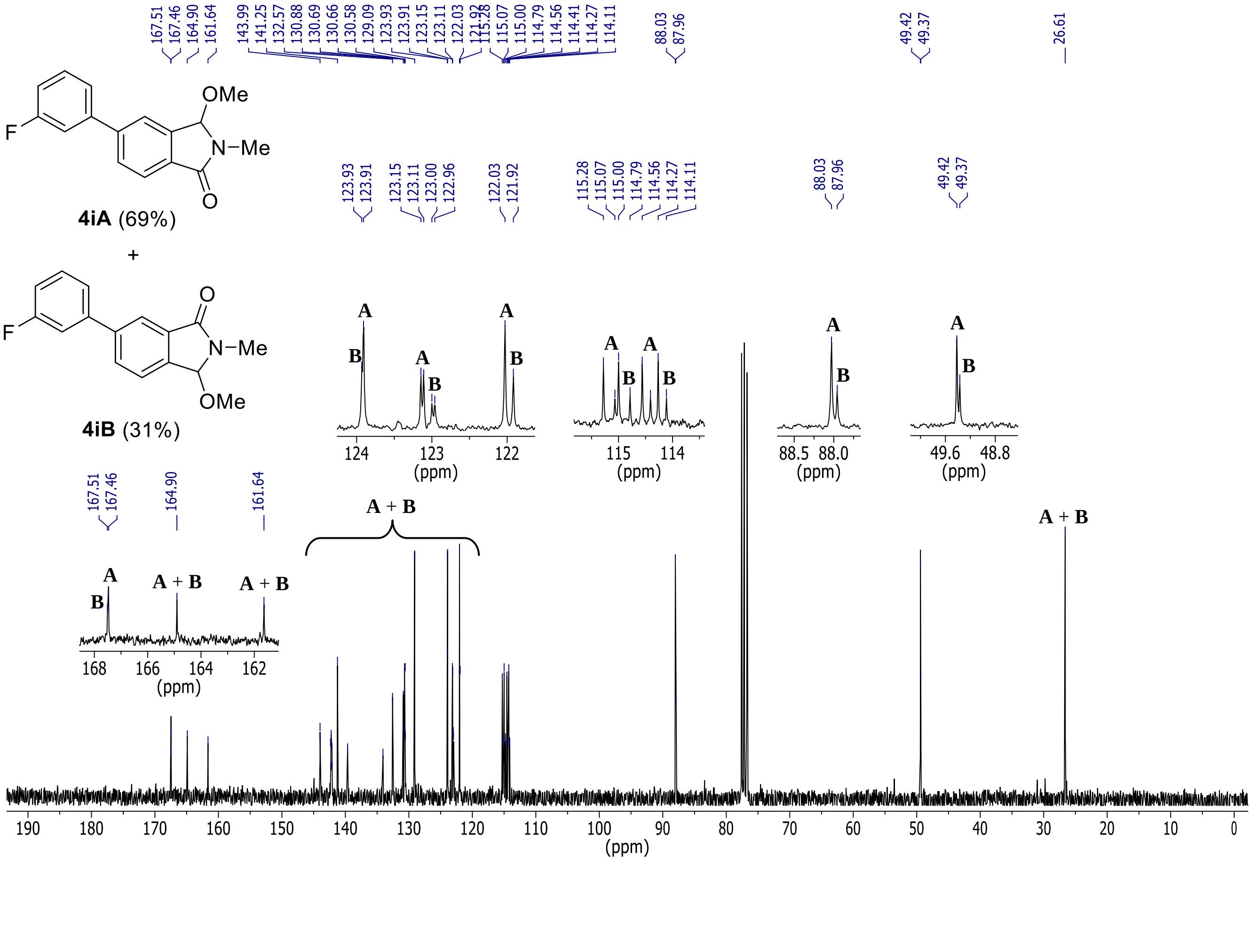

^{19}F NMR

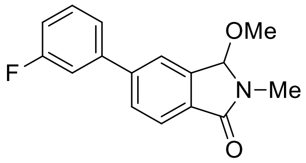

4iA (69%)

+

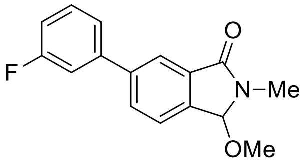

4iB (31%)

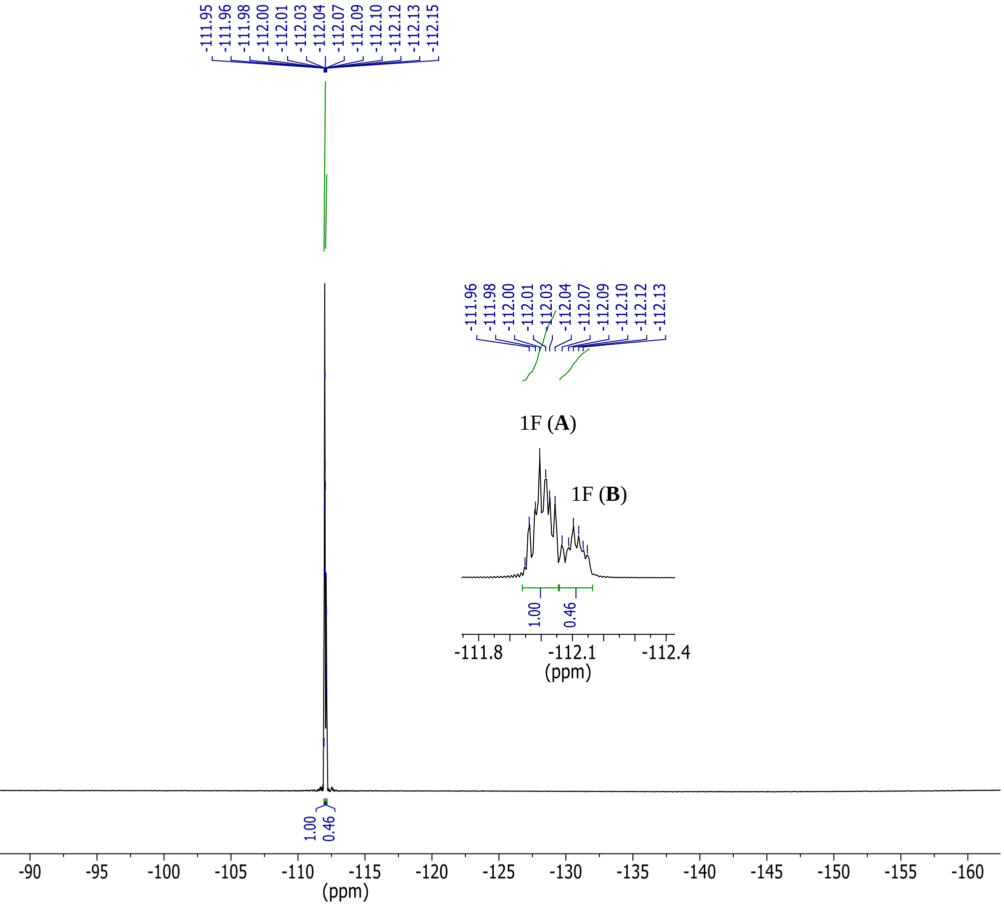

^{1}H NMR

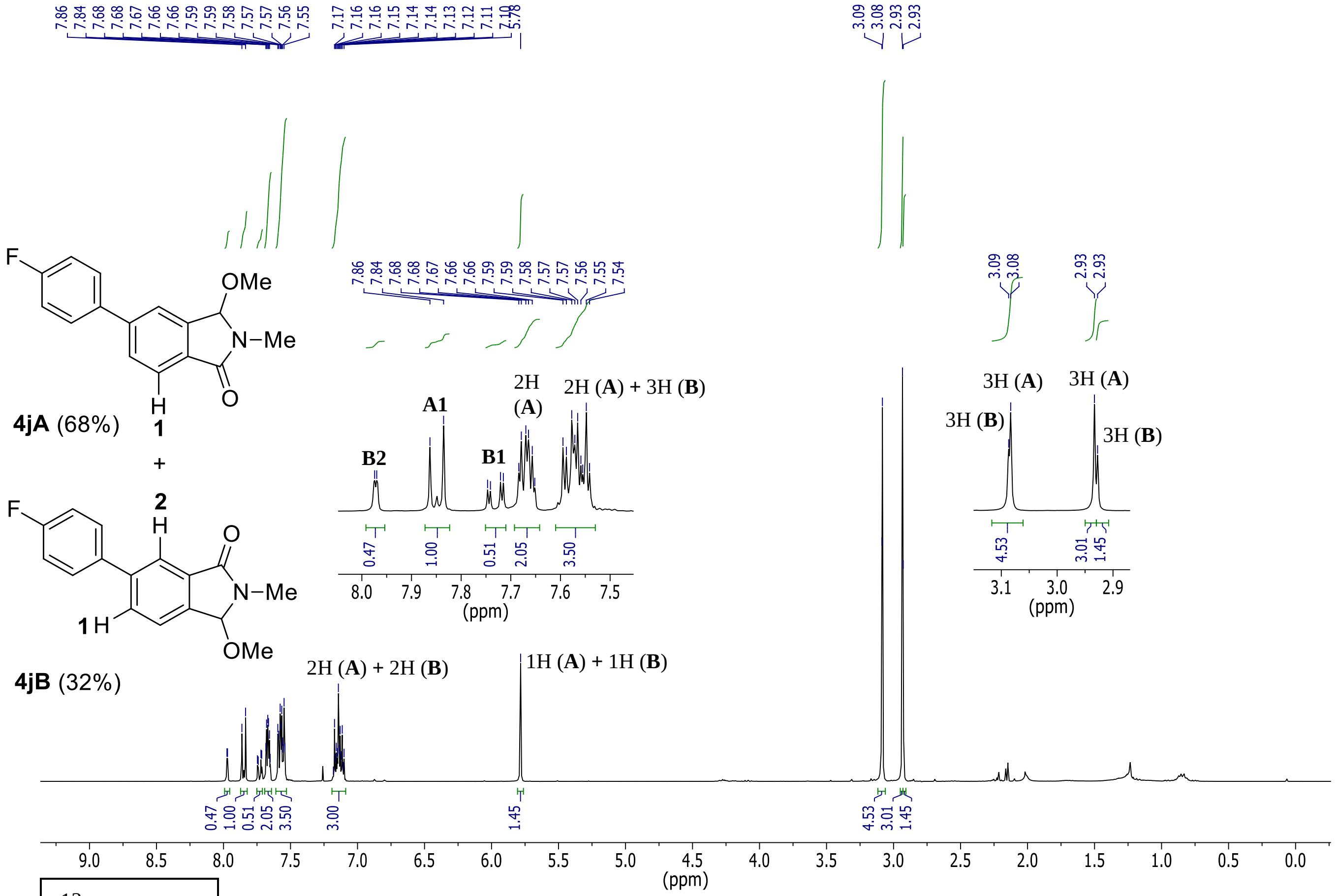

^{13}C NMR

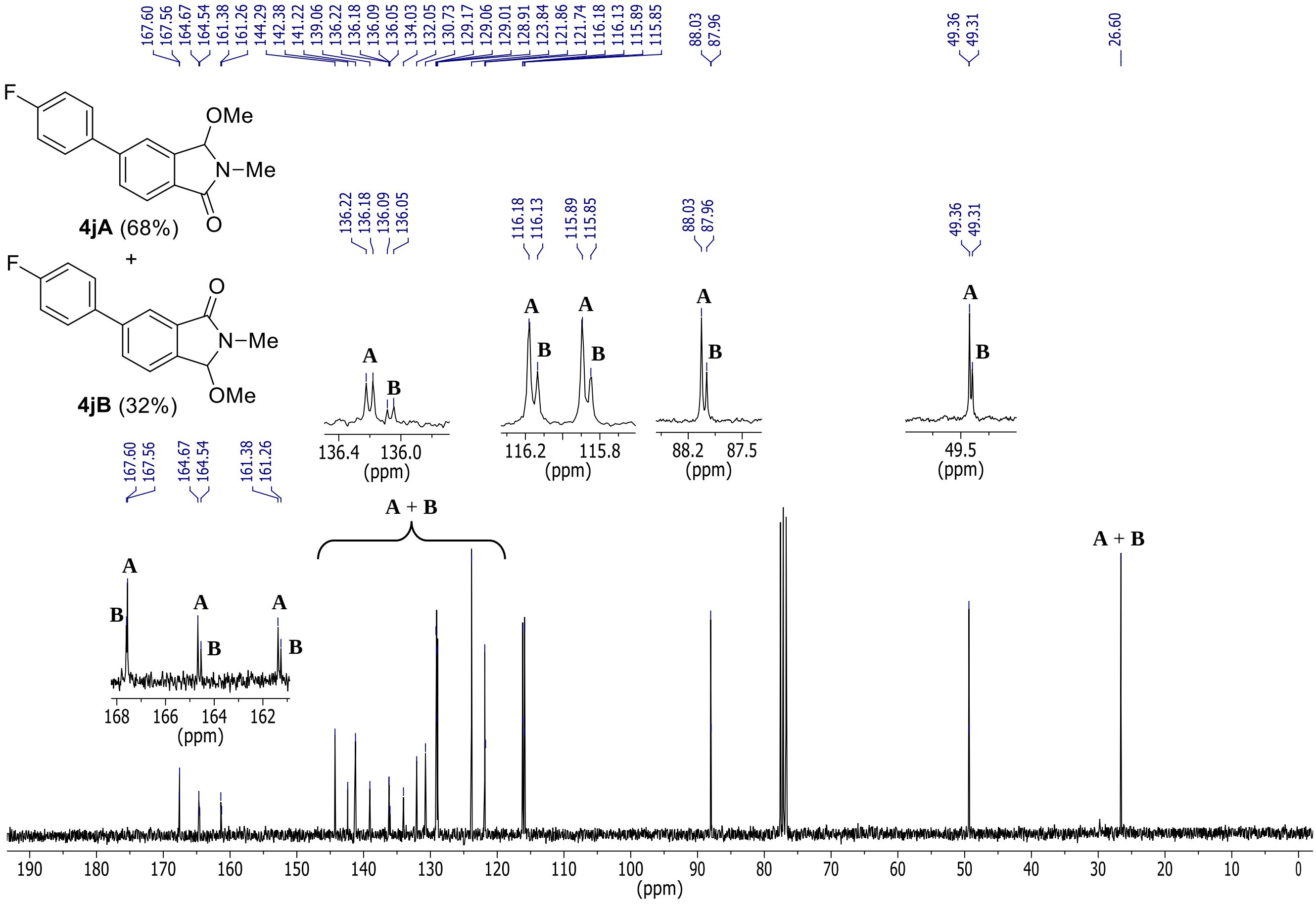

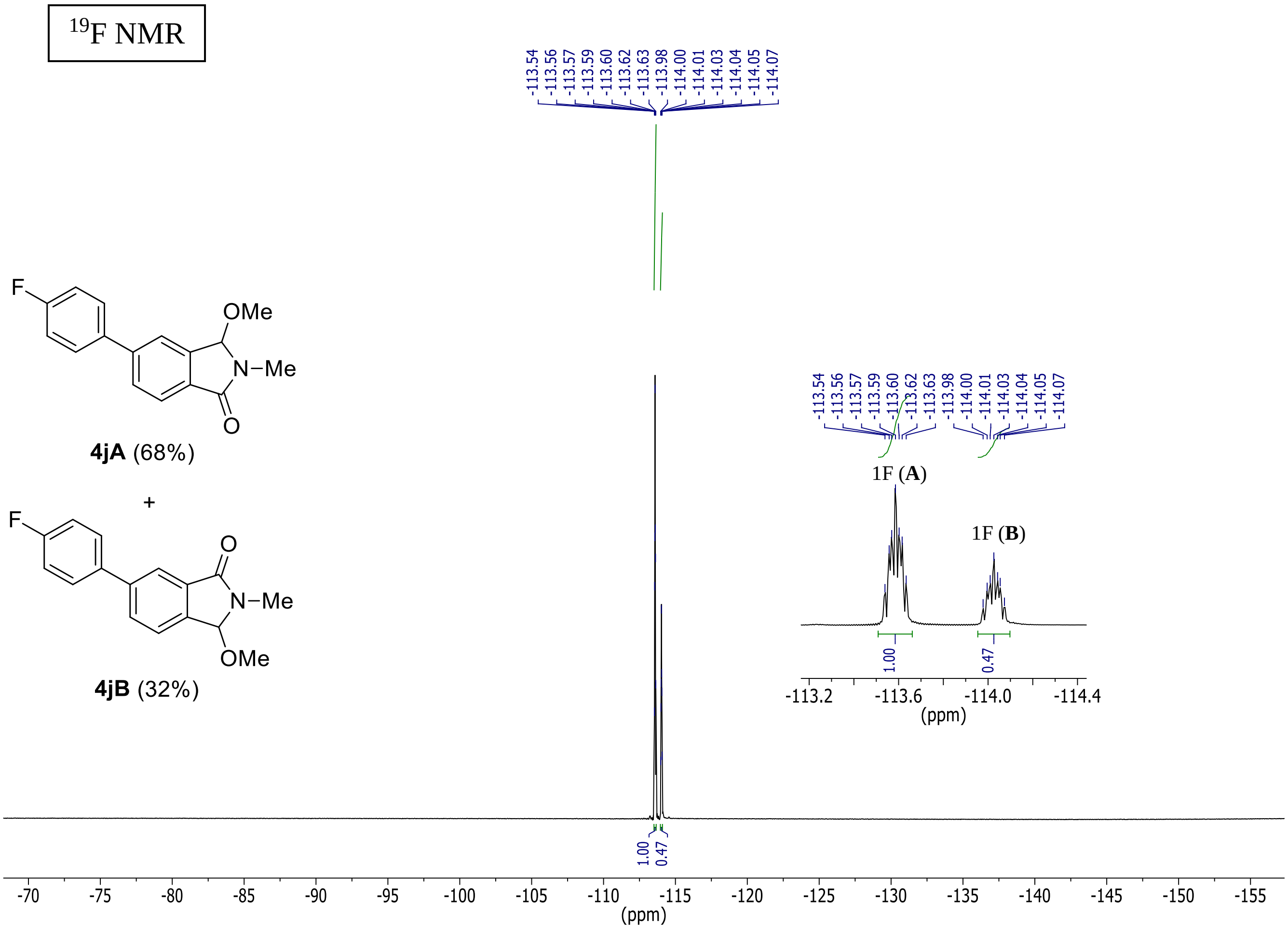

19F NMR
4jA (68%)
+
4jB (32%)
-113.54
-113.56
-113.57
-113.59
-113.60
-113.62
-113.63
-113.98
-114.00
-114.01
-114.03
-114.04
-114.05
-114.07
1F (A)
1F (B)
1.00
0.47
-113.2
-113.6
-114.0
-114.4
(ppm)
-70
-75
-80
-85
-90
-95
-100
-105
-110
-115
-120
-125
-130
-135
-140
-145
-150
-155
(ppm)

^{1}H NMR

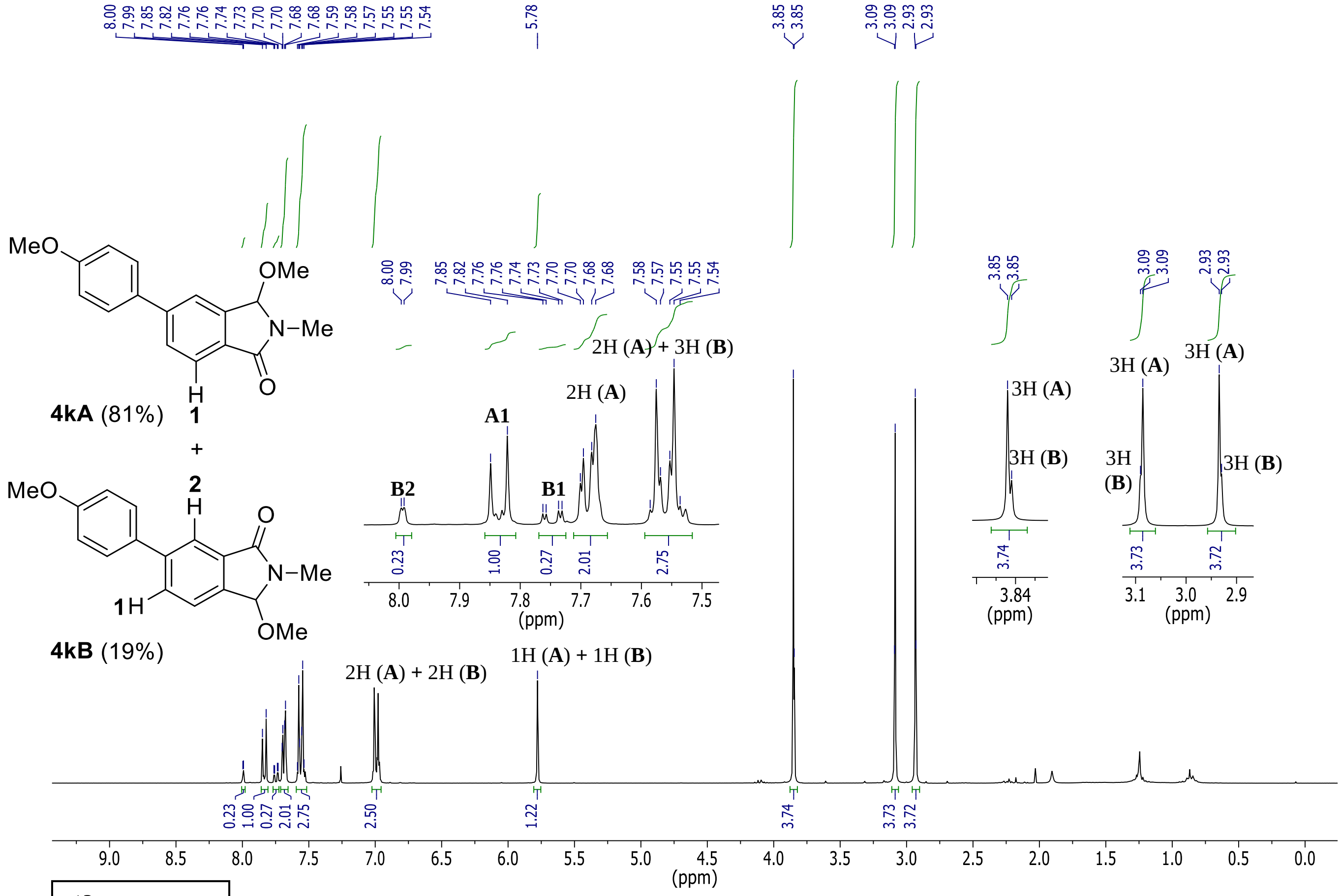

^{13}C NMR

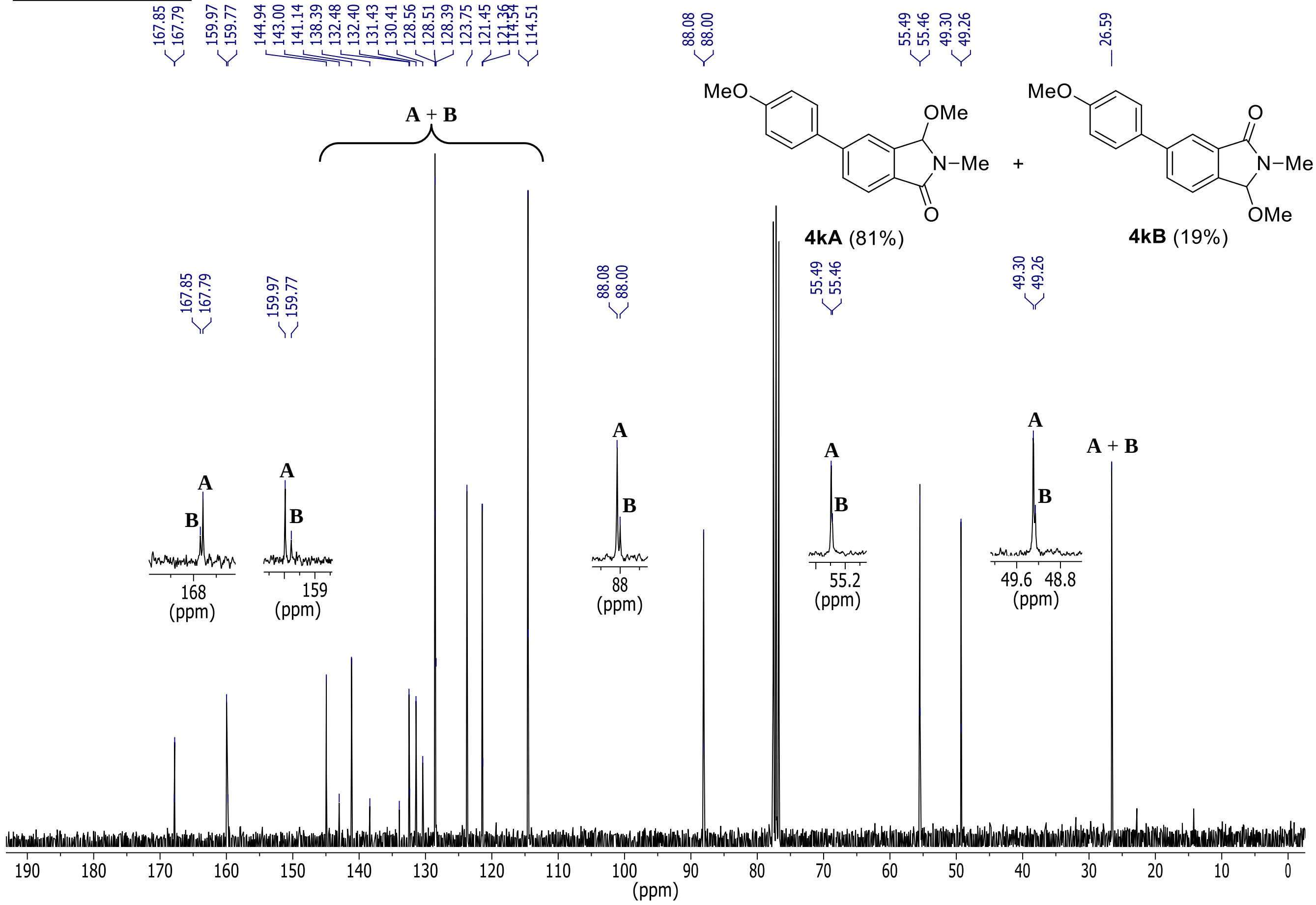

^{1}H NMR

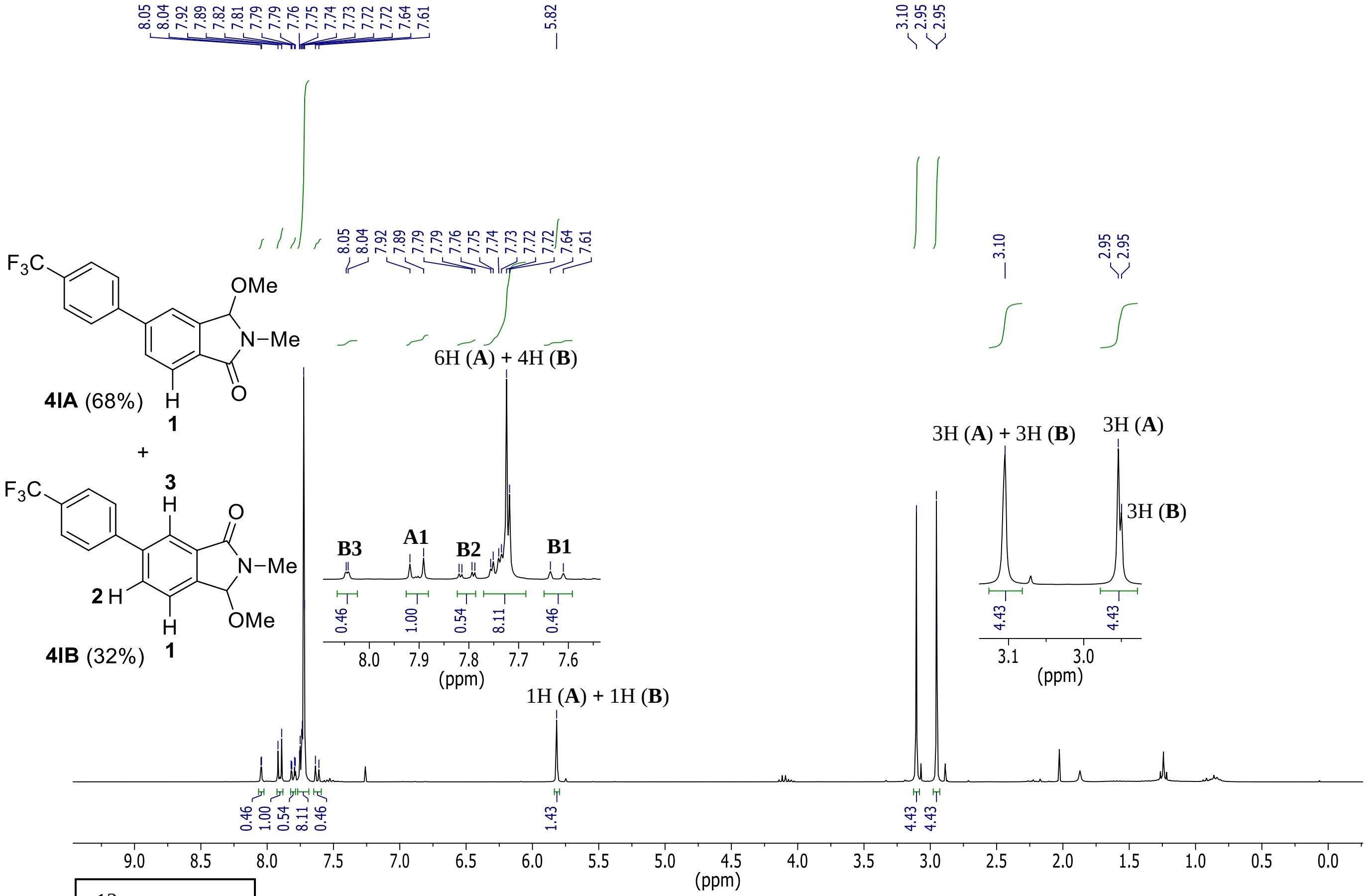

^{13}C NMR

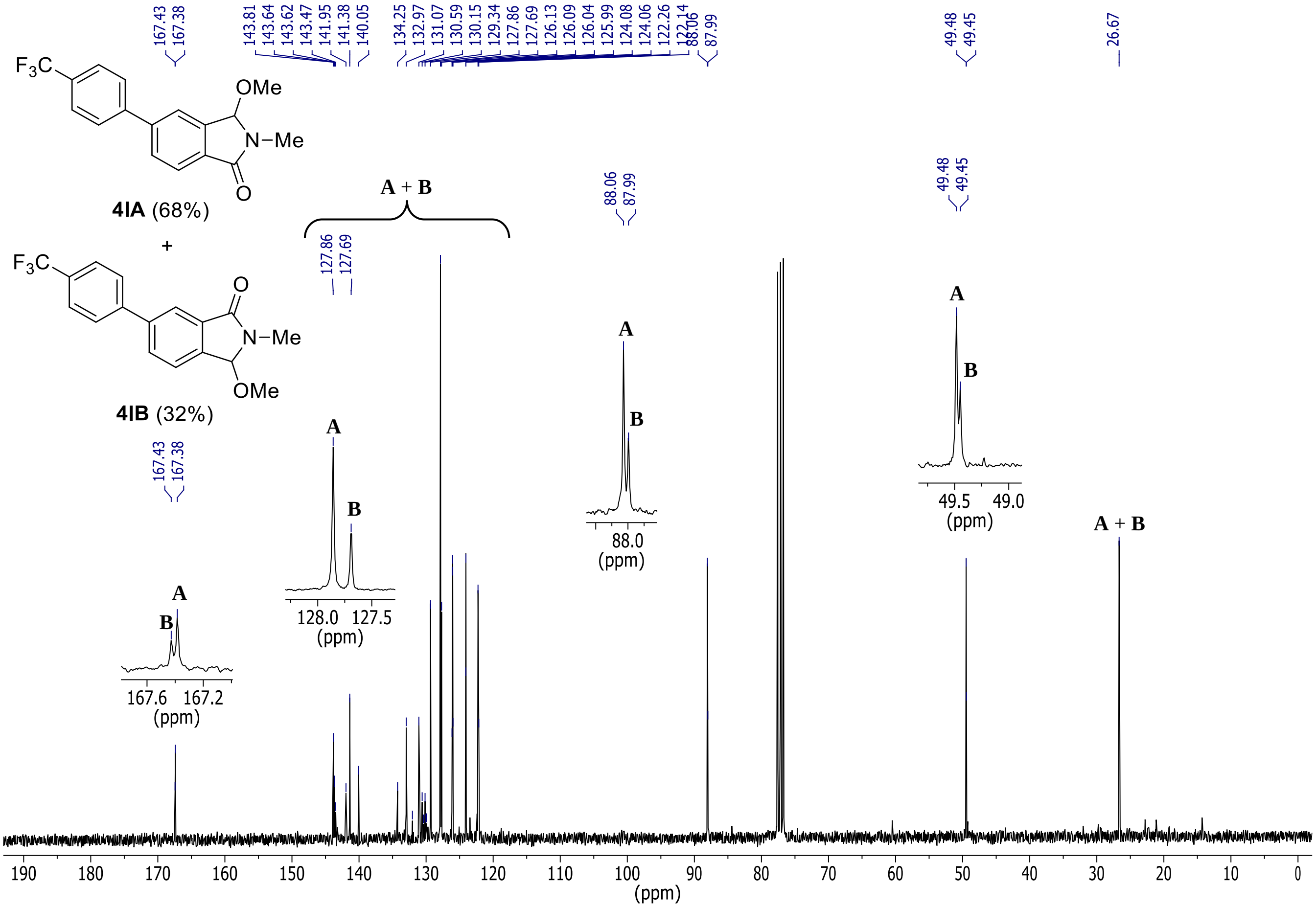

^{1}H NMR

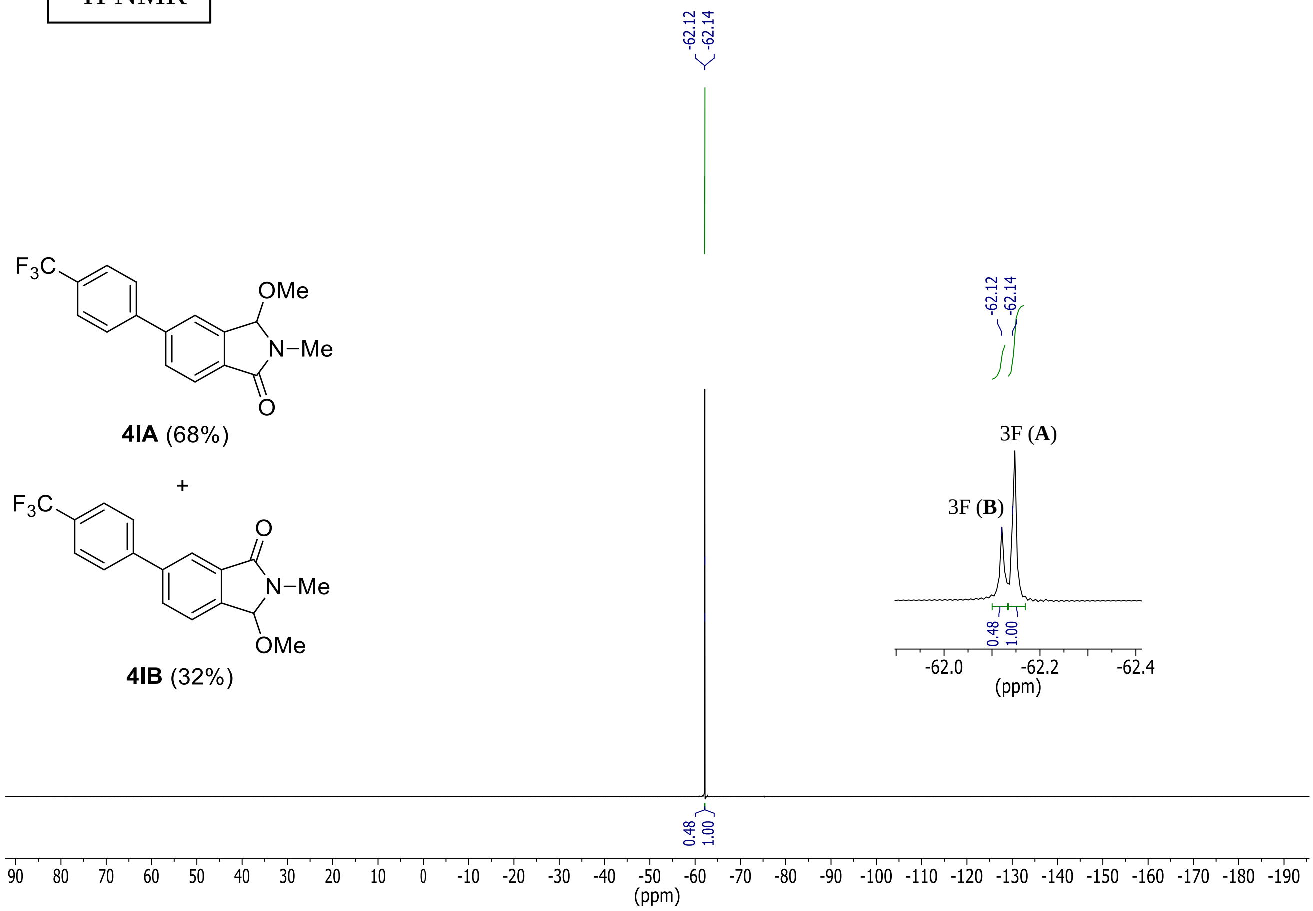
-62.12
-62.14
F3C
OMe
N−Me
O
4IA (68%)
+
4IB (32%)
3F (A)
3F (B)
0.48
1.00
-62.0
-62.2
-62.4
(ppm)
90 80 70 60 50 40 30 20 10 0 -10 -20 -30 -40 -50 -60 -70 -80 -90 -100 -110 -120 -130 -140 -150 -160 -170 -180 -190

^{1}H NMR

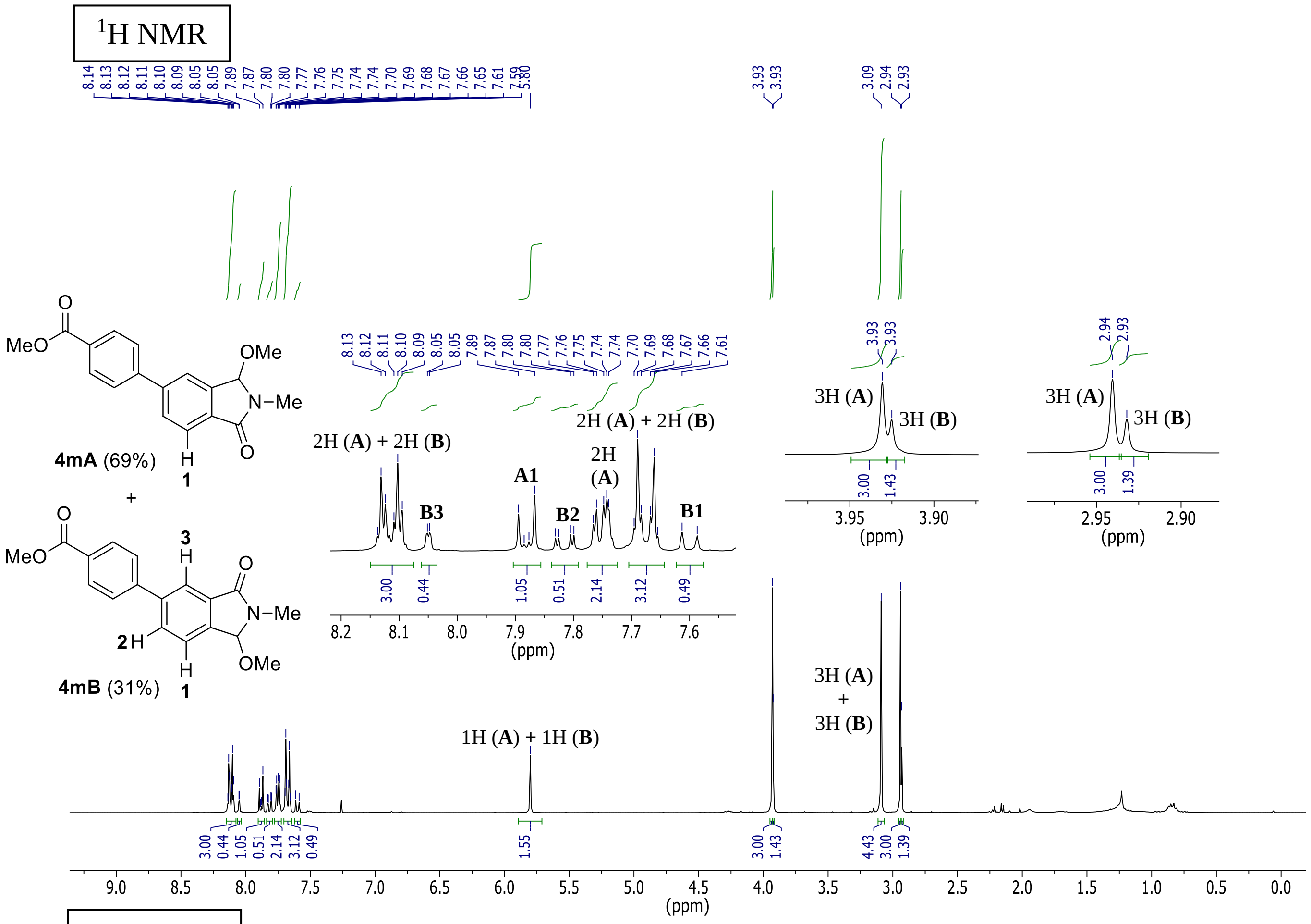

^{13}C NMR

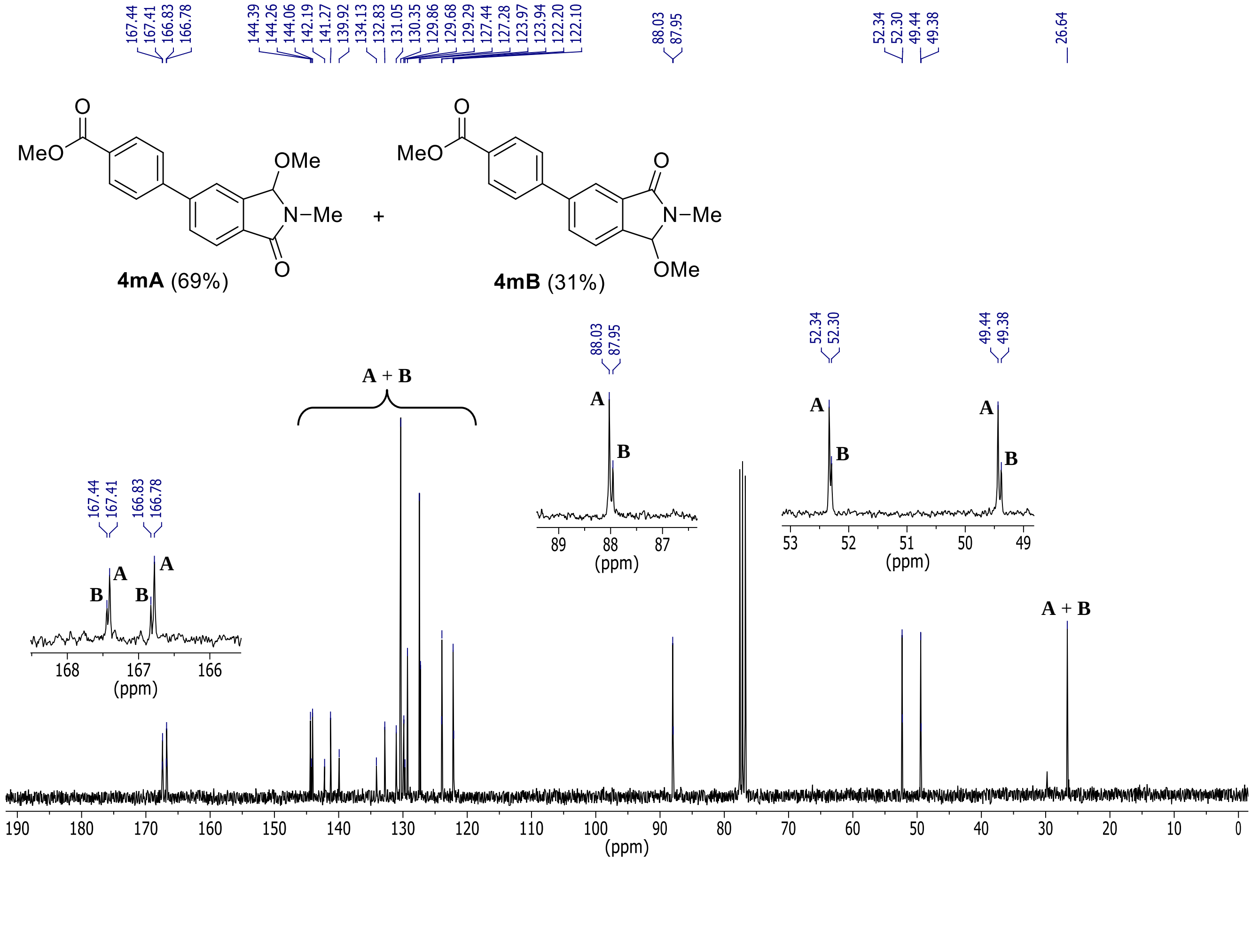

^{1}H NMR

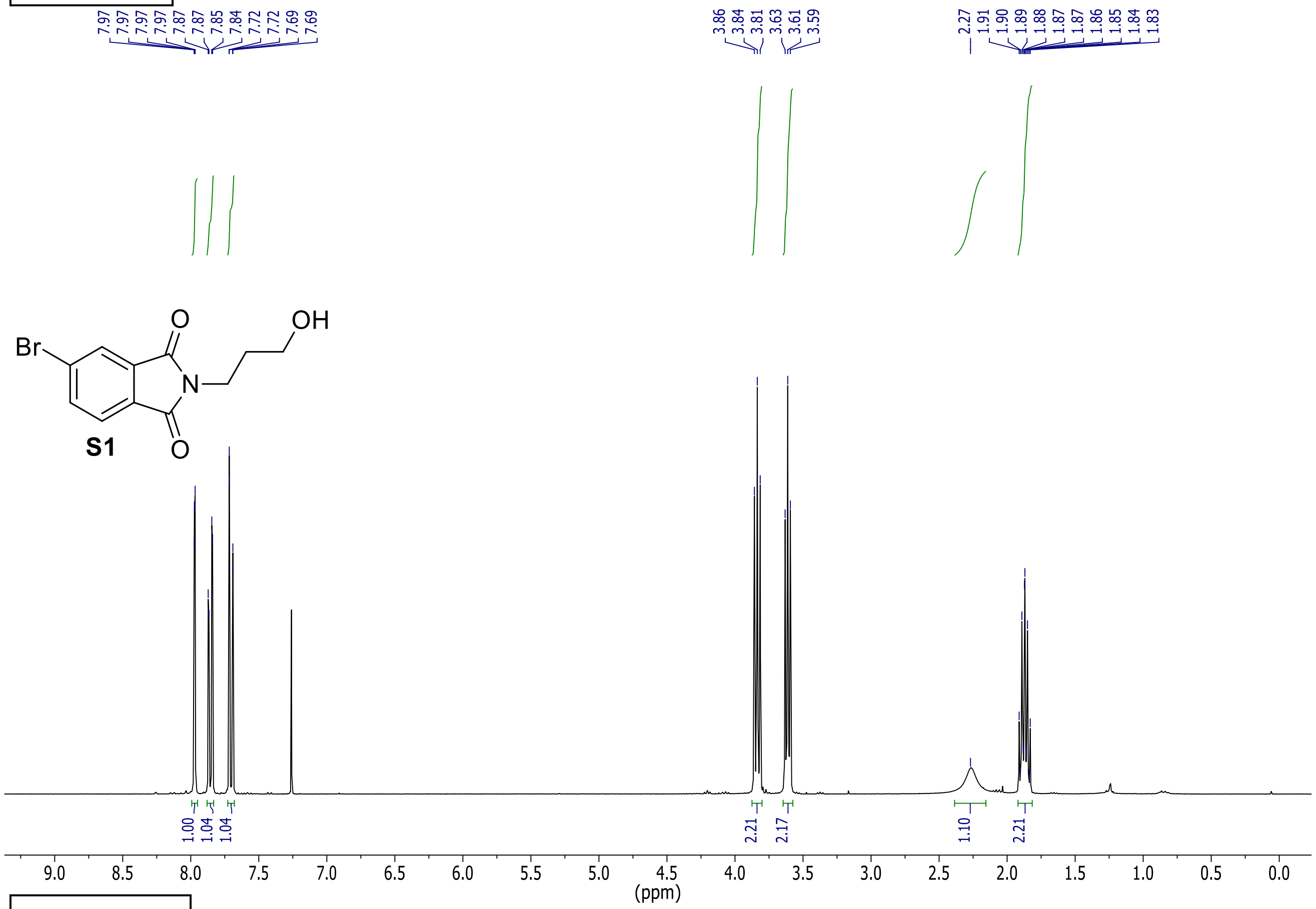

^{13}C NMR

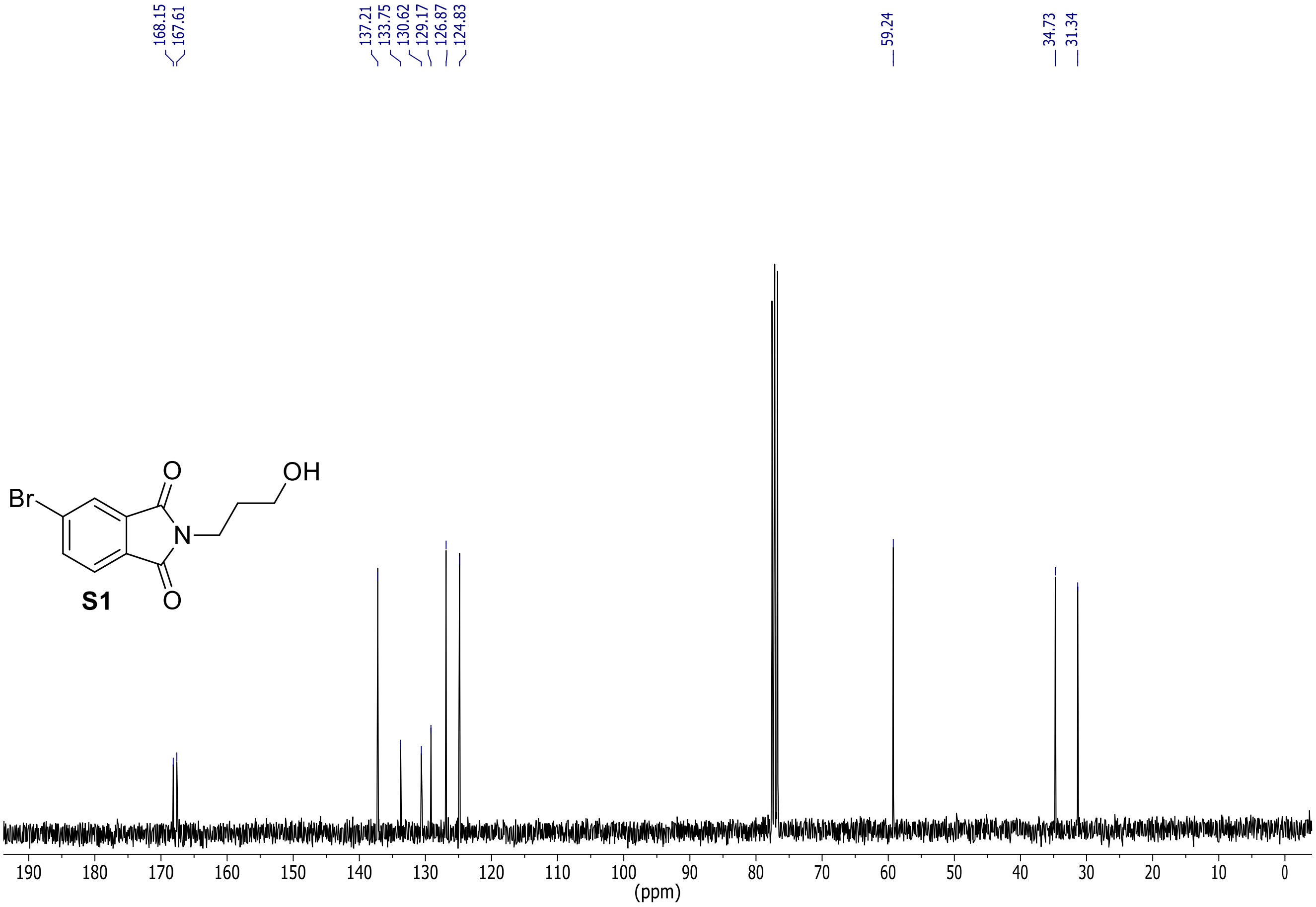

^{1}H NMR

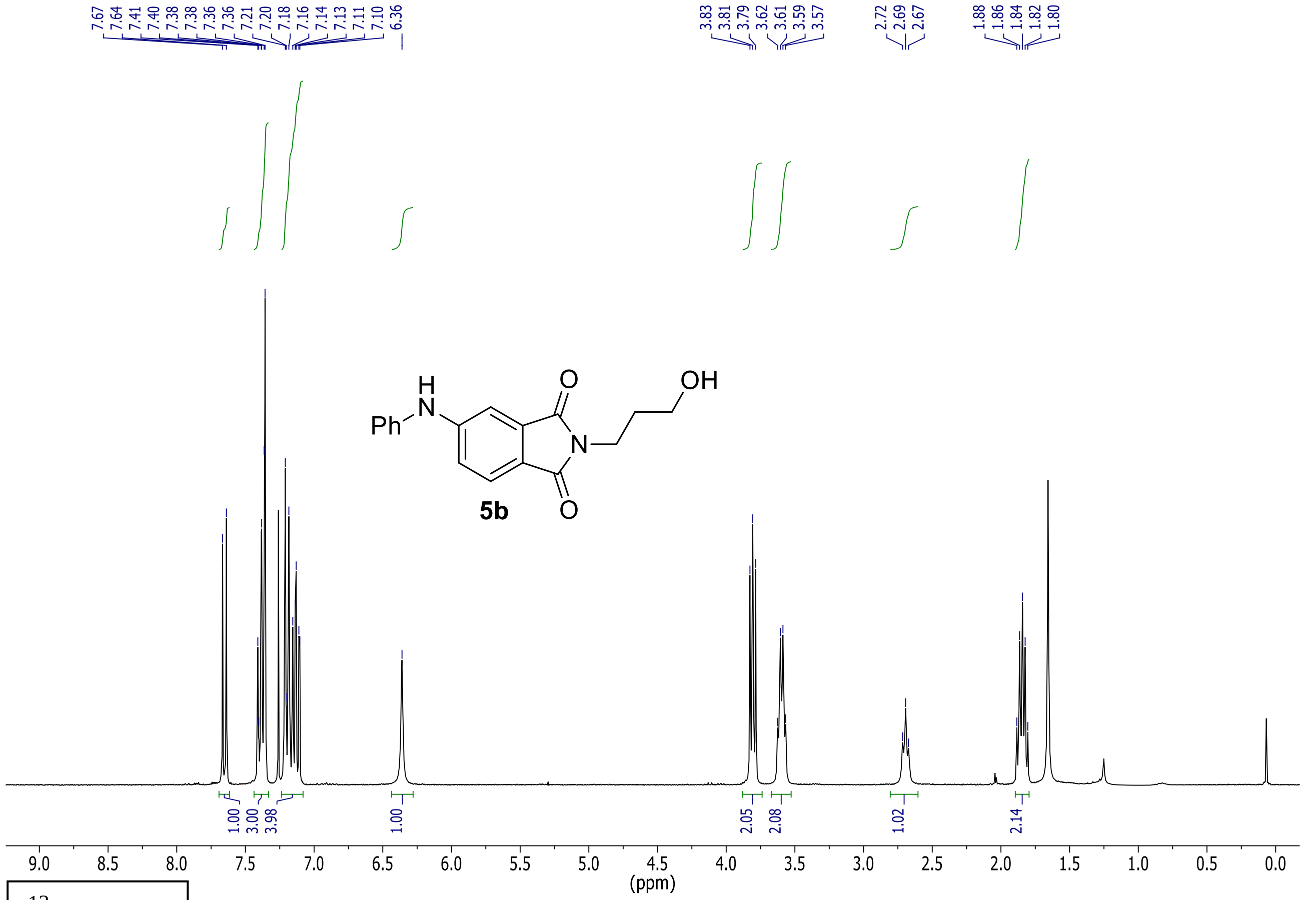

^{13}C NMR

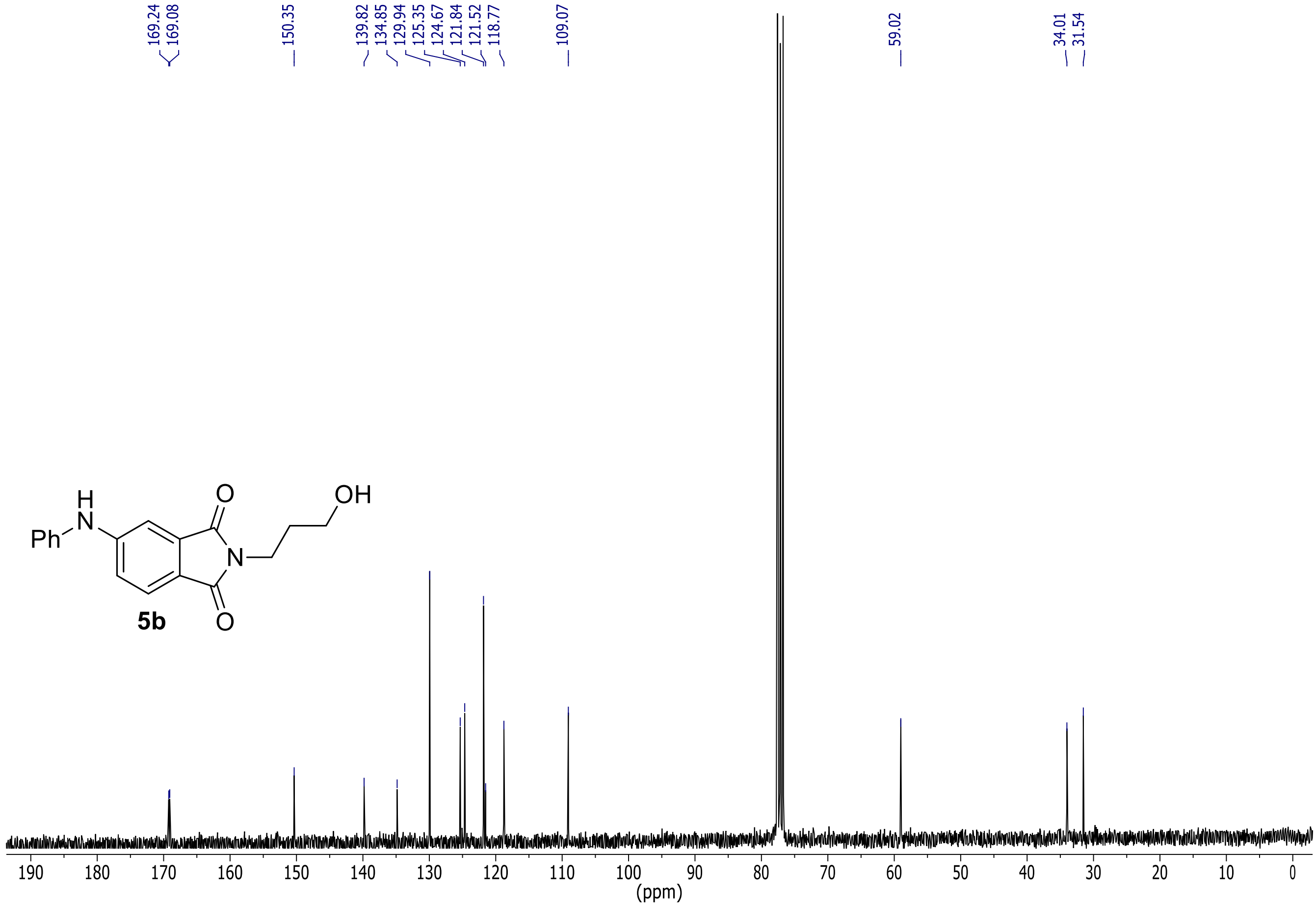

^{1}H NMR

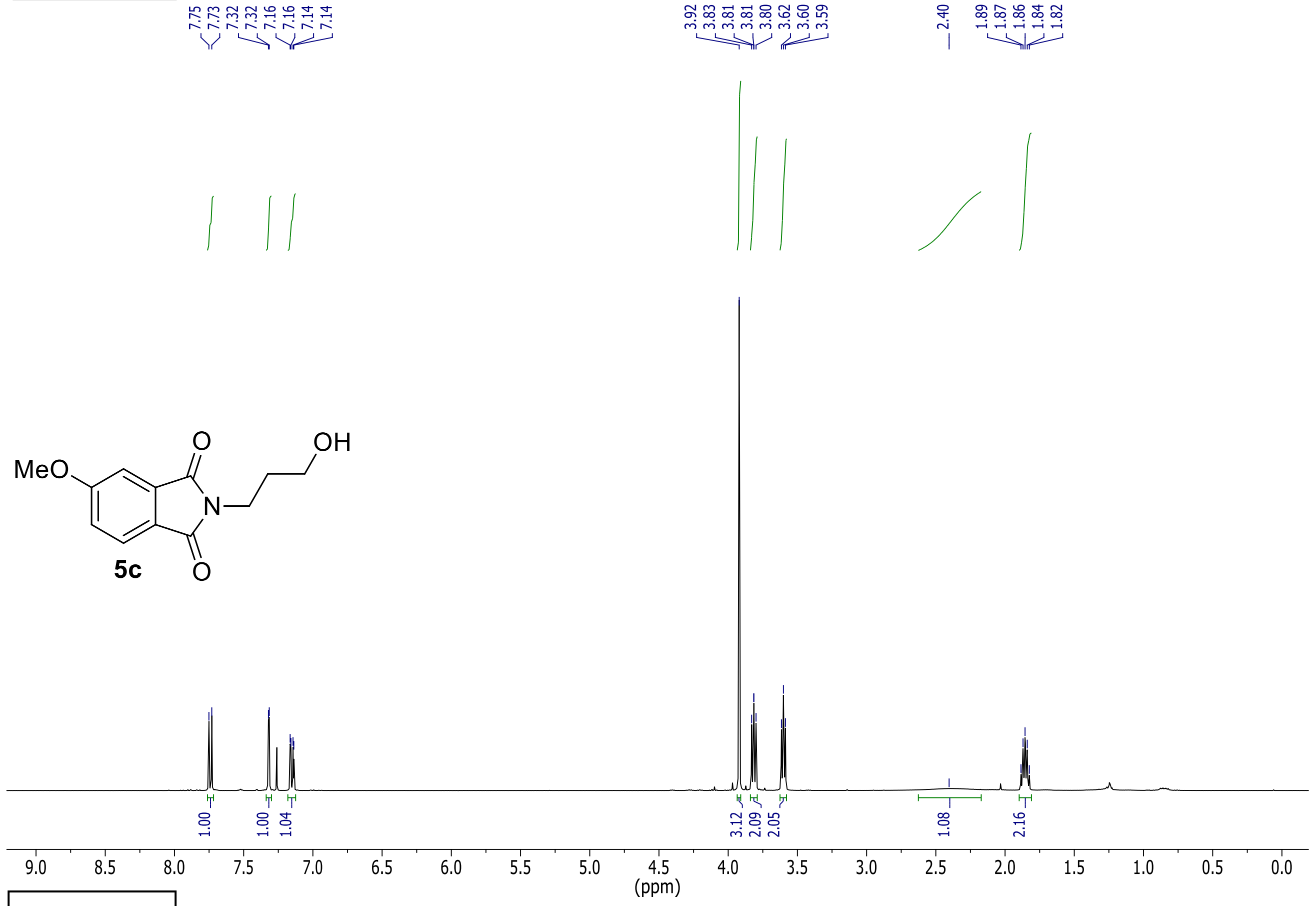

^{13}C NMR

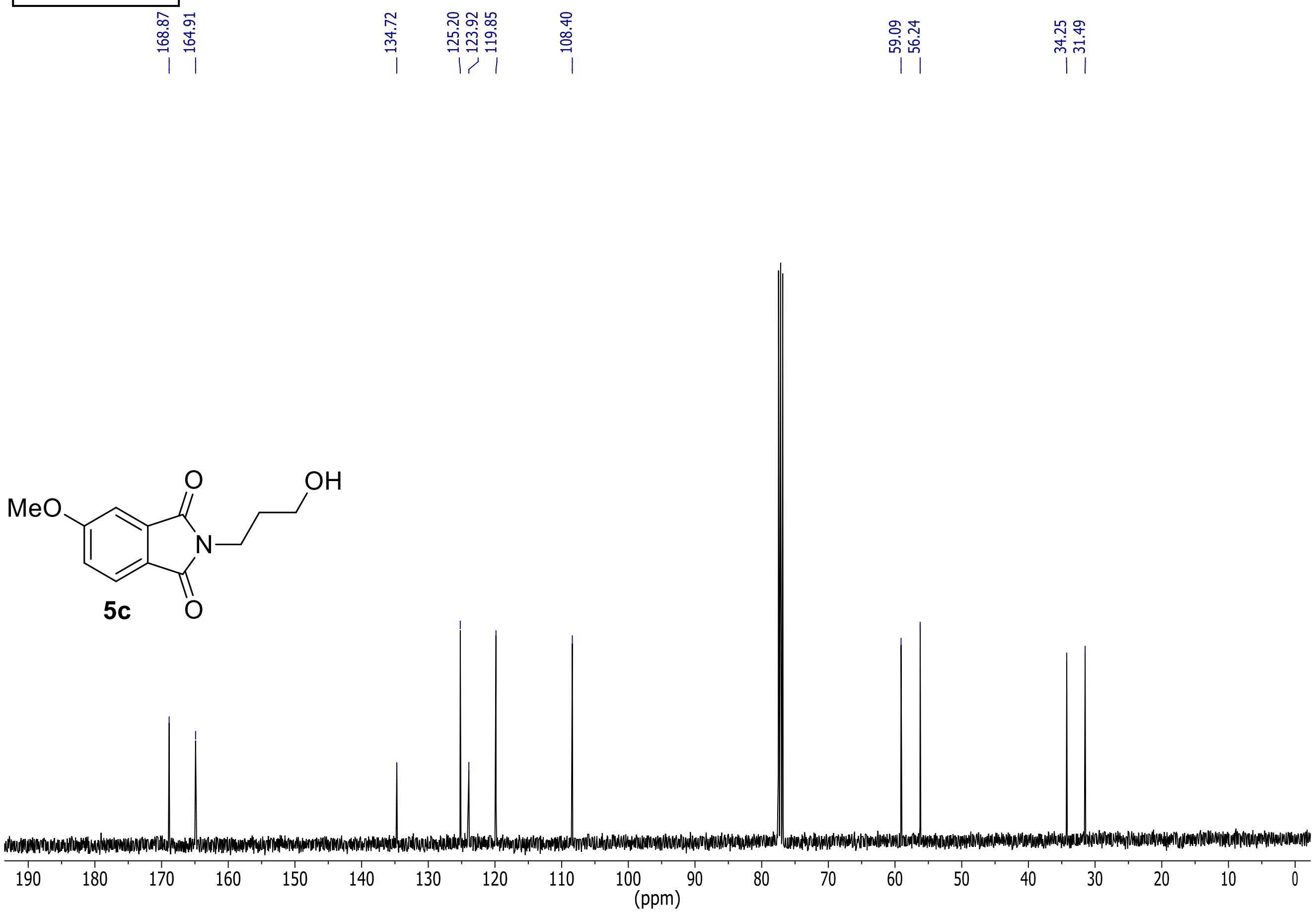

^{1}H NMR

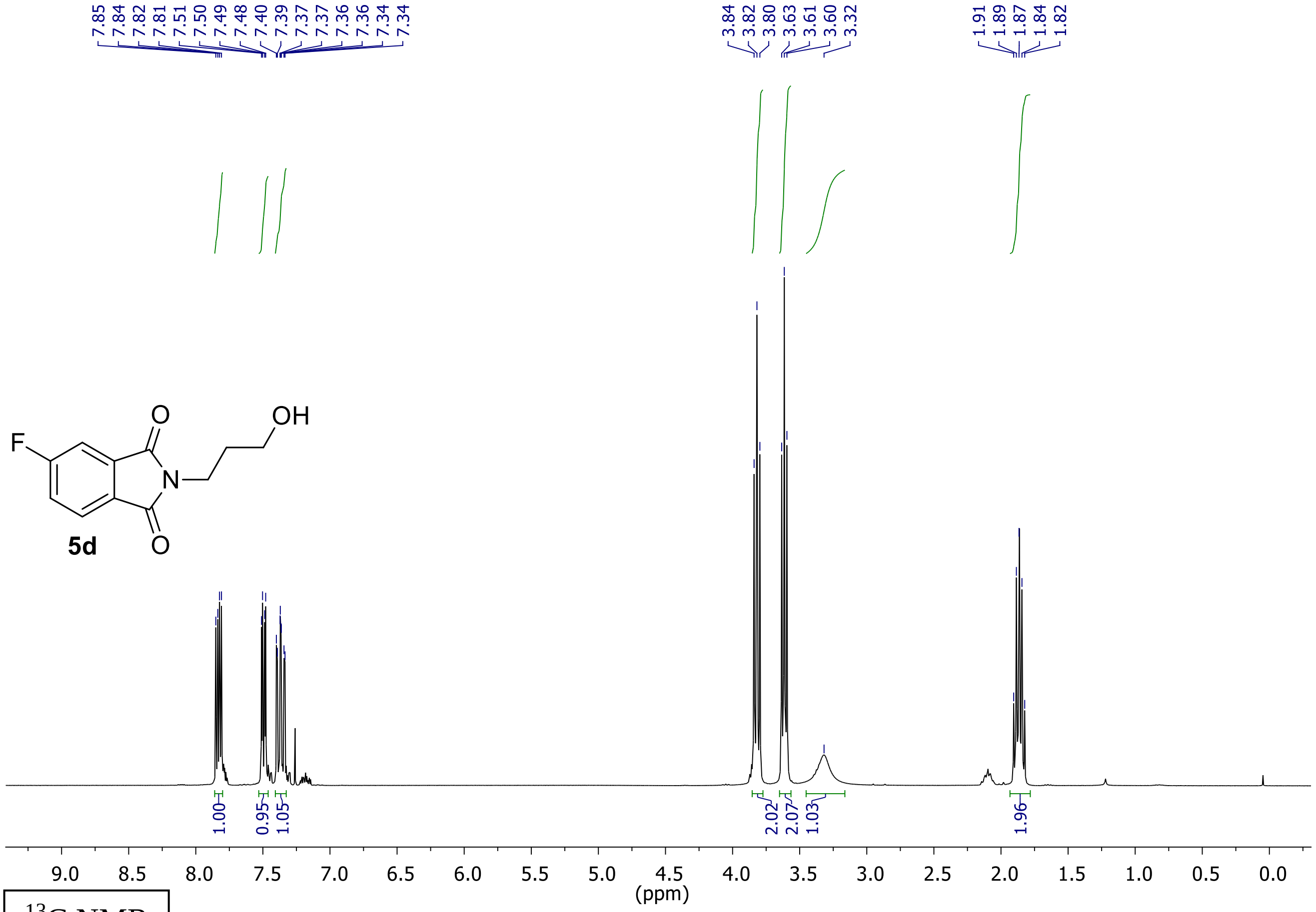

^{13}C NMR

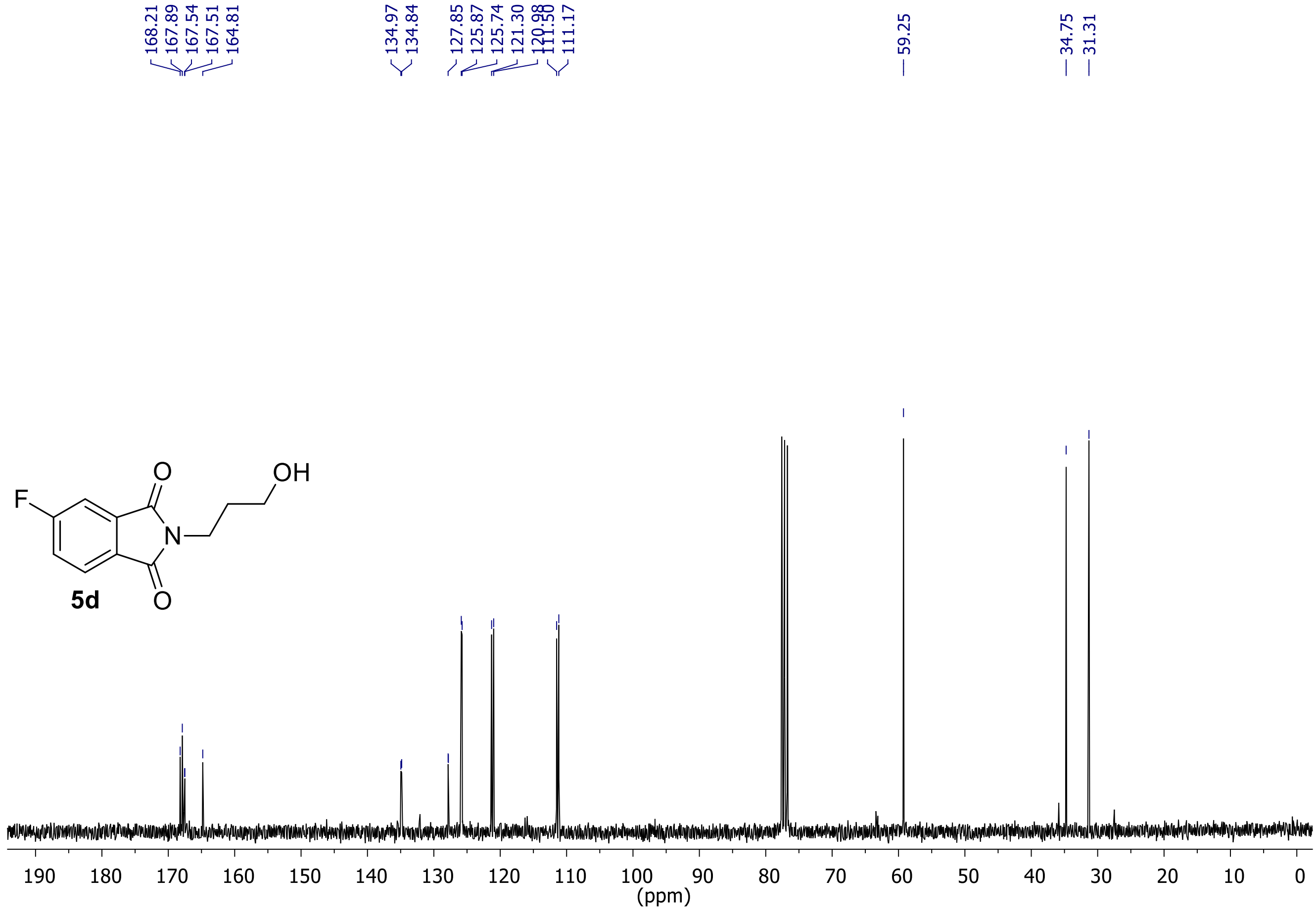

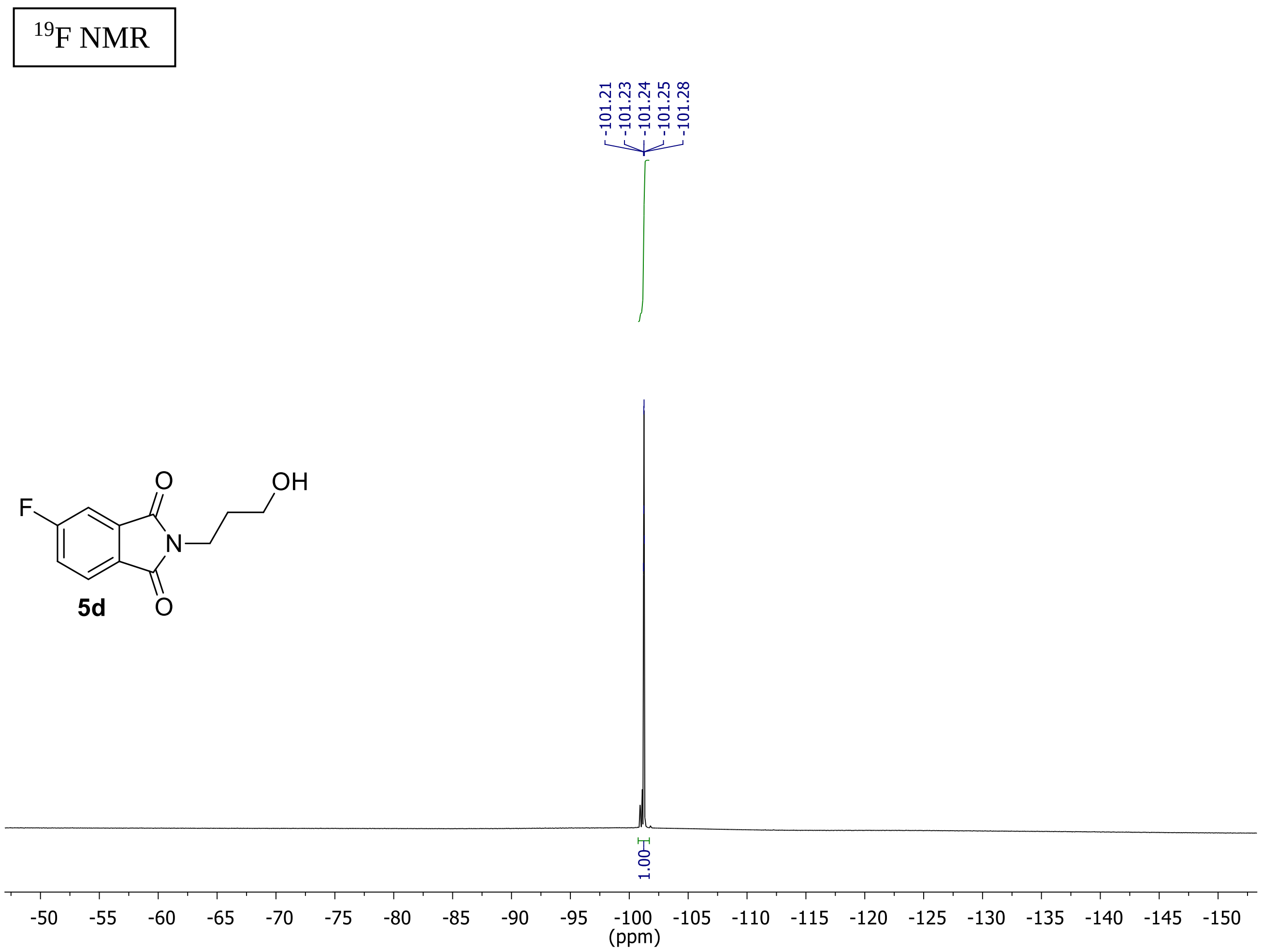

19F NMR
-101.21
-101.23
-101.24
-101.25
-101.28
F
O
OH
N
5d
O
1.00
-50 -55 -60 -65 -70 -75 -80 -85 -90 -95 -100 -105 -110 -115 -120 -125 -130 -135 -140 -145 -150
(ppm)

^{1}H NMR

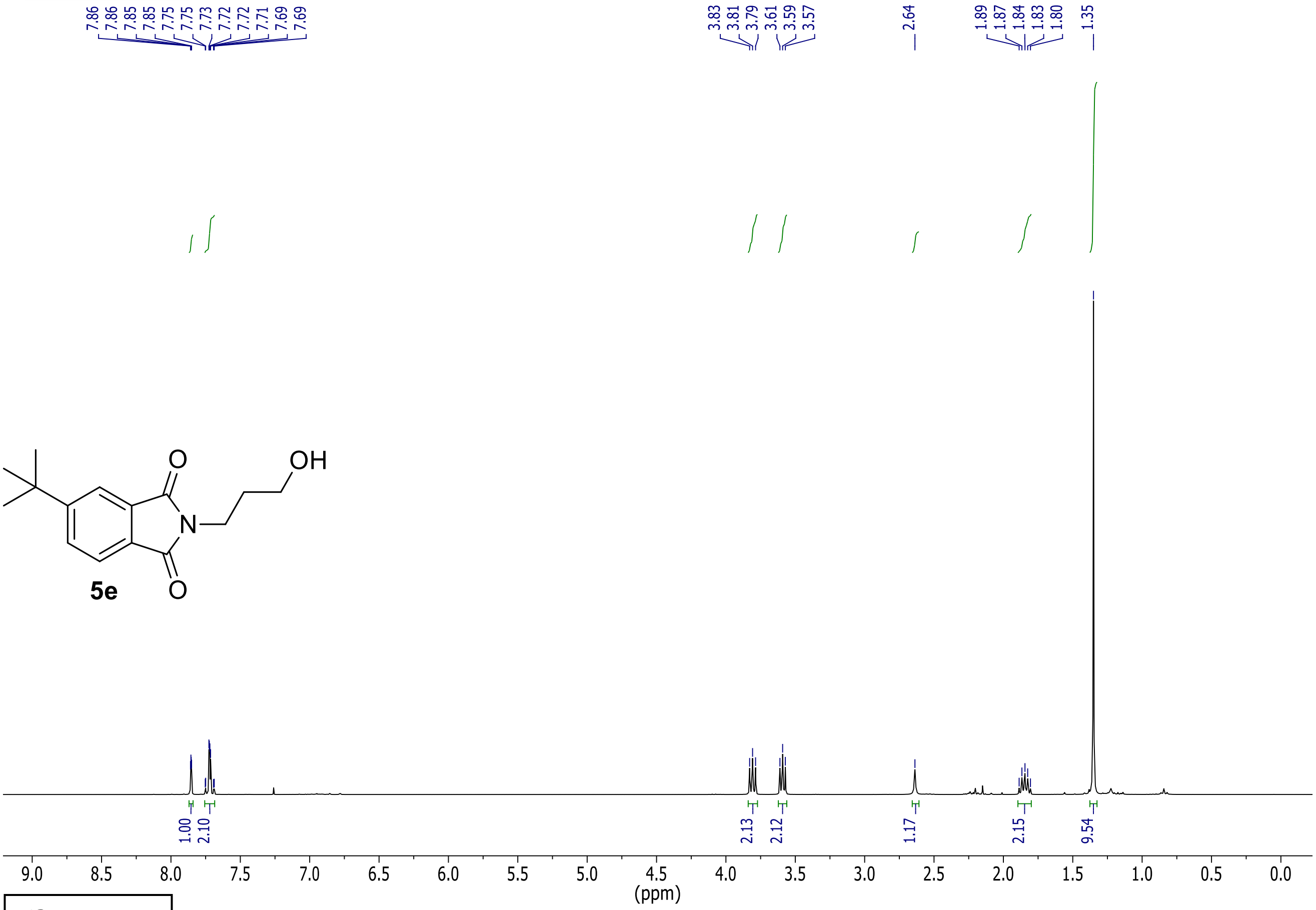

^{13}C NMR

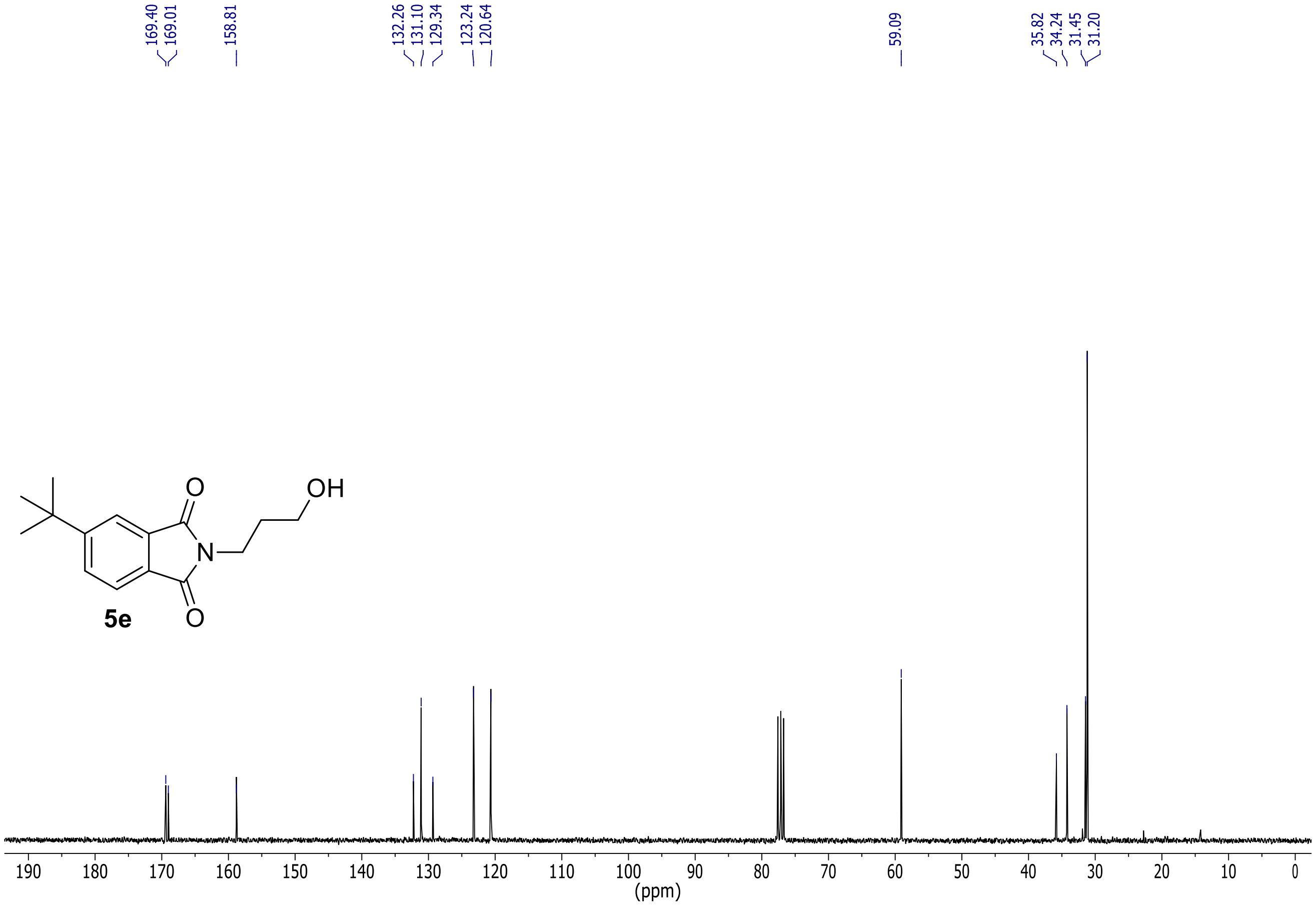

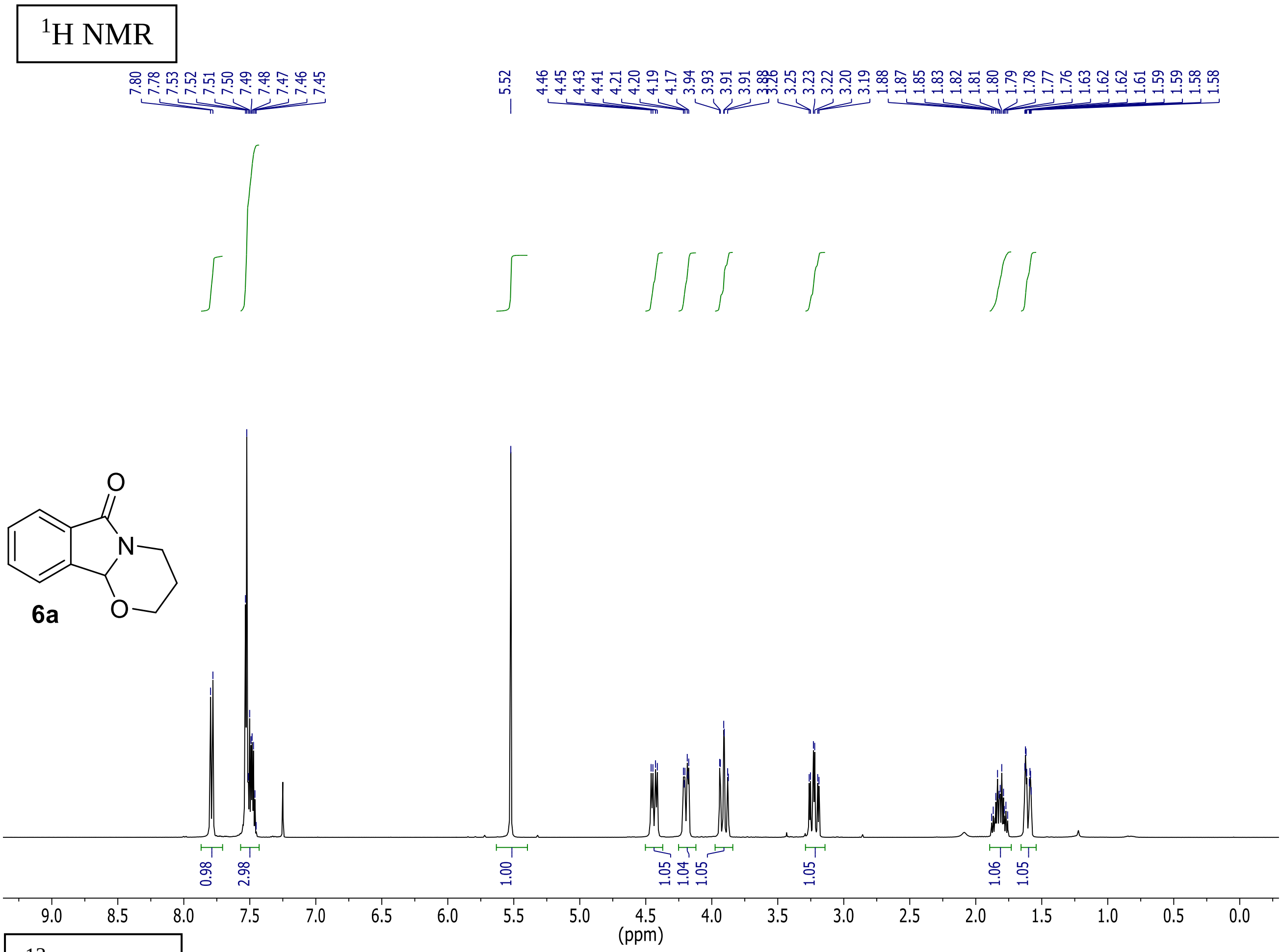

^{1}H NMR
7.80 7.78 7.53 7.52 7.51 7.50 7.49 7.48 7.47 7.46 7.45
5.52
4.46 4.45 4.43 4.41 4.21 4.20 4.19 4.17 3.94 3.93 3.91 3.91
3.25 3.23 3.22 3.20 3.19
1.88 1.87 1.85 1.83 1.82 1.81 1.80 1.79 1.78 1.77 1.76 1.63 1.62 1.62 1.61 1.59 1.59 1.58 1.58
0.98 2.98 1.00 1.05 1.04 1.05 1.05 1.06 1.05
O
N
O
6a
9.0 8.5 8.0 7.5 7.0 6.5 6.0 5.5 5.0 4.5 4.0 3.5 3.0 2.5 2.0 1.5 1.0 0.5 0.0
(ppm)

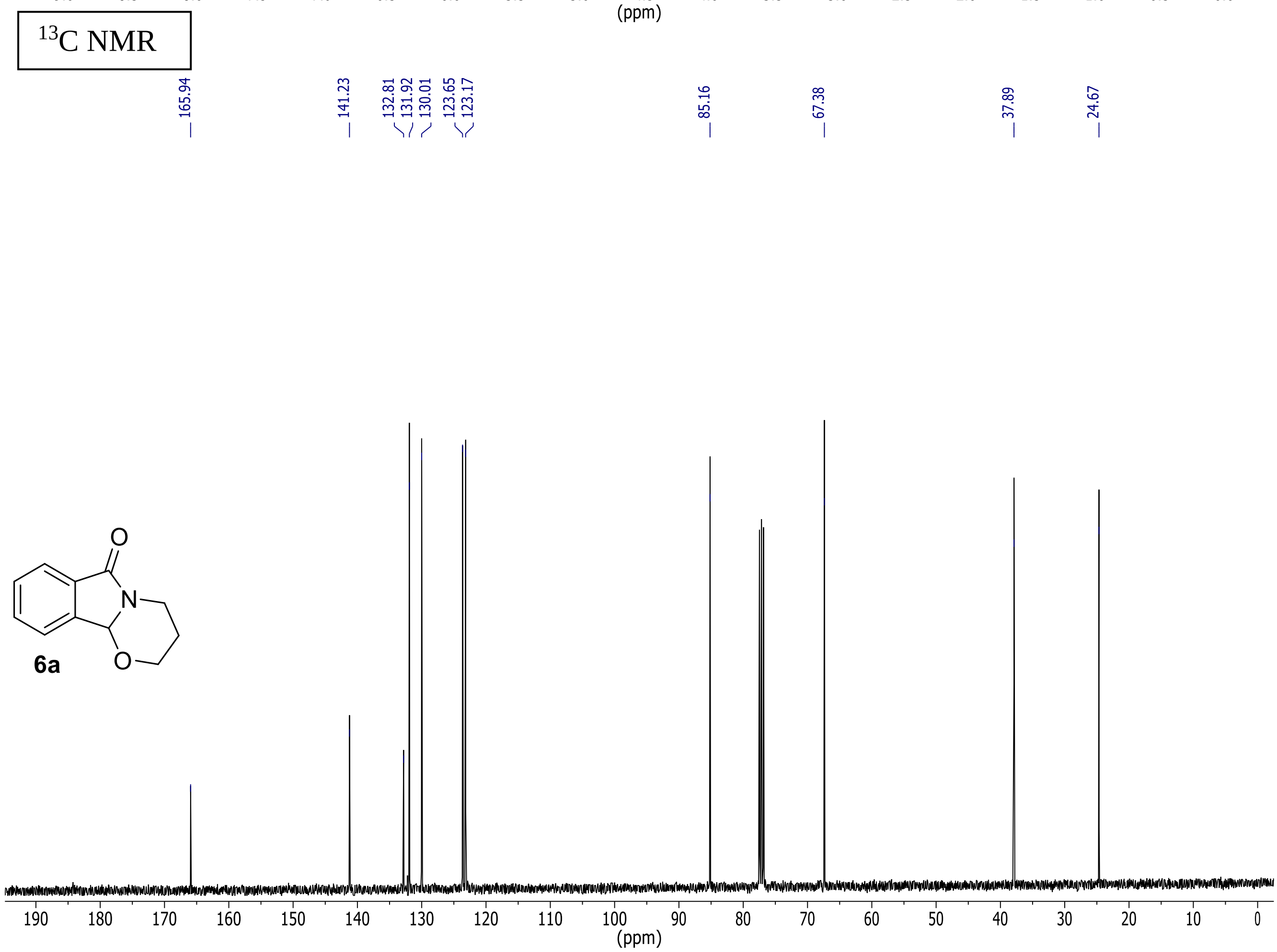

^{13}C NMR
165.94
141.23
132.81 131.92 130.01
123.65 123.17
85.16
67.38
37.89
24.67
O
N
O
6a
190 180 170 160 150 140 130 120 110 100 90 80 70 60 50 40 30 20 10 0
(ppm)

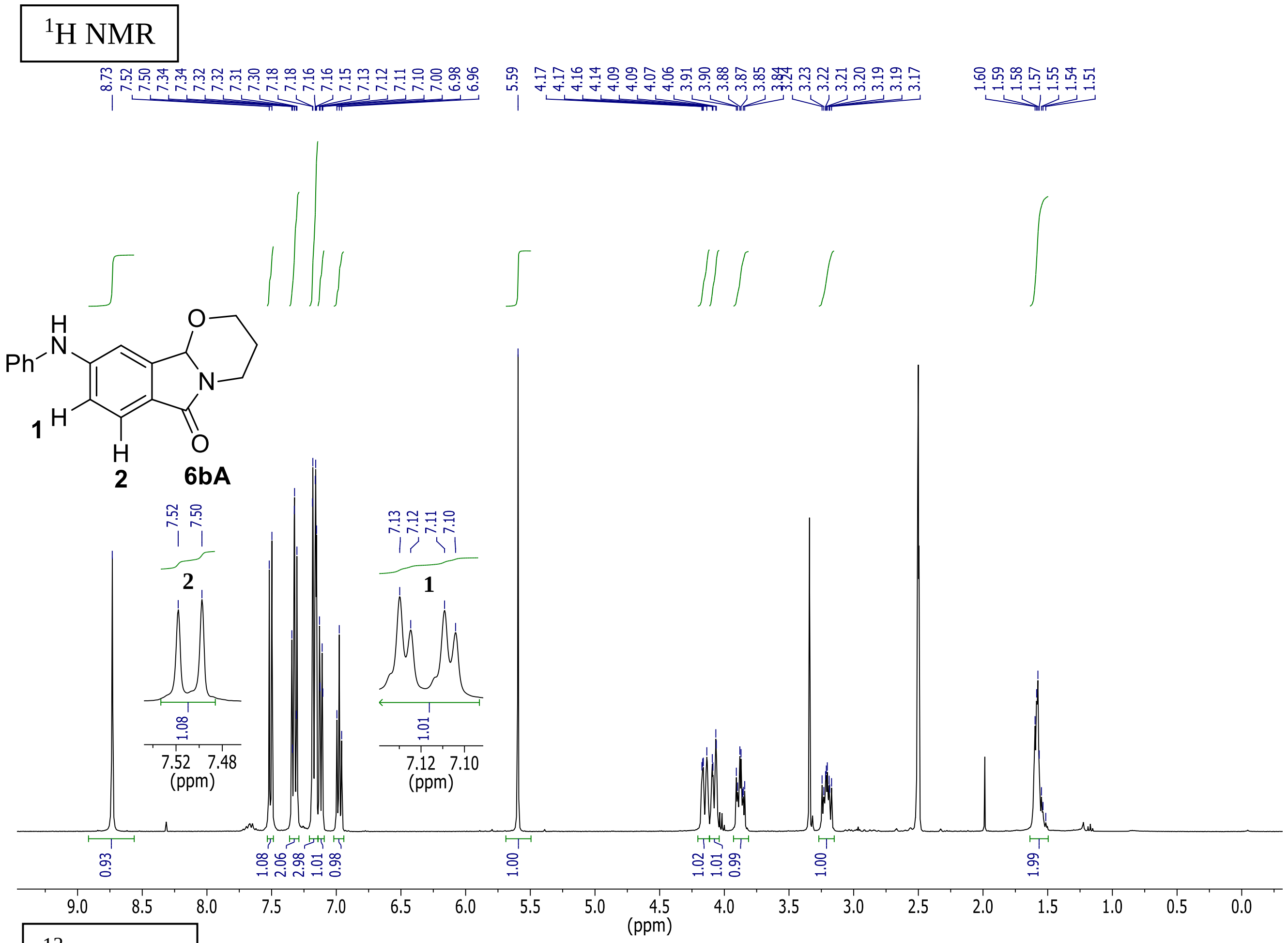
1H NMR
6bA
1
2
7.52 7.48
(ppm)
7.12 7.10
(ppm)
(ppm)

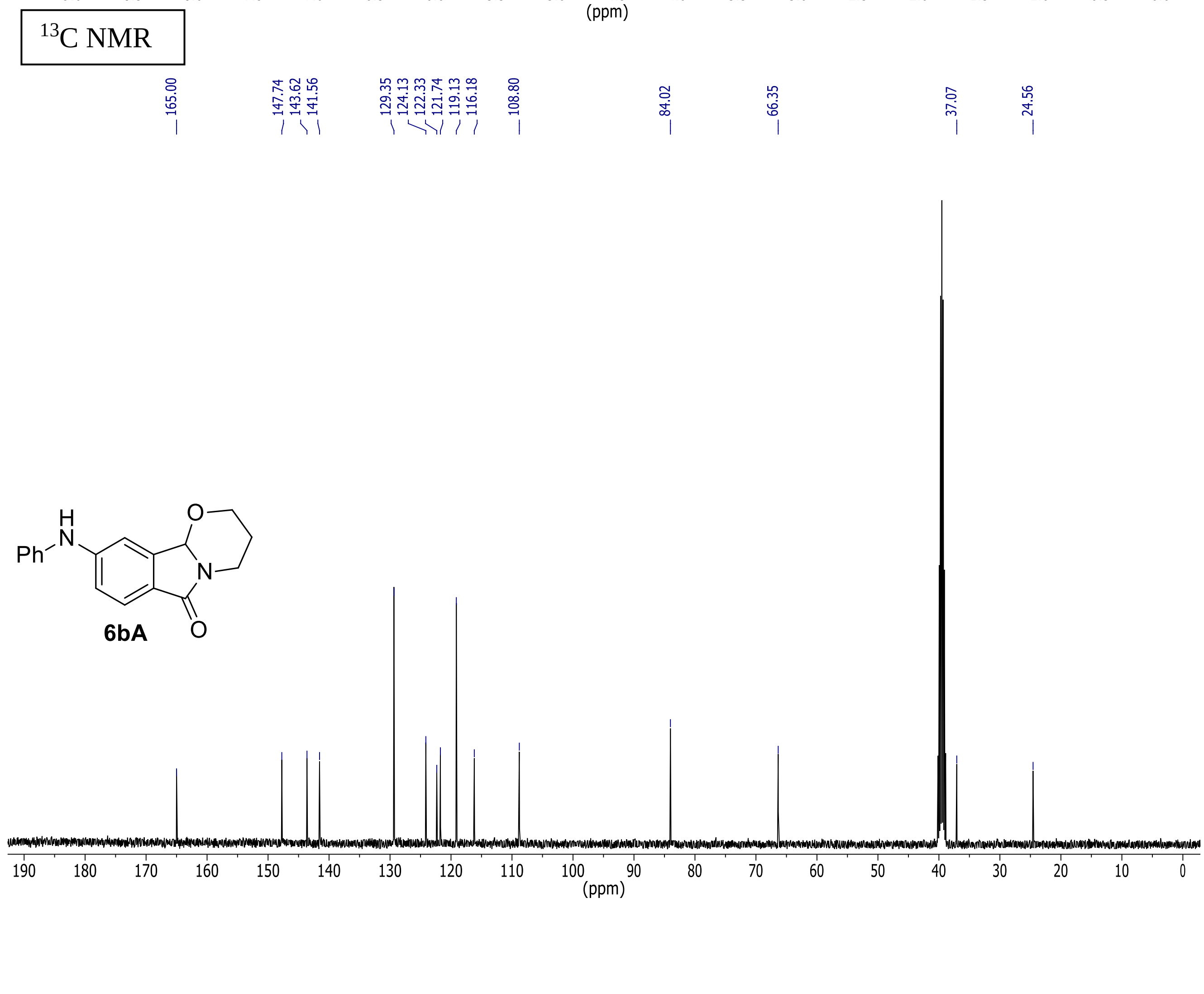
13C NMR
165.00
147.74
143.62
141.56
129.35
124.13
122.33
121.74
119.13
116.18
108.80
84.02
66.35
37.07
24.56
6bA
(ppm)

^{1}H NMR

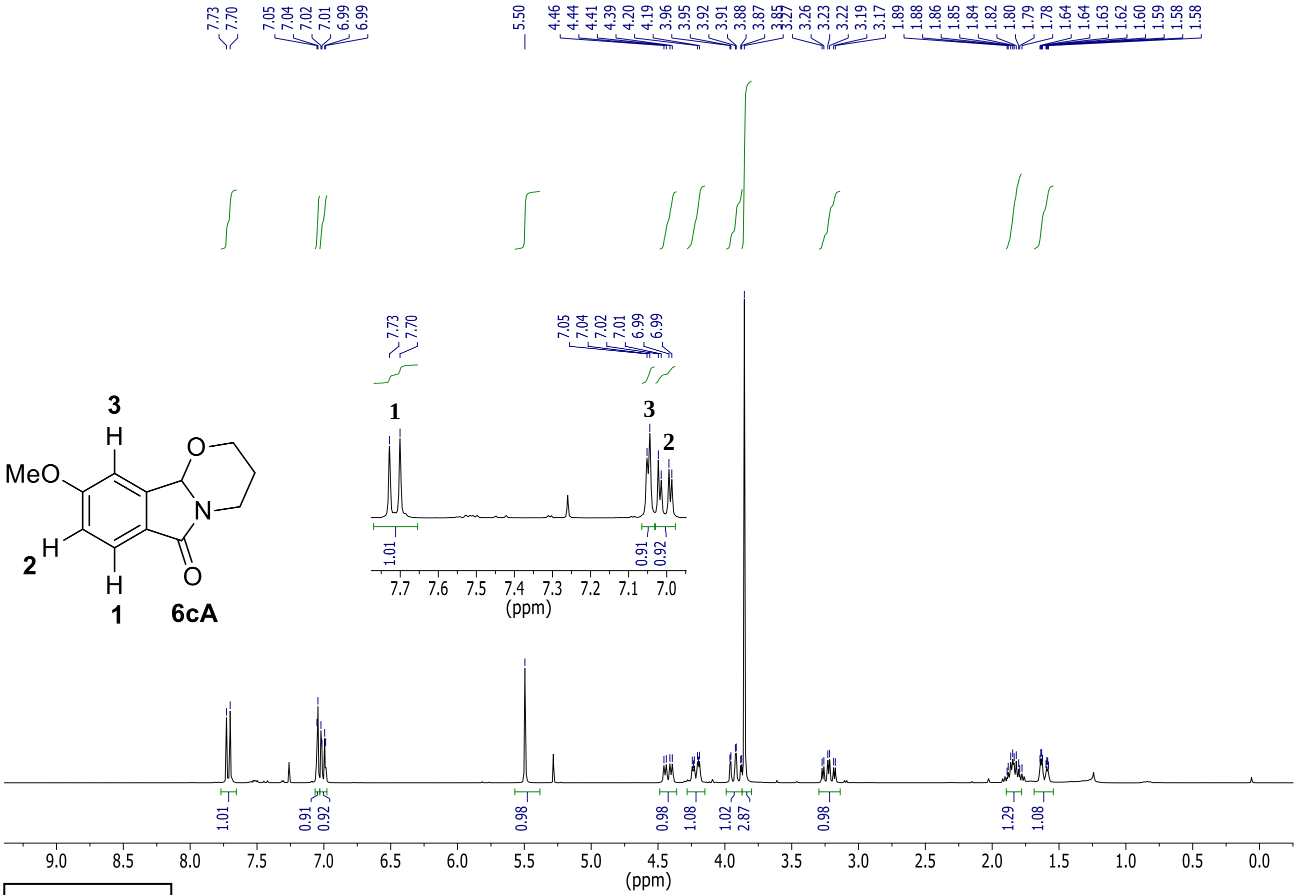

^{13}C NMR

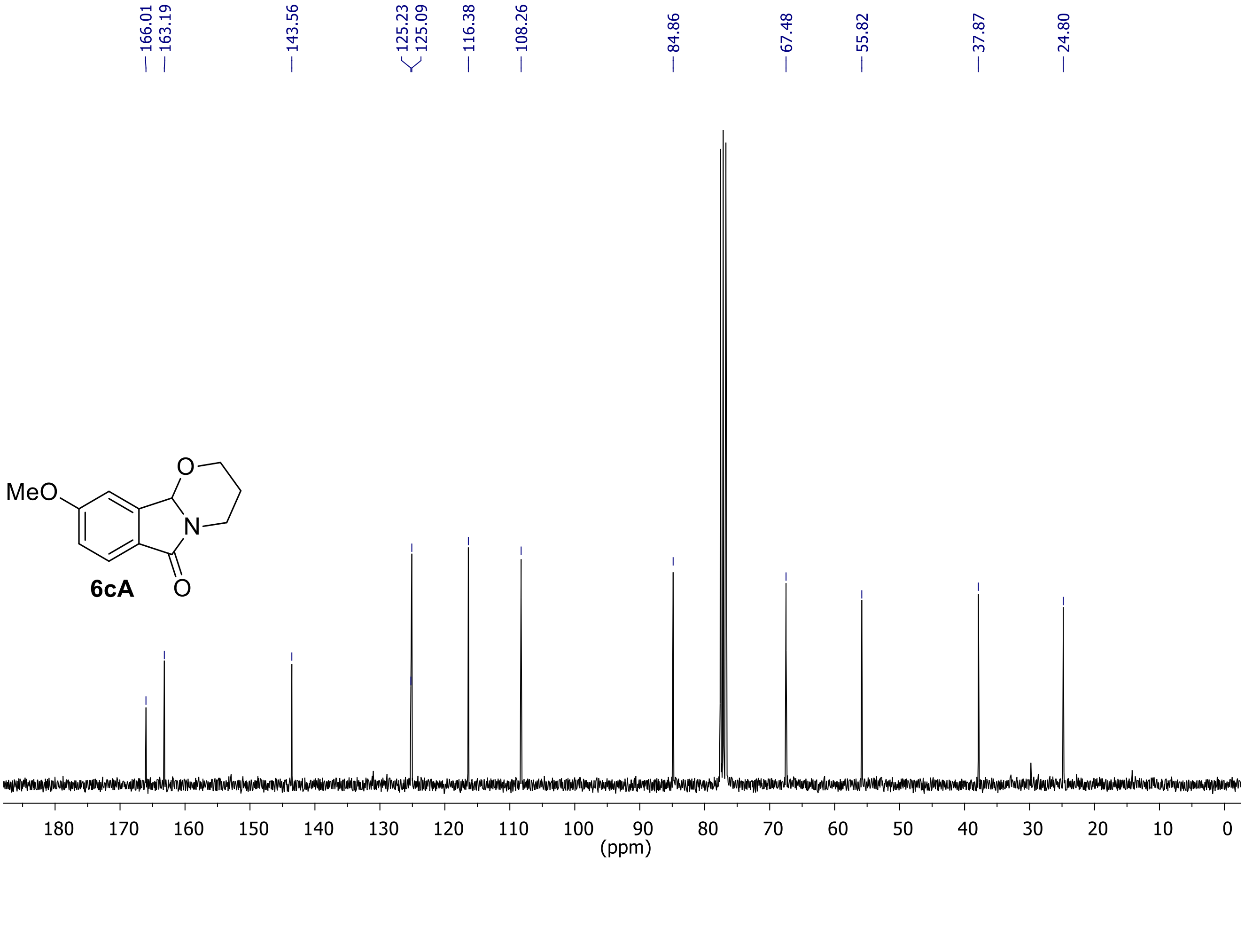

^{1}H NMR

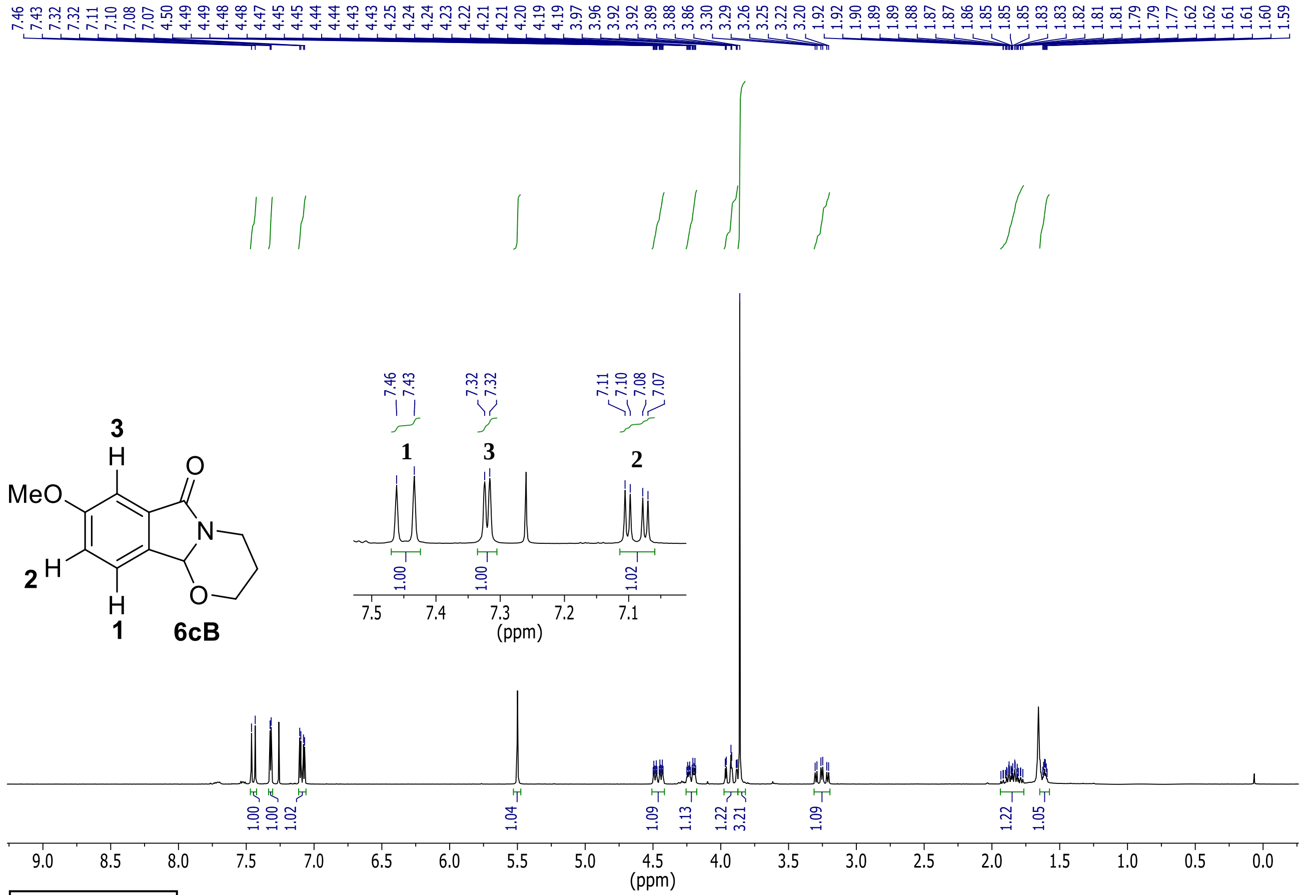

^{13}C NMR

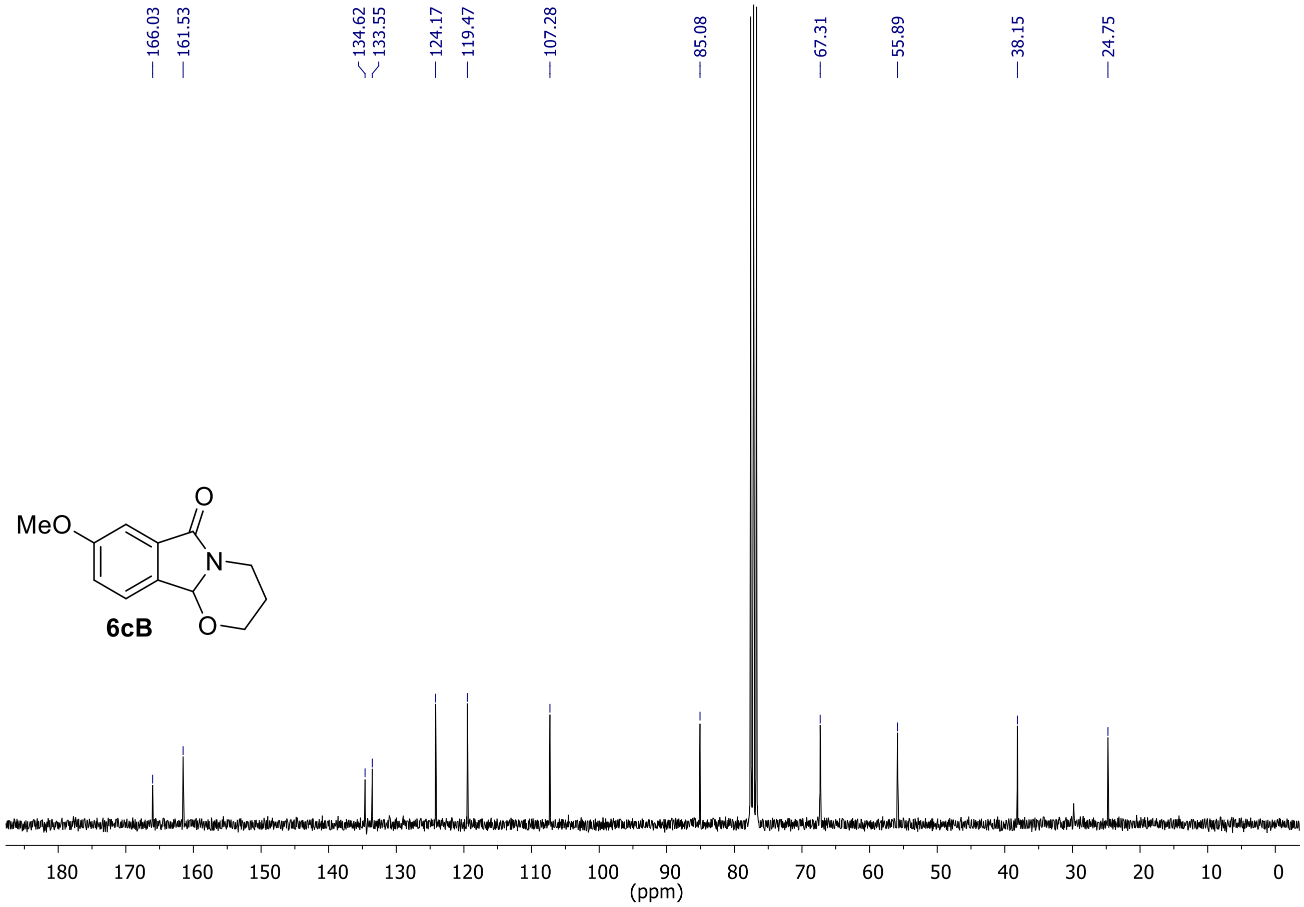

^{1}H NMR

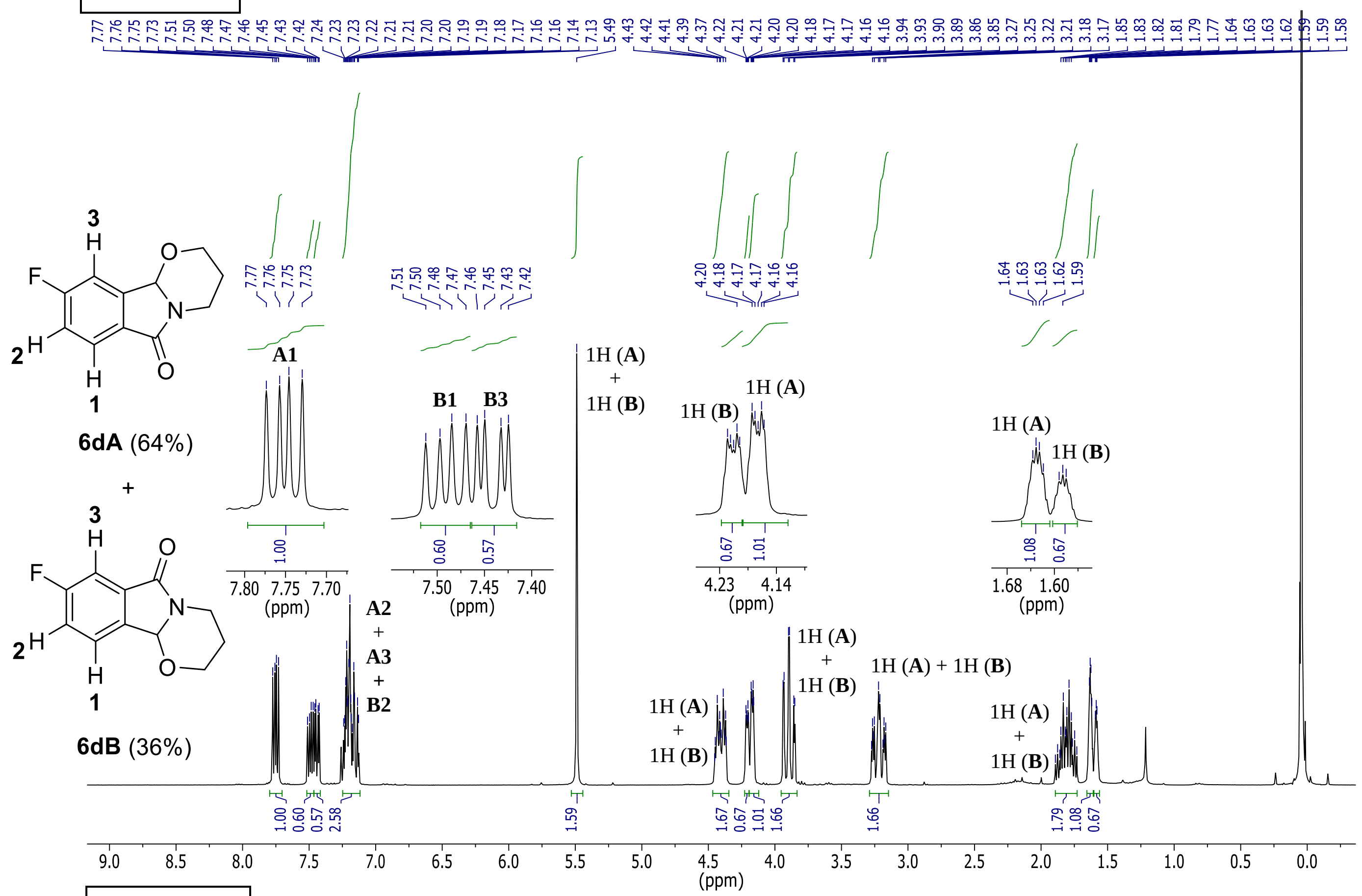

^{13}C NMR

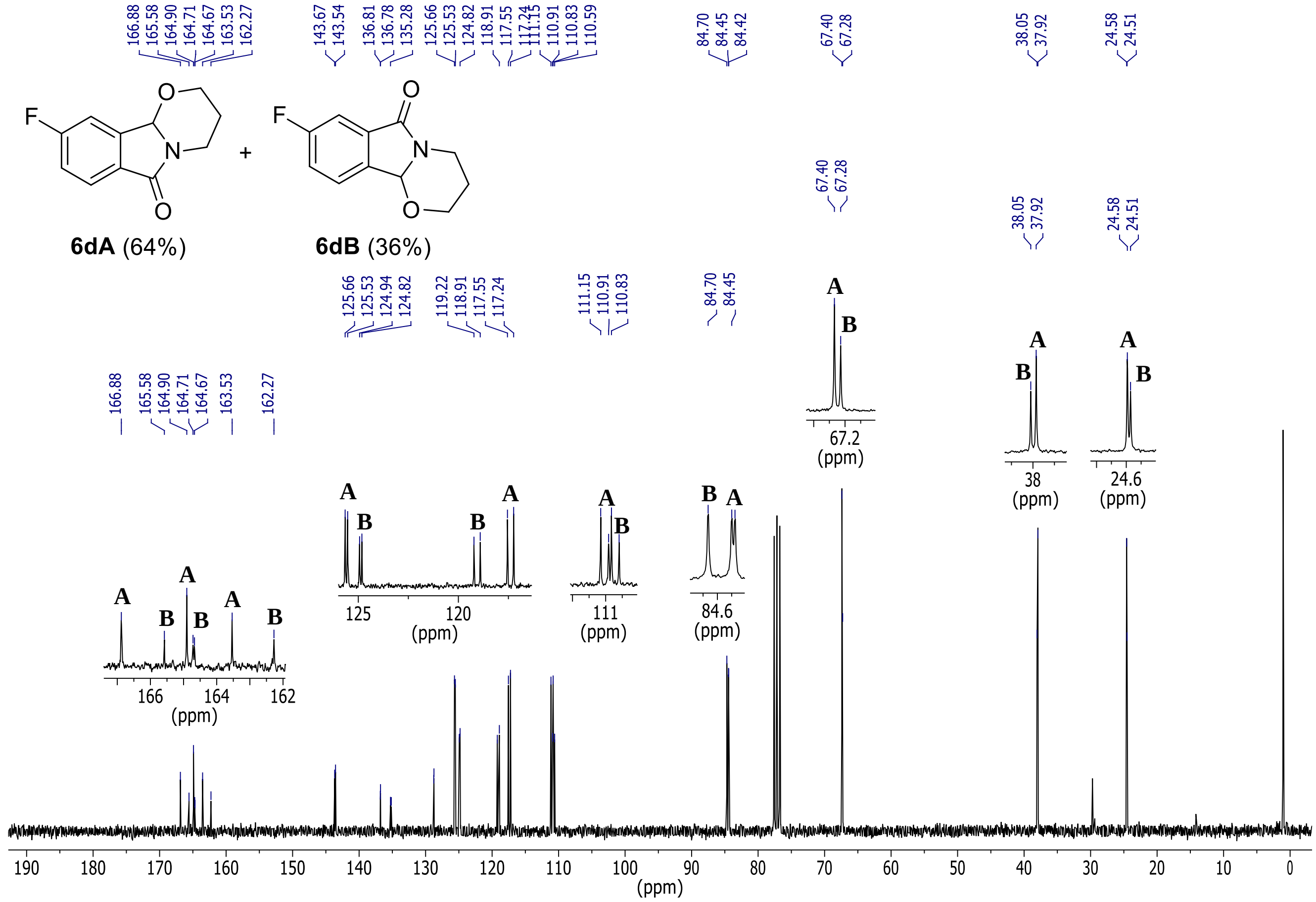

^{19}F NMR

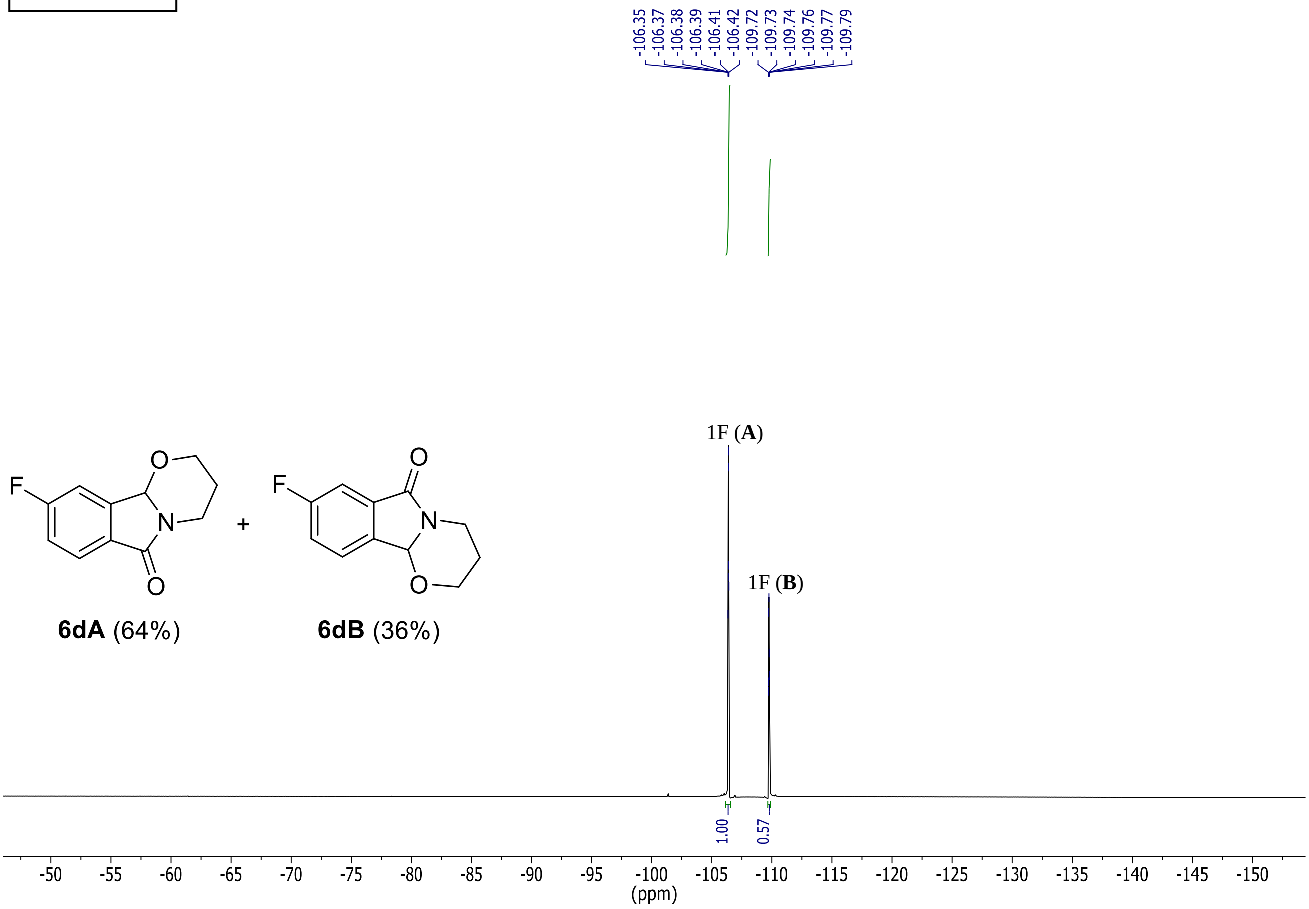

-106.35
-106.37
-106.38
-106.39
-106.41
-106.42
-109.72
-109.73
-109.74
-109.76
-109.77
-109.79
1F (**A**)
1F (**B**)
6dA (64%)
6dB (36%)
1.00
0.57
-50 -55 -60 -65 -70 -75 -80 -85 -90 -95 -100 -105 -110 -115 -120 -125 -130 -135 -140 -145 -150
(ppm)

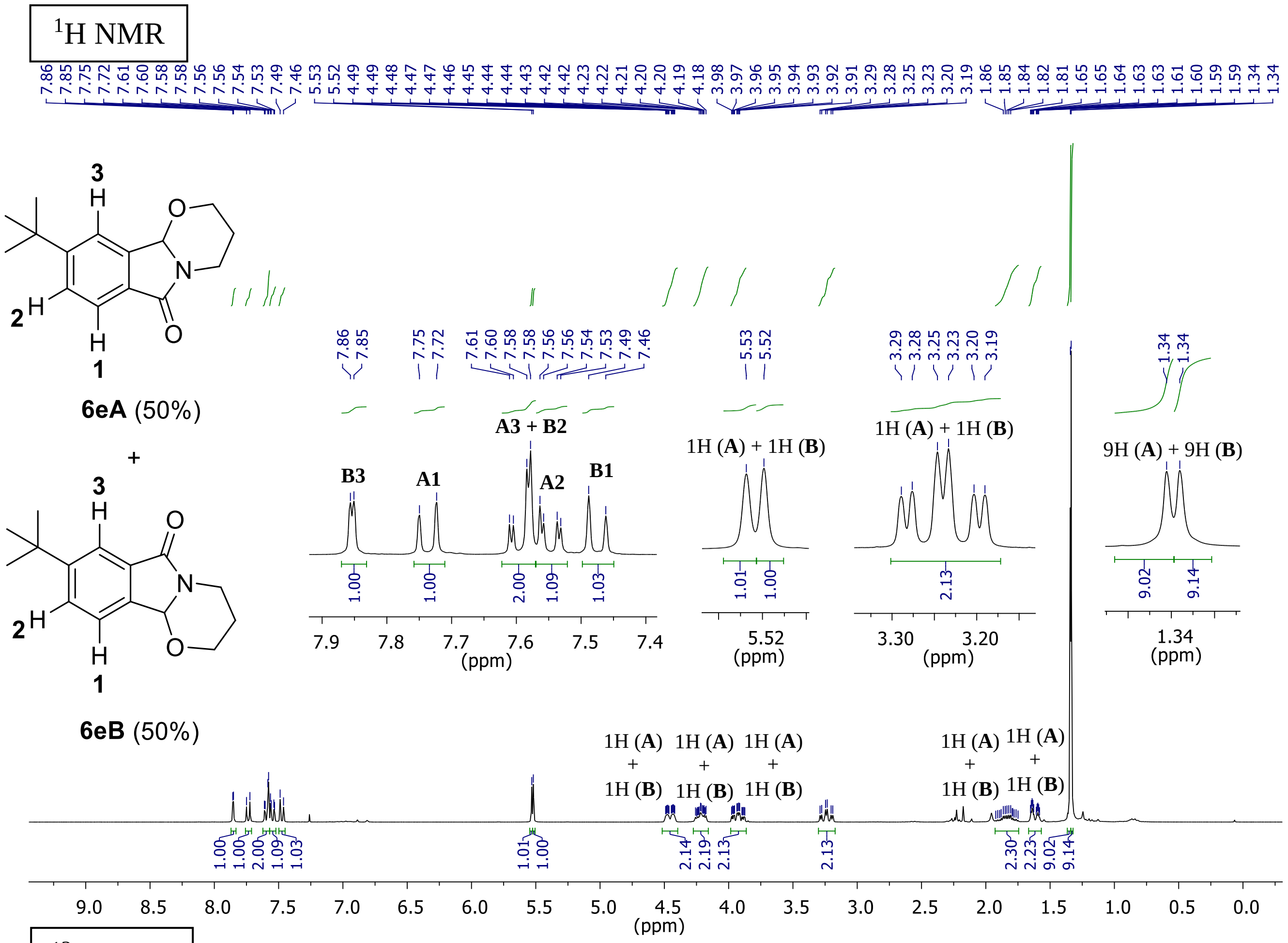

1H NMR
7.86 7.85 7.75 7.72 7.61 7.60 7.58 7.58 7.56 7.56 7.54 7.53 7.49 7.46 5.53 5.52 4.49 4.49 4.48 4.47 4.47 4.46 4.45 4.44 4.44 4.43 4.42 4.42 4.23 4.22 4.21 4.20 4.20 4.19 4.18 3.98 3.97 3.96 3.95 3.94 3.93 3.92 3.91 3.29 3.28 3.25 3.23 3.20 3.19 1.86 1.85 1.84 1.82 1.81 1.65 1.64 1.63 1.63 1.61 1.60 1.59 1.34 1.34
3
H
2 H
H
1
O
N
O
6eA (50%)
+
6eB (50%)
B3
A1
A3 + B2
A2
B1
1H (A) + 1H (B)
1H (A) + 1H (B)
9H (A) + 9H (B)
1.00 1.00 2.00 1.09 1.03
1.01 1.00
2.13
9.02 9.14
7.9 7.8 7.7 7.6 7.5 7.4
(ppm)
5.52
(ppm)
3.30 3.20
(ppm)
1.34
(ppm)
1H (A) + 1H (B)
1H (A) + 1H (B)
1H (A) + 1H (B)
1H (A) + 1H (B)
1H (A) + 1H (B)
2.14 2.19 2.13 2.13 2.30 2.23 9.02 9.14
9.0 8.5 8.0 7.5 7.0 6.5 6.0 5.5 5.0 4.5 4.0 3.5 3.0 2.5 2.0 1.5 1.0 0.5 0.0
(ppm)

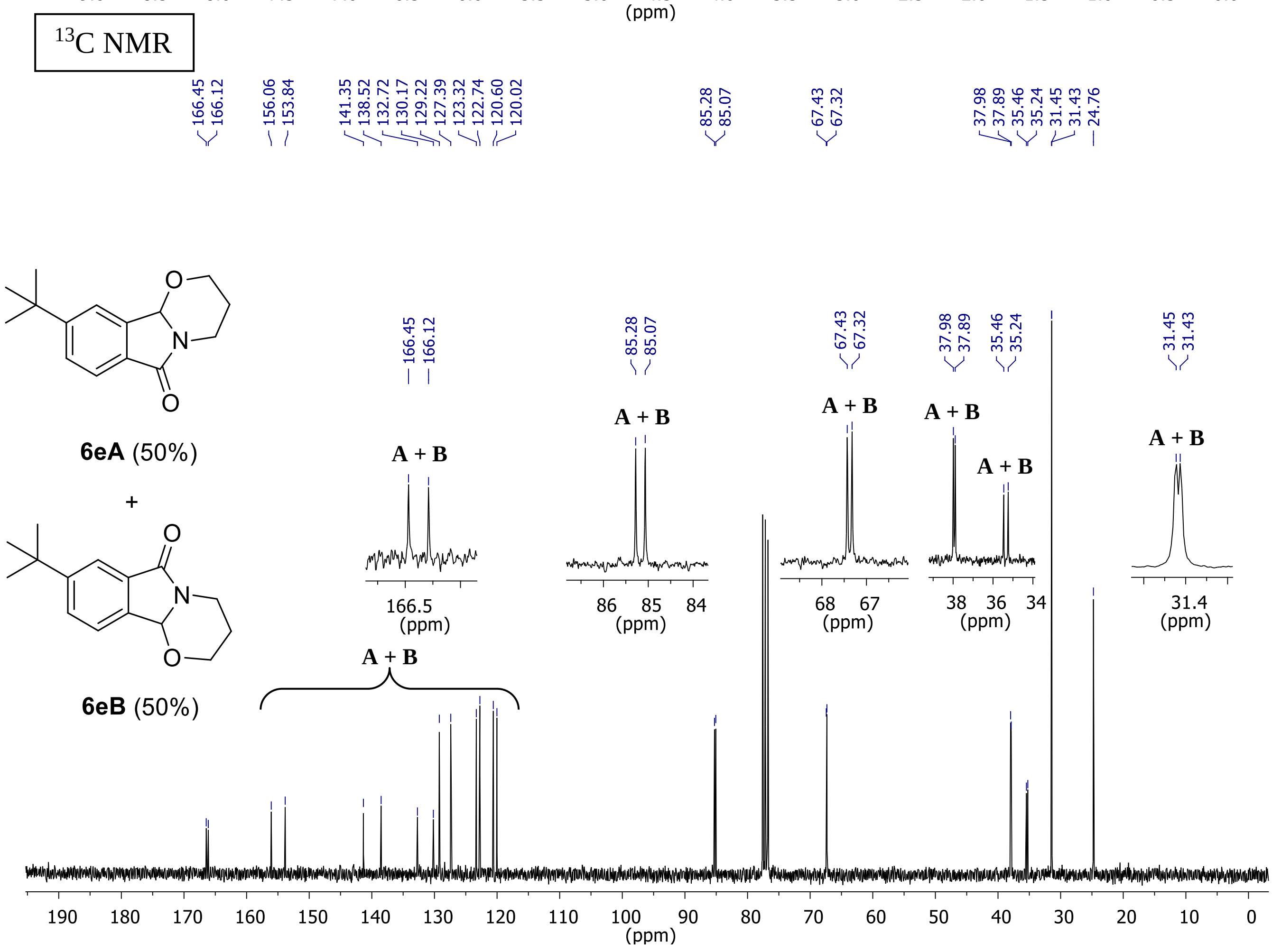

13C NMR
166.45 166.12 156.06 153.84 141.35 138.52 132.72 130.17 129.22 127.39 123.32 122.74 120.60 120.02 85.28 85.07 67.43 67.32 37.98 37.89 35.46 35.24 31.45 31.43 24.76
O
N
O
6eA (50%)
+
6eB (50%)
A + B
166.5
(ppm)
86 85 84
(ppm)
68 67
(ppm)
38 36 34
(ppm)
31.4
(ppm)
190 180 170 160 150 140 130 120 110 100 90 80 70 60 50 40 30 20 10 0
(ppm)

^{1}H NMR

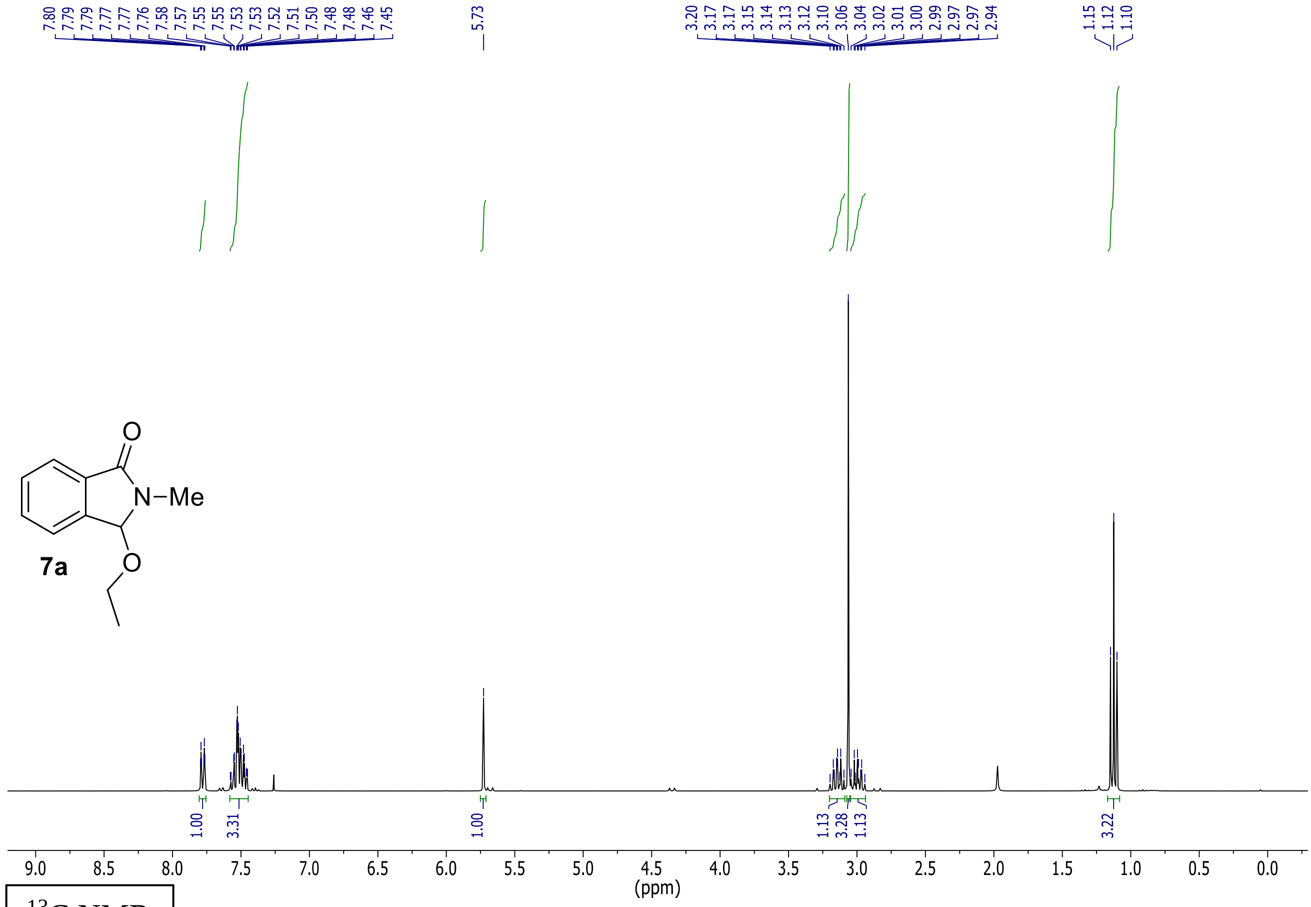

^{13}C NMR

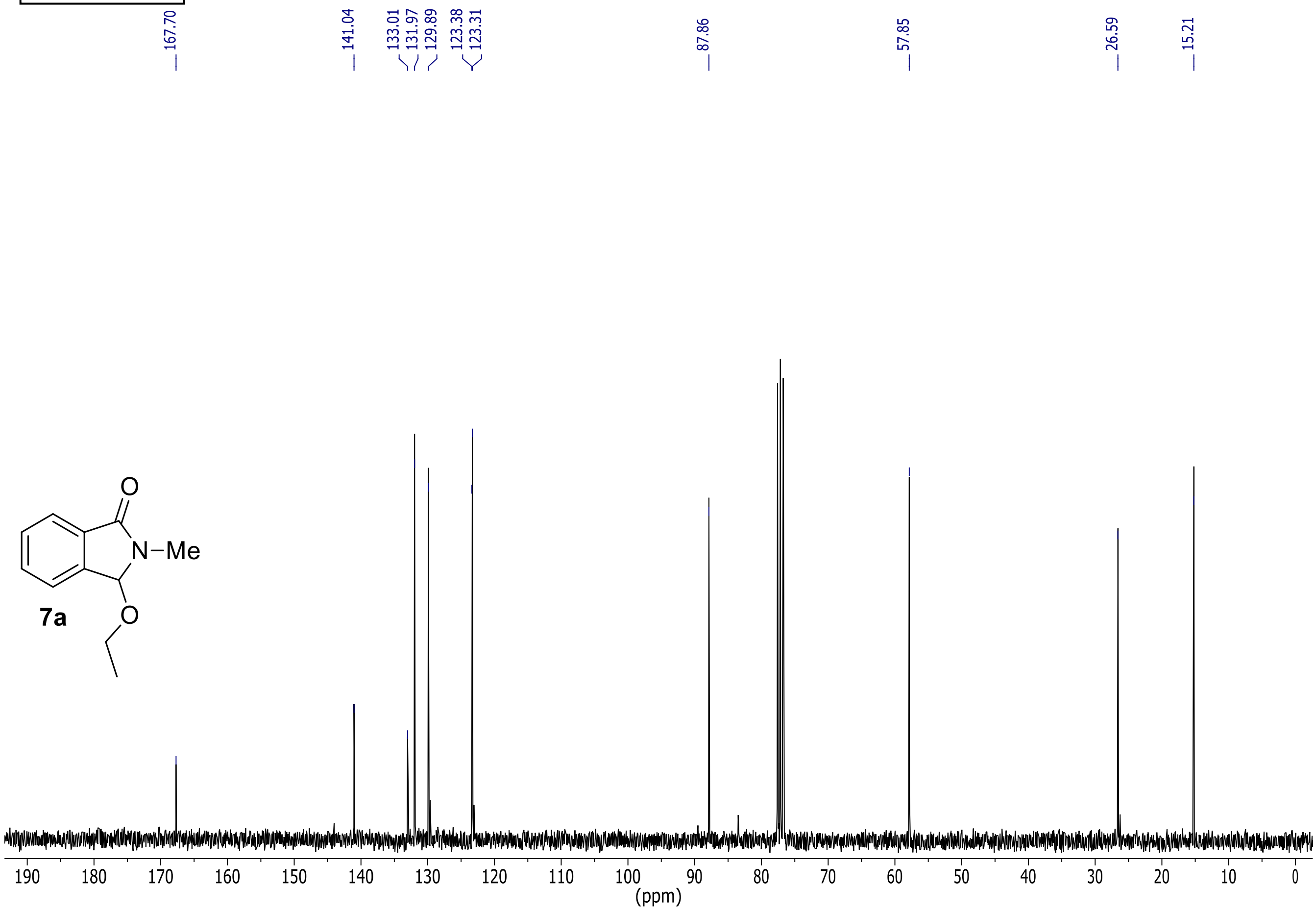

^{1}H NMR

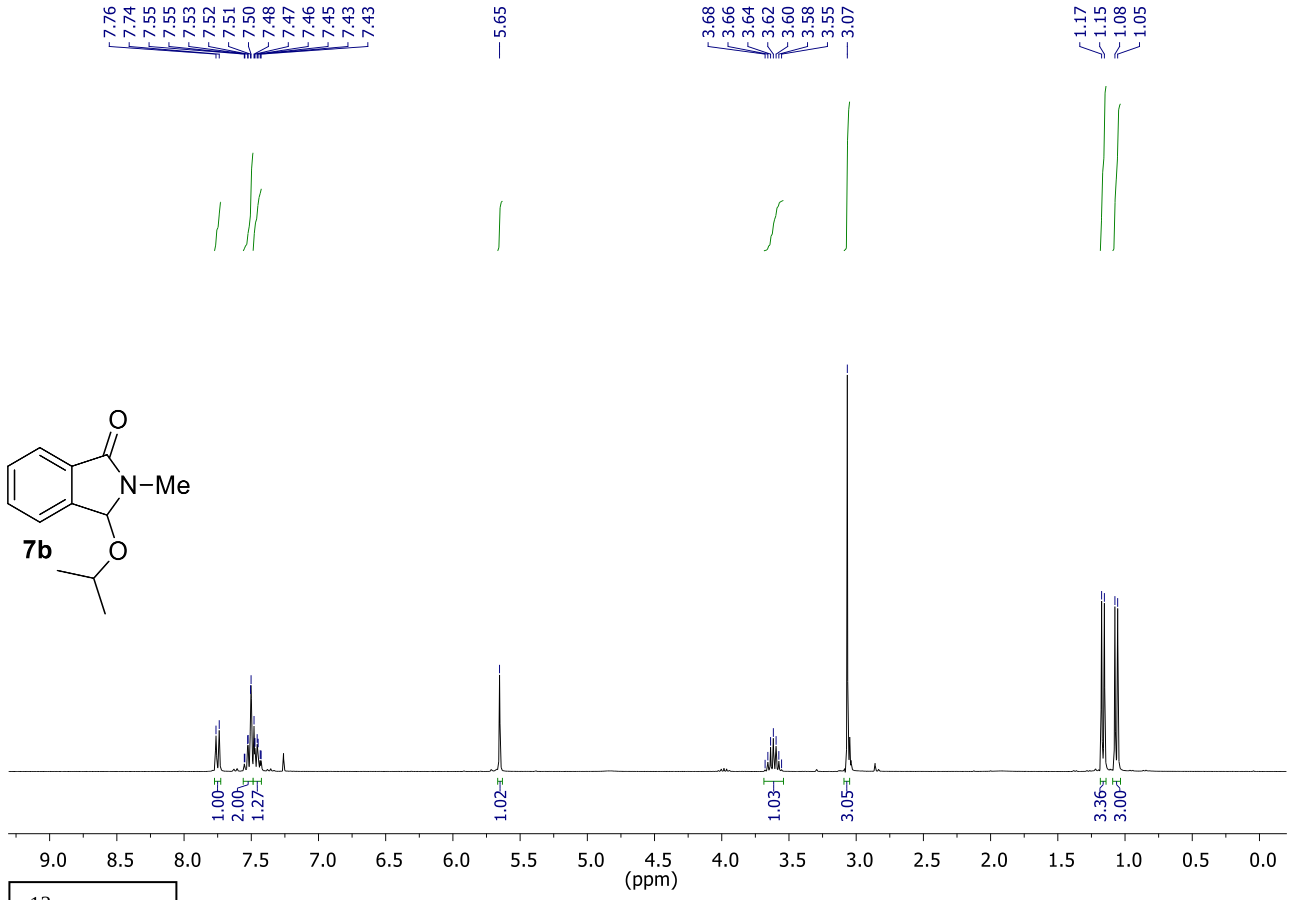

^{13}C NMR

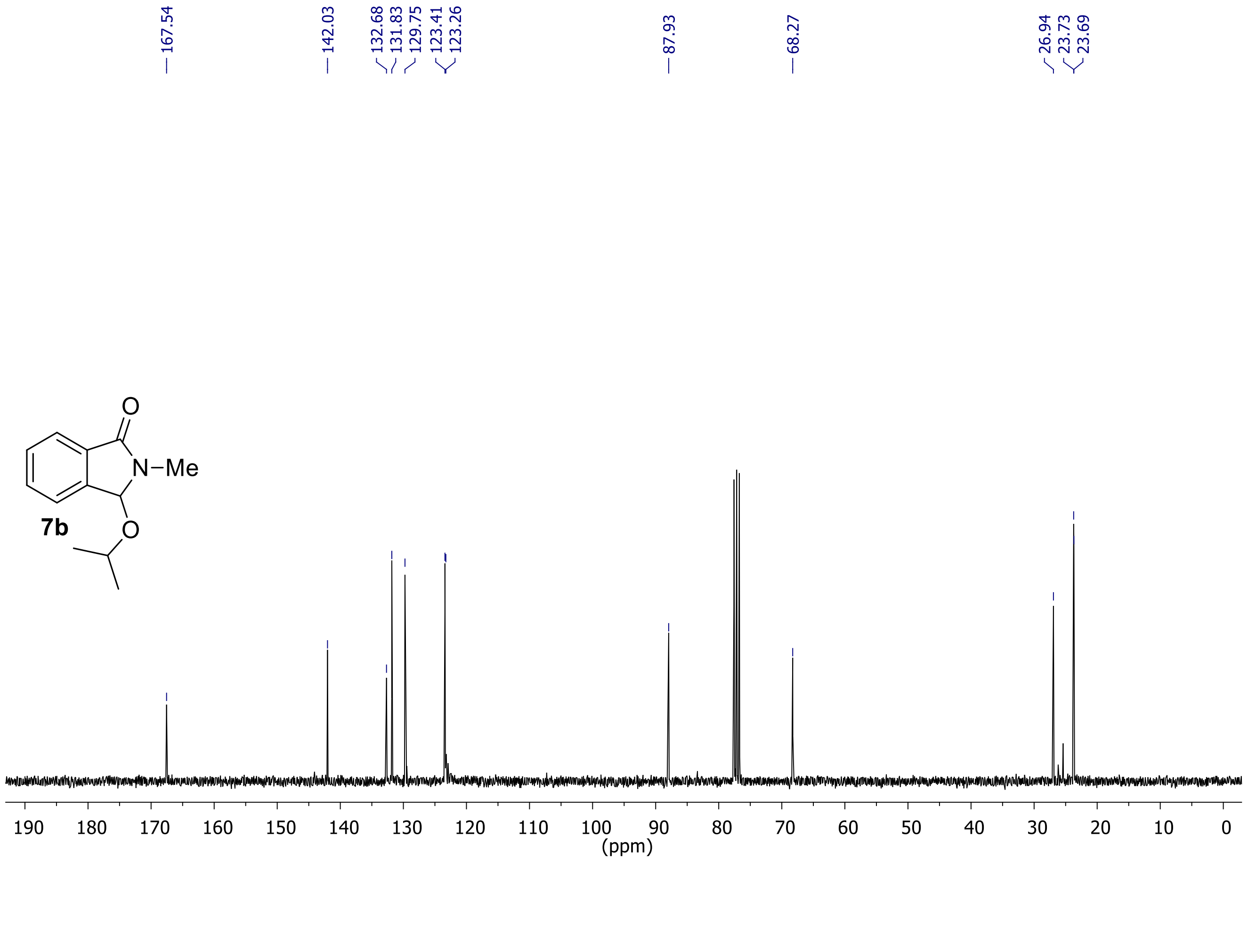

^1H NMR

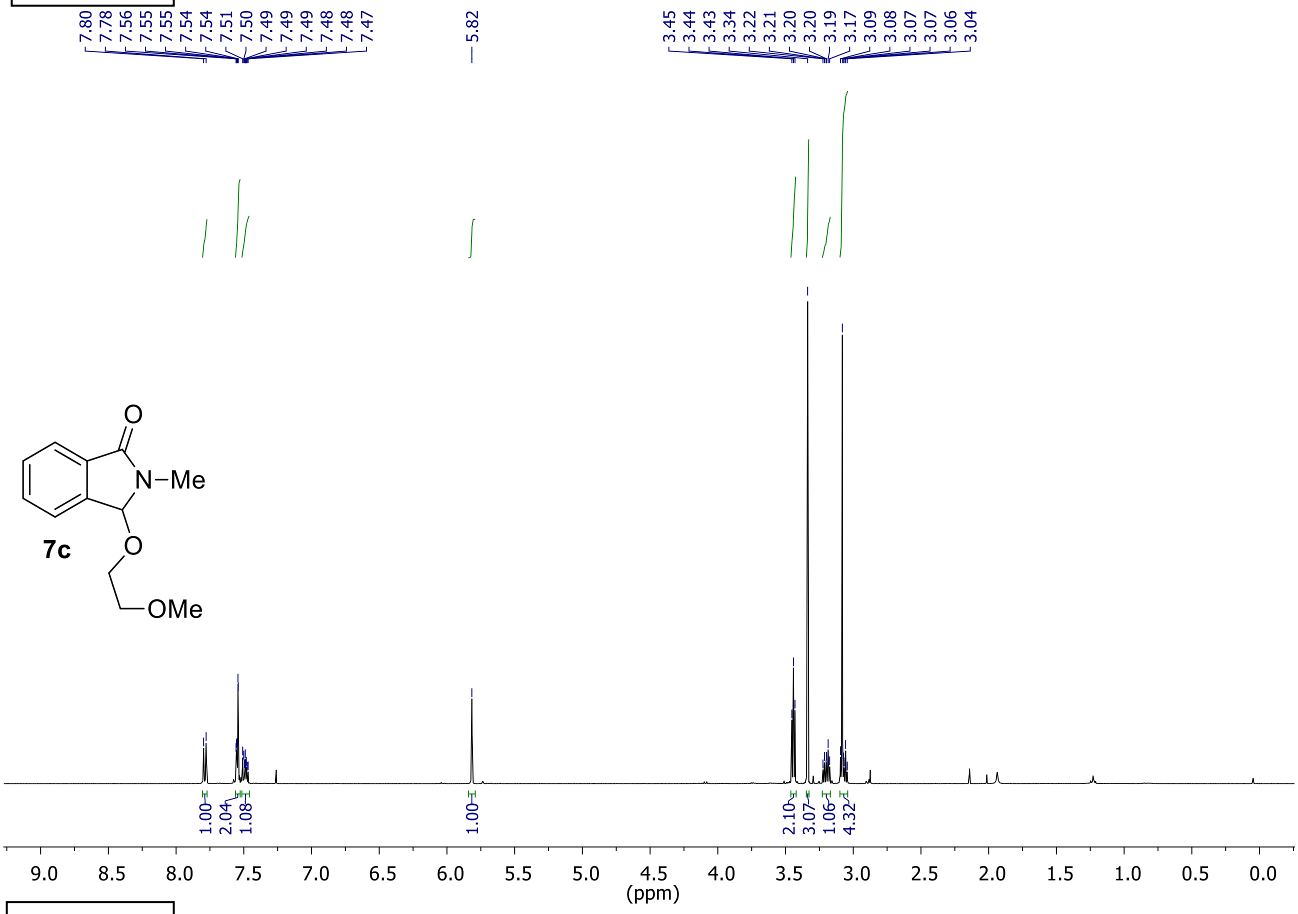

7c

^{13}C NMR

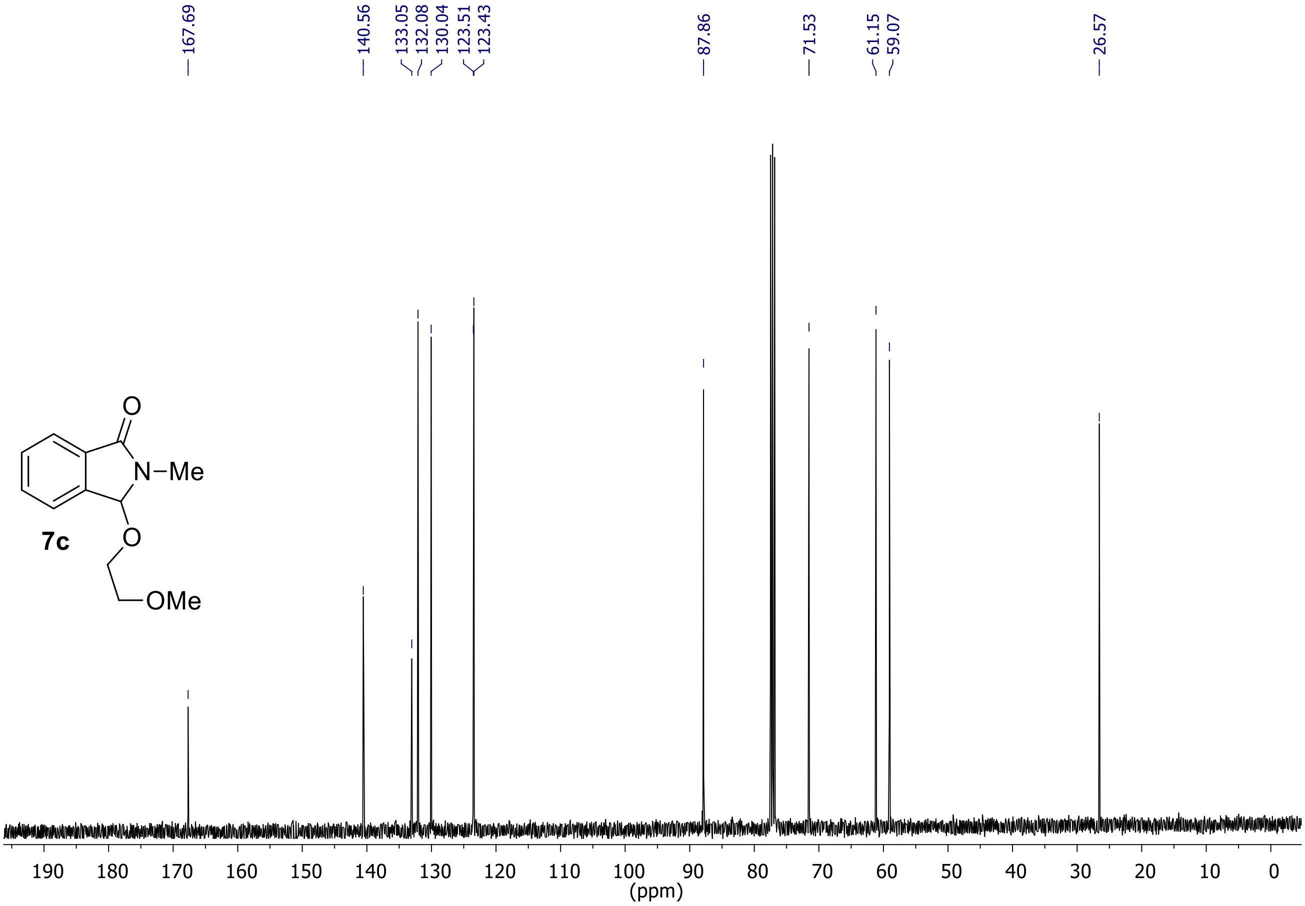

7c

^{1}H NMR

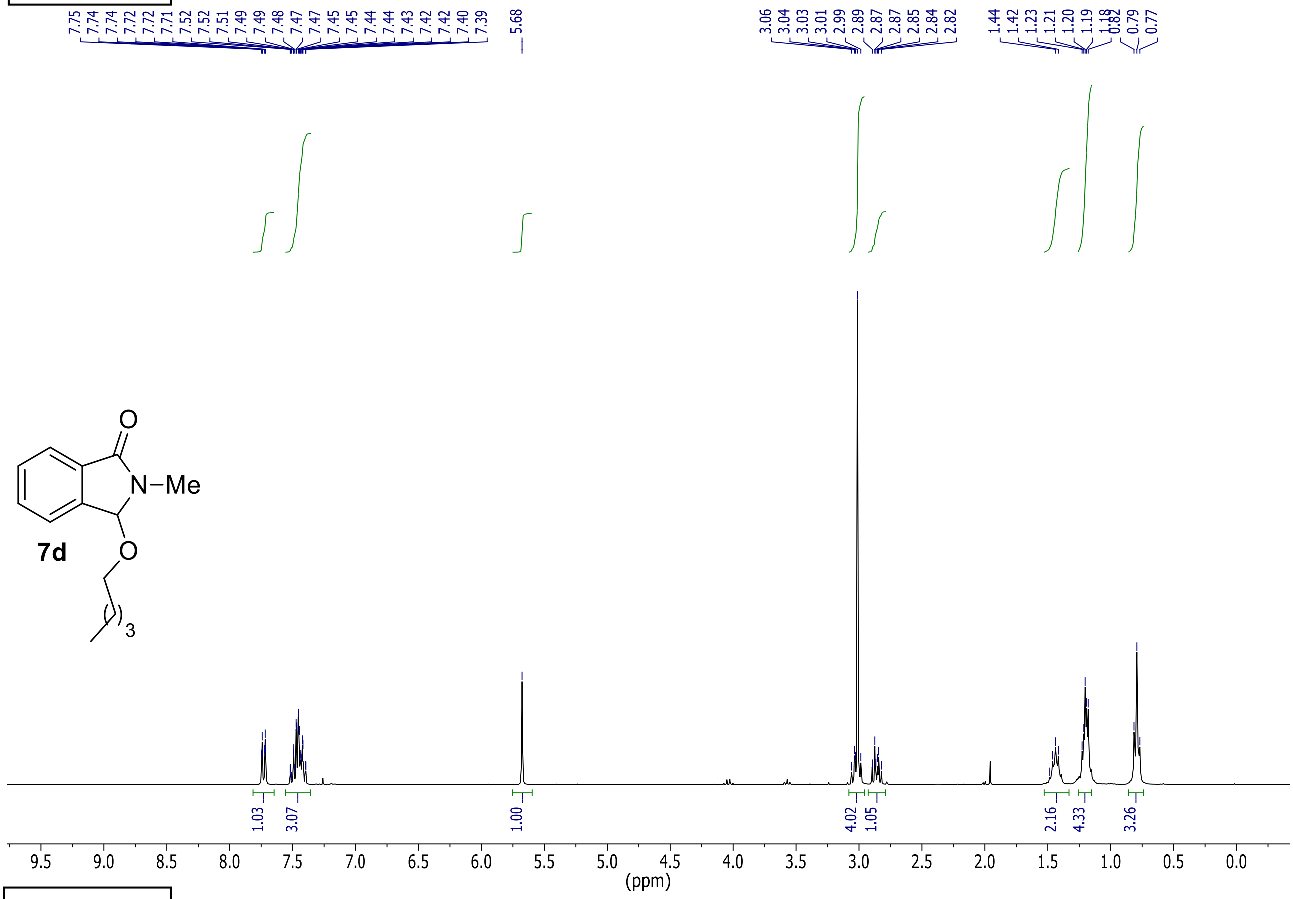

^{13}C NMR

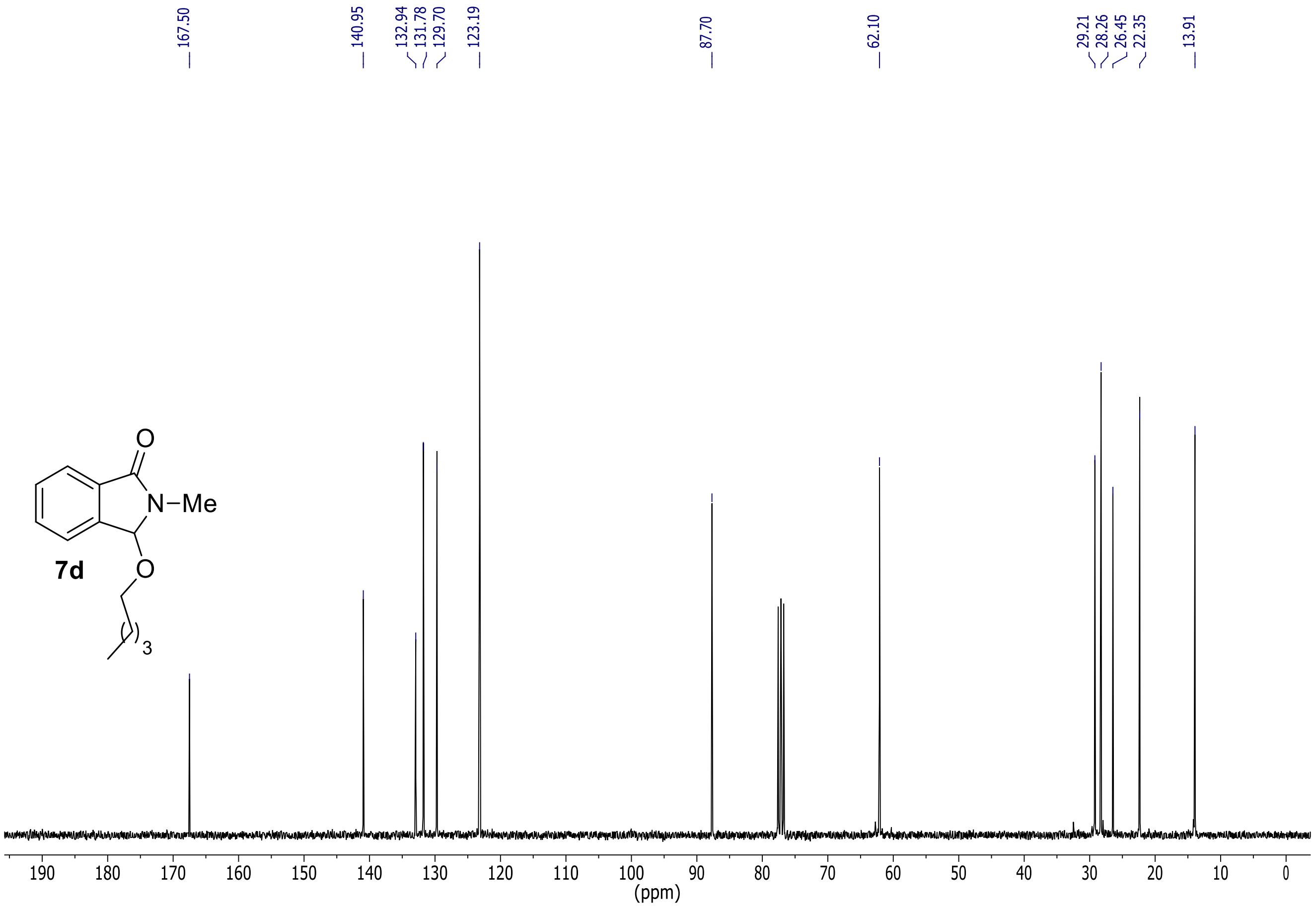

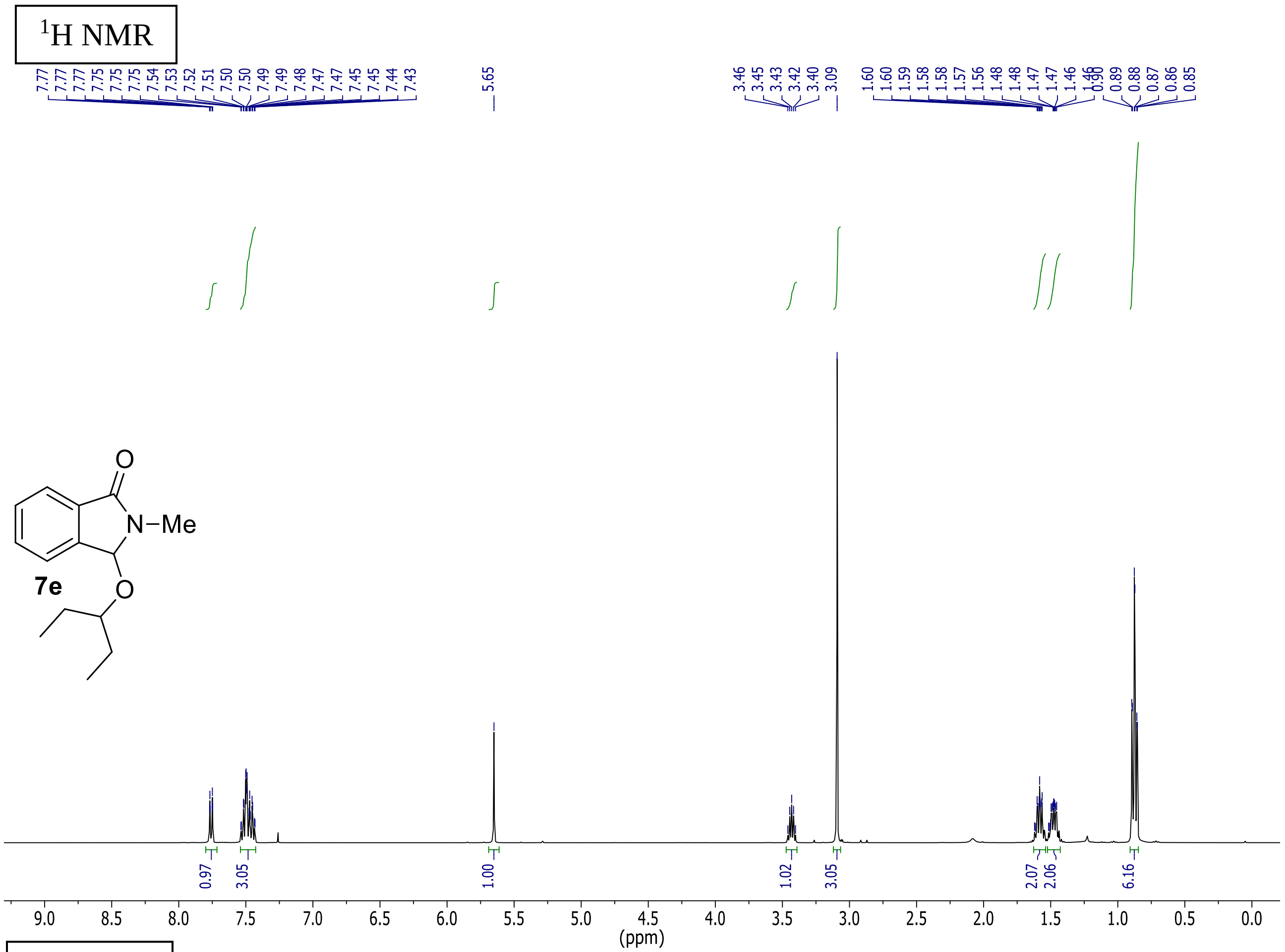

^{1}H NMR
7.77 7.77 7.77 7.75 7.75 7.75 7.54 7.53 7.52 7.51 7.50 7.50 7.49 7.48 7.47 7.47 7.45 7.45 7.44 7.43
5.65
3.46 3.45 3.43 3.42 3.40 3.09
1.60 1.60 1.59 1.58 1.58 1.57 1.56 1.48 1.48 1.47 1.47 1.46 1.46 0.90 0.89 0.88 0.87 0.86 0.85
O
N−Me
7e
O
0.97 3.05 1.00 1.02 3.05 2.07 2.06 6.16
9.0 8.5 8.0 7.5 7.0 6.5 6.0 5.5 5.0 4.5 4.0 3.5 3.0 2.5 2.0 1.5 1.0 0.5 0.0
(ppm)

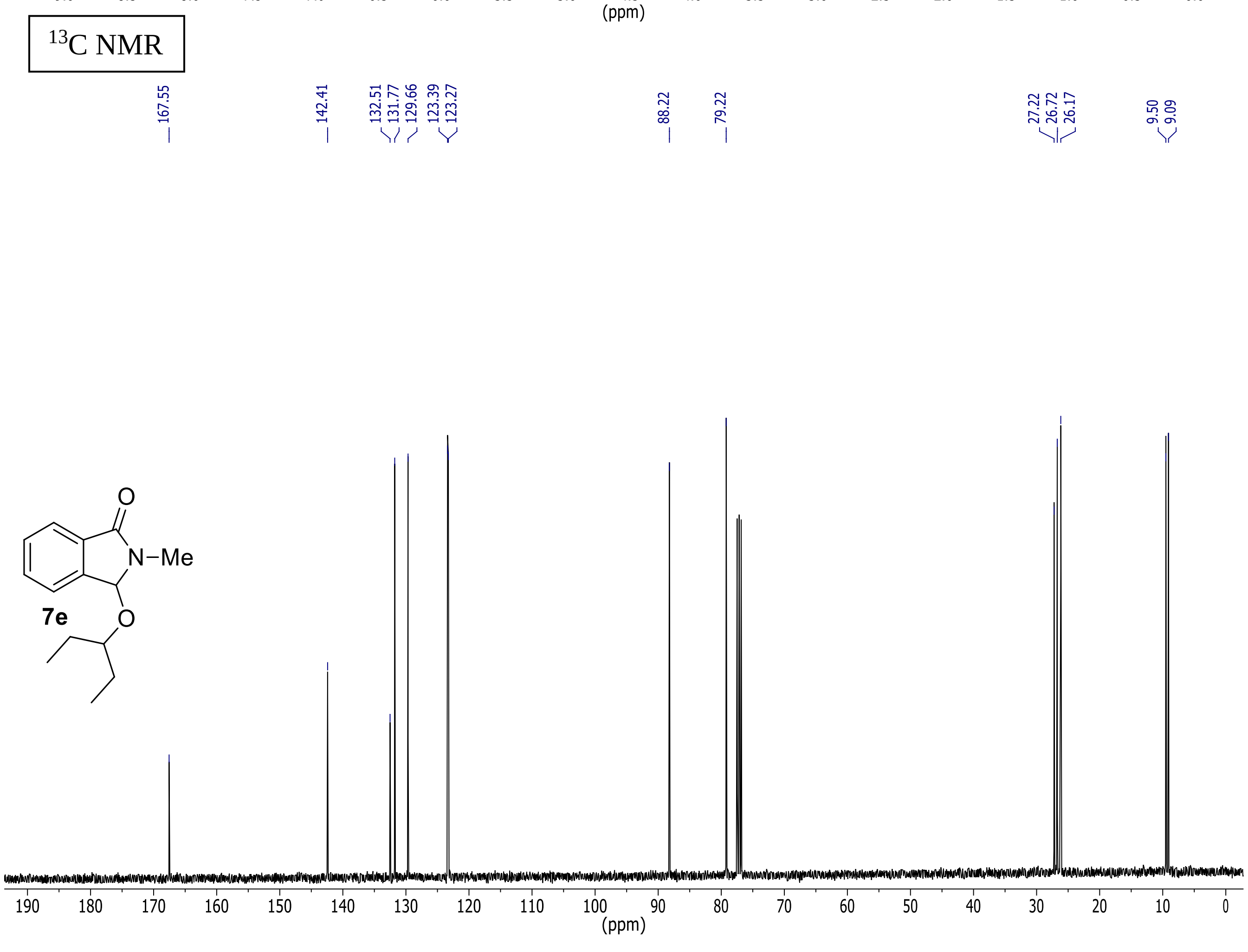

^{13}C NMR
167.55
142.41
132.51 131.77 129.66 123.39 123.27
88.22
79.22
27.22 26.72 26.17
9.50 9.09
O
N−Me
7e
O
190 180 170 160 150 140 130 120 110 100 90 80 70 60 50 40 30 20 10 0
(ppm)

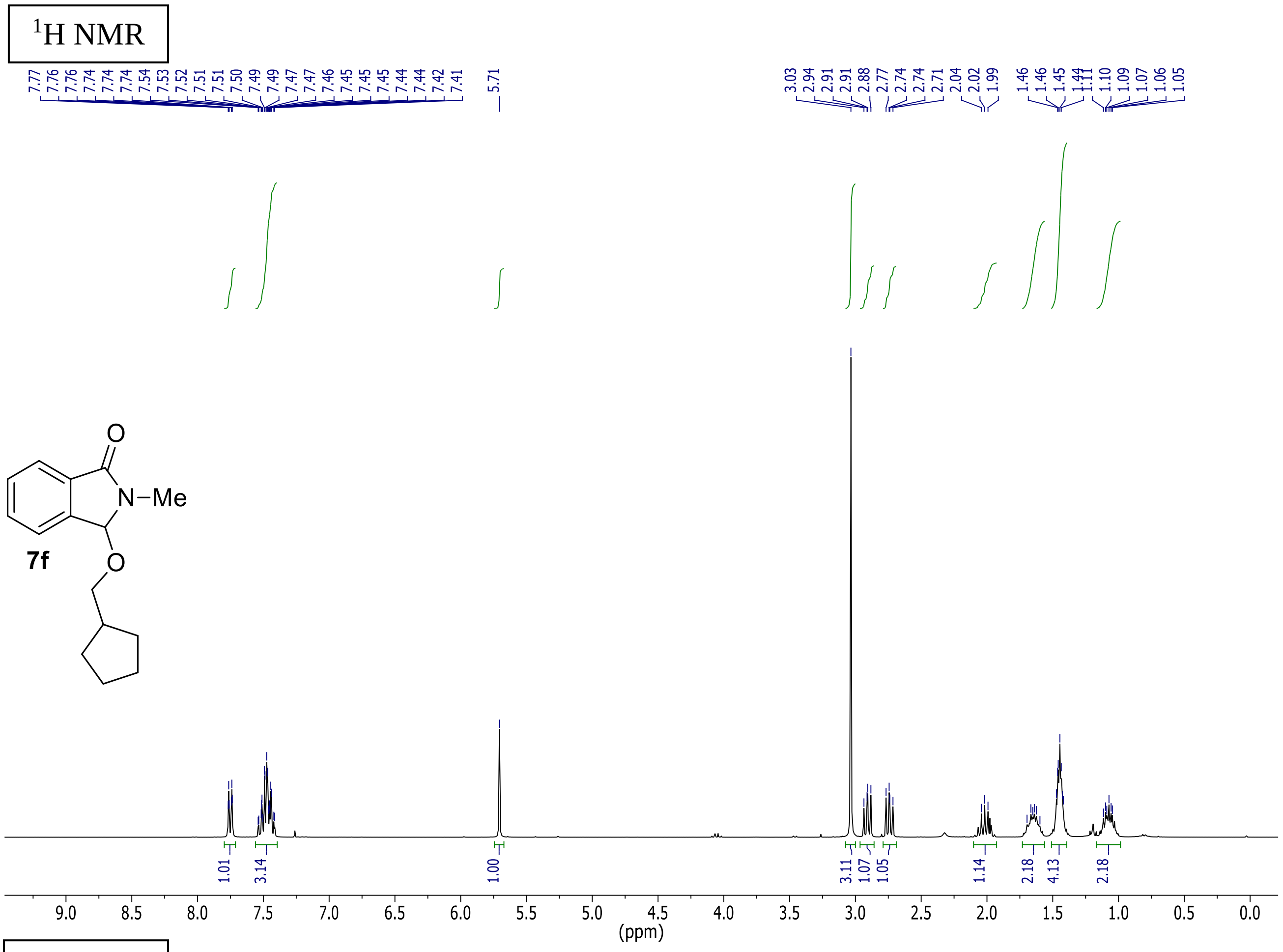

1H NMR
7.77 7.76 7.76 7.74 7.74 7.54 7.53 7.52 7.51 7.51 7.50 7.49 7.49 7.47 7.47 7.46 7.45 7.45 7.45 7.44 7.44 7.42 7.41
5.71
3.03 2.94 2.91 2.91 2.88 2.77 2.74 2.74 2.71 2.04 2.02 1.99
1.46 1.46 1.45 1.44 1.11 1.10 1.09 1.07 1.06 1.05
N−Me
7f
1.01 3.14 1.00 3.11 1.07 1.05 1.14 2.18 4.13 2.18
9.0 8.5 8.0 7.5 7.0 6.5 6.0 5.5 5.0 4.5 4.0 3.5 3.0 2.5 2.0 1.5 1.0 0.5 0.0
(ppm)

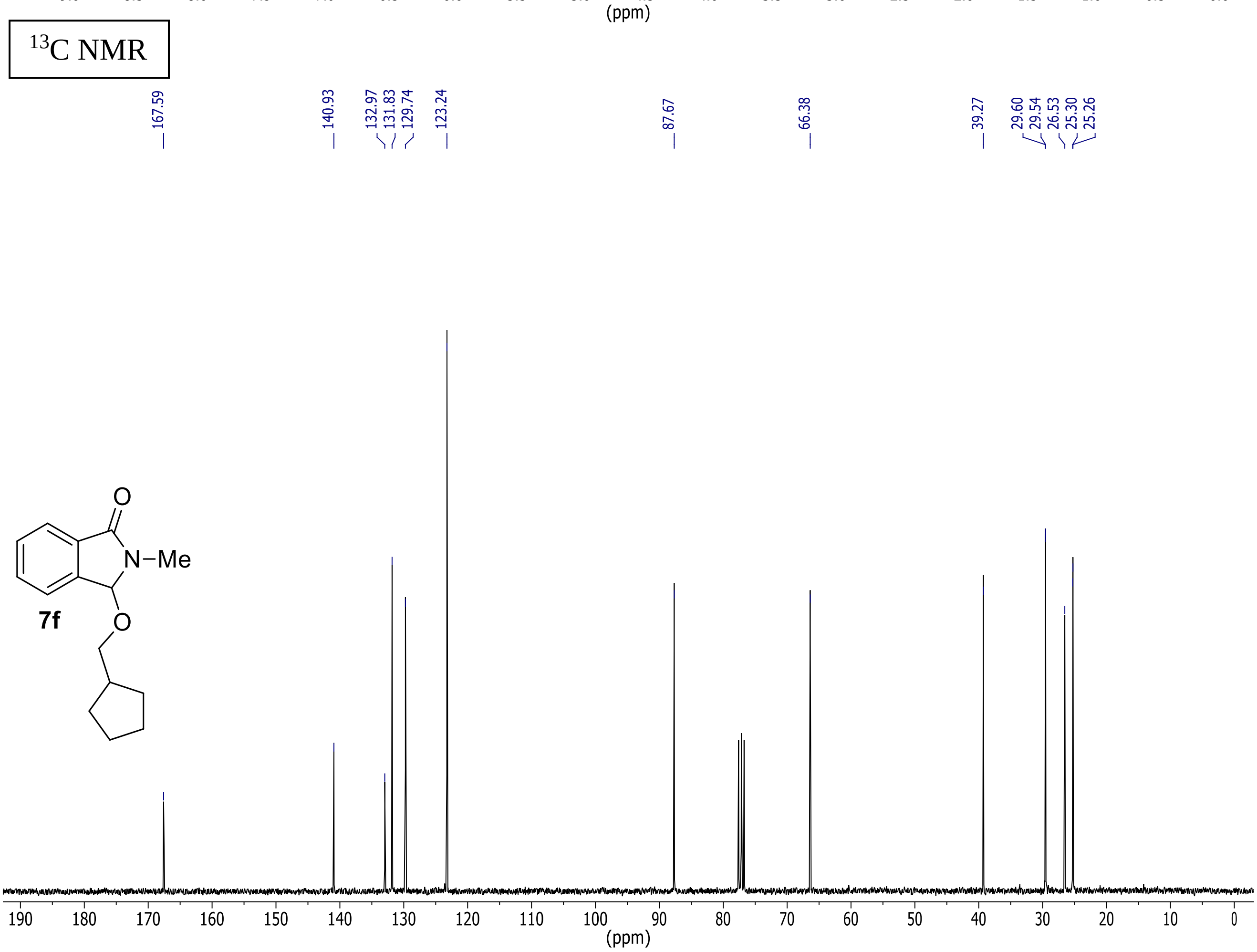

13C NMR
167.59
140.93
132.97 131.83 129.74
123.24
87.67
66.38
39.27
29.60 29.54 26.53 25.30 25.26
N−Me
7f
190 180 170 160 150 140 130 120 110 100 90 80 70 60 50 40 30 20 10 0
(ppm)

^{1}H NMR

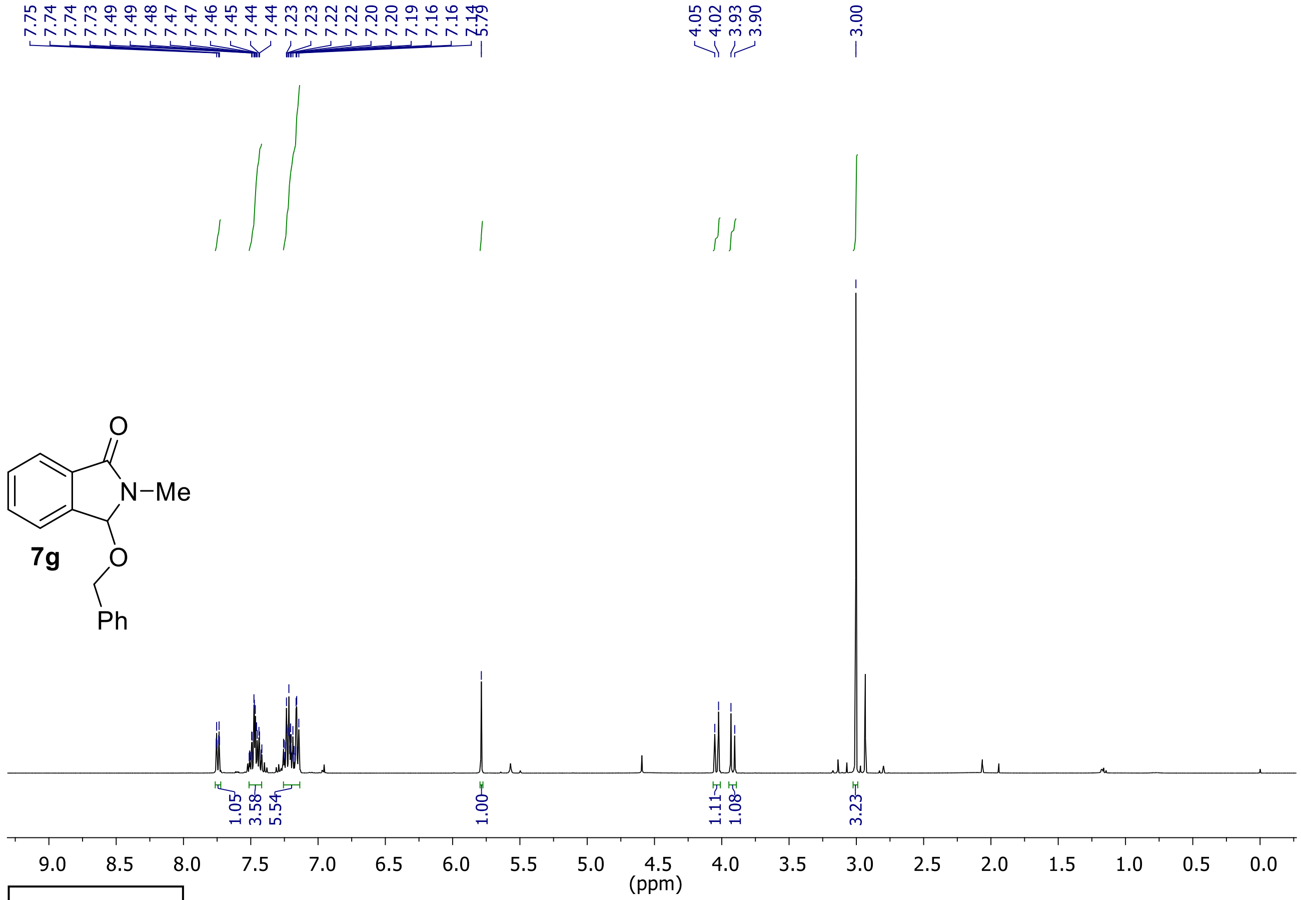

^{13}C NMR

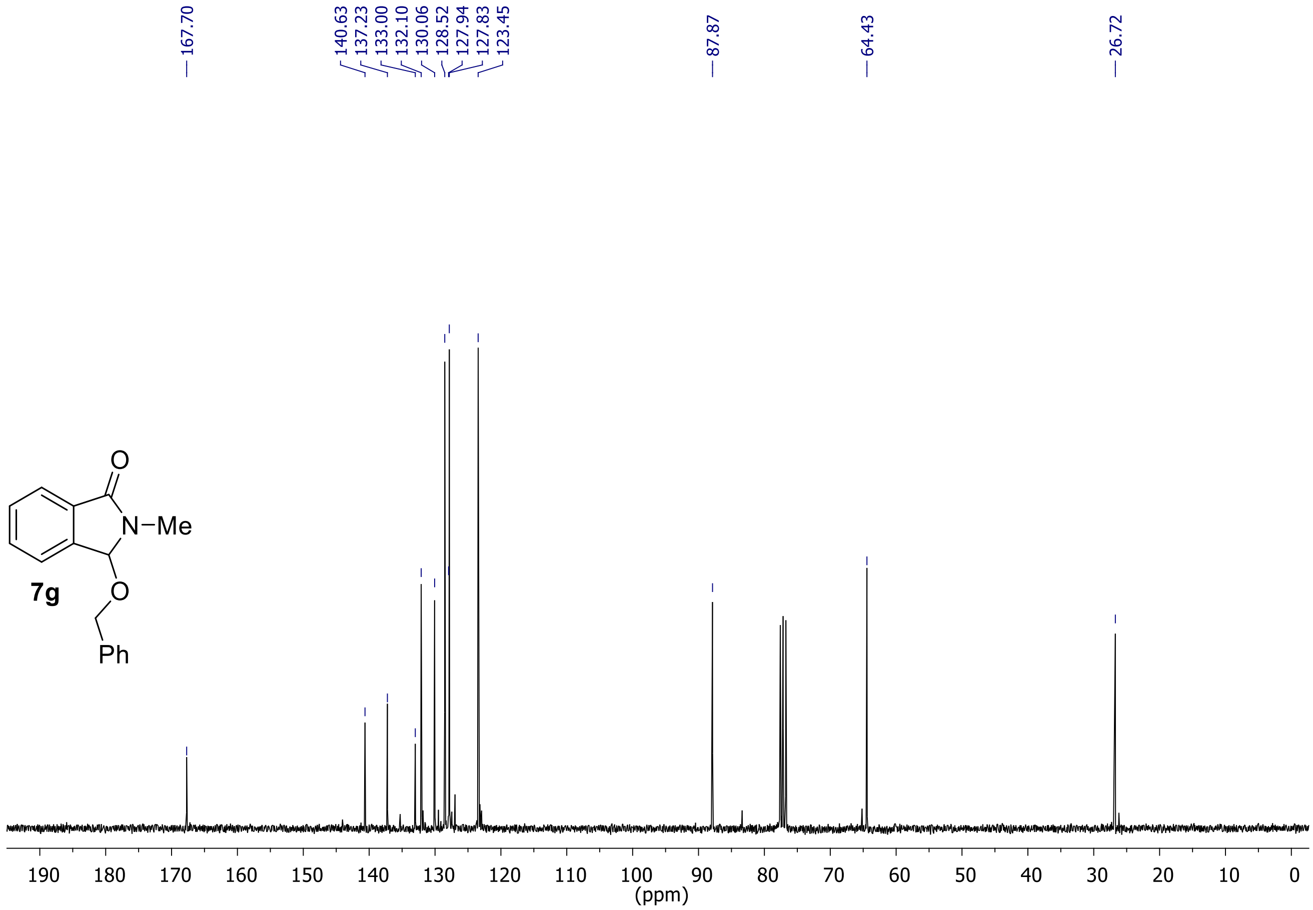

^{1}H NMR

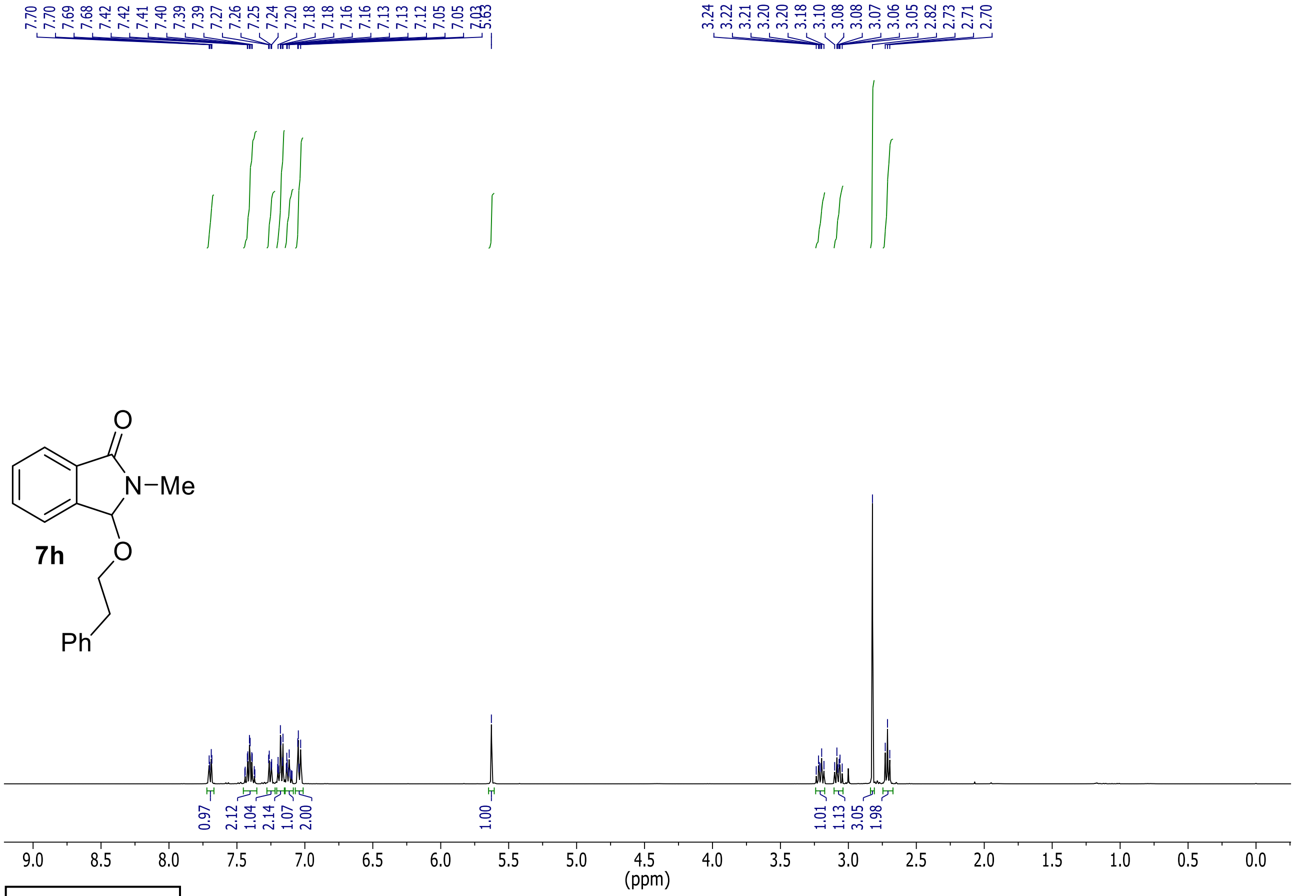

^{13}C NMR

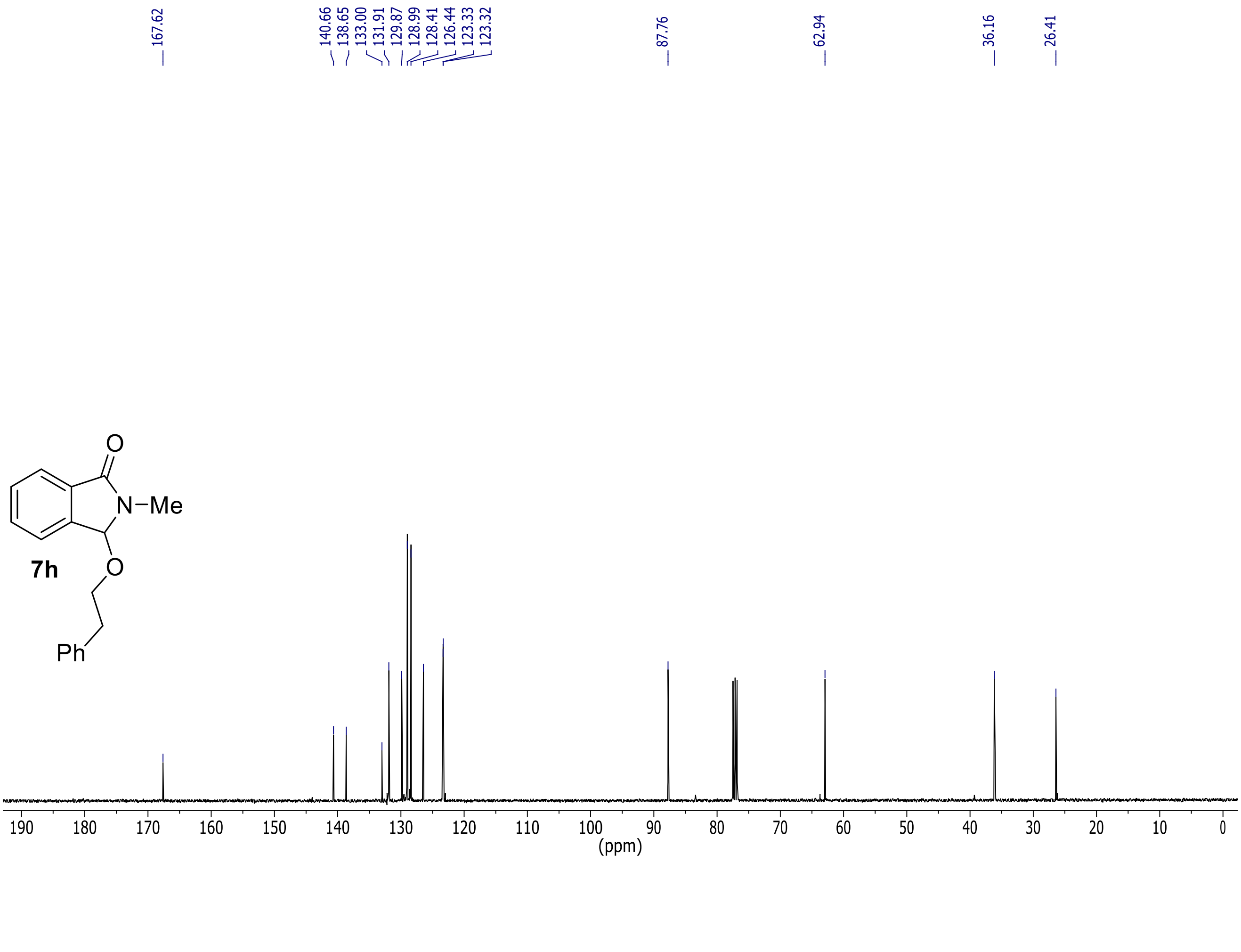

^{1}H NMR

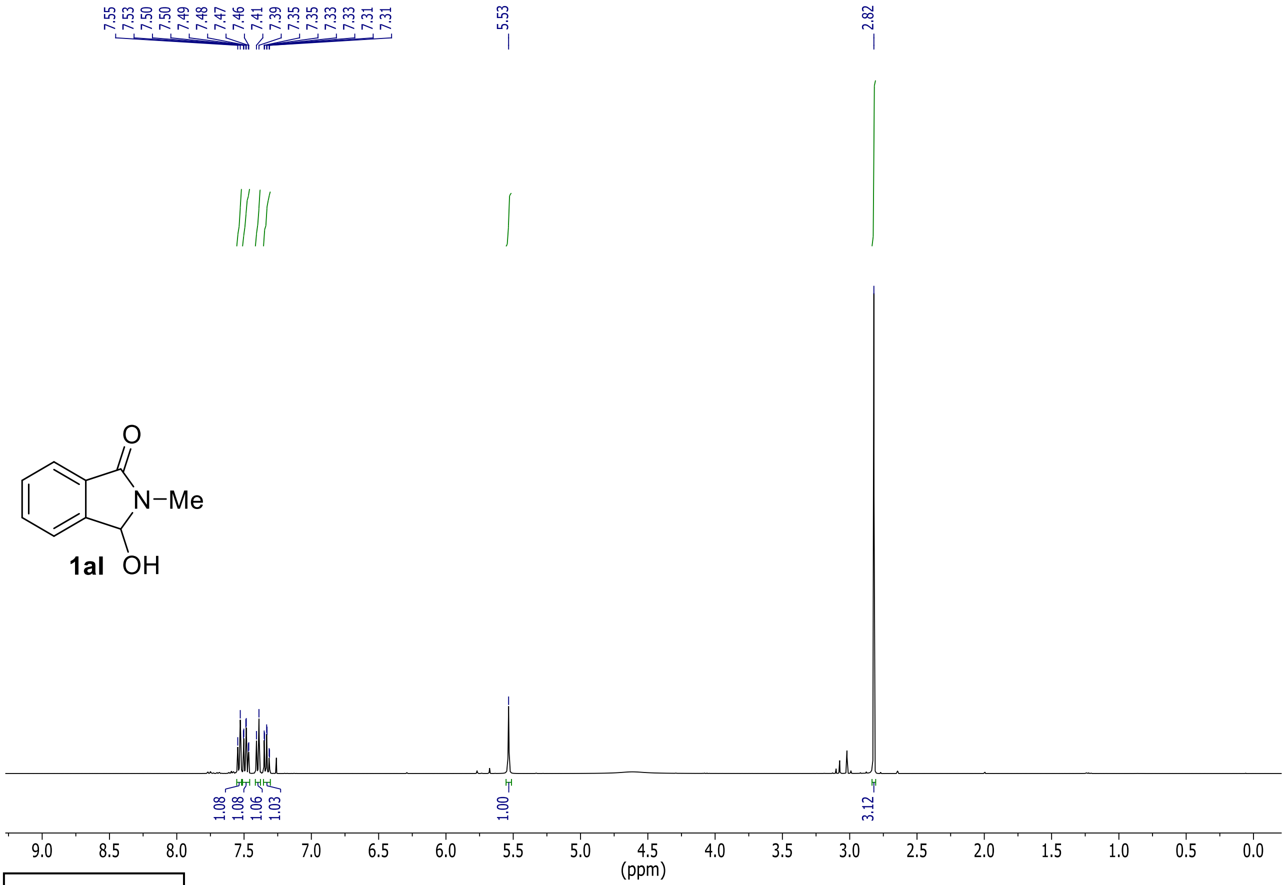

^{13}C NMR

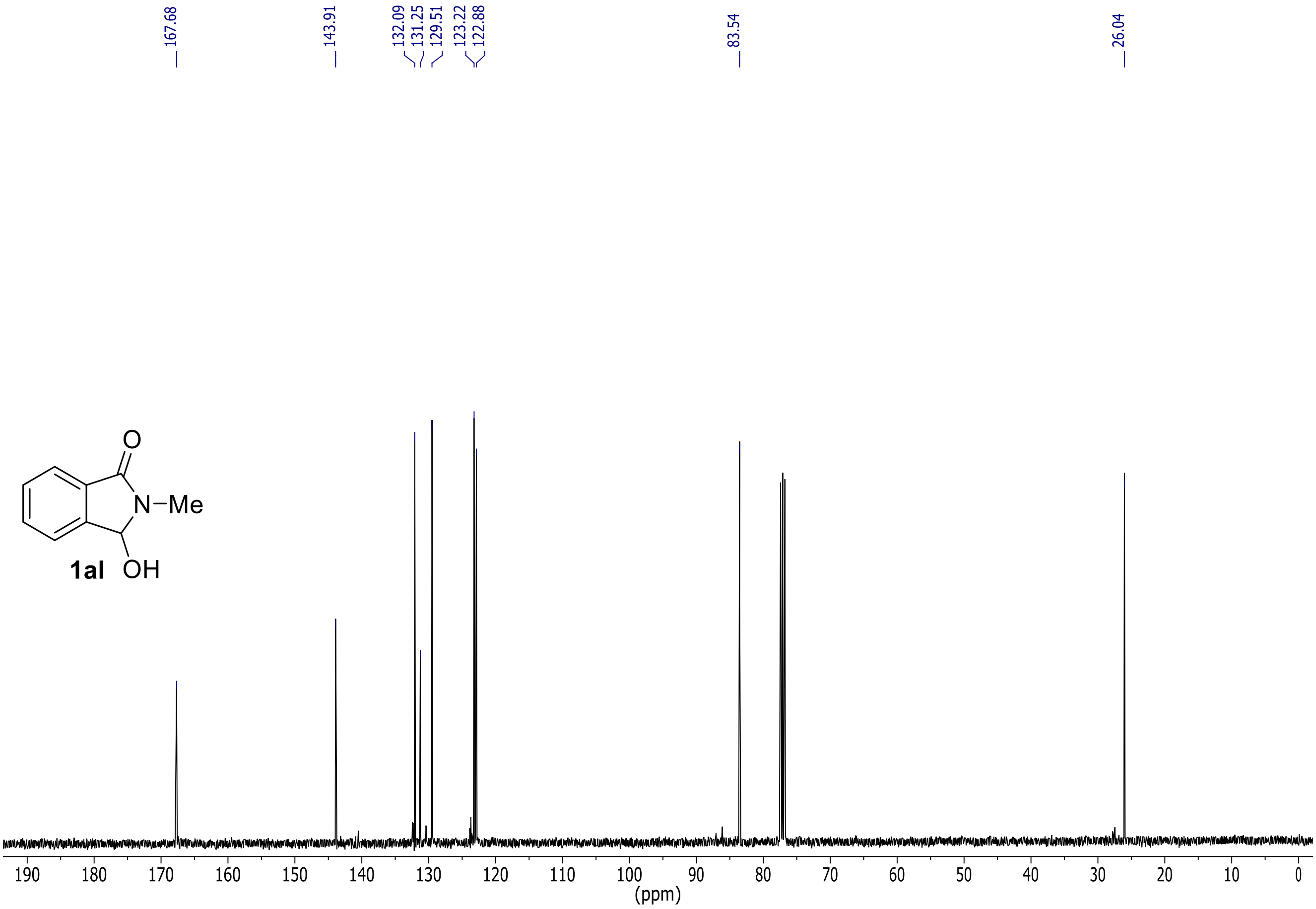

Supplement: Supplementary file 1 [file SC-008-C7SC01175J-s001.pdf]
